# Guanylate-Binding protein 2b regulates the AMPK/mTOR/ULK1 signalling pathway to induce autophagy during *Mycobacterium bovis* infection

**DOI:** 10.1080/21505594.2022.2073024

**Published:** 2022-05-21

**Authors:** Youli Yu, Jialiang Pan, Mengting Liu, Haiqin Jiang, Jingshu Xiong, Lei Tao, Feng Xue, Fang Tang, Hongsheng Wang, Jianjun Dai

**Affiliations:** a MOE Joint International Research Laboratory of Animal Health and Food Safety, College of Veterinary Medicine, Nanjing Agricultural University, Nanjing, China; b Institute of Dermatology, Chinese Academy of Medical Sciences and Peking Union Medical College, Nanjing, China; c Nanjing Institute for Food and Drug Control, Nanjing, China; d China Pharmaceutical University, Nanjing, China

**Keywords:** *Mycobacterium bovis*, RNA-Seq, GBP2b, autophagy, AMPK, mTOR, ULK1

## Abstract

Autophagic isolation and degradation of intracellular pathogens are employed by host cells as primary innate immune defense mechanisms to control intercellular *M. bovis* infection. In this study, RNA-Seq technology was used to obtain the total mRNA from bone marrow-derived macrophages (BMDMs) infected with *M. bovis* at 6 and 24 h after infection. One of the differential genes, GBP2b, was also investigated. Analysis of the significant pathway involved in GBP2b-coexpressed mRNA demonstrated that GBP2b was associated with autophagy and autophagy-related mammalian target of rapamycin (mTOR) signaling and AMP-activated protein kinase (AMPK) signaling. The results of in vivo and in vitro experiments showed significant up-regulation of GBP2b during *M. bovis* infection. For in vitro validation, small interfering RNA-GBP2b plasmids were transfected into BMDMs and RAW264.7 cells lines to down-regulate the expression of GBP2b. The results showed that the down-regulation of GBP2b impaired autophagy via the AMPK/mTOR/ULK1 pathway, thereby promoting the intracellular survival of *M. bovis*. Further studies revealed that the activation of AMPK signaling was essential for the regulation of autophagy during *M. bovis* infection. These findings expand the understanding of how GBP2b regulates autophagy and suggest that GBP2b may be a potential target for the treatment of diseases caused by *M. bovis*.

## Introduction

*Mycobacterium bovis* belongs to the *M. tuberculosis* complex and is the main pathogen causing bovine tuberculosis. *M. bovis* is more than 99% genetically homologous to *M. tuberculosis* and is also a common cause of human tuberculosis, in addition to *M. tuberculosis*. Tuberculosis led by *M. bovis* has caused enormous economic losses and health risks to farm communities and humans in many countries [1–3]. Therefore, new treatment strategies should be developed to control *M. bovis* infections in humans and animals.

Mycobacterial complex species persist in the mononuclear phagocytes of the hosts, particularly in macrophages, by disrupting their protective immune response. Macrophages are key mononuclear phagocytes that regulate the cytoprotective immune response to eliminate intracellular pathogens [4,5]. Macrophages have developed numerous defense strategies against intracellular bacterial infection, including the induction of nitrogen intermediates and reactive oxygen species; the establishment of an acidic and oxidative environment by acidification and phagocytosis of lysosomes, to degrade contents; the restriction of microbial access to essential nutrients, such as Zn^2+^, amino acids, or fatty acids [6]; and the production of antimicrobial peptides and cytokines; and, most importantly, the induction of autophagy to eliminate mycobacteria from macrophages [7]. Autophagy is an intracellular lysosomal degradation pathway that maintains intracellular environmental homeostasis by removing cellular debris, intracellular pathogens, and dysfunctional organelles [8,9]. It also involves the clearance of intracellular mycobacteria via multiple signaling pathways, including the AMPK and mTOR pathways [10,11].

In resting cells, inflammatory vesicle activation, antimicrobial autophagy, and the induction of cell death are all major effector mechanisms that act against intracellular pathogens [12]. Furthermore, additional resistance against pathogens is produced by a large number of interferon-induced genes that enhance cytoplasmic immunity [13]. Among the many effector proteins with antimicrobial functions induced by interferon, the guanylate-binding protein 1 (GBP1/GBP2b) is a 65 kDa protein that exerts considerable antimicrobial functions [14,15]. GBP1/GBP2b is considered to disrupt the structural integrity of bacteria and release ligands for the stimulation of inflammatory vesicles [16–18]; and activate antimicrobial effector mechanisms, such as the release of phagocytic oxidases, antimicrobial peptides, and autophagic effectors, to mediate intracellular bacterial killing [19,20]. However, the role of GBP2b in *M. bovis* infection remains unclear.

Gene expression in eukaryotic cells is a process that is guided by a variety of complex mechanisms [6]. Transcriptional regulation, which is the first layer of gene expression control, defines rapid signal-dependent expression changes. In this investigation, a ribonucleic acid (RNA) sequence-based transcriptomic analysis was used to obtain a global profile of genes that were differentially expressed at 6 and 24 h after *M. bovis* infection with BMDMs. A comprehensive analysis of transcriptome data was performed using a bioinformatics approach, to identify genes and pathways that are differentially expressed after *M. bovis* infection of macrophages. A subsequent gene co-expression analysis identified the differentially expressed GBP2b gene as a potential key molecule that participates in the process of autophagy and *M. bovis* infection. Furthermore, in vitro experiments using BMDMs and RAW264.7 cells to study GBP2b loss of function and autophagy-related pathways experimentally showed that GBP2b is involved in regulating *M. bovis*-induced autophagy in macrophages by mediating the AMPK/mTOR/ULK1 signaling pathway.

## Materials and methods

### Bacterial culture

Virulent *M. bovis* Beijing strain was acquired from the China Institute of Veterinary Drug Control and cultured in 7H9 Middlebrook media (#BD271310, BD Biosciences, USA) containing 0.05% Tween-80 (#9005-65-6, Sigma–Aldrich, USA) and 10% albumin-dextrose-catalase (#211886, BD Biosciences, USA) enrichment solution. Growth was determined at 37°C for a one-week to medium logarithmic period.

### Macrophage infection and RNA isolation

As previously described [21,22], BMDMs were isolated and cultured in Dulbecco’s modified Eagle’s medium (DMEM, #11965118, Thermo Fisher Scientific, USA) containing 10 ng/mL of recombinant murine M-CSF (#AF-315-02-100, Pepro Tech, USA) and 10% fetal bovine serum (#10270-106, ThermoFisher Scientific, USA) for 7 days. BMDMs were suspended in fresh DMEM without antibiotics and plated in six-well plates at 2 × 10^6^ cells per well overnight, following infection with *M. bovis* (MOI 10). The uninfected macrophages and infection macrophages as control were incubated for a further 6 h or 24 h at 37°C with 5% CO_2_. Cells were subsequently washed with warm PBS three times, and total RNA was obtained in different samples using TRIzol reagent (#15596–018, Invitrogen, USA) according to the manufacturer’s protocol. Cells were homogenized for 2 minutes and rested horizontally for 5 minutes. The mix was centrifuged for 5 minutes at 12,000×g at 4°C then the supernatant was transferred into a new EP tube with 0.3 mL chloroform/isoamyl alcohol (24:1,#X205-950, Amresco, USA). Then centrifuged at 12,000×g for 10 minutes at 4°C After centrifugation, the upper aqueous phase where RNA remained was transferred into a new tube with an equal volume of supernatant of isopropyl alcohol, then centrifuged at 13,600 rpm for 20 minutes at 4°C After removing the supernatant, the RNA pellet was washed twice with 1 mL 75% ethanol, then the mix was centrifuged at 13,600 rpm for 3 minutes at 4°C to collect residual ethanol, followed by an air dry for 5–10 minutes in the biosafety cabinet. Finally, 25 µL-100 µL of DEPC-treated water was added to dissolve the RNA. RNA samples were quantified and the quality was assayed by a Nanodrop Lite spectrophotometer (Thermo Fisher Scientific, Waltham, USA). Finally, RNA samples with a spectral A260/A280 nm ratio between 1.8 and 2.0 and an A260/A230 nm ratio >1.5 were selected for further analysis.

### mRNA library construction

The mRNA was purified using oligonucleotide (dT)-modified magnetic beads. Purified mRNA was cleaved into small fragments using fragmentation buffer at a suitable temperature. First-strand cDNA was then obtained by random hexamer-initiated reverse transcription, followed by second-strand cDNA synthesis. Then, A-Tail Mix and RNA Index junction were added by incubation to end the repair. The cDNA fragment obtained in the previous step is PCR amplified and the product is purified by Amputee XP microbeads and dissolved in EB solution. The product was validated for quality control on an Agilent Technologies 2100 Bioanalyzer. The double-stranded PCR product obtained in the previous step was denatured by heating and cyclized with a splint oligonucleotide sequence to obtain the final library. Single-stranded circular DNA (SsCir DNA) was formatted as the final library. The final library was amplified with phi29 to produce a DNA nanoball (DNB) with more than 300 molecular copies, and the DNB was loaded into a patterned nanoarray to generate 100 base pair reads on a BGIseq500 platform (UW Genetics, Shenzhen, China).

### Quantitative Real-Time Polymerase Chain Reaction (qRT-PCR)

The reaction cDNA was reverse transcribed using Prime-Script^TM^ RT Master Mix (#RR036B, TAKARA, Japan) and FastStart Universal SYBR Green (#4913850001, Takara, Japan) was used for the qRT-PCR on a Lightcycler480 RT-PCR instrument (Roche Diagnostics, Penzberg, Germany). GAPDH was used as a standardized internal control. The quantification was performed with the 2^−^^△^^△^^CT of each sample^/2^−^^△^^△^^CT of the control^ method and primers in this study were shown in Table 1.

**Table 1.** Primers for qRT-PCR in this study

Names	Sense (5’−3’)	Antisense (5’−3’)
GBP2b	GAGTACTCTCTGGAA	TAGATGAAGGTGCTG
AMPK	AAAGGGTACACAGACGCCAG	CTCCGAATCTTCTGCCGGTT
ULK1	CCACTTGGGGAGAAGGTGTG	ACTCAACAGCAGACAGCCAG
mTOR	CCGCTACTGTGTCTTGGCAT	CAGCTCGCGGATCTCAAAGA
Egr3	GCCTGACAATCTGTACCCCG	TCCATCACATTCTCTGTAGCCA
GBP5	GAACGCCAAAGAAACAGTGAG	GAATAGCCTCCAACCTCTGTG
Itgb8	ACTGGGCCAAAGTGAACACA	TCTTGAACACACCATCCGCA
Ptgs2	CATCCCCTTCCTGCGAAGTT	GGCCCTGGTGTAGTAGGAGA
Socs1	AGCAGAGAGAACTGCGGC	GCTGGCGGCAGGACG
Ptgs2	CATCCCCTTCCTGCGAAGTT	GGCCCTGGTGTAGTAGGAGA
Cxcl 5	CACTCGCAGTGGAAAGAACG	CGTGGGTGGAGAGAATCAGC
Cxcl 3	GAAAGGAGGAAGCCCCTCAC	ACACATCCAGACACCGTTGG
Cxcl 2	GCTGTCCCTCAACGGAAGAA	CAGGTACGATCCAGGCTTCC
IL-1β	TGCCACCTTTTGACAGTGATG	TGATGTGCTGCTGCGAGATT

### Analysis of differentially expressed mRNAs

As previously described [23–25], the R package DESeq2 algorithm determines differentially expressed genes and mRNAs for identifying transcripts from RNA-Seq data. The false discovery rate (FDR) determines the q-value significance threshold for multiple tests. FDR < 0.05 and folding change >2 indicate the threshold of differentially expressed genes.

### Pathway analysis

As previously described [23–25], pathway analysis was used to determine the significant pathway of the differential genes following KEGG. Fisher’s exact test and χ2 test were used to select the significant pathway, and the threshold of significance was defined by *p*-value and FDR. Enrichment Re was calculated as above.

### GBP2b-mRNA co-expression correlation analysis

The degree of correlation between samples was analyzed by calculating the Pearson correlation coefficient between GBP2b and the differential mRNA in the tested samples. The greater the correlation is, the greater the likelihood of regulation between GBP2b and mRNA is considered. In this analysis, a Pearson correlation coefficient of |R| > 0.9 was taken.

### GBP2b-mRNA-pathway

A GBP2b-comRNA-pathway network was constructed based on the Pearson correlation coefficient threshold |R| > 0.9 to preserve the co-expression relationship of GBP2b-mRNA and the regulatory relationship among the significant pathways involved in GBP2b-coexpressed mRNA to show the pathways that GBP2b may mainly regulate.

### Animal and *M.* *bovis* infection

C57BL/6 mice (SPF, 6–8 weeks old) were purchased from Yangzhou University (Yangzhou, China) and raised in a level III biosafety facility (Nanjing Institute for Food and Drug Control, Nanjing, China). The mice were divided into two groups of 10 mice each. In one group, 200 colony-forming units (CFU) of *M. bovis* per mouse were given intranasally. The other group was inoculated with the same dose of sterile PBS. After 4 weeks of *M. bovis* infection, the spleen and lungs of the two groups of mice were collected to detect the expression of GBP2b.

### Knockdown of GBP2b and AMPK expression

Initially, three different sequences specifically targeting mouse GBP2b and AMPK were designed by Gene Chem Co., Ltd (http://www.genechem.com.cn). The sequences of the small interfering RNAs (siRNAs) are shown in Table 2. Briefly, BMDMs and RAW264.7 cells were inoculated at a density of 1 × 10^6^/well in 6-well plates for 12 h. Then, according to the manufacturer’s protocol, control siRNA (siCon), siRNA-GBP2b (siGBP2b-1, siGBP2b-2, and siGBP2b-3) or siRNA-AMPK (siAMPK-1, siAMPK-2, and siAMPK-3) were transfected into BMDMs or RAW264.7 cells using Lipofectamine 3000 transfection reagent (#L3000015, Thermo Fisher Scientific, USA) for 48 h, respectively. Finally, the effect of GBP2b or AMPK knockdown efficiency was examined by Western blot and qRT-PCR, and the best siRNA was selected. The most effective siRNAs were siGBP2b-3 and siAMPK-2.

**Table 2.** The specific siRNA sequence in this study

Names	Sense (5’−3’)	Antisense (5’−3’)
siGBP2b-1	GCAGCACCUUCAUCUACAATT	UUGUAGAUGAAGGUGCUGCTT
siGBP2b −2	GACCAGCUGAAUAAAGAAUTT	AUUCUUUAUUCAGCUGGUCTT
siGBP2b −3	GAGCAACAAAGAAUCAUAUTT	AUAUGAUUCUUUGUUGCUCTT
siAMPK-1	GGGAACACGAGUGGUUUAATT	UUAAACCACUCGUGUUCCCTT
siAMPK-2	GCCGACCCAAUGAUAUCAUTT	AUGAUAUCAUUGGGUCGGCTT
siAMPK-3	GGUCCACAGAGAUUUGAAATT	UUUCAAAUCUCUGUGGACCTT
siCon	UUCUCCGAACGUGUCACGUTT	ACGUGACACGUUCGGAGAATT

### Cell viability and CFU assay

As previously described [26], cell viability was assessed by MTS tetrazolium analysis. In brief, RAW264.7 cells and BMDMs were inoculated in 96-well cell plates at a density of 5 × 10^4^/well and transfected with siCon or siGBP2b vector for 48 h. Following adding a 20 µL MTS reagent to each well, the cells were cultured at 37°C for 3 h; and then measured absorbance at 490 nm by using a multimode microplate reader (TECAN, manidoff, Switzerland). To detect intracellular bacterial CFU, two types of cells were inoculated in 24-well plates at a density of 1 × 10^5^/well, then transfected with siCon, siGBP2b, and siAMPK vectors for 48 h. Finally, the cells were infected with *M. bovis* (MOI 10) at different time points (0, 6, 12, 24, 48, and 72 h), respectively. Cells were subsequently lysed with sterile water and cell lysates were inoculated in 7H11 agar (#BD283810, BD Biosciences, USA) after a series of dilutions and cultured at 37°C for 3–4 weeks. Bacterial CFU was counted to evaluate the viability of intracellular bacteria after down-regulation of GBP2b.

#### Immunofluorescence assay

After infection of BMDMs and RAW264.7 cells transfected with siGBP2b and siCon vectors at different time points (0, 6, 12, and 24 h) by *M. bovis*, the cells were fixed with 4% paraformaldehyde for 20 min and washed three times with PBS at room temperature. Subsequently, blocking cells with 3% BSA for 1 h and incubating with rabbit anti-LC3A/B (1:200, #12741, Cell signaling technology, USA) at 4°C for 12 h. After washing three times with cold PBS, donkey anti-rabbit IgG Alexa Fluor 647 (1:500, #4414, Cell Signaling Technology, USA) was added and incubated for 1 h. Finally, the nucleus was stained with DAPI (1:1000, #28718-90-3, Sigma–Aldrich, USA), and the cells were visualized immediately with an OLYMPUS microscope (Suzhou Jing Kai Instrument and Equipment Co., Ltd., Suzhou, China). Approximately 100 cells are used to calculate the fluorescence intensity. The image J software was used for the analysis.

### Immunohistochemistry (IHC)

Following sacrifice, the spleens of mice were collected and fixed in 10% formalin solution (#50-00-0, Sigma–Aldrich, USA) at 4°C overnight. Serial sections (8 mm thick) in a cryostat and blocked at room temperature with endogenous peroxidase blocker for 15 min. Rabbit antibodies against GBP1 (1:200, #PA5– 23509, Thermo Fisher Scientific, USA) were incubated with tissue sections at 4°C for 12 h and washed three times with cold PBS. The HRP-conjugated anti-rabbit immunoglobulin G (1:1000, #12–348, Merck KGaA, Germany) were incubated for 1 h. Lastly, observation was made by confocal microscopy at a magnification of × 200.

### Nuclear and cytoplasmic protein extraction

NE-PER™ nucleus and cytoplasm extraction reagent (#78835, Thermo Fisher Scientific, USA) was used to extract proteins from the nucleus and cytoplasm of cells according to the manufacturer’s instructions.

#### Western blot

Cells were lysed with RIPA buffer (#P0013C, Beyotime, China). Protein concentration was quantified using a BCA protein assay kit (#P0012S, Beyotime, China) and transferred to PVDF membrane (# 88518, Thermo Fisher Scientific, USA) after separation on SDS-PAGE gels. The membranes were incubated with primary antibodies at 4°C for 12 h, and then washed three times with TBST buffer. Finally, the membranes were incubated with HRP-conjugated secondary antibodies for observation. An image processing system (Bio-Rad) and ImageJ software were used to determine and quantify these protein bands, respectively. Primary antibodies and a secondary antibody, including anti-GBP1 (#PA5– 23509), were purchased from Thermo Fisher Scientific. The other antibodies, including anti-GAPDH (#5174), anti-LC3A/B (#12741), anti-Beclin-1 (#3738), anti-AMPKa (#5831), anti-phospho-AMPKα (#50081), anti-mTOR (#2983), anti-phospho-mTOR (#2971), anti-ULK1(#8054), anti-phospho-ULK1 (#6887) and anti-rabbit IgG-HRP-linked antibody (#7074) were purchased from Cell Signaling Technology. The ratio of target protein/GAPDH or LC3II/LC3I is shown below the protein band.

### Statistical analysis

The gene expression was calculated by RSEM (v1.2.12) https://github.com/deweylab/RSEM). The heatmap was drawn by heatmap (v1.0.8) (https://cran.r-project.org/web/packages/pheatmap/index.html) according to the gene expression in different samples. Essentially, differential expression analysis was performed using the DESeq2(v1.4.5) (http://www.bioconductor.org/packages/release/bioc/html/DESeq2.html) with a Q value ≤0.05. KEGG (https://www.kegg.jp/) enrichment analysis of annotated different expression genes was performed by Pyper (https://en.wikipedia.org/wiki/Hypergeometric _distribution) based on the Hypergeometric test. GraphPad Prism 8.0.1 was applied to analyze all the experimental data. Data are presented as means ± SEM. *P* < 0.05 was considered significant.

#### Animal ethics statement

All procedures using SPF C57BL/6 mice were approved by the International Society for the Evaluation and Accreditation of Laboratory Animal Management. The Animal Experimentation Ethics Committee of Nanjing Agricultural University, Nanjing, China (PTA2019024) approved all the animal experiments in the research.

## Results

### Differentially expressed mRNAs in BMDMs infected with M. bovis

**Figure 1.**
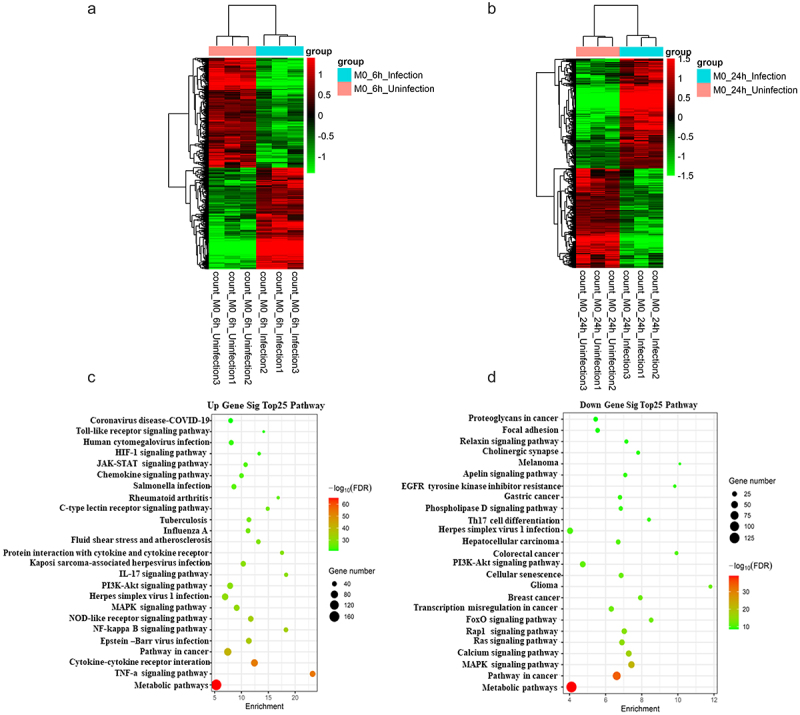
Differential expression of mRnas in BMDM cells infected with *M. bovis*.

RNA-Seq was performed in infected and uninfected BMDM samples that were collected at 6 or 24 h after infection of the cells with *M. bovis*, with uninfected macrophages serving as controls, to determine the regulatory network of mRNAs in vitro after *M. bovis* infection of BMDMs. Systematic clustering was used to screen for mRNAs that were differentially expressed by >2-fold (FDR <0.05); 2194 differential mRNAs were identified in the 6-h samples (Figure 1, Supplementary Table S1), and 1913 differential mRNAs were identified in the 24-h samples (Figure 1, Supplementary Table S2).

(a and b) Hierarchical clustering shows a distinguished mRNA expression profiling between 6-h (A) and 24-h (b) infected and uninfected groups. (c) Red and green shades indicate genes that are up and down-regulated in expression in all samples, respectively. (d) The top 25 pathways are associated with up-or down-regulation of genes.

The DESeq2 package was used to calculate the differences in the detected gene count values, andto identify upregulated and downregulated genes. Pathway analysis revealed the pathways that corresponded to the top 25 upregulated transcripts and those that corresponded to the downregulated transcripts (Figure 1; Supplementary Table S3). The most abundant network among the up-regulated and down-regulated genes was the “metabolic pathway.” The TNF signaling pathway is involved in apoptosis in *M. tuberculosis*-infected host cells [27]. The NF-κB signaling pathway is central to mediating the release of inflammatory cytokines. The NOD-like receptor signaling pathway is a major pathogen-pattern-recognition receptor that likewise mediates inflammatory vesicle activation and cell pyroptosis. Pathways such as the MAPK signaling pathway, the PI3K–Akt signaling pathway, and calcium signaling, were reported to be related to apoptotic and autophagic processes after *M. tuberculosis* infection of the host [28–30]. The differentially expressed mRNAs identified here, either up-regulated or down-regulated, may be involved in *M. bovis* infection or host resistance to infection.

**Figure 2.**
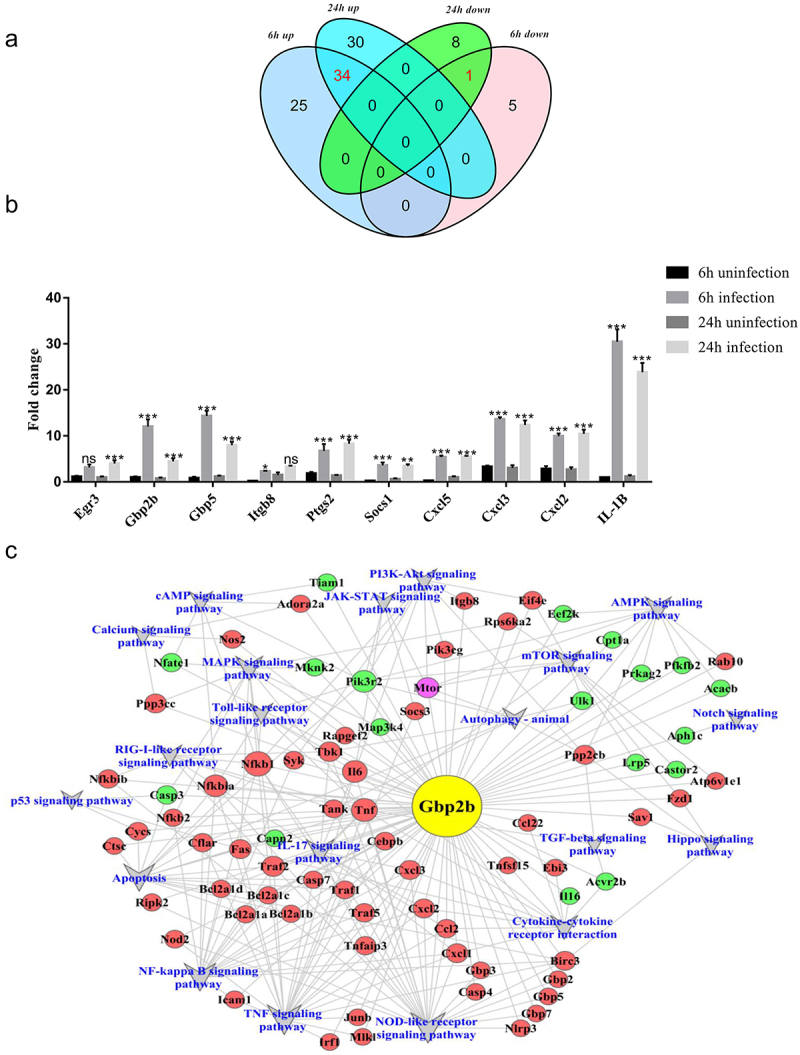
GBP2b was identified for further study.

A Venn diagram was drawn to capture the overlap between the differentially expressed groups of genes (Figure 2(a); Supplementary Table S4). The Venn diagram showed that 34 genes were upregulated and one gene was downregulated in both the 6-h and 24-h differential results (log2F > 4.5). An additional 10 mRNAs from 35 differentially expressed genes that have been reported to be associated with pathogenic infections were selected for qRT-PCR, to verify the confidence of the RNA-Seq data. As shown in Figure 2(b), the expression levels of the 10 mRNAs were in good agreement with the RNA-Seq data, with three genes, GBP2b, GBP5, and IL-1β, differing significantly between the infected and uninfected groups or between the 6 h and 24 h of infection groups. In particular, GBP2b exhibited the most significant differential expression. Therefore, we targeted the differential GBP2b gene for further study. In addition, previous studies reported that GBP2b acts as a host defense protein against infection by a variety of pathogens, including *Shigella flexneri* and *Listeria* species [19,31]. However, whether GBP2b also exerts antimicrobial effects during infection with virulent *M. bovis* and the underlying pathways remain unclear. Therefore, it is important to further investigate the role of GBP2b in infections with *M. bovis*.

(a) Venn diagram shows the overlap between 6 h and 24 h up-and down-regulated genes (log 2Fc > 4.5). (b) The mRNA levels of ten genes from these 35 genes were verified using qRT-PCR. (c) GBP2b-mRNAco-expression correlation network. V shape represents the signal pathway. Red and green circles represent up and down-regulated genes, respectively. Purplecircles represent down regulated genes in the 6-h sample and up regulated genes in the 24-h sample. Lines represent interactions between pathways, *n *= 3; *represent *P*< 0.05 and **represent *P*< 0.01 and ***represent *P*< 0.001; ns represents no significance.

### GBP2b-comRNA-pathway regulatory network analysis

In this study, the samples were screened for differential mRNAs associated with GBP2b based on a Pearson’s correlation coefficient threshold of |R| > 0.9, and a GBP2b-comRNA-pathway network was constructed to identify the possible major pathways that are regulated by GBP2b (Figure 2(c), Supplementary Table S5). The main pathways involved in the regulation of GBP2b included pattern recognition receptors that recognize pathogens and activate natural immune responses, such as Toll-like receptors, NOD-like receptors, and RIG-I-like receptor signaling pathways; as well as their downstream pathways, including the MAPK, TNF, and NF-κB signaling pathways. The networks also included several pathways involved in autophagy, such as the autophagy-animal, AMPK signaling pathway and mTOR signaling pathway. These networks provided extensive information on the regulatory pathway of the GBP2b host antimicrobial protein during *M. bovis* infection. Among the many signaling pathways mentioned above, this study focused on how GBP2b is involved in autophagy after infection of macrophages with *M. bovis*, and its association with the AMPK and mTOR signaling pathways.

#### *M.* *bovis* infection significantly increases GBP1/GBP2b expression in vivo and in vitro

**Figure 3.**
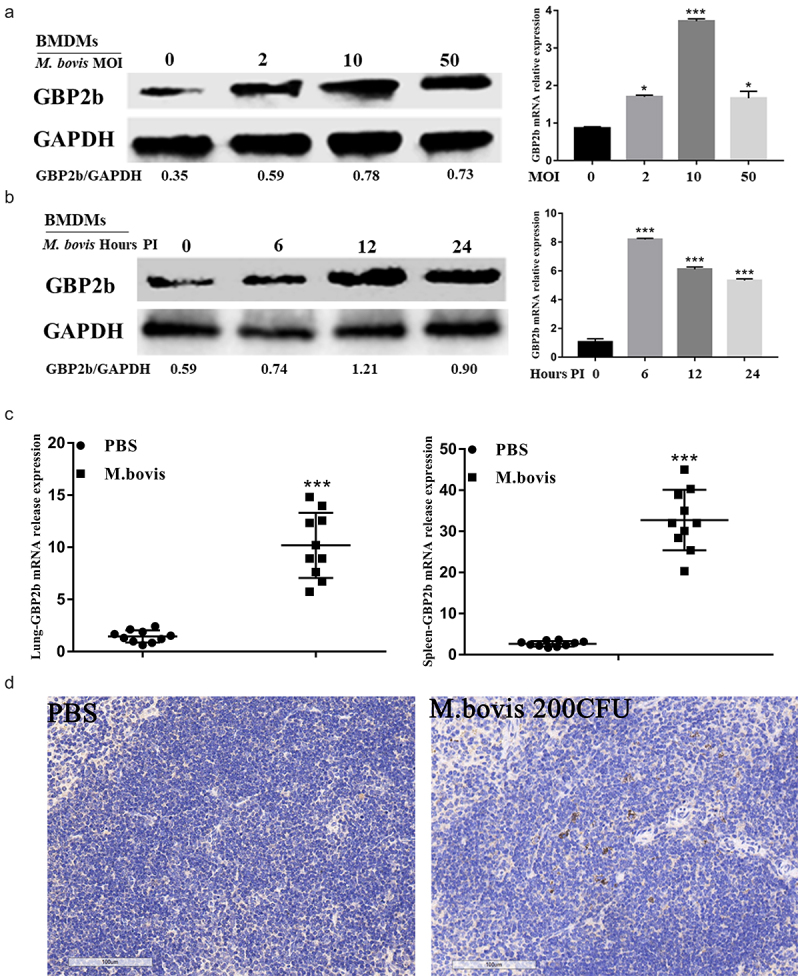
*M. bovis* induced upregulation of GBP2b *in vitro* and *in vivo*. (a and b) Detection of GBP2b protein and mRNA levels by Western blot and qRT-PCR in BMDM cells infected with the indicated MOI (0, 2, 10, and 50) *M. bovis* for 24 h (a) or infected with *M. bovis* for MOI 10 at different time points (0, 6, 12 and 24 h) (b). (c) After 4 weeks, the spleen and lung tissues of mice nasally inoculated with PBS or *M.* *bovis* were collected for qRT-PCR. (d) Immunohistochemistry analysis for GBP2b in spleen tissue from a mouse inoculated with PBS or *M. bovis*. Scale bar = 100 *µ*m. GAPDH acts as load control. MOI represents multiplicity of infection; Hour PI represents hours post-infection; *n* = 3; * represents *P* < 0.05 and *** represents *P* < 0.001.

The expression of GBP2b during infection with *M. bovis* was first examined in vitro and in vivo simultaneously to further determine the expression of GBP2b in transcriptome data analysis and the role it plays in the infection of macrophages with *M. bovis*. In vitro, BMDMs were infected with *M. bovis* at different MOIs (0, 2, 10, and 50) for 24 h, or with *M. bovis* at an MOI of 10 at different time points (0, 6, 12, and 24 h), respectively. The results of qRT-PCR indicated that the GBP2b mRNA was increased at different MOIs and infection times, with the highest levels detected at an MOI of 10 and an infection time of 6 h (Figure 3). Consistent with the mRNA levels, the GBP2b protein was upregulated at different MOIs and infection times, with the highest expression detected at an MOI of 10. Nevertheless, its expression was highest at 12 h post-infection and remained high at 24 h compared with that observed at 0 and 6 h (Figure 3). The difference in the expression of GBP2b at the protein and mRNA levels may be attributed to asynchronous expression. In in vivo experiments, the level of the GBP2b mRNA in the lung and spleen of mice inoculated with *M. bovis* or PBS for 4 weeks was measured; we found that the GBP2b mRNA was significantly upregulated in the infection group compared with the PBS group (Figure 3). Similarly, the results of the immunohistochemical analysis showed that the expression of the GBP2b protein was increased in the *M. bovis-*infected spleen of mice (Figure 3; Supplementary Figure S1).

The results reported above showed that, consistent with the results of the transcriptome data analysis, *M. bovis* infection-induced remarkable expression of GBP2b. The relationship between *M. bovis* infection and GBP2b upregulation inspired us to further explore the function of GBP2b in the host immune response to *M. bovis* infection.

### Autophagy in mouse macrophages induced by *M.* *bovis* infection

**Figure 4.**
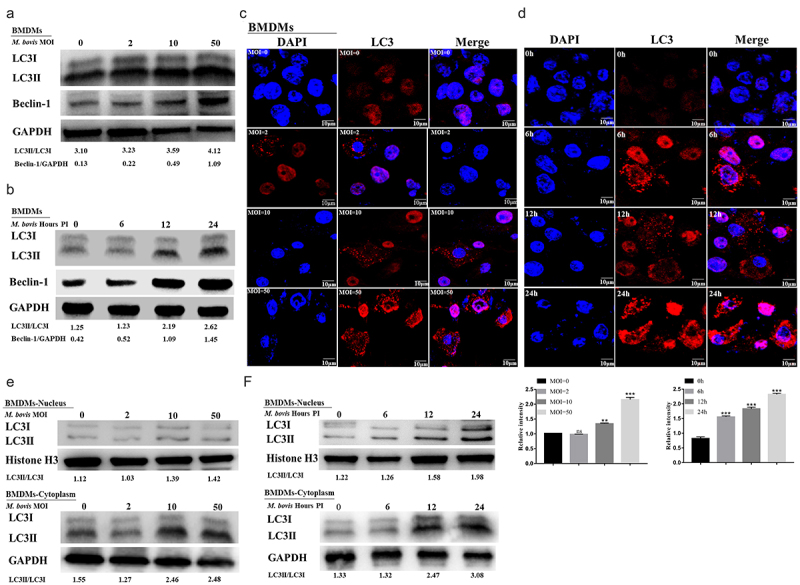
Autophagy is induced in *M. bovis*-infected BMDM cells. (a and b) Detection of protein level of LC3I/LC3II and Beclin-1 in BMDM cells infected with the indicated MOI (0, 2, 10, and 50) *M. bovis* for 24 h (a) or infected with *M. bovis* for MOI 10 at different time points (0, 6, 12 and 24 h) by Western blot (b). (c and d) BMDM cells infected with the indicated MOI (0, 2, 10, and 50) *M. bovis* for 24 h (c) or infected with *M. bovis* for MOI 10 at different time points (0, 6, 12, and 24 h) (d) were immune-stained by anti-LC3II antibody (red). DAPI (blue) for staining cell nuclei. Scale bar = 10 *µ*m. (e and f) Detection of protein level of LC3I/LC3II in the nucleus and cytoplasm of BMDM cells infected with the indicated MOI (0, 2, 10, and 50) *M. bovis* for 24 h (e) or infected with *M. bovis* for MOI 10 at different time points (0, 6, 12 and 24 h) by Western blot (f). GAPDH acts as load control. MOI represents a multiplicity of infection; Hour PI represents hours post-infection; *n* = 3; ** represents *P* <0.01 and *** represents *P* <0.001; ns represents no significance.

In addition to being necessary for cell survival and the maintenance of the internal environmental homeostasis, especially in the face of various stresses, autophagy is also an important mechanism for the autonomous defense of macrophages against intracellular mycobacteria [8]. Therefore, the present study first investigated whether *M. bovi*s infection induced autophagy in mouse macrophages. Under cellular homeostatic conditions, the autophagy marker protein LC3-I is localized in the cytoplasm. In the presence of various stresses (such as infection), autophagy is induced, as evidenced by the conversion of LC3-I to LC3-II and its binding to the autophagosome membrane [30,31] Thus, LC3-II is linked to the number of autophagosomes [32]. Another autophagy marker protein, Beclin-1, contributes to the formation of autophagosomes. In this study, the expression levels of LC3I/II and Beclin-1 were elevated in macrophages infected with *M. bovi*s at different MOIs or at different times, which revealed that their expression was time- and dose-dependently upregulated (Figure 4); similar results were obtained when LC3 puncta were counted (Figure 4). In addition, a Western blot assay of LC3I/II protein levels in the nucleus and cytoplasm revealed higher levels of LC3II protein expression in the cytoplasm compared with the nucleus after infection with *M. bovis* at different MOIs or at different time points (Figure 4).

### Knockdown of GBP2b impairs the induction of autophagy in *M. bovis*-infected mouse macrophages

**Figure 5.**
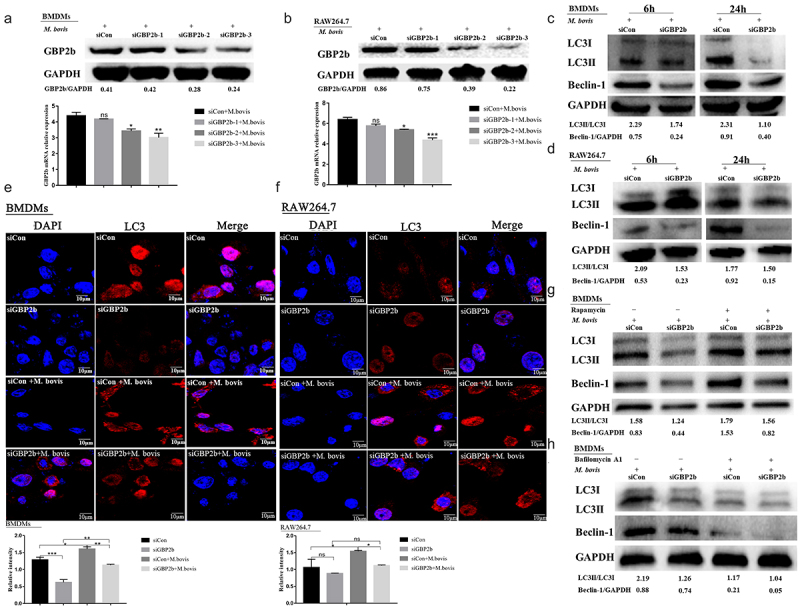
Downregulation of GBP2b reduces the induction of autophagy in mouse macrophages with *M. bovis* infection. **Note**: Both BMDM cells and RAW264.7 cells were transfected with GBP2b-specific siRNA (siGBP2b) and control siRNA (siCon), respectively, and then infected 6 and 24 h with *M. bovis* (MOI 10). (a and b) the protein and mRNA levels of GBP2b was determined in BMDM cells (a) or RAW26.7 cells (b) infected 24 h with *M. bovis* by Western blot and qRT-PCR. (c and d) the LC3I/LC3II and Beclin-1 protein levels were determined in BMDM cells (c) or RAW264.7 cells (d) by Western blot after 6 and 24 h infected with *M. bovis*, respectively. (e and f) BMDM cells (e) and RAW264.7 cells (f) infected 24 h with *M. bovis* were immune-stained by anti-LC3II antibody (red). DAPI (blue) for staining cell nuclei. Scale bar = 10 *µ*m. (g and h) the LC3I/LC3II and Beclin-1 protein levels in BMDM cells were detected by Western blot after being exposed to 1.5 nM rapamycin (g) or 100 nM bafilomycin A1 (h) for 12 h and then infected with *M. bovis* for 24 h. GAPDH acts as load control. *n* = 3; * represents *P* < 0.05 and ** represents *P*  < 0.01 and *** represents *P*  < 0.001; ns represents no significance.

The GBP2*b*-comRNA-pathway regulatory network analysis described above demonstrated that GBP2b is associated with the autophagic signaling pathway. GBP2b-specific siRNA was applied to down-regulate GBP2b expression in BMDMs and RAW264.7 cell infection models, to investigate whether GBP2b is involved in the autophagy regulation in mouse macrophages induced by *M. bovis*. The knockdown efficiency was detected by Western blot and qRT-PCR after siRNA transfection for 48 h. It was found that siGBP2b downregulated the expression of GBP2b in RAW264.7 cells and BMDMs (Figure 5). Among them, siGBP2b-3 inhibited the effect best; thus, this siRNA was applied in subsequent experiments. Subsequent infection of both cell types with *M. bovis* for 6 and 24 h revealed that the down-regulation of GBP2b downregulated the expression levels of LC3-II and Beclin-1 in both types of cells (Figure 5). In addition, the number of LC3 puncta was decreased in both GBP2b-knockdown cell types after *M. bovis* infection, as assessed using immunofluorescence microscopy (Figure 5). These results suggest that the down-regulation of GBP2b impairs autophagy during *M. bovis* infection, thus indicating that GBP2b plays a critical role in autophagy induction.

In the next experiment, the signaling pathway regulated by GBP2b-dependent autophagy was investigated. First, we assessed whether transfection with a GBP2b-specific siRNA and a control siRNA affected macrophage viability using an MTS analysis. The viability of cells transfected with the two siRNAs was not significantly different (Supplementary Figure S2A and B). To further validate the role of GBP2b in autophagy regulation, the levels of Beclin-1 and LC3-II were examined in the presence or absence of rapamycin (1.5 nM) and bafilomycin A1 (100 nM) treatment, as described previously [33]. Rapamycin increases autophagy by inhibiting its target, mTOR, whereas bafilomycin A1 prevents the maturation of autophagic vacuoles and the degradation of LC3II by inhibiting the fusion of lysosomes with autophagosomes [34]. Our experiments showed that the levels of the Beclin-1 and LC3-II proteins were remarkably reduced in both GBP2b-knockdown cell types. However, rapamycin reversed this effect (Figure 5). In contrast, bafilomycin A1 downregulated the LC3-II and Beclin-1 proteins (Figure 5). Taken together, these data indicated that GBP2b plays an important role in macrophage autophagy induced by *M. bovis*.

### GBP2b-Mediated autophagy involves the AMPK/mTOR/ULK1 signaling pathway during *M. bovis* infection

**Figure 6.**
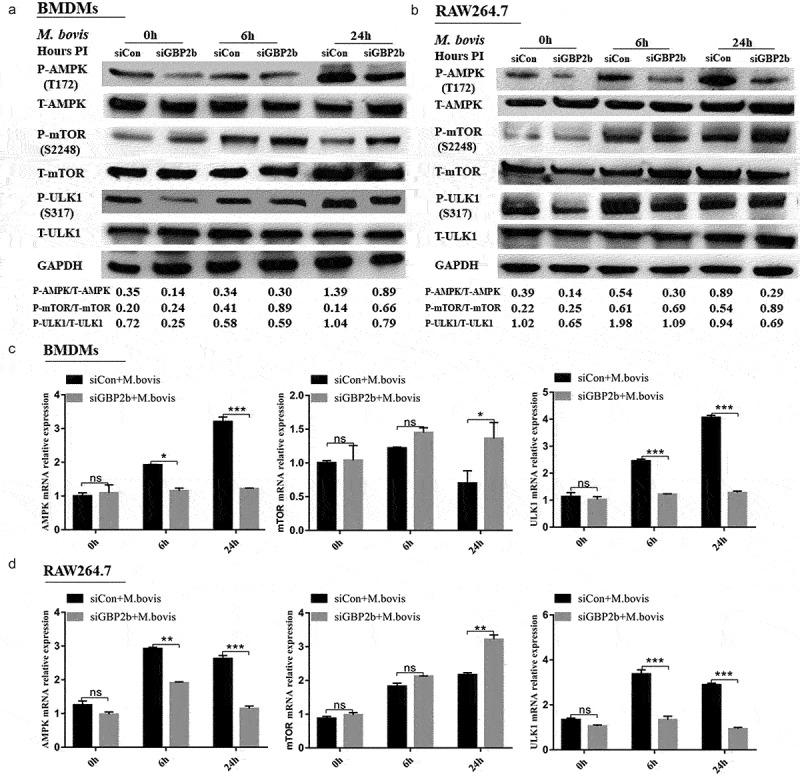
Downregulation of GBP2b expression reduces autophagy by downregulating AMPK/mTOR/ULK1 signaling during *M. bovis* infection. (a and b) the protein levels of *p*-AMPK, AMPK, *p*-mTOR, mTOR, *p*-ULK1, and ULK1 were determined by Western blot in BMDM cells (a) and RAW264.7 cells (b) transfected with siGBP2b and siCon and then infected 0, 6, and 24 h with *M. bovis* (MOI 10). (c and d) the mRNA levels of AMPK, mTOR, and ULK1 were determined by qRT-PCR in BMDM cells (c) and RAW264.7 cells (d) transfected with siGBP2b and siCon and then infected 0, 6, and 24 h with *M. bovis* (MOI 10). GAPDH acts as load control. *n* = 3; * represents *P* < 0.05 and ** represents *P* < 0.01 and *** represents *P* < 0.001; ns represents no significance.

**Figure 7.**
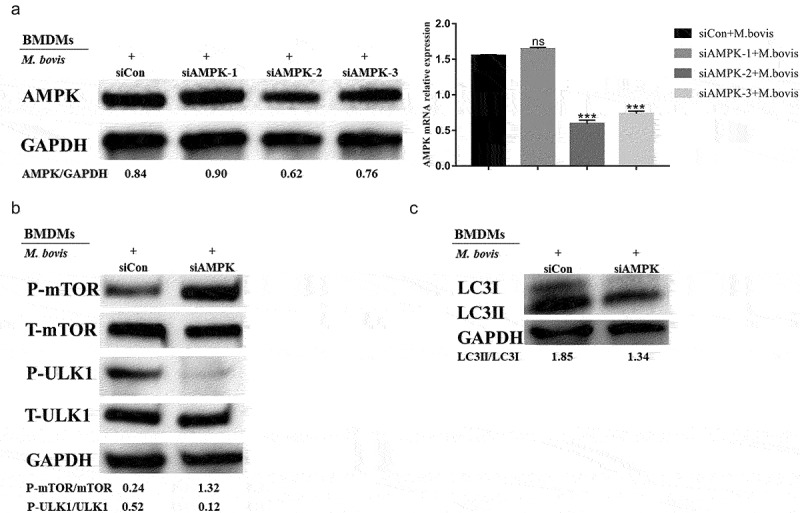
Autophagy is mediated by the AMPK signal during *M. bovis* infection. **Note**: BMDM cells transfected with siCon or AMPK-specific siRNA (siAMPK-1, siAMPK-2, siAMPK-3) and then were infected with *M. bovis* (MOI 10) for 24 h. (a) The protein and mRNA levels of AMPK in BMDM cells were determined by Western blot and qRT-PCR, respectively. (b) The p-ULK1, ULK1, p-mTOR, and mTOR protein levels in BMDM cells were determined by Western blot after being infected with *M. bovis* for 24 h. GAPDH acts as load control. *n*=*3*; *** represents *P* < 0.001; ns represents no significance.

AMPK is a protein kinase that plays a major role in the regulation of the reprogramming of metabolism and cell growth, and its enzymatic activity is entirely dependent on the phosphorylation of the thr172α subunit [33,35]. AMPK inhibits mTOR activity by phosphorylating tuberous sclerosis syndrome 2, thereby promoting autophagy [36]. In addition, ULK1 is a homolog of yeast Atg1 kinase, which plays an important role in autophagy induction [37–39]. The literature reported that, in the absence of nutrients, AMPK activates ULK1 via the phosphorylation of serines 317 and 777, thereby promoting autophagy. Under nutrient-sufficient conditions, the highly active mTOR phosphorylates ULK1 at serine 757 to block its activation, thus interfering with the interaction between AMPK and ULK1 [38,39]. In this study, the analysis of the GBP2b-comRNA-pathway regulatory network revealed that GBP2b was involved in the AMPK and mTOR signaling pathways, implying that GBP2b may participate in the cellular autophagy process through AMPK–mTOR signaling. Next, after 0, 6, and 24 h of infection with *M. bovis* in the two types of cells transfected with negative control and GBP2b-knockdown vectors, the AMPK, and ULK1 protein phosphorylation levels and mRNA levels were decreased after GBP2b knockdown, whereas the mTOR protein phosphorylation levels and mRNA levels were increased (Figure 6). ULK1 and mTOR activities were examined by siRNA inhibition of AMPK activity, to verify the relationship between AMPK and mTOR and ULK1 (Figure 7). The down-regulation of AMPK in BMDMs decreased the level of phosphorylation at Ser317 of ULK1 and upregulated mTOR phosphorylation (Figure 7). The down-regulation of AMPK expression also downregulated the LC3II and Beclin-1 proteins (Figure 7). The results presented suggest that GBP2b is involved in cellular autophagy via AMPK/mTOR/ULK1 signaling during *M. bovis* infection.

### Down-Regulation of GBP2b promotes the intracellular survival of *M.* *bovis*

**Figure 8.**
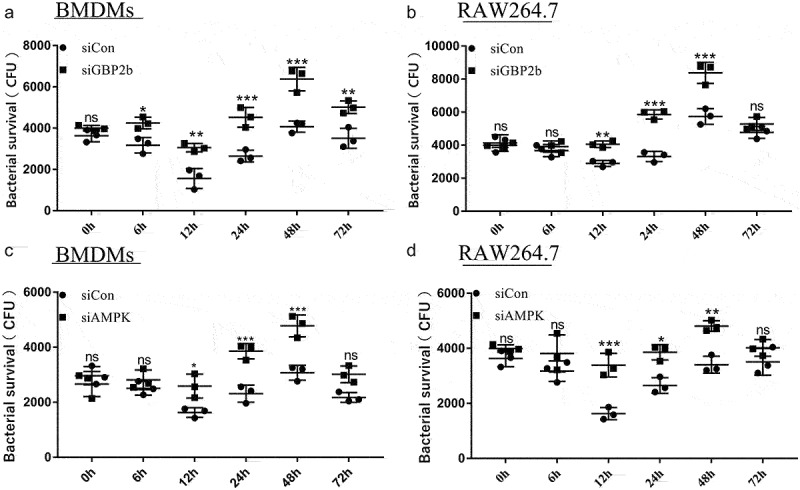
Downregulation of GBP2b and AMPK promotes *M. bovis* replication in macrophages. (a and b) Calculation of bacterial CFUs in BMDM cells (a) and RAW264.7 cells (b) transfected with siCon and siGBP2b after infected with *M. bovis* (MOI = 10) at different time points (0, 6, 12, 24, 48 and 72 h). (c and d) Calculation of bacterial CFUs in BMDM cells (c) and RAW264.7 cells (d) transfected with siCon and siAMPK after being infected with *M. bovis* (MOI = 10) at different time points (0, 6, 12, 24, 48 and 72 h). *n* = 3; * represents *P* < 0.05 and ** represents *P* < 0.01 and *** represents *P* < 0.001; ns represents no significance.

Numerous studies have elucidated the critical role of autophagy in the resistance to intracellular mycobacterial infection [29,36]. The experimental results reported above suggest that GBP2b promotes autophagy and that AMPK, which is a key protein in the regulation of the autophagic process, is also involved in the killing of intracellular *M. bovis* by macrophages [36]. We examined the survival of intracellular bacteria in BMDMs and RAW264.7 cells after transfection with siCon, siGBP2b, or siAMPK vectors for 48 h, respectively, and after different times of infection. The results showed that both GBP2b and AMPK knockdown attenuated the killing ability of macrophages, thereby increasing the CFU of intracellular *M. bovis* (Figure 8). These results suggest that GBP2b plays an antibacterial function by regulating autophagy during *M. bovis* infection.

## Discussion

At the cellular level, macrophages are the initial cells of the host immune system that actively fight against Mycobacterium infection, as well as the main effector cells for host phagocytosis and clearance of Mycobacterium; thus, their role in early infection is well established [4,5]. Several studies have highlighted significant differences in the production of key innate factors and cytokines at the transcriptional and protein levels in macrophages infected with *M. bovis* [40]. However, these studies assessed only a portion of the intrinsic macrophage response, and much remains unclear regarding the overall transcriptional differences in *M. bovis* or *M. tuberculosis* infections. Uncovering pathogen interference or manipulation of host cell processes is an important aspect of research in the field of infection biology, particularly for controlling disease and developing effective therapeutic strategies. In this regard, the transcriptional regulation of host cells, both as a pathogen autoimmune evasion strategy and as a response to pathogen infection, remains to be fully elucidated. In this study, mRNA information was obtained using the RNA-Seq technique at two different time points after infection of mouse BMDMs with *M. bovis*: early (6 h) and mid-term (24 h) infection. A total of 2986 mRNAs were screened for differential expression. One of the differential genes, GBP2b, was further investigated by constructing a GBP2b-comRNA pathway network to identify the possible major pathways regulated by GBP2b; a potential autophagy-related pathway, the AMPK/mTOR/ULK1 signaling cascade, was identified and selected for subsequent validation.

Autophagy, as a therapeutic strategy to maintain intracellular homeostasis and eliminate intracellular invading pathogens, has been widely studied [38]. New evidence indicates that GBP2b plays a significant role in autophagy regulation associated with *M. tuberculosis* infection [21,30]. Kim et al. reported that GBP1/GBP2b binds to p62/Sqstm1, which transmits ubiquitinated mycobacteria to autolysosomes, thus producing ubiquitin-derived peptides that eliminate mycobacteria [19]. HGBP1/2 promotes the fusion of non-fused *Chlamydia* inclusions with autophagosomes and is an important resistance factor for the IFNG-mediated elimination of *Chlamydia trachomatis* from macrophages [18,40]. In the current study, the regulation of macrophage autophagy induced by *M. bovis* was investigated by detecting the expression of Beclin-1 and LC3-II. Our data indicated that autophagy occurred in BMDMs infected with *M. bovis* at different MOIs or at different times. However, autophagy was less extensive when GBP2b was downregulated, as evidenced by the down-regulation of Beclin-1 and LC3-II, suggesting that GBP2b mediates autophagy occurrence during *M. bovis* infection.

Two nutrient-sensing pathways, namely, AMPK and the mammalian targets of rapamycin complex 1 (mTORC1), regulate the autophagic pathway. Some growth factors activate mTOR and block phagocytosis via the RTK–PI3K–AKT axis [41]. Nevertheless, various inhibitors of mTOR have been shown to induce autophagy and cause cellular stress, with such factors as starvation and intracellular infection inhibiting mTORC1 [42]. In the presence of infection or other stresses in macrophages, AMPK senses low cellular energy and is activated; in turn, activated AMPK inhibits mTORC1 phosphorylation on the one hand and activates ULK1 kinase by phosphorylating the Ser317 and Ser777 sites of ULK1, with the latter promoting autophagosome formation by activating ATG9, which was recently shown to lead to the induction of autophagy [39]. Herein, increased phosphorylation of the autophagy-associated proteins AMPK and ULK1 was observed in *M. bovis-*infected macrophages. However, down-regulation of GBP2b expression was followed by upregulation of mTOR phosphorylation and down-regulation of AMPK and ULK1 phosphorylation. These results suggest that GBP2b mediates the autophagic process through the AMPK/mTOR/ULK1 signaling cascade, thereby participating in the intracellular immune response process of macrophages.

Overall, our results validate GBP2b as a molecule that is critical for cell-mediated autophagy and the killing of intracellular *M. bovis*. Moreover, this study not only explored the important defense mechanisms against lethal pathogens but also provides an alternative consideration for drug target screening and treatment of Mycobacterium infections. However, the current study had several limitations. For example, GBP2b-knockdown mice were not used to further investigate the function of GBP2b in vivo and its biological mechanisms. Furthermore, the mechanism via which GBP2b regulates the autophagic process could have been investigated in greater depth in vitro.

## Supplementary Material

Supplemental Material

## Data Availability

The supplement tablets can be found at the following link: https://doi.org/10.6084/m9.figshare. 16696033v3 The original dataset is available in a publicly accessible repository. The original contributions presented in the study are publicly available. These data can be found in the following link: https://dataview.ncbi.nlm.nih. gov/object/PRJNA766808?reviewer = 1773orf0n9v4duecrgrtdrfta5; The BioProject ID is PRJNA766 808.
