# Supplementary material for: Guanylate-Binding protein 2b regulates the AMPK/mTOR/ULK1 signalling pathway to induce autophagy during Mycobacterium bovis infection
Source: Virulence. 2022 May 21;13(1):875–89. doi: 10.1080/21505594.2022.2073024 (PMC9132469; doi:10.1080/21505594.2022.2073024)
Supplement: Supplemental Material [file KVIR_A_2073024_SM9990.zip › Supplementary table 1 .pdf]

st_gene_id	gene_id	gene_symbol	log2FoldChange	foldchange	style	pvalue	padj
G10090_27315	12985	Csf3	11.02736378	2087.215408	up	9.01749E-40	1.43785E-37
G10090_4537	16175	Il1a	9.253197494	610.2250078	up	1.26425E-27	8.83958E-26
G10090_7545	12981	Csf2	8.781245522	439.9651746	up	1.98128E-10	2.54472E-09
G10090_13969	319909	Ism1	8.695723785	414.6423843	up	2.55805E-10	3.23155E-09
G10090_30411	16193	Il6	8.644650509	400.2202928	up	1.36292E-47	3.24002E-45
G10090_3976	16159	Il12a	8.617524339	392.7654833	up	5.42934E-15	1.28138E-13
G10090_22095	14825	Cxcl1	8.540039366	372.227121	up	4.27091E-89	4.65351E-86
G10090_30788	20311	Cxcl5	8.402037732	338.2714789	up	1.17889E-18	3.9523E-17
G10090_25486	13874	Ereg	8.14338039	282.7494464	up	1.16883E-20	4.83621E-19
G10090_21260	13614	Edn1	8.136984719	281.4987525	up	2.24577E-32	2.1913E-30
G10090_13592	16176	Il1b	7.937315434	245.1150799	up	6.50351E-18	2.05395E-16
G10090_18002	54448	Il1f6	7.854034081	231.3661636	up	6.52444E-10	7.84793E-09
G10090_10574	20310	Cxcl2	7.728635238	212.1050615	up	7.08503E-60	2.98828E-57
G10090_2060	330122	Cxcl3	7.617987278	196.445768	up	1.67522E-21	7.37489E-20
G10090_16411	19225	Ptgs2	7.480746634	178.6196059	up	1.21135E-83	9.31672E-81
G10090_14621	195727	Nhs	7.459765444	176.0407294	up	2.76543E-09	3.0182E-08
G10090_30906	11839	Areg	7.371589542	165.6035216	up	1.1926E-05	6.84212E-05
G10090_29841	18788	Serpinb2	7.260448986	153.3249836	up	9.55658E-13	1.70235E-11
G10090_20902	330096	Shisa3	7.069241291	134.2930926	up	3.7304E-10	4.61011E-09
G10090_1413	228846	D630003M21Rik	7.026870066	130.4063278	up	4.53172E-08	4.0171E-07
G10090_25739	12700	Cish	6.974157939	125.7276315	up	5.63343E-34	6.13809E-32
G10090_545	20306	Ccl7	6.96682846	125.0905036	up	4.69523E-35	5.43276E-33
G10090_2753	319211	Nol4	6.949463767	123.5939031	up	2.55167E-06	1.66568E-05
G10090_10825	12563	Cdh6	6.891753396	118.7475054	up	1.56834E-05	8.76704E-05
G10090_10511	16878	Lif	6.792319161	110.8387981	up	1.39401E-21	6.24203E-20
G10090_19593	11684	Alox12	6.666954379	101.6139298	up	5.83942E-06	3.56112E-05
G10090_930	268902	Robo2	6.660184209	101.1382008	up	2.28589E-25	1.34772E-23
G10090_26255	383548	Serpinb3b	6.601400569	97.10007928	up	0.000375507	0.001500534
G10090_18998	20296	Ccl2	6.500224483	90.52375239	up	7.50396E-18	2.32498E-16
G10090_15116	20303	Ccl4	6.407171583	84.86934178	up	1.56024E-85	1.27501E-82
G10090_20420	20302	Ccl3	6.394034529	84.10003693	up	4.6278E-219	3.0255E-215
G10090_10116	238455	Macc1	6.2837508	77.9107663	up	8.41393E-07	5.97568E-06
G10090_32612	12044	Bcl2a1a	6.235496665	75.34796617	up	7.91346E-67	4.31119E-64
G10090_859	21926	Tnf	6.187308887	72.87281854	up	1.7873E-88	1.79761E-85
G10090_32309	26464	Vnn3	6.115429515	69.33104089	up	4.52881E-22	2.1611E-20
G10090_33977	11535	Adm	6.056226746	66.54354155	up	4.72644E-10	5.77014E-09
G10090_29988	76509	Plet1	6.043562332	65.96195858	up	2.20662E-05	0.000118598
G10090_22230	16160	Il12b	5.9784454	63.05091492	up	9.04701E-16	2.33313E-14
G10090_13780	13655	Egr3	5.962548746	62.35998801	up	3.11832E-23	1.5989E-21
G10090_19061	14609	Gja1	5.935982589	61.22218327	up	1.06732E-39	1.64179E-37
G10090_6724	12515	Cd69	5.897536604	59.61223686	up	3.39503E-20	1.36166E-18
G10090_9494	21939	Cd40	5.895071598	59.51046964	up	1.05928E-26	6.89062E-25
G10090_26551	18126	Nos2	5.832000555	56.96486884	up	1.47269E-46	3.37814E-44
G10090_23580	229898	Gbp5	5.826194276	56.73606843	up	3.0122E-51	8.2051E-49
G10090_12897	213248	Wdr49	5.810460007	56.12065755	up	4.11339E-05	0.000208379
G10090_2280	108078	Olr1	5.796263961	55.57114114	up	6.91415E-45	1.36974E-42
G10090_26937	68725	1110032F04Rik	5.775736194	54.78603132	up	2.93884E-05	0.000153272
G10090_6534	83996	Mmp1b	5.774405691	54.73552904	up	0.000513276	0.001986115
G10090_15542	72281	Sh2d4a	5.774343039	54.73315211	up	7.35594E-10	8.81567E-09
G10090_12624	270893	Tmem132e	5.738337604	53.38407773	up	3.48372E-06	2.19834E-05
G10090_2615	27218	Slamf1	5.728911482	53.03641967	up	0.0008109	0.002970726
G10090_28754	15200	Hbegf	5.721666601	52.77075068	up	2.65326E-15	6.52022E-14
G10090_6360	628900	Serpina3i	5.708254319	52.28243098	up	4.49722E-15	1.07301E-13
G10090_4970	230718	Nt5c1a	5.69956344	51.96842533	up	0.000797263	0.002924041
G10090_21977	12494	Cd38	5.679330296	51.24467868	up	2.54338E-52	7.91778E-50
G10090_31058	210622	Pamr1	5.59569241	48.35832635	up	0.000900315	0.003255426
G10090_12526	18124	Nr4a3	5.575814177	47.69658854	up	6.76296E-24	3.57999E-22
G10090_20164	22271	Upp1	5.557976131	47.11048016	up	4.92949E-17	1.45165E-15
G10090_19731	16763	Lad1	5.54206808	46.59386427	up	4.81317E-20	1.86742E-18
G10090_28120	268481	Krt222	5.536219214	46.4053493	up	0.002656633	0.008432987

G10090_31822	16891	Lipg	5.532628301	46.28998855	up	7.08026E-05	0.000341981
G10090_13653	16007	Cyr61	5.53040825	46.2188113	up	0.001460859	0.00497674
G10090_18702	12703	Socs1	5.522116054	45.95392084	up	1.66266E-61	7.24644E-59
G10090_21595	21983	Tpbg	5.460895874	44.04468023	up	2.52354E-12	4.21934E-11
G10090_5391	14293	Fpr1	5.449775786	43.70649512	up	9.10877E-87	8.50694E-84
G10090_11386	14468	Gbp2b	5.419889977	42.81041682	up	2.34853E-29	1.91919E-27
G10090_33829	210530	P3h2	5.386159334	41.82110666	up	2.30283E-07	1.80081E-06
G10090_20212	19419	Rasgrp1	5.378062029	41.587038	up	6.3174E-101	1.65199E-97
G10090_9912	13661	Ehf	5.373626326	41.45937108	up	0.000227614	0.000962812
G10090_25587	12047	Bcl2a1d	5.339790722	40.49833587	up	1.35463E-74	8.05079E-72
G10090_14199	329278	Tnn	5.329749513	40.21744466	up	7.93025E-06	4.70454E-05
G10090_14544	14038	Wfdc18	5.287601905	39.05950889	up	0.000260331	0.001085405
G10090_13659	232941	Ppm1n	5.268469903	38.54494869	up	1.26235E-18	4.18913E-17
G10090_33881	15978	Ifng	5.253285377	38.14138632	up	0.003220378	0.009989666
G10090_21609	12702	Socs3	5.236146723	37.69096225	up	0	0
G10090_5046	102638514	Gm35060	5.184908139	36.3758269	up	5.68423E-09	5.92202E-08
G10090_25096	20521	Slc22a12	5.182202398	36.30766881	up	0.004191794	0.012570576
G10090_32705	71920	Epgn	5.179309896	36.23494742	up	0.003452069	0.010617691
G10090_10528	14581	Gfi1	5.123376927	34.85701025	up	0.000597894	0.002272517
G10090_29857	11828	Aqp3	5.119365107	34.76021516	up	0.000121102	0.000551438
G10090_14277	24066	Spry4	5.106487003	34.45131159	up	0.008509543	0.023223184
G10090_30523	14086	Fscn1	5.081596167	33.8620208	up	0.000477806	0.00186487
G10090_8030	246779	Il27	5.077956923	33.77671042	up	2.06611E-16	5.6634E-15
G10090_4839	18049	Ngf	5.075516413	33.71962094	up	5.71399E-09	5.93882E-08
G10090_19242	65099	Irak1bp1	5.056467212	33.27731692	up	6.36183E-91	7.5619E-88
G10090_23922	15945	Cxcl10	5.003152593	32.0700032	up	3.36634E-09	3.63159E-08
G10090_20551	226359	C1ql2	5.000991458	32.02199879	up	0.001051612	0.003734338
G10090_9152	171283	Havcr1	4.993757585	31.86183793	up	0.012846587	0.033214283
G10090_16554	214105	Sox30	4.99090242	31.79884412	up	0.001164732	0.004069714
G10090_8473	14584	Gfpt2	4.971428384	31.37249543	up	2.82363E-06	1.82406E-05
G10090_25612	75811	Saxo1	4.966150233	31.25792777	up	0.001184994	0.004129476
G10090_17223	117167	Steap4	4.937953704	30.65294332	up	0.000135778	0.00060923
G10090_32382	20293	Ccl12	4.932887232	30.54548478	up	0.002535785	0.008106451
G10090_18252	11540	Adora2a	4.913928011	30.14669643	up	1.44051E-44	2.7698E-42
G10090_17162	100504518	3425401B19Rik	4.91245599	30.11595264	up	0.009698691	0.026017723
G10090_15121	240913	Adamts4	4.898142787	29.81864482	up	1.39004E-10	1.82845E-09
G10090_29396	21664	Phlda1	4.880201783	29.45012358	up	2.90617E-32	2.81468E-30
G10090_20712	627214	Fam196a	4.876181527	29.36817125	up	0.000384603	0.001530802
G10090_28924	100039674	Gm10634	4.870720522	29.25721462	up	0.002411876	0.007752034
G10090_69	11876	Artn	4.851560942	28.8712355	up	1.51148E-21	6.69917E-20
G10090_15785	80910	Gpr84	4.850022327	28.84046113	up	5.0616E-114	1.6545E-110
G10090_11054	66183	Sptssb	4.846104992	28.76225715	up	0.019833197	0.048146872
G10090_9997	238393	Serpina3f	4.842214228	28.68479352	up	3.24563E-12	5.36493E-11
G10090_15412	70433	Draxin	4.841792737	28.67641433	up	0.017510092	0.043271586
G10090_30722	20471	Six1	4.832229972	28.48696404	up	8.97224E-12	1.3916E-10
G10090_18578	18383	Tnfrsf11b	4.79989046	27.85550294	up	0.000386138	0.001536444
G10090_10059	171171	Ntng2	4.796772857	27.79537337	up	6.02859E-22	2.80512E-20
G10090_24422	64292	Ptges	4.783821571	27.54696688	up	3.68222E-33	3.73217E-31
G10090_8180	22329	Vcam1	4.744025673	26.79748446	up	8.55306E-06	5.05567E-05
G10090_3576	100502846	Gm19410	4.730704763	26.55119267	up	0.000951878	0.003419178
G10090_1262	241134	Nyap2	4.719569144	26.34704292	up	0.006067884	0.017337759
G10090_12163	17022	Lum	4.716717853	26.295023	up	0.016851008	0.041866032
G10090_5307	17880	Myh11	4.689451162	25.80271841	up	0.002916705	0.009149692
G10090_3471	13838	Epha4	4.677783851	25.59488911	up	1.32124E-12	2.32193E-11
G10090_32251	83430	Il23a	4.64018264	24.9364232	up	0.004138229	0.012435611
G10090_6171	74482	Ifitm7	4.628896518	24.74210803	up	0.004273883	0.012784494
G10090_9096	12046	Bcl2a1c	4.59845853	24.22556705	up	7.96124E-16	2.07357E-14
G10090_25546	16365	Acod1	4.595591309	24.17746881	up	2.42799E-30	2.04813E-28
G10090_16409	320910	Itgb8	4.554669594	23.50131525	up	3.75433E-19	1.33029E-17
G10090_20295	629303	Heatr9	4.505633553	22.71594711	up	0.017510513	0.043271586
G10090_24644	12045	Bcl2a1b	4.484557365	22.38650437	up	2.4441E-99	5.326E-96
G10090_26027	114671	4930444G20Rik	4.465264433	22.08912602	up	2.02795E-06	1.34392E-05
G10090_25187	70945	Mmrn1	4.456575162	21.95648431	up	0.007330518	0.020452696

G10090_29508	226695	Ifi205	4.423413219	21.45754649	up	2.87092E-06	1.84913E-05
G10090_1514	23984	Pde10a	4.390063977	20.96722424	up	1.02039E-05	5.93637E-05
G10090_8186	74400	Zfp819	4.365312312	20.61056733	up	0.012768012	0.033044594
G10090_20999	14289	Fpr2	4.354612736	20.45827693	up	4.45466E-26	2.78682E-24
G10090_23954	77596	Adgrf1	4.341899446	20.2787869	up	8.82442E-09	8.8617E-08
G10090_8661	16918	Mycl	4.336543623	20.20364403	up	8.75328E-09	8.80378E-08
G10090_31525	11541	Adora2b	4.321362365	19.99215886	up	1.62552E-33	1.7003E-31
G10090_33596	26878	B3galt2	4.314150249	19.89246629	up	0.000137151	0.000614422
G10090_33103	14294	Fpr3	4.297367767	19.66240332	up	0.008351739	0.022835421
G10090_24559	73230	Bmper	4.272943246	19.33232497	up	0.014369661	0.036482197
G10090_5581	19273	Ptpru	4.272546225	19.32700556	up	1.7842E-05	9.80598E-05
G10090_5855	80859	Nfkbiz	4.270136282	19.29474781	up	1.11507E-43	2.11297E-41
G10090_21658	66282	Tma16	4.268730211	19.27595203	up	1.03132E-74	6.42121E-72
G10090_31185	20715	Serpina3g	4.242928053	18.93427207	up	1.7594E-26	1.13882E-24
G10090_32049	80885	Hcar2	4.242116303	18.92362147	up	8.13356E-35	9.32862E-33
G10090_27421	20415	Shbg	4.236968096	18.85621353	up	0.003814087	0.011608283
G10090_29586	622976	Gm6377	4.221836457	18.65947457	up	9.92186E-41	1.66318E-38
G10090_2802	230775	Adgrb2	4.20259109	18.41221246	up	0.013196082	0.03395764
G10090_5877	216799	Nlrp3	4.202407312	18.40986717	up	3.897E-23	1.99036E-21
G10090_29194	225638	Alpk2	4.167336621	17.96773464	up	7.55667E-17	2.16674E-15
G10090_1642	55932	Gbp3	4.146431491	17.70925346	up	6.95031E-27	4.61296E-25
G10090_13039	19417	Rasgrf1	4.144670212	17.68764675	up	2.80937E-12	4.67334E-11
G10090_7316	54378	Cacng6	4.140726365	17.63936064	up	0.013666192	0.034981493
G10090_21139	240063	Zfp811	4.136986514	17.59369393	up	4.39771E-20	1.73193E-18
G10090_18458	20210	Saa3	4.116294068	17.3431502	up	2.2238E-28	1.70036E-26
G10090_15377	326623	Tnfsf15	4.080585888	16.91915823	up	1.22417E-28	9.58443E-27
G10090_26300	102680	Slc6a20a	4.036342246	16.4081678	up	0.019864615	0.048205241
G10090_32897	378431	Txlnb	4.035470643	16.39825782	up	1.27303E-09	1.46264E-08
G10090_2998	17329	Cxcl9	4.01271267	16.14161102	up	0.000703262	0.002626434
G10090_10115	83382	Siglece	4.001380255	16.01531484	up	4.08873E-96	6.68252E-93
G10090_14738	229900	Gbp7	4.000546346	16.00606031	up	8.32124E-56	2.94055E-53
G10090_3217	14066	F3	3.999241697	15.99159236	up	5.09846E-12	8.17943E-11
G10090_14180	245126	Tarm1	3.999112451	15.99015979	up	3.09603E-19	1.11517E-17
G10090_18890	12823	Col19a1	3.983465125	15.81766922	up	5.70133E-09	5.9351E-08
G10090_22244	104252	Cdc42ep2	3.935643257	15.30194626	up	2.06661E-45	4.3582E-43
G10090_4829	15464	Hrc	3.894488838	14.87160898	up	0.000114597	0.000525152
G10090_22779	16153	Il10	3.871458906	14.63609628	up	1.20577E-09	1.39394E-08
G10090_8946	216233	Socs2	3.853349995	14.45353013	up	7.62165E-06	4.53587E-05
G10090_15355	12769	Ccr9	3.845283504	14.37294212	up	1.12549E-07	9.34927E-07
G10090_15519	23893	Grem2	3.809726881	14.02303656	up	1.29486E-05	7.38348E-05
G10090_23049	16819	Lcn2	3.80356595	13.96327983	up	4.03749E-16	1.07955E-14
G10090_20606	236285	Lancl3	3.800770345	13.93624846	up	6.7991E-45	1.36767E-42
G10090_10405	227326	Gpr55	3.777565141	13.71388231	up	7.4488E-05	0.000358458
G10090_6927	208177	Phldb2	3.761723129	13.56411607	up	0.002060888	0.006731481
G10090_23975	110168	Gpr18	3.741043492	13.37107443	up	4.67043E-25	2.71404E-23
G10090_21070	14469	Gbp2	3.736037374	13.32475751	up	1.78799E-65	8.99155E-63
G10090_27334	13653	Egr1	3.719759336	13.17525825	up	8.24809E-11	1.12572E-09
G10090_3055	21923	Tnc	3.714852095	13.13051953	up	7.12853E-09	7.27032E-08
G10090_33011	231201	AF366264	3.708178347	13.06991947	up	5.91207E-05	0.000289949
G10090_23492	225030	Kcng3	3.681173894	12.82755134	up	2.18823E-14	4.80859E-13
G10090_8621	232035	Ccser1	3.657845145	12.62179459	up	2.14614E-15	5.33474E-14
G10090_24819	14283	Fosl1	3.651152498	12.56337782	up	3.93243E-28	2.9214E-26
G10090_22267	18413	Osm	3.648719419	12.54220777	up	3.13257E-27	2.14442E-25
G10090_6365	72432	Spink5	3.644221061	12.50316176	up	0.001492514	0.005073096
G10090_15051	54199	Ccrl2	3.638288237	12.45185034	up	1.48274E-58	5.87481E-56
G10090_5785	13640	Efna5	3.617768466	12.27599847	up	0.012694756	0.032868106
G10090_8848	12503	Cd247	3.61687354	12.26838584	up	2.68366E-07	2.06892E-06
G10090_13131	17133	Maff	3.607934704	12.1926068	up	7.06513E-25	4.01637E-23
G10090_11209	16323	Inhba	3.590532034	12.04641561	up	0.000871548	0.003163655
G10090_466	30953	Schip1	3.565940343	11.84281666	up	0.000150128	0.000665847
G10090_8057	105245043	LOC105245043	3.565722281	11.84102676	up	4.03859E-05	0.000204748
G10090_17600	246729	Oas1h	3.563704498	11.82447723	up	0.012661906	0.032809042
G10090_361	12153	Bmp1	3.551286082	11.72313143	up	0.001073241	0.003796706

G10090_31218	11847	Arg2	3.549571424	11.70920664	up	2.83752E-10	3.56736E-09
G10090_28332	15937	Ier3	3.543868905	11.66301518	up	3.15887E-28	2.36013E-26
G10090_23183	20299	Ccl22	3.540954378	11.63947739	up	4.05994E-07	3.03162E-06
G10090_25394	12311	Calcr	3.535551556	11.59596964	up	0.004442563	0.013234567
G10090_12275	56619	Clec4e	3.527153348	11.52866334	up	1.27015E-33	1.35018E-31
G10090_10600	17357	Marcksl1	3.508054698	11.37705063	up	1.24619E-57	4.52608E-55
G10090_29054	112419	Ifit1bl2	3.493272304	11.2610723	up	5.02784E-13	9.28517E-12
G10090_17837	21822	Tgtp1	3.4922176	11.25284272	up	2.61391E-06	1.70203E-05
G10090_16064	215257	Il1f9	3.467320149	11.06031178	up	6.87763E-06	4.12122E-05
G10090_5938	193385	Ripor2	3.451559264	10.94013976	up	1.4343E-08	1.38495E-07
G10090_32018	66107	Wfdc21	3.439891243	10.85201651	up	0.001446833	0.004932813
G10090_18162	16165	Il13ra2	3.413620033	10.65619168	up	2.45508E-07	1.90506E-06
G10090_23167	76187	Adhfe1	3.404361174	10.58802186	up	6.17501E-15	1.44692E-13
G10090_21089	20728	Spic	3.397198778	10.53558689	up	6.47554E-09	6.65008E-08
G10090_24351	14204	Il4i1	3.396977226	10.53396909	up	2.80981E-11	4.09112E-10
G10090_21829	218952	Fermt2	3.393281663	10.50702013	up	0.000126494	0.000572091
G10090_5539	12796	Camp	3.392550648	10.50169756	up	6.18577E-05	0.000302125
G10090_12014	100702	Gbp6	3.379649535	10.40820613	up	5.89849E-51	1.57393E-48
G10090_28380	20555	Slfn1	3.369690644	10.33660594	up	1.09359E-06	7.5976E-06
G10090_8272	320159	Togaram2	3.368990118	10.33158803	up	8.25795E-05	0.000391489
G10090_641	270757	Bpifc	3.355187224	10.23321257	up	0.005980643	0.017144686
G10090_1399	268859	Rbfox1	3.341497502	10.13656894	up	5.15492E-39	7.65916E-37
G10090_20631	20656	Sod2	3.340952921	10.13274337	up	1.1091E-65	5.8006E-63
G10090_15154	13660	Ehd1	3.330528137	10.05978898	up	1.34589E-33	1.41915E-31
G10090_7602	58217	Trem1	3.292768773	9.799911853	up	5.79358E-06	3.53646E-05
G10090_10280	208431	Shroom4	3.282814547	9.732527708	up	5.56999E-08	4.8649E-07
G10090_10229	117606	Boc	3.27774972	9.698419914	up	6.97903E-07	5.01102E-06
G10090_23811	17386	Mmp13	3.2744829	9.676483782	up	2.54378E-37	3.39387E-35
G10090_5280	14081	Acsl1	3.268985073	9.639678779	up	1.5561E-38	2.21152E-36
G10090_4907	60440	Iigp1	3.262317757	9.595232449	up	5.02546E-08	4.42179E-07
G10090_32841	230738	Zc3h12a	3.245115538	9.481501462	up	5.33213E-45	1.10663E-42
G10090_21951	217306	Cd300e	3.22243041	9.3335791	up	1.61138E-09	1.81969E-08
G10090_16833	71897	Lypd6b	3.212289946	9.268204941	up	3.80064E-06	2.38223E-05
G10090_17768	104099	Itga9	3.20995425	9.253212032	up	0.006000635	0.01719069
G10090_8222	17112	Tm4sf1	3.195396058	9.160307684	up	1.76568E-05	9.72465E-05
G10090_10333	631323	Gm12250	3.184557711	9.091748088	up	1.01932E-09	1.1953E-08
G10090_9574	20733	Spint2	3.180078051	9.063561416	up	6.84154E-11	9.43599E-10
G10090_33435	15957	Ifit1	3.159394571	8.934546924	up	3.34933E-06	2.12276E-05
G10090_15119	545384	Ifi214	3.138060303	8.803396841	up	0.008122319	0.022324851
G10090_28533	14528	Gch1	3.119756294	8.692410416	up	1.65794E-91	2.16776E-88
G10090_23187	16491	Kcna3	3.119655618	8.691803852	up	2.12509E-11	3.16228E-10
G10090_21029	327957	Scimp	3.118740166	8.686290283	up	2.01619E-07	1.59496E-06
G10090_29261	100039796	Tgtp2	3.118462049	8.684615933	up	2.66881E-06	1.73175E-05
G10090_2313	210297	Lrch2	3.108915573	8.627338564	up	5.96068E-06	3.62324E-05
G10090_29671	65221	Slc15a3	3.098504397	8.565303679	up	5.4233E-115	2.3637E-111
G10090_1599	12654	Chil1	3.088949957	8.508766234	up	0.000299488	0.00122637
G10090_16803	14745	Lpar1	3.083896985	8.479016807	up	2.73363E-15	6.69329E-14
G10090_25202	243659	Styk1	3.080034301	8.456345376	up	0.01578429	0.039543896
G10090_18194	17472	Gbp4	3.075127885	8.427635306	up	1.34522E-05	7.63735E-05
G10090_2518	14990	H2-M2	3.074154406	8.421950561	up	3.39671E-14	7.3046E-13
G10090_4002	66665	Msantd3	3.069297694	8.393646436	up	9.79284E-52	2.72429E-49
G10090_19624	17750	Mt2	3.06621651	8.375739135	up	1.71488E-34	1.91641E-32
G10090_8011	210808	Lacc1	3.063886605	8.362223511	up	3.02382E-40	4.94205E-38
G10090_7100	17295	Met	3.062101062	8.351880456	up	5.90157E-45	1.20567E-42
G10090_30236	56193	Plek	3.057637414	8.326079965	up	3.19872E-53	1.02008E-50
G10090_17595	56221	Ccl24	3.05573653	8.315116807	up	3.62555E-05	0.000185462
G10090_30130	12642	Ch25h	3.049147086	8.277224493	up	9.36187E-05	0.000439205
G10090_22965	244871	Zc3h12c	3.046988212	8.26484757	up	3.05536E-58	1.17496E-55
G10090_29284	100038882	Isg15	3.046637478	8.262838547	up	1.29028E-14	2.87892E-13
G10090_15210	17387	Mmp14	3.039671064	8.22303554	up	2.03594E-13	3.92625E-12
G10090_24780	20511	Slc1a2	3.03813539	8.214287203	up	0.000184732	0.000797677
G10090_974	17863	Myb	3.036652691	8.205849478	up	0.000266792	0.00110916
G10090_24282	77446	Heg1	3.030486103	8.170849637	up	0.002904746	0.00911874

G10090_26035	57349	Ppbp	3.029729569	8.166566052	up	1.57742E-10	2.05018E-09
G10090_27593	74410	Ttll11	3.008445618	8.046969796	up	0.000483992	0.001887325
G10090_9195	21817	Tgm2	3.006829973	8.037963201	up	4.41449E-58	1.64913E-55
G10090_18402	56552	Vmn2r26	3.00016275	8.000902526	up	2.7771E-05	0.000145534
G10090_7626	21667	Tdgf1	2.99612843	7.978560237	up	0.002544499	0.008120535
G10090_7761	17392	Mmp3	2.983318647	7.908031659	up	0.000271538	0.001126741
G10090_30491	20304	Ccl5	2.982939953	7.905956149	up	0.003087894	0.009619779
G10090_15335	209590	Il23r	2.979506251	7.887161867	up	0.001442773	0.004921538
G10090_2893	57444	Isg20	2.976930257	7.873091565	up	1.16387E-09	1.35028E-08
G10090_15778	18035	Nfkbia	2.969646315	7.833441733	up	5.62703E-24	2.99079E-22
G10090_13879	66895	Pxdc1	2.969629853	7.83335235	up	0.000600578	0.00228007
G10090_15448	21462	Tcp10b	2.967070187	7.819466537	up	0.005487262	0.015911721
G10090_4168	18578	Pde4b	2.963065221	7.797789562	up	4.67281E-15	1.10884E-13
G10090_10628	54525	Syt7	2.945786016	7.704952248	up	0.012160079	0.031659305
G10090_23290	20167	Rtn2	2.937053853	7.658457538	up	5.43813E-14	1.13584E-12
G10090_9698	64213	St7	2.932144094	7.63243868	up	3.14442E-29	2.55362E-27
G10090_30625	12890	Cplx2	2.920556774	7.571382608	up	2.64974E-06	1.72365E-05
G10090_11276	436440	Gpr31b	2.920056765	7.568758972	up	5.32017E-07	3.89263E-06
G10090_25470	12029	Bcl6b	2.916704562	7.551192848	up	0.000171519	0.00074679
G10090_20831	223970	Rmi2	2.915988496	7.547445826	up	1.59598E-11	2.41074E-10
G10090_11211	16911	Lmo4	2.911434385	7.523658598	up	1.00713E-49	2.48457E-47
G10090_14634	218194	Phactr1	2.91048065	7.518686511	up	0.00729634	0.02037148
G10090_5291	19739	Rgs9	2.909473094	7.513437408	up	1.04751E-14	2.35736E-13
G10090_16668	23886	Gdf15	2.900513742	7.466922422	up	0.003435044	0.010575277
G10090_731	140486	Igf2bp1	2.89821551	7.455036983	up	0.003192983	0.009909388
G10090_20832	15953	Ifi47	2.895401927	7.44051214	up	9.15241E-15	2.09209E-13
G10090_16517	14857	Gsta1	2.884902561	7.386559583	up	2.6628E-06	1.72956E-05
G10090_26123	58185	Rsad2	2.87272557	7.324476094	up	3.53272E-13	6.64609E-12
G10090_9403	71803	Slc25a18	2.862642682	7.273464342	up	0.001766193	0.005880564
G10090_25773	73707	Gucy2g	2.861742894	7.268929406	up	0.00203387	0.006654867
G10090_2999	223672	Apol9a	2.849362283	7.206817352	up	0.010465196	0.027726939
G10090_10065	19332	Rab20	2.842836873	7.174294029	up	8.10192E-32	7.46004E-30
G10090_3674	21897	Tlr1	2.8414025	7.167164659	up	1.84523E-20	7.44642E-19
G10090_5551	69538	Antxr1	2.840590439	7.16313156	up	1.37814E-07	1.1269E-06
G10090_29452	53314	Batf	2.839928089	7.159843679	up	1.02121E-27	7.32574E-26
G10090_33330	667277	C1rb	2.834846684	7.134669908	up	0.005197192	0.015174919
G10090_7510	73914	Irak3	2.82005285	7.061882663	up	6.83351E-32	6.33675E-30
G10090_29065	99382	Abtb2	2.816810279	7.046028346	up	2.5694E-23	1.32786E-21
G10090_3855	20698	Sphk1	2.806754129	6.99708554	up	1.20219E-05	6.89113E-05
G10090_6140	58203	Zbp1	2.805952296	6.993197721	up	1.41511E-12	2.47691E-11
G10090_9456	11639	Ak4	2.804559698	6.986450621	up	5.92535E-11	8.25069E-10
G10090_7431	11988	Slc7a2	2.803818302	6.982861228	up	2.22135E-28	1.70036E-26
G10090_18479	23872	Ets2	2.78912501	6.912104409	up	1.48905E-23	7.75669E-22
G10090_24356	547347	Gm6034	2.781373843	6.875067331	up	7.58973E-06	4.52305E-05
G10090_946	320405	Cadps2	2.780610942	6.87143274	up	4.70784E-06	2.9104E-05
G10090_11179	15186	Hdc	2.773927461	6.839673516	up	0.002578985	0.00822244
G10090_1713	71898	Apol9b	2.764057361	6.793040053	up	0.001612735	0.005424881
G10090_1236	13618	Ednrb	2.753266838	6.742421585	up	5.13182E-12	8.21999E-11
G10090_19481	276891	Timd4	2.752083009	6.736891241	up	9.56886E-05	0.000448272
G10090_5045	219140	Spata13	2.748306382	6.719278751	up	7.1033E-22	3.28182E-20
G10090_16125	76787	Ppfia3	2.742498876	6.692284956	up	1.65789E-45	3.61281E-43
G10090_26682	15894	Icam1	2.731418485	6.641082796	up	1.56639E-32	1.53989E-30
G10090_8810	70686	Dusp16	2.728513221	6.627722603	up	6.57999E-39	9.55926E-37
G10090_31160	16948	Lox	2.725526143	6.614014198	up	1.49907E-17	4.52663E-16
G10090_8775	21929	Tnfaip3	2.723111686	6.602954413	up	4.3059E-24	2.30736E-22
G10090_15272	15958	Ifit2	2.722809934	6.601573495	up	9.09334E-09	9.11775E-08
G10090_3970	213053	Slc39a14	2.722199355	6.598780162	up	7.55402E-23	3.78425E-21
G10090_32887	12579	Cdkn2b	2.71548252	6.568129262	up	3.61386E-14	7.7461E-13
G10090_25681	12156	Bmp2	2.711543595	6.550221052	up	0.015555138	0.039082136
G10090_16391	22169	Cmpk2	2.708233796	6.535210906	up	3.92866E-10	4.84597E-09
G10090_31776	15220	Foxq1	2.693288442	6.467859958	up	0.005083636	0.01488561
G10090_32752	18356	Olfr56	2.692015988	6.462157835	up	0.000922818	0.003328249
G10090_24776	16145	Igtp	2.683312802	6.423291644	up	1.45926E-12	2.54397E-11

G10090_9719	231655	Oasl1	2.682735	6.42071962	up	2.2166E-07	1.73857E-06
G10090_8108	224840	Treml4	2.660368989	6.321947213	up	2.54849E-06	1.66524E-05
G10090_9310	98170	Tmem132a	2.654759103	6.297412199	up	1.06319E-39	1.64179E-37
G10090_17992	16181	Il1rn	2.650981245	6.280943297	up	2.02164E-21	8.75264E-20
G10090_6498	50518	a	2.650112857	6.277163807	up	0.00625056	0.017797489
G10090_643	240354	Malt1	2.642493139	6.244097819	up	9.35978E-45	1.82655E-42
G10090_935	102637572	Gm38499	2.635260523	6.212872824	up	0.003557064	0.010914953
G10090_5621	67102	D16Ertd472e	2.616323318	6.13185388	up	1.18518E-18	3.96323E-17
G10090_12639	54427	Dnmt3l	2.60459789	6.082219531	up	3.82901E-08	3.44558E-07
G10090_3497	244886	Tmem266	2.601246412	6.068106524	up	0.002365687	0.007622316
G10090_33117	72275	2200002D01Rik	2.597964313	6.05431741	up	0.000476112	0.001858812
G10090_1457	240327	Gm4951	2.596725347	6.049120277	up	0.002077173	0.006782976
G10090_16279	14829	Grpr	2.591854295	6.028730737	up	0.000598518	0.002274229
G10090_23978	56405	Dusp14	2.591297076	6.026402682	up	0.000666324	0.002502781
G10090_3480	19731	Rgl1	2.589540261	6.019068607	up	4.56755E-22	2.17166E-20
G10090_12840	66643	Lix1	2.586205412	6.005171351	up	5.4734E-05	0.000270362
G10090_9594	18715	Pim2	2.583820247	5.995251381	up	6.48657E-25	3.71982E-23
G10090_18667	14102	Fas	2.581771516	5.986743733	up	1.33137E-25	8.05911E-24
G10090_20979	170676	Peg10	2.576195645	5.963650228	up	1.24804E-07	1.02889E-06
G10090_22289	231805	Pilra	2.566117174	5.922134163	up	2.11951E-12	3.60841E-11
G10090_17732	17883	Myh3	2.555722778	5.879619434	up	0.009719574	0.026063051
G10090_12381	20620	Plk2	2.555088624	5.877035546	up	1.59434E-20	6.55536E-19
G10090_12100	20530	Slc31a2	2.544948803	5.835874275	up	8.70624E-42	1.55937E-39
G10090_3575	16560	Kif1a	2.541870787	5.823436605	up	0.019882067	0.048229689
G10090_12332	67742	Samsn1	2.528163999	5.768371171	up	3.42851E-52	1.0425E-49
G10090_1508	20971	Sdc4	2.520045607	5.736002319	up	2.26822E-19	8.261E-18
G10090_9558	211323	Nrg1	2.490497847	5.619718427	up	3.60723E-10	4.46634E-09
G10090_5517	224079	Atp13a4	2.489605466	5.616243419	up	0.001753567	0.005845968
G10090_1353	14058	F10	2.487753803	5.609039734	up	8.72604E-13	1.57037E-11
G10090_4698	12475	Cd14	2.485473615	5.600181617	up	1.14864E-25	7.01797E-24
G10090_16081	19730	Ralgds	2.462016661	5.509863816	up	6.55513E-25	3.74272E-23
G10090_22012	12795	Plk3	2.456687227	5.489547476	up	1.68521E-42	3.14774E-40
G10090_13584	58218	Trem3	2.446058766	5.449254101	up	0.010613327	0.028051193
G10090_7856	73379	Dcbld2	2.442320082	5.435150868	up	1.45123E-21	6.47606E-20
G10090_9034	24088	Tlr2	2.438488511	5.420735116	up	3.21049E-11	4.62813E-10
G10090_19414	15951	Ifi204	2.436805914	5.414416663	up	8.25271E-12	1.29227E-10
G10090_27464	26388	Ifi202b	2.43044927	5.39061274	up	6.70217E-18	2.10146E-16
G10090_16932	70082	Lysmd2	2.422700958	5.36173887	up	0.013578107	0.034783257
G10090_2698	373864	Col27a1	2.421187812	5.356118248	up	0.000375501	0.001500534
G10090_3605	74243	Slx4ip	2.407953325	5.307208857	up	4.16861E-78	3.02803E-75
G10090_20940	223775	Pim3	2.401814405	5.284673732	up	1.1964E-16	3.35684E-15
G10090_9924	227960	Gca	2.401091956	5.282028024	up	3.66779E-12	6.00957E-11
G10090_14425	68016	Cavin4	2.400519984	5.279934322	up	0.017464929	0.043199763
G10090_26125	85031	Pla1a	2.389756281	5.240688216	up	0.000927808	0.0033419
G10090_8492	66441	Magohb	2.38967561	5.240395179	up	1.47404E-13	2.92016E-12
G10090_25895	14362	Fzd1	2.386586632	5.229186887	up	1.24286E-10	1.64311E-09
G10090_23592	320292	Rasgef1b	2.378339033	5.199377958	up	1.77526E-14	3.9332E-13
G10090_32377	23832	Xcr1	2.375643239	5.189671566	up	0.00016	0.000703192
G10090_28557	15162	Hck	2.374531181	5.185672807	up	1.1346E-58	4.63589E-56
G10090_15948	219148	Fam167a	2.374422503	5.185282185	up	0.001662479	0.005576429
G10090_17759	99899	Ifi44	2.373740525	5.182831624	up	2.87299E-06	1.84955E-05
G10090_15160	240660	Slc35g1	2.373594254	5.182306175	up	7.91204E-11	1.08211E-09
G10090_11303	68713	Ifitm1	2.370768818	5.172166855	up	1.43998E-06	9.77936E-06
G10090_886	209176	Ido2	2.370705527	5.171939957	up	2.82174E-13	5.347E-12
G10090_18774	74190	Exoc3l4	2.370194505	5.170108313	up	7.04612E-36	8.29982E-34
G10090_28135	54396	Irgm2	2.36917556	5.166458064	up	4.77241E-09	5.03219E-08
G10090_5430	257632	Nod2	2.365672604	5.153928791	up	3.31783E-19	1.18526E-17
G10090_27798	320116	Fndc9	2.364964672	5.151400372	up	4.02162E-08	3.60155E-07
G10090_7662	233424	Tmc3	2.357714211	5.125576251	up	4.11209E-20	1.62926E-18
G10090_9766	14538	Gcnt2	2.357304035	5.124119194	up	5.98434E-08	5.18772E-07
G10090_3721	66674	Spryd7	2.343369618	5.074865609	up	4.19103E-52	1.2454E-49
G10090_4653	16452	Jak2	2.342603382	5.072170993	up	4.21097E-86	3.67056E-83
G10090_19706	22350	Ezr	2.339761916	5.062190907	up	1.69897E-12	2.91905E-11

G10090_9604	69217	Plekha4	2.338437009	5.05754415	up	1.91958E-06	1.27404E-05
G10090_26711	231931	Gimap6	2.335130636	5.045966528	up	0.000506895	0.001964913
G10090_23788	24063	Spry1	2.333656841	5.040814416	up	0.000515444	0.001993323
G10090_264	18793	Plaur	2.33083562	5.03096663	up	3.09806E-48	7.50131E-46
G10090_30952	18587	Pde6b	2.32285382	5.00320935	up	0.002129814	0.006934093
G10090_6270	320181	Fndc7	2.319580097	4.991869078	up	0.00012546	0.000567806
G10090_22568	381308	Ifi211	2.319435776	4.991369739	up	3.00166E-10	3.75926E-09
G10090_11013	64450	Gpr85	2.318565674	4.988360307	up	3.34656E-08	3.03652E-07
G10090_30761	14425	Galnt3	2.316896397	4.98259184	up	6.28059E-15	1.46903E-13
G10090_29007	225845	Pla2g16	2.30759759	4.95058011	up	2.16283E-09	2.39045E-08
G10090_1029	56318	Acpp	2.299218172	4.921909639	up	1.29423E-13	2.57958E-12
G10090_20189	14064	F2rl2	2.297747794	4.916895843	up	1.27239E-11	1.94125E-10
G10090_9376	212167	Gsap	2.295910047	4.910636545	up	2.10641E-25	1.25188E-23
G10090_5902	68279	Mcoln2	2.287870663	4.883348239	up	7.32104E-47	1.70933E-44
G10090_7755	14571	Gpd2	2.283419909	4.868306193	up	1.59637E-45	3.53772E-43
G10090_654	66597	Trim13	2.272949816	4.833103251	up	5.72571E-41	9.85048E-39
G10090_20453	433470	AA467197	2.264490204	4.80484604	up	2.29402E-08	2.13635E-07
G10090_20496	319765	Igf2bp2	2.262965829	4.799771841	up	3.81249E-17	1.12779E-15
G10090_4373	665563	Mthfd2l	2.255474787	4.774914125	up	4.62934E-16	1.23528E-14
G10090_11921	71586	Ifih1	2.254524915	4.771771351	up	4.95943E-12	7.98579E-11
G10090_3428	18033	Nfkb1	2.250449966	4.758312313	up	1.00228E-64	4.68028E-62
G10090_25621	16835	Ldlr	2.24896446	4.75341532	up	4.9941E-11	7.02884E-10
G10090_22037	319554	Idi1	2.242864584	4.733359777	up	3.36045E-09	3.62823E-08
G10090_14822	16633	Klra2	2.241284349	4.728177998	up	3.72457E-07	2.79717E-06
G10090_14607	16822	Lcp2	2.24110155	4.727578947	up	2.59527E-68	1.47536E-65
G10090_1003	18037	Nfkbie	2.240696474	4.726251736	up	2.65927E-11	3.89361E-10
G10090_1405	22138	Ttn	2.235799401	4.71023618	up	0.000329182	0.001331288
G10090_10624	29816	Hip1r	2.234301301	4.705347592	up	1.31374E-05	7.4748E-05
G10090_11652	74123	Foxp4	2.232691329	4.700099599	up	4.53131E-31	4.00317E-29
G10090_28667	19124	Procr	2.229645455	4.690187033	up	4.25146E-29	3.43135E-27
G10090_12240	26570	Slc7a11	2.228667794	4.687009742	up	7.61629E-14	1.56331E-12
G10090_20345	15368	Hmox1	2.226036684	4.678469615	up	1.71167E-19	6.33996E-18
G10090_3651	71409	Fmnl2	2.225015064	4.67515781	up	6.00417E-63	2.70705E-60
G10090_13487	381560	Xkr8	2.223741696	4.671033189	up	1.39097E-32	1.37779E-30
G10090_3425	64380	Ms4a4c	2.222780464	4.667922032	up	0.000887632	0.003214009
G10090_25288	107449	Unc5b	2.221145275	4.66263428	up	7.88774E-10	9.40987E-09
G10090_24219	21950	Tnfsf9	2.216853812	4.648785345	up	2.50534E-10	3.16801E-09
G10090_17365	50723	Icosl	2.214601888	4.641534649	up	1.10537E-14	2.47903E-13
G10090_15945	17384	Mmp10	2.212850912	4.635904708	up	0.009036904	0.02445819
G10090_17623	93730	Lztfl1	2.211249648	4.630762118	up	1.14579E-21	5.18382E-20
G10090_2423	13032	Ctsc	2.208375609	4.62154621	up	7.03474E-31	6.09134E-29
G10090_18155	56744	Pf4	2.201388732	4.599218478	up	4.24623E-09	4.5285E-08
G10090_9575	57783	Tnip1	2.199334817	4.59267539	up	3.44887E-54	1.12735E-51
G10090_23098	665700	Hmcn2	2.196220487	4.582771923	up	4.25784E-08	3.78201E-07
G10090_23380	108116	Slco3a1	2.194234299	4.576467067	up	1.12874E-10	1.50135E-09
G10090_24527	71436	Flrt3	2.188703918	4.558957367	up	2.42685E-14	5.31509E-13
G10090_19461	72747	Ttc39c	2.177843724	4.524767693	up	7.92567E-27	5.20744E-25
G10090_2328	12633	Cflar	2.176246214	4.519760145	up	2.34949E-41	4.15129E-39
G10090_17086	21426	Tfec	2.173049391	4.509756038	up	3.07675E-33	3.14285E-31
G10090_2003	381290	Atp2b4	2.16336441	4.479582909	up	4.3812E-27	2.96809E-25
G10090_25238	209387	Trim30d	2.161422805	4.47355826	up	6.90418E-06	4.13523E-05
G10090_8983	14254	Flt1	2.159236959	4.466785445	up	0.003308898	0.010228987
G10090_1133	233765	Plekha7	2.158522235	4.46457311	up	0.000523551	0.002022285
G10090_15793	13537	Dusp2	2.157486242	4.46136827	up	1.31189E-10	1.73087E-09
G10090_1409	192656	Ripk2	2.157287038	4.460752296	up	8.76787E-17	2.48676E-15
G10090_4755	56489	Ikbke	2.152317053	4.445411749	up	7.40983E-16	1.94155E-14
G10090_32086	17164	Mapkapk2	2.151877672	4.444058081	up	6.39042E-94	9.28387E-91
G10090_15780	209212	Osgin2	2.14758229	4.430846323	up	1.02532E-27	7.32574E-26
G10090_6964	387609	Zhx2	2.140624106	4.4095276	up	8.45462E-22	3.87874E-20
G10090_6058	18034	Nfkb2	2.136519316	4.396999341	up	5.68341E-19	1.98161E-17
G10090_7949	22035	Tnfsf10	2.133550884	4.38796155	up	0.003070049	0.009571026
G10090_24932	102639868	Gm36079	2.131605146	4.382047571	up	0.000550505	0.002116393
G10090_7126	70859	Lrrc63	2.129818621	4.376624531	up	0.001955705	0.006428066

G10090_29013	81905	Cacng8	2.12656474	4.366764536	up	4.7153E-06	2.91364E-05
G10090_9705	12363	Casp4	2.123972137	4.358924265	up	8.48883E-36	9.90996E-34
G10090_23972	14077	Fabp3	2.11388988	4.32856817	up	4.70614E-26	2.93013E-24
G10090_8515	14962	Cfb	2.112846897	4.325440002	up	0.007752668	0.021471326
G10090_12807	59027	Nampt	2.112283263	4.323750464	up	1.94234E-21	8.46535E-20
G10090_10198	54483	Mefv	2.104566493	4.300685081	up	1.41468E-05	7.98312E-05
G10090_12174	547253	Parp14	2.10394097	4.298820797	up	6.83773E-12	1.07845E-10
G10090_24402	18712	Pim1	2.102020865	4.293103233	up	1.31146E-11	1.99852E-10
G10090_22575	24110	Usp18	2.091186375	4.260983235	up	1.39351E-09	1.59267E-08
G10090_17474	57746	Piwil2	2.087789529	4.25096249	up	2.35373E-12	3.96585E-11
G10090_5747	215798	Adgrg6	2.074104888	4.210830746	up	3.33495E-10	4.14097E-09
G10090_27453	16992	Lta	2.073762945	4.209832828	up	0.017556223	0.043368149
G10090_15843	73656	Ms4a6c	2.06821273	4.193668225	up	2.96411E-08	2.71209E-07
G10090_11882	67338	Rffl	2.065699167	4.186368088	up	1.65971E-50	4.1732E-48
G10090_17562	74178	Stk40	2.064693063	4.183449624	up	3.5703E-22	1.70995E-20
G10090_21108	78749	Filip1l	2.063032779	4.178637991	up	2.7596E-05	0.000144733
G10090_31382	12609	Cebpd	2.060304389	4.170742921	up	2.32763E-19	8.45384E-18
G10090_5937	19024	Ppfibp2	2.056828794	4.160707281	up	2.74344E-33	2.82444E-31
G10090_13010	72333	Palld	2.04986228	4.140664408	up	5.09055E-40	8.21716E-38
G10090_27220	66341	Eid3	2.046812765	4.13192127	up	1.1136E-05	6.43407E-05
G10090_33475	223881	Rnd1	2.044722379	4.125938677	up	2.64554E-11	3.87785E-10
G10090_13331	102644	Oaf	2.043938981	4.123698859	up	5.17007E-09	5.41222E-08
G10090_30787	60533	Cd274	2.038025172	4.106829838	up	7.94646E-24	4.17269E-22
G10090_22052	67138	Herc6	2.03702941	4.103996242	up	9.28754E-10	1.09401E-08
G10090_5134	71720	Osbpl3	2.035653471	4.100084011	up	2.67403E-65	1.29493E-62
G10090_17165	14042	Ext1	2.031141261	4.087280515	up	1.37396E-09	1.57308E-08
G10090_33396	71733	Susd2	2.026721467	4.074778017	up	0.001673034	0.005607516
G10090_13057	59028	Rcl1	2.026297705	4.073581309	up	9.51728E-16	2.44476E-14
G10090_22453	11622	Ahr	2.025245921	4.070612583	up	3.13009E-21	1.33745E-19
G10090_28535	18263	Odc1	2.004918205	4.013659429	up	4.98977E-17	1.46281E-15
G10090_3275	16782	Lamc2	2.003200212	4.008882721	up	7.61259E-10	9.10655E-09
G10090_29152	108673	Ccdc86	2.002951936	4.008192883	up	1.23024E-36	1.577E-34
G10090_5113	15959	Ifit3	1.998420367	3.995622725	up	0.016650119	0.041450934
G10090_8689	230752	Eva1b	1.997800615	3.993906655	up	4.98429E-12	8.01593E-11
G10090_621	15370	Nr4a1	1.996406383	3.990048777	up	2.78593E-11	4.06087E-10
G10090_5425	327959	Xaf1	1.995975307	3.988856731	up	9.89206E-09	9.85062E-08
G10090_25291	12035	Bcat1	1.99356832	3.982207286	up	0.002124842	0.006919628
G10090_19983	230157	Tmeff1	1.988521112	3.968300044	up	1.38313E-08	1.34058E-07
G10090_33722	14782	Gsr	1.987614902	3.965808191	up	5.82636E-27	3.88672E-25
G10090_4375	14062	F2r	1.981913953	3.950167837	up	0.000121794	0.000554092
G10090_4258	67916	Plpp3	1.974774	3.930666601	up	8.83918E-10	1.04496E-08
G10090_20128	15001	H2-Oa	1.972854769	3.925441075	up	1.51182E-05	8.47642E-05
G10090_5986	94090	Trim9	1.971042164	3.920512241	up	0.000329618	0.001332639
G10090_3012	171463	Il17rd	1.964180392	3.901909704	up	8.09037E-05	0.00038452
G10090_20416	12349	Car2	1.957600501	3.884154266	up	9.77298E-06	5.7173E-05
G10090_6196	236312	Ifi209	1.956934443	3.882361459	up	6.5117E-07	4.7065E-06
G10090_11666	667373	Ifit1bl1	1.955321864	3.878024354	up	0.001048456	0.003726292
G10090_2880	22029	Traf1	1.952968178	3.871702708	up	4.79229E-25	2.76032E-23
G10090_3522	14164	Fgf1	1.952571003	3.87063697	up	0.017443266	0.043162509
G10090_20042	14747	Cmklr1	1.951066029	3.86660135	up	1.74516E-06	1.16597E-05
G10090_780	16161	Il12rb1	1.948835663	3.860628311	up	3.97408E-05	0.000201715
G10090_8822	98496	Pid1	1.945154477	3.850790075	up	1.02275E-34	1.16283E-32
G10090_9644	192657	Ell2	1.936153702	3.826840314	up	5.10956E-39	7.65916E-37
G10090_7339	243910	Nfkbid	1.930120857	3.810871221	up	3.08225E-14	6.66122E-13
G10090_11033	22323	Vasp	1.929724596	3.809824646	up	1.25847E-17	3.81775E-16
G10090_19379	13603	Opn3	1.929529607	3.809309758	up	4.40805E-06	2.73542E-05
G10090_1100	75607	Wnk2	1.927698412	3.804477716	up	0.019155322	0.046700697
G10090_2111	18218	Dusp8	1.927453521	3.803831978	up	3.18951E-05	0.000165095
G10090_18351	16362	Irf1	1.926833254	3.802196923	up	1.12796E-15	2.86926E-14
G10090_16730	227659	Slc2a6	1.924503975	3.796063104	up	2.2896E-26	1.46032E-24
G10090_15152	103142	Rdh9	1.920892676	3.786572827	up	0.005688523	0.016407994
G10090_4098	103220	Ttc41	1.918172459	3.77943993	up	2.38821E-18	7.77116E-17
G10090_14539	12874	Cpd	1.913548541	3.767346005	up	8.32419E-38	1.13374E-35

G10090_3580	627049	Zfp800	1.913251234	3.766569719	up	3.72341E-11	5.33811E-10
G10090_3402	54598	Calcrl	1.913219323	3.766486408	up	9.39508E-16	2.41812E-14
G10090_3003	622675	Zfp827	1.910477531	3.759335129	up	1.67612E-11	2.5202E-10
G10090_17818	22341	Vegfc	1.908308435	3.753687204	up	2.02292E-11	3.01937E-10
G10090_4840	15251	Hif1a	1.905609927	3.746672632	up	6.84457E-20	2.6399E-18
G10090_16069	12444	Ccnd2	1.903681322	3.7416674	up	0.007998829	0.022041031
G10090_13356	241447	Cers6	1.895985491	3.721761202	up	9.61578E-11	1.29615E-09
G10090_12975	73112	Abracl	1.8930658	3.714236809	up	5.11129E-11	7.17062E-10
G10090_28970	102871	D330045A20Rik	1.889936979	3.706190346	up	3.12921E-10	3.90778E-09
G10090_2713	74732	Stx11	1.887934713	3.701050223	up	6.78565E-28	4.95656E-26
G10090_16743	100861753	Gm21188	1.884860234	3.693171436	up	0.000103111	0.000479268
G10090_5331	12142	Prdm1	1.875841934	3.670157394	up	4.22149E-07	3.15046E-06
G10090_12053	12927	Bcar1	1.873791508	3.664944899	up	3.44563E-06	2.17956E-05
G10090_19003	20515	Slc20a1	1.869301319	3.653555996	up	1.07947E-09	1.26131E-08
G10090_20	71704	Arhgef3	1.868975607	3.652731239	up	7.54914E-10	9.03892E-09
G10090_29960	12608	Cebpb	1.868258243	3.65091541	up	7.94625E-25	4.49216E-23
G10090_18418	320924	Ccbe1	1.867683465	3.649461156	up	0.001124233	0.003947193
G10090_26532	98402	Sh3bp4	1.866293905	3.645947797	up	7.10778E-21	2.98824E-19
G10090_23033	18412	Sqstm1	1.860062885	3.630234855	up	3.57599E-24	1.93207E-22
G10090_33886	74185	Gbe1	1.859768451	3.62949405	up	1.60731E-24	8.90492E-23
G10090_18212	231070	Insig1	1.859282642	3.628272069	up	6.07853E-11	8.43703E-10
G10090_2287	20441	St3gal3	1.859274335	3.628251178	up	1.15676E-25	7.03469E-24
G10090_29361	69524	Esam	1.857998919	3.625045038	up	8.01966E-05	0.000381715
G10090_13832	104215	Rhoq	1.856771594	3.621962463	up	5.34238E-22	2.50364E-20
G10090_22648	14579	Gem	1.856505731	3.621295058	up	3.03167E-07	2.30997E-06
G10090_10035	380863	Tmem171	1.85644007	3.621130248	up	9.42714E-20	3.55216E-18
G10090_23953	219132	Phf11d	1.854552821	3.616396395	up	9.61593E-07	6.75959E-06
G10090_4395	21353	Tank	1.851841419	3.609606121	up	5.11799E-36	6.13924E-34
G10090_4262	14854	Gss	1.847852779	3.599640371	up	4.23446E-08	3.76594E-07
G10090_13732	270084	Lpcat2	1.846528926	3.596338768	up	1.98318E-28	1.53865E-26
G10090_31578	59014	Rrs1	1.836746424	3.572035513	up	1.38116E-36	1.74248E-34
G10090_315	11529	Adh7	1.836180206	3.570633864	up	1.29221E-07	1.06104E-06
G10090_17598	14726	Pdpn	1.832880409	3.562476283	up	4.97131E-06	3.05882E-05
G10090_5931	16656	Hivep3	1.826052231	3.545655161	up	8.94603E-07	6.32267E-06
G10090_6198	67775	Rtp4	1.825909848	3.54530525	up	7.31192E-06	4.36944E-05
G10090_19582	19012	Plpp1	1.8252429	3.543666658	up	8.53473E-15	1.95775E-13
G10090_6299	17912	Myo1b	1.823004782	3.538173469	up	3.93263E-05	0.000199919
G10090_22343	17390	Mmp2	1.822056434	3.535848433	up	0.001397865	0.004788339
G10090_13651	12224	Klf5	1.821959114	3.535609924	up	0.014787199	0.0374115
G10090_9289	15357	Hmgcr	1.820457063	3.531930766	up	3.21677E-09	3.48462E-08
G10090_5605	243197	Mfsd7a	1.820304605	3.531557545	up	1.49904E-21	6.66666E-20
G10090_17347	20649	Sntb1	1.819445312	3.529454719	up	0.008214794	0.022531663
G10090_14655	52463	Tet1	1.81337909	3.514645277	up	0.016644054	0.041443726
G10090_19928	227731	Slc25a25	1.811556311	3.51020749	up	4.55312E-19	1.60032E-17
G10090_15259	20779	Src	1.808067081	3.501728136	up	1.01867E-28	8.07221E-27
G10090_15904	106931	Kctd1	1.807815838	3.501118367	up	3.2537E-07	2.46908E-06
G10090_24458	330723	Htra4	1.80577528	3.49616986	up	0.009149949	0.024738541
G10090_579	13121	Cyp51	1.803538219	3.490752858	up	1.09798E-08	1.0843E-07
G10090_11865	65963	Tmem176b	1.798902186	3.47955349	up	0.001564809	0.00528133
G10090_14936	17748	Mt1	1.795649954	3.471718454	up	4.38393E-09	4.65637E-08
G10090_17457	18036	Nfkbib	1.795361527	3.471024449	up	4.02881E-55	1.35068E-52
G10090_26207	16164	Il13ra1	1.79530201	3.470881259	up	1.02202E-12	1.81069E-11
G10090_3098	72230	Zfp558	1.794996277	3.470145794	up	9.28345E-07	6.54699E-06
G10090_19713	12562	Cdh5	1.790613644	3.459620146	up	0.008621064	0.023463866
G10090_12955	64008	Aqp9	1.788186707	3.453805182	up	2.45235E-13	4.66731E-12
G10090_692	69601	Dab2ip	1.785620518	3.447667197	up	0.000586329	0.002238321
G10090_24269	14130	Fcgr2b	1.784623046	3.445284321	up	1.86127E-07	1.4821E-06
G10090_2496	73916	Ift57	1.783491631	3.44258346	up	2.03393E-24	1.1127E-22
G10090_19599	80719	Igsf6	1.783028138	3.441477644	up	6.94878E-39	9.98409E-37
G10090_32370	69716	Trip13	1.78102652	3.436706193	up	1.13758E-11	1.74168E-10
G10090_5952	13179	Dcn	1.770653361	3.412084466	up	0.003189695	0.009903886
G10090_32374	20308	Ccl9	1.770460633	3.41162868	up	6.21341E-18	1.96708E-16
G10090_22664	52118	Pvr	1.767852818	3.405467392	up	8.98934E-22	4.10964E-20

G10090_8105	170741	Pilrb1	1.767297426	3.404156647	up	6.62523E-06	3.99009E-05
G10090_10490	207215	Fbxo40	1.765953063	3.40098599	up	0.000948003	0.003407642
G10090_25449	80861	Dhx58	1.760642634	3.388490283	up	2.47913E-07	1.9203E-06
G10090_8729	74481	Batf2	1.755393273	3.376183393	up	7.41147E-09	7.54125E-08
G10090_30819	60322	Chst7	1.755177971	3.375679582	up	0.014144413	0.036022244
G10090_15730	102635944	Gm33153	1.754407556	3.373877413	up	1.22886E-06	8.45208E-06
G10090_7496	219131	Phf11a	1.754237555	3.373479873	up	0.000471278	0.00184186
G10090_8562	216850	Kdm6b	1.754237275	3.373479219	up	7.72847E-07	5.5098E-06
G10090_22181	224796	Clic5	1.75317131	3.370987575	up	1.00144E-05	5.8507E-05
G10090_29720	19015	Ppard	1.749936568	3.363437774	up	4.16574E-25	2.44247E-23
G10090_424	16173	Il18	1.743108689	3.347557157	up	0.000633293	0.002386943
G10090_2670	13025	Ctla2b	1.739519549	3.339239449	up	0.000314781	0.001277394
G10090_8153	67434	Ankrd33b	1.739497788	3.33918908	up	0.000274874	0.001138529
G10090_22569	232314	Ppp4r2	1.738400517	3.336650359	up	1.38599E-36	1.74248E-34
G10090_8309	21938	Tnfrsf1b	1.735137747	3.329112783	up	2.71043E-22	1.31255E-20
G10090_18211	56772	Mllt11	1.734121624	3.32676884	up	3.43538E-05	0.000176355
G10090_29204	20775	Sqle	1.720765062	3.296111536	up	3.10802E-08	2.83582E-07
G10090_33780	20348	Sema3c	1.719099736	3.292308976	up	3.2576E-06	2.06863E-05
G10090_16853	21417	Zeb1	1.709082776	3.2695289	up	9.77394E-05	0.000456408
G10090_8735	11796	Birc3	1.706326341	3.263288054	up	1.48951E-10	1.94559E-09
G10090_32339	14284	Fosl2	1.70375419	3.257475189	up	5.14075E-15	1.21547E-13
G10090_25454	17873	Gadd45b	1.702281496	3.254151675	up	5.45651E-14	1.13786E-12
G10090_20772	269717	Orai2	1.699844031	3.248658357	up	2.48457E-10	3.14785E-09
G10090_7328	269295	Rtn4rl2	1.696903123	3.242042769	up	0.004682751	0.013861663
G10090_17961	76650	Srxn1	1.692186316	3.231460409	up	7.44671E-13	1.34856E-11
G10090_13572	68027	Tmem178	1.691067895	3.228956254	up	6.10428E-05	0.000298815
G10090_2447	327978	Slfn5	1.689766338	3.226044496	up	8.49222E-07	6.02473E-06
G10090_26355	68184	Denr	1.689748623	3.226004884	up	5.27617E-28	3.87562E-26
G10090_1668	236451	Phf11b	1.688784357	3.223849413	up	1.65729E-05	9.1936E-05
G10090_7308	73246	Rassf6	1.688693773	3.223647001	up	6.65632E-05	0.000323417
G10090_18251	14137	Fdft1	1.68640267	3.218531682	up	5.91543E-11	8.24566E-10
G10090_21664	107869	Cth	1.68585502	3.217310153	up	4.02115E-06	2.50843E-05
G10090_6224	433256	Acsl5	1.681367096	3.20731732	up	7.12366E-37	9.22197E-35
G10090_13250	83397	Akap12	1.680773443	3.205997816	up	0.002539402	0.008114046
G10090_17118	244418	Prag1	1.676572185	3.196675242	up	3.49403E-16	9.4001E-15
G10090_6033	22153	Tubb4a	1.676401932	3.196298023	up	5.92459E-06	3.60298E-05
G10090_9039	17096	Lyn	1.676252529	3.195967038	up	1.28316E-40	2.12371E-38
G10090_5453	23792	Adam23	1.674615646	3.192342948	up	0.00552028	0.015986193
G10090_4837	229003	Helz2	1.673403989	3.189662962	up	1.21756E-08	1.18981E-07
G10090_32593	73385	Fam177a	1.673236163	3.189291936	up	0.000103772	0.000480971
G10090_25260	100101807	1700047I17Rik2	1.673236163	3.189291936	up	0.000103772	0.000480971
G10090_32717	20556	Slfn2	1.670389517	3.183005207	up	5.74438E-20	2.22212E-18
G10090_31209	20969	Sdc1	1.670104538	3.182376522	up	4.75402E-16	1.26596E-14
G10090_3886	226075	Glis3	1.661167223	3.162723038	up	2.12839E-08	1.99346E-07
G10090_20690	75767	Rab11fip1	1.657821223	3.155396327	up	2.06741E-09	2.30054E-08
G10090_12985	108079	Prkaa2	1.656664738	3.15286793	up	2.66619E-06	1.73091E-05
G10090_32059	21941	Tnfrsf8	1.654530525	3.148207266	up	0.00827916	0.02267016
G10090_199	52696	Zwint	1.652888157	3.144625377	up	1.64546E-07	1.32559E-06
G10090_16012	50493	Txnrd1	1.652832166	3.144503336	up	8.1498E-18	2.51912E-16
G10090_16480	18619	Penk	1.652824052	3.14448565	up	1.86356E-05	0.000101737
G10090_11861	217344	Rhbdf2	1.652520528	3.143824161	up	9.89117E-25	5.55052E-23
G10090_33079	13197	Gadd45a	1.651300945	3.141167651	up	6.64298E-07	4.79079E-06
G10090_7998	19246	Ptpn1	1.649616724	3.13750275	up	6.88036E-17	1.99469E-15
G10090_14419	16402	Itga5	1.649417993	3.137070589	up	9.36994E-20	3.54081E-18
G10090_3667	75345	Slamf7	1.648158174	3.134332372	up	5.04763E-05	0.000250656
G10090_14309	217151	Arl5c	1.64239381	3.121833967	up	2.5755E-08	2.37815E-07
G10090_4931	19201	Pstpip2	1.634893976	3.105647257	up	1.04114E-13	2.10076E-12
G10090_27165	83921	Cemip2	1.632584716	3.100680156	up	6.16009E-07	4.47462E-06
G10090_26167	214459	Fnbp1l	1.628694398	3.092330241	up	4.83647E-06	2.98146E-05
G10090_29059	66143	Eef1e1	1.628427256	3.091757691	up	1.034E-15	2.6457E-14
G10090_23177	16449	Jag1	1.627745072	3.090296087	up	1.043E-24	5.82789E-23
G10090_4460	107569	Nt5c3	1.625182833	3.084812565	up	2.87455E-11	4.18072E-10
G10090_14772	110460	Acat2	1.62417221	3.082652377	up	2.34668E-08	2.18073E-07

G10090_27114	70178	Abhd17c	1.621029947	3.075945512	up	6.94642E-18	2.16249E-16
G10090_223	23871	Ets1	1.618249376	3.070022813	up	0.001334986	0.004597028
G10090_12136	69543	Capns2	1.610209164	3.052961009	up	0.015363151	0.038696436
G10090_991	54394	Crlf3	1.609442723	3.051339533	up	3.93378E-24	2.11663E-22
G10090_8543	234311	Ddx60	1.60660531	3.045344226	up	4.24789E-06	2.6423E-05
G10090_6434	574428	Zmynd15	1.605455776	3.042918673	up	2.41672E-08	2.24104E-07
G10090_22594	67956	Kmt5a	1.604349916	3.040587097	up	8.52686E-51	2.22977E-48
G10090_1011	11576	Afp	1.603896681	3.039632022	up	4.42223E-14	9.2981E-13
G10090_36	18174	Slc11a2	1.602719725	3.037153289	up	6.90176E-19	2.36232E-17
G10090_29285	72472	Slc16a10	1.592913977	3.016580279	up	2.52101E-12	4.21934E-11
G10090_14921	70110	Ifi35	1.590655591	3.011861837	up	4.9382E-10	6.01743E-09
G10090_30980	213208	Il20rb	1.589660193	3.009784497	up	3.51607E-05	0.000180144
G10090_17593	22420	Wnt6	1.586784532	3.0037912	up	0.007899293	0.021831167
G10090_24794	67712	Slc25a37	1.580224888	2.990164569	up	5.87172E-08	5.10457E-07
G10090_8972	78317	Ccdc88b	1.578589778	2.986777521	up	5.75599E-27	3.85946E-25
G10090_20553	11610	Agtrap	1.576663218	2.98279167	up	4.83179E-22	2.28071E-20
G10090_32401	18950	Pnp	1.574623645	2.978577804	up	1.5099E-23	7.83412E-22
G10090_5895	19698	Relb	1.573627634	2.976522158	up	4.36393E-10	5.34143E-09
G10090_29	16706	Ksr1	1.573606707	2.976478982	up	2.37748E-11	3.51249E-10
G10090_8855	67921	Ube2f	1.572543752	2.974286767	up	1.10854E-20	4.60133E-19
G10090_26315	20128	Trim30a	1.565199086	2.959183345	up	3.49168E-12	5.74261E-11
G10090_13605	276950	Slfn8	1.563704355	2.956119017	up	1.56744E-05	8.76572E-05
G10090_8505	231134	Dok7	1.552704765	2.933666277	up	0.00617662	0.017617649
G10090_6041	272589	Tbcel	1.5524246	2.933096629	up	5.92125E-09	6.12503E-08
G10090_25207	70556	Slc25a33	1.551160283	2.930527312	up	3.08157E-06	1.97218E-05
G10090_28319	211770	Trib1	1.550942955	2.93008589	up	9.64451E-06	5.64973E-05
G10090_30388	243308	A430033K04Rik	1.547869524	2.923850454	up	7.13241E-07	5.10993E-06
G10090_3477	11717	Ampd3	1.54062315	2.909201348	up	7.07014E-20	2.71889E-18
G10090_9450	74568	Mlkl	1.540367153	2.908685175	up	1.67652E-18	5.50765E-17
G10090_22866	19672	Rcn1	1.539924522	2.907792902	up	0.000138289	0.000619222
G10090_3397	26443	Psma6	1.538073675	2.904064859	up	7.05607E-30	5.87631E-28
G10090_5401	80285	Parp9	1.537810904	2.903535965	up	2.88846E-08	2.64472E-07
G10090_9162	12522	Cd83	1.535843941	2.899579997	up	4.59237E-05	0.000230323
G10090_255	14933	Gk	1.534256897	2.896392052	up	2.87224E-18	9.29569E-17
G10090_22325	68836	Mrpl52	1.529545698	2.886949152	up	1.65278E-12	2.84718E-11
G10090_12608	59010	Sqor	1.528998654	2.885854682	up	1.9877E-09	2.22511E-08
G10090_15020	73728	Psd	1.52873871	2.885334756	up	0.001690818	0.005664217
G10090_23215	15163	Hcls1	1.528078734	2.884015132	up	3.50339E-17	1.04106E-15
G10090_15557	545812	Pilrb2	1.526880536	2.881620872	up	8.61776E-05	0.000407071
G10090_32175	215900	Calhm6	1.524192928	2.876257676	up	0.013816235	0.035310258
G10090_30263	22330	Vcl	1.523140813	2.87416087	up	1.29598E-18	4.27903E-17
G10090_18200	13011	Cst7	1.520121044	2.868151128	up	0.001022577	0.003643104
G10090_26397	15982	Ifrd1	1.520003849	2.867918148	up	6.37188E-11	8.81611E-10
G10090_33654	56213	Htra1	1.519857218	2.867626677	up	0.004946484	0.01453377
G10090_27810	23845	Clec5a	1.517575738	2.863095392	up	6.30373E-12	9.99046E-11
G10090_24464	326618	Tpm4	1.516810223	2.861576593	up	3.0744E-10	3.843E-09
G10090_25277	100340	Smpdl3b	1.51676318	2.861483285	up	0.000166625	0.00072888
G10090_11144	18783	Pla2g4a	1.515992693	2.859955488	up	2.09142E-21	9.02485E-20
G10090_32151	230073	Ddx58	1.513843514	2.855698192	up	3.77952E-08	3.40339E-07
G10090_28462	14422	B4galnt2	1.512613365	2.853264248	up	0.008344388	0.02282487
G10090_28208	76072	Rnf183	1.511156428	2.850384272	up	0.000139783	0.000625054
G10090_16082	50934	Slc7a8	1.509037609	2.846201117	up	9.99447E-34	1.07998E-31
G10090_1466	52398	Sept11	1.503117641	2.834545919	up	2.83345E-19	1.02341E-17
G10090_10160	12182	Bst1	1.502253161	2.832847935	up	1.67815E-06	1.12407E-05
G10090_4570	100042856	Gm4070	1.501737361	2.831835302	up	0.000195874	0.000840515
G10090_19701	68770	Phtf2	1.501519812	2.831408311	up	1.98797E-13	3.84507E-12
G10090_2061	17868	Mybpc3	1.500347544	2.829108573	up	0.006104103	0.017433628
G10090_18689	20529	Slc31a1	1.498597326	2.825678495	up	6.64182E-18	2.08754E-16
G10090_3159	19188	Psme2	1.498228538	2.824956277	up	9.20537E-22	4.19373E-20
G10090_13244	21825	Thbs1	1.494626319	2.817911531	up	0.020506223	0.049514103
G10090_25636	71934	Car13	1.49437275	2.817416297	up	5.54947E-16	1.47179E-14
G10090_19044	67065	Polr3d	1.493932541	2.81655675	up	2.36369E-16	6.41187E-15
G10090_9103	19271	Ptprj	1.491214713	2.811255763	up	3.68226E-19	1.3083E-17

G10090_8531	107607	Nod1	1.489922146	2.808738176	up	4.26879E-11	6.04707E-10
G10090_31567	21969	Top1	1.48641764	2.801923642	up	4.58298E-25	2.67511E-23
G10090_19040	14266	Aff2	1.483857952	2.796956764	up	0.000837941	0.003055235
G10090_10824	12122	Bid	1.477882141	2.785395403	up	1.45319E-16	4.03301E-15
G10090_25193	68774	Ms4a6d	1.47591048	2.781591338	up	1.09977E-07	9.1589E-07
G10090_15907	15439	Hp	1.47585247	2.781479494	up	0.004208142	0.012610923
G10090_16139	81703	Jdp2	1.472987922	2.775962198	up	1.33582E-07	1.09366E-06
G10090_31059	15944	Irgm1	1.47235665	2.774747802	up	8.65019E-08	7.28745E-07
G10090_8813	414084	Tnip3	1.470462605	2.771107359	up	1.60568E-13	3.15704E-12
G10090_9464	50498	Ebi3	1.469265215	2.768808383	up	3.3053E-16	8.91069E-15
G10090_20484	15024	H2-T10	1.467912591	2.766213655	up	5.97422E-09	6.17494E-08
G10090_28727	98238	Lrrc59	1.465970965	2.762493299	up	3.08073E-27	2.12003E-25
G10090_5205	329739	Fam102b	1.465199878	2.761017204	up	7.52079E-20	2.8837E-18
G10090_31364	211228	Lrrc25	1.464844408	2.760336994	up	9.32087E-13	1.66717E-11
G10090_12124	67154	Mtdh	1.464600438	2.75987024	up	3.639E-15	8.77858E-14
G10090_2841	11864	Arnt2	1.460756463	2.752526518	up	0.000898735	0.003250611
G10090_28643	54683	Prdx5	1.46014635	2.751362726	up	8.4568E-08	7.14294E-07
G10090_7551	210105	Zfp719	1.45963681	2.750391155	up	2.92185E-14	6.33553E-13
G10090_21031	21928	Tnfaip2	1.458534445	2.74829038	up	8.16408E-10	9.70412E-09
G10090_26694	17167	Marco	1.454599128	2.740803935	up	4.81275E-06	2.96824E-05
G10090_6303	22040	Trex1	1.453802603	2.73929113	up	2.7727E-07	2.13379E-06
G10090_10113	19250	Ptpn14	1.452998167	2.737764148	up	0.001280615	0.004430813
G10090_9311	213311	Fbxl21	1.452282278	2.736405961	up	0.006718293	0.018943646
G10090_2045	114715	Spred1	1.45046483	2.732960919	up	1.85116E-45	3.96785E-43
G10090_25633	69573	Hilpda	1.450258075	2.732569282	up	0.002339279	0.007554311
G10090_2236	68481	Mpzl1	1.449832384	2.731763112	up	0.000773275	0.002846443
G10090_16317	20525	Slc2a1	1.44927122	2.730700746	up	2.04683E-09	2.28347E-08
G10090_3722	231549	Lrrc8d	1.44422737	2.72117053	up	1.50085E-11	2.27388E-10
G10090_6294	14701	Gng12	1.440687249	2.71450144	up	6.91381E-18	2.15747E-16
G10090_22451	100034251	Wfdc17	1.440151259	2.713493135	up	6.48943E-10	7.81301E-09
G10090_21886	110196	Fdps	1.429681592	2.69387254	up	3.19144E-09	3.46004E-08
G10090_13401	20442	St3gal1	1.428555189	2.691770086	up	1.23285E-16	3.45171E-15
G10090_18042	56348	Hsd17b12	1.427089442	2.689036693	up	8.40021E-30	6.95144E-28
G10090_3043	66878	Riok3	1.426389246	2.687731917	up	4.34681E-20	1.71705E-18
G10090_29875	17872	Ppp1r15a	1.425749238	2.68653985	up	3.46033E-15	8.37848E-14
G10090_10418	71566	Clmp	1.422231692	2.679997569	up	0.000614703	0.002328287
G10090_11878	76089	Rapgef2	1.420482523	2.676750224	up	5.20183E-13	9.55252E-12
G10090_609	20491	Sla	1.418986566	2.673976091	up	4.71521E-05	0.00023531
G10090_1260	108670	Epsti1	1.417240677	2.670742115	up	1.00567E-05	5.86527E-05
G10090_31588	170459	Stard4	1.414620744	2.665896451	up	9.92219E-13	1.76028E-11
G10090_1082	108058	Camk2d	1.413007389	2.662916865	up	1.57243E-13	3.09632E-12
G10090_29276	380713	Scarf1	1.409045584	2.655614223	up	6.47633E-12	1.02516E-10
G10090_26196	69065	Chac1	1.409029385	2.655584405	up	4.5737E-06	2.83283E-05
G10090_29130	12228	Btg3	1.408268277	2.654183795	up	5.15416E-07	3.78991E-06
G10090_31988	54711	Plagl2	1.406272306	2.650514267	up	1.10637E-10	1.4746E-09
G10090_24360	24135	Zfp68	1.404760825	2.647738833	up	1.11212E-22	5.50796E-21
G10090_17548	22695	Zfp36	1.400999924	2.640845542	up	1.78201E-05	9.80311E-05
G10090_10561	73102	Slc22a23	1.39927467	2.637689362	up	0.000384049	0.001529116
G10090_24908	13649	Egfr	1.397405778	2.634274667	up	8.77321E-05	0.000413368
G10090_17899	28146	Serp1	1.396927661	2.633401798	up	1.56894E-16	4.32784E-15
G10090_10928	224794	Enpp4	1.396843488	2.633248158	up	8.71002E-12	1.35576E-10
G10090_24063	11655	Alas1	1.396074131	2.631844281	up	2.54744E-15	6.29638E-14
G10090_7578	16443	Itsn1	1.394340678	2.628683918	up	5.06713E-13	9.34454E-12
G10090_10942	170625	Snx18	1.393534397	2.627215233	up	4.56084E-12	7.36209E-11
G10090_16982	12977	Csf1	1.393521711	2.627192132	up	0.000225815	0.000955512
G10090_16156	231713	Naa25	1.392083878	2.624575098	up	6.67064E-16	1.75138E-14
G10090_5766	20737	Spn	1.389815232	2.62045118	up	0.00042763	0.001686146
G10090_19565	15490	Hsd17b7	1.389264951	2.619451864	up	1.02867E-06	7.18089E-06
G10090_3807	13518	Dst	1.38906428	2.619087538	up	2.14163E-11	3.18202E-10
G10090_4121	16418	Eif6	1.387718184	2.616644958	up	1.03592E-12	1.83283E-11
G10090_17825	243771	Parp12	1.386345663	2.614156772	up	8.58508E-10	1.01768E-08
G10090_2426	208846	Daam1	1.382660022	2.607486931	up	2.71903E-08	2.50185E-07
G10090_14796	74198	Dtx2	1.380324876	2.603269867	up	1.77784E-14	3.9332E-13

G10090_1170	17250	Abcc1	1.377427983	2.598047815	up	3.42404E-12	5.63846E-11
G10090_5614	18826	Lcp1	1.377097778	2.59745324	up	2.9176E-96	5.44966E-93
G10090_16824	15277	Hk2	1.376432689	2.596256079	up	1.91572E-14	4.23109E-13
G10090_32306	240672	Dusp5	1.375306465	2.594230131	up	0.000423537	0.00167152
G10090_22501	240047	Mmp25	1.374209979	2.592259197	up	0.006491138	0.01839437
G10090_32014	15519	Hsp90aa1	1.372936163	2.589971394	up	2.14439E-07	1.68702E-06
G10090_21560	69581	Rhou	1.370609223	2.585797364	up	0.001628822	0.005470548
G10090_17824	18563	Pcx	1.370357703	2.585346594	up	3.52011E-07	2.65735E-06
G10090_8307	231991	Creb5	1.366520313	2.578479031	up	0.000344951	0.00138819
G10090_18520	217684	Susd6	1.361109925	2.568827341	up	1.27747E-18	4.22857E-17
G10090_15534	56792	Stap1	1.357183119	2.561844873	up	4.8461E-14	1.01706E-12
G10090_23010	338362	Ust	1.352377798	2.553326088	up	0.00239153	0.0076961
G10090_10706	26410	Map3k8	1.35146996	2.551719875	up	2.80603E-10	3.53117E-09
G10090_20290	72446	Prr5l	1.350803595	2.550541534	up	0.002014086	0.006593435
G10090_20952	108960	Irak2	1.349262446	2.547818392	up	8.62554E-13	1.55557E-11
G10090_8195	226551	Suco	1.348562438	2.546582468	up	9.21639E-08	7.73455E-07
G10090_21224	23960	Oas1g	1.348007069	2.545602343	up	1.9426E-05	0.000105567
G10090_5801	12040	Bckdhb	1.345646326	2.541440274	up	1.14376E-12	2.01817E-11
G10090_14213	14852	Gspt1	1.345644743	2.541437485	up	1.69006E-22	8.27625E-21
G10090_10321	214855	Arid5a	1.344925265	2.540170375	up	1.25287E-08	1.22158E-07
G10090_661	12593	Cdyl	1.34477371	2.539903544	up	4.035E-08	3.61107E-07
G10090_6018	18792	Plau	1.344378468	2.539207805	up	0.001440631	0.004916798
G10090_3363	19325	Rab10	1.342674209	2.536210004	up	3.28412E-14	7.07412E-13
G10090_15444	30955	Pik3cg	1.342210366	2.535394714	up	5.82756E-21	2.47387E-19
G10090_11300	20963	Syk	1.33992878	2.53138822	up	1.89362E-18	6.18979E-17
G10090_19066	328830	A530064D06Rik	1.338836235	2.52947194	up	0.000627119	0.002367086
G10090_14558	320207	Pik3r5	1.338010485	2.52802457	up	8.99531E-16	2.32438E-14
G10090_14588	19073	Srgn	1.337710312	2.527498633	up	4.96701E-10	6.04127E-09
G10090_14719	16648	Kpna3	1.337465669	2.527070073	up	2.09523E-09	2.32556E-08
G10090_22745	74241	Chpf	1.334757521	2.522330844	up	0.000318647	0.001291477
G10090_27358	14629	Gclc	1.329277068	2.512767291	up	4.72173E-06	2.91623E-05
G10090_4660	70615	Ankrd24	1.326888856	2.50861114	up	3.00468E-06	1.92863E-05
G10090_11943	231225	Tapt1	1.325757001	2.506643802	up	2.12759E-15	5.2987E-14
G10090_7992	66628	Thg1l	1.32463859	2.504701346	up	1.10459E-09	1.28721E-08
G10090_10395	381417	Gm14085	1.324240952	2.504011091	up	0.006356054	0.018085395
G10090_2567	269346	Slc28a2	1.322412218	2.500839061	up	0.003150192	0.009795188
G10090_30321	12051	Bcl3	1.319668025	2.496086664	up	0.000324004	0.001311565
G10090_28559	19696	Rel	1.319331084	2.495503771	up	6.99667E-08	6.00272E-07
G10090_7867	11982	Atp10a	1.319323872	2.495491295	up	0.000243761	0.001023497
G10090_8921	23962	Oasl2	1.319218112	2.495308366	up	0.000494883	0.001922912
G10090_1907	27966	Rrp9	1.318730161	2.49446454	up	5.32387E-06	3.26805E-05
G10090_3895	226043	Cbwd1	1.31862404	2.49428106	up	3.26681E-10	4.07184E-09
G10090_11005	319710	Frmd6	1.317709053	2.492699638	up	5.329E-07	3.8969E-06
G10090_29080	105244826	Gm40363	1.315019647	2.48805719	up	0.000108483	0.000499968
G10090_7884	67245	Peli1	1.31490616	2.487861479	up	2.67834E-07	2.06604E-06
G10090_2574	16408	Itgal	1.31477889	2.487642017	up	5.56153E-10	6.73306E-09
G10090_5984	72947	Phykpl	1.314660941	2.487438646	up	4.51729E-06	2.79922E-05
G10090_4992	73158	Larp1	1.310563004	2.480383166	up	2.92678E-28	2.212E-26
G10090_4898	17909	Myo10	1.309794855	2.479062862	up	1.14518E-27	8.0501E-26
G10090_15470	20558	Slfn4	1.305396408	2.471516278	up	6.21227E-11	8.61351E-10
G10090_19463	70546	Zdhhc2	1.303315296	2.467953644	up	1.73919E-06	1.16257E-05
G10090_7908	671535	Parp10	1.303245209	2.467833752	up	6.08067E-09	6.2701E-08
G10090_27170	53380	Psmd10	1.30284164	2.467143514	up	1.69159E-12	2.91021E-11
G10090_10506	239759	Liph	1.30248853	2.466539737	up	0.00064405	0.00242469
G10090_7393	170768	Pfkfb3	1.300000635	2.46228991	up	6.29417E-17	1.83288E-15
G10090_7974	71982	Snx10	1.299415843	2.46129203	up	1.44863E-22	7.1475E-21
G10090_2175	12986	Csf3r	1.298932568	2.460467684	up	3.96273E-07	2.96581E-06
G10090_17576	101985	Usb1	1.298009719	2.4588943	up	6.52688E-22	3.02621E-20
G10090_11507	56360	Acot9	1.297788965	2.45851808	up	1.13182E-25	6.94768E-24
G10090_9853	672511	Rnf213	1.296928344	2.457051921	up	4.26111E-05	0.000214946
G10090_12070	66412	Arrdc4	1.295441547	2.454521063	up	1.57339E-07	1.27224E-06
G10090_542	74096	Hvcn1	1.291939539	2.448570167	up	9.51924E-07	6.69882E-06
G10090_15397	19057	Ppp3cc	1.291307113	2.447497037	up	1.97717E-10	2.54194E-09

G10090_2604	20482	Skil	1.289701203	2.444774165	up	5.33921E-09	5.57589E-08
G10090_1169	76482	Rmc1	1.288600514	2.442909662	up	3.41777E-11	4.91611E-10
G10090_23055	71869	Serpinb12	1.287220751	2.440574432	up	0.000463116	0.00181186
G10090_5750	75234	Rnf19b	1.285946625	2.438419973	up	9.37647E-13	1.67483E-11
G10090_17528	67895	Ppa1	1.284139451	2.435367429	up	4.3578E-13	8.12814E-12
G10090_33110	16649	Kpna4	1.283696638	2.434620045	up	1.417E-12	2.47691E-11
G10090_6708	242960	Fbxl5	1.282035481	2.431818373	up	7.19334E-13	1.30811E-11
G10090_20549	208715	Hmgcs1	1.281221111	2.430446052	up	0.004613593	0.013675523
G10090_27396	67844	Rab32	1.276259308	2.422101474	up	5.31022E-09	5.55449E-08
G10090_8952	213783	Plekhg1	1.275851734	2.421417306	up	0.000395442	0.001569643
G10090_2477	67222	Srfbp1	1.275755613	2.421255982	up	6.19442E-16	1.6329E-14
G10090_30712	230734	Yrdc	1.275569658	2.420943916	up	3.78839E-14	8.08045E-13
G10090_3608	380921	Dgkh	1.274651314	2.41940336	up	6.33952E-11	8.78064E-10
G10090_14380	94180	Acsbg1	1.274608623	2.419331768	up	0.009957972	0.026582141
G10090_8481	93691	Klf7	1.272697122	2.416128393	up	0.000323108	0.001308344
G10090_19718	12266	C3	1.272630519	2.416016853	up	1.63216E-05	9.07336E-05
G10090_14832	12212	Chic1	1.272277617	2.415425937	up	0.017749709	0.043779937
G10090_6153	13684	Eif4e	1.270135401	2.411842003	up	2.99533E-36	3.66018E-34
G10090_26850	20617	Snca	1.266781951	2.406242345	up	0.020682265	0.049893102
G10090_32047	17118	Marcks	1.265076593	2.403399693	up	1.53705E-08	1.4723E-07
G10090_8620	286940	Flnb	1.263732821	2.401162132	up	6.44449E-13	1.17684E-11
G10090_6395	231830	Micall2	1.260536035	2.395847423	up	1.4175E-08	1.36983E-07
G10090_28000	652925	Tmem243	1.257887023	2.391452312	up	2.19407E-10	2.80151E-09
G10090_31638	14489	Mtpn	1.255068703	2.386785146	up	3.55646E-32	3.41917E-30
G10090_5363	218793	Ube2e2	1.254942161	2.386575805	up	1.97402E-13	3.82375E-12
G10090_30832	69747	Zswim7	1.252867071	2.383145557	up	0.000110314	0.000507317
G10090_8702	18987	Pou2f2	1.252629375	2.382752948	up	0.000154095	0.000681594
G10090_394	22033	Traf5	1.250912835	2.379919597	up	1.36262E-14	3.03513E-13
G10090_4327	27052	Aoah	1.250852679	2.379820364	up	0.002827422	0.008895222
G10090_17329	13733	Adgre1	1.250200723	2.378745163	up	3.30611E-07	2.50739E-06
G10090_8014	19106	Eif2ak2	1.24986434	2.378190593	up	6.48284E-08	5.58387E-07
G10090_25163	16477	Junb	1.24771011	2.374642132	up	2.16379E-14	4.76289E-13
G10090_42	17134	Mafg	1.24715072	2.373721569	up	4.80011E-23	2.43261E-21
G10090_17077	21933	Tnfrsf10b	1.245677488	2.37129884	up	0.000119795	0.000546135
G10090_31651	269799	Clec4a1	1.2411195	2.363818883	up	3.09346E-05	0.000160601
G10090_19641	66102	Cxcl16	1.239787342	2.361637184	up	1.2359E-09	1.42373E-08
G10090_5456	76954	St5	1.232216835	2.349277009	up	2.73814E-05	0.000143894
G10090_151	74315	Rnf145	1.231171994	2.347576211	up	6.34868E-17	1.84464E-15
G10090_15032	12508	Cd53	1.230482952	2.346455259	up	1.27628E-07	1.04952E-06
G10090_5680	71865	Fbxo30	1.22921834	2.344399347	up	1.21018E-09	1.3978E-08
G10090_30583	72123	Ccdc71l	1.229146998	2.344283418	up	2.57674E-14	5.61515E-13
G10090_8302	214150	Ago3	1.228743765	2.343628282	up	4.99817E-05	0.000248389
G10090_8993	210106	Tent4a	1.228480798	2.343201136	up	1.36998E-10	1.80388E-09
G10090_6136	209086	Samd9l	1.224213162	2.336279956	up	1.03802E-06	7.23848E-06
G10090_5358	100169864	Gm44504	1.223235209	2.334696809	up	2.19868E-07	1.72555E-06
G10090_23672	213391	Rassf4	1.221848807	2.332454288	up	3.74674E-06	2.35435E-05
G10090_8397	15902	Id2	1.218385129	2.326861159	up	3.99958E-12	6.52866E-11
G10090_30068	12575	Cdkn1a	1.213566736	2.319102741	up	2.3686E-10	3.01847E-09
G10090_24717	67010	Rbm7	1.213354851	2.318762165	up	6.55862E-16	1.72543E-14
G10090_4463	75723	Amotl1	1.213155074	2.318441098	up	7.60393E-05	0.000364448
G10090_12518	20847	Stat2	1.2071957	2.308884011	up	9.70573E-05	0.000453872
G10090_720	16975	Lrp8	1.205913455	2.306832822	up	3.48262E-06	2.19834E-05
G10090_15794	17474	Clec4d	1.20563706	2.306390917	up	1.04076E-09	1.21717E-08
G10090_25896	22038	Plscr1	1.202975235	2.302139465	up	2.47381E-05	0.000131431
G10090_9742	22145	Tuba4a	1.202303434	2.301067707	up	1.68244E-15	4.23853E-14
G10090_3127	67923	Eloc	1.198460784	2.294946917	up	1.93194E-09	2.16825E-08
G10090_2317	69185	Dtwd1	1.197699208	2.29373577	up	3.59929E-10	4.46073E-09
G10090_27607	20810	Srm	1.193103364	2.28644048	up	2.09195E-05	0.000113072
G10090_1834	107527	Il1rl2	1.191586088	2.284037105	up	0.001304949	0.004511423
G10090_17190	12369	Casp7	1.188014277	2.278389301	up	1.50996E-09	1.71527E-08
G10090_11364	73205	C9orf72	1.186144967	2.275439089	up	7.85639E-22	3.61698E-20
G10090_4605	18081	Ninj1	1.183652458	2.271511262	up	6.37353E-10	7.68055E-09
G10090_5868	20112	Rps6ka2	1.183095703	2.270634825	up	5.44471E-09	5.677E-08

G10090_10819	108943	Trmt10a	1.182605586	2.269863569	up	1.15067E-06	7.93794E-06
G10090_28421	64705	Dpys	1.181974246	2.268870466	up	6.2485E-05	0.000304979
G10090_3957	319520	Dusp4	1.181764513	2.268540653	up	4.22804E-14	8.93079E-13
G10090_10320	69577	Fastkd3	1.181343181	2.267878233	up	8.72762E-12	1.35688E-10
G10090_28481	17299	Mettl1	1.18091897	2.267211482	up	7.99919E-09	8.0889E-08
G10090_28138	208768	Sde2	1.180800041	2.267024591	up	1.1224E-13	2.26123E-12
G10090_7472	52874	Pum3	1.180399725	2.26639563	up	1.46361E-11	2.22004E-10
G10090_26579	74754	Dhcr24	1.18029395	2.26622947	up	3.89148E-07	2.91583E-06
G10090_2650	434204	Whamm	1.177484076	2.261819928	up	3.49533E-08	3.16273E-07
G10090_22545	68523	Ciao2b	1.177234253	2.261428296	up	3.42339E-18	1.09708E-16
G10090_33459	18128	Notch1	1.176772032	2.26070388	up	7.46384E-07	5.32986E-06
G10090_26886	110558	H2-Q9	1.176520349	2.260309528	up	0.003062744	0.009552809
G10090_18909	13058	Cybb	1.173384813	2.255402332	up	7.59733E-09	7.71235E-08
G10090_4610	16859	Lgals9	1.17290299	2.254649212	up	7.27259E-19	2.46985E-17
G10090_23368	192156	Mvd	1.171569248	2.252565798	up	2.23424E-09	2.46521E-08
G10090_2281	217119	Xylt2	1.166096353	2.244036828	up	8.03229E-11	1.09741E-09
G10090_26893	55989	Nop58	1.161196785	2.236428732	up	1.23301E-13	2.46885E-12
G10090_564	503610	Zdhhc18	1.160973265	2.236082264	up	2.06059E-13	3.96209E-12
G10090_10273	69077	Psmd11	1.160732645	2.23570935	up	1.9244E-15	4.82022E-14
G10090_15776	100037283	Rnaset2a	1.160174962	2.23484529	up	5.07228E-06	3.11801E-05
G10090_26114	66205	Cd302	1.159015628	2.233050113	up	5.71367E-09	5.93882E-08
G10090_13580	69028	Mitd1	1.156427958	2.229048423	up	0.00010659	0.000492286
G10090_14364	72075	Ogfr	1.155359684	2.227398489	up	5.58269E-10	6.75242E-09
G10090_7095	22030	Traf2	1.155150325	2.227075279	up	4.27706E-09	4.55766E-08
G10090_2538	108067	Eif2b3	1.154600261	2.226226313	up	3.31447E-11	4.77276E-10
G10090_29214	84035	Kremen1	1.154430459	2.225964306	up	1.19134E-12	2.09646E-11
G10090_9969	66234	Msmo1	1.151627268	2.221643404	up	1.45465E-06	9.87001E-06
G10090_12502	59029	Psmd14	1.150057064	2.219226721	up	1.68422E-25	1.00553E-23
G10090_8688	20846	Stat1	1.149314518	2.218084793	up	6.00464E-07	4.36656E-06
G10090_15281	13690	Eif4g2	1.147296723	2.214984683	up	1.50352E-16	4.15614E-15
G10090_13453	67724	Pop1	1.146798378	2.214219702	up	2.50825E-06	1.64306E-05
G10090_17557	17254	Slc3a2	1.146119793	2.213178468	up	4.13002E-11	5.86319E-10
G10090_31470	12549	Arhgap31	1.14506931	2.211567552	up	6.09938E-19	2.10977E-17
G10090_4850	19255	Ptpn2	1.144937404	2.211365358	up	1.82215E-12	3.11433E-11
G10090_28106	69237	Gtpbp4	1.144861526	2.211249055	up	2.54668E-21	1.09532E-19
G10090_21580	17973	Nck1	1.144642794	2.210913825	up	3.86622E-14	8.23304E-13
G10090_5738	215418	Csrnp1	1.144382092	2.210514339	up	8.00122E-05	0.000380976
G10090_9810	50790	Acsl4	1.143770642	2.209577665	up	1.1939E-28	9.40374E-27
G10090_270	76884	Cyfip2	1.143223136	2.208739285	up	1.84524E-08	1.74199E-07
G10090_13424	224093	Fam43a	1.14257722	2.207750621	up	1.90726E-07	1.51596E-06
G10090_12082	239273	Abcc4	1.140720822	2.204911609	up	1.54507E-08	1.47756E-07
G10090_17302	71306	Mfap3l	1.138712867	2.201844929	up	0.000724023	0.002690904
G10090_20566	26442	Psma5	1.138316615	2.201240252	up	1.87158E-16	5.15176E-15
G10090_16754	70617	Fam241a	1.137814282	2.200473933	up	2.95101E-06	1.89791E-05
G10090_4806	15039	H2-T22	1.135879159	2.197524361	up	0.007681747	0.021292948
G10090_31173	99929	Tiparp	1.134075198	2.194778271	up	2.24752E-05	0.000120386
G10090_22228	13664	Eif1a	1.130432933	2.189244266	up	2.26242E-16	6.16275E-15
G10090_8569	13163	Daxx	1.128472375	2.186271201	up	2.93038E-06	1.88557E-05
G10090_30528	209200	Dtx3l	1.127809972	2.185267621	up	0.000301651	0.001233683
G10090_33619	353258	Ltv1	1.126102312	2.182682539	up	6.6204E-13	1.20728E-11
G10090_22628	12462	Cct3	1.125583213	2.181897325	up	4.76778E-20	1.85532E-18
G10090_24989	56715	Rabgef1	1.123981164	2.179475768	up	3.93841E-11	5.60944E-10
G10090_30725	193838	Eme2	1.123262159	2.178389841	up	0.000336839	0.001358893
G10090_943	108899	2700081O15Rik	1.123183686	2.178271354	up	0.010540821	0.027909605
G10090_938	56736	Rnf14	1.121821402	2.176215462	up	1.15577E-34	1.30273E-32
G10090_15015	241694	Ralgapa2	1.12126081	2.175370009	up	5.39752E-08	4.7269E-07
G10090_4602	216238	Eea1	1.121259863	2.175368581	up	1.45826E-19	5.41668E-18
G10090_5003	102747	Lrrc49	1.12038022	2.174042614	up	1.82371E-08	1.72291E-07
G10090_24986	67417	Ears2	1.120102205	2.173623705	up	4.75524E-05	0.000237127
G10090_27312	66403	Asf1a	1.119752013	2.173096157	up	6.57016E-09	6.73764E-08
G10090_8615	56312	Nupr1	1.117932666	2.170357445	up	0.000232785	0.000981192
G10090_23285	330817	Dhps	1.117847973	2.170230039	up	1.01244E-17	3.10016E-16
G10090_22410	11898	Ass1	1.116196559	2.167747257	up	1.68947E-05	9.3482E-05

G10090_2861	72244	1600014C10Rik	1.115338124	2.166457784	up	0.004121431	0.012407947
G10090_18358	11491	Adam17	1.114273398	2.164859503	up	1.53378E-12	2.65971E-11
G10090_31212	13178	Dck	1.1132277	2.163290931	up	0.002596961	0.008271685
G10090_6674	11973	Atp6v1e1	1.112973008	2.16290906	up	1.27179E-21	5.734E-20
G10090_30630	19181	Psmc2	1.110969063	2.159906802	up	5.7579E-15	1.35648E-13
G10090_10533	109042	Cavin3	1.110873865	2.159764283	up	0.003936244	0.011925841
G10090_10407	71712	Dram1	1.110762352	2.159597351	up	1.93359E-22	9.39839E-21
G10090_19287	20821	Trim21	1.11006724	2.158557075	up	0.000536139	0.002066634
G10090_2467	210148	Slc30a6	1.109004281	2.156967264	up	3.93656E-13	7.39518E-12
G10090_28041	66220	Zdhhc12	1.10735343	2.154500496	up	5.73887E-12	9.15069E-11
G10090_16392	231986	Jazf1	1.106257026	2.152863763	up	2.40739E-05	0.000128319
G10090_6178	56738	Mocs1	1.104833021	2.150739837	up	4.50716E-12	7.29345E-11
G10090_17727	80294	Pofut2	1.104140393	2.149707531	up	1.96118E-11	2.93056E-10
G10090_23421	225471	Ticam2	1.103449137	2.148677761	up	1.10083E-09	1.28513E-08
G10090_2508	18187	Nrp2	1.103135565	2.148210794	up	5.74346E-16	1.51709E-14
G10090_7114	55948	Sfn	1.100658994	2.144526278	up	0.00490202	0.014419328
G10090_11740	12389	Cav1	1.098932647	2.141961646	up	4.62491E-05	0.000231511
G10090_17055	16068	Il18bp	1.094386656	2.135222859	up	0.000316112	0.001281997
G10090_14337	100604	Lrrc8c	1.093754606	2.134287616	up	7.868E-24	4.14815E-22
G10090_19604	30877	Gnl3	1.093500583	2.133911854	up	2.82033E-07	2.16662E-06
G10090_13460	66980	Zdhhc6	1.091162034	2.130455672	up	1.71171E-20	6.98765E-19
G10090_1670	268566	Gphn	1.090461743	2.12942179	up	1.4515E-08	1.39568E-07
G10090_33847	21854	Timm17a	1.090192031	2.129023731	up	1.68517E-13	3.30339E-12
G10090_20662	50884	Nckap1	1.089512106	2.128020584	up	3.52721E-07	2.66118E-06
G10090_21479	11745	Anxa3	1.088371739	2.126339172	up	8.37603E-05	0.000396799
G10090_5609	83431	Ndel1	1.085993154	2.122836344	up	6.57263E-15	1.53186E-13
G10090_23983	112407	Egln3	1.084751294	2.12100981	up	0.002824632	0.008888582
G10090_2365	21899	Tlr6	1.08297773	2.118403968	up	9.0961E-10	1.07242E-08
G10090_6816	69926	Dnah17	1.078019157	2.111135468	up	0.004077511	0.012312578
G10090_27483	19053	Ppp2cb	1.076805116	2.109359676	up	7.38257E-17	2.12615E-15
G10090_32877	235036	Ppan	1.076120164	2.108358447	up	4.0443E-12	6.58521E-11
G10090_7257	625098	Slc38a6	1.074837187	2.106484334	up	9.4961E-11	1.28266E-09
G10090_8791	54369	Nme6	1.074761804	2.106374271	up	2.6604E-08	2.45135E-07
G10090_30717	12362	Casp1	1.074185326	2.105532765	up	2.7374E-08	2.51345E-07
G10090_1280	66973	Mrps18b	1.073836934	2.105024368	up	2.57832E-09	2.82814E-08
G10090_25452	18938	Ppp1r14b	1.072274309	2.102745589	up	5.13631E-12	8.21999E-11
G10090_19825	66861	Dnajc10	1.071765528	2.102004165	up	2.57829E-12	4.29989E-11
G10090_7448	73919	Lyrm1	1.070971118	2.100847029	up	0.000267586	0.001111751
G10090_29354	11761	Aox1	1.070159052	2.099664835	up	0.008625111	0.023469996
G10090_9945	320705	Bend6	1.070143873	2.099642745	up	0.013882835	0.035459675
G10090_14557	68215	Fam98b	1.06694068	2.094986113	up	3.71055E-17	1.10012E-15
G10090_19761	16190	Il4ra	1.066546695	2.094414071	up	9.98928E-11	1.34234E-09
G10090_13317	17869	Myc	1.066051412	2.093695173	up	0.003734236	0.011413077
G10090_12292	72580	Zup1	1.064671061	2.091692912	up	0.00010632	0.000491387
G10090_7945	192185	Nadk	1.064243438	2.091073014	up	4.82362E-12	7.77667E-11
G10090_23617	18720	Pip5k1a	1.063889316	2.090559806	up	4.06549E-20	1.61569E-18
G10090_10632	76167	Snrnp35	1.062191117	2.088100452	up	5.87185E-06	3.57589E-05
G10090_9466	100043332	Ankrd66	1.06179527	2.087527597	up	0.002510476	0.008031435
G10090_13741	217026	Heatr6	1.0608981	2.086229829	up	1.05192E-32	1.04992E-30
G10090_6828	76357	Trmt5	1.060555784	2.085734876	up	1.14725E-10	1.52442E-09
G10090_1573	228608	Smox	1.060241463	2.085280504	up	0.000109037	0.00050232
G10090_2256	243725	Ppp1r9a	1.058904026	2.08334826	up	0.0092093	0.024868152
G10090_5705	83490	Pik3ap1	1.05867047	2.083011018	up	1.38676E-12	2.43382E-11
G10090_22529	11987	Slc7a1	1.058538794	2.082820907	up	3.4431E-06	2.17902E-05
G10090_13664	67036	Mrpl45	1.058491317	2.082752366	up	1.90287E-17	5.68036E-16
G10090_29789	20773	Sptlc2	1.057231316	2.080934156	up	3.72755E-09	3.9949E-08
G10090_13181	71776	Tha1	1.052479017	2.074090746	up	0.000527328	0.002034467
G10090_6550	80750	N4bp1	1.050577703	2.071359123	up	6.04409E-06	3.67053E-05
G10090_27338	77891	Ube2s	1.050346832	2.071027675	up	1.12059E-11	1.71766E-10
G10090_21986	21753	Tes	1.048127262	2.067843871	up	1.59671E-11	2.41074E-10
G10090_15473	68303	Fam114a1	1.047706498	2.06724087	up	1.44621E-08	1.39346E-07
G10090_13704	20788	Srebf2	1.045535777	2.064132779	up	6.16079E-06	3.73273E-05
G10090_21969	64010	Sav1	1.044905098	2.063230633	up	3.32813E-24	1.80562E-22

G10090_28845	11479	Acvr1b	1.044803892	2.063085901	up	2.27431E-12	3.85189E-11
G10090_30316	67101	2310039H08Rik	1.043021322	2.06053836	up	0.011071759	0.029139483
G10090_21757	74356	4931428F04Rik	1.042101516	2.05922506	up	0.00659426	0.018646184
G10090_27440	26374	Cop1	1.0406881	2.057208614	up	3.14773E-09	3.41548E-08
G10090_26895	13063	Cycs	1.040367696	2.056751785	up	4.10959E-18	1.31056E-16
G10090_17025	60406	Sap30	1.038785074	2.054496786	up	6.59062E-06	3.97291E-05
G10090_7573	12521	Cd82	1.038637892	2.0542872	up	3.23547E-16	8.74045E-15
G10090_21773	211378	6720489N17Rik	1.038594069	2.0542248	up	0.000960577	0.003445689
G10090_11026	17087	Ly96	1.035867189	2.050345717	up	1.46801E-06	9.94519E-06
G10090_22237	19166	Psma2	1.035068413	2.049210818	up	4.14758E-13	7.75817E-12
G10090_10353	321006	Dcaf1	1.033580515	2.047098491	up	1.4655E-07	1.19015E-06
G10090_25117	56613	Rps6ka4	1.032889179	2.04611776	up	1.54835E-18	5.09943E-17
G10090_4741	76820	Fam49a	1.031881703	2.044689395	up	0.001048492	0.003726292
G10090_11304	105245911	Gm41291	1.03170679	2.044441511	up	0.000626976	0.002367086
G10090_24585	80291	Rilpl2	1.031257119	2.043804383	up	1.33483E-21	5.99756E-20
G10090_5288	218756	Slc4a7	1.030802369	2.043160259	up	2.84178E-06	1.83397E-05
G10090_12028	68262	Agpat4	1.030551338	2.042804778	up	1.62552E-08	1.5491E-07
G10090_9139	104662	Tsr1	1.030428796	2.042631269	up	2.60512E-08	2.4037E-07
G10090_18900	22682	Zfand5	1.026695457	2.037352277	up	1.80034E-09	2.02751E-08
G10090_28509	18442	P2ry2	1.025990966	2.036357648	up	2.75946E-09	3.01672E-08
G10090_17310	74112	Usp16	1.025653409	2.035881243	up	2.96225E-07	2.26764E-06
G10090_2582	19175	Psmb6	1.024166988	2.033784738	up	2.56786E-16	6.95129E-15
G10090_7748	233016	Blvrb	1.024063836	2.033639328	up	6.81123E-11	9.4041E-10
G10090_21673	68567	Cgref1	1.023907546	2.033419033	up	0.007386452	0.020579132
G10090_13001	235293	Sc5d	1.023311686	2.032579365	up	0.000248779	0.00104156
G10090_33154	215449	Rap1b	1.022564142	2.031526441	up	6.03087E-15	1.41823E-13
G10090_20304	17768	Mthfd2	1.021266422	2.029699882	up	1.03063E-07	8.60507E-07
G10090_5817	434223	Gm1966	1.019810683	2.027652864	up	0.000862597	0.003134644
G10090_6439	52857	Gramd1a	1.017097736	2.023843504	up	1.58646E-17	4.77948E-16
G10090_28495	192174	Rwdd4a	1.016263334	2.022673326	up	1.10632E-08	1.09171E-07
G10090_1256	69181	Dyrk2	1.01432534	2.019958061	up	8.5061E-08	7.17068E-07
G10090_14369	18227	Nr4a2	1.014212293	2.019799787	up	0.010281956	0.027313404
G10090_12448	20288	Msr1	1.013378396	2.018632653	up	9.53627E-15	2.17224E-13
G10090_33971	53382	Txnl1	1.012941674	2.01802168	up	1.92946E-13	3.74299E-12
G10090_8546	75624	Metap1	1.011736114	2.016336064	up	1.71735E-21	7.53501E-20
G10090_5012	56436	Adrm1	1.011400491	2.015867047	up	1.03611E-19	3.89284E-18
G10090_22508	22019	Tpp2	1.008069607	2.011218196	up	3.98787E-13	7.47011E-12
G10090_993	51886	Fubp1	1.007607376	2.010573916	up	1.57742E-10	2.05018E-09
G10090_17110	58244	Stx6	1.005891315	2.008183795	up	1.10067E-07	9.16054E-07
G10090_7221	68147	Gar1	1.005818849	2.008082927	up	0.010916057	0.028775695
G10090_33594	18477	Prdx1	1.004824538	2.006699425	up	2.96658E-09	3.22964E-08
G10090_30228	246154	Vasn	1.004733567	2.006572895	up	0.012019349	0.031317853
G10090_14494	214133	Tet2	1.00396022	2.005497573	up	8.73159E-13	1.57037E-11
G10090_17535	72098	Tmem68	1.003066284	2.004255293	up	4.54781E-15	1.08311E-13
G10090_14530	63986	Gmfg	1.002866949	2.003978388	up	2.15464E-19	7.93575E-18
G10090_24583	72515	Wdr43	1.000094266	2.000130685	up	2.63824E-10	3.32963E-09
G10090_11698	56480	Tbk1	0.868490871	1.825752076	up	6.84797E-14	1.41226E-12
G10090_1114	18708	Pik3r1	0.721214897	1.648569715	up	0.002429396	0.007802591
G10090_3214	105787	Prkaa1	0.271795554	1.20730949	up	0.01576843	0.039511733
G10090_25693	56717	Mtor	-0.190004461	0.87660301	down	0.136529683	0.236471799
G10090_998	22241	Ulk1	-0.475252857	0.719340702	down	0.000228198	0.000964036
G10090_5940	19128	Pros1	-1.000859879	0.499702077	down	0.001150626	0.004027962
G10090_8781	19714	Rev3l	-1.001072643	0.499628388	down	9.49652E-15	2.16696E-13
G10090_21751	233057	Zfp940	-1.002572235	0.499109326	down	0.013900183	0.035490118
G10090_26432	226844	Flvcr1	-1.002820933	0.499023294	down	1.42054E-12	2.47979E-11
G10090_5716	94218	Cnnm3	-1.003296498	0.498858825	down	6.40134E-09	6.57999E-08
G10090_12900	320678	Iffo1	-1.003419717	0.49881622	down	1.69431E-09	1.90975E-08
G10090_4229	16592	Fabp5	-1.003614957	0.49874872	down	6.66013E-09	6.82454E-08
G10090_14098	67971	Tppp3	-1.004143789	0.498565933	down	0.012408775	0.032236188
G10090_2415	19224	Ptgs1	-1.004737317	0.498360864	down	0.011530899	0.030224889
G10090_3840	12334	Capn2	-1.005639129	0.498049441	down	5.95217E-08	5.16764E-07
G10090_14401	27015	Polk	-1.005737769	0.49801539	down	6.11328E-06	3.7091E-05
G10090_426	72278	Ccpg1	-1.007222142	0.497503251	down	4.71447E-20	1.84005E-18

G10090_1395	104806	Fancm	-1.007309357	0.497473176	down	7.38812E-05	0.000355669
G10090_21626	69178	Snx5	-1.007557456	0.497387634	down	1.66412E-10	2.15857E-09
G10090_5096	109065	Dnaaf2	-1.008036461	0.497222518	down	0.00109198	0.003854655
G10090_15764	50782	Rgs11	-1.010265693	0.49645481	down	0.001744174	0.005820832
G10090_4314	52504	Cenpo	-1.010587399	0.496344118	down	0.001360423	0.004672321
G10090_1581	209773	Dennd2a	-1.010594336	0.496341731	down	1.08562E-05	6.27798E-05
G10090_11187	16542	Kdr	-1.010807841	0.496268283	down	0.000625456	0.002362853
G10090_18973	212980	Slc45a3	-1.011178879	0.496140667	down	0.016899908	0.041968907
G10090_22701	16518	Kcnj2	-1.011345812	0.496083263	down	5.56909E-06	3.40579E-05
G10090_21188	14431	Gamt	-1.012393851	0.495723016	down	1.11508E-06	7.71816E-06
G10090_14957	140579	Elmo2	-1.012561128	0.495665542	down	4.91536E-10	5.99518E-09
G10090_26115	74525	Fam234b	-1.013882092	0.495211907	down	4.68877E-05	0.00023417
G10090_3295	60534	Fancg	-1.01472261	0.494923479	down	0.001029901	0.003668196
G10090_9187	11302	Aatk	-1.015540994	0.494642808	down	7.95973E-05	0.000379415
G10090_30607	66066	Gng11	-1.015716747	0.494582553	down	0.002649146	0.008417396
G10090_5719	78887	Sfi1	-1.016040388	0.494471615	down	0.000312999	0.001272135
G10090_21653	210992	Lpcat1	-1.018578528	0.493602454	down	0.002680625	0.00849883
G10090_10427	243842	Bicra	-1.019278462	0.493363037	down	9.03835E-06	5.32086E-05
G10090_1221	195209	Zfp469	-1.021564573	0.492581868	down	0.002109305	0.006874169
G10090_13189	75687	Ripor1	-1.021568377	0.492580569	down	5.89889E-08	5.12478E-07
G10090_13968	19651	Rbl2	-1.022814961	0.49215513	down	7.17247E-09	7.30943E-08
G10090_32757	14070	F8a	-1.023293516	0.491991905	down	8.52183E-05	0.000402976
G10090_25862	16329	Inpp1	-1.023529185	0.491911543	down	0.002726331	0.008616577
G10090_2194	14421	B4galnt1	-1.02370457	0.491851746	down	6.0693E-13	1.10987E-11
G10090_33876	18803	Plcg1	-1.024037998	0.491738085	down	2.83974E-08	2.60194E-07
G10090_22297	70726	Angptl6	-1.024357651	0.491629145	down	0.019983087	0.048420842
G10090_31928	67087	Ctnnbip1	-1.024991552	0.491413177	down	1.29544E-07	1.0626E-06
G10090_15813	67177	Cdt1	-1.025888108	0.491107885	down	5.96512E-05	0.000292112
G10090_21456	193740	Hspa1a	-1.026555105	0.490880885	down	0.000505791	0.001961215
G10090_7319	12523	Cd84	-1.027354417	0.490608993	down	1.77577E-10	2.29429E-09
G10090_9122	20454	St3gal5	-1.027995234	0.490391122	down	0.000313854	0.00127482
G10090_23902	69534	Avpi1	-1.028668196	0.490162427	down	0.001353615	0.004651384
G10090_26142	73739	Cby1	-1.031302038	0.489268383	down	1.65093E-05	9.16989E-05
G10090_28762	230787	Themis2	-1.031834365	0.489087885	down	0.005380105	0.015656549
G10090_15357	399566	Btbd6	-1.032696667	0.488795644	down	0.000245583	0.001029498
G10090_8725	68053	Ubxn2b	-1.033959008	0.488368141	down	1.351E-08	1.31041E-07
G10090_24153	101835	AW146154	-1.034594747	0.488152983	down	0.002464148	0.00789675
G10090_18393	54678	Zfp108	-1.035983704	0.487683239	down	0.009639735	0.025870185
G10090_2962	19687	Rfc1	-1.036541277	0.487494796	down	5.87844E-12	9.35044E-11
G10090_966	319939	Tns3	-1.038045569	0.486986752	down	2.89026E-07	2.21773E-06
G10090_26507	100038947	LOC100038947	-1.03884993	0.486715313	down	5.9462E-19	2.06224E-17
G10090_24352	80837	Rhoj	-1.039231019	0.486586764	down	0.001750522	0.005837304
G10090_3286	68801	Elovl5	-1.0395607	0.486475583	down	2.51146E-12	4.21532E-11
G10090_10863	17245	Mdm1	-1.039748926	0.486412117	down	0.000201318	0.00086077
G10090_5665	228357	Lrp4	-1.040220783	0.486253054	down	0.00112455	0.003947246
G10090_883	21886	Tle2	-1.041537307	0.485809529	down	0.006144742	0.017534374
G10090_1258	226525	Rasal2	-1.042291797	0.48555553	down	0.000214785	0.000912381
G10090_10345	76779	Cluap1	-1.042938192	0.485338027	down	0.000209334	0.000891254
G10090_7274	171167	Fut10	-1.043494099	0.48515105	down	0.010593519	0.028010163
G10090_29409	11555	Adrb2	-1.043697703	0.485082587	down	0.000293595	0.001204505
G10090_28934	67486	Polr3g	-1.045555708	0.484458265	down	3.38952E-05	0.000174274
G10090_14421	69274	Ctdspl	-1.045733719	0.484398493	down	3.70497E-05	0.000189081
G10090_8395	98314	D2hgdh	-1.046211757	0.484238014	down	1.39113E-11	2.115E-10
G10090_28668	216188	Aldh1l2	-1.046650212	0.484090869	down	0.005209199	0.015206582
G10090_11526	100017	Ldlrap1	-1.048320277	0.48353081	down	1.97577E-05	0.000107236
G10090_685	76044	Ncapg2	-1.04869617	0.483404842	down	0.000135492	0.000608365
G10090_19645	22340	Vegfb	-1.048843595	0.483355447	down	0.000120645	0.000549627
G10090_14489	231003	Klhl17	-1.049171724	0.483245524	down	1.17914E-05	6.77084E-05
G10090_8659	70380	Mospd1	-1.049364306	0.483181021	down	1.65152E-06	1.1085E-05
G10090_16178	75764	Slx1b	-1.050169164	0.482911537	down	7.46152E-07	5.32986E-06
G10090_8771	20322	Sord	-1.05057276	0.482776461	down	4.72508E-09	4.98631E-08
G10090_5175	18710	Pik3r3	-1.051198924	0.48256697	down	0.001513155	0.005129505
G10090_7108	22129	Ttc3	-1.051494705	0.482468044	down	2.37844E-08	2.20867E-07

G10090_15819	83669	Wdr6	-1.051999043	0.482299412	down	4.79003E-11	6.74888E-10
G10090_5594	20744	Strbp	-1.05237786	0.482172789	down	1.82753E-05	9.98953E-05
G10090_8110	68021	Bphl	-1.053148447	0.481915314	down	1.364E-06	9.29355E-06
G10090_5478	16438	Itpr1	-1.053373184	0.481840249	down	6.70943E-06	4.03208E-05
G10090_5376	73827	Tmem198b	-1.053566704	0.481775621	down	0.000101733	0.000473701
G10090_386	242083	Ppm1l	-1.053697017	0.481732106	down	0.000814617	0.002981001
G10090_4919	72169	Trim29	-1.053732379	0.481720298	down	0.000797189	0.002924041
G10090_6874	70435	Inf2	-1.054204784	0.481562587	down	8.66586E-11	1.17537E-09
G10090_10905	27801	Zdhhc8	-1.054219535	0.481557663	down	4.53863E-11	6.40848E-10
G10090_5418	68964	Ctc1	-1.055782758	0.481036157	down	0.011597865	0.030364854
G10090_21107	12368	Casp6	-1.057277497	0.480538026	down	1.79321E-07	1.43314E-06
G10090_14675	319604	Fam168a	-1.057470783	0.48047365	down	1.44712E-15	3.66687E-14
G10090_11792	107702	Rnh1	-1.058597855	0.480098437	down	1.2835E-14	2.86869E-13
G10090_2645	74166	Tmem38a	-1.058614786	0.480092803	down	0.000537393	0.002070245
G10090_1089	319518	Pdpr	-1.059206553	0.479895918	down	6.76554E-18	2.11625E-16
G10090_6295	76967	2700049A03Rik	-1.061730814	0.479056986	down	0.000119485	0.000544913
G10090_15146	60455	Rxylt1	-1.062743659	0.478720782	down	7.16273E-11	9.85817E-10
G10090_12800	80288	Bcl9l	-1.063495768	0.478471279	down	2.96359E-06	1.90506E-05
G10090_13204	22700	Zfp40	-1.064489689	0.478141758	down	0.008794262	0.023890501
G10090_556	56526	Sept6	-1.064626744	0.478096337	down	0.002122493	0.0069137
G10090_30512	16971	Lrp1	-1.06562964	0.477764101	down	1.52121E-13	3.00904E-12
G10090_8905	101706	Numa1	-1.067218603	0.477238189	down	7.93402E-16	2.0706E-14
G10090_27891	219022	Ttc5	-1.06734226	0.477197285	down	2.02359E-08	1.89939E-07
G10090_253	20541	Slc8a1	-1.067399874	0.477178228	down	1.39161E-07	1.1365E-06
G10090_26472	208647	Creb3l2	-1.067414289	0.477173461	down	0.00059653	0.002269569
G10090_5367	105171	Arrdc3	-1.068905885	0.476680368	down	1.68124E-05	9.30663E-05
G10090_7469	56743	Lat2	-1.069104855	0.476614631	down	1.94439E-08	1.83162E-07
G10090_1531	22249	Unc13b	-1.070430571	0.476176863	down	0.016362953	0.040829314
G10090_24302	269209	Stk36	-1.071883906	0.475697416	down	0.000291329	0.00119596
G10090_11040	216848	Chd3	-1.071930221	0.475682145	down	5.91388E-09	6.12224E-08
G10090_2702	71729	Rgs12	-1.072340394	0.475546923	down	4.40491E-06	2.73477E-05
G10090_15709	108168101	Gm46430	-1.076096464	0.474310442	down	2.73107E-06	1.76951E-05
G10090_11948	208922	Cpeb3	-1.076943639	0.474032001	down	2.38115E-05	0.000126993
G10090_7101	23805	Apc2	-1.078032188	0.473674467	down	0.002287869	0.007402595
G10090_3027	51789	Tnk2	-1.078091427	0.473655018	down	0.003861559	0.011736273
G10090_3474	108012	Ap1s2	-1.078830249	0.473412515	down	4.18912E-11	5.94065E-10
G10090_30139	19718	Rfc2	-1.078853885	0.473404759	down	2.74554E-06	1.77801E-05
G10090_13357	243834	Zfp324	-1.079278685	0.473265386	down	3.00498E-07	2.29633E-06
G10090_7838	232539	Klhl42	-1.080708106	0.472796708	down	0.00074826	0.002766044
G10090_13427	18709	Pik3r2	-1.080791678	0.47276932	down	8.34289E-12	1.30326E-10
G10090_16345	619331	Zfp551	-1.082094273	0.472342654	down	0.011949013	0.031146999
G10090_11154	16332	Inppl1	-1.083070322	0.472023201	down	8.75636E-10	1.0361E-08
G10090_4915	214580	Pstk	-1.084335009	0.4716096	down	0.002633647	0.008372217
G10090_1771	268970	Arhgap28	-1.084553287	0.471538251	down	0.009224133	0.024903065
G10090_19622	67911	Zfp169	-1.085798085	0.47113157	down	1.40816E-07	1.14786E-06
G10090_28937	68943	Pink1	-1.086312804	0.470963512	down	8.69264E-16	2.25062E-14
G10090_5781	217893	Pacs2	-1.08645387	0.470917464	down	5.62727E-30	4.71644E-28
G10090_17144	67397	Erp29	-1.086724518	0.470829128	down	2.62835E-17	7.82817E-16
G10090_1366	75216	Cep128	-1.088440357	0.470269491	down	0.00854979	0.023313556
G10090_10617	245622	Fam199x	-1.089320161	0.469982792	down	1.06495E-10	1.42229E-09
G10090_24666	16440	Itpr3	-1.089528359	0.469914973	down	3.76406E-08	3.39415E-07
G10090_1250	216445	Arhgap9	-1.090343754	0.469649457	down	5.84767E-14	1.21555E-12
G10090_16826	21366	Slc6a6	-1.091982803	0.46911619	down	2.87477E-36	3.546E-34
G10090_1392	18016	Nf2	-1.092095515	0.469079541	down	4.46844E-23	2.27334E-21
G10090_18647	234730	Fuk	-1.092432827	0.46896988	down	0.000923466	0.003329011
G10090_17092	103140	Gstt3	-1.093619954	0.468584145	down	2.00246E-09	2.23899E-08
G10090_25876	52163	Camk1	-1.094710366	0.468230116	down	9.70765E-06	5.68162E-05
G10090_5173	50708	Hist1h1c	-1.097038344	0.467475174	down	0.013733508	0.035133167
G10090_27815	110173	Manba	-1.097799065	0.467228743	down	1.00207E-08	9.95599E-08
G10090_26310	268670	Zfp759	-1.097894332	0.467197891	down	0.00018549	0.000800689
G10090_18477	21812	Tgfbr1	-1.098484402	0.467006844	down	1.61219E-12	2.78092E-11
G10090_31101	233210	Prr12	-1.099541433	0.466664804	down	5.9889E-09	6.18521E-08
G10090_4677	225651	Mppe1	-1.101283955	0.466101495	down	9.47817E-07	6.6771E-06

G10090_29904	11545	Parp1	-1.10132955	0.466086764	down	1.66988E-07	1.34196E-06
G10090_743	637515	Nlrp1b	-1.102357856	0.465754671	down	2.29227E-05	0.000122541
G10090_13958	241576	Ldlrad3	-1.103129069	0.465505762	down	1.36328E-07	1.11545E-06
G10090_8138	229615	Pias3	-1.10373388	0.465310652	down	2.16653E-10	2.77176E-09
G10090_4593	230594	Tut4	-1.104290703	0.465131096	down	6.4797E-09	6.65008E-08
G10090_9336	217935	Wdr60	-1.104315499	0.465123101	down	1.03226E-05	5.99592E-05
G10090_5764	328232	Gfod1	-1.104379729	0.465102394	down	0.002685229	0.008507236
G10090_20959	66443	Tnfaip8l1	-1.105321741	0.464798803	down	0.000286979	0.001179952
G10090_13005	333433	Gpd1l	-1.106471351	0.464428576	down	2.63126E-05	0.000138781
G10090_13571	666938	Bend4	-1.106750531	0.464338712	down	4.89268E-07	3.61014E-06
G10090_10872	66939	Aagab	-1.10837079	0.463817516	down	2.3893E-18	7.77116E-17
G10090_3040	16001	Igf1r	-1.109365292	0.4634979	down	1.99546E-07	1.58149E-06
G10090_2267	231866	Zfp12	-1.109612139	0.463418602	down	2.14842E-09	2.37854E-08
G10090_3767	270035	Letm2	-1.1102376	0.463217736	down	0.006858185	0.019304795
G10090_32204	15511	Hspa1b	-1.110504136	0.463132166	down	7.97334E-10	9.50332E-09
G10090_21203	105246961	AB010352	-1.111037686	0.462960918	down	4.42789E-06	2.74643E-05
G10090_5882	19090	Prkdc	-1.113202286	0.462266818	down	8.39936E-11	1.14278E-09
G10090_25406	11565	Adssl1	-1.113759961	0.462088163	down	5.95302E-07	4.33143E-06
G10090_28749	110078	Pygb	-1.114016555	0.462005985	down	1.0459E-14	2.35736E-13
G10090_8884	24100	Tpra1	-1.116466685	0.461222026	down	2.0757E-09	2.3078E-08
G10090_29735	619441	Tnfsfm13	-1.120523704	0.45992684	down	7.12864E-07	5.10993E-06
G10090_3861	16554	Kif13b	-1.121452618	0.4596308	down	3.91382E-14	8.29388E-13
G10090_13276	69876	Thap3	-1.124102611	0.458787309	down	0.00027821	0.001150321
G10090_31603	26384	Gnpda1	-1.124260335	0.458737154	down	1.00071E-08	9.95002E-08
G10090_20326	51791	Rgs14	-1.124689153	0.458600822	down	2.23412E-19	8.15952E-18
G10090_2577	212503	Paox	-1.124905274	0.458532127	down	0.000545104	0.002097479
G10090_20972	76491	Abhd14b	-1.126520714	0.458018979	down	0.007476257	0.020785044
G10090_21400	13034	Ctse	-1.127304138	0.457770329	down	1.38926E-05	7.85328E-05
G10090_29851	382019	Zfp882	-1.127539467	0.457695665	down	0.003936668	0.011925841
G10090_4347	27387	Sh2d3c	-1.128131356	0.457507927	down	1.23461E-06	8.48712E-06
G10090_15502	73095	Slc25a42	-1.128398081	0.457423351	down	0.000669757	0.002514955
G10090_18370	231464	Cnot6l	-1.129145912	0.457186303	down	9.31911E-13	1.66717E-11
G10090_12702	68201	Ccdc34	-1.12935034	0.457121525	down	0.000723649	0.002690279
G10090_10216	69596	Ap5s1	-1.13146465	0.456452092	down	7.86722E-05	0.000375279
G10090_31909	72345	Amer1	-1.131860679	0.45632681	down	1.1665E-06	8.03582E-06
G10090_31595	17688	Msh6	-1.133372434	0.455848889	down	3.66818E-06	2.30917E-05
G10090_5297	228730	Kiz	-1.133475191	0.455816423	down	1.25067E-07	1.0304E-06
G10090_11922	320487	Heatr5a	-1.133614493	0.455772413	down	2.00353E-09	2.23899E-08
G10090_8714	75590	Dusp9	-1.134364193	0.455535631	down	0.017113692	0.042435335
G10090_14735	29813	Zfp385a	-1.137174521	0.454649124	down	5.09984E-07	3.75241E-06
G10090_6392	77605	H2afv	-1.137312705	0.454605579	down	0.000102296	0.000476155
G10090_6415	73422	Prox2	-1.138078887	0.454364213	down	0.004166538	0.012509182
G10090_14595	195046	Nlrp1a	-1.13918968	0.454014513	down	0.00155866	0.005263538
G10090_19854	74653	Pomk	-1.142993141	0.452819144	down	9.19373E-07	6.49071E-06
G10090_8010	12628	Cfh	-1.143378148	0.452698318	down	0.001103896	0.003888319
G10090_16473	18018	Nfatc1	-1.143509395	0.452657136	down	1.08137E-10	1.44274E-09
G10090_11596	63913	Fam129a	-1.143605271	0.452627056	down	0.000858093	0.003119147
G10090_13700	71904	Paqr7	-1.143884536	0.452539448	down	3.89335E-10	4.80695E-09
G10090_4948	22775	Zik1	-1.144239015	0.45242827	down	0.004464255	0.013287079
G10090_7281	235050	Zfp810	-1.145256504	0.452109299	down	0.001386393	0.004750285
G10090_17752	20598	Smpd2	-1.145768036	0.451949024	down	3.58028E-07	2.69811E-06
G10090_11457	236920	Stard8	-1.147400411	0.451437944	down	1.7192E-20	6.98765E-19
G10090_5778	76088	Dock8	-1.149380363	0.450818816	down	7.41369E-17	2.13042E-15
G10090_14599	320832	Sirpb1a	-1.151420312	0.450181816	down	0.000108335	0.000499463
G10090_30927	18590	Pdgfa	-1.152475677	0.449852618	down	0.009890587	0.026435604
G10090_23847	231842	Amz1	-1.152615461	0.449809033	down	2.12137E-05	0.000114379
G10090_4006	64009	Syne1	-1.153317024	0.44959035	down	0.00042514	0.001676832
G10090_11049	216190	Appl2	-1.153838601	0.449427839	down	7.75611E-11	1.0619E-09
G10090_3856	72194	Fbxl20	-1.156057654	0.448737092	down	7.27124E-15	1.68268E-13
G10090_9203	72140	Cep89	-1.15733345	0.448340443	down	0.000812266	0.002974895
G10090_18001	269401	Zfp512b	-1.157373637	0.448327954	down	4.46145E-13	8.29779E-12
G10090_1840	66355	Gmpr	-1.15750951	0.448285733	down	0.000124465	0.000564277
G10090_12799	78339	Ttyh3	-1.157524626	0.448281036	down	1.03289E-14	2.33249E-13

G10090_17496	13849	Ephx1	-1.157814268	0.448191046	down	4.825E-07	3.56423E-06
G10090_12377	15926	Idh1	-1.159717804	0.447600079	down	4.76931E-11	6.72694E-10
G10090_2482	212514	Spice1	-1.160472913	0.447365866	down	5.81643E-08	5.05987E-07
G10090_9550	18768	Pkib	-1.162371703	0.446777456	down	0.014490842	0.036754173
G10090_8871	233826	Palb2	-1.163329969	0.446480797	down	0.007339414	0.020465522
G10090_6562	106393	Srl	-1.16413761	0.44623092	down	0.017482716	0.043227404
G10090_11599	70122	Mllt3	-1.164236487	0.446200339	down	3.9199E-06	2.4476E-05
G10090_11431	244668	Sipa1l2	-1.16488323	0.446000357	down	3.46664E-05	0.00017775
G10090_6266	228543	Rhov	-1.165639918	0.445766493	down	8.72735E-06	5.14937E-05
G10090_8123	19009	Pou6f1	-1.167125033	0.445307855	down	2.05523E-09	2.28894E-08
G10090_7091	77300	Raph1	-1.167155487	0.445298456	down	2.36026E-16	6.41187E-15
G10090_457	75564	Rsph9	-1.168693794	0.444823899	down	0.000493996	0.001920606
G10090_760	668101	Sirpb1b	-1.168895217	0.444761799	down	7.64813E-05	0.000366298
G10090_32317	75219	Dusp18	-1.17235524	0.443696402	down	6.61468E-14	1.36846E-12
G10090_11273	22719	Zfp61	-1.173984553	0.443195595	down	3.35024E-08	3.03775E-07
G10090_13368	17979	Ncoa3	-1.174173015	0.443137703	down	3.99058E-12	6.5221E-11
G10090_8463	223701	Mkl1	-1.176088517	0.442549729	down	1.95049E-10	2.51011E-09
G10090_6937	66970	Ssbp2	-1.176255524	0.442498502	down	9.17008E-06	5.38871E-05
G10090_9623	213081	Wdr19	-1.177049781	0.442254957	down	9.50654E-11	1.28274E-09
G10090_14649	232201	Arhgap25	-1.177081225	0.442245318	down	6.75766E-07	4.86544E-06
G10090_23754	11858	Rnd2	-1.17759695	0.442087256	down	0.000943734	0.003394585
G10090_18619	18642	Pfkm	-1.177948445	0.44197956	down	2.1623E-06	1.42428E-05
G10090_20807	107227	Macrod1	-1.178520581	0.441804316	down	0.000154367	0.000682567
G10090_6051	381413	Gpr176	-1.178791917	0.441721231	down	0.012269279	0.031911839
G10090_10592	16195	Il6st	-1.180590827	0.441170789	down	1.15743E-15	2.93853E-14
G10090_7900	233064	Wdr62	-1.180886234	0.441080463	down	2.87973E-05	0.000150429
G10090_27473	108671	Dnajc9	-1.180897555	0.441077002	down	0.001879464	0.00621183
G10090_22757	16600	Klf4	-1.181876876	0.440777695	down	5.68154E-05	0.000279586
G10090_17820	80880	Kank3	-1.182060089	0.440721722	down	0.000606724	0.002299396
G10090_3881	11733	Ank1	-1.183093097	0.440406267	down	0.001181358	0.0041212
G10090_26157	67041	Oxct1	-1.183443697	0.440299253	down	9.77928E-15	2.22372E-13
G10090_1593	26407	Map3k4	-1.184215251	0.440063844	down	2.42826E-10	3.08547E-09
G10090_12720	67881	Mdp1	-1.184268679	0.440047547	down	8.39232E-07	5.96357E-06
G10090_2296	56233	Hdac7	-1.184809764	0.439882538	down	1.24738E-07	1.02889E-06
G10090_11904	209039	Tns2	-1.185154029	0.439777583	down	0.008314437	0.022752463
G10090_19073	22761	Zfpm1	-1.185213445	0.439759471	down	1.28594E-05	7.33899E-05
G10090_544	107250	Kazald1	-1.185242145	0.439750723	down	0.007095121	0.019869074
G10090_17366	230815	Man1c1	-1.186441156	0.439385402	down	1.06487E-18	3.58845E-17
G10090_13396	16010	Igfbp4	-1.187822807	0.438964809	down	7.69548E-09	7.79987E-08
G10090_2548	329470	Accs	-1.188743569	0.438684741	down	2.66597E-05	0.000140442
G10090_5703	11856	Arhgap6	-1.188909355	0.438634333	down	2.32164E-09	2.55948E-08
G10090_9249	243897	Ggn	-1.190079103	0.438278829	down	0.006189467	0.017642746
G10090_12324	68968	Cdan1	-1.190905184	0.438027944	down	4.55752E-12	7.36209E-11
G10090_32719	217653	Mis18bp1	-1.193240031	0.437319616	down	0.00313232	0.00974425
G10090_22629	12367	Casp3	-1.196486213	0.436336718	down	1.63097E-15	4.11679E-14
G10090_2504	68346	Sirt5	-1.196931498	0.436202064	down	0.000144702	0.000643529
G10090_407	14256	Flt3l	-1.197285227	0.436095127	down	0.000568509	0.002180481
G10090_961	56758	Mbnl1	-1.197338549	0.436079009	down	5.03269E-12	8.08383E-11
G10090_7301	232560	Caprin2	-1.199267943	0.435496206	down	0.005969852	0.017117504
G10090_9021	69519	Rwdd2a	-1.201690775	0.434765457	down	0.000731569	0.00271355
G10090_1885	269023	Zfp608	-1.202586062	0.43449574	down	0.000172126	0.000749183
G10090_19431	230101	Gba2	-1.204807585	0.4338272	down	1.22032E-13	2.44719E-12
G10090_29266	331487	Uprt	-1.205309553	0.433676282	down	4.21532E-05	0.000212965
G10090_8533	140570	Plxnb2	-1.206170089	0.43341768	down	5.86701E-32	5.47937E-30
G10090_8270	71276	Ccdc57	-1.207050587	0.43315324	down	0.000120371	0.00054857
G10090_450	269180	Inpp4a	-1.208647027	0.432674191	down	5.90065E-14	1.22462E-12
G10090_13795	15394	Hoxa1	-1.208879691	0.432604419	down	0.011851491	0.030942143
G10090_5574	54354	Rassf5	-1.209105503	0.432536713	down	2.33928E-12	3.9466E-11
G10090_18318	212377	Mms22l	-1.209582508	0.432393725	down	0.007733276	0.021422157
G10090_21243	67370	Zfp606	-1.210723169	0.43205199	down	2.84481E-06	1.83502E-05
G10090_12438	13433	Dnmt1	-1.211114944	0.431934679	down	7.83061E-06	4.64965E-05
G10090_15609	229521	Syt11	-1.212150113	0.431624866	down	0.000708948	0.002643141
G10090_12125	81896	Ift122	-1.212260253	0.431591916	down	1.54459E-07	1.2505E-06

G10090_1332	215615	Rnpep	-1.212559728	0.431502335	down	8.41934E-11	1.14431E-09
G10090_5386	382018	Unc13a	-1.213707414	0.431159205	down	0.004648704	0.013770234
G10090_1999	72750	Fam117b	-1.213762838	0.431142641	down	1.15314E-19	4.32013E-18
G10090_4921	14886	Gtf2i	-1.215452511	0.430637986	down	1.00753E-27	7.27815E-26
G10090_9864	22643	Zfp101	-1.218336677	0.429777936	down	3.46086E-05	0.000177523
G10090_5356	107321	Lpxn	-1.21844434	0.429745864	down	2.98138E-15	7.28628E-14
G10090_4091	18640	Pfkfb2	-1.218715897	0.429664981	down	2.32084E-12	3.92055E-11
G10090_5759	17101	Lyst	-1.21930863	0.429488489	down	7.57658E-11	1.03949E-09
G10090_23571	217666	L2hgdh	-1.220184185	0.429227916	down	3.65141E-05	0.000186566
G10090_20480	14697	Gnb5	-1.221149859	0.428940707	down	6.65117E-07	4.79405E-06
G10090_12587	11486	Ada	-1.221351885	0.428880645	down	1.28206E-06	8.77642E-06
G10090_20049	620913	Gm12185	-1.221641421	0.428794581	down	0.015554633	0.039082136
G10090_32055	108902	B4gat1	-1.226417565	0.427377374	down	6.83986E-09	6.99775E-08
G10090_12379	235584	Dusp7	-1.227110652	0.427172106	down	0.000135972	0.000609642
G10090_13894	320404	Itpkb	-1.227750581	0.426982669	down	1.72149E-07	1.38004E-06
G10090_4250	68145	Etaa1	-1.227968101	0.426918296	down	3.08461E-05	0.000160236
G10090_20235	216846	Cntrob	-1.229304082	0.42652314	down	0.000119409	0.000544758
G10090_26430	19340	Rab3d	-1.230302773	0.426227986	down	6.95207E-07	4.99716E-06
G10090_10314	17127	Smad3	-1.23092038	0.42604556	down	0.000463983	0.001814709
G10090_14094	76612	Lrrc27	-1.232702695	0.425519545	down	1.49657E-05	8.39455E-05
G10090_1630	233899	Ccdc189	-1.235116821	0.4248081	down	0.009313603	0.025118681
G10090_1182	269604	Gpr157	-1.235614102	0.424661698	down	0.000326385	0.001320387
G10090_14339	53310	Dlg3	-1.235664918	0.424646741	down	0.004452462	0.013261036
G10090_4701	67260	Cers4	-1.236164921	0.424499594	down	0.013522641	0.03466834
G10090_12566	104759	Pld4	-1.236826284	0.424305039	down	0.000704718	0.002631122
G10090_11050	101148	Bmt2	-1.237538955	0.42409549	down	6.24886E-05	0.000304979
G10090_24864	19221	Ptgfrn	-1.238111678	0.423927165	down	0.007877862	0.021776541
G10090_9888	208111	Zfp976	-1.238353809	0.423856022	down	0.000265247	0.001103437
G10090_3621	170770	Bbc3	-1.239789477	0.423434441	down	2.08104E-06	1.37631E-05
G10090_17211	67865	Rgs10	-1.240138772	0.423331934	down	4.8559E-13	9.00234E-12
G10090_28002	104082	Wdr7	-1.240895584	0.42310992	down	3.65181E-07	2.74726E-06
G10090_8619	19141	Lgmn	-1.243270921	0.42241386	down	5.59156E-06	3.41794E-05
G10090_29657	226970	Arhgef4	-1.243796449	0.422260016	down	2.60381E-05	0.000137499
G10090_2715	544696	Tbc1d32	-1.246297042	0.421528756	down	1.4927E-08	1.43192E-07
G10090_29906	12399	Runx3	-1.246500039	0.421469448	down	0.01579444	0.039561743
G10090_32210	319190	Hist2h2be	-1.247087032	0.421297999	down	0.001473934	0.005016056
G10090_16520	21372	Tbl1x	-1.249549137	0.420579624	down	1.0369E-11	1.59876E-10
G10090_11998	228662	Btbd3	-1.250279288	0.420366822	down	0.000280712	0.001158556
G10090_27812	12846	Comt	-1.250663642	0.420254845	down	2.15271E-26	1.37974E-24
G10090_5141	21944	Tnfsf12	-1.252054558	0.419849869	down	2.11081E-05	0.000113857
G10090_225	100041734	4930522L14Rik	-1.252733996	0.419652187	down	0.000193407	0.000831295
G10090_22987	71446	Wrb	-1.253561251	0.419411623	down	1.40047E-10	1.84032E-09
G10090_32594	218442	Serinc5	-1.256528363	0.418549929	down	4.24238E-12	6.89914E-11
G10090_2235	381605	Tbc1d2	-1.257317909	0.418320931	down	0.016945391	0.042073869
G10090_1316	56338	Txnip	-1.257540746	0.418256323	down	6.3305E-21	2.67868E-19
G10090_19657	16973	Lrp5	-1.258050573	0.418108543	down	1.56285E-06	1.05223E-05
G10090_26500	240334	Pcyox1l	-1.258445799	0.417994018	down	0.001879261	0.00621183
G10090_11407	20975	Synj2	-1.259406042	0.417715898	down	6.09265E-10	7.36242E-09
G10090_19417	13435	Dnmt3a	-1.259921049	0.41756681	down	4.40656E-07	3.28108E-06
G10090_2127	66049	Rogdi	-1.260475107	0.417406477	down	3.55595E-07	2.68132E-06
G10090_21466	69226	Snx24	-1.261157531	0.417209082	down	3.49343E-14	7.50026E-13
G10090_17720	18971	Pold1	-1.261596067	0.417082282	down	0.002747547	0.008675241
G10090_27793	214345	Lrrc1	-1.26283798	0.416723401	down	0.008893955	0.024126236
G10090_20371	333193	Proser3	-1.262944005	0.416692776	down	1.52154E-06	1.02653E-05
G10090_3010	194126	Mtmr11	-1.264263056	0.41631197	down	4.30841E-11	6.09659E-10
G10090_5732	72128	2610008E11Rik	-1.26429839	0.416301774	down	9.79802E-07	6.88019E-06
G10090_20206	18718	Pip4k2a	-1.264364004	0.41628284	down	2.94902E-20	1.18641E-18
G10090_4776	110639	Prps2	-1.264754123	0.416170289	down	0.00010907	0.00050232
G10090_7499	29877	Hdgfl3	-1.264946966	0.416114664	down	5.38468E-06	3.30228E-05
G10090_1644	19726	Rfx3	-1.269826786	0.414709561	down	0.001115561	0.003920957
G10090_18165	12580	Cdkn2c	-1.270239373	0.414590978	down	0.002376838	0.007656359
G10090_51	11513	Adcy7	-1.270645007	0.414474426	down	9.67082E-26	5.9927E-24
G10090_29440	67661	Ift172	-1.27126589	0.41429609	down	7.7582E-10	9.26378E-09

G10090_16259	140792	Colec12	-1.271655545	0.414184209	down	4.21002E-37	5.5046E-35
G10090_10911	109242	Kif24	-1.27209183	0.414058974	down	0.008583178	0.023386024
G10090_22251	22436	Xdh	-1.272108711	0.414054129	down	4.7856E-18	1.51873E-16
G10090_33703	100637	N4bp2l1	-1.273157654	0.413753192	down	3.43496E-11	4.9354E-10
G10090_7999	99151	Cercam	-1.274483876	0.413373017	down	1.48327E-07	1.20383E-06
G10090_30048	192289	Tmlhe	-1.274770449	0.413290914	down	3.92481E-11	5.60227E-10
G10090_6688	77318	Ankrd55	-1.276252206	0.41286665	down	0.000748147	0.002766044
G10090_17725	14132	Fcgrt	-1.276463534	0.412806178	down	3.13481E-07	2.383E-06
G10090_2223	52468	Ctdsp2	-1.277455506	0.412522437	down	2.21516E-19	8.11294E-18
G10090_1261	242584	Wdr78	-1.27811764	0.41233315	down	0.002461448	0.007890029
G10090_8679	219144	Arl11	-1.279339145	0.411984183	down	1.89051E-05	0.000103037
G10090_6798	12815	Col11a2	-1.279672262	0.411889067	down	0.004136099	0.012435291
G10090_4796	19724	Rfx1	-1.280922834	0.411532184	down	1.78819E-18	5.85978E-17
G10090_20358	215707	Ccdc92	-1.281893537	0.411255382	down	0.001528245	0.005169936
G10090_14215	214359	Tmem51	-1.282598883	0.411054364	down	5.29007E-11	7.41347E-10
G10090_19381	338364	Trim65	-1.28403129	0.410646444	down	1.50665E-09	1.713E-08
G10090_3029	74552	Nipal3	-1.284887772	0.410402729	down	1.36472E-17	4.13049E-16
G10090_13429	12193	Zfp36l2	-1.285250222	0.410299636	down	2.64298E-09	2.8918E-08
G10090_5922	109689	Arrb1	-1.28570444	0.410170477	down	1.36449E-29	1.12206E-27
G10090_7051	76117	Arhgap15	-1.287683396	0.409608229	down	2.48955E-10	3.1511E-09
G10090_18427	218973	Wdhd1	-1.287721183	0.409597501	down	0.020438782	0.049369495
G10090_15120	68552	Smim14	-1.291700773	0.408469207	down	2.82677E-19	1.02341E-17
G10090_12150	75939	4930579G24Rik	-1.291938024	0.408402039	down	0.012545217	0.032519569
G10090_21922	98952	Fam102a	-1.292053246	0.408369423	down	3.94884E-10	4.86627E-09
G10090_2314	76051	Ganc	-1.293236699	0.408034572	down	1.15515E-08	1.13305E-07
G10090_19894	219072	Haus4	-1.294007258	0.407816695	down	0.005631435	0.016268451
G10090_2122	235633	Als2cl	-1.295994131	0.407255438	down	1.60507E-05	8.94556E-05
G10090_32512	17084	Ly86	-1.296978507	0.406977656	down	0.002768077	0.008731629
G10090_2346	241950	Bbs12	-1.297529656	0.406822209	down	0.001319201	0.004549867
G10090_5144	12286	Cacna1a	-1.29763044	0.40679379	down	1.43484E-20	5.91815E-19
G10090_30097	56078	Car5b	-1.298556588	0.406532729	down	4.17228E-09	4.45327E-08
G10090_7539	56364	Zmym3	-1.300513007	0.40598181	down	1.21212E-10	1.6041E-09
G10090_4997	69890	Zfp219	-1.300853051	0.405886131	down	1.95076E-16	5.35844E-15
G10090_31967	22057	Tob1	-1.301169405	0.405797138	down	1.75093E-10	2.26443E-09
G10090_4523	110094	Phka2	-1.301473071	0.405711733	down	5.07372E-11	7.12555E-10
G10090_19314	170826	Ppargc1b	-1.304550038	0.404847356	down	0.010911564	0.02876965
G10090_22084	68659	Fam198b	-1.305807313	0.404494695	down	0.000421878	0.001666986
G10090_9440	17686	Msh3	-1.30625691	0.404368659	down	8.2921E-15	1.90879E-13
G10090_9219	77827	Krba1	-1.310517191	0.403176319	down	0.00858524	0.023386024
G10090_6423	56016	Hebp2	-1.3128753	0.402517859	down	0.017677718	0.043618827
G10090_6846	101490	Inpp5f	-1.313080183	0.4024607	down	5.89503E-19	2.04993E-17
G10090_10108	15107	Hadh	-1.313680687	0.402293215	down	2.72553E-14	5.91965E-13
G10090_28944	217473	Ankmy2	-1.315611921	0.401755054	down	3.19302E-10	3.98366E-09
G10090_7793	76306	Slc18b1	-1.315995145	0.40164835	down	0.01425307	0.036235444
G10090_21587	69528	1700030J22Rik	-1.316154185	0.401604075	down	0.001149957	0.004027775
G10090_2077	15893	Ica1	-1.317947452	0.401105193	down	0.004886584	0.014380394
G10090_14472	235627	Nbeal2	-1.321242802	0.400190049	down	3.07273E-07	2.33717E-06
G10090_1922	239546	Zfp647	-1.322926261	0.399723345	down	0.00292233	0.00916514
G10090_1667	228355	Madd	-1.324618935	0.399254636	down	3.07109E-09	3.33509E-08
G10090_9198	11630	Crybg1	-1.324816089	0.399200079	down	4.42327E-14	9.2981E-13
G10090_10867	64214	Rgs18	-1.326377578	0.398768242	down	0.002354651	0.007596118
G10090_11585	231801	Agfg2	-1.326588347	0.398709988	down	7.97715E-20	3.03201E-18
G10090_6118	69399	1700025G04Rik	-1.327208139	0.398538737	down	3.60101E-05	0.000184351
G10090_5504	71279	Slc29a3	-1.329197051	0.397989686	down	1.27444E-13	2.54401E-12
G10090_25812	76454	Fbxo31	-1.330626259	0.397595612	down	1.13666E-07	9.4361E-07
G10090_3678	263764	Creg2	-1.332843161	0.39698512	down	0.001092729	0.003855217
G10090_1150	17207	Mcf2l	-1.33370248	0.396748733	down	0.00186272	0.00616428
G10090_6866	22678	Zfp2	-1.333839732	0.396710989	down	0.004052318	0.012247817
G10090_6649	68910	Zfp467	-1.335818167	0.396167334	down	0.000128461	0.000579983
G10090_8798	80987	Nckipsd	-1.336369131	0.396016067	down	2.52248E-15	6.24647E-14
G10090_18608	74551	Pck2	-1.336413483	0.396003893	down	1.78293E-05	9.80311E-05
G10090_2468	70652	Tmem144	-1.337518886	0.395700588	down	0.000155751	0.00068822
G10090_10445	57441	Gmnn	-1.338163098	0.395523934	down	0.00230567	0.007456502

G10090_20171	54725	Cadm1	-1.340457615	0.394895377	down	4.56419E-08	4.04314E-07
G10090_1132	71583	9130008F23Rik	-1.340776041	0.394808227	down	0.005002397	0.01467497
G10090_6143	232227	Iqsec1	-1.340881973	0.394779239	down	1.65261E-07	1.33054E-06
G10090_12503	320271	Scai	-1.341051116	0.394732957	down	4.462E-07	3.31858E-06
G10090_13528	11920	Atm	-1.342050038	0.394459739	down	2.74732E-12	4.57596E-11
G10090_5385	102124	Enkd1	-1.342278144	0.394397375	down	0.000138535	0.00062011
G10090_11764	16950	Loxl3	-1.343432929	0.394081812	down	5.32417E-08	4.66892E-07
G10090_14791	433904	Ociad2	-1.345817201	0.39343107	down	0.004084206	0.012324871
G10090_3923	231093	Agbl5	-1.346017463	0.393376461	down	1.03747E-05	6.02348E-05
G10090_1370	384763	Zfp667	-1.346481593	0.393249928	down	0.005815428	0.01672975
G10090_11669	57778	Fmnl1	-1.346941757	0.393124517	down	7.24605E-19	2.46724E-17
G10090_829	21685	Tef	-1.348842419	0.39260694	down	3.30392E-09	3.5731E-08
G10090_9768	19339	Rab3a	-1.350664601	0.392111375	down	5.59928E-06	3.42105E-05
G10090_12564	101631	Pwwp2b	-1.350914356	0.3920435	down	5.57643E-16	1.47595E-14
G10090_10189	230661	Tesk2	-1.352277864	0.39167315	down	2.71778E-05	0.000142883
G10090_17330	94219	Cnnm2	-1.353658831	0.391298414	down	1.18172E-07	9.78526E-07
G10090_27861	243300	Nyap1	-1.354447122	0.391084667	down	2.70887E-05	0.000142472
G10090_2162	230751	Oscp1	-1.35453816	0.391059989	down	0.000694317	0.002595993
G10090_7405	106633	Ift140	-1.354628838	0.39103541	down	1.69918E-17	5.1073E-16
G10090_7315	74868	Tmem65	-1.357872405	0.390157245	down	1.23576E-18	4.11134E-17
G10090_10067	73451	Zfp763	-1.358352197	0.390027513	down	0.016807401	0.041785977
G10090_29385	331004	Slc9a9	-1.359167154	0.389807255	down	0.002613357	0.008315804
G10090_28052	12217	Bsn	-1.359238671	0.389787932	down	0.001984317	0.006509019
G10090_238	434437	Amt	-1.359261406	0.389781789	down	0.008028499	0.022118127
G10090_3780	235406	Snx33	-1.360935955	0.389329629	down	8.98969E-14	1.83084E-12
G10090_27732	74131	Sash3	-1.361741196	0.389112385	down	6.74619E-15	1.56672E-13
G10090_4025	264134	Ttc26	-1.362207096	0.388986746	down	0.007036745	0.019726724
G10090_2920	230793	Ahdc1	-1.362462783	0.388917813	down	1.65192E-05	9.17151E-05
G10090_33561	117160	Ttyh2	-1.365270853	0.388161557	down	3.77342E-08	3.40024E-07
G10090_26778	27357	Gyg	-1.366796636	0.387751257	down	1.01037E-06	7.06823E-06
G10090_15652	104732	Tedc1	-1.367265295	0.387625317	down	0.011369204	0.029831898
G10090_425	65973	Asph	-1.368669053	0.387248336	down	8.58512E-06	5.07003E-05
G10090_14567	668158	Ccdc85c	-1.370495699	0.386758338	down	0.001337663	0.004603829
G10090_6130	192654	Pla2g15	-1.371250342	0.386556086	down	7.66394E-09	7.77394E-08
G10090_11989	16909	Lmo2	-1.371666316	0.386444646	down	5.80126E-08	5.05003E-07
G10090_31019	74895	Ccdc181	-1.372160667	0.38631225	down	1.27822E-06	8.75467E-06
G10090_7527	22017	Tpmt	-1.374798103	0.385606666	down	3.86773E-07	2.89969E-06
G10090_11609	320534	Tmem104	-1.375020495	0.385547229	down	5.52026E-07	4.03226E-06
G10090_28151	347722	Agap1	-1.375214251	0.385495453	down	4.88085E-07	3.60345E-06
G10090_23471	213988	Tnrc6b	-1.375557446	0.38540376	down	3.69479E-18	1.18116E-16
G10090_20612	14718	Got1	-1.376284115	0.385209686	down	3.12495E-28	2.3482E-26
G10090_31547	72852	Mblac2	-1.376785029	0.385075962	down	1.46678E-08	1.40912E-07
G10090_22291	229474	Fhdc1	-1.378567721	0.38460043	down	0.012790081	0.033088703
G10090_15992	16194	Il6ra	-1.379307935	0.38440315	down	1.49376E-12	2.59374E-11
G10090_32888	78802	Ttc30a1	-1.380604697	0.384057786	down	0.000422689	0.001669685
G10090_11522	18007	Neo1	-1.38221859	0.383628394	down	4.12659E-05	0.000208886
G10090_19163	100201	Tmem64	-1.382627056	0.383519794	down	6.78521E-08	5.82894E-07
G10090_28690	66824	Pycard	-1.384503941	0.383021175	down	1.60176E-08	1.52805E-07
G10090_11016	320204	Etfbkmt	-1.385535757	0.382747337	down	0.000293276	0.001203575
G10090_6094	74253	Klrg2	-1.385895694	0.382651857	down	0.002215312	0.007185612
G10090_33048	243510	Ccdc142	-1.385938248	0.38264057	down	3.81701E-06	2.39135E-05
G10090_2973	214552	Cep164	-1.386713404	0.382435034	down	4.73351E-19	1.65927E-17
G10090_2946	107986	Ddb2	-1.386881498	0.382390477	down	2.58778E-05	0.000136763
G10090_6660	69726	Smyd3	-1.390556784	0.381417572	down	1.89335E-05	0.000103122
G10090_23026	72307	2510002D24Rik	-1.391244017	0.381235926	down	1.18023E-06	8.12614E-06
G10090_24510	12053	Bcl6	-1.391470647	0.381176043	down	4.2702E-05	0.000215322
G10090_30953	16565	Kif21b	-1.392441633	0.380919584	down	2.9641E-27	2.05056E-25
G10090_6357	17347	Mknk2	-1.393250764	0.380706006	down	6.18815E-19	2.13483E-17
G10090_519	109245	Lrrc39	-1.393511397	0.380637235	down	0.002468078	0.007905467
G10090_19296	19253	Ptpn18	-1.394183401	0.380459977	down	0.000221742	0.000939495
G10090_22480	12492	Scarb2	-1.395662395	0.380070144	down	1.40282E-30	1.19882E-28
G10090_22588	329416	Nostrin	-1.399155328	0.379151063	down	4.28683E-06	2.66272E-05
G10090_6728	269608	Plekhg5	-1.399694963	0.379009269	down	0.019602771	0.047665844

G10090_14579	13549	Dyrk1b	-1.399893558	0.3789571	down	1.77712E-30	1.50882E-28
G10090_3502	241627	Wdr76	-1.408001504	0.376833334	down	0.000213272	0.000907428
G10090_11449	23821	Bace1	-1.4132248	0.375471471	down	2.37809E-05	0.000126912
G10090_18066	68636	Fahd1	-1.41327472	0.37545848	down	0.019322263	0.047081361
G10090_17648	15258	Hipk2	-1.41362277	0.375367911	down	1.06387E-27	7.55985E-26
G10090_18518	117198	Ivns1abp	-1.416916943	0.374511794	down	3.49281E-11	5.013E-10
G10090_6354	209131	Snx30	-1.417736014	0.37429923	down	4.64627E-08	4.10473E-07
G10090_12735	17977	Ncoa1	-1.418036687	0.37422123	down	4.43726E-26	2.78682E-24
G10090_13559	246738	Dnajc28	-1.419140386	0.373935051	down	9.68393E-09	9.67283E-08
G10090_21993	73822	Mfsd12	-1.42144763	0.37333751	down	3.9744E-27	2.70653E-25
G10090_14488	327762	Dna2	-1.42237763	0.373096924	down	7.39266E-07	5.28769E-06
G10090_25315	72309	Tmem158	-1.424449952	0.372561383	down	1.41036E-09	1.60912E-08
G10090_2523	224697	Adamts10	-1.426660344	0.371991009	down	1.51658E-06	1.02371E-05
G10090_12974	213002	Ifitm6	-1.428165499	0.371603115	down	0.000101329	0.000472157
G10090_9346	76478	Haus8	-1.42921133	0.371333832	down	0.001156353	0.004043678
G10090_29407	106639	Vmac	-1.429562984	0.371243331	down	4.09217E-08	3.65223E-07
G10090_5287	52028	Bbs1	-1.43058976	0.370979209	down	0.000285461	0.001174819
G10090_2898	212937	Tifab	-1.431894538	0.370643846	down	0.000189552	0.000816603
G10090_4301	70266	Kyat1	-1.43210331	0.370590214	down	0.000906946	0.003276682
G10090_18979	19775	Xpr1	-1.433072658	0.370341298	down	8.1709E-41	1.38746E-38
G10090_12849	24001	Tiam2	-1.437473114	0.369213417	down	0.00721725	0.020172199
G10090_28196	223646	Naprt	-1.437585932	0.369184546	down	0.000103177	0.000479325
G10090_17984	212569	Zfp273	-1.44302511	0.367795285	down	0.000784183	0.002880774
G10090_9054	212285	Arap2	-1.444039632	0.367536737	down	0.000515152	0.001992783
G10090_8698	67378	Bbs2	-1.445430125	0.36718267	down	0.014167439	0.036059814
G10090_31662	27280	Phlda3	-1.445956273	0.367048784	down	0.001275393	0.004416251
G10090_29190	209966	Pgbd5	-1.447785619	0.366583659	down	0.003775025	0.011505468
G10090_949	226421	Rab7b	-1.447801381	0.366579654	down	3.98963E-10	4.91191E-09
G10090_17525	12192	Zfp36l1	-1.448367482	0.366435839	down	0.015137055	0.038215292
G10090_14194	94185	Tnfrsf21	-1.44892264	0.36629486	down	6.08017E-15	1.42726E-13
G10090_532	114714	Rad51c	-1.449829862	0.366064592	down	0.02053185	0.049557678
G10090_8161	107971	Frs3	-1.450746047	0.365832196	down	0.016987794	0.042163137
G10090_16437	233328	Lrrk1	-1.450803476	0.365817633	down	1.68995E-27	1.17532E-25
G10090_3702	21425	Tfeb	-1.451114054	0.36573889	down	2.11187E-10	2.70713E-09
G10090_2760	11549	Adra1a	-1.453421924	0.365154288	down	0.003995154	0.01208622
G10090_12033	50883	Chek2	-1.454606937	0.364854477	down	3.16226E-07	2.40108E-06
G10090_7289	80890	Trim2	-1.455874526	0.364534048	down	0.000143263	0.000637899
G10090_2939	94089	Trim7	-1.456330484	0.364418856	down	0.009847759	0.026353481
G10090_8498	100705	Acacb	-1.457604874	0.364097093	down	0.003547549	0.010890866
G10090_24967	66257	Nicn1	-1.460839547	0.363281663	down	3.90708E-14	8.29388E-13
G10090_15880	20166	Rtkn	-1.46283044	0.362780687	down	0.00078286	0.002876866
G10090_5556	100041420	Gm3325	-1.463500652	0.362612194	down	0.01011429	0.02692819
G10090_7661	73086	Rps6ka5	-1.46599997	0.361984551	down	2.44943E-11	3.61064E-10
G10090_12215	268759	9930012K11Rik	-1.466529935	0.361851602	down	0.000597682	0.002272372
G10090_2506	14347	Fut7	-1.467291741	0.361660579	down	0.001137663	0.003988989
G10090_23601	269529	Fbxo10	-1.468035947	0.361474067	down	4.57678E-08	4.04881E-07
G10090_19031	20933	Med22	-1.469743653	0.361046446	down	4.43481E-20	1.74129E-18
G10090_25937	58223	Mmp19	-1.470196173	0.360933217	down	4.03882E-12	6.58448E-11
G10090_356	70561	Txndc16	-1.47026223	0.360916691	down	1.45589E-16	4.03301E-15
G10090_22968	330050	Fam185a	-1.47115678	0.360692973	down	5.66579E-05	0.000279022
G10090_474	30841	Kdm2b	-1.471712262	0.360554121	down	8.62692E-21	3.60374E-19
G10090_26899	81535	Sgpp1	-1.472004999	0.360480969	down	1.56491E-09	1.77234E-08
G10090_12168	54610	Tbc1d8	-1.472365628	0.360390871	down	1.03184E-05	5.99592E-05
G10090_1273	18514	Pbx1	-1.473999256	0.359983015	down	9.56682E-07	6.72868E-06
G10090_25867	140887	Lnx2	-1.474092534	0.359959741	down	3.18753E-08	2.90432E-07
G10090_6700	232334	Vgll4	-1.474228984	0.359925698	down	2.44578E-08	2.26637E-07
G10090_15857	22042	Tfrc	-1.474967984	0.359741378	down	2.80061E-06	1.81098E-05
G10090_32209	21858	Timp2	-1.47678636	0.359288245	down	1.93494E-11	2.89798E-10
G10090_13790	18846	Plxna3	-1.482437721	0.357883585	down	7.31843E-07	5.23747E-06
G10090_11897	12009	Cep131	-1.482798069	0.357794206	down	4.56092E-10	5.57327E-09
G10090_10139	74682	Wdr35	-1.483531155	0.357612444	down	1.46173E-07	1.18782E-06
G10090_10144	12189	Brca1	-1.483691449	0.357572713	down	0.005756452	0.016581982
G10090_1510	21847	Klf10	-1.484809379	0.357295741	down	1.23126E-09	1.41964E-08

G10090_24397	224833	AI661453	-1.492048593	0.355507378	down	0.001746472	0.005826772
G10090_25915	76498	Paqr4	-1.492518467	0.355391611	down	1.4108E-05	7.9647E-05
G10090_3008	72475	Ssbp3	-1.493211442	0.355220946	down	2.5886E-23	1.33252E-21
G10090_13298	18627	Per2	-1.494248913	0.354965591	down	3.33596E-07	2.52709E-06
G10090_15060	225870	Rin1	-1.496556942	0.35439817	down	0.000444591	0.001744605
G10090_9345	72512	Tmem173	-1.497687168	0.354120638	down	6.27671E-07	4.55427E-06
G10090_17939	19341	Rab4a	-1.49935682	0.353711046	down	1.86099E-13	3.62747E-12
G10090_14400	74580	Pyroxd2	-1.499937457	0.353568718	down	0.004560739	0.01354034
G10090_19851	102448	Xylb	-1.500391834	0.353457379	down	2.73922E-05	0.000143894
G10090_7374	18600	Padi2	-1.502822717	0.352862319	down	1.30914E-06	8.9477E-06
G10090_10724	381199	Tmem151a	-1.504603815	0.352426957	down	0.000249249	0.001042856
G10090_21317	11307	Abcg1	-1.505407495	0.352230686	down	6.05696E-12	9.61101E-11
G10090_2689	18796	Plcb2	-1.505479368	0.352213139	down	1.77978E-13	3.47841E-12
G10090_10939	12615	Cenpa	-1.506111723	0.352058792	down	0.020403311	0.049292922
G10090_9417	329877	Dennd4c	-1.508064131	0.351582671	down	4.39388E-26	2.77536E-24
G10090_10424	74152	Stra6l	-1.510367865	0.351021702	down	0.001234706	0.004285582
G10090_10090	68521	Fam189b	-1.511910533	0.350646557	down	1.46934E-12	2.55813E-11
G10090_2746	104923	Adi1	-1.515658131	0.349736886	down	1.61162E-09	1.81969E-08
G10090_6744	67149	Nkain1	-1.515674927	0.349732815	down	1.15514E-09	1.34157E-08
G10090_25429	11481	Acvr2b	-1.515949723	0.349666206	down	8.78583E-09	8.82972E-08
G10090_6026	74239	Iqce	-1.516308036	0.349579372	down	1.43653E-08	1.38515E-07
G10090_19342	68178	Cgnl1	-1.523795967	0.347769673	down	9.56965E-14	1.9396E-12
G10090_28368	74114	Crot	-1.524710865	0.347549201	down	1.96619E-21	8.54082E-20
G10090_178	13360	Dhcr7	-1.525309469	0.347405026	down	2.10833E-11	3.14327E-10
G10090_10990	73852	D3Ertd751e	-1.526089998	0.347217123	down	9.95908E-05	0.000464431
G10090_24173	235527	Plscr4	-1.528458098	0.346647654	down	0.003191439	0.009906948
G10090_9928	56218	Patz1	-1.529181734	0.346473824	down	2.31733E-07	1.8089E-06
G10090_13459	22685	Zfp239	-1.5293824	0.346425636	down	0.003440758	0.010587882
G10090_5372	27360	Add3	-1.532256136	0.34573627	down	2.07732E-16	5.67035E-15
G10090_12719	668880	Stard9	-1.534295169	0.345247969	down	9.69379E-12	1.49996E-10
G10090_13800	226265	Eno4	-1.537160663	0.344562915	down	0.000960941	0.003446053
G10090_25094	216543	Cep68	-1.538357407	0.344277211	down	6.05403E-09	6.24755E-08
G10090_3462	268857	Nlrc3	-1.538457168	0.344253406	down	1.36346E-05	7.72747E-05
G10090_570	15216	Hfe	-1.539004639	0.344122794	down	3.16233E-06	2.01596E-05
G10090_22886	11689	Alox5	-1.542004613	0.34340796	down	0.000362258	0.001449807
G10090_11218	15531	Ndst1	-1.54225197	0.343349086	down	0.001382927	0.004739651
G10090_19702	71819	Kif23	-1.542456282	0.343300465	down	0.002101514	0.006853904
G10090_12941	239570	Ttc38	-1.543158702	0.34313336	down	1.09881E-18	3.69329E-17
G10090_10268	246102	Rttn	-1.544783411	0.342747153	down	4.75442E-06	2.93503E-05
G10090_6001	17305	Mfng	-1.545128752	0.342665119	down	0.001127062	0.003955
G10090_18243	12504	Cd4	-1.545140066	0.342662432	down	0.001944832	0.006398761
G10090_18497	107568	Wwp1	-1.547142774	0.342187088	down	4.94001E-07	3.63891E-06
G10090_12576	216438	March9	-1.54931947	0.341671195	down	0.000167618	0.000732841
G10090_1152	14051	Eya4	-1.550767171	0.34132851	down	0.000678026	0.002543083
G10090_23838	13848	Ephb6	-1.550840776	0.341311097	down	0.009448115	0.025423771
G10090_2499	241274	Pnpla7	-1.551537189	0.34114638	down	4.45955E-20	1.74577E-18
G10090_23023	110958	M1ap	-1.551832986	0.341076441	down	0.000274901	0.001138529
G10090_17655	71687	Tmem25	-1.552485664	0.340922172	down	0.015676264	0.039325774
G10090_14907	223649	Nrbp2	-1.556510988	0.339972277	down	0.007649997	0.021218436
G10090_32524	244238	Mrgpre	-1.556796795	0.339904933	down	0.003227346	0.010008908
G10090_14800	52055	Rab11fip5	-1.562108704	0.338655726	down	7.65735E-27	5.05656E-25
G10090_14996	233571	P2ry6	-1.562481836	0.338568149	down	3.88504E-06	2.42815E-05
G10090_12404	230872	Crocc	-1.56262118	0.33853545	down	2.34165E-07	1.82462E-06
G10090_30961	77938	Fam53b	-1.56310386	0.338422206	down	1.09894E-21	4.98909E-20
G10090_20114	66090	Ypel3	-1.564224787	0.338159365	down	1.0416E-17	3.18198E-16
G10090_7350	20503	Slc16a7	-1.56619571	0.337697707	down	1.66186E-07	1.33634E-06
G10090_21152	27494	Amot	-1.567014737	0.337506049	down	9.26582E-06	5.43764E-05
G10090_21927	382913	Neil2	-1.570419661	0.336710435	down	0.003799935	0.011567904
G10090_11860	668923	Zfp442	-1.573523771	0.335986746	down	7.80213E-08	6.63283E-07
G10090_12209	60599	Trp53inp1	-1.574906657	0.335664843	down	3.12419E-11	4.52368E-10
G10090_16288	320528	Vps13c	-1.576307102	0.335339166	down	7.0014E-13	1.27498E-11
G10090_12333	217304	Cd300lb	-1.577700532	0.335015434	down	2.12633E-15	5.2987E-14
G10090_11841	67590	Tctn3	-1.579000764	0.334713637	down	6.55556E-21	2.76497E-19

G10090_11815	140795	P2ry14	-1.580516493	0.334362164	down	2.13639E-07	1.68375E-06
G10090_7710	29856	Smtn	-1.580862266	0.334282036	down	2.54434E-05	0.00013474
G10090_3037	81004	Tbl1xr1	-1.581327297	0.334174303	down	6.16834E-17	1.80427E-15
G10090_16205	242341	Atp6v0d2	-1.584776679	0.33337627	down	6.85752E-05	0.000331713
G10090_11722	79196	Osbpl5	-1.587615366	0.332720955	down	0.001046212	0.003722237
G10090_10483	78408	Fam131a	-1.588102879	0.332608541	down	2.84091E-05	0.000148635
G10090_23404	213575	Dync2li1	-1.590340875	0.332092978	down	0.001508733	0.005117169
G10090_7063	56711	Plag1	-1.594221188	0.331200972	down	1.95027E-07	1.5492E-06
G10090_89	23876	Fbln5	-1.596057793	0.330779609	down	0.011093765	0.029191182
G10090_700	213989	Tmem82	-1.5976516	0.330414384	down	0.000978505	0.003501357
G10090_15348	76850	Ago4	-1.598677754	0.330179452	down	1.06393E-25	6.56174E-24
G10090_514	70552	Lrrc56	-1.599344516	0.33002689	down	1.43362E-05	8.0726E-05
G10090_17044	16970	Lrmp	-1.602453203	0.329316521	down	4.85162E-08	4.27747E-07
G10090_1904	329540	Nol4l	-1.606320451	0.328434947	down	1.40573E-10	1.84537E-09
G10090_7581	75735	Pank1	-1.607828385	0.328091839	down	6.32433E-06	3.82119E-05
G10090_5736	12289	Cacna1d	-1.608283125	0.32798844	down	3.79151E-06	2.37993E-05
G10090_9979	52538	Acaa2	-1.608434471	0.327954035	down	2.20442E-14	4.83604E-13
G10090_15452	20527	Slc2a3	-1.609351334	0.327745679	down	1.45053E-08	1.39568E-07
G10090_10870	26411	Map4k1	-1.610331594	0.327523063	down	0.000201277	0.00086077
G10090_15457	20648	Snta1	-1.611639151	0.327226354	down	3.43102E-08	3.10669E-07
G10090_1499	80911	Acox3	-1.614396765	0.32660148	down	3.76563E-16	1.00893E-14
G10090_9281	208285	Cyp4f17	-1.615223514	0.326414372	down	0.001576146	0.00531822
G10090_10363	12589	Ift81	-1.616677025	0.326085676	down	0.000168341	0.000734911
G10090_986	329828	AI464131	-1.61702164	0.326007794	down	0.001666705	0.005589167
G10090_33669	277010	Marveld1	-1.61778504	0.325835333	down	3.33512E-07	2.52709E-06
G10090_3887	226409	Zranb3	-1.619230983	0.325508928	down	0.001496041	0.005082031
G10090_10087	232811	Kmt5c	-1.62333037	0.324585314	down	3.82988E-19	1.3534E-17
G10090_12246	74764	Klc4	-1.625906714	0.324006191	down	1.15145E-10	1.52845E-09
G10090_4527	235534	Pxylp1	-1.626098263	0.323963175	down	0.004740747	0.014017473
G10090_1294	21885	Tle1	-1.626675228	0.323833641	down	4.09863E-15	9.81495E-14
G10090_4455	245007	Zbtb38	-1.62997239	0.323094391	down	7.98447E-16	2.07549E-14
G10090_10080	67454	Ikbip	-1.632363346	0.322559375	down	8.92795E-08	7.50694E-07
G10090_10406	268445	Ankrd13b	-1.636133246	0.321717597	down	1.64963E-11	2.4849E-10
G10090_18855	27375	Tjp3	-1.637622747	0.321385613	down	1.38634E-09	1.58586E-08
G10090_2828	223433	Otulinl	-1.638539211	0.32118152	down	2.95567E-22	1.42603E-20
G10090_3764	545622	Ptpn3	-1.641459214	0.320532108	down	1.25118E-05	7.15627E-05
G10090_7267	224674	Slc37a1	-1.645860727	0.319555688	down	0.009848039	0.026353481
G10090_11131	20148	Dhrs3	-1.646251688	0.319469102	down	1.45755E-09	1.66006E-08
G10090_25327	22117	Tst	-1.653877641	0.317784873	down	0.000315798	0.001281121
G10090_1400	320365	Fry	-1.654541622	0.317638651	down	8.08993E-12	1.26982E-10
G10090_13980	26401	Map3k1	-1.655908836	0.317337773	down	1.01122E-14	2.29543E-13
G10090_6611	64384	Sirt3	-1.663500332	0.31567232	down	1.09114E-11	1.67646E-10
G10090_9560	16832	Ldhb	-1.665120028	0.315318118	down	0.001825145	0.006052188
G10090_5185	52685	Cd300lg	-1.665603853	0.31521239	down	0.002097293	0.006841842
G10090_15849	268515	Bahcc1	-1.668439698	0.314593398	down	0.000876534	0.003177343
G10090_6749	224014	Fgd4	-1.668574203	0.31456407	down	2.43419E-05	0.000129431
G10090_1340	380839	Serpinb1c	-1.669633561	0.314333173	down	2.29107E-05	0.000122541
G10090_4518	18596	Pdgfrb	-1.669663616	0.314326625	down	4.99229E-13	9.23256E-12
G10090_23503	230837	Asap3	-1.672790296	0.313646138	down	0.009373709	0.025250751
G10090_6177	77864	Ypel2	-1.673228213	0.313550948	down	2.95727E-18	9.54724E-17
G10090_104	121022	Mrps6	-1.673245557	0.313547179	down	3.22644E-13	6.07863E-12
G10090_18111	16480	Jup	-1.675244499	0.313113041	down	0.000117814	0.000537858
G10090_32926	52187	Rragd	-1.67639109	0.312864291	down	2.15984E-05	0.000116357
G10090_8462	12894	Cpt1a	-1.678880872	0.31232482	down	2.32223E-37	3.13023E-35
G10090_16547	241062	Pgap1	-1.682418069	0.311560001	down	7.4679E-14	1.53526E-12
G10090_6430	260305	Nphp4	-1.682676016	0.3115043	down	2.40847E-05	0.000128324
G10090_5107	17196	Mbp	-1.682931246	0.311449196	down	2.9294E-42	5.31971E-40
G10090_4166	15184	Hdac5	-1.686114471	0.310762759	down	2.69059E-38	3.78274E-36
G10090_4953	19280	Ptprs	-1.6869351	0.310586042	down	5.29909E-23	2.67512E-21
G10090_10932	106068	Slc45a4	-1.687286416	0.310510419	down	9.58303E-14	1.9396E-12
G10090_22922	69310	Pacrg	-1.687722366	0.310416604	down	0.006689048	0.018869321
G10090_25427	232984	B3gnt8	-1.688223623	0.31030877	down	4.37042E-09	4.64957E-08
G10090_19338	667214	9930111J21Rik1	-1.689951925	0.309937253	down	2.00885E-05	0.000108941

G10090_2338	353211	Prune2	-1.690347628	0.309852255	down	1.30871E-05	7.45271E-05
G10090_4180	71887	Ppm1j	-1.690723034	0.309771638	down	0.004588117	0.013612352
G10090_24234	194655	Klf11	-1.691793528	0.30954187	down	2.35419E-15	5.84079E-14
G10090_27204	73251	Setd7	-1.69244804	0.309401471	down	2.56312E-15	6.32318E-14
G10090_22916	217140	Scrn2	-1.697544685	0.308310369	down	0.00187253	0.006192039
G10090_18761	269060	Dagla	-1.697795905	0.308256687	down	2.23481E-08	2.08567E-07
G10090_7412	263406	Plekhg3	-1.698146296	0.308181829	down	2.65796E-15	6.52022E-14
G10090_7398	217333	Trim47	-1.700224647	0.307738181	down	5.36129E-10	6.50872E-09
G10090_9351	109889	Mzf1	-1.701144044	0.307542129	down	0.001155686	0.004042427
G10090_5443	12393	Runx2	-1.70254706	0.30724319	down	7.72396E-12	1.21529E-10
G10090_11684	68428	Steap3	-1.702783495	0.307192842	down	1.0828E-15	2.75976E-14
G10090_19631	22701	Zfp41	-1.703153373	0.307114094	down	4.87929E-09	5.13661E-08
G10090_29218	11854	Rhod	-1.704361686	0.306856982	down	0.002585239	0.008238362
G10090_20087	70415	Stk26	-1.705411422	0.306633787	down	0.0153734	0.038714792
G10090_14243	234797	6430548M08Rik	-1.705553086	0.306603679	down	2.09286E-26	1.34799E-24
G10090_2385	12121	Bicd1	-1.705815837	0.306547844	down	0.016590078	0.041340817
G10090_32941	67266	Fam69a	-1.706941752	0.3063087	down	1.55232E-21	6.85695E-20
G10090_4252	17528	Mpz	-1.708333817	0.306013283	down	0.005413213	0.015738885
G10090_5489	18439	P2rx7	-1.710663077	0.305519617	down	1.15152E-07	9.5534E-07
G10090_5310	18628	Per3	-1.710943317	0.305460277	down	5.77515E-08	5.03101E-07
G10090_29031	67856	Echdc3	-1.712678208	0.305093171	down	0.013899062	0.035490118
G10090_13118	18974	Pole2	-1.714246481	0.304761701	down	0.000199022	0.000852903
G10090_32353	15242	Hhex	-1.715684578	0.304458062	down	2.59709E-15	6.3949E-14
G10090_3910	19277	Ptpro	-1.716814182	0.30421977	down	6.65957E-10	8.00312E-09
G10090_10667	14166	Fgf11	-1.717121661	0.30415494	down	1.67735E-07	1.34713E-06
G10090_5309	70719	Arhgap45	-1.72089371	0.303360739	down	1.65895E-25	9.99189E-24
G10090_13943	70152	Mettl7a1	-1.725218958	0.302452616	down	3.51429E-38	4.88822E-36
G10090_2189	223752	Gramd4	-1.731916032	0.301051867	down	1.27954E-09	1.46883E-08
G10090_21939	71769	Bbs10	-1.732281715	0.300975568	down	4.16835E-08	3.71261E-07
G10090_5640	17193	Mbd4	-1.733346441	0.300753527	down	4.43369E-07	3.29941E-06
G10090_15930	74478	Snx29	-1.741211271	0.299118434	down	2.44697E-28	1.86012E-26
G10090_11425	244202	Nlrp10	-1.741944451	0.29896646	down	5.06922E-22	2.38417E-20
G10090_22718	66972	Slc25a23	-1.742663492	0.298817492	down	8.55684E-12	1.33509E-10
G10090_2572	21813	Tgfbr2	-1.742909214	0.298766602	down	4.65795E-15	1.10732E-13
G10090_6931	100532	Rell1	-1.744811099	0.298373001	down	4.4332E-18	1.41032E-16
G10090_30458	17064	Cd93	-1.748338015	0.297644468	down	2.39104E-13	4.57059E-12
G10090_33753	78004	Prr15	-1.748395172	0.297632676	down	0.002410813	0.007750523
G10090_5408	225187	Ankrd29	-1.758724415	0.29550933	down	0.008035024	0.022130289
G10090_2249	268977	Ltbp1	-1.762133659	0.294811834	down	0.010497972	0.027808141
G10090_3566	67893	Tmem86a	-1.762380617	0.294761373	down	1.60226E-08	1.52805E-07
G10090_2943	104175	Sbk1	-1.763602155	0.294511902	down	1.3173E-05	7.49181E-05
G10090_11420	233071	Arhgap33	-1.768523931	0.293508882	down	0.006517413	0.01845283
G10090_4760	51938	Ccdc39	-1.770456543	0.293115966	down	0.017862033	0.044040369
G10090_19719	19414	Rasa3	-1.772873378	0.292625342	down	4.27016E-32	4.07536E-30
G10090_32721	216134	Pdxk	-1.774581994	0.292278985	down	1.72086E-20	6.98765E-19
G10090_16621	72691	Calhm2	-1.774596256	0.292276095	down	1.59305E-09	1.80183E-08
G10090_7342	97130	C77080	-1.77806735	0.291573731	down	1.57231E-13	3.09632E-12
G10090_18110	106369	Ypel1	-1.781190945	0.290943124	down	0.001628709	0.005470548
G10090_23627	15364	Hmga2	-1.782280727	0.290723434	down	2.88379E-21	1.23625E-19
G10090_1526	11520	Plin2	-1.783142816	0.290549763	down	6.61757E-38	9.10787E-36
G10090_14789	76373	Zfp773	-1.783751108	0.290427283	down	0.001822718	0.006047207
G10090_6677	74201	Cep97	-1.784082364	0.290360606	down	1.88721E-11	2.82973E-10
G10090_6576	114663	Impa2	-1.786229269	0.289928835	down	9.48714E-13	1.69228E-11
G10090_256	78795	Armc9	-1.788614992	0.289449789	down	2.54207E-06	1.66188E-05
G10090_26628	66514	Asrgl1	-1.791397076	0.288892153	down	3.97413E-05	0.000201715
G10090_6141	223645	Mroh6	-1.792296975	0.288712009	down	0.001584521	0.005340969
G10090_5655	12287	Cacna1b	-1.792604738	0.288650426	down	0.000125092	0.000566727
G10090_582	52662	Ldlrad4	-1.793856214	0.288400143	down	0.000142409	0.000635277
G10090_18207	16598	Klf2	-1.794405582	0.288290343	down	2.16767E-05	0.000116683
G10090_24600	76467	Msrb2	-1.79498794	0.288173996	down	0.000149415	0.00066291
G10090_4779	14007	Celf2	-1.7957441	0.288022995	down	1.80896E-21	7.9104E-20
G10090_27870	223254	Farp1	-1.797703544	0.287632072	down	0.008497352	0.023204445
G10090_20596	100039596	Tcf24	-1.797958006	0.287581344	down	0.004286925	0.012817642

G10090_21820	57740	Stk32c	-1.798318156	0.287509562	down	0.002813127	0.008856643
G10090_19203	18105	Nqo2	-1.799116769	0.287350454	down	1.41665E-12	2.47691E-11
G10090_13515	215653	Rassf2	-1.805340891	0.286113427	down	9.11877E-15	2.08805E-13
G10090_9580	56742	Psrc1	-1.80582025	0.286018377	down	0.001960414	0.006441922
G10090_577	55925	Syt8	-1.809187886	0.285351512	down	0.019990242	0.048423963
G10090_4761	19016	Pparg	-1.811204374	0.284952949	down	0.000187368	0.000808175
G10090_9741	13139	Dgka	-1.811731076	0.284848936	down	8.42813E-15	1.93669E-13
G10090_5911	67729	Mansc1	-1.812097845	0.28477653	down	4.60146E-05	0.000230602
G10090_524	108115	Slco4a1	-1.812380718	0.284720698	down	1.79345E-42	3.30272E-40
G10090_12444	75744	Svip	-1.813063251	0.28458603	down	2.29243E-05	0.000122541
G10090_9747	338372	Map3k9	-1.81310928	0.284576951	down	7.05001E-18	2.18952E-16
G10090_5303	236266	Alms1	-1.817940353	0.283625597	down	6.21643E-08	5.365E-07
G10090_4293	14263	Fmo5	-1.818098731	0.283594463	down	2.33869E-13	4.48362E-12
G10090_23622	215821	Arfgef3	-1.820440217	0.283134564	down	7.69302E-06	4.57418E-05
G10090_7524	17691	Sik1	-1.820900723	0.283044202	down	4.2258E-10	5.17829E-09
G10090_24174	18604	Pdk2	-1.821207824	0.282983958	down	1.83642E-15	4.61754E-14
G10090_30559	21646	Tcte2	-1.821819548	0.282863994	down	0.017183979	0.042585392
G10090_14528	15559	Htr2b	-1.827484953	0.281755377	down	1.63543E-05	9.08767E-05
G10090_11559	242466	Zfp462	-1.827946349	0.281665281	down	1.00226E-06	7.01905E-06
G10090_3104	72754	Arhgef10l	-1.828478722	0.281561363	down	9.74541E-17	2.75803E-15
G10090_13198	20913	Stxbp4	-1.828909277	0.281477347	down	8.50406E-14	1.73735E-12
G10090_28168	17095	Lyl1	-1.832271275	0.280822167	down	9.69044E-13	1.72384E-11
G10090_272	78779	Spata2l	-1.834627395	0.28036392	down	9.21231E-14	1.87327E-12
G10090_16389	80909	Castor2	-1.836075894	0.280082569	down	1.06301E-22	5.30489E-21
G10090_11548	12416	Cbx2	-1.836624822	0.279976022	down	0.00363282	0.01112913
G10090_7837	212706	N4bp3	-1.836866404	0.279929143	down	4.94389E-08	4.35296E-07
G10090_24284	66994	Cep19	-1.837584824	0.279789781	down	3.07185E-06	1.96788E-05
G10090_27608	399603	Fam84b	-1.839872349	0.2793465	down	6.14398E-29	4.92838E-27
G10090_12479	16842	Lef1	-1.8404815	0.279228576	down	0.00288767	0.009073847
G10090_7678	14190	Fgl2	-1.840935423	0.279140735	down	0.000139681	0.000624814
G10090_10225	108100	Baiap2	-1.84317858	0.278707054	down	3.30849E-18	1.06286E-16
G10090_13609	67155	Smarca2	-1.847546608	0.277864492	down	2.42333E-24	1.32021E-22
G10090_17336	241303	Fam78a	-1.850217623	0.277350528	down	1.02485E-14	2.31833E-13
G10090_10248	24099	Tnfsf13b	-1.851029111	0.277194567	down	0.000423023	0.001670501
G10090_16930	74134	Cyp2s1	-1.8521777	0.276973969	down	0.000732437	0.002715998
G10090_17932	231670	Fbxo21	-1.852689069	0.276875812	down	6.65266E-29	5.30387E-27
G10090_2789	18441	P2ry1	-1.853243045	0.276769516	down	4.15111E-05	0.000210046
G10090_20340	320024	Nceh1	-1.856118832	0.276218369	down	2.52994E-34	2.8033E-32
G10090_9896	19099	Mapk8ip1	-1.86329705	0.274847441	down	0.003828676	0.011647263
G10090_15584	20708	Serpinb6b	-1.86387291	0.274737755	down	0.000414203	0.001638639
G10090_5852	18576	Pde3b	-1.86609569	0.274314789	down	0.000158339	0.0006973
G10090_374	16581	Kifc2	-1.866889279	0.274163937	down	1.45172E-08	1.39568E-07
G10090_16471	218121	Mboat1	-1.876199063	0.27240044	down	1.10435E-07	9.18533E-07
G10090_30671	21350	Tal2	-1.876218798	0.272396713	down	1.17042E-14	2.62042E-13
G10090_3310	74202	Fblim1	-1.876971127	0.272254702	down	3.60467E-07	2.71493E-06
G10090_33567	121021	Cspg4	-1.878537858	0.271959201	down	0.00167686	0.0056189
G10090_17643	114229	Kiss1r	-1.880865007	0.271520869	down	0.001937948	0.006380928
G10090_17602	14296	Frat1	-1.881660801	0.271371139	down	0.000202232	0.000864395
G10090_27088	211389	Suox	-1.88214543	0.271279995	down	0.000233385	0.00098309
G10090_15239	213402	Armc2	-1.885220819	0.270702324	down	0.015457196	0.038889752
G10090_12095	76074	Gbp8	-1.885259922	0.270694988	down	0.002770517	0.008737218
G10090_15285	207798	Gramd1c	-1.885483006	0.270653133	down	0.00715148	0.02000976
G10090_15313	16403	Itga6	-1.888279999	0.270128919	down	9.16114E-08	7.69312E-07
G10090_1357	320869	Spata33	-1.890658692	0.269683901	down	0.017713376	0.043698564
G10090_25998	66881	Pcyox1	-1.892114726	0.269411861	down	4.72855E-27	3.1869E-25
G10090_22277	236576	Spry3	-1.892414278	0.269355928	down	3.82996E-15	9.18839E-14
G10090_2260	215690	Nav1	-1.893752631	0.269106169	down	4.61673E-24	2.46382E-22
G10090_10101	109050	Inka2	-1.895093821	0.268856113	down	0.002586157	0.008239279
G10090_24863	13819	Epas1	-1.897249326	0.26845472	down	3.21831E-05	0.000166322
G10090_30826	14281	Fos	-1.903570684	0.267281023	down	3.80597E-12	6.22818E-11
G10090_1951	22390	Wee1	-1.905769304	0.266874006	down	5.42173E-08	4.74492E-07
G10090_29914	242681	Rab42	-1.913190475	0.265504741	down	0.008805687	0.023916568
G10090_3227	223753	Cerk	-1.917647646	0.264685736	down	2.60021E-18	8.43618E-17

G10090_14350	268512	Slc26a11	-1.91818829	0.264586565	down	1.0393E-12	1.83633E-11
G10090_23234	93840	Vangl2	-1.919166131	0.264407292	down	5.42888E-06	3.32627E-05
G10090_12866	212073	Syne3	-1.920012661	0.264252191	down	1.55528E-13	3.0718E-12
G10090_12996	97761	Sgsm2	-1.923468661	0.263619928	down	3.70248E-10	4.57993E-09
G10090_16495	236727	Slc9a7	-1.926836331	0.26300528	down	9.98744E-08	8.36016E-07
G10090_4659	229706	Slc6a17	-1.927645257	0.262857853	down	3.96117E-06	2.47218E-05
G10090_31743	272396	Tarsl2	-1.928547322	0.262693549	down	1.89198E-13	3.6812E-12
G10090_2222	504193	Npcd	-1.930691667	0.262303385	down	0.015985705	0.039987199
G10090_1119	170744	Tlr8	-1.934722525	0.261571539	down	9.83963E-12	1.51893E-10
G10090_21766	216198	Tcp11l2	-1.936319853	0.261282091	down	3.84417E-34	4.22375E-32
G10090_1946	26382	Fgd2	-1.936950735	0.261167859	down	2.91126E-07	2.23123E-06
G10090_497	74048	Vsir	-1.940498907	0.26052633	down	2.16649E-19	7.95699E-18
G10090_3470	14182	Fgfr1	-1.945874106	0.259557466	down	1.45193E-10	1.9003E-09
G10090_6573	68870	Ak8	-1.948042761	0.259167594	down	4.68362E-07	3.46763E-06
G10090_12491	15064	Mr1	-1.949764701	0.258858446	down	3.20311E-06	2.04097E-05
G10090_23278	214931	Fbxl16	-1.95270697	0.258331062	down	0.007388893	0.020581547
G10090_28549	216148	Shc2	-1.953014683	0.258275968	down	0.00029107	0.00119527
G10090_428	16419	Itgb5	-1.957452893	0.257482646	down	4.27699E-28	3.15941E-26
G10090_17644	242297	Fam110b	-1.957812422	0.257418488	down	0.000626381	0.002365665
G10090_10937	215243	Traf3ip3	-1.969913642	0.255268311	down	7.69892E-19	2.60786E-17
G10090_13148	22317	Vamp1	-1.970094715	0.255236274	down	4.82788E-09	5.08658E-08
G10090_8846	12877	Cpeb1	-1.970142063	0.255227898	down	6.95855E-05	0.000336476
G10090_501	70727	Rasgef1a	-1.977475562	0.253933817	down	0.000105627	0.00048853
G10090_9110	18483	Palm	-1.981227134	0.253274347	down	0.002255239	0.007304247
G10090_23008	668039	Gm14434	-1.990643171	0.251626684	down	0.001510098	0.00512047
G10090_15361	239554	Foxred2	-1.990797798	0.251599716	down	1.15907E-13	2.33152E-12
G10090_7643	66279	Tmem218	-1.991479709	0.251480822	down	1.40812E-07	1.14786E-06
G10090_18442	12767	Cxcr4	-1.994344831	0.250981889	down	3.97612E-20	1.58984E-18
G10090_14536	223666	Arhgap39	-1.998236885	0.250305711	down	5.41543E-39	7.95581E-37
G10090_21976	106618	Wdr90	-2.003166286	0.249451926	down	6.6651E-06	4.01041E-05
G10090_16307	272347	Zfp398	-2.003942363	0.249317773	down	8.33921E-17	2.37549E-15
G10090_20910	415115	Neurl2	-2.007313956	0.248735795	down	1.96456E-15	4.91141E-14
G10090_12650	77889	Lbh	-2.009963542	0.248279398	down	6.05688E-11	8.4159E-10
G10090_1806	24058	Sigirr	-2.013961124	0.24759239	down	6.67307E-05	0.00032411
G10090_25350	67731	Fbxo32	-2.018843992	0.246755818	down	6.71997E-07	4.84097E-06
G10090_18930	242705	E2f2	-2.019325046	0.246673553	down	2.55171E-06	1.66568E-05
G10090_24930	239833	Lmln	-2.024285601	0.245826849	down	3.75327E-07	2.81711E-06
G10090_2239	17859	Mxi1	-2.028270949	0.245148706	down	1.25725E-33	1.34742E-31
G10090_16753	207683	Igsf11	-2.028560257	0.24509955	down	0.014565671	0.036922479
G10090_14371	52570	Ccdc69	-2.028722258	0.24507203	down	0.000143073	0.000637804
G10090_17846	213068	Tmem71	-2.04063435	0.243056842	down	9.33683E-05	0.000438345
G10090_32801	11732	Ank	-2.042901909	0.242675117	down	7.25695E-23	3.64941E-21
G10090_23998	17685	Msh2	-2.043214356	0.242622566	down	3.07193E-15	7.47961E-14
G10090_30036	621823	Psme2b	-2.045437246	0.242249023	down	1.10704E-06	7.66663E-06
G10090_14116	224829	Trerf1	-2.048015088	0.241816553	down	8.63253E-07	6.11432E-06
G10090_1703	66395	Ahnak	-2.049800647	0.241517453	down	1.23295E-55	4.24231E-53
G10090_16689	171580	Mical1	-2.050163886	0.241456652	down	5.2053E-16	1.38332E-14
G10090_31213	17122	Mxd4	-2.054780561	0.240685217	down	4.62992E-52	1.34525E-49
G10090_10759	12298	Cacnb4	-2.055860258	0.240505158	down	0.017936201	0.044173259
G10090_6499	329650	Med12l	-2.056200211	0.240448493	down	0.001401846	0.004799458
G10090_14317	83554	Fstl3	-2.058195927	0.240116104	down	0.000250962	0.001048346
G10090_4800	286942	Kif19a	-2.060011545	0.239814111	down	0.002214436	0.007184553
G10090_7959	330460	Tmem150b	-2.060106668	0.239798299	down	1.00392E-07	8.39812E-07
G10090_8575	12695	Patj	-2.06544134	0.238913231	down	0.014082234	0.035891854
G10090_10970	26404	Map3k12	-2.067524398	0.238568522	down	1.38455E-31	1.24849E-29
G10090_9657	381126	Garem1	-2.068967821	0.238329952	down	1.26059E-09	1.44963E-08
G10090_22130	228413	Prrg4	-2.071575531	0.237899553	down	1.40064E-09	1.59942E-08
G10090_9793	54635	Pdgfc	-2.074073893	0.237487932	down	1.32122E-05	7.50756E-05
G10090_6746	207592	Tbc1d16	-2.074522692	0.237414064	down	1.04504E-10	1.39999E-09
G10090_5459	68318	Aph1c	-2.082920285	0.236036146	down	5.39651E-31	4.70396E-29
G10090_20588	21824	Thbd	-2.086661718	0.235424812	down	5.29405E-14	1.10752E-12
G10090_1901	19415	Rasal1	-2.092316797	0.234503799	down	8.74617E-10	1.03583E-08
G10090_5823	13821	Epb41l1	-2.101355942	0.233039119	down	3.39689E-37	4.48629E-35

G10090_20852	544922	Zkscan4	-2.104691782	0.232500902	down	0.000170325	0.00074258
G10090_9587	211535	Ccdc114	-2.111713738	0.231372012	down	1.20818E-05	6.92241E-05
G10090_10341	20341	Selenbp1	-2.114417791	0.230938756	down	7.97079E-25	4.49216E-23
G10090_8148	207818	Smagp	-2.116691839	0.230575025	down	1.5393E-08	1.47338E-07
G10090_20743	16513	Kcnj10	-2.11975881	0.230085375	down	5.08726E-06	3.12575E-05
G10090_8410	269233	Fam171a1	-2.122756577	0.229607779	down	0.00048579	0.001893769
G10090_12932	494448	Cbx6	-2.127484557	0.228856543	down	6.67505E-10	8.01435E-09
G10090_2206	18032	Nfix	-2.130080939	0.228445046	down	5.58562E-07	4.07772E-06
G10090_5932	100503085	Klhl3	-2.135983862	0.227512251	down	0.00057063	0.002184771
G10090_32285	270160	Rab39	-2.136565082	0.227420611	down	0.000141627	0.00063287
G10090_30808	230126	Shb	-2.139616713	0.226940073	down	1.85704E-17	5.55626E-16
G10090_23655	20887	Sult1a1	-2.14148468	0.226646427	down	0.001082041	0.003823698
G10090_11549	278097	Armcx6	-2.147872256	0.225645161	down	0.009902103	0.026454841
G10090_2403	211401	Mtss1	-2.149214796	0.225435278	down	1.83883E-22	8.97117E-21
G10090_33398	226999	Slc9a2	-2.152908534	0.224858833	down	0.003345975	0.010330253
G10090_5558	26364	Adgre5	-2.155543475	0.224448526	down	8.02209E-12	1.26068E-10
G10090_18069	15442	Hpse	-2.156534892	0.224294338	down	6.68662E-12	1.05589E-10
G10090_2251	268297	Scml4	-2.15669938	0.224268767	down	0.015679178	0.039325774
G10090_25905	234740	Tmem231	-2.162360975	0.223390389	down	1.24328E-08	1.21403E-07
G10090_8547	271375	Cd200r2	-2.164735547	0.223023007	down	0.000263354	0.001096259
G10090_26254	73420	Ccsap	-2.166294803	0.222782096	down	9.18799E-06	5.39681E-05
G10090_10758	233651	Dchs1	-2.167288063	0.222628768	down	2.4197E-05	0.000128765
G10090_2308	22635	Zan	-2.16800717	0.222517827	down	2.77961E-05	0.000145606
G10090_23161	171210	Acot2	-2.175290769	0.221397254	down	7.99303E-14	1.63807E-12
G10090_9489	108099	Prkag2	-2.176222058	0.221254383	down	3.94082E-17	1.16312E-15
G10090_29195	15586	Hyal1	-2.182323738	0.220320594	down	3.69699E-16	9.92569E-15
G10090_11643	16578	Kif9	-2.187845287	0.219478985	down	6.15116E-09	6.33778E-08
G10090_2660	17260	Mef2c	-2.191132735	0.218979431	down	5.02311E-32	4.75921E-30
G10090_1611	330323	Mindy4	-2.196981061	0.218093539	down	3.02017E-07	2.30389E-06
G10090_9582	620235	Siglec15	-2.199098419	0.217773691	down	0.000218029	0.000924661
G10090_7597	13631	Eef2k	-2.200274181	0.217596283	down	1.13231E-06	7.8209E-06
G10090_4874	83383	Tfap4	-2.202830044	0.217211133	down	2.78738E-06	1.80421E-05
G10090_13188	64297	Gprc5b	-2.204870369	0.216904161	down	8.56817E-09	8.63088E-08
G10090_10774	108797	Mex3b	-2.212524978	0.215756364	down	0.001830856	0.006064972
G10090_18543	227094	Nemp2	-2.214668306	0.215436066	down	7.9458E-07	5.66165E-06
G10090_509	329727	Dennd2c	-2.224000348	0.21404702	down	5.10863E-13	9.39456E-12
G10090_12369	54135	Lsr	-2.233332733	0.212666879	down	6.72768E-14	1.38964E-12
G10090_13338	104111	Adcy3	-2.233869409	0.212587782	down	0.000494517	0.00192206
G10090_26974	320782	Tmem154	-2.239249147	0.211796529	down	6.78904E-06	4.0756E-05
G10090_22201	231605	Galnt9	-2.244976247	0.210957422	down	6.34243E-07	4.5994E-06
G10090_28530	12766	Cxcr3	-2.247757131	0.21055118	down	0.00043937	0.00172671
G10090_14689	277396	Klhl23	-2.251666911	0.209981348	down	0.016443504	0.040999012
G10090_5411	75691	Anks6	-2.260154292	0.208749653	down	1.2886E-06	8.81658E-06
G10090_817	16572	Kif5a	-2.260379362	0.20871709	down	1.57171E-06	1.05765E-05
G10090_2749	332175	Zdhhc23	-2.263834254	0.208217863	down	0.004281731	0.01280504
G10090_17169	52609	Cbx7	-2.269182826	0.207447356	down	3.15382E-11	4.55648E-10
G10090_19259	54712	Plxnc1	-2.276109698	0.206453718	down	1.79412E-20	7.26257E-19
G10090_26619	192678	Rassf3	-2.276378074	0.206415316	down	2.6585E-14	5.78368E-13
G10090_16472	15213	Hey1	-2.276712365	0.206367492	down	0.017569516	0.043392789
G10090_17721	67896	Ccdc80	-2.277321099	0.206280435	down	1.40991E-07	1.14857E-06
G10090_30950	14555	Gpd1	-2.286643176	0.204951837	down	9.19132E-05	0.000432134
G10090_2955	17179	Matk	-2.287534462	0.204825258	down	6.59362E-18	2.07739E-16
G10090_8536	241452	Dhrs9	-2.294049127	0.20390243	down	7.66852E-08	6.52348E-07
G10090_6939	72160	Tmem163	-2.294085247	0.203897325	down	0.01242172	0.032263406
G10090_30608	332397	Nanos1	-2.294848974	0.203789415	down	0.001989919	0.006524119
G10090_2039	53374	Chst3	-2.295102788	0.203753566	down	0.000193798	0.000832426
G10090_1638	51801	Ramp1	-2.296547742	0.203549595	down	0.001898461	0.006262196
G10090_1629	105670	Rcbtb2	-2.304928004	0.202370653	down	3.32407E-22	1.59787E-20
G10090_1683	12337	Capn5	-2.308311245	0.201896633	down	7.40994E-14	1.52575E-12
G10090_2845	22032	Traf4	-2.30974268	0.201696411	down	0.001316454	0.004542792
G10090_95	100042480	Nhsl2	-2.314202589	0.201073855	down	4.3671E-10	5.34143E-09
G10090_25396	56857	Slc37a2	-2.315558477	0.200884969	down	1.15534E-09	1.34157E-08
G10090_19286	67477	Abhd15	-2.322250431	0.19995532	down	1.66701E-10	2.16018E-09

G10090_23594	66329	Susd3	-2.323738292	0.199749211	down	1.08109E-27	7.6407E-26
G10090_24679	239743	Klhl6	-2.328357984	0.199110612	down	1.25419E-09	1.44353E-08
G10090_8371	210789	Tbc1d4	-2.332213157	0.198579258	down	3.74964E-11	5.36983E-10
G10090_22514	13609	S1pr1	-2.332908415	0.198483582	down	6.00741E-14	1.2448E-12
G10090_5792	12495	Entpd1	-2.333010282	0.198469568	down	8.06054E-21	3.37794E-19
G10090_33145	212398	Frat2	-2.335284057	0.198157014	down	7.01183E-07	5.03181E-06
G10090_2995	225049	Ttc7	-2.346120682	0.196674159	down	8.36179E-20	3.169E-18
G10090_22702	114602	Zmynd10	-2.346425681	0.196632584	down	0.003631553	0.011127856
G10090_13764	12322	Camk2a	-2.349051534	0.196275019	down	0.001193699	0.004153169
G10090_32656	235041	Kank2	-2.35138936	0.195957221	down	6.39484E-19	2.20033E-17
G10090_8427	320664	Cass4	-2.35231397	0.195831674	down	1.26307E-08	1.22968E-07
G10090_1974	71085	Arhgap19	-2.35708905	0.195184575	down	1.09882E-06	7.61777E-06
G10090_239	268739	Arhgef40	-2.361384414	0.194604312	down	2.18133E-06	1.4361E-05
G10090_1426	12443	Ccnd1	-2.36169859	0.194561938	down	1.69603E-13	3.3197E-12
G10090_18407	72324	Plxdc1	-2.36229185	0.194481947	down	2.15078E-36	2.67823E-34
G10090_7641	70839	P2ry12	-2.363723577	0.19428904	down	0.000114293	0.000523976
G10090_7458	18019	Nfatc2	-2.372523046	0.193107614	down	1.09842E-14	2.46766E-13
G10090_9343	225288	Fhod3	-2.376940569	0.192517224	down	0.0008327	0.00303782
G10090_9049	213980	Fbxw10	-2.382902261	0.191723321	down	8.90581E-08	7.49314E-07
G10090_24090	381310	Stum	-2.383472856	0.191647508	down	0.001901033	0.006267275
G10090_14120	14788	Gpr162	-2.387529624	0.191109364	down	4.37453E-12	7.09639E-11
G10090_3577	215114	Hip1	-2.389790466	0.190810112	down	1.3946E-16	3.88794E-15
G10090_1456	211586	Tfdp2	-2.390961677	0.190655271	down	9.77814E-17	2.76132E-15
G10090_5267	11877	Arvcf	-2.392069628	0.190508909	down	2.26423E-05	0.000121232
G10090_9638	74760	Rab3il1	-2.394660544	0.190167084	down	1.44934E-16	4.03193E-15
G10090_5998	19260	Ptpn22	-2.397243278	0.189826948	down	3.75457E-14	8.02141E-13
G10090_24166	68738	Acss1	-2.407924349	0.188426744	down	5.37716E-05	0.00026601
G10090_24655	66109	Tspan13	-2.414451927	0.187576119	down	2.51019E-07	1.94206E-06
G10090_32190	67168	Lpar6	-2.420382021	0.186806684	down	3.64781E-19	1.29959E-17
G10090_568	76877	Rab36	-2.425477023	0.186148123	down	0.004348562	0.012984117
G10090_12020	68703	Rere	-2.426707836	0.185989382	down	8.93945E-52	2.54094E-49
G10090_27740	12531	Cdc25b	-2.432449966	0.185250589	down	2.75502E-07	2.12189E-06
G10090_9222	210710	Gab3	-2.434184888	0.185027948	down	0.000361541	0.001447383
G10090_22265	13803	Enc1	-2.434552096	0.184980859	down	1.20902E-13	2.42825E-12
G10090_32621	71837	1700003E16Rik	-2.435205694	0.184897075	down	0.017786514	0.043862442
G10090_11167	329731	Fam19a3	-2.436226719	0.184766266	down	0.010999765	0.028984669
G10090_10733	66775	Hacd4	-2.439851845	0.184302578	down	1.07526E-22	5.34565E-21
G10090_4599	67492	Zfand4	-2.441318087	0.184115362	down	1.1043E-09	1.28721E-08
G10090_200	11891	Rab27a	-2.455368824	0.182330924	down	0.000157651	0.000695208
G10090_29737	230766	Fam167b	-2.456432111	0.182196593	down	0.018102276	0.044523564
G10090_30102	71918	Zcchc24	-2.459802003	0.181771509	down	7.39154E-08	6.3125E-07
G10090_25189	239849	Cd200r4	-2.462863122	0.181386234	down	3.46555E-06	2.19005E-05
G10090_15078	11810	Apobec1	-2.464601576	0.181167795	down	7.28107E-13	1.32039E-11
G10090_24997	19725	Rfx2	-2.466057365	0.180985075	down	2.17557E-11	3.22513E-10
G10090_32056	233781	Xylt1	-2.485884273	0.178514817	down	4.46014E-39	6.78097E-37
G10090_10630	233552	Gdpd5	-2.494874646	0.177405833	down	5.59109E-13	1.02386E-11
G10090_5193	21778	Tex9	-2.506359635	0.17599915	down	4.95989E-10	6.03822E-09
G10090_4473	21961	Tns1	-2.508153465	0.175780451	down	4.81236E-31	4.22293E-29
G10090_1809	235493	Fam214a	-2.509559835	0.175609179	down	3.12756E-13	5.90937E-12
G10090_9117	14605	Tsc22d3	-2.51019345	0.175532071	down	2.05324E-33	2.13064E-31
G10090_8028	330814	Adgrl1	-2.51308056	0.175181149	down	0.00036076	0.001445584
G10090_26117	69987	Spaca9	-2.513668646	0.175109754	down	2.86098E-07	2.19655E-06
G10090_31893	30944	Zfp354c	-2.524441487	0.173807051	down	0.000221464	0.000938925
G10090_3469	19218	Ptger3	-2.535148151	0.172521952	down	1.29693E-08	1.25983E-07
G10090_18597	433586	Maml3	-2.536760023	0.172329307	down	1.01201E-08	1.00395E-07
G10090_19937	11601	Angpt2	-2.537779664	0.172207554	down	7.74962E-06	4.60574E-05
G10090_14696	71706	Slc46a3	-2.547307337	0.171074029	down	6.32909E-05	0.000308664
G10090_4858	16582	Kifc3	-2.553456416	0.170346426	down	5.77671E-32	5.43385E-30
G10090_1829	320277	Spef2	-2.56655196	0.168807164	down	0.001113313	0.00391516
G10090_11185	65962	Slc9a3r2	-2.569722225	0.168436625	down	0.001711202	0.005728103
G10090_213	75729	Fam227a	-2.57657452	0.167638507	down	0.000122926	0.000557884
G10090_9364	217364	Engase	-2.576848202	0.167606708	down	3.19452E-19	1.14748E-17
G10090_17808	629378	Dact3	-2.577301675	0.167554034	down	0.000339121	0.001367255

G10090_9634	140742	Sesn1	-2.587606497	0.1663615	down	9.76061E-19	3.29767E-17
G10090_6275	246049	Slc36a2	-2.592766696	0.165767525	down	7.44386E-09	7.56831E-08
G10090_8564	74011	Slc25a27	-2.599601673	0.164984035	down	2.06018E-07	1.62564E-06
G10090_11428	545554	Ankrd34a	-2.603462898	0.164543062	down	0.000744663	0.002755738
G10090_10166	217946	Cdca7l	-2.604177768	0.16446155	down	1.01739E-07	8.50539E-07
G10090_3345	74309	Osbp2	-2.604617589	0.164411419	down	8.38345E-06	4.95989E-05
G10090_5540	381836	Sbk2	-2.621009452	0.162553953	down	4.6135E-09	4.88829E-08
G10090_6734	17059	Klrb1c	-2.621767047	0.162468615	down	3.10807E-05	0.000161134
G10090_23075	224813	Lrrc73	-2.629975281	0.161546872	down	0.016369719	0.040838405
G10090_22279	66425	Pcp4l1	-2.63250255	0.161264127	down	9.96507E-07	6.98249E-06
G10090_10288	18569	Pdcd4	-2.63914559	0.160523276	down	2.28912E-31	2.03607E-29
G10090_17767	213956	Fam83f	-2.642316505	0.160170848	down	1.07592E-15	2.74759E-14
G10090_11371	72421	Ttc30b	-2.642783354	0.160119026	down	3.07693E-11	4.46228E-10
G10090_15953	23969	Pacsin1	-2.648109011	0.159529042	down	0.002380847	0.007664913
G10090_20955	16170	Il16	-2.648318668	0.15950586	down	5.3772E-13	9.86071E-12
G10090_4565	140481	Man2a2	-2.649383865	0.159388134	down	2.07925E-19	7.67971E-18
G10090_21797	97998	Deptor	-2.652367202	0.159058877	down	5.05761E-11	7.11057E-10
G10090_22737	14456	Gas6	-2.669594688	0.157170821	down	1.34676E-19	5.01678E-18
G10090_13762	230145	Galnt12	-2.680391607	0.155998968	down	1.52983E-09	1.73482E-08
G10090_27587	20342	Selenbp2	-2.682914019	0.155726458	down	0.006028295	0.017254807
G10090_32275	81897	Tlr9	-2.684632666	0.155541055	down	1.5253E-09	1.73119E-08
G10090_11957	252972	Tpcn1	-2.687432256	0.155239516	down	1.2017E-23	6.28489E-22
G10090_24935	13611	S1pr4	-2.693456214	0.154592666	down	6.50314E-05	0.00031668
G10090_14398	226419	Dyrk3	-2.703493429	0.153520857	down	4.33594E-14	9.14394E-13
G10090_4891	217303	Cd300a	-2.713991007	0.152407837	down	9.18778E-28	6.6739E-26
G10090_5690	102103	Mtus1	-2.723008683	0.151458171	down	1.66518E-13	3.26911E-12
G10090_5626	381680	Nxpe5	-2.728471762	0.150885726	down	1.85385E-09	2.08418E-08
G10090_11155	12265	Ciita	-2.732634708	0.150450968	down	1.2495E-08	1.21919E-07
G10090_24096	277154	Nynrin	-2.732645977	0.150449793	down	1.43689E-10	1.8825E-09
G10090_8022	194590	Reps2	-2.73547597	0.150154959	down	1.71196E-12	2.92983E-11
G10090_11728	66790	Grtp1	-2.74041429	0.14964186	down	0.000106455	0.000491837
G10090_14743	20667	Sox12	-2.750579177	0.148591225	down	0.006181475	0.017627651
G10090_5253	21844	Tiam1	-2.762141396	0.147405126	down	4.04562E-41	7.05287E-39
G10090_7570	16174	Il18rap	-2.783991735	0.145189423	down	6.47753E-05	0.00031555
G10090_6194	170706	Tmem37	-2.797946718	0.143791797	down	2.60242E-11	3.82322E-10
G10090_5544	319430	C5ar2	-2.809111831	0.142683278	down	0.002666595	0.008456398
G10090_23867	72480	Tspyl4	-2.814849674	0.142116929	down	2.99074E-10	3.74918E-09
G10090_20713	74229	Paqr8	-2.818586233	0.141749325	down	0.000281803	0.001161975
G10090_11410	16774	Lama3	-2.82026242	0.14158473	down	0.000188032	0.000810591
G10090_7721	84036	Kcnn1	-2.832027051	0.140434855	down	0.005079418	0.014877552
G10090_28414	319734	Cacna2d4	-2.835202104	0.140126128	down	0.008812466	0.023930009
G10090_7850	73873	Fam161a	-2.842357422	0.139432867	down	1.792E-07	1.43305E-06
G10090_23994	69574	Cmbl	-2.845000812	0.139177624	down	0.00034018	0.001371103
G10090_4864	68526	Gpr155	-2.845449142	0.13913438	down	7.60122E-20	2.90602E-18
G10090_11920	668212	Efr3b	-2.850208571	0.138676134	down	8.58871E-13	1.55107E-11
G10090_21445	53883	Celsr2	-2.858099302	0.137919724	down	0.000238892	0.001004023
G10090_11137	107221	Ffar4	-2.869311113	0.136852044	down	0.000357726	0.001434306
G10090_328	208618	Etl4	-2.894666159	0.134467911	down	4.92877E-07	3.63268E-06
G10090_24158	12487	Cd28	-2.896411997	0.134305287	down	1.66595E-25	9.99189E-24
G10090_4126	72500	Ier5l	-2.903868682	0.13361291	down	3.43645E-10	4.26295E-09
G10090_22532	18440	P2rx6	-2.906708272	0.133350185	down	0.000224099	0.000948558
G10090_11258	14357	Dtx1	-2.910668658	0.132984623	down	0.006826536	0.019236414
G10090_6556	72555	Shisa9	-2.91528309	0.132559954	down	0.002627693	0.008357356
G10090_1597	77609	Ccdc151	-2.916268719	0.132469422	down	0.009610365	0.025817858
G10090_18295	30951	Cbx8	-2.920560844	0.132075901	down	1.70802E-12	2.92735E-11
G10090_3913	236899	Pcyt1b	-2.924546968	0.131711483	down	1.51978E-07	1.23118E-06
G10090_22691	73910	Arhgap18	-2.927641683	0.131429252	down	1.12101E-31	1.02498E-29
G10090_15229	237868	Sarm1	-2.927896761	0.131406017	down	0.001112384	0.003912945
G10090_31850	240055	Neurl1b	-2.935090286	0.130752435	down	3.11977E-15	7.58196E-14
G10090_17399	76166	Rsg1	-2.949691962	0.129435749	down	0.009845926	0.026353481
G10090_9212	78286	Nav2	-2.950569074	0.12935708	down	8.90811E-13	1.59991E-11
G10090_27882	16574	Kif5c	-2.961331376	0.128395685	down	2.20581E-05	0.000118598
G10090_16461	213573	Cracr2b	-2.966217099	0.127961605	down	4.21749E-10	5.17295E-09

G10090_4946	27029	Sgsh	-2.968896599	0.127724164	down	3.63356E-14	7.77558E-13
G10090_23706	17385	Mmp11	-2.975269457	0.127161209	down	0.001493023	0.005073096
G10090_28365	170733	Klra17	-2.98277982	0.126500955	down	0.002736925	0.008647969
G10090_24512	230991	Fndc10	-2.988052352	0.126039483	down	2.75577E-05	0.00014459
G10090_22957	67217	L3hypdh	-2.992401241	0.125660119	down	0.016852078	0.041866032
G10090_2041	104086	Cyp27a1	-3.000276904	0.12497601	down	1.46165E-31	1.30897E-29
G10090_25498	107771	Bmyc	-3.000644096	0.124944206	down	1.74293E-05	9.61552E-05
G10090_354	29811	Ndrg2	-3.015006584	0.123706517	down	5.77557E-08	5.03101E-07
G10090_15516	18143	Npas2	-3.022762061	0.123043294	down	5.20926E-07	3.82217E-06
G10090_9189	546611	Klhl33	-3.027582373	0.12263287	down	8.57706E-05	0.000405295
G10090_11232	69993	Chn2	-3.042254967	0.121391981	down	0.003765145	0.011483384
G10090_7010	214642	Cped1	-3.045370339	0.121130129	down	0.000230517	0.000972888
G10090_14680	64051	Sv2a	-3.06353608	0.119614477	down	3.24053E-08	2.9485E-07
G10090_395	213556	Plekhh2	-3.063783778	0.119593942	down	2.01165E-05	0.000109048
G10090_1879	27355	Pald1	-3.064347825	0.119547194	down	1.01486E-16	2.8536E-15
G10090_9105	243219	2900026A02Rik	-3.090521153	0.117397928	down	3.73016E-15	8.98192E-14
G10090_3827	79221	Hdac9	-3.097466903	0.116834082	down	3.04343E-07	2.31758E-06
G10090_3270	231125	Zfyve28	-3.129086702	0.114301267	down	6.30798E-36	7.49789E-34
G10090_107	330908	Opcml	-3.147141049	0.112879777	down	1.8225E-10	2.35002E-09
G10090_15915	320495	Ipcef1	-3.169867427	0.111115545	down	0.007021882	0.019689278
G10090_9340	18027	Nfia	-3.201532586	0.108703283	down	2.31831E-05	0.000123823
G10090_1226	140571	Plxnb3	-3.204877278	0.108451561	down	9.00298E-10	1.06336E-08
G10090_7427	14537	Gcnt1	-3.214942715	0.107697546	down	7.57167E-16	1.97999E-14
G10090_12611	13170	Dbp	-3.228353016	0.106701102	down	6.02478E-11	8.38022E-10
G10090_12551	227929	Cytip	-3.258509576	0.104493885	down	5.82978E-22	2.7223E-20
G10090_18091	269642	Nat8l	-3.274755312	0.103323813	down	0.004025046	0.012168204
G10090_6588	224454	Zdhhc14	-3.280657467	0.102901972	down	4.25618E-33	4.28073E-31
G10090_6337	232288	Frmd4b	-3.288782911	0.102324044	down	1.29329E-31	1.17429E-29
G10090_13093	171543	Bmf	-3.290099068	0.102230737	down	9.50542E-51	2.43693E-48
G10090_1232	19331	Rab19	-3.293742608	0.101972878	down	1.18052E-10	1.56386E-09
G10090_9646	237847	Rtn4rl1	-3.29398905	0.10195546	down	0.009691773	0.0260045
G10090_23423	320472	Ppm1e	-3.297174077	0.101730622	down	6.17606E-06	3.74025E-05
G10090_12925	319660	Agmo	-3.308333223	0.100946779	down	3.06712E-12	5.08271E-11
G10090_18507	12772	Ccr2	-3.309586114	0.100859151	down	3.28339E-19	1.17617E-17
G10090_20779	20452	St8sia4	-3.326341931	0.099694524	down	3.77828E-77	2.60006E-74
G10090_6907	245269	Nim1k	-3.337758753	0.098908701	down	2.1964E-07	1.7248E-06
G10090_9703	19735	Rgs2	-3.349196058	0.098127679	down	9.24932E-18	2.84553E-16
G10090_6598	56229	Thsd1	-3.35221339	0.097922663	down	3.30409E-10	4.10655E-09
G10090_13177	17444	Grap2	-3.354869697	0.097742533	down	0.001034709	0.003683317
G10090_33386	630146	Cd101	-3.36031749	0.097374141	down	1.44931E-05	8.14694E-05
G10090_5873	74694	Tbc1d30	-3.366283489	0.0969723	down	4.63936E-13	8.61643E-12
G10090_15406	218215	Rnf144b	-3.375290937	0.096368741	down	1.65245E-24	9.11637E-23
G10090_25410	244556	Zfp791	-3.440969935	0.092079899	down	4.44031E-05	0.000223125
G10090_13126	230603	Ttc39a	-3.442328917	0.091993203	down	2.29399E-12	3.8802E-11
G10090_3924	259302	Srgap3	-3.445526633	0.091789527	down	1.20711E-18	4.02627E-17
G10090_22616	231871	Daglb	-3.449542805	0.091534359	down	3.22684E-26	2.0481E-24
G10090_1890	16880	Lifr	-3.450124032	0.091497489	down	2.75562E-07	2.12189E-06
G10090_10416	170835	Inpp5j	-3.486212745	0.089237089	down	0.00042118	0.001664732
G10090_12919	330812	Rnf150	-3.498239688	0.088496261	down	9.76404E-12	1.50904E-10
G10090_91	68507	Ppfia4	-3.527451476	0.086722402	down	0.013039144	0.033626589
G10090_1453	26874	Abcd2	-3.537979612	0.086091845	down	1.93592E-24	1.06354E-22
G10090_9164	16658	Mafb	-3.56117295	0.084718864	down	7.21966E-31	6.21033E-29
G10090_14495	72948	Tppp	-3.57231926	0.084066845	down	0.000141828	0.000633549
G10090_8359	13116	Cyp46a1	-3.591053633	0.082982237	down	0.003066946	0.009563635
G10090_1229	70737	Cgn	-3.594503562	0.082784038	down	0.000352334	0.001414855
G10090_15145	383787	Ankrd63	-3.598202395	0.082572066	down	0.009768928	0.02618365
G10090_25085	381810	Lpar5	-3.598630198	0.082547584	down	9.45088E-40	1.4888E-37
G10090_10042	76184	Abca6	-3.654063577	0.07943598	down	0.000150365	0.000666676
G10090_15509	225872	Npas4	-3.663190628	0.078935023	down	1.1294E-06	7.80904E-06
G10090_28737	17427	Mns1	-3.667354369	0.078707538	down	0.008228428	0.022561898
G10090_4167	57442	Kcne3	-3.676238906	0.078224324	down	0.001101188	0.003879825
G10090_78	58235	Nectin1	-3.690656654	0.077446473	down	9.87227E-27	6.454E-25
G10090_24308	17057	Klrb1a	-3.71683115	0.076054048	down	0.001712512	0.005731021

G10090_21897	71326	Treml1	-3.725609539	0.075592686	down	6.92718E-09	7.08153E-08
G10090_10384	226841	Vash2	-3.760273172	0.073798066	down	1.30518E-09	1.49695E-08
G10090_5821	110197	Dgkg	-3.761573022	0.073731605	down	3.26728E-18	1.05221E-16
G10090_22465	207474	Kctd12b	-3.776920554	0.072951399	down	7.13591E-17	2.05965E-15
G10090_2641	56226	Espn	-3.786972351	0.072444885	down	0.006064836	0.017337759
G10090_277	224022	Slc7a4	-3.822143008	0.070700145	down	1.13598E-24	6.3204E-23
G10090_3571	18125	Nos1	-3.839768734	0.069841641	down	0.010137296	0.026983946
G10090_12380	12802	Cnr2	-3.842689254	0.0697004	down	4.00499E-20	1.5965E-18
G10090_26459	321019	Gpr183	-3.858007021	0.068964273	down	1.98877E-28	1.53865E-26
G10090_7502	53791	Tlr5	-3.960190408	0.064248634	down	0.002818161	0.008870354
G10090_4072	73667	2410004P03Rik	-4.008588676	0.062129029	down	0.003444874	0.010598053
G10090_11259	26399	Map2k6	-4.011059792	0.062022703	down	1.12842E-08	1.11017E-07
G10090_8306	108723	Card11	-4.014480787	0.061875806	down	6.77642E-05	0.000328397
G10090_33796	16627	Klra1	-4.037737583	0.060886341	down	1.60099E-05	8.92663E-05
G10090_22168	243548	Prickle2	-4.038762589	0.060843097	down	0.017289781	0.042823241
G10090_19329	26904	Sh2d1b1	-4.045869134	0.060544129	down	0.007451649	0.020725445
G10090_13328	333050	Ksr2	-4.092114447	0.058634173	down	0.000652472	0.002453571
G10090_18455	68169	Ndnf	-4.122063	0.057429547	down	0.00418082	0.012543418
G10090_25918	654812	Angptl7	-4.138607836	0.056774707	down	5.15352E-05	0.000255623
G10090_14724	53881	Slc5a3	-4.141806673	0.056648962	down	1.37063E-45	3.08982E-43
G10090_33681	432779	Lrrc14b	-4.147952631	0.056408148	down	9.8178E-16	2.51701E-14
G10090_13929	214239	Ccdc9b	-4.166252624	0.055697152	down	0.01712782	0.042454264
G10090_10740	215512	Fam117a	-4.201156458	0.054365813	down	9.40449E-18	2.88647E-16
G10090_23282	20981	Syt3	-4.226089668	0.053434315	down	0.011399031	0.02990416
G10090_32965	26897	Acot1	-4.32265154	0.049974934	down	8.31097E-11	1.13312E-09
G10090_12917	380912	Zfp395	-4.451867138	0.045693503	down	3.83465E-21	1.63316E-19
G10090_2864	433940	Fam222a	-4.51337684	0.043786294	down	0.004086528	0.012324871
G10090_16576	240168	Rasgrp3	-4.652378274	0.039764414	down	1.48042E-12	2.574E-11
G10090_26818	68918	1190005I06Rik	-4.691741558	0.038694128	down	0.002203115	0.007155493
G10090_13256	17082	Il1rl1	-4.694019615	0.038633077	down	0.000430312	0.001694166
G10090_31489	71781	Slc16a14	-4.709296781	0.038226137	down	0.017341786	0.04292765
G10090_3015	16512	Kcnh3	-4.743845112	0.037321606	down	0.00317118	0.009855759
G10090_13108	110862	Kcnq3	-4.782030577	0.036346731	down	0.011097035	0.029193909
G10090_3845	23890	Gpr34	-4.791912433	0.036098622	down	0.001431279	0.004893821
G10090_15354	71751	Map3k13	-4.803877216	0.035800481	down	0.010343147	0.027453643
G10090_1406	270152	Jaml	-4.853580878	0.034588089	down	0.000249352	0.001042955
G10090_16513	68515	Myadml2	-4.874945808	0.034079647	down	0.002438792	0.00782508
G10090_22356	269152	Kif26b	-4.887430967	0.033785992	down	1.40656E-07	1.14786E-06
G10090_6719	100503041	Pdzd7	-4.940970639	0.032555145	down	0.008899468	0.024136185
G10090_29148	240638	Slc16a12	-5.107571041	0.029004669	down	2.58266E-05	0.000136548
G10090_8657	74511	Lrrc17	-5.202926129	0.027149583	down	0.005705484	0.016449659
G10090_10147	20440	St6gal1	-5.20296934	0.02714877	down	8.55185E-75	5.59077E-72
G10090_25300	102631503	Gm29825	-5.233991961	0.026571216	down	0.002378857	0.007660974
G10090_3771	54167	Icos	-5.254794641	0.026190826	down	0.000870987	0.003162499
G10090_6461	70355	Gprc5c	-5.256303388	0.02616345	down	1.23068E-19	4.59747E-18
G10090_28649	22342	Lin7b	-5.273950105	0.025845374	down	0.000199369	0.000853831
G10090_9160	241525	Ypel4	-5.363406995	0.02429146	down	2.75983E-10	3.47973E-09
G10090_21186	57875	Angptl4	-5.535548716	0.021559257	down	6.18298E-17	1.80452E-15
G10090_3779	666528	Zfp541	-5.540491784	0.021485516	down	9.4062E-05	0.000441126
G10090_27139	208666	Diras1	-5.647322009	0.019952011	down	0.000510072	0.001975218
G10090_13464	319555	Nwd1	-5.87364521	0.017055192	down	2.49337E-05	0.000132416
G10090_29945	63828	Fn3k	-6.040181443	0.015195822	down	9.45735E-05	0.000443366
G10090_18185	225579	Slc27a6	-6.07012542	0.014883675	down	8.33139E-12	1.30303E-10
G10090_14602	20677	Sox4	-6.297663592	0.012712014	down	2.47752E-12	4.1637E-11
G10090_16222	15446	Hpgd	-7.618103425	0.005090054	down	3.68551E-09	3.95308E-08
