# Supplementary material for: Guanylate-Binding protein 2b regulates the AMPK/mTOR/ULK1 signalling pathway to induce autophagy during Mycobacterium bovis infection
Source: Virulence. 2022 May 21;13(1):875–89. doi: 10.1080/21505594.2022.2073024 (PMC9132469; doi:10.1080/21505594.2022.2073024)
Supplement: Supplemental Material [file KVIR_A_2073024_SM9990.zip › Supplementary table 2 .pdf]

st_gene_id	gene_id	gene_symbol	log2FoldChange	foldchange	style	pvalue	padj
G10090_998	22241	Ulk1	-0.717930367	0.607968985	down	4.47696E-11	5.36077E-10
G10090_11698	56480	Tbk1	0.449190339	1.36527383	up	1.81297E-07	1.36104E-06
G10090_3214	105787	Prkaa1	0.501346354	1.415533952	up	3.11729E-07	2.25814E-06
G10090_3428	18033	Nfkb1	0.675852789	1.597540814	up	5.13839E-07	3.61303E-06
G10090_9345	72512	Tmem173	-0.366831676	0.775483679	down	0.021992016	0.054135772
G10090_25693	56717	Mtor	0.025307883	1.017696854	up	0.762473981	0.848018819
G10090_9187	11302	Aatk	-1.541295843	0.343576712	down	1.61293E-20	4.84349E-19
G10090_21279	11303	Abca1	1.108992054	2.156948984	up	6.5363E-12	8.67821E-11
G10090_4334	11433	Acp5	1.076051171	2.108257623	up	1.26789E-05	7.03258E-05
G10090_15182	11475	Acta2	1.003226998	2.004478576	up	0.003940877	0.012264871
G10090_28845	11479	Acvr1b	1.174847869	2.257690725	up	5.70536E-24	2.25909E-22
G10090_25429	11481	Acvr2b	-1.044975783	0.484653044	down	7.35302E-09	6.74638E-08
G10090_12587	11486	Ada	-1.251397344	0.420041173	down	4.8643E-08	4.00958E-07
G10090_2930	11496	Adam22	-2.141890032	0.226582755	down	7.01011E-07	4.823E-06
G10090_1526	11520	Plin2	-1.392739747	0.38084088	down	7.10356E-24	2.79622E-22
G10090_315	11529	Adh7	3.17074303	9.005104577	up	8.1154E-16	1.57874E-14
G10090_33977	11535	Adm	6.628147972	98.91709542	up	1.76886E-06	1.12688E-05
G10090_18252	11540	Adora2a	4.286643069	19.516779	up	2.12864E-48	4.2646E-46
G10090_31525	11541	Adora2b	5.044388306	32.99986702	up	9.8215E-63	4.39447E-60
G10090_2760	11549	Adra1a	-2.638782403	0.160563692	down	0.004075514	0.012619522
G10090_25406	11565	Adssl1	-1.228257317	0.426832721	down	2.35966E-25	1.03848E-23
G10090_1011	11576	Afp	2.178108633	4.525598609	up	1.50194E-15	2.8236E-14
G10090_7097	11600	Angpt1	4.494903934	22.54763063	up	0.008900059	0.024893831
G10090_19937	11601	Angpt2	-2.31141387	0.201462905	down	3.46834E-05	0.000177489
G10090_20553	11610	Agtrap	1.025639091	2.035861039	up	1.41997E-08	1.24746E-07
G10090_22453	11622	Ahr	2.32583852	5.013570883	up	1.61808E-28	9.28184E-27
G10090_24701	11629	Aif1	-1.639237652	0.321026066	down	2.0852E-05	0.000111291
G10090_9198	11630	Crybg1	-1.015586029	0.494627368	down	9.47029E-14	1.5026E-12
G10090_13883	11637	Ak2	1.097280379	2.139509936	up	1.01056E-37	1.1304E-35
G10090_9456	11639	Ak4	4.417284622	21.36658774	up	1.38052E-29	8.69909E-28
G10090_24063	11655	Alas1	1.607732084	3.047723631	up	1.80614E-31	1.29646E-29
G10090_207	11670	Aldh3a1	4.149998712	17.7530957	up	0.008875553	0.024830461
G10090_19593	11684	Alox12	5.740727445	53.47258235	up	8.88543E-05	0.000418341
G10090_22886	11689	Alox5	-1.705466157	0.306622154	down	1.1225E-10	1.28232E-09
G10090_27664	11699	Ambp	2.143405317	4.418036437	up	0.016100376	0.041528699
G10090_3477	11717	Ampd3	1.054458242	2.076938132	up	3.21605E-12	4.46885E-11
G10090_16116	11727	Ang	-2.209565149	0.216199464	down	0.001585413	0.005462269
G10090_32801	11732	Ank	-2.065942949	0.238830178	down	2.15584E-25	9.58208E-24
G10090_20137	11758	Prdx6	1.953139246	3.872161822	up	0.000112355	0.000516074
G10090_5572	11764	Ap1b1	-1.047138256	0.483927136	down	3.50181E-14	5.79591E-13
G10090_19792	11770	Fabp4	-3.022454806	0.123069502	down	8.44034E-11	9.84315E-10
G10090_8735	11796	Birc3	1.18875236	2.279555224	up	3.32515E-15	6.0561E-14
G10090_15078	11810	Apobec1	-2.796322295	0.143953793	down	1.19376E-18	3.0697E-17
G10090_8852	11813	Apoc2	-1.381583622	0.383797276	down	0.012209902	0.032717173
G10090_29857	11828	Aqp3	4.74041052	26.7304185	up	0.003385364	0.010750354
G10090_30906	11839	Areg	5.466018516	44.20134964	up	0.000180081	0.000787884
G10090_32788	11846	Arg1	5.400622807	42.24248516	up	4.50226E-06	2.68356E-05
G10090_31218	11847	Arg2	3.08254536	8.471076747	up	8.61302E-15	1.48985E-13
G10090_29218	11854	Rhod	-1.346778429	0.393169025	down	5.10707E-05	0.000253334
G10090_23754	11858	Rnd2	-1.073945942	0.47501799	down	0.000102757	0.000475786
G10090_2841	11864	Arnt2	1.769800617	3.410068257	up	3.42427E-05	0.000175368
G10090_69	11876	Artn	6.271071703	77.22904912	up	1.6494E-25	7.42951E-24
G10090_5267	11877	Arvcf	-1.755821742	0.296104487	down	0.002331838	0.007717026
G10090_12001	11881	Arsb	-1.272553551	0.41392648	down	9.14024E-05	0.000428984
G10090_22410	11898	Ass1	1.990389259	3.973441926	up	2.31314E-16	4.7476E-15
G10090_12280	11911	Atf4	1.038619098	2.054260438	up	2.11888E-28	1.20516E-26
G10090_14606	11936	Fxyd2	-1.686834845	0.310607626	down	9.35951E-08	7.36416E-07
G10090_6674	11973	Atp6v1e1	1.095337816	2.136631063	up	9.61472E-41	1.18402E-38

G10090_6718	11980	Atp8a1	-1.549283487	0.341679717	down	9.76688E-15	1.68294E-13
G10090_22529	11987	Slc7a1	1.594416475	3.01972354	up	5.60712E-23	2.0508E-21
G10090_7431	11988	Slc7a2	2.608339105	6.098012489	up	5.92434E-38	6.68256E-36
G10090_10237	12013	Bach1	-1.271653611	0.414184764	down	2.22311E-21	7.20791E-20
G10090_17364	12014	Bach2	-2.758985696	0.147727908	down	0.004444313	0.013616034
G10090_25470	12029	Bcl6b	5.594349912	48.31334752	up	0.000166903	0.000736712
G10090_25291	12035	Bcat1	3.945936988	15.41151716	up	2.79766E-05	0.000146064
G10090_5801	12040	Bckdhb	2.614954438	6.126038518	up	3.46656E-56	1.03404E-53
G10090_32612	12044	Bcl2a1a	6.360085564	82.14412917	up	4.30804E-51	9.17886E-49
G10090_24644	12045	Bcl2a1b	2.989209065	7.940385576	up	2.34521E-23	8.79322E-22
G10090_9096	12046	Bcl2a1c	3.850248362	14.42249004	up	8.27165E-11	9.65482E-10
G10090_25587	12047	Bcl2a1d	3.691557942	12.92021293	up	1.27771E-25	5.79415E-24
G10090_30321	12051	Bcl3	1.244672301	2.36964723	up	4.36515E-16	8.7063E-15
G10090_24510	12053	Bcl6	-1.475001529	0.359733014	down	9.64907E-10	9.96303E-09
G10090_15065	12062	Bdkrb2	5.357824215	41.00773668	up	0.003620537	0.011362748
G10090_2385	12121	Bicd1	-2.16656738	0.222740008	down	0.00197908	0.006656273
G10090_10915	12140	Fabp7	-1.808180466	0.28555084	down	2.61228E-09	2.56321E-08
G10090_361	12153	Bmp1	3.087231136	8.498634962	up	0.006833904	0.019773979
G10090_5815	12166	Bmpr1a	1.182879221	2.270294133	up	0.000130782	0.000590674
G10090_29587	12176	Bnip3	1.793777883	3.467216401	up	1.44837E-26	7.06962E-25
G10090_10160	12182	Bst1	3.512269998	11.41034097	up	1.86507E-32	1.4471E-30
G10090_2707	12190	Brca2	-1.296123757	0.407218848	down	2.19503E-07	1.62784E-06
G10090_13429	12193	Zfp36l2	-1.194394444	0.436969823	down	2.27826E-24	9.40956E-23
G10090_28052	12217	Bsn	-1.67346028	0.313500515	down	0.000352695	0.001432443
G10090_29130	12228	Btg3	1.501682351	2.831727325	up	2.95444E-12	4.11812E-11
G10090_31816	12257	Tspo	1.015632294	2.021788794	up	5.88482E-28	3.29133E-26
G10090_31441	12259	C1qa	-3.780778905	0.072756558	down	1.29133E-19	3.6187E-18
G10090_2549	12260	C1qb	-4.303313326	0.050649319	down	5.24842E-19	1.38954E-17
G10090_10347	12262	C1qc	-3.911177764	0.066468852	down	5.24405E-24	2.10122E-22
G10090_11155	12265	Ciita	-2.422070914	0.186588126	down	4.27189E-08	3.55717E-07
G10090_19718	12266	C3	1.401945676	2.642577305	up	7.6012E-12	9.99323E-11
G10090_15255	12267	C3ar1	-1.847262872	0.277919145	down	3.69639E-10	4.02405E-09
G10090_11453	12282	Hyou1	1.616074122	3.065397409	up	5.27791E-73	3.22025E-70
G10090_5144	12286	Cacna1a	-1.42514126	0.372382902	down	8.75877E-20	2.49616E-18
G10090_2374	12304	Pdia4	1.807627712	3.500661855	up	1.4524E-36	1.48822E-34
G10090_33333	12308	Calb2	4.080890376	16.92272948	up	0.001196953	0.004259463
G10090_25394	12311	Calcr	7.882163073	235.9214972	up	4.3426E-10	4.70086E-09
G10090_15941	12316	Aspm	-2.174848188	0.221465183	down	0.000359484	0.00145737
G10090_16568	12317	Calr	1.268869207	2.409726158	up	2.44936E-41	3.19201E-39
G10090_16149	12321	Calu	1.076284844	2.108599125	up	1.33664E-20	4.03762E-19
G10090_1683	12337	Capn5	-1.572854793	0.336142579	down	1.86476E-14	3.1485E-13
G10090_20416	12349	Car2	4.494926476	22.54798295	up	3.74968E-24	1.51602E-22
G10090_9705	12363	Casp4	1.28883017	2.443298568	up	1.49959E-23	5.70226E-22
G10090_17190	12369	Casp7	1.360321595	2.567424043	up	1.4821E-07	1.12907E-06
G10090_5443	12393	Runx2	-1.244096397	0.422172234	down	3.89476E-11	4.70561E-10
G10090_2243	12398	Cbfa2t3	-2.493312673	0.177598011	down	0.01313865	0.03488135
G10090_29906	12399	Runx3	1.458171865	2.747599762	up	5.83848E-08	4.73535E-07
G10090_24451	12409	Cbr2	-4.779998732	0.036397957	down	3.59644E-05	0.000183486
G10090_11692	12428	Ccna2	-1.633731868	0.322253544	down	0.002398912	0.00791753
G10090_10985	12442	Ccnb2	-1.710217449	0.305614003	down	0.003565535	0.011213724
G10090_1426	12443	Ccnd1	-2.434198673	0.185026181	down	9.14682E-11	1.06117E-09
G10090_16069	12444	Ccnd2	2.549487405	5.854262371	up	0.000147724	0.000659161
G10090_22628	12462	Cct3	1.614435781	3.061918285	up	8.92092E-44	1.39239E-41
G10090_4698	12475	Cd14	2.485995291	5.602206996	up	3.11198E-27	1.5763E-25
G10090_13831	12479	Cd1d1	3.533802207	11.58191739	up	2.96081E-43	4.51624E-41
G10090_29911	12481	Cd2	-5.795525854	0.018004161	down	0.00080117	0.002959303
G10090_1279	12483	Cd22	-1.457744008	0.364061981	down	0.019774753	0.049503266
G10090_15689	12484	Cd24a	1.095746947	2.137237072	up	6.03262E-05	0.000293817
G10090_24158	12487	Cd28	-2.505517969	0.176101857	down	1.02509E-62	4.43865E-60

G10090_12399	12489	Cd33	1.509963776	2.84802888	up	3.04369E-10	3.3297E-09
G10090_12400	12490	Cd34	-3.052412053	0.12054034	down	0.000536314	0.002087859
G10090_21977	12494	Cd38	6.831720019	113.9075859	up	2.04117E-27	1.06609E-25
G10090_5792	12495	Entpd1	-2.186165351	0.219734704	down	3.28161E-41	4.19515E-39
G10090_8848	12503	Cd247	5.528921299	46.17119921	up	9.72192E-20	2.75893E-18
G10090_18243	12504	Cd4	-2.682107949	0.15581349	down	9.09356E-08	7.18017E-07
G10090_15032	12508	Cd53	1.247488376	2.374277192	up	1.73976E-22	6.12936E-21
G10090_6724	12515	Cd69	2.989190826	7.940285195	up	9.73151E-07	6.53457E-06
G10090_9162	12522	Cd83	1.101476279	2.145741494	up	0.00986492	0.02723505
G10090_1183	12524	Cd86	1.143667103	2.209419095	up	0.000155064	0.000689215
G10090_27740	12531	Cdc25b	-2.082764047	0.23606171	down	5.4946E-16	1.08782E-14
G10090_19713	12562	Cdh5	2.08585708	4.245272259	up	0.000159764	0.000708463
G10090_10825	12563	Cdh6	5.247644229	37.99253913	up	6.29211E-05	0.000305016
G10090_32887	12579	Cdkn2b	3.150348182	8.878698313	up	1.88975E-17	4.41917E-16
G10090_18165	12580	Cdkn2c	-1.673264218	0.313543123	down	9.89845E-12	1.2788E-10
G10090_8520	12606	Cebpa	-1.69881419	0.308039189	down	1.63344E-14	2.76839E-13
G10090_29960	12608	Cebpb	1.378680445	2.600304265	up	2.02631E-38	2.36514E-36
G10090_10939	12615	Cenpa	-2.428685139	0.185734646	down	1.42969E-06	9.28882E-06
G10090_8010	12628	Cfh	-2.475394513	0.179817519	down	3.25591E-17	7.35759E-16
G10090_2328	12633	Cflar	1.222644005	2.333740267	up	3.05277E-17	6.9453E-16
G10090_30130	12642	Ch25h	3.377568947	10.39320672	up	0.002480734	0.008155167
G10090_1599	12654	Chil1	4.812077609	28.09180868	up	1.84389E-44	3.01835E-42
G10090_25739	12700	Cish	7.178796589	144.8882297	up	5.56069E-31	3.82775E-29
G10090_21609	12702	Socs3	4.839291522	28.62674082	up	4.9649E-234	6.6643E-230
G10090_18702	12703	Socs1	6.364196043	82.37850521	up	2.1893E-102	3.2653E-99
G10090_26889	12709	Ckb	-1.69681132	0.308467132	down	3.47243E-09	3.32694E-08
G10090_28530	12766	Cxcr3	-2.818189504	0.14178831	down	2.31033E-08	1.98447E-07
G10090_18442	12767	Cxcr4	-2.296349848	0.203577518	down	1.53556E-18	3.90374E-17
G10090_24748	12768	Ccr1	1.933214054	3.819050651	up	1.29522E-10	1.46468E-09
G10090_15355	12769	Ccr9	1.555903394	2.940177786	up	0.003450303	0.010914187
G10090_18507	12772	Ccr2	-3.653815368	0.079449648	down	3.40739E-21	1.08382E-19
G10090_26041	12774	Ccr5	-1.411963276	0.375799935	down	4.95026E-06	2.93236E-05
G10090_5516	12775	Ccr7	3.712194722	13.10635603	up	0.010040916	0.027669723
G10090_22012	12795	Plk3	1.539451179	2.906839022	up	3.95633E-16	7.91443E-15
G10090_5539	12796	Camp	3.101054068	8.580454497	up	1.21389E-07	9.38059E-07
G10090_12380	12802	Cnr2	-1.863195545	0.274866779	down	1.53831E-14	2.61376E-13
G10090_15062	12819	Col15a1	-1.792858193	0.28859972	down	0.00247966	0.008153964
G10090_18890	12823	Col19a1	2.659542602	6.318326989	up	6.80234E-05	0.000327034
G10090_19357	12827	Col4a2	1.617453292	3.068329231	up	0.012335191	0.032956862
G10090_18775	12830	Col4a5	-1.666549184	0.315005913	down	1.50926E-07	1.14716E-06
G10090_14746	12831	Col5a1	4.092688422	17.06168729	up	6.25949E-07	4.33769E-06
G10090_14858	12850	Coq7	1.055760063	2.07881311	up	1.03926E-11	1.33749E-10
G10090_9692	12856	Cox17	1.561693702	2.952002007	up	5.40785E-17	1.19195E-15
G10090_14539	12874	Cpd	1.271165925	2.413565409	up	9.10152E-21	2.80206E-19
G10090_30625	12890	Cplx2	3.731536488	13.28325202	up	9.95335E-07	6.66353E-06
G10090_8462	12894	Cpt1a	-1.048024156	0.483630067	down	1.27807E-12	1.8427E-11
G10090_4834	12916	Crem	1.529392304	2.886642215	up	1.19464E-09	1.21944E-08
G10090_23906	12925	Crip1	-2.413038993	0.187759916	down	1.76124E-13	2.73307E-12
G10090_12053	12927	Bcar1	2.217199415	4.649899112	up	1.10391E-05	6.21029E-05
G10090_16982	12977	Csf1	1.441058018	2.715199149	up	0.000948098	0.003457299
G10090_7545	12981	Csf2	8.506842222	363.7597789	up	8.05387E-11	9.4088E-10
G10090_11973	12983	Csf2rb	1.085923286	2.12273354	up	6.59815E-17	1.43777E-15
G10090_27315	12985	Csf3	10.04812996	1058.738039	up	4.40062E-44	7.03209E-42
G10090_13048	13003	Vcan	1.611612698	3.055932545	up	0.001081059	0.00389663
G10090_12417	13008	Csrp2	1.419988515	2.675833808	up	0.006540631	0.019031914
G10090_18200	13011	Cst7	2.017620099	4.049152838	up	3.10324E-05	0.000160211
G10090_2670	13025	Ctla2b	1.547498502	2.923098616	up	1.11303E-05	6.25484E-05
G10090_14676	13026	Pcyt1a	-1.245464491	0.421772082	down	2.22476E-26	1.07808E-24
G10090_2423	13032	Ctsc	1.490101245	2.80908688	up	6.10524E-30	3.95897E-28

G10090_21400	13034	Ctse	-2.128281796	0.228730111	down	7.50351E-25	3.15736E-23
G10090_31148	13051	Cx3cr1	-3.912639036	0.066401561	down	8.51377E-07	5.77173E-06
G10090_18909	13058	Cybb	1.15292236	2.223638644	up	1.45121E-57	5.26476E-55
G10090_26895	13063	Cycs	1.175813721	2.259202705	up	7.03768E-16	1.37971E-14
G10090_12496	13078	Cyp1b1	4.800435856	27.86603543	up	1.71629E-11	2.16549E-10
G10090_12904	13086	Cyp2a4	-2.959936278	0.128519905	down	0.011398541	0.030864354
G10090_9741	13139	Dgka	-1.484863451	0.35728235	down	1.2913E-08	1.14184E-07
G10090_12611	13170	Dbp	-3.724619782	0.075644564	down	2.6896E-41	3.4714E-39
G10090_5952	13179	Dcn	3.265123817	9.613913462	up	3.53134E-13	5.35001E-12
G10090_33079	13197	Gadd45a	1.027904481	2.039060358	up	0.009169265	0.02554037
G10090_178	13360	Dhcr7	-1.639046422	0.321068621	down	1.51716E-15	2.84822E-14
G10090_12882	13401	Dmwd	2.623429329	6.1621309	up	2.31223E-08	1.98447E-07
G10090_24395	13418	Dnajc1	1.373128487	2.590316682	up	2.5547E-25	1.12064E-23
G10090_3807	13518	Dst	1.575778703	2.980963485	up	3.29409E-18	8.05402E-17
G10090_1998	13527	Dtna	4.557342679	23.54489982	up	0.012294909	0.032875412
G10090_8194	13528	Dtnb	1.624830937	3.084060223	up	1.60443E-12	2.29353E-11
G10090_19379	13603	Opn3	1.241333626	2.364169749	up	0.002103581	0.007032719
G10090_16431	13605	Ect2	-1.502825963	0.352861525	down	0.001054333	0.003811556
G10090_23029	13610	S1pr3	5.394815925	42.07279986	up	0.001751102	0.005964234
G10090_24935	13611	S1pr4	-2.453742654	0.182536559	down	2.86669E-05	0.000149204
G10090_23818	13612	Edil3	1.521714716	2.871321178	up	0.000182381	0.000796387
G10090_21260	13614	Edn1	9.526437926	737.4686578	up	2.16692E-19	5.98365E-18
G10090_1236	13618	Ednrb	3.078013252	8.444507306	up	6.25466E-12	8.35386E-11
G10090_22602	13628	Eef1a2	-1.716789385	0.304224999	down	5.58645E-18	1.34869E-16
G10090_7597	13631	Eef2k	-1.967338008	0.255724447	down	6.78751E-05	0.000326672
G10090_18847	13637	Efna2	2.572366552	5.947842942	up	0.000799976	0.00295652
G10090_8150	13642	Efnb2	2.309204385	4.956096867	up	0.003277162	0.010453286
G10090_24908	13649	Egfr	1.944402114	3.848782418	up	5.19247E-09	4.86727E-08
G10090_27334	13653	Egr1	2.54541097	5.837744095	up	1.21367E-15	2.31737E-14
G10090_13780	13655	Egr3	5.096888473	34.22286112	up	1.79885E-16	3.74356E-15
G10090_15154	13660	Ehd1	2.759176319	6.770096128	up	1.76424E-56	5.38214E-54
G10090_6153	13684	Eif4e	1.455153839	2.741857968	up	4.18796E-25	1.80756E-23
G10090_22265	13803	Enc1	-3.427214459	0.09296204	down	3.03279E-62	1.27216E-59
G10090_20377	13806	Eno1	1.369738616	2.584237413	up	1.18231E-25	5.37972E-24
G10090_24863	13819	Epas1	-2.68377657	0.155633381	down	3.24231E-11	3.95651E-10
G10090_5823	13821	Epb41l1	-1.116972902	0.461060219	down	2.90925E-08	2.46845E-07
G10090_3471	13838	Epha4	4.901958334	29.89761163	up	1.8407E-15	3.41738E-14
G10090_23838	13848	Ephb6	-2.671351317	0.156979566	down	0.00174235	0.005938946
G10090_17496	13849	Ephx1	-1.493121756	0.355243029	down	1.26997E-12	1.83299E-11
G10090_23832	13850	Ephx2	4.700536342	26.00174139	up	0.013803157	0.036408389
G10090_25486	13874	Ereg	9.237730053	603.7176014	up	3.38936E-16	6.86205E-15
G10090_24710	13885	Esd	1.410810026	2.658864074	up	1.89045E-33	1.54729E-31
G10090_4779	14007	Celf2	-1.333022304	0.396935829	down	9.53256E-12	1.23271E-10
G10090_3099	14009	Etv1	2.346167738	5.084717907	up	1.03714E-11	1.33603E-10
G10090_14544	14038	Wfdc18	4.841720244	28.67497343	up	0.001315574	0.004626395
G10090_834	14048	Eya1	-1.863457654	0.274816846	down	1.08553E-31	7.96231E-30
G10090_1152	14051	Eya4	-1.379058836	0.384469528	down	6.05966E-07	4.21445E-06
G10090_19457	14057	Sfxn1	1.220036384	2.329525922	up	2.76218E-19	7.49026E-18
G10090_1353	14058	F10	2.924280884	7.590952288	up	1.63781E-19	4.53286E-18
G10090_4375	14062	F2r	1.600638042	3.032774101	up	0.001803986	0.006117965
G10090_20189	14064	F2rl2	3.045375961	8.255616535	up	3.34759E-16	6.78771E-15
G10090_3217	14066	F3	5.421289025	42.8519522	up	2.80856E-27	1.44442E-25
G10090_14616	14069	F8	-2.416913134	0.187256391	down	0.001944538	0.006553233
G10090_23972	14077	Fabp3	1.760156703	3.387349156	up	1.30182E-09	1.32381E-08
G10090_5280	14081	Acsl1	3.669343003	12.72278852	up	1.3652E-130	3.0542E-127
G10090_30523	14086	Fscn1	3.798776836	13.91700472	up	0.000801168	0.002959303
G10090_18667	14102	Fas	1.358496714	2.564178532	up	8.53704E-23	3.07219E-21
G10090_24269	14130	Fcgr2b	1.534587039	2.897054928	up	1.08926E-09	1.11697E-08
G10090_17725	14132	Fcgrt	-1.816011867	0.28400498	down	2.40925E-15	4.44223E-14

G10090_10667	14166	Fgf11	-1.577483198	0.335065906	down	5.36294E-08	4.37344E-07
G10090_9935	14168	Fgf13	-1.89016444	0.269776308	down	0.000685807	0.002586567
G10090_3470	14182	Fgfr1	-2.260647102	0.208678359	down	4.59251E-11	5.48934E-10
G10090_22161	14187	Akr1b8	1.042766295	2.060174148	up	0.000530099	0.002066664
G10090_24351	14204	Il4i1	2.255757013	4.775848304	up	9.92075E-07	6.65498E-06
G10090_29840	14235	Foxm1	-1.083617227	0.471844297	down	0.007288435	0.020913354
G10090_9423	14238	Foxf2	2.268725632	4.818972713	up	0.0057748	0.017081346
G10090_1230	14261	Fmo1	5.800166333	55.72166002	up	0.000228835	0.000973174
G10090_4293	14263	Fmo5	-2.551818012	0.170539991	down	3.07004E-23	1.13837E-21
G10090_19040	14266	Aff2	1.530873484	2.889607383	up	0.004062386	0.012584677
G10090_2805	14268	Fn1	-2.856664753	0.138056933	down	0.002599706	0.008517417
G10090_24819	14283	Fosl1	3.784223899	13.77732502	up	3.89126E-15	7.03941E-14
G10090_32339	14284	Fosl2	2.116033816	4.335005477	up	1.98178E-20	5.92459E-19
G10090_20999	14289	Fpr2	2.894884379	7.437843432	up	8.36315E-18	1.99394E-16
G10090_5391	14293	Fpr1	2.805524432	6.991124038	up	4.5063E-36	4.35166E-34
G10090_33103	14294	Fpr3	4.358040184	20.50693797	up	0.006183122	0.018121409
G10090_17602	14296	Frat1	-2.469204148	0.180590743	down	1.14781E-10	1.30901E-09
G10090_19788	14313	Fst	7.77966568	219.7418245	up	4.46708E-08	3.71049E-07
G10090_17338	14345	Fut4	-1.201566402	0.434802939	down	0.00103872	0.003760179
G10090_2506	14347	Fut7	-1.294237227	0.407751693	down	2.71485E-05	0.000141906
G10090_23311	14381	G6pdx	1.108833889	2.156712526	up	1.2E-29	7.59791E-28
G10090_2957	14411	Slc6a12	1.086111519	2.123010518	up	2.75102E-08	2.33715E-07
G10090_20850	14412	Slc6a13	1.799973452	3.482138175	up	1.39502E-12	2.00485E-11
G10090_967	14420	Galc	-1.05120415	0.482565222	down	6.35712E-28	3.54073E-26
G10090_28799	14427	Galr1	4.721444814	26.3813194	up	0.000199417	0.000863198
G10090_21188	14431	Gamt	-1.231359763	0.425915824	down	1.01558E-06	6.77877E-06
G10090_22179	14433	Gapdh	1.330982809	2.515739967	up	2.89817E-27	1.47917E-25
G10090_850	14453	Gas2	1.912495187	3.764596359	up	1.21338E-11	1.54968E-10
G10090_22737	14456	Gas6	-2.881419344	0.13570828	down	1.64322E-27	8.61598E-26
G10090_11386	14468	Gbp2b	4.699348669	25.98034474	up	1.13378E-18	2.92107E-17
G10090_21070	14469	Gbp2	3.655779605	12.60373661	up	4.36022E-75	2.92636E-72
G10090_28533	14528	Gch1	2.00529622	4.014711227	up	2.89013E-32	2.21681E-30
G10090_7427	14537	Gcnt1	-2.356110378	0.195317026	down	7.57781E-47	1.35623E-44
G10090_9766	14538	Gcnt2	2.275010112	4.840010282	up	1.72915E-11	2.17938E-10
G10090_30950	14555	Gpd1	-1.674253739	0.313328143	down	0.003201428	0.010231611
G10090_2295	14562	Gdf3	-2.822515693	0.141363768	down	4.89417E-06	2.90042E-05
G10090_7755	14571	Gpd2	2.11313281	4.326297301	up	1.88882E-76	1.3344E-73
G10090_22648	14579	Gem	3.457236507	10.98327584	up	1.0141E-20	3.11494E-19
G10090_10528	14581	Gfi1	4.280399469	19.43249808	up	0.00939777	0.026090231
G10090_106	14588	Gfra4	-2.588078114	0.166307125	down	0.001662426	0.005696896
G10090_9117	14605	Tsc22d3	-2.762792813	0.147338584	down	3.40691E-35	3.15386E-33
G10090_19061	14609	Gja1	5.330286257	40.23241003	up	5.3272E-10	5.67748E-09
G10090_27358	14629	Gclc	1.175992934	2.259483362	up	0.000962649	0.003503787
G10090_30800	14630	Gclm	1.090662529	2.129718171	up	5.67572E-06	3.32251E-05
G10090_12388	14664	Slc6a9	3.933023028	15.27418003	up	2.24721E-25	9.95522E-24
G10090_28553	14667	Gm2a	-1.000570509	0.499802316	down	7.19283E-18	1.73028E-16
G10090_30501	14673	Gna12	-1.650100186	0.31861803	down	3.10401E-30	2.04241E-28
G10090_8325	14676	Gna15	-1.486377698	0.356907544	down	4.21062E-14	6.91789E-13
G10090_2029	14702	Gng2	-1.018413143	0.493659042	down	5.27129E-10	5.62452E-09
G10090_3284	14723	Gp1ba	-1.265966467	0.415820714	down	0.009075491	0.02532175
G10090_17598	14726	Pdpn	2.476411147	5.565113635	up	6.0997E-48	1.16966E-45
G10090_18924	14733	Gpc1	1.028820396	2.040355295	up	0.001472078	0.005115119
G10090_16803	14745	Lpar1	1.0017135	2.002376826	up	0.001580018	0.005446918
G10090_33722	14782	Gsr	2.123117712	4.35634349	up	3.10446E-23	1.14167E-21
G10090_14120	14788	Gpr162	-1.7164392	0.304298853	down	8.64015E-09	7.8575E-08
G10090_5741	14814	Grin2d	1.139770628	2.203459879	up	0.011400928	0.030864354
G10090_22095	14825	Cxcl1	7.094709898	136.6848844	up	1.73892E-35	1.65543E-33
G10090_25684	14828	Hspa5	1.470472767	2.771126879	up	8.83268E-71	5.15483E-68
G10090_4262	14854	Gss	1.124657523	2.180497782	up	5.42442E-05	0.000267888

G10090_16517	14857	Gsta1	5.115606344	34.6697696	up	8.1431E-17	1.75449E-15
G10090_19134	14858	Gsta2	6.462258249	88.17258485	up	1.10854E-10	1.26745E-09
G10090_22885	14860	Gsta4	1.818342369	3.526757476	up	0.000636104	0.002420873
G10090_25302	14863	Gstm2	1.370178966	2.585026314	up	0.001491619	0.005174891
G10090_255	14933	Gk	1.453194073	2.738135939	up	4.03667E-37	4.36969E-35
G10090_18039	14936	Gys1	2.206663381	4.616064495	up	2.72894E-31	1.92793E-29
G10090_27825	14960	H2-Aa	-1.805668639	0.286048436	down	2.92123E-09	2.85176E-08
G10090_26108	14961	H2-Ab1	-1.518539766	0.34903902	down	6.51181E-11	7.68761E-10
G10090_8515	14962	Cfb	4.879243604	29.43057052	up	2.10337E-16	4.33696E-15
G10090_2518	14990	H2-M2	3.88071022	14.73025214	up	1.40586E-47	2.62096E-45
G10090_24279	14998	H2-DMa	-1.596427288	0.330694902	down	2.25884E-08	1.94862E-07
G10090_28333	14999	H2-DMb1	-1.101603219	0.465998359	down	4.47622E-06	2.66923E-05
G10090_20128	15001	H2-Oa	2.199506396	4.593221625	up	1.06912E-19	3.02121E-18
G10090_22897	15018	H2-Q7	1.629084286	3.093166055	up	0.000628983	0.002400581
G10090_20484	15024	H2-T10	1.58223917	2.994342335	up	7.01106E-06	4.05275E-05
G10090_4806	15039	H2-T22	1.265139051	2.403503745	up	0.000248382	0.001045807
G10090_12491	15064	Mr1	-1.087320671	0.470634612	down	3.41866E-09	3.28716E-08
G10090_28557	15162	Hck	1.14438339	2.210516326	up	5.7717E-55	1.58109E-52
G10090_23215	15163	Hcls1	1.417986045	2.672122309	up	3.03273E-17	6.91144E-16
G10090_11179	15186	Hdc	4.259866032	19.15788019	up	0.000178776	0.000783194
G10090_28754	15200	Hbegf	7.176852845	144.6931532	up	6.76479E-14	1.08747E-12
G10090_16472	15213	Hey1	-2.586368077	0.166504367	down	0.008280909	0.023405905
G10090_570	15216	Hfe	-2.169916601	0.222223516	down	1.19095E-15	2.27722E-14
G10090_1266	15234	Hgf	-1.809792922	0.285231867	down	3.76492E-09	3.59435E-08
G10090_4840	15251	Hif1a	1.403629947	2.645664176	up	2.0312E-07	1.51303E-06
G10090_16824	15277	Hk2	1.190419953	2.282191657	up	2.12317E-13	3.26452E-12
G10090_20345	15368	Hmox1	1.249364494	2.377366772	up	1.28799E-07	9.90187E-07
G10090_621	15370	Nr4a1	1.669835286	3.181782648	up	3.11215E-09	3.01838E-08
G10090_15907	15439	Hp	2.80985635	7.012147534	up	5.35222E-25	2.2953E-23
G10090_18069	15442	Hpse	-1.639940368	0.320869737	down	5.24767E-07	3.68407E-06
G10090_16222	15446	Hpgd	-4.609601501	0.040961107	down	0.000377751	0.001524061
G10090_11852	15460	Hr	-3.065171364	0.119478972	down	8.55969E-10	8.87919E-09
G10090_11099	15465	Hrh1	-2.035026843	0.2440034	down	0.005846966	0.017260574
G10090_2211	15466	Hrh2	1.564231443	2.95719923	up	0.001723548	0.005882326
G10090_19503	15483	Hsd11b1	2.977954242	7.87868165	up	9.55331E-15	1.64825E-13
G10090_32014	15519	Hsp90aa1	1.281359039	2.430678424	up	4.54641E-14	7.43318E-13
G10090_12063	15551	Htr1b	5.319357032	39.92877849	up	0.00155369	0.005365368
G10090_12615	15557	Htr1f	3.404403709	10.58833404	up	0.000607108	0.002328346
G10090_14528	15559	Htr2b	-1.187783646	0.438976725	down	2.23425E-05	0.000118586
G10090_9963	15566	Htr7	5.945144936	61.61223374	up	1.914E-08	1.66397E-07
G10090_29195	15586	Hyal1	-1.194303148	0.436997476	down	1.84974E-09	1.84055E-08
G10090_26682	15894	Icam1	1.504148837	2.836572693	up	5.03694E-52	1.12685E-49
G10090_32334	15903	Id3	-1.373288536	0.386010357	down	9.81996E-14	1.54892E-12
G10090_12377	15926	Idh1	-1.02099019	0.492778019	down	4.98406E-09	4.68495E-08
G10090_22730	15930	Ido1	2.412986683	5.325757293	up	0.01132441	0.030733433
G10090_28332	15937	Ier3	3.978686744	15.76536584	up	1.43183E-47	2.6328E-45
G10090_23922	15945	Cxcl10	1.308635901	2.477072168	up	0.014142808	0.037230617
G10090_19414	15951	Ifi204	1.039265935	2.05518168	up	0.000680667	0.002571452
G10090_20832	15953	Ifi47	2.088166079	4.252072156	up	2.23259E-11	2.77997E-10
G10090_33435	15957	Ifit1	2.072726939	4.206810813	up	1.1871E-05	6.63108E-05
G10090_30309	15979	Ifngr1	-1.081273919	0.472611317	down	2.09589E-20	6.2518E-19
G10090_26397	15982	Ifrd1	1.636746265	3.109637185	up	2.83795E-18	6.9897E-17
G10090_4336	16000	Igf1	-2.199452591	0.217720236	down	6.98122E-26	3.22024E-24
G10090_13653	16007	Cyr61	4.558319155	23.56084139	up	0.004043705	0.012535485
G10090_13396	16010	Igfbp4	-1.880364655	0.271615054	down	4.82902E-57	1.66205E-54
G10090_24776	16145	Igtp	1.248582594	2.376078655	up	1.30661E-08	1.15462E-07
G10090_15535	16149	Cd74	-1.42270372	0.373012603	down	1.25976E-20	3.82573E-19
G10090_22779	16153	Il10	4.847654475	28.79316497	up	9.56792E-18	2.27713E-16
G10090_3976	16159	Il12a	10.44164206	1390.744859	up	4.03812E-17	9.06131E-16

G10090_22230	16160	Il12b	7.094141314	136.6310258	up	5.0698E-10	5.42247E-09
G10090_780	16161	Il12rb1	1.489353484	2.807631283	up	0.001550102	0.005355731
G10090_9074	16162	Il12rb2	-2.473565521	0.180045629	down	5.07279E-05	0.000251726
G10090_26207	16164	Il13ra1	2.358919318	5.129859517	up	1.5286E-21	5.02902E-20
G10090_18162	16165	Il13ra2	2.460717169	5.504903099	up	0.008347479	0.02356925
G10090_20955	16170	Il16	-1.050204184	0.482899815	down	3.07542E-17	6.98501E-16
G10090_4537	16175	Il1a	10.137476	1126.378514	up	2.8397E-154	7.6235E-151
G10090_13592	16176	Il1b	9.071354294	537.9597014	up	8.46484E-39	9.96698E-37
G10090_6376	16177	Il1r1	2.087384904	4.249770413	up	9.49649E-11	1.09794E-09
G10090_17992	16181	Il1rn	4.102357026	17.17641474	up	2.00835E-61	8.16911E-59
G10090_30411	16193	Il6	9.121207273	556.8740567	up	1.45563E-29	9.08786E-28
G10090_15992	16194	Il6ra	-1.345673141	0.393470358	down	4.91773E-09	4.62908E-08
G10090_11209	16323	Inhba	6.26068002	76.67476956	up	1.89734E-10	2.1188E-09
G10090_25862	16329	Inpp1	-1.054867582	0.4813414	down	0.000492902	0.001936833
G10090_25546	16365	Acod1	3.60373285	12.15714745	up	1.9437E-107	3.2613E-104
G10090_15313	16403	Itga6	-1.968928557	0.25544267	down	2.99128E-09	2.90746E-08
G10090_4121	16418	Eif6	1.480700533	2.790842161	up	6.80677E-40	8.1578E-38
G10090_428	16419	Itgb5	-2.66145632	0.158059941	down	2.36267E-48	4.66383E-46
G10090_17119	16421	Itgb7	-1.720608553	0.303420706	down	1.53983E-06	9.95141E-06
G10090_5478	16438	Itpr1	-1.286148839	0.41004415	down	1.05677E-05	5.95509E-05
G10090_23177	16449	Jag1	2.30135421	4.92920236	up	1.76845E-30	1.19889E-28
G10090_4653	16452	Jak2	2.600465035	6.064820873	up	1.43008E-93	1.9196E-90
G10090_25163	16477	Junb	1.06433574	2.091206803	up	3.63404E-26	1.72978E-24
G10090_18111	16480	Jup	-1.427584527	0.371752789	down	0.001280724	0.004517948
G10090_23187	16491	Kcna3	2.382060455	5.212807031	up	1.05459E-17	2.50102E-16
G10090_20743	16513	Kcnj10	-2.760651977	0.147557384	down	1.36121E-17	3.20552E-16
G10090_2275	16531	Kcnma1	3.477433252	11.13811549	up	0.01886293	0.047566618
G10090_419	16548	Khk	-1.56493189	0.337993665	down	5.91675E-13	8.78545E-12
G10090_29077	16551	Kif11	-1.968895987	0.255448437	down	5.85426E-05	0.000286376
G10090_3861	16554	Kif13b	-1.676755899	0.312785188	down	1.12286E-33	9.53936E-32
G10090_3575	16560	Kif1a	3.412692227	10.64934082	up	8.28179E-08	6.57401E-07
G10090_927	16564	Kif21a	2.473425803	5.553609751	up	6.55754E-07	4.5302E-06
G10090_11788	16571	Kif4	-1.811295744	0.284934902	down	0.014938988	0.038929534
G10090_27882	16574	Kif5c	-2.497494353	0.177083985	down	1.64276E-07	1.2409E-06
G10090_8709	16576	Kif7	-1.863320657	0.274842943	down	6.55435E-07	4.5302E-06
G10090_11643	16578	Kif9	-1.208367786	0.432757946	down	5.62463E-07	3.93021E-06
G10090_374	16581	Kifc2	-1.156140355	0.44871137	down	8.34157E-06	4.76666E-05
G10090_4229	16592	Fabp5	-1.999325339	0.250116937	down	4.86331E-19	1.29782E-17
G10090_18207	16598	Klf2	-1.370792429	0.386678799	down	4.61929E-08	3.83218E-07
G10090_33796	16627	Klra1	-5.927014107	0.016435805	down	1.61675E-11	2.04732E-10
G10090_14822	16633	Klra2	1.979044808	3.942319788	up	0.001224909	0.004349722
G10090_18113	16635	Klra4	-5.535361602	0.021562054	down	9.04086E-05	0.000424914
G10090_9164	16658	Mafb	-1.466600874	0.36183381	down	5.13082E-09	4.81615E-08
G10090_29	16706	Ksr1	1.558954827	2.946403113	up	4.28379E-13	6.46808E-12
G10090_10196	16728	L1cam	-1.948096787	0.259157888	down	2.09985E-29	1.29295E-27
G10090_19731	16763	Lad1	3.567897864	11.85889649	up	0.000272969	0.001135088
G10090_13966	16765	Stmn1	-1.33011578	0.397736321	down	0.00939541	0.026089075
G10090_11410	16774	Lama3	-2.921368585	0.132001975	down	8.06696E-05	0.000382895
G10090_3275	16782	Lamc2	1.589600672	3.009660326	up	1.14225E-05	6.4045E-05
G10090_14465	16797	Lat	1.654273504	3.147646452	up	1.41531E-05	7.77956E-05
G10090_23049	16819	Lcn2	6.13860824	70.4539281	up	5.13998E-59	1.9165E-56
G10090_12913	16828	Ldha	1.732301257	3.322573828	up	1.36656E-27	7.33734E-26
G10090_9560	16832	Ldhb	-1.971596811	0.254970667	down	1.82455E-13	2.8278E-12
G10090_4284	16847	Lepr	6.230976222	75.11224544	up	1.28719E-05	7.13078E-05
G10090_13192	16848	Lfng	-1.208818558	0.432622751	down	1.4916E-17	3.50031E-16
G10090_4610	16859	Lgals9	1.149380866	2.218186803	up	1.08259E-44	1.79403E-42
G10090_10511	16878	Lif	5.747965971	53.74154793	up	3.21996E-06	1.9664E-05
G10090_1890	16880	Lifr	-2.778050843	0.145788533	down	0.006502002	0.018950765
G10090_12923	16889	Lipa	-1.398181944	0.379406962	down	6.18857E-15	1.08729E-13

G10090_11989	16909	Lmo2	-1.231079471	0.425998581	down	2.35771E-10	2.60904E-09
G10090_11211	16911	Lmo4	2.114001272	4.328902394	up	3.98913E-29	2.41199E-27
G10090_188	16913	Psmb8	1.158889898	2.232855513	up	4.60225E-08	3.8204E-07
G10090_31160	16948	Lox	3.639711254	12.46413841	up	4.35237E-64	2.01455E-61
G10090_19657	16973	Lrp5	-1.748956527	0.297516889	down	1.83968E-17	4.30961E-16
G10090_720	16975	Lrp8	1.04686401	2.066034019	up	0.000168448	0.000742714
G10090_24633	16976	Lrpap1	1.152154675	2.22245572	up	1.02482E-32	8.09186E-31
G10090_3697	16985	Lsp1	-3.234446453	0.106251385	down	6.23764E-23	2.27521E-21
G10090_498	16995	Ltb4r1	2.314971845	4.975949502	up	2.74652E-07	2.00363E-06
G10090_10515	16997	Ltbp2	-1.092242916	0.469031618	down	7.17034E-08	5.74612E-07
G10090_11195	16998	Ltbp3	-1.082290989	0.472278253	down	2.65878E-11	3.27721E-10
G10090_12163	17022	Lum	5.996242879	63.8335456	up	6.2821E-05	0.000304641
G10090_24308	17057	Klrb1a	-1.498099807	0.354019367	down	0.00072234	0.002699509
G10090_30458	17064	Cd93	-1.747037267	0.297912948	down	1.23688E-22	4.39222E-21
G10090_12386	17076	Ly75	1.906266809	3.74837894	up	1.18049E-07	9.14883E-07
G10090_32512	17084	Ly86	-1.877159016	0.272219248	down	7.43509E-06	4.27964E-05
G10090_28168	17095	Lyl1	-1.781884267	0.290803338	down	4.73868E-17	1.0479E-15
G10090_8222	17112	Tm4sf1	5.794946128	55.52040273	up	2.83832E-22	9.7689E-21
G10090_21842	17113	M6pr	1.097871382	2.14038657	up	1.0428E-28	6.08585E-27
G10090_31213	17122	Mxd4	-1.674732157	0.313224256	down	4.61534E-34	4.02284E-32
G10090_10314	17127	Smad3	-1.6898175	0.309966133	down	4.16473E-06	2.49903E-05
G10090_24871	17132	Maf	-1.78305735	0.290566976	down	1.55599E-27	8.22288E-26
G10090_13131	17133	Maff	3.25661005	9.557346007	up	6.21868E-25	2.63323E-23
G10090_2996	17159	Man2b1	-1.723731838	0.302764542	down	2.36589E-12	3.32887E-11
G10090_26694	17167	Marco	3.220915964	9.323786462	up	4.61371E-38	5.24829E-36
G10090_2955	17179	Matk	-2.74520305	0.149145975	down	3.0916E-23	1.14007E-21
G10090_5640	17193	Mbd4	-1.532932725	0.345574166	down	2.13911E-14	3.58021E-13
G10090_2096	17219	Mcm6	-1.138779359	0.454143659	down	2.1471E-05	0.000114276
G10090_1170	17250	Abcc1	1.304539887	2.470049385	up	3.46392E-09	3.32115E-08
G10090_17557	17254	Slc3a2	1.03755943	2.052752127	up	2.95563E-11	3.61325E-10
G10090_15442	17258	Mef2a	-1.112089362	0.462623558	down	3.68426E-50	7.72716E-48
G10090_2660	17260	Mef2c	-2.295469269	0.203701814	down	3.77855E-29	2.295E-27
G10090_12567	17289	Mertk	-1.215145578	0.430729614	down	1.91108E-16	3.95871E-15
G10090_7100	17295	Met	3.21158549	9.263680457	up	4.84577E-75	3.09737E-72
G10090_28481	17299	Mettl1	1.278593552	2.426023545	up	1.60253E-09	1.61129E-08
G10090_12759	17304	Mfge8	-1.27080487	0.414428502	down	0.000569822	0.002202972
G10090_6001	17305	Mfng	-1.263584916	0.416507703	down	0.000822513	0.003030631
G10090_23740	17311	Kitl	-2.917030945	0.132399452	down	7.12922E-07	4.89993E-06
G10090_32861	17319	Mif	1.765052129	3.398862805	up	2.64224E-22	9.14094E-21
G10090_5518	17345	Mki67	1.729283731	3.315631629	up	7.75335E-12	1.01634E-10
G10090_10600	17357	Marcksl1	1.935003603	3.82379082	up	1.49507E-18	3.81527E-17
G10090_19916	17381	Mmp12	1.413500349	2.663826922	up	1.31323E-16	2.75861E-15
G10090_15945	17384	Mmp10	5.364646098	41.20210376	up	5.80043E-09	5.40689E-08
G10090_23811	17386	Mmp13	5.954510016	62.013483	up	1.534E-207	6.8634E-204
G10090_15210	17387	Mmp14	1.458941349	2.74906563	up	6.78036E-10	7.16072E-09
G10090_22343	17390	Mmp2	1.692273138	3.231654885	up	0.003295124	0.010501058
G10090_7761	17392	Mmp3	9.779593324	878.9232154	up	4.87121E-14	7.94487E-13
G10090_20259	17395	Mmp9	3.270350173	9.648804306	up	0.001409532	0.004918801
G10090_13177	17444	Grap2	-3.845492021	0.069565122	down	0.000646244	0.002455289
G10090_1867	17448	Mdh2	1.093235865	2.133520341	up	8.29863E-21	2.57853E-19
G10090_20016	17470	Cd200	3.423060863	10.72615323	up	4.54701E-15	8.1271E-14
G10090_18194	17472	Gbp4	3.517269623	11.44995182	up	4.69976E-09	4.43635E-08
G10090_4252	17528	Mpz	-1.582848435	0.333822144	down	0.016148593	0.041621076
G10090_32096	17533	Mrc1	-2.986503532	0.126174867	down	0.000968363	0.003522584
G10090_23998	17685	Msh2	-1.231449323	0.425889385	down	6.45239E-09	5.98139E-08
G10090_7524	17691	Sik1	-1.576059673	0.335396683	down	9.05138E-14	1.43953E-12
G10090_14332	17698	Msn	1.121968194	2.176436901	up	4.54849E-49	9.39299E-47
G10090_14936	17748	Mt1	1.692496692	3.232155687	up	5.41352E-15	9.58651E-14
G10090_19624	17750	Mt2	2.721028963	6.593429046	up	3.76983E-37	4.11402E-35

G10090_28373	17751	Mt3	4.33107799	20.12724757	up	2.25085E-05	0.000119372
G10090_27107	17755	Map1b	3.172006964	9.012997336	up	0.001835672	0.006220709
G10090_8406	17761	Map7	-2.00038843	0.249932699	down	0.012785258	0.034044143
G10090_20304	17768	Mthfd2	2.576745008	5.965921556	up	3.15256E-52	7.17234E-50
G10090_1589	17771	Tesmin	-1.171048162	0.444098572	down	0.004217792	0.013018033
G10090_15687	17772	Mtm1	1.201988802	2.300565929	up	1.286E-20	3.89662E-19
G10090_974	17863	Myb	6.483535865	89.48263684	up	5.13155E-06	3.03039E-05
G10090_18121	17948	Naip2	1.00067391	2.000934456	up	2.07724E-14	3.48535E-13
G10090_21682	17974	Nck2	-1.289258432	0.40916129	down	0.004448996	0.013625114
G10090_29919	18011	Neurl1a	-2.464594217	0.181168719	down	3.3578E-09	3.24257E-08
G10090_7458	18019	Nfatc2	-2.387209535	0.19115177	down	1.09443E-27	5.92358E-26
G10090_4057	18022	Nfe2	-2.101806744	0.232966312	down	1.36184E-08	1.20184E-07
G10090_9340	18027	Nfia	-1.733136377	0.300797321	down	5.12592E-05	0.000254175
G10090_22739	18030	Nfil3	2.570722776	5.941069956	up	6.24797E-26	2.91203E-24
G10090_2206	18032	Nfix	-2.292962382	0.204056082	down	3.5093E-08	2.95517E-07
G10090_6058	18034	Nfkb2	1.396158614	2.631998404	up	5.18232E-20	1.49919E-18
G10090_15778	18035	Nfkbia	1.653993032	3.147034582	up	4.84052E-35	4.45029E-33
G10090_17457	18036	Nfkbib	1.237937291	2.358610661	up	6.96682E-29	4.15212E-27
G10090_1003	18037	Nfkbie	2.253290049	4.767688728	up	2.57029E-37	2.82796E-35
G10090_4839	18049	Ngf	6.32723125	80.29461049	up	2.32914E-05	0.000123184
G10090_31997	18073	Nid1	2.023733044	4.066346188	up	0.000925837	0.003379796
G10090_32434	18104	Nqo1	1.766003766	3.401105518	up	1.92774E-10	2.15096E-09
G10090_12526	18124	Nr4a3	4.619926127	24.58874381	up	1.98473E-12	2.8132E-11
G10090_26551	18126	Nos2	7.760106551	216.78281	up	3.92548E-34	3.46656E-32
G10090_19393	18164	Nptx1	-5.042089253	0.030351481	down	1.54367E-22	5.45281E-21
G10090_14761	18173	Slc11a1	1.372675855	2.589504122	up	1.51657E-19	4.22342E-18
G10090_14369	18227	Nr4a2	2.564601773	5.91591684	up	8.59713E-07	5.82237E-06
G10090_32990	18230	Nxn	3.323251241	10.00917556	up	2.23521E-08	1.93071E-07
G10090_28535	18263	Odc1	1.43861811	2.710611044	up	3.318E-11	4.04519E-10
G10090_18578	18383	Tnfrsf11b	6.745218613	107.2786081	up	1.12751E-05	6.33244E-05
G10090_20356	18405	Orm1	1.734601613	3.327875849	up	0.004947342	0.014919832
G10090_22267	18413	Osm	2.991770178	7.954494096	up	8.81803E-16	1.71047E-14
G10090_31773	18415	Hspa4l	1.501352735	2.831080428	up	1.92547E-13	2.97417E-12
G10090_5489	18439	P2rx7	-1.245981792	0.421620876	down	3.12861E-19	8.44977E-18
G10090_22532	18440	P2rx6	-2.528459671	0.173323638	down	1.79699E-05	9.69105E-05
G10090_9950	18451	P4ha1	1.124375389	2.180071404	up	1.75915E-18	4.43854E-17
G10090_21162	18452	P4ha2	2.140826351	4.410145796	up	2.61688E-07	1.91529E-06
G10090_31046	18453	P4hb	1.10349394	2.148744489	up	5.88811E-34	5.0991E-32
G10090_33594	18477	Prdx1	2.081287498	4.231847086	up	2.37293E-41	3.12274E-39
G10090_9110	18483	Palm	-1.629184203	0.323270955	down	0.000164964	0.000728394
G10090_17824	18563	Pcx	2.462256542	5.510780034	up	3.08261E-43	4.6492E-41
G10090_10288	18569	Pdcd4	-1.435198289	0.369796048	down	2.63689E-17	6.02981E-16
G10090_3247	18575	Pde1c	-2.736112148	0.150088761	down	0.000269659	0.001122017
G10090_5852	18576	Pde3b	-1.41763527	0.374325368	down	9.71822E-05	0.000452985
G10090_4168	18578	Pde4b	1.972272807	3.923857929	up	5.46933E-19	1.44517E-17
G10090_11132	18591	Pdgfb	-1.229819915	0.426370664	down	2.19834E-07	1.62939E-06
G10090_7374	18600	Padi2	-1.91969207	0.264310919	down	1.17214E-09	1.19829E-08
G10090_24174	18604	Pdk2	-1.070218206	0.476246962	down	2.04067E-15	3.7782E-14
G10090_14887	18605	Enpp1	-2.194209205	0.218512966	down	2.19331E-06	1.38155E-05
G10090_5227	18606	Enpp2	3.236716868	9.426465116	up	0.00682278	0.019754567
G10090_2684	18612	Etv4	4.841933689	28.67921617	up	0.006098984	0.017929624
G10090_5310	18628	Per3	-2.086003026	0.235532325	down	6.58095E-34	5.6265E-32
G10090_31978	18636	Cfp	1.254885531	2.386482125	up	9.0899E-12	1.17774E-10
G10090_2971	18641	Pfkl	1.881637077	3.684929652	up	2.27543E-22	7.91271E-21
G10090_32068	18645	Pfn2	1.573938801	2.977164217	up	0.008608188	0.024173161
G10090_15914	18648	Pgam1	1.144780032	2.21112415	up	2.97181E-26	1.4196E-24
G10090_17362	18654	Pgf	2.32488106	5.01024468	up	0.002038937	0.006838742
G10090_24476	18655	Pgk1	1.666291265	3.1739761	up	2.801E-127	5.3711E-124
G10090_30413	18671	Abcb1a	1.230144526	2.345904895	up	0.000691563	0.002604615

G10090_24402	18712	Pim1	2.289503959	4.888879882	up	1.25814E-19	3.53307E-18
G10090_10841	18719	Pip5k1b	1.081279687	2.115912087	up	0.002904441	0.009401088
G10090_9550	18768	Pkib	-1.477627055	0.35907894	down	4.0926E-05	0.000206522
G10090_11144	18783	Pla2g4a	2.098030088	4.28124408	up	6.24031E-17	1.36423E-15
G10090_3062	18784	Pla2g5	1.902077795	3.737510922	up	0.006413595	0.01871515
G10090_29841	18788	Serpinb2	8.484086972	358.0673024	up	4.35698E-18	1.05949E-16
G10090_264	18793	Plaur	1.956843568	3.882116918	up	6.71197E-23	2.43499E-21
G10090_2689	18796	Plcb2	-1.109568522	0.463432613	down	2.58572E-30	1.71822E-28
G10090_5156	18798	Plcb4	2.005668577	4.01574755	up	1.22077E-08	1.08355E-07
G10090_29159	18817	Plk1	-1.399228363	0.379131869	down	0.008677299	0.024341773
G10090_9501	18823	Plp1	2.182114019	4.538180588	up	0.002307923	0.007649199
G10090_22494	18830	Pltp	-1.5790006	0.334713675	down	1.95564E-10	2.18028E-09
G10090_6217	18968	Pola1	-1.166302531	0.445561805	down	3.05914E-05	0.00015836
G10090_19767	18973	Pole	-1.225983635	0.427505938	down	0.005351256	0.015997753
G10090_19582	19012	Plpp1	3.304159904	9.877595596	up	6.84159E-53	1.66972E-50
G10090_4761	19016	Pparg	-2.104485193	0.232534198	down	7.53027E-05	0.000358563
G10090_14588	19073	Srgn	1.978711015	3.94140777	up	4.0436E-17	9.06131E-16
G10090_25462	19088	Prkar2b	1.707792261	3.26660556	up	3.57757E-32	2.72851E-30
G10090_28667	19124	Procr	2.575004903	5.958730102	up	2.01019E-34	1.79886E-32
G10090_922	19125	Prodh	-1.447808234	0.366577912	down	0.000399844	0.001602121
G10090_5940	19128	Pros1	-1.608060847	0.328038978	down	1.50355E-10	1.69314E-09
G10090_8619	19141	Lgmn	-2.465269824	0.181083898	down	3.04062E-24	1.24056E-22
G10090_12301	19153	Prx	1.048708953	2.068677789	up	0.0145692	0.03812132
G10090_9751	19155	Npepps	1.013814567	2.01924304	up	1.42317E-17	3.34557E-16
G10090_11673	19157	Cyth1	-1.165365205	0.445851382	down	1.04172E-36	1.09242E-34
G10090_6841	19171	Psmb10	1.125668228	2.182025904	up	1.69774E-16	3.54413E-15
G10090_16961	19173	Psmb5	1.384710401	2.611195364	up	2.71982E-31	1.92793E-29
G10090_2582	19175	Psmb6	1.19373405	2.287440236	up	2.77029E-25	1.20732E-23
G10090_3159	19188	Psme2	2.005738361	4.015941797	up	4.32241E-87	4.46306E-84
G10090_3469	19218	Ptger3	-3.77261918	0.073169227	down	2.05453E-16	4.24277E-15
G10090_24864	19221	Ptgfrn	-1.59839929	0.330243188	down	6.31767E-05	0.000306034
G10090_16411	19225	Ptgs2	7.464680007	176.6414374	up	7.93213E-20	2.27333E-18
G10090_7998	19246	Ptpn1	1.35828639	2.56380474	up	9.98654E-16	1.92877E-14
G10090_10113	19250	Ptpn14	3.932110869	15.26452582	up	1.06036E-11	1.36333E-10
G10090_19296	19253	Ptpn18	-1.938932878	0.260809282	down	1.07337E-07	8.3611E-07
G10090_5998	19260	Ptpn22	-1.371389626	0.386518768	down	5.13529E-06	3.03127E-05
G10090_1085	19275	Ptprn	1.717272086	3.288140819	up	2.36117E-13	3.6098E-12
G10090_3910	19277	Ptpro	-1.484301575	0.357421525	down	9.57049E-09	8.66833E-08
G10090_4953	19280	Ptprs	-1.18145331	0.440907123	down	2.59787E-31	1.85485E-29
G10090_3363	19325	Rab10	1.159721871	2.234143527	up	1.39184E-24	5.76949E-23
G10090_10065	19332	Rab20	1.781683542	3.438271671	up	1.40506E-30	9.57369E-29
G10090_12548	19348	Kif20a	-1.482559678	0.357853333	down	0.002387786	0.007884687
G10090_9579	19395	Rasgrp2	-1.918495397	0.264530248	down	0.01943284	0.048792931
G10090_24374	19400	Rapsn	-2.659372941	0.158288358	down	0.000257975	0.0010801
G10090_19719	19414	Rasa3	-1.102857285	0.465593465	down	4.21496E-11	5.07876E-10
G10090_1901	19415	Rasal1	-1.304928516	0.404741162	down	4.25309E-05	0.000213657
G10090_13039	19417	Rasgrf1	2.753715581	6.744519109	up	4.39624E-05	0.000220189
G10090_20212	19419	Rasgrp1	5.381282818	41.67998397	up	5.2629E-210	3.5322E-206
G10090_931	19663	Rbpms	1.198076272	2.294335341	up	3.52441E-05	0.000180016
G10090_16081	19730	Ralgds	1.73050079	3.31842988	up	1.60124E-33	1.32676E-31
G10090_3480	19731	Rgl1	2.600802454	6.066239486	up	3.35592E-34	2.98321E-32
G10090_9703	19735	Rgs2	-2.848682278	0.138822924	down	2.84951E-30	1.88419E-28
G10090_5291	19739	Rgs9	3.71073645	13.09311485	up	4.94943E-20	1.43491E-18
G10090_5868	20112	Rps6ka2	1.026311377	2.036809957	up	4.33037E-09	4.10788E-08
G10090_1285	20135	Rrm2	-1.245735807	0.42169277	down	0.004266691	0.013135732
G10090_11131	20148	Dhrs3	-2.487647988	0.178296713	down	7.25762E-22	2.44158E-20
G10090_23290	20167	Rtn2	1.619387303	3.072445251	up	9.74895E-05	0.00045406
G10090_22937	20181	Rxra	-1.171754896	0.443881074	down	5.78844E-16	1.14262E-14
G10090_31793	20201	S100a8	3.477912361	11.14181499	up	1.28163E-05	7.10295E-05

G10090_18458	20210	Saa3	5.155015806	35.62988215	up	1.89926E-53	4.72107E-51
G10090_20604	20229	Sat1	-1.147486558	0.451410988	down	6.47588E-12	8.61504E-11
G10090_23389	20250	Scd2	1.405492437	2.649081882	up	5.56894E-14	9.02801E-13
G10090_14556	20259	Scin	3.073671069	8.419129477	up	0.001417085	0.004939375
G10090_18998	20296	Ccl2	7.161767057	143.1880274	up	2.00207E-25	8.95791E-24
G10090_23183	20299	Ccl22	4.412238534	21.29198477	up	1.76557E-06	1.12532E-05
G10090_20420	20302	Ccl3	5.610275536	48.84962344	up	8.3516E-199	2.8026E-195
G10090_15116	20303	Ccl4	6.27464979	77.42082575	up	3.49113E-85	3.34725E-82
G10090_30491	20304	Ccl5	4.309389997	19.82693816	up	9.54685E-08	7.50717E-07
G10090_545	20306	Ccl7	8.05404632	265.7721861	up	1.30411E-15	2.47597E-14
G10090_10574	20310	Cxcl2	6.371197494	82.77926274	up	1.22365E-36	1.26347E-34
G10090_30788	20311	Cxcl5	8.695299329	414.5204102	up	7.1552E-08	5.73741E-07
G10090_7533	20335	Sec61g	1.072399963	2.102928739	up	1.41101E-10	1.59293E-09
G10090_33780	20348	Sema3c	2.454157161	5.479928848	up	3.43119E-07	2.47086E-06
G10090_33335	20349	Sema3e	2.820556531	7.064348571	up	0.011606051	0.03132047
G10090_10301	20351	Sema4a	1.001862171	2.002583184	up	2.14217E-05	0.000114104
G10090_9518	20361	Sema7a	3.275481874	9.683186449	up	5.10353E-07	3.59039E-06
G10090_5522	20363	Selenop	-2.539977236	0.17194544	down	9.03175E-57	2.95691E-54
G10090_15783	20365	Serf1	3.707621086	13.06487201	up	2.1949E-22	7.65252E-21
G10090_3306	20392	Sgce	1.007613099	2.010581892	up	0.003085835	0.009899897
G10090_1592	20410	Sorbs3	-2.370384332	0.193394097	down	3.45575E-09	3.31807E-08
G10090_27421	20415	Shbg	3.800407466	13.93274354	up	2.46247E-06	1.53524E-05
G10090_10147	20440	St6gal1	-4.146678306	0.056457995	down	3.33427E-42	4.71114E-40
G10090_13401	20442	St3gal1	1.086510188	2.123597264	up	1.7722E-18	4.45472E-17
G10090_20779	20452	St8sia4	-1.587955234	0.332642582	down	5.27852E-20	1.52373E-18
G10090_9122	20454	St3gal5	-1.532120582	0.345768756	down	1.19344E-08	1.0609E-07
G10090_30722	20471	Six1	5.191338319	36.53831805	up	1.021E-22	3.65464E-21
G10090_26111	20474	Six4	4.121501487	17.40586355	up	0.011797324	0.031757841
G10090_4815	20475	Six5	2.959659397	7.779402739	up	3.27411E-05	0.000168256
G10090_15366	20501	Slc16a1	1.168437021	2.24768057	up	1.22499E-05	6.82569E-05
G10090_7350	20503	Slc16a7	-1.359548509	0.389704229	down	2.53188E-19	6.90761E-18
G10090_29020	20510	Slc1a1	4.496203975	22.56795791	up	0.005019324	0.015113143
G10090_24780	20511	Slc1a2	7.321080283	159.9060023	up	6.20211E-17	1.35809E-15
G10090_19003	20515	Slc20a1	1.932949382	3.818350084	up	4.37179E-26	2.05903E-24
G10090_16317	20525	Slc2a1	2.167520202	4.492505288	up	3.74719E-15	6.80629E-14
G10090_15452	20527	Slc2a3	-1.714526898	0.30470247	down	1.08673E-08	9.73127E-08
G10090_13625	20539	Slc7a5	1.018846726	2.026298512	up	0.000665915	0.002523596
G10090_8655	20540	Slc7a7	-1.296205502	0.407195775	down	1.67927E-15	3.13066E-14
G10090_28380	20555	Slfn1	1.942877275	3.844716643	up	0.005621506	0.016697825
G10090_11768	20562	Slit1	-4.75480518	0.03703915	down	0.001527229	0.005286228
G10090_6029	20563	Slit2	2.526344617	5.761101267	up	4.68156E-08	3.87905E-07
G10090_29217	20568	Slpi	3.651032861	12.56233604	up	3.88969E-08	3.25913E-07
G10090_26850	20617	Snca	2.381751304	5.211690115	up	9.81434E-08	7.69048E-07
G10090_12381	20620	Plk2	2.368454683	5.163877166	up	3.08548E-23	1.14007E-21
G10090_22563	20621	Snn	-2.856751399	0.138048642	down	5.6192E-15	9.89849E-14
G10090_20631	20656	Sod2	2.741301684	6.686733796	up	4.23913E-37	4.55215E-35
G10090_4331	20680	Sox7	-3.220174542	0.107307696	down	0.004819907	0.014597836
G10090_3855	20698	Sphk1	2.982540809	7.903769146	up	5.32086E-07	3.7335E-06
G10090_15584	20708	Serpinb6b	-1.561914829	0.338701239	down	0.000602141	0.002314586
G10090_19167	20713	Serpini1	2.118685131	4.342979464	up	8.52136E-09	7.75472E-08
G10090_31185	20715	Serpina3g	2.991093356	7.950763222	up	1.64736E-08	1.44243E-07
G10090_11102	20719	Serpinb6a	-1.182891672	0.440467759	down	1.72464E-33	1.42024E-31
G10090_16603	20720	Serpine2	3.211045264	9.260212268	up	0.000110028	0.000506272
G10090_10102	20723	Serpinb9	1.609745773	3.051980561	up	2.12534E-11	2.65381E-10
G10090_21089	20728	Spic	1.370457502	2.585525444	up	0.008771886	0.024576295
G10090_9574	20733	Spint2	1.843821215	3.589595336	up	1.80235E-08	1.56894E-07
G10090_3952	20741	Sptb	-1.10615361	0.464530874	down	0.019815493	0.049577513
G10090_15259	20779	Src	2.215739774	4.645196974	up	1.84511E-35	1.74415E-33
G10090_8688	20846	Stat1	1.537471011	2.902851985	up	1.14946E-26	5.65173E-25

G10090_24810	20856	Stc2	3.359201512	10.26172605	up	0.002384575	0.007876022
G10090_32427	20863	Stfa3	5.552913472	46.94545129	up	0.014170913	0.037282666
G10090_8383	20877	Aurkb	-1.265071651	0.416078702	down	0.007695485	0.021938167
G10090_23655	20887	Sult1a1	-5.03794157	0.030438866	down	8.65732E-27	4.30397E-25
G10090_19031	20933	Med22	-1.432191898	0.370567459	down	1.60372E-36	1.61855E-34
G10090_1508	20971	Sdc4	1.658898616	3.157753632	up	2.6175E-22	9.07875E-21
G10090_7926	20972	Syngr1	-1.799831238	0.287208183	down	4.65395E-21	1.46643E-19
G10090_23282	20981	Syt3	-5.715169758	0.01903542	down	0.000289584	0.001198978
G10090_4395	21353	Tank	1.045284277	2.063772976	up	1.72843E-18	4.37749E-17
G10090_130	21413	Tcf4	-1.241136248	0.423039345	down	1.38688E-29	8.69909E-28
G10090_3702	21425	Tfeb	-1.683456636	0.311335795	down	3.46218E-16	6.97791E-15
G10090_17086	21426	Tfec	1.587021733	3.00428511	up	3.74961E-28	2.11475E-26
G10090_29396	21664	Phlda1	4.834091749	28.52374979	up	9.89066E-37	1.04537E-34
G10090_7626	21667	Tdgf1	3.62418174	12.33069097	up	2.05389E-10	2.28223E-09
G10090_11339	21682	Tec	-1.253758214	0.419354367	down	2.29284E-19	6.28097E-18
G10090_829	21685	Tef	-1.234998666	0.424842892	down	2.93325E-40	3.54711E-38
G10090_21986	21753	Tes	1.224027999	2.335980125	up	1.87012E-36	1.85945E-34
G10090_18477	21812	Tgfbr1	-1.648857094	0.318892684	down	1.54961E-13	2.41584E-12
G10090_32992	21814	Tgfbr3	-2.994836168	0.125448214	down	0.008265339	0.023376664
G10090_18917	21816	Tgm1	3.058740209	8.332446844	up	2.14599E-11	2.67711E-10
G10090_9195	21817	Tgm2	1.089080283	2.127383727	up	1.56475E-07	1.18397E-06
G10090_20588	21824	Thbd	-1.867281528	0.274089406	down	6.57591E-09	6.08748E-08
G10090_13244	21825	Thbs1	3.650484414	12.55756131	up	1.28869E-05	7.13616E-05
G10090_5253	21844	Tiam1	-1.040760956	0.486071026	down	9.49803E-14	1.50344E-12
G10090_23208	21857	Timp1	2.518993639	5.731821329	up	0.001182041	0.004215384
G10090_32209	21858	Timp2	-2.643685689	0.16001891	down	6.56157E-28	3.6395E-26
G10090_1294	21885	Tle1	-1.300532102	0.405976436	down	1.3657E-16	2.85988E-15
G10090_3055	21923	Tnc	5.045268277	33.02000142	up	2.33317E-23	8.77258E-22
G10090_859	21926	Tnf	4.457927867	21.97708088	up	8.45955E-52	1.83149E-49
G10090_8775	21929	Tnfaip3	2.174583912	4.514555388	up	1.4953E-48	3.04113E-46
G10090_9494	21939	Cd40	3.092406053	8.529174125	up	7.20238E-24	2.81859E-22
G10090_4809	21942	Tnfrsf9	2.818528187	7.054423495	up	0.00439971	0.013501901
G10090_5141	21944	Tnfsf12	-1.027588391	0.490529433	down	2.29758E-08	1.9767E-07
G10090_24217	21949	Tnfsf8	-3.017982616	0.123451595	down	0.001385633	0.004846105
G10090_24219	21950	Tnfsf9	3.259188231	9.574440818	up	2.2186E-07	1.6435E-06
G10090_28306	21953	Tnni2	-1.860609292	0.275359962	down	0.009139742	0.025468705
G10090_4473	21961	Tns1	-3.993813497	0.062768585	down	5.99194E-43	8.93665E-41
G10090_21595	21983	Tpbg	2.924611815	7.592693731	up	8.54628E-05	0.000404217
G10090_30199	21991	Tpi1	1.604140348	3.04014545	up	8.96846E-29	5.27999E-27
G10090_15304	22027	Hsp90b1	1.049117964	2.069264352	up	3.493E-54	9.19343E-52
G10090_2880	22029	Traf1	2.243452976	4.735290636	up	7.41362E-82	6.21957E-79
G10090_394	22033	Traf5	1.299213323	2.460946549	up	1.94023E-17	4.52934E-16
G10090_6262	22041	Trf	-2.613076619	0.163450238	down	7.03372E-46	1.21043E-43
G10090_15857	22042	Tfrc	-1.779647078	0.291254637	down	8.76305E-11	1.01929E-09
G10090_6485	22066	Trpc4	-2.945155622	0.129843381	down	0.005922448	0.017452692
G10090_25327	22117	Tst	-1.009961652	0.496559446	down	0.008474562	0.023852808
G10090_21011	22142	Tuba1a	1.078193679	2.111390866	up	1.52643E-13	2.38247E-12
G10090_9742	22145	Tuba4a	1.401790972	2.64229395	up	8.03195E-19	2.09345E-17
G10090_6033	22153	Tubb4a	2.185747322	4.549624006	up	3.93091E-09	3.74749E-08
G10090_33199	22164	Tnfsf4	2.343547449	5.075491193	up	0.000154796	0.00068848
G10090_6787	22166	Txn1	1.462330524	2.755531317	up	8.29667E-17	1.77901E-15
G10090_9730	22201	Uba1	1.068528192	2.097292661	up	1.23431E-19	3.47339E-18
G10090_6661	22223	Uchl1	2.581622812	5.98612669	up	3.01318E-27	1.53204E-25
G10090_7188	22228	Ucp2	-1.300969889	0.405853261	down	4.07814E-17	9.1083E-16
G10090_33651	22236	Ugt1a2	-1.250007521	0.420446016	down	0.003090253	0.009909332
G10090_1531	22249	Unc13b	-2.480225679	0.179216369	down	1.07208E-16	2.27699E-15
G10090_9116	22253	Unc5c	7.68247096	205.4254277	up	1.47601E-07	1.12507E-06
G10090_20164	22271	Upp1	6.557963638	94.22014437	up	4.55214E-29	2.74006E-27
G10090_12169	22325	Vav2	-1.007835355	0.497291834	down	2.28106E-11	2.83506E-10

G10090_8180	22329	Vcam1	5.853565088	57.82274077	up	0.000235151	0.000997291
G10090_9264	22337	Vdr	1.615630935	3.064455883	up	0.004881276	0.014753741
G10090_4300	22339	Vegfa	1.379324618	2.601465578	up	0.000188623	0.000820708
G10090_17818	22341	Vegfc	1.634790287	3.105424056	up	4.94493E-15	8.79149E-14
G10090_10888	22354	Vipr1	-1.828231171	0.28160968	down	3.09237E-05	0.000159792
G10090_1951	22390	Wee1	-1.435579466	0.369698356	down	4.04886E-13	6.12025E-12
G10090_17593	22420	Wnt6	2.076044101	4.216494583	up	0.002240696	0.007444768
G10090_22251	22436	Xdh	-1.149630228	0.450740744	down	1.3975E-56	4.36248E-54
G10090_10357	22437	Xirp1	-6.112720438	0.014450664	down	3.23332E-05	0.000166287
G10090_26032	22439	Xk	-1.706174598	0.306471623	down	0.000105586	0.000487375
G10090_22675	22591	Xpc	-1.191023655	0.437991976	down	1.94073E-18	4.86016E-17
G10090_19631	22701	Zfp41	-1.064369591	0.478181562	down	0.000709466	0.002661588
G10090_21737	22751	Zfp90	-1.151181191	0.450256438	down	4.69109E-13	7.05134E-12
G10090_5453	23792	Adam23	2.456900131	5.490357647	up	0.000210687	0.000905847
G10090_27810	23845	Clec5a	1.912508081	3.764630005	up	2.61825E-18	6.48429E-17
G10090_223	23871	Ets1	2.949403038	7.72429379	up	1.55576E-08	1.36401E-07
G10090_18479	23872	Ets2	3.11984376	8.692937426	up	5.64323E-24	2.24775E-22
G10090_31750	23882	Gadd45g	-2.196433904	0.218176269	down	3.43267E-12	4.73554E-11
G10090_3845	23890	Gpr34	-5.695801169	0.019292699	down	5.58868E-15	9.85767E-14
G10090_15519	23893	Grem2	3.746913914	13.42559306	up	1.71651E-11	2.16549E-10
G10090_6614	23897	Hax1	1.075198994	2.107012676	up	1.03422E-27	5.62037E-26
G10090_5899	23945	Mgll	-1.604932351	0.328751105	down	0.000655673	0.00248759
G10090_31245	23959	Nt5e	1.755254086	3.375857684	up	0.000112327	0.000516074
G10090_21224	23960	Oas1g	1.33898502	2.529732817	up	3.58686E-09	3.43412E-08
G10090_8921	23962	Oasl2	1.384376946	2.6105919	up	4.33117E-05	0.000217255
G10090_15953	23969	Pacsin1	-3.744973443	0.074584857	down	0.000288358	0.001194272
G10090_13295	23972	Papss2	1.401712732	2.642150657	up	0.000238411	0.001008251
G10090_1514	23984	Pde10a	5.318987725	39.91855866	up	1.79083E-10	2.00486E-09
G10090_12849	24001	Tiam2	-1.069961182	0.476331815	down	0.014349888	0.037679685
G10090_4521	24014	Rnasel	1.398508268	2.636288516	up	3.60243E-19	9.69047E-18
G10090_1225	24056	Sh3bp5	-1.030623394	0.489498589	down	8.55748E-07	5.79844E-06
G10090_1806	24058	Sigirr	-2.024908286	0.24572077	down	0.001629485	0.005601172
G10090_14277	24066	Spry4	4.993315812	31.85208288	up	0.003412165	0.010819136
G10090_20960	24068	Sra1	1.111804938	2.161158581	up	3.69507E-17	8.32197E-16
G10090_9034	24088	Tlr2	1.019219631	2.026822334	up	1.51571E-05	8.30085E-05
G10090_22575	24110	Usp18	1.275280814	2.420459264	up	0.000228348	0.000972128
G10090_30409	24115	Best1	1.113708747	2.164012372	up	0.010188697	0.028019439
G10090_5558	26364	Adgre5	-1.667467251	0.314805521	down	4.43828E-12	6.00909E-11
G10090_1946	26382	Fgd2	-2.677579039	0.156303389	down	0.000182272	0.000796173
G10090_31603	26384	Gnpda1	-1.469265035	0.361166244	down	5.3797E-32	4.05684E-30
G10090_18511	26386	Hsf4	-1.337874601	0.395603035	down	0.000115008	0.000526696
G10090_11259	26399	Map2k6	2.013147493	4.036619197	up	0.000410379	0.001639439
G10090_13980	26401	Map3k1	-1.106711656	0.464351224	down	1.93707E-09	1.92317E-08
G10090_10870	26411	Map4k1	-1.192783034	0.437458167	down	0.000220087	0.000942036
G10090_16574	26415	Mapk13	1.65755388	3.15481166	up	0.010244988	0.028162701
G10090_3397	26443	Psma6	1.103208972	2.148320101	up	1.94685E-18	4.86641E-17
G10090_32309	26464	Vnn3	4.77217531	27.32548713	up	1.00034E-26	4.95483E-25
G10090_21039	26569	Slc27a4	1.295366093	2.454392693	up	3.94266E-14	6.49353E-13
G10090_12240	26570	Slc7a11	2.91640901	7.549646061	up	3.17918E-19	8.56911E-18
G10090_33596	26878	B3galt2	5.476390005	44.52025667	up	4.55843E-16	9.0783E-15
G10090_20163	26886	Cenph	-1.755043552	0.296264249	down	0.010146434	0.027926098
G10090_32965	26897	Acot1	-2.65607129	0.158651021	down	2.26542E-07	1.67633E-06
G10090_17113	26934	Racgap1	-1.580137017	0.334450124	down	0.000285948	0.001185386
G10090_4946	27029	Sgsh	-2.049541052	0.241560915	down	1.44144E-18	3.68543E-17
G10090_4327	27052	Aoah	1.436136446	2.705952371	up	3.65985E-06	2.21389E-05
G10090_22051	27053	Asns	1.967996177	3.912243528	up	7.1322E-42	9.67025E-40
G10090_2615	27218	Slamf1	4.14137097	17.64724379	up	0.0004568	0.001804479
G10090_9404	27275	Nufip1	1.028010782	2.039210607	up	5.72591E-19	1.51E-17
G10090_31662	27280	Phlda3	-1.650751174	0.318474292	down	0.001711	0.005842471

G10090_1879	27355	Pald1	-2.511156055	0.17541499	down	1.48061E-23	5.64609E-22
G10090_4035	27373	Csnk1e	-1.511235757	0.350810599	down	2.1573E-22	7.54098E-21
G10090_20121	27401	Skp2	-1.023081956	0.492064057	down	7.68988E-06	4.41494E-05
G10090_6683	27404	Abca8b	-1.730759301	0.301293342	down	0.000120979	0.000550661
G10090_7700	27416	Abcc5	1.888041679	3.70132464	up	4.73597E-42	6.62197E-40
G10090_21152	27494	Amot	-1.28174735	0.411297056	down	0.000727735	0.002715652
G10090_6997	28010	Miip	-1.018186204	0.493736702	down	0.000356668	0.001446827
G10090_6115	28105	Trim36	-1.520024289	0.348680046	down	0.001124665	0.0040257
G10090_3312	28240	Trpm2	1.11290209	2.162802741	up	0.004479374	0.013711889
G10090_14735	29813	Zfp385a	-1.009966063	0.496557928	down	9.5868E-08	7.52976E-07
G10090_10624	29816	Hip1r	3.991371756	15.90459529	up	4.78478E-06	2.8381E-05
G10090_18800	29817	Igfbp7	2.133118258	4.386645912	up	0.017666775	0.044947141
G10090_699	29870	Gtse1	-1.137205613	0.454639326	down	0.018316242	0.046379725
G10090_7499	29877	Hdgfl3	-1.197542318	0.43601742	down	4.45267E-11	5.34398E-10
G10090_474	30841	Kdm2b	-1.272505772	0.413940188	down	1.05161E-13	1.65678E-12
G10090_17736	30925	Slamf6	2.488883822	5.613434845	up	2.54576E-07	1.86935E-06
G10090_16196	30939	Pttg1	-1.137981969	0.454394737	down	0.001151306	0.004112288
G10090_466	30953	Schip1	6.266626185	76.9914419	up	9.87144E-06	5.58854E-05
G10090_16012	50493	Txnrd1	1.753737787	3.372311461	up	1.78776E-14	3.0223E-13
G10090_32707	50527	Ero1l	1.978762414	3.941548192	up	1.19069E-22	4.23941E-21
G10090_15395	50706	Postn	-3.627039046	0.080937997	down	0.000198223	0.000858583
G10090_185	50768	Dlc1	4.417039023	21.36295068	up	0.000853565	0.003138079
G10090_15764	50782	Rgs11	1.004071057	2.005651654	up	0.01438141	0.037747685
G10090_9810	50790	Acsl4	1.003642553	2.005056031	up	3.14202E-15	5.73035E-14
G10090_10008	50873	Prkn	-1.886052488	0.270546318	down	3.36848E-09	3.24891E-08
G10090_12033	50883	Chek2	-1.640480538	0.32074962	down	5.66292E-07	3.95491E-06
G10090_20662	50884	Nckap1	1.204510782	2.304591074	up	5.23622E-08	4.27789E-07
G10090_8691	50908	C1s1	1.012511063	2.017419437	up	0.003559028	0.011202268
G10090_27586	50930	Tnfsf14	3.351403432	10.20640883	up	2.68285E-11	3.30081E-10
G10090_4908	50931	Il27ra	-1.217351621	0.430071483	down	0.00032244	0.001321964
G10090_6056	50997	Mpp2	4.50664345	22.73185402	up	6.17171E-17	1.35364E-15
G10090_1638	51801	Ramp1	-5.117201126	0.028811706	down	2.28149E-08	1.96563E-07
G10090_13013	51902	Rnf24	1.061095707	2.086515601	up	8.08948E-07	5.50189E-06
G10090_8677	51944	Knstrn	-1.717500121	0.304075162	down	0.002268179	0.007532351
G10090_14800	52055	Rab11fip5	-1.185916122	0.439545335	down	5.17829E-28	2.90829E-26
G10090_22664	52118	Pvr	1.569939591	2.968922823	up	1.95955E-29	1.21212E-27
G10090_25876	52163	Camk1	-1.718030449	0.303963405	down	7.46697E-13	1.10142E-11
G10090_32926	52187	Rragd	-1.573291379	0.336040872	down	6.13055E-06	3.56235E-05
G10090_14655	52463	Tet1	4.489049863	22.45632375	up	7.01537E-16	1.37873E-14
G10090_9979	52538	Acaa2	-1.083128918	0.47200403	down	3.88222E-11	4.69468E-10
G10090_14371	52570	Ccdc69	-1.472745703	0.360295939	down	0.000105287	0.000486327
G10090_17169	52609	Cbx7	-1.518365729	0.349081128	down	7.05911E-10	7.43403E-09
G10090_24852	52614	Adgre4	-1.212793936	0.43143229	down	0.000222678	0.000951913
G10090_5185	52685	Cd300lg	-3.674648629	0.078310598	down	2.7216E-08	2.31362E-07
G10090_199	52696	Zwint	1.269782526	2.411252152	up	4.75558E-08	3.93067E-07
G10090_842	52822	Rufy3	-1.184077864	0.440105753	down	3.92974E-20	1.14671E-18
G10090_7377	52906	Ahi1	1.100755903	2.144670335	up	3.75418E-10	4.08366E-09
G10090_14339	53310	Dlg3	-1.108897349	0.463648262	down	0.016401847	0.042176626
G10090_29452	53314	Batf	2.064828742	4.183843079	up	1.34224E-23	5.14768E-22
G10090_2039	53374	Chst3	-2.070402605	0.238093047	down	0.000186759	0.000813653
G10090_7851	53376	Usp2	-1.524615425	0.347572194	down	2.99967E-06	1.84277E-05
G10090_20116	53599	Cd164	1.561861839	2.952346064	up	6.37824E-17	1.39212E-15
G10090_7502	53791	Tlr5	-1.796812675	0.287809741	down	0.001462046	0.00508289
G10090_11366	53867	Col5a3	3.318731476	9.977867255	up	8.965E-07	6.05928E-06
G10090_14724	53881	Slc5a3	-2.183179893	0.220189886	down	5.26221E-15	9.33087E-14
G10090_22794	53945	Slc40a1	-1.452422918	0.365407229	down	9.53576E-07	6.41546E-06
G10090_18394	54123	Irf7	1.970913925	3.920163766	up	0.001951768	0.006571
G10090_12369	54135	Lsr	-1.463413279	0.362634156	down	1.46671E-10	1.65304E-09
G10090_20967	54137	Acrbp	-1.105704636	0.464675461	down	0.00530705	0.01586913

G10090_2868	54141	Spag5	-1.133446318	0.455825545	down	0.019107552	0.048077467
G10090_3771	54167	Icos	-5.007677896	0.031084132	down	0.000512946	0.002005613
G10090_15051	54199	Ccrl2	1.879492886	3.679457029	up	1.84011E-28	1.05106E-26
G10090_10805	54352	Irx5	4.351369577	20.41233866	up	0.011658617	0.031424422
G10090_7316	54378	Cacng6	4.797817436	27.81550582	up	0.003081025	0.00989027
G10090_12639	54427	Dnmt3l	2.424586609	5.368751424	up	2.81769E-09	2.75669E-08
G10090_18002	54448	Il1f6	7.302654905	157.8767488	up	1.22134E-08	1.08355E-07
G10090_10198	54483	Mefv	1.552692683	2.93364171	up	2.11117E-11	2.63856E-10
G10090_4781	54486	Hpgds	-1.928710196	0.262663894	down	2.17093E-19	5.98365E-18
G10090_1410	54524	Syt6	-4.832342161	0.035101046	down	0.000416517	0.001660503
G10090_10628	54525	Syt7	5.483447864	44.7385894	up	3.95497E-07	2.8238E-06
G10090_8980	54607	Socs6	-1.156822273	0.448499327	down	8.23459E-17	1.77136E-15
G10090_9793	54635	Pdgfc	-4.274190517	0.051682135	down	8.44628E-21	2.61835E-19
G10090_11510	54637	Praf2	1.387958678	2.617081183	up	1.39262E-24	5.76949E-23
G10090_2519	54672	Adgrg3	-1.935550566	0.261421451	down	0.008428264	0.023749457
G10090_28643	54683	Prdx5	2.407754968	5.306479215	up	5.03877E-48	9.80223E-46
G10090_19259	54712	Plxnc1	-2.423196164	0.186442651	down	7.54242E-16	1.47368E-14
G10090_20171	54725	Cadm1	-2.454687562	0.182417044	down	6.61698E-29	3.96517E-27
G10090_1642	55932	Gbp3	3.306157252	9.891280165	up	3.21957E-27	1.62467E-25
G10090_10829	55984	Camkk1	3.409059392	10.62255856	up	0.003253554	0.010388311
G10090_15227	55994	Smad9	-4.719813067	0.037948507	down	0.011050912	0.030076317
G10090_30322	56009	Alyref2	-1.149739016	0.450706757	down	0.000515623	0.002014906
G10090_17409	56079	Astn2	3.648279174	12.53838104	up	0.0083317	0.023534597
G10090_30236	56193	Plek	1.85229256	3.610735046	up	3.47745E-31	2.43114E-29
G10090_33654	56213	Htra1	1.33645122	2.525293756	up	0.003524024	0.011117033
G10090_2641	56226	Espn	-2.895472751	0.134392753	down	0.002035091	0.00682755
G10090_6598	56229	Thsd1	-1.47118194	0.360686682	down	0.008472469	0.023851919
G10090_23765	56295	Higd1a	1.110109877	2.158620869	up	2.20827E-06	1.38967E-05
G10090_8615	56312	Nupr1	1.556043498	2.940463328	up	4.6466E-15	8.29406E-14
G10090_1029	56318	Acpp	2.611048899	6.109477065	up	1.98907E-34	1.7919E-32
G10090_1316	56338	Txnip	-1.238921426	0.423689292	down	2.76907E-45	4.64615E-43
G10090_18042	56348	Hsd17b12	1.275631531	2.421047745	up	5.51026E-24	2.20132E-22
G10090_23978	56405	Dusp14	3.040804414	8.229497918	up	0.000588246	0.002266368
G10090_32392	56410	Cbln3	3.821650718	14.13941688	up	1.16121E-11	1.48447E-10
G10090_8020	56464	Ctsf	-1.451956962	0.365525266	down	1.1998E-08	1.06584E-07
G10090_556	56526	Sept6	-1.416032508	0.374741456	down	0.00012927	0.000585422
G10090_20644	56532	Ripk3	1.151471438	2.22140345	up	2.48507E-17	5.70207E-16
G10090_25117	56613	Rps6ka4	1.390112692	2.62099153	up	5.36422E-33	4.33758E-31
G10090_12275	56619	Clec4e	3.839663637	14.31706273	up	2.83031E-44	4.57726E-42
G10090_16358	56620	Clec4n	2.947559866	7.714431619	up	2.65163E-06	1.64325E-05
G10090_5236	56696	Gpr132	1.015774834	2.021988559	up	0.002452154	0.008073401
G10090_7063	56711	Plag1	-1.264673489	0.41619355	down	4.48618E-13	6.76607E-12
G10090_9580	56742	Psrc1	-2.51013044	0.175539737	down	7.47947E-05	0.00035665
G10090_18155	56744	Pf4	3.804900329	13.97620073	up	5.64896E-11	6.6984E-10
G10090_32633	56753	Tacstd2	4.444441003	21.77258807	up	0.00060816	0.002331714
G10090_961	56758	Mbnl1	-1.326389329	0.398764994	down	1.45112E-15	2.73957E-14
G10090_10779	56791	Ube2l6	1.332852781	2.519002896	up	0.000808318	0.002981602
G10090_25396	56857	Slc37a2	-2.553228538	0.170373335	down	5.94097E-06	3.46419E-05
G10090_12344	57248	Ly6i	2.729291855	6.631300601	up	2.68248E-25	1.17286E-23
G10090_15725	57278	Bcam	2.419720357	5.350672978	up	0.007638404	0.021798256
G10090_26035	57349	Ppbp	5.42563488	42.98123046	up	2.9852E-21	9.5862E-20
G10090_4167	57442	Kcne3	-3.147408666	0.11285884	down	0.002146112	0.007158862
G10090_21820	57740	Stk32c	-1.705981465	0.306512653	down	0.002321798	0.007685695
G10090_17474	57746	Piwil2	2.5847276	5.999023154	up	1.22875E-14	2.10109E-13
G10090_11669	57778	Fmnl1	-1.011797166	0.495928085	down	3.4599E-15	6.29298E-14
G10090_9575	57783	Tnip1	1.479981184	2.789450952	up	4.52355E-91	5.05997E-88
G10090_4658	57785	Rangrf	1.184982787	2.27360682	up	0.005587064	0.016617583
G10090_21186	57875	Angptl4	-2.089659348	0.234936154	down	1.73631E-07	1.30862E-06
G10090_6140	58203	Zbp1	2.594000632	6.03770652	up	5.88334E-25	2.49911E-23

G10090_7602	58217	Trem1	3.596257498	12.09431787	up	3.8123E-06	2.29782E-05
G10090_13584	58218	Trem3	3.534155728	11.5847558	up	1.21261E-10	1.37823E-09
G10090_15025	58250	Chst11	2.610600645	6.107579106	up	2.72493E-52	6.30634E-50
G10090_22046	58251	Cep295nl	-3.323359678	0.099900819	down	0.001310769	0.004610707
G10090_18406	58809	Rnase4	-2.808037956	0.142789524	down	1.16377E-55	3.32367E-53
G10090_17176	58861	Cysltr1	1.04496076	2.063310237	up	2.00301E-09	1.97985E-08
G10090_367	58994	Smpd3	-5.853681166	0.017292842	down	0.000404108	0.001616794
G10090_12608	59010	Sqor	1.20938756	2.312394522	up	1.36105E-07	1.04396E-06
G10090_31578	59014	Rrs1	1.287019694	2.440234331	up	8.60353E-21	2.66095E-19
G10090_12807	59027	Nampt	1.158030223	2.231525392	up	1.88087E-13	2.90864E-12
G10090_13057	59028	Rcl1	2.388270977	5.235295522	up	1.56077E-33	1.30939E-31
G10090_5800	59030	Mkks	1.181848251	2.268672328	up	4.70355E-15	8.38456E-14
G10090_30787	60533	Cd274	2.116855529	4.337475262	up	1.91014E-91	2.33089E-88
G10090_12209	60599	Trp53inp1	-1.6984573	0.308115401	down	1.3144E-13	2.06353E-12
G10090_11596	63913	Fam129a	-2.513359938	0.175147228	down	1.6746E-31	1.20851E-29
G10090_21782	63955	Cables1	-3.313344973	0.10059671	down	0.000673381	0.002547574
G10090_12955	64008	Aqp9	4.357197993	20.49497028	up	4.47028E-17	9.93454E-16
G10090_14680	64051	Sv2a	-1.976892763	0.254036418	down	1.58444E-06	1.02004E-05
G10090_31847	64136	Sdf2l1	1.146459276	2.213699316	up	7.6902E-10	8.04564E-09
G10090_24422	64292	Ptges	3.962438901	15.58881005	up	2.57659E-35	2.40177E-33
G10090_11013	64450	Gpr85	1.541564155	2.911099508	up	5.09851E-08	4.1781E-07
G10090_11012	64451	Dip2a	1.232892772	2.350377959	up	3.22168E-12	4.47203E-11
G10090_28421	64705	Dpys	1.045734588	2.064417247	up	0.000204031	0.000880896
G10090_19242	65099	Irak1bp1	2.329525639	5.026400536	up	1.42469E-07	1.08966E-06
G10090_29671	65221	Slc15a3	1.843846751	3.589658873	up	2.28852E-38	2.62554E-36
G10090_11185	65962	Slc9a3r2	-1.615171948	0.326426039	down	0.006524307	0.018992794
G10090_11865	65963	Tmem176b	-1.23156475	0.425855312	down	0.013704618	0.036169306
G10090_3111	65970	Lima1	-1.007850933	0.497286464	down	2.21576E-12	3.12747E-11
G10090_2127	66049	Rogdi	-1.537800821	0.344410058	down	3.37428E-09	3.25147E-08
G10090_30607	66066	Gng11	-1.093403611	0.468654418	down	0.003067066	0.009853813
G10090_32018	66107	Wfdc21	6.100114802	68.59896011	up	1.72679E-08	1.50707E-07
G10090_24655	66109	Tspan13	-1.911778224	0.26576477	down	2.67314E-07	1.95434E-06
G10090_11913	66120	Fkbp11	1.8710259	3.657926027	up	6.46594E-06	3.74751E-05
G10090_33327	66141	Ifitm3	1.198332551	2.294742941	up	4.89618E-16	9.7221E-15
G10090_29059	66143	Eef1e1	1.157743824	2.231082442	up	3.33252E-10	3.63382E-09
G10090_1753	66168	Grina	1.239915247	2.361846568	up	9.72395E-06	5.50969E-05
G10090_11054	66183	Sptssb	4.430535312	21.56373697	up	0.005291514	0.015836788
G10090_26114	66205	Cd302	1.324123792	2.50380775	up	6.93278E-22	2.33816E-20
G10090_28041	66220	Zdhhc12	1.096852225	2.13887508	up	7.06131E-10	7.43403E-09
G10090_18090	66235	Eif1ax	1.013604423	2.018948938	up	4.62702E-19	1.23722E-17
G10090_7643	66279	Tmem218	-1.031650263	0.489150302	down	2.2486E-05	0.0001193
G10090_21658	66282	Tma16	4.097181625	17.11490792	up	1.97679E-77	1.56085E-74
G10090_23594	66329	Susd3	-1.958645826	0.257269828	down	2.1674E-16	4.45528E-15
G10090_27220	66341	Eid3	1.47015267	2.770512104	up	0.000667756	0.002528431
G10090_1703	66395	Ahnak	-1.303346509	0.40518523	down	7.37761E-28	4.07529E-26
G10090_22279	66425	Pcp4l1	-3.864621689	0.0686488	down	1.89575E-07	1.41922E-06
G10090_23067	66431	Oxld1	-1.170597268	0.444237391	down	0.000622445	0.002379685
G10090_8492	66441	Magohb	1.084982216	2.121349333	up	0.002311165	0.007656162
G10090_29768	66447	Mgst3	-1.133281511	0.455877619	down	0.000105394	0.000486653
G10090_24840	66537	Pomp	1.0242449	2.033894575	up	2.26405E-17	5.2217E-16
G10090_9733	66540	Fam107b	-1.288558883	0.409359737	down	9.81665E-09	8.85543E-08
G10090_3885	66573	Dzip1	-3.930123469	0.065601678	down	0.018353151	0.046429391
G10090_654	66597	Trim13	1.15867929	2.232529579	up	3.62698E-12	4.96785E-11
G10090_3045	66599	Rdm1	-1.403168259	0.3780979	down	0.002465298	0.008110708
G10090_7992	66628	Thg1l	1.109687275	2.157988647	up	7.05187E-09	6.48781E-08
G10090_4002	66665	Msantd3	2.154918611	4.453435228	up	1.10938E-36	1.15436E-34
G10090_19008	66725	Lrrk2	1.213319156	2.318704795	up	1.10599E-06	7.3348E-06
G10090_482	66748	Erich2	5.582873142	47.93053513	up	0.000516177	0.002016487
G10090_10733	66775	Hacd4	-3.337356367	0.098936291	down	6.76362E-26	3.13062E-24

G10090_11728	66790	Grtp1	-1.56825228	0.33721666	down	0.010204515	0.028057189
G10090_19831	66859	Slc16a9	2.303370512	4.936096202	up	2.52605E-05	0.000132761
G10090_19825	66861	Dnajc10	1.221952163	2.332621393	up	2.89616E-23	1.07688E-21
G10090_13879	66895	Pxdc1	2.539219874	5.812746023	up	3.09276E-05	0.000159792
G10090_8381	66949	Trim59	-1.235826785	0.424599099	down	0.000442812	0.001755777
G10090_6937	66970	Ssbp2	-1.115041836	0.461677767	down	2.59134E-10	2.85345E-09
G10090_27105	66977	Nuf2	-1.926849344	0.263002908	down	0.000137924	0.000618146
G10090_13664	67036	Mrpl45	1.656052997	3.151531313	up	5.08302E-61	1.94941E-58
G10090_4778	67052	Ndc80	-1.890563756	0.269701648	down	0.010783116	0.02949324
G10090_19044	67065	Polr3d	1.021089599	2.029451128	up	1.00596E-10	1.15905E-09
G10090_5621	67102	D16Ertd472e	2.376643137	5.193269652	up	1.59802E-23	6.05938E-22
G10090_1188	67103	Ptgr1	1.638212166	3.112798447	up	3.42336E-11	4.16985E-10
G10090_11481	67111	Naaa	1.363795505	2.573613672	up	6.05847E-12	8.09989E-11
G10090_11717	67121	Mastl	-1.277514946	0.412505441	down	0.003257199	0.010397475
G10090_22052	67138	Herc6	1.107487606	2.154700881	up	6.79221E-07	4.68028E-06
G10090_6744	67149	Nkain1	-1.239457449	0.423531903	down	9.18914E-07	6.2014E-06
G10090_32190	67168	Lpar6	-1.115854928	0.461417642	down	7.79327E-12	1.01958E-10
G10090_15813	67177	Cdt1	-1.509891275	0.351137681	down	5.78623E-17	1.27117E-15
G10090_292	67182	Pdzk1ip1	-2.579582985	0.167289293	down	1.15932E-10	1.32101E-09
G10090_4701	67260	Cers4	-1.387449199	0.382240036	down	0.00277585	0.009034976
G10090_5414	67399	Pdlim7	1.280591878	2.429386242	up	5.46351E-17	1.20224E-15
G10090_7797	67448	Plxdc2	-1.002424325	0.499160499	down	0.0007288	0.002718166
G10090_19286	67477	Abhd15	-1.696208177	0.308596119	down	8.77455E-21	2.7076E-19
G10090_4599	67492	Zfand4	-1.338534911	0.395422012	down	2.95779E-06	1.81788E-05
G10090_13072	67547	Slc39a8	-1.733878044	0.300642725	down	5.21285E-09	4.88291E-08
G10090_695	67554	Slc25a30	1.954138042	3.874843497	up	3.49257E-12	4.79354E-11
G10090_11841	67590	Tctn3	-1.201809883	0.434729565	down	7.31437E-17	1.58612E-15
G10090_18496	67603	Dusp6	1.112054135	2.161531911	up	0.001123394	0.004022225
G10090_10051	67647	4930523C07Rik	-1.624649629	0.324288635	down	0.005115113	0.015377417
G10090_12484	67704	1810037I17Rik	1.226991804	2.340783984	up	2.88997E-16	5.8865E-15
G10090_24794	67712	Slc25a37	1.155389201	2.227444062	up	7.74703E-08	6.1861E-07
G10090_6072	67725	Nudt13	-1.066374684	0.477517435	down	5.26995E-09	4.92952E-08
G10090_12332	67742	Samsn1	1.781979226	3.438976426	up	1.56136E-10	1.75381E-09
G10090_30470	67749	Mgarp	3.692047662	12.92459942	up	2.0214E-07	1.50657E-06
G10090_6198	67775	Rtp4	1.713494163	3.279541571	up	7.08564E-08	5.68843E-07
G10090_27403	67776	Vwa5a	-1.070497986	0.476154613	down	9.50102E-13	1.38773E-11
G10090_13602	67784	Plxnd1	-1.077834501	0.473739377	down	9.82844E-08	7.69703E-07
G10090_17211	67865	Rgs10	-1.476670489	0.359317103	down	6.20253E-37	6.60767E-35
G10090_3566	67893	Tmem86a	-1.194291586	0.437000978	down	1.63581E-20	4.90123E-19
G10090_17528	67895	Ppa1	1.879609105	3.679753446	up	3.15571E-16	6.40833E-15
G10090_4258	67916	Plpp3	2.974109634	7.857713865	up	7.92901E-30	5.11688E-28
G10090_6245	67972	Atp2b1	-1.388860142	0.381866392	down	1.72923E-25	7.76302E-24
G10090_6616	67991	Nacc2	-1.184166122	0.44007883	down	7.50537E-18	1.7958E-16
G10090_2005	68010	Bambi	-1.362538208	0.38889748	down	0.003569574	0.01122117
G10090_13572	68027	Tmem178	1.98368018	3.955006813	up	9.16217E-05	0.000429863
G10090_27156	68067	Mrnip	-1.566339591	0.33766403	down	0.002952209	0.009530424
G10090_1403	68070	Pdzd2	2.538415215	5.809504885	up	3.40835E-06	2.0739E-05
G10090_20651	68153	Gtf2e2	1.016601545	2.023147556	up	1.15798E-15	2.22051E-14
G10090_28294	68176	Inka1	-1.375018861	0.385547666	down	0.003858243	0.012046801
G10090_26355	68184	Denr	1.425595509	2.686253597	up	1.08428E-20	3.32291E-19
G10090_12028	68262	Agpat4	1.06068304	2.085918861	up	3.46517E-11	4.21696E-10
G10090_5902	68279	Mcoln2	1.608287275	3.048896709	up	6.79714E-22	2.29819E-20
G10090_12901	68298	Ncapd2	-1.020215675	0.49304264	down	0.004904742	0.014814662
G10090_15473	68303	Fam114a1	1.203263288	2.302599162	up	5.13824E-13	7.68047E-12
G10090_27969	68304	Kdelc2	-1.129722959	0.457003475	down	2.10487E-06	1.32896E-05
G10090_5459	68318	Aph1c	-1.560333145	0.339072775	down	1.12853E-56	3.60671E-54
G10090_1856	68385	Tlcd1	-1.278380075	0.412258151	down	0.003060425	0.009834829
G10090_11684	68428	Steap3	-1.628065661	0.323521689	down	3.47644E-12	4.78118E-11
G10090_24386	68487	Tmem140	-1.286670736	0.409895843	down	4.5331E-10	4.89523E-09

G10090_12429	68499	Mrpl53	1.148581064	2.216957424	up	1.12833E-10	1.28789E-09
G10090_7769	68519	Eml1	2.652721139	6.288522701	up	0.00047876	0.001886234
G10090_21673	68567	Cgref1	2.045529631	4.128247969	up	6.47547E-06	3.75142E-05
G10090_22084	68659	Fam198b	-1.744261009	0.29848679	down	1.06647E-05	6.00725E-05
G10090_12020	68703	Rere	-1.110599452	0.463101568	down	3.95252E-15	7.1406E-14
G10090_11303	68713	Ifitm1	4.028525314	16.3195041	up	1.47774E-27	7.89198E-26
G10090_4114	68732	Carmil1	1.338921134	2.529620798	up	7.61247E-22	2.55456E-20
G10090_24166	68738	Acss1	-1.903356916	0.26732063	down	7.32782E-05	0.00035004
G10090_658	68743	Anln	-1.901017493	0.267754459	down	4.1451E-05	0.000208701
G10090_25193	68774	Ms4a6d	1.447470001	2.727293567	up	1.9889E-13	3.06861E-12
G10090_21800	68777	Tmem53	4.36841665	20.65496414	up	8.99949E-05	0.000423118
G10090_29256	68797	Pdgfrl	-1.946729449	0.259403626	down	0.005868518	0.017320386
G10090_22325	68836	Mrpl52	1.088716386	2.126847196	up	2.48994E-11	3.07757E-10
G10090_40	68857	Dtwd2	-1.026338674	0.490954532	down	0.004823706	0.014604626
G10090_29519	68876	Atp23	-1.616112127	0.326213382	down	3.2926E-05	0.000169103
G10090_26196	69065	Chac1	2.085183258	4.243289935	up	1.34434E-06	8.77679E-06
G10090_2317	69185	Dtwd1	1.275546015	2.420904242	up	2.95772E-09	2.88457E-08
G10090_33663	69189	Mcemp1	1.359748037	2.56640354	up	0.006061286	0.017830516
G10090_22922	69310	Pacrg	-2.700702571	0.153818126	down	8.29402E-08	6.57983E-07
G10090_7434	69325	1700012B09Rik	5.700492912	52.00191733	up	0.000605922	0.002325128
G10090_29361	69524	Esam	3.379066292	10.40399923	up	1.55517E-11	1.97306E-10
G10090_5551	69538	Antxr1	3.379868099	10.40978307	up	1.1056E-16	2.34077E-15
G10090_618	69550	Bst2	1.387767649	2.616734674	up	3.17441E-17	7.18552E-16
G10090_25633	69573	Hilpda	2.499333106	5.654239942	up	1.74315E-14	2.95061E-13
G10090_23994	69574	Cmbl	-3.124891682	0.114634112	down	5.66369E-05	0.000277966
G10090_21560	69581	Rhou	2.339234062	5.060339091	up	3.66678E-09	3.50564E-08
G10090_5324	69583	Tnfsf13	-2.369204047	0.19355238	down	2.23252E-27	1.16151E-25
G10090_7094	69656	Pir	1.012414358	2.017284213	up	4.93818E-07	3.48686E-06
G10090_32370	69716	Trip13	2.010439855	4.029050407	up	1.61992E-14	2.74896E-13
G10090_18398	69743	Casz1	1.011174903	2.015551859	up	0.012038671	0.032312554
G10090_30832	69747	Zswim7	1.299857439	2.462045526	up	2.61319E-08	2.22851E-07
G10090_12937	69810	Clec4b1	-2.639977635	0.160430724	down	0.019575557	0.04909617
G10090_4997	69890	Zfp219	-1.041658146	0.48576884	down	3.45517E-12	4.7568E-11
G10090_33981	69942	Rnf113a1	1.103063879	2.148104055	up	0.004753734	0.014426719
G10090_26117	69987	Spaca9	-1.024042346	0.491736604	down	0.011254316	0.030561739
G10090_28430	70021	Nt5dc2	-2.032956957	0.244353732	down	1.0962E-29	6.9736E-28
G10090_10544	70045	2610528A11Rik	-2.066116558	0.23880144	down	2.26131E-05	0.00011988
G10090_14921	70110	Ifi35	1.254283393	2.385486287	up	2.76237E-07	2.01299E-06
G10090_11599	70122	Mllt3	-1.463898434	0.362512228	down	7.22922E-05	0.000345576
G10090_13943	70152	Mettl7a1	-1.492868259	0.355305454	down	2.2932E-18	5.70031E-17
G10090_8251	70166	Lipn	-3.164717267	0.111512917	down	4.86419E-11	5.79343E-10
G10090_32679	70186	Fam162a	2.523131749	5.748285625	up	3.30364E-12	4.58108E-11
G10090_14155	70208	Med23	1.427305002	2.689438506	up	6.86193E-14	1.10177E-12
G10090_3716	70218	Kif18b	-1.916568795	0.264883743	down	0.007397264	0.021184867
G10090_6461	70355	Gprc5c	-5.339492919	0.02469747	down	7.26817E-54	1.87617E-51
G10090_13235	70381	Tecpr1	-1.04447148	0.484822487	down	9.32419E-11	1.07988E-09
G10090_15412	70433	Draxin	5.903853508	59.87382394	up	0.000186628	0.000813345
G10090_3753	70536	Qpct	1.649256405	3.136719245	up	6.1748E-07	4.28628E-06
G10090_19463	70546	Zdhhc2	1.881681594	3.685043357	up	1.36968E-07	1.04998E-06
G10090_670	70551	Tmtc4	-1.192002685	0.43769485	down	0.002390101	0.007890391
G10090_514	70552	Lrrc56	-1.000691047	0.499760559	down	0.000500064	0.001960294
G10090_25207	70556	Slc25a33	1.54698164	2.922051569	up	1.49208E-07	1.13603E-06
G10090_356	70561	Txndc16	-1.500825908	0.353351048	down	9.5526E-22	3.18966E-20
G10090_13971	70598	Filip1	-2.418523769	0.187047454	down	0.01078394	0.02949324
G10090_4660	70615	Ankrd24	1.256628312	2.389366747	up	1.49384E-07	1.13672E-06
G10090_2468	70652	Tmem144	-1.443887532	0.367575488	down	6.52512E-08	5.26362E-07
G10090_8810	70686	Dusp16	2.938749313	7.667463072	up	3.2297E-39	3.83648E-37
G10090_5309	70719	Arhgap45	-1.244511001	0.422050927	down	4.68825E-25	2.017E-23
G10090_501	70727	Rasgef1a	-1.996964165	0.250526624	down	0.001413963	0.004930772

G10090_1229	70737	Cgn	-3.289357417	0.102283305	down	1.59279E-05	8.67696E-05
G10090_7641	70839	P2ry12	-1.049881658	0.483007783	down	0.001352804	0.004746128
G10090_7126	70859	Lrrc63	2.034321624	4.0963007	up	0.001081764	0.003897081
G10090_25187	70945	Mmrn1	3.590318562	12.04463326	up	0.015426144	0.040043538
G10090_1974	71085	Arhgap19	-3.762530164	0.073682705	down	4.97295E-42	6.88164E-40
G10090_4424	71198	Otud1	-1.65288046	0.3180046	down	4.15139E-06	2.49214E-05
G10090_8553	71233	Enkur	1.289382083	2.444233448	up	0.010021857	0.027622873
G10090_17302	71306	Mfap3l	1.505363537	2.838961998	up	1.60196E-12	2.29245E-11
G10090_21897	71326	Treml1	-2.517832223	0.174605121	down	0.00016262	0.000719223
G10090_24527	71436	Flrt3	2.763158928	6.788811031	up	6.68746E-22	2.26681E-20
G10090_10418	71566	Clmp	1.13644966	2.198393525	up	1.28222E-06	8.42035E-06
G10090_7944	71592	Pogk	1.024965506	2.03491073	up	3.51723E-08	2.95999E-07
G10090_11793	71643	Zgrf1	-1.069026829	0.476640409	down	0.000255048	0.001069512
G10090_17655	71687	Tmem25	-1.304366361	0.404898903	down	1.15707E-05	6.47408E-05
G10090_14696	71706	Slc46a3	-4.170922379	0.055517162	down	1.31929E-27	7.112E-26
G10090_5134	71720	Osbpl3	1.712588685	3.277483881	up	1.27226E-23	4.90733E-22
G10090_33396	71733	Susd2	5.546352297	46.73243471	up	1.34677E-47	2.54615E-45
G10090_12119	71738	Mamdc2	-5.17562569	0.027668233	down	0.000121304	0.000551765
G10090_13717	71740	Nectin4	-1.614418166	0.326596635	down	1.51934E-07	1.15351E-06
G10090_19103	71761	Amdhd1	-2.22117435	0.214466713	down	0.008544633	0.024009757
G10090_31489	71781	Slc16a14	-5.18543252	0.027480794	down	0.002761447	0.008994637
G10090_9403	71803	Slc25a18	2.251646835	4.762261475	up	0.007090601	0.020446217
G10090_1419	71816	Rnf180	-1.771594247	0.292884907	down	6.88587E-15	1.20038E-13
G10090_19702	71819	Kif23	-1.544844642	0.342732607	down	1.29897E-05	7.18419E-05
G10090_30384	71853	Pdia6	1.576851564	2.983181103	up	7.54979E-53	1.80966E-50
G10090_14105	71862	Gpr160	-3.065110103	0.119484046	down	3.32809E-05	0.000170768
G10090_23055	71869	Serpinb12	1.567506539	2.963920064	up	1.15226E-06	7.62289E-06
G10090_24197	71876	Cenpu	-1.189712853	0.438390107	down	0.017162584	0.043847234
G10090_21802	71878	Fam83d	-1.350588305	0.392132112	down	0.019108492	0.048077467
G10090_16833	71897	Lypd6b	4.068368708	16.77648661	up	6.09063E-05	0.000296427
G10090_7571	71910	Plpp5	1.35198433	2.552629812	up	2.4999E-08	2.13597E-07
G10090_30102	71918	Zcchc24	-2.122854252	0.229592234	down	2.15548E-16	4.43757E-15
G10090_25636	71934	Car13	1.030738297	2.043069521	up	5.16707E-13	7.71496E-12
G10090_21284	71973	Rbpms2	1.72774474	3.312096575	up	0.004575144	0.01396048
G10090_22688	71988	Esco2	-1.992401885	0.251320126	down	0.01149629	0.031080505
G10090_20151	72027	Slc39a4	1.350336798	2.549716419	up	1.06214E-09	1.09334E-08
G10090_16044	72043	Sulf2	-3.497949897	0.088514039	down	1.37092E-05	7.56031E-05
G10090_18604	72080	Sapcd2	-3.638359987	0.080305355	down	0.004601068	0.014023645
G10090_9515	72097	2010300C02Rik	-2.361005391	0.194655445	down	0.014476222	0.037941315
G10090_27940	72155	Cenpn	-1.285924574	0.410107896	down	0.001618448	0.005568939
G10090_30619	72157	Pgm2	1.711937944	3.276005875	up	2.1504E-45	3.65378E-43
G10090_3098	72230	Zfp558	3.066423065	8.3769384	up	6.99082E-29	4.15212E-27
G10090_2861	72244	1600014C10Rik	1.082259558	2.117349692	up	0.006857693	0.019838538
G10090_15542	72281	Sh2d4a	6.406401927	84.8240773	up	1.71711E-06	1.09704E-05
G10090_12591	72290	Lsm11	1.235363805	2.354407111	up	5.6629E-10	6.0137E-09
G10090_25315	72309	Tmem158	-1.311721895	0.402839793	down	0.000761904	0.00283219
G10090_18407	72324	Plxdc1	-2.977160136	0.126994671	down	4.86411E-26	2.2829E-24
G10090_13010	72333	Palld	1.88174218	3.685198114	up	5.75925E-42	7.88841E-40
G10090_13103	72431	Ceacam18	3.134252222	8.780190378	up	0.015025144	0.039131259
G10090_6365	72432	Spink5	3.629473794	12.37600512	up	0.001364298	0.004783161
G10090_23867	72480	Tspyl4	-1.802101758	0.286756529	down	3.88916E-05	0.00019722
G10090_4126	72500	Ier5l	-3.679884212	0.078026921	down	1.89959E-23	7.18259E-22
G10090_6556	72555	Shisa9	-3.923292871	0.065913013	down	0.000269127	0.001120151
G10090_7765	72565	Uaca	-1.291279348	0.408588542	down	0.000714533	0.002676855
G10090_8783	72599	Pdia5	1.415939286	2.668334043	up	0.000736621	0.002743523
G10090_16621	72691	Calhm2	-1.28819938	0.409461758	down	7.7849E-12	1.01948E-10
G10090_19461	72747	Ttc39c	1.497451764	2.823435675	up	6.13951E-14	9.91704E-13
G10090_3104	72754	Arhgef10l	-1.054743447	0.481382818	down	1.95164E-07	1.457E-06
G10090_11766	72925	March1	1.675426791	3.194138324	up	4.23016E-64	2.01455E-61

G10090_5984	72947	Phykpl	1.636126465	3.108301534	up	1.01988E-10	1.17308E-09
G10090_14495	72948	Tppp	-2.564885835	0.169002227	down	0.018766858	0.047368848
G10090_15027	72958	Zfp493	-1.823530683	0.282528697	down	0.000119039	0.000542835
G10090_7661	73086	Rps6ka5	-1.26033505	0.417447001	down	6.32935E-09	5.87544E-08
G10090_10561	73102	Slc22a23	2.062397	4.176796921	up	0.001828512	0.00619801
G10090_12975	73112	Abracl	1.198785889	2.295464132	up	4.50012E-07	3.18929E-06
G10090_30780	73149	Clec4a3	-1.242827446	0.422543728	down	1.653E-05	8.98672E-05
G10090_7308	73246	Rassf6	3.359001267	10.26030184	up	4.75857E-10	5.11403E-09
G10090_23773	73316	Calr3	2.434168153	5.404526224	up	2.78412E-11	3.41602E-10
G10090_22357	73338	Itpripl1	-1.158309312	0.448037281	down	8.16503E-23	2.94621E-21
G10090_7856	73379	Dcbld2	2.238944269	4.720515017	up	8.26753E-17	1.7756E-15
G10090_26254	73420	Ccsap	-2.286717001	0.204941349	down	1.47973E-08	1.29905E-07
G10090_10067	73451	Zfp763	-1.247495451	0.421178749	down	0.007652809	0.021828231
G10090_23393	73680	Zbtb8a	-1.943268987	0.260026581	down	0.000143308	0.000640996
G10090_25773	73707	Gucy2g	4.208381686	18.48626272	up	0.011241927	0.030540455
G10090_15020	73728	Psd	1.35206671	2.552775575	up	5.54872E-06	3.25384E-05
G10090_3009	73804	Kif2c	-2.053164749	0.240954934	down	0.003564052	0.011211688
G10090_21993	73822	Mfsd12	-1.178387503	0.441845072	down	1.96741E-12	2.79455E-11
G10090_22691	73910	Arhgap18	-2.137054993	0.227343397	down	1.48909E-14	2.53334E-13
G10090_7510	73914	Irak3	1.178986252	2.264176228	up	2.36049E-08	2.02459E-07
G10090_2496	73916	Ift57	1.370975642	2.586454194	up	1.22431E-23	4.74969E-22
G10090_1475	73988	4930438A08Rik	3.029462237	8.165052924	up	0.002089665	0.006989677
G10090_497	74048	Vsir	-1.480897042	0.358265979	down	1.97132E-10	2.19412E-09
G10090_2839	74107	Cep55	-1.59440263	0.331159321	down	0.003441715	0.01089683
G10090_25431	74116	Pi16	-2.63867894	0.160575207	down	0.000320933	0.001317396
G10090_23903	74121	Acoxl	1.314882422	2.487820544	up	0.013150159	0.034905
G10090_11652	74123	Foxp4	3.170133948	9.001303573	up	2.56957E-30	1.71599E-28
G10090_13322	74127	Krt80	-2.64144886	0.160267204	down	8.41482E-06	4.80035E-05
G10090_16930	74134	Cyp2s1	-1.734611915	0.300489833	down	0.008502916	0.023922581
G10090_13843	74155	Errfi1	1.195802294	2.290721855	up	2.29877E-08	1.9767E-07
G10090_33886	74185	Gbe1	1.968821195	3.914481417	up	3.62139E-30	2.37121E-28
G10090_18774	74190	Exoc3l4	1.535741349	2.89937381	up	1.28764E-14	2.19619E-13
G10090_17830	74191	P2ry13	1.345333085	2.54088853	up	2.06697E-06	1.30688E-05
G10090_14796	74198	Dtx2	1.714303106	3.281380981	up	4.27718E-18	1.04197E-16
G10090_22745	74241	Chpf	2.789063526	6.911809837	up	4.0627E-31	2.81101E-29
G10090_3605	74243	Slx4ip	1.91533414	3.772011671	up	9.95308E-26	4.54422E-24
G10090_20477	74318	Hopx	-1.448437935	0.366417945	down	0.003528872	0.011127096
G10090_21757	74356	4931428F04Rik	1.446218292	2.724928342	up	4.64314E-05	0.000231863
G10090_8186	74400	Zfp819	3.381714592	10.42311499	up	0.000110895	0.000509951
G10090_27593	74410	Ttll11	2.875987386	7.341054873	up	1.03074E-06	6.87316E-06
G10090_17649	74427	Eaf1	1.092071678	2.131799384	up	1.45046E-20	4.36536E-19
G10090_15930	74478	Snx29	-1.880874777	0.27151903	down	1.50698E-21	4.97008E-20
G10090_2123	74486	Osbpl10	-2.611549446	0.163623351	down	0.005356556	0.016006467
G10090_9450	74568	Mlkl	2.010528216	4.029297182	up	1.0341E-31	7.62675E-30
G10090_14400	74580	Pyroxd2	-2.144849259	0.226118469	down	2.7833E-08	2.36308E-07
G10090_17591	74637	Shpk	-1.03860172	0.486799057	down	0.000204371	0.000881796
G10090_5873	74694	Tbc1d30	-2.598694297	0.165087833	down	7.81212E-08	6.23402E-07
G10090_2713	74732	Stx11	1.511670272	2.851399673	up	2.08295E-35	1.9552E-33
G10090_10998	74747	Ddit4	3.569691772	11.87365152	up	1.14984E-20	3.50779E-19
G10090_9638	74760	Rab3il1	-2.142076773	0.226553428	down	8.1674E-09	7.46297E-08
G10090_20963	74840	Manf	1.787931922	3.453195281	up	1.325E-82	1.1857E-79
G10090_1366	75216	Cep128	-1.40745002	0.37697741	down	5.2872E-09	4.94221E-08
G10090_9427	75317	Parpbp	-1.874645817	0.272693872	down	0.005604397	0.016658064
G10090_7030	75472	Cfap126	-2.92043725	0.132087216	down	0.007075675	0.020416334
G10090_1100	75607	Wnk2	3.118272867	8.683477186	up	8.97622E-07	6.0638E-06
G10090_8546	75624	Metap1	1.577662725	2.984858882	up	1.76433E-26	8.58065E-25
G10090_3475	75646	Rai14	1.318072892	2.49332836	up	8.15247E-12	1.0645E-10
G10090_969	75659	Wdr54	1.289259162	2.444025202	up	0.000182709	0.000797302
G10090_5411	75691	Anks6	-1.708244597	0.306032208	down	0.000627948	0.002398676

G10090_12444	75744	Svip	-1.071417257	0.475851308	down	0.00107852	0.003889793
G10090_22481	75747	Sesn3	-1.491066817	0.355749389	down	5.49729E-05	0.000270889
G10090_20690	75767	Rab11fip1	2.038644809	4.108594098	up	2.2405E-12	3.15906E-11
G10090_25612	75811	Saxo1	6.746623041	107.383092	up	8.81601E-10	9.1169E-09
G10090_685	76044	Ncapg2	-2.013354792	0.247696469	down	8.98891E-06	5.11263E-05
G10090_2314	76051	Ganc	-1.125097316	0.458471095	down	2.71796E-09	2.66495E-08
G10090_28208	76072	Rnf183	1.229051644	2.344128478	up	0.017548663	0.044705961
G10090_7051	76117	Arhgap15	-2.196662198	0.218141748	down	3.09493E-16	6.29442E-15
G10090_10021	76131	Depdc1a	-1.756968979	0.295869117	down	0.016247241	0.04181912
G10090_10042	76184	Abca6	-4.780691081	0.036380493	down	7.97482E-08	6.34898E-07
G10090_23167	76187	Adhfe1	3.859383892	14.51410686	up	4.62571E-41	5.85763E-39
G10090_16558	76192	Abhd12	-1.448586506	0.366380213	down	1.26313E-23	4.88617E-22
G10090_29362	76238	Grhpr	1.224327826	2.336465648	up	2.25422E-06	1.41328E-05
G10090_26636	76263	Gstk1	-1.096720284	0.467578246	down	0.000908071	0.00332217
G10090_14789	76373	Zfp773	-1.628853505	0.323345065	down	0.00197584	0.006647042
G10090_9019	76408	Abcc3	-1.48314519	0.357708129	down	6.0253E-08	4.86628E-07
G10090_3683	76464	Knl1	-1.772355813	0.29273034	down	0.019322273	0.048551066
G10090_24600	76467	Msrb2	-1.407098428	0.377069293	down	0.005136717	0.01542509
G10090_9346	76478	Haus8	-1.437945159	0.369092632	down	0.001239984	0.004396277
G10090_19121	76497	Ppp1r11	1.111605135	2.160859296	up	8.07206E-17	1.74198E-15
G10090_25915	76498	Paqr4	-1.684299811	0.31115389	down	1.93108E-09	1.91865E-08
G10090_29988	76509	Plet1	2.53796016	5.807672738	up	0.004526175	0.013833071
G10090_20094	76524	Cln6	-1.034191992	0.488289279	down	1.78141E-09	1.77783E-08
G10090_14094	76612	Lrrc27	-1.274225389	0.413447087	down	3.02264E-08	2.55497E-07
G10090_17961	76650	Srxn1	1.231357277	2.347877725	up	2.25153E-06	1.41226E-05
G10090_14080	76737	Creld2	1.396366811	2.632378258	up	2.37513E-21	7.68227E-20
G10090_16125	76787	Ppfia3	2.680604171	6.411243353	up	7.97698E-43	1.17665E-40
G10090_15111	76788	Klhdc10	1.000939122	2.001302324	up	3.45607E-16	6.97606E-15
G10090_15348	76850	Ago4	-1.450186674	0.365974067	down	2.28929E-26	1.10537E-24
G10090_5456	76954	St5	1.065212835	2.092478551	up	0.003283794	0.010469922
G10090_8210	76974	Urah	2.839172753	7.156096056	up	0.012513965	0.033381349
G10090_21920	77011	Ticrr	-1.658716372	0.316720823	down	0.006013848	0.017694844
G10090_5709	77097	Tanc2	-1.299251244	0.406337032	down	8.00405E-10	8.34797E-09
G10090_301	77128	Crebrf	-1.399660504	0.379018322	down	3.10329E-42	4.43144E-40
G10090_24282	77446	Heg1	2.366432197	5.156643099	up	0.003910502	0.012187293
G10090_385	77577	Spns3	-1.726629435	0.302157062	down	0.000231755	0.000984759
G10090_23954	77596	Adgrf1	3.535218905	11.59329619	up	1.76651E-18	4.44875E-17
G10090_19204	77619	Prelid2	1.858937822	3.627404977	up	0.000522029	0.002036977
G10090_26146	77777	Ulbp1	-1.529887342	0.346304408	down	0.007448083	0.021321308
G10090_6177	77864	Ypel2	-1.40870609	0.376649341	down	8.49056E-20	2.42487E-18
G10090_10817	77963	Hook1	1.784733499	3.445548103	up	0.003262507	0.010411556
G10090_625	77974	Rdh12	2.492744998	5.628478558	up	1.40518E-05	7.7302E-05
G10090_12873	77976	Nuak1	-1.802772971	0.286623147	down	6.52953E-08	5.26402E-07
G10090_2378	78177	Ninl	-1.246463809	0.421480033	down	9.36808E-06	5.31928E-05
G10090_24467	78244	Dnajc21	1.090242263	2.129097861	up	7.24986E-09	6.66084E-08
G10090_3364	78248	Armcx1	2.423518646	5.364778647	up	0.018773938	0.04737781
G10090_7302	78255	Ralgps2	-2.224568105	0.2139628	down	1.83336E-05	9.87133E-05
G10090_9212	78286	Nav2	-2.795370461	0.144048799	down	2.87247E-16	5.85976E-15
G10090_8972	78317	Ccdc88b	1.54665583	2.921391745	up	5.99617E-46	1.04528E-43
G10090_11966	78558	Htra3	-1.55786278	0.339653875	down	0.018415428	0.046561483
G10090_18744	78688	Nol3	1.698604793	3.24586904	up	0.001724266	0.00588328
G10090_21108	78749	Filip1l	1.402891625	2.644310562	up	3.05313E-05	0.000158232
G10090_256	78795	Armc9	-1.250779354	0.42022114	down	4.70013E-12	6.34068E-11
G10090_30738	78903	Wrnip1	1.194223268	2.288216038	up	5.84609E-08	4.73865E-07
G10090_33376	78923	Chsy3	1.847645344	3.599122842	up	5.69211E-06	3.33065E-05
G10090_11722	79196	Osbpl5	-2.33759633	0.197839674	down	8.03428E-05	0.000381614
G10090_3827	79221	Hdac9	-1.432567067	0.370471106	down	5.54851E-05	0.000273012
G10090_25508	80782	Klrb1b	3.723966603	13.21373675	up	0.013250329	0.035113455
G10090_24352	80837	Rhoj	-1.378953199	0.384497681	down	5.49329E-07	3.84444E-06

G10090_5855	80859	Nfkbiz	1.970773155	3.919781278	up	1.60913E-47	2.91884E-45
G10090_2686	80879	Slc16a3	2.27583934	4.842793009	up	5.20838E-32	3.94983E-30
G10090_17820	80880	Kank3	-1.384949364	0.382902938	down	1.24685E-07	9.6076E-07
G10090_32049	80885	Hcar2	4.797598494	27.81128489	up	2.11904E-30	1.4222E-28
G10090_6445	80891	Fcrls	-2.965970896	0.127983444	down	1.81278E-06	1.15377E-05
G10090_15785	80910	Gpr84	3.208926679	9.246623709	up	2.3555E-61	9.29939E-59
G10090_14274	80914	Uck2	3.436613665	10.82739041	up	7.40595E-18	1.77518E-16
G10090_12418	80986	Ckap2	-1.825112543	0.282219085	down	0.001418688	0.004942395
G10090_8798	80987	Nckipsd	-1.110123228	0.46325446	down	7.35581E-16	1.43932E-14
G10090_26899	81535	Sgpp1	-1.478004242	0.358985072	down	2.93465E-15	5.37405E-14
G10090_32275	81897	Tlr9	-3.830677293	0.070283152	down	1.86388E-11	2.3426E-10
G10090_29013	81905	Cacng8	2.419364748	5.349354257	up	1.0961E-12	1.59403E-11
G10090_10115	83382	Siglece	2.97585672	7.867235224	up	1.5109E-36	1.53642E-34
G10090_4874	83383	Tfap4	-1.87804446	0.272052226	down	1.21772E-07	9.40477E-07
G10090_13250	83397	Akap12	3.534329149	11.58614844	up	3.30784E-08	2.79253E-07
G10090_27165	83921	Cemip2	1.042695053	2.060072418	up	7.85597E-08	6.26191E-07
G10090_6534	83996	Mmp1b	5.639448293	49.84746708	up	0.000595612	0.002292116
G10090_26125	85031	Pla1a	5.783169543	55.0690396	up	4.53847E-07	3.21477E-06
G10090_3308	93686	Rbfox2	2.422750597	5.361923356	up	1.05853E-14	1.8193E-13
G10090_8481	93691	Klf7	1.499546931	2.827539014	up	4.4409E-12	6.00909E-11
G10090_21250	93692	Glrx	1.49059224	2.810043064	up	1.06516E-34	9.66059E-33
G10090_23792	93704	Pcdhgb7	1.255549251	2.387580293	up	0.000917543	0.003354079
G10090_24186	93708	Pcdhgc5	-2.230437338	0.213094116	down	0.012340998	0.032965814
G10090_17918	93716	Pcdhga8	4.872372261	29.29073034	up	0.000551876	0.00214099
G10090_30182	93723	Pcdhga11	2.103360932	4.297092794	up	0.001118862	0.004013492
G10090_25090	94043	Tm2d1	1.214182674	2.32009306	up	2.04127E-17	4.7487E-16
G10090_3094	94045	P2rx5	1.065212446	2.092477988	up	1.28659E-07	9.89677E-07
G10090_10195	94092	Trim16	-1.593160483	0.331444569	down	2.95009E-18	7.2261E-17
G10090_11175	94176	Dock2	-1.030674677	0.489481189	down	6.58537E-21	2.05571E-19
G10090_14380	94180	Acsbg1	2.003202739	4.008889743	up	3.86603E-05	0.000196196
G10090_14194	94185	Tnfrsf21	-1.331966728	0.397226361	down	4.63803E-10	4.98849E-09
G10090_16	94190	Ophn1	-1.73625349	0.300148115	down	8.2093E-28	4.51612E-26
G10090_10210	94249	Slc24a3	-2.410472452	0.188094236	down	0.001235607	0.004383074
G10090_14431	94352	Loxl2	4.115462155	17.33315237	up	0.001090502	0.003926452
G10090_7342	97130	C77080	-1.797463336	0.287679967	down	4.39579E-21	1.39162E-19
G10090_9310	98170	Tmem132a	1.349852048	2.548859849	up	1.01396E-09	1.04454E-08
G10090_28727	98238	Lrrc59	1.268856857	2.409705529	up	3.34238E-31	2.34894E-29
G10090_8822	98496	Pid1	1.17784457	2.262385173	up	1.12136E-24	4.68908E-23
G10090_12733	98682	Mfsd6	-1.553223906	0.340747764	down	0.000188506	0.000820465
G10090_14163	98878	Ehd4	-1.023828366	0.491809543	down	2.64675E-06	1.64174E-05
G10090_29065	99382	Abtb2	3.552032993	11.72920229	up	4.80483E-21	1.51043E-19
G10090_15688	99526	Usp53	1.057608223	2.081477875	up	0.001622399	0.005579674
G10090_30313	99543	Olfml3	-3.907397514	0.066643246	down	5.3971E-10	5.74506E-09
G10090_17759	99899	Ifi44	1.657953684	3.155686051	up	0.004110536	0.012722095
G10090_31173	99929	Tiparp	1.432758238	2.699623539	up	1.9376E-12	2.75513E-11
G10090_25277	100340	Smpdl3b	2.906990337	7.500518534	up	1.00454E-06	6.71513E-06
G10090_6931	100532	Rell1	-1.765737755	0.294076263	down	1.16145E-28	6.71988E-27
G10090_27475	100561	Slc15a4	-1.324440595	0.399303993	down	9.6382E-19	2.49756E-17
G10090_14337	100604	Lrrc8c	1.314981446	2.487991309	up	1.46452E-15	2.76098E-14
G10090_33703	100637	N4bp2l1	-1.300999673	0.405844882	down	2.9696E-15	5.43065E-14
G10090_12014	100702	Gbp6	3.320505425	9.990143663	up	9.23089E-28	5.0574E-26
G10090_8906	100855	Tbc1d14	-1.08120168	0.472634982	down	3.71786E-14	6.13835E-13
G10090_16170	100900	Hscb	-1.047130869	0.483929613	down	8.97908E-08	7.09812E-07
G10090_4854	101488	Slco2b1	-6.537458749	0.010765367	down	9.1799E-05	0.000430544
G10090_12564	101631	Pwwp2b	-1.417841226	0.374271934	down	9.44951E-23	3.39146E-21
G10090_5690	102103	Mtus1	-2.365134573	0.194099113	down	6.8777E-09	6.33627E-08
G10090_24131	102141	Snx25	1.396555854	2.632723213	up	1.96052E-10	2.18391E-09
G10090_4623	102580	Alg9	1.20771404	2.309713709	up	4.45497E-11	5.34398E-10
G10090_13331	102644	Oaf	2.493215815	5.630315688	up	3.68617E-19	9.89589E-18

G10090_28970	102871	D330045A20Rik	1.961406676	3.894415124	up	2.17498E-38	2.51679E-36
G10090_4098	103220	Ttc41	1.271262858	2.413727578	up	2.65459E-07	1.94183E-06
G10090_31335	103784	Wdr92	1.990001955	3.972375366	up	5.9493E-12	7.96185E-11
G10090_5468	104010	Cdh22	6.243646868	75.7748333	up	3.16152E-06	1.93247E-05
G10090_9230	104027	Synpo	3.047601755	8.268363158	up	1.79337E-07	1.34784E-06
G10090_2041	104086	Cyp27a1	-3.244645965	0.105502862	down	7.05583E-57	2.36776E-54
G10090_13338	104111	Adcy3	-3.068154218	0.119232198	down	1.8119E-08	1.57623E-07
G10090_13832	104215	Rhoq	1.170636658	2.25111016	up	1.45383E-22	5.14902E-21
G10090_22244	104252	Cdc42ep2	2.833596211	7.128488531	up	7.20111E-68	3.86642E-65
G10090_14715	104271	Tex15	-1.341947181	0.394487863	down	0.014535866	0.038053348
G10090_4177	105349	Akr1c18	3.469982363	11.08074028	up	0.00512854	0.015410879
G10090_1629	105670	Rcbtb2	-1.669924861	0.314269711	down	6.51447E-16	1.28405E-14
G10090_13092	105841	Dennd3	1.291165045	2.447256033	up	5.85979E-05	0.000286543
G10090_2551	106014	Fam19a5	-4.697773298	0.03853269	down	0.014537553	0.038053348
G10090_10932	106068	Slc45a4	-1.809800176	0.285230433	down	2.85665E-20	8.44598E-19
G10090_6562	106393	Srl	-1.626681684	0.323832192	down	0.001378465	0.00482606
G10090_21819	106582	Nrm	-1.561105992	0.338891183	down	8.41261E-09	7.67136E-08
G10090_15904	106931	Kctd1	2.450268638	5.465178578	up	1.67401E-05	9.08255E-05
G10090_7681	106952	Arap3	-1.136943066	0.45472207	down	6.02294E-07	4.19108E-06
G10090_11137	107221	Ffar4	-3.319409275	0.100174744	down	0.000134879	0.000606392
G10090_20807	107227	Macrod1	-1.472535293	0.360348491	down	1.59434E-09	1.60426E-08
G10090_17671	107272	Psat1	1.216352177	2.323584607	up	1.07279E-10	1.22972E-09
G10090_25288	107449	Unc5b	2.016447033	4.04586178	up	2.11776E-07	1.57315E-06
G10090_1834	107527	Il1rl2	1.75100057	3.36591926	up	4.14665E-15	7.45119E-14
G10090_18497	107568	Wwp1	-2.2262002	0.213720885	down	4.16558E-34	3.65454E-32
G10090_10038	107746	Rapgef1	1.030418851	2.042617189	up	3.62474E-20	1.06233E-18
G10090_12616	107766	Haao	-2.485690651	0.178538776	down	0.000820097	0.003022558
G10090_26010	107767	Scamp1	1.109827124	2.158197844	up	1.43874E-05	7.89543E-05
G10090_11965	107769	Tm6sf1	-1.268052035	0.415220036	down	1.58437E-33	1.32093E-31
G10090_25498	107771	Bmyc	-2.547172771	0.171089986	down	0.000961709	0.003502176
G10090_21664	107869	Cth	2.771024796	6.825926104	up	0.004306227	0.013245298
G10090_26516	107885	Mthfs	-1.457215581	0.364195353	down	1.86483E-06	1.18577E-05
G10090_2946	107986	Ddb2	-1.117620831	0.460853199	down	2.7303E-06	1.68733E-05
G10090_11758	108000	Cenpf	-2.368148694	0.193694018	down	0.000217677	0.000932613
G10090_3474	108012	Ap1s2	-1.350722399	0.392095666	down	2.2191E-21	7.20791E-20
G10090_2280	108078	Olr1	4.84094705	28.65960956	up	1.73063E-37	1.91985E-35
G10090_9489	108099	Prkag2	-1.462011964	0.36298656	down	2.15059E-25	9.58208E-24
G10090_10225	108100	Baiap2	-1.249547352	0.420580145	down	1.07372E-14	1.84068E-13
G10090_17667	108105	B3gnt5	2.675618424	6.389125258	up	4.38352E-12	5.94945E-11
G10090_23380	108116	Slco3a1	1.0838236	2.119646379	up	1.04989E-07	8.19342E-07
G10090_3706	108153	Adamts7	5.262311819	38.38077184	up	7.77797E-21	2.42236E-19
G10090_10040	108159	Ubxn8	1.037268666	2.052338453	up	8.62458E-11	1.00406E-09
G10090_29152	108673	Ccdc86	1.134713367	2.195749336	up	1.13877E-28	6.61718E-27
G10090_8306	108723	Card11	-3.03336963	0.122141923	down	0.005028347	0.015136915
G10090_18403	108995	Tbc1d10c	-1.878719308	0.271924999	down	0.000430462	0.001711776
G10090_10101	109050	Inka2	-2.405292433	0.188770806	down	7.00887E-05	0.00033612
G10090_4024	109135	Plekha5	1.020185376	2.02817955	up	0.000582624	0.002247936
G10090_3302	109212	Pimreg	-2.135523402	0.227584877	down	0.004243404	0.013085048
G10090_9602	109232	Sccpdh	1.151917085	2.222089746	up	2.22759E-06	1.3992E-05
G10090_10911	109242	Kif24	-1.172803123	0.443558679	down	0.000163539	0.00072305
G10090_5147	109332	Cdcp1	2.541909694	5.823593655	up	0.00338707	0.010753225
G10090_2103	109552	Sri	1.010011718	2.013927457	up	1.00775E-07	7.88286E-07
G10090_17804	109575	Tbx10	1.447201114	2.726785307	up	0.011033303	0.030040573
G10090_11289	109624	Cald1	2.288522444	4.885554939	up	0.00015861	0.000704275
G10090_26799	109652	Acy1	2.779184127	6.864640301	up	3.2217E-14	5.33887E-13
G10090_1265	109676	Ank2	-2.026231853	0.245495442	down	0.000254053	0.001066006
G10090_29802	109700	Itga1	1.671746094	3.185999618	up	4.86262E-05	0.000241923
G10090_4833	110074	Dut	-1.153995089	0.449379093	down	0.003774197	0.011800849
G10090_4523	110094	Phka2	-1.361688118	0.389126701	down	6.53912E-16	1.28702E-14

G10090_23975	110168	Gpr18	2.104640118	4.300904561	up	8.92809E-53	2.10249E-50
G10090_27815	110173	Manba	-1.270526957	0.414508343	down	3.4846E-12	4.78749E-11
G10090_5821	110197	Dgkg	-3.458967568	0.090938337	down	3.32291E-10	3.62629E-09
G10090_30878	110208	Pgd	1.085525963	2.122149013	up	1.12809E-08	1.00949E-07
G10090_26886	110558	H2-Q9	1.60606777	3.044209758	up	0.000182505	0.000796672
G10090_6696	110751	Adam33	-2.170528878	0.222129225	down	0.000113919	0.000522068
G10090_5542	110789	Adgrv1	3.444136469	10.88399632	up	2.24582E-06	1.40934E-05
G10090_23983	112407	Egln3	3.200135599	9.190450607	up	1.31865E-18	3.38438E-17
G10090_6642	114642	Brdt	-1.4403953	0.36846633	down	0.000551654	0.00214075
G10090_26027	114671	4930444G20Rik	4.215252829	18.57451748	up	3.61215E-06	2.18602E-05
G10090_12192	114713	Rasa2	1.187579042	2.277702057	up	2.54065E-09	2.49475E-08
G10090_2045	114715	Spred1	1.490251531	2.809379518	up	1.05678E-41	1.40447E-39
G10090_11280	114716	Spred2	1.469762821	2.769763549	up	2.70558E-18	6.67593E-17
G10090_2978	116838	Rims2	1.410821365	2.658884972	up	5.39897E-08	4.40014E-07
G10090_18518	117198	Ivns1abp	-1.556566752	0.339959137	down	8.80783E-65	4.3788E-62
G10090_8241	117591	Slc2a9	-1.047767565	0.483716091	down	5.79292E-07	4.04149E-06
G10090_126	117600	Srgap1	2.206636313	4.615977887	up	0.004738011	0.014388761
G10090_10229	117606	Boc	2.998055398	7.989224099	up	2.18661E-07	1.62339E-06
G10090_33567	121021	Cspg4	-2.117205651	0.230492921	down	0.000130327	0.000589015
G10090_4565	140481	Man2a2	-2.465862	0.181009585	down	6.48368E-19	1.70648E-17
G10090_731	140486	Igf2bp1	5.457035701	43.92698881	up	2.40967E-07	1.77622E-06
G10090_23398	140497	Cd300c2	-1.029217708	0.489975763	down	2.80353E-15	5.15505E-14
G10090_1226	140571	Plxnb3	-2.123794009	0.229442729	down	5.04684E-05	0.000250531
G10090_9634	140742	Sesn1	-2.642994936	0.160095545	down	1.42299E-28	8.19777E-27
G10090_16259	140792	Colec12	-1.648916295	0.318879599	down	5.69699E-55	1.58109E-52
G10090_11815	140795	P2ry14	1.105384878	2.151562693	up	2.54849E-23	9.52879E-22
G10090_25867	140887	Lnx2	-1.324490959	0.399290054	down	1.98618E-18	4.95547E-17
G10090_12757	142688	Asb13	-1.194472245	0.436946259	down	0.000390486	0.001570251
G10090_10942	170625	Snx18	1.052815376	2.07457437	up	5.06563E-19	1.34379E-17
G10090_20979	170676	Peg10	3.711683525	13.10171281	up	6.27048E-31	4.29432E-29
G10090_6194	170706	Tmem37	-3.166753608	0.111355629	down	1.97273E-17	4.59721E-16
G10090_28365	170733	Klra17	-5.458483976	0.022742206	down	1.34185E-09	1.36245E-08
G10090_8105	170741	Pilrb1	2.186007063	4.550443189	up	1.2615E-21	4.181E-20
G10090_1119	170744	Tlr8	-2.054048773	0.240807332	down	3.57074E-24	1.44804E-22
G10090_30760	170757	Adgrl4	-4.33888656	0.049415705	down	0.015914212	0.041127545
G10090_7393	170768	Pfkfb3	1.760896998	3.389087765	up	2.65698E-77	1.98137E-74
G10090_3621	170770	Bbc3	-1.471355826	0.360643212	down	4.26422E-08	3.55299E-07
G10090_10416	170835	Inpp5j	-1.339068233	0.395275863	down	0.004313435	0.013261393
G10090_10059	171171	Ntng2	5.156350895	35.66286976	up	9.87214E-33	7.84105E-31
G10090_23161	171210	Acot2	-1.28067109	0.411604001	down	5.46559E-08	4.44634E-07
G10090_5182	171211	Edaradd	-1.414577887	0.375119486	down	4.22367E-05	0.000212418
G10090_3012	171463	Il17rd	2.496499031	5.643143478	up	0.002916922	0.009436936
G10090_13093	171543	Bmf	-2.488933873	0.178137867	down	2.89207E-18	7.10995E-17
G10090_16689	171580	Mical1	-1.366562495	0.387814192	down	1.52348E-12	2.18714E-11
G10090_14231	192136	Sugct	-2.783527918	0.145236108	down	0.000731618	0.002727918
G10090_10046	192187	Stab1	-1.360331653	0.389492741	down	3.06455E-14	5.09103E-13
G10090_9073	192188	Stab2	-3.649805764	0.079670765	down	0.000296426	0.001224284
G10090_30048	192289	Tmlhe	-1.148117543	0.4512136	down	1.67148E-09	1.6756E-08
G10090_6130	192654	Pla2g15	-1.718386535	0.30388839	down	7.45829E-20	2.14374E-18
G10090_9644	192657	Ell2	1.657761149	3.155264938	up	1.76693E-53	4.47501E-51
G10090_25301	192663	Abcg4	4.363970912	20.59141279	up	1.31799E-13	2.06675E-12
G10090_26619	192678	Rassf3	-1.659047682	0.316648098	down	2.29805E-29	1.40852E-27
G10090_3010	194126	Mtmr11	-1.227460049	0.427068664	down	1.18389E-07	9.16987E-07
G10090_8022	194590	Reps2	-2.790881515	0.144497705	down	3.13618E-14	5.20358E-13
G10090_24234	194655	Klf11	-1.670642474	0.314113429	down	1.11362E-12	1.61777E-11
G10090_14621	195727	Nhs	4.857829848	28.99696191	up	1.33166E-06	8.70249E-06
G10090_22465	207474	Kctd12b	-3.17164523	0.110978704	down	3.55138E-46	6.27239E-44
G10090_6746	207592	Tbc1d16	-1.501459098	0.353195998	down	4.39288E-06	2.62536E-05
G10090_4973	207792	BC034090	3.880122495	14.72425255	up	0.014629886	0.038257737

G10090_8148	207818	Smagp	-2.96952875	0.127668211	down	1.48637E-09	1.50237E-08
G10090_10472	208098	Panx3	4.551410661	23.44828757	up	0.007259794	0.020848997
G10090_10280	208431	Shroom4	2.623386059	6.161946086	up	5.82475E-05	0.000285037
G10090_328	208618	Etl4	-2.276770378	0.206359194	down	0.000557346	0.002159717
G10090_32770	208634	Tspan10	-2.666646043	0.157492383	down	0.002071906	0.006938921
G10090_11948	208922	Cpeb3	-1.459807194	0.363541711	down	3.96272E-10	4.30352E-09
G10090_6354	209131	Snx30	-1.081139067	0.472655495	down	4.40966E-20	1.28397E-18
G10090_886	209176	Ido2	2.52153566	5.74192967	up	1.76016E-22	6.18499E-21
G10090_15780	209212	Osgin2	1.606022823	3.044114918	up	2.51133E-18	6.23097E-17
G10090_15335	209590	Il23r	4.389350516	20.95685782	up	4.93427E-21	1.54749E-19
G10090_4758	209630	Frmd4a	-1.157570442	0.4482668	down	1.9474E-14	3.2798E-13
G10090_18204	209737	Kif15	-1.902878508	0.26740929	down	0.000382971	0.001541877
G10090_21083	209760	Tmc7	3.6010049	12.13418157	up	0.012533484	0.033420133
G10090_1581	209773	Dennd2a	-1.057014537	0.480625621	down	8.59268E-06	4.89879E-05
G10090_29190	209966	Pgbd5	-1.572459258	0.33623475	down	0.000422474	0.001681753
G10090_20900	210104	Zfp658	1.215843558	2.322765576	up	4.84965E-11	5.78125E-10
G10090_7551	210105	Zfp719	1.248434988	2.375835563	up	4.14727E-12	5.63449E-11
G10090_10393	210126	Lpp	1.930056579	3.810701436	up	2.92728E-24	1.19795E-22
G10090_31058	210622	Pamr1	4.817041164	28.18862426	up	0.000179828	0.000787032
G10090_9222	210710	Gab3	-2.596612769	0.165326195	down	3.98825E-16	7.96641E-15
G10090_32558	210741	Kcnk12	-3.557398062	0.084940825	down	0.004598175	0.014018012
G10090_8371	210789	Tbc1d4	-2.012002449	0.247928762	down	1.41837E-11	1.80462E-10
G10090_8011	210808	Lacc1	1.45924881	2.749651561	up	3.20667E-05	0.000164979
G10090_21653	210992	Lpcat1	-1.162890765	0.446616741	down	2.75052E-07	2.00544E-06
G10090_353	211135	D130040H23Rik	1.090651955	2.129702562	up	0.01358868	0.035884488
G10090_9558	211323	Nrg1	4.762661105	27.14587542	up	2.37433E-19	6.49096E-18
G10090_2403	211401	Mtss1	-1.634303361	0.322125916	down	8.69497E-19	2.26187E-17
G10090_29233	211548	Nomo1	1.659832611	3.159798609	up	5.17485E-41	6.49178E-39
G10090_1456	211586	Tfdp2	-1.750915724	0.297113132	down	2.84388E-25	1.23538E-23
G10090_18759	211612	Ptchd1	-1.367311686	0.387612853	down	0.004240476	0.013082028
G10090_12644	211651	Fancd2	-1.446401778	0.366935456	down	0.003031212	0.009747952
G10090_31823	211673	Arfgef1	1.064984533	2.09214745	up	3.40027E-25	1.47232E-23
G10090_7199	211949	Spsb4	1.147482761	2.215270329	up	0.001092366	0.003931053
G10090_12866	212073	Syne3	-1.627955774	0.323546332	down	8.7246E-32	6.50613E-30
G10090_9376	212167	Gsap	1.409567803	2.656575662	up	8.35316E-32	6.26394E-30
G10090_33145	212398	Frat2	-3.20735583	0.108265401	down	9.66023E-16	1.87113E-14
G10090_2577	212503	Paox	-1.185241178	0.439751018	down	8.37295E-13	1.22697E-11
G10090_17984	212569	Zfp273	-1.205621148	0.433582626	down	0.001558774	0.00538154
G10090_10056	212712	Satb2	1.268512448	2.409130338	up	0.005169957	0.015511026
G10090_2898	212937	Tifab	-1.281720243	0.411304784	down	0.000196418	0.00085214
G10090_3970	213053	Slc39a14	2.02217919	4.061968888	up	2.30362E-17	5.30387E-16
G10090_17846	213068	Tmem71	-1.387008201	0.382356896	down	0.003515161	0.0110935
G10090_30980	213208	Il20rb	1.437384206	2.708293712	up	0.000780638	0.002894613
G10090_10552	213233	Tapbpl	1.04548026	2.064053348	up	5.98704E-22	2.03453E-20
G10090_9311	213311	Fbxl21	1.088564103	2.126622709	up	0.019768724	0.049497405
G10090_15239	213402	Armc2	-2.866053739	0.137161383	down	0.001659736	0.005689132
G10090_395	213556	Plekhh2	-2.581768638	0.167036045	down	0.000117896	0.000539003
G10090_16461	213573	Cracr2b	-2.517405966	0.174656717	down	1.56942E-27	8.26132E-26
G10090_8952	213783	Plekhg1	1.241693431	2.364759441	up	7.15384E-07	4.91433E-06
G10090_17767	213956	Fam83f	-2.04867401	0.241706134	down	1.38587E-06	9.02597E-06
G10090_9049	213980	Fbxw10	-1.808867195	0.285414949	down	1.235E-07	9.52179E-07
G10090_16554	214105	Sox30	3.313107951	9.939049928	up	0.002605584	0.008530429
G10090_13929	214239	Ccdc9b	-2.348888694	0.196297174	down	0.008640082	0.024252576
G10090_21722	214403	Gm4788	-2.103487708	0.232695028	down	0.0119082	0.031994688
G10090_26167	214459	Fnbp1l	1.717063824	3.287666191	up	8.86824E-12	1.15124E-10
G10090_14538	214639	4930486L24Rik	1.241150426	2.363869555	up	0.003425785	0.010853037
G10090_7010	214642	Cped1	-2.418590712	0.187038775	down	0.00021125	0.000907685
G10090_7169	214763	Cgas	1.090267336	2.129134864	up	7.64869E-09	7.0033E-08
G10090_10321	214855	Arid5a	1.338966442	2.529700242	up	5.00962E-19	1.33157E-17

G10090_17592	214944	Mob3b	1.243218696	2.367260866	up	9.36185E-11	1.08331E-09
G10090_24032	215085	Slc35f1	2.575921494	5.96251708	up	0.003107079	0.009956152
G10090_261	215113	Slc43a2	-1.012562733	0.49566499	down	2.52726E-07	1.85678E-06
G10090_3577	215114	Hip1	-1.179229914	0.441587147	down	1.12646E-18	2.90779E-17
G10090_16064	215257	Il1f9	4.443949311	21.7651689	up	2.56014E-07	1.87785E-06
G10090_33716	215387	Ncaph	-1.471033371	0.360723828	down	2.92471E-08	2.48E-07
G10090_5738	215418	Csrnp1	1.182075672	2.269029982	up	1.07301E-14	1.84068E-13
G10090_10740	215512	Fam117a	-2.980393162	0.126710399	down	7.07725E-10	7.44498E-09
G10090_5646	215627	Zbtb8b	2.309608285	4.957484578	up	0.007450812	0.021324575
G10090_13515	215653	Rassf2	-1.504303593	0.352500304	down	5.45289E-11	6.47735E-10
G10090_1774	215789	Phactr2	-1.092210606	0.469042122	down	3.41607E-09	3.28702E-08
G10090_32721	216134	Pdxk	-1.552732987	0.340863733	down	1.68808E-16	3.52946E-15
G10090_28549	216148	Shc2	-2.000491204	0.249914895	down	0.000143538	0.000641809
G10090_28668	216188	Aldh1l2	4.080210072	16.91475144	up	3.99795E-07	2.84995E-06
G10090_21766	216198	Tcp11l2	-1.909010037	0.266275198	down	6.99398E-56	2.04087E-53
G10090_8946	216233	Socs2	4.690621627	25.82366077	up	5.55157E-15	9.80509E-14
G10090_9811	216505	Pik3ip1	-2.182986203	0.22021945	down	0.001604115	0.005522452
G10090_25094	216543	Cep68	-1.181493703	0.440894779	down	1.11256E-11	1.42665E-10
G10090_5877	216799	Nlrp3	2.815044929	7.037411766	up	6.67669E-19	1.75041E-17
G10090_14093	216805	Flcn	-1.019779552	0.493191708	down	8.45789E-13	1.23806E-11
G10090_6488	216991	Adap2	-1.160434968	0.447377632	down	1.72894E-09	1.72721E-08
G10090_14577	217143	Gpr179	2.063717356	4.180621277	up	0.001496753	0.005190111
G10090_33132	217166	Nr1d1	-1.113651646	0.462122857	down	1.60949E-06	1.03518E-05
G10090_25670	217214	Nags	5.241265362	37.82492619	up	0.00374048	0.011700876
G10090_10507	217262	Abca9	-1.681270093	0.311808013	down	4.9751E-05	0.000247153
G10090_4891	217303	Cd300a	-2.755233846	0.148112587	down	2.44877E-57	8.64996E-55
G10090_12333	217304	Cd300lb	-1.576772556	0.335230994	down	1.37791E-18	3.5297E-17
G10090_28247	217305	Cd300ld	-1.177137416	0.442228094	down	1.73007E-05	9.37154E-05
G10090_21951	217306	Cd300e	3.91631006	15.09825654	up	7.2372E-18	1.73783E-16
G10090_13153	217310	Hid1	3.290874842	9.787055236	up	2.86381E-05	0.000149112
G10090_7398	217333	Trim47	-2.075149586	0.237310923	down	3.33197E-24	1.3553E-22
G10090_9364	217364	Engase	-2.878515973	0.135981663	down	3.05885E-36	2.997E-34
G10090_32719	217653	Mis18bp1	-1.006184709	0.497861131	down	1.99984E-06	1.26862E-05
G10090_6313	217692	Sipa1l1	1.846947318	3.597381884	up	3.14679E-12	4.37714E-11
G10090_1538	217732	Cipc	-1.08081454	0.472761828	down	5.16727E-08	4.2267E-07
G10090_4484	217835	Rin3	-1.65008228	0.318621985	down	1.97E-09	1.95298E-08
G10090_26570	217944	Rapgef5	-1.447492946	0.366658033	down	1.78883E-29	1.11164E-27
G10090_16471	218121	Mboat1	-1.006619121	0.497711242	down	6.25487E-05	0.00030354
G10090_30958	218203	Mylip	-1.657953762	0.316888287	down	9.55423E-11	1.10272E-09
G10090_15406	218215	Rnf144b	-2.592818386	0.165761586	down	1.65888E-12	2.36633E-11
G10090_2690	218581	Depdc1b	-2.677313207	0.156332192	down	0.001229356	0.004363206
G10090_21981	218630	Ccno	3.166497075	8.978640866	up	8.14519E-06	4.6604E-05
G10090_21829	218952	Fermt2	4.888804112	29.62624981	up	0.002136677	0.007130935
G10090_13705	219114	Ska3	-1.402838233	0.378184402	down	0.01192873	0.032043093
G10090_23953	219132	Phf11d	1.2410092	2.363638167	up	0.000902645	0.003305022
G10090_5045	219140	Spata13	1.680506317	3.205404258	up	9.95909E-28	5.43418E-26
G10090_8679	219144	Arl11	-1.489334224	0.35617688	down	4.56892E-30	2.97711E-28
G10090_15948	219148	Fam167a	2.379627673	5.204024208	up	0.000714038	0.002676273
G10090_2215	223267	Ggact	-1.05163794	0.482420146	down	0.007529864	0.021518707
G10090_2828	223433	Otulinl	-2.162614577	0.223351124	down	5.75348E-22	1.96013E-20
G10090_6141	223645	Mroh6	-1.984595936	0.252683622	down	2.50158E-05	0.000131578
G10090_14536	223666	Arhgap39	-1.034090586	0.488323602	down	9.0232E-08	7.1288E-07
G10090_3227	223753	Cerk	-1.244499579	0.422054269	down	2.34518E-13	3.58943E-12
G10090_20940	223775	Pim3	2.017059986	4.047581095	up	1.12404E-22	4.01276E-21
G10090_14978	223864	Rapgef3	-4.583120683	0.041719894	down	2.92839E-08	2.48155E-07
G10090_33475	223881	Rnd1	3.244671033	9.478580589	up	2.06596E-40	2.52104E-38
G10090_20059	223920	Soat2	3.12358361	8.715501072	up	2.81206E-07	2.04698E-06
G10090_20831	223970	Rmi2	4.082904452	16.94637098	up	6.50795E-07	4.50058E-06
G10090_6749	224014	Fgd4	-1.361323583	0.389225036	down	2.59299E-11	3.19905E-10

G10090_277	224022	Slc7a4	-2.080013572	0.236512187	down	3.4759E-07	2.50037E-06
G10090_5517	224079	Atp13a4	2.057831059	4.1635988	up	0.017532071	0.044689135
G10090_518	224250	Cldnd1	1.157528251	2.23074909	up	4.08692E-07	2.91182E-06
G10090_6588	224454	Zdhhc14	-2.092286777	0.234508679	down	1.36079E-15	2.57265E-14
G10090_18190	224530	Acat3	3.74660512	13.42271976	up	2.48268E-07	1.82503E-06
G10090_19023	224656	Zfp523	1.437828242	2.709127404	up	9.54959E-10	9.86791E-09
G10090_2523	224697	Adamts10	-1.174286895	0.443102725	down	2.41796E-09	2.37951E-08
G10090_22181	224796	Clic5	1.366415552	2.578291802	up	0.010738319	0.029392425
G10090_14116	224829	Trerf1	-1.525253661	0.347418465	down	0.000196258	0.000851721
G10090_8108	224840	Treml4	3.510944471	11.39986212	up	3.80335E-12	5.18298E-11
G10090_23492	225030	Kcng3	3.287402816	9.763529781	up	4.05475E-05	0.000204844
G10090_348	225372	Apbb3	2.175368061	4.517009853	up	1.418E-23	5.42274E-22
G10090_18185	225579	Slc27a6	-4.972621487	0.031848705	down	3.65138E-12	4.99618E-11
G10090_29194	225638	Alpk2	3.682168172	12.83639489	up	1.04549E-08	9.40593E-08
G10090_15509	225872	Npas4	-1.911891196	0.26574396	down	0.003450779	0.010914187
G10090_3895	226043	Cbwd1	1.247373855	2.374088728	up	2.19716E-05	0.000116756
G10090_3886	226075	Glis3	2.737893128	6.67095416	up	4.4778E-21	1.41425E-19
G10090_19524	226255	Atrnl1	2.100624858	4.288951074	up	9.06596E-12	1.17577E-10
G10090_3887	226409	Zranb3	-1.501892881	0.353089817	down	0.000380043	0.001531468
G10090_949	226421	Rab7b	-1.573192281	0.336063955	down	5.93976E-07	4.13535E-06
G10090_10384	226841	Vash2	-1.753329713	0.296616403	down	5.97336E-06	3.48005E-05
G10090_798	226922	Kcnq5	4.1904285	18.25764142	up	0.004612391	0.014048587
G10090_18543	227094	Nemp2	-1.733446095	0.300732753	down	1.9855E-06	1.26011E-05
G10090_10405	227326	Gpr55	3.662067551	12.65878952	up	4.91186E-09	4.6268E-08
G10090_6084	227671	Gbgt1	1.156159889	2.228634281	up	3.59098E-16	7.19428E-15
G10090_19928	227731	Slc25a25	1.065077797	2.092282703	up	5.34481E-08	4.3613E-07
G10090_4491	227933	Ccdc148	-4.661711576	0.039507994	down	0.005547592	0.016511159
G10090_6837	228026	Pdk1	1.36462478	2.575093435	up	2.11303E-10	2.34213E-09
G10090_10309	228094	Cerkl	-1.805685308	0.286045131	down	5.55816E-06	3.25795E-05
G10090_1667	228355	Madd	-1.602884881	0.329217999	down	5.45256E-16	1.08109E-14
G10090_22130	228413	Prrg4	-2.353777179	0.195633158	down	7.84152E-11	9.1687E-10
G10090_4338	228421	Kif18a	-1.652525936	0.318082755	down	0.00034533	0.001408925
G10090_1573	228608	Smox	1.504798859	2.837851031	up	5.02278E-11	5.97172E-10
G10090_18148	228775	Trib3	1.20188499	2.300400395	up	2.30645E-05	0.00012208
G10090_7218	228785	Mylk2	5.888174875	59.22666208	up	1.45977E-09	1.4766E-08
G10090_1413	228846	D630003M21Rik	5.073969536	33.68348568	up	0.005290639	0.015836788
G10090_4837	229003	Helz2	1.524825555	2.877519201	up	1.1499E-32	9.02635E-31
G10090_20758	229595	Adamtsl4	1.198627927	2.295212813	up	0.002197418	0.007305229
G10090_22842	229715	Amigo1	-1.317114328	0.401336889	down	0.001123339	0.004022225
G10090_1330	229841	Cenpe	-1.809328529	0.285323696	down	0.001656076	0.005678034
G10090_23580	229898	Gbp5	4.602766408	24.29801253	up	2.18752E-68	1.22346E-65
G10090_14738	229900	Gbp7	3.146284995	8.853727645	up	6.77402E-52	1.49062E-49
G10090_15293	229905	Kyat3	1.042588888	2.059920827	up	0.007269645	0.020863896
G10090_5859	229933	Clca2	4.559920928	23.58701467	up	0.003447537	0.010909075
G10090_32151	230073	Ddx58	1.001107557	2.00153599	up	6.05345E-06	3.52213E-05
G10090_25323	230500	Efcab7	-1.008005399	0.497233223	down	0.016093489	0.041518912
G10090_4593	230594	Tut4	-1.063909933	0.47833394	down	3.6151E-12	4.95664E-11
G10090_13126	230603	Ttc39a	-1.48387485	0.35752726	down	0.006115255	0.017961722
G10090_4970	230718	Nt5c1a	3.902248092	14.95180851	up	0.013532087	0.035769412
G10090_32841	230738	Zc3h12a	1.405748206	2.649551567	up	6.52815E-12	8.67598E-11
G10090_17366	230815	Man1c1	-1.001606031	0.499443702	down	5.61306E-22	1.91715E-20
G10090_24512	230991	Fndc10	-2.776717375	0.145923346	down	0.000557899	0.002161235
G10090_4480	231004	Samd11	-1.697925792	0.308228935	down	1.71874E-06	1.09756E-05
G10090_3923	231093	Agbl5	-1.001549848	0.499463152	down	2.51175E-06	1.56379E-05
G10090_3270	231125	Zfyve28	-3.451882853	0.091386011	down	2.26627E-25	1.00066E-23
G10090_8505	231134	Dok7	2.91850339	7.560613943	up	6.33715E-15	1.11194E-13
G10090_3506	231148	Ablim2	1.192177881	2.284974209	up	0.000236939	0.001002658
G10090_33011	231201	AF366264	3.258632424	9.57075292	up	0.001678746	0.005746035
G10090_11943	231225	Tapt1	1.211680149	2.316072074	up	9.04472E-19	2.3483E-17

G10090_7744	231532	Arhgap24	-1.331086094	0.397468905	down	1.7561E-07	1.32205E-06
G10090_2362	231583	Slc26a1	-2.552439807	0.170466505	down	0.000777333	0.00288475
G10090_22201	231605	Galnt9	-1.905073095	0.267002824	down	3.99185E-07	2.84711E-06
G10090_26343	231630	Ficd	1.059668418	2.084452387	up	7.88342E-13	1.1603E-11
G10090_9719	231655	Oasl1	2.098528813	4.282724319	up	2.04343E-05	0.000109192
G10090_18158	231668	Vsig10	-2.210767663	0.216019333	down	8.69423E-05	0.000410491
G10090_16156	231713	Naa25	1.342768002	2.536374895	up	7.60821E-41	9.45602E-39
G10090_22289	231805	Pilra	2.168735241	4.496290472	up	6.4169E-13	9.51758E-12
G10090_11739	231834	Snx8	-1.088223399	0.470340217	down	2.25692E-36	2.22755E-34
G10090_23847	231842	Amz1	-2.450164916	0.182989793	down	4.54376E-12	6.1359E-11
G10090_22616	231871	Daglb	-3.619524971	0.081360651	down	4.30764E-27	2.15752E-25
G10090_26711	231931	Gimap6	2.719592309	6.586866495	up	0.000236765	0.001002239
G10090_16392	231986	Jazf1	1.735131246	3.329097783	up	4.64192E-14	7.58011E-13
G10090_8307	231991	Creb5	1.231352719	2.347870309	up	7.72152E-19	2.01646E-17
G10090_8621	232035	Ccser1	3.304440749	9.879518628	up	1.93633E-14	3.26525E-13
G10090_3070	232078	Thnsl2	-2.666123586	0.157549427	down	0.005131693	0.015413453
G10090_14649	232201	Arhgap25	-1.289933712	0.40896982	down	6.09217E-09	5.67096E-08
G10090_6337	232288	Frmd4b	-3.034804541	0.122020501	down	2.7978E-16	5.71612E-15
G10090_6700	232334	Vgll4	-1.129659464	0.457023589	down	1.60659E-21	5.27267E-20
G10090_13536	232431	Gprc5a	2.389046557	5.238110729	up	0.000205654	0.000886479
G10090_6265	232533	Stk38l	1.48167358	2.79272512	up	1.33719E-15	2.53519E-14
G10090_7838	232539	Klhl42	-1.120910578	0.459803522	down	4.76691E-05	0.000237514
G10090_13659	232941	Ppm1n	2.149210405	4.435849458	up	0.002689404	0.008774883
G10090_25427	232984	B3gnt8	-1.190011321	0.438299421	down	2.35477E-26	1.12886E-24
G10090_7748	233016	Blvrb	1.400577931	2.6400732	up	1.30265E-15	2.47597E-14
G10090_15183	233020	Hipk4	2.185320095	4.548276922	up	0.016213472	0.041756223
G10090_5029	233046	Rasgrp4	-1.16420191	0.446211033	down	1.53119E-06	9.90513E-06
G10090_7900	233064	Wdr62	-1.109806076	0.46335631	down	2.2253E-17	5.14118E-16
G10090_11420	233071	Arhgap33	-1.727436447	0.301988089	down	0.002042179	0.006847907
G10090_3117	233079	Ffar2	3.457961345	10.98879543	up	0.006507977	0.018956005
G10090_23386	233115	Dpy19l3	1.520955347	2.869810244	up	0.00309952	0.009935747
G10090_11029	233186	Siglecf	-4.010700251	0.062038162	down	4.78232E-15	8.51368E-14
G10090_7662	233424	Tmc3	3.259878468	9.579022667	up	2.75191E-54	7.38779E-52
G10090_14996	233571	P2ry6	-1.259031981	0.417824217	down	1.13876E-11	1.45855E-10
G10090_10758	233651	Dchs1	-2.539119851	0.172047657	down	9.23282E-07	6.22462E-06
G10090_1133	233765	Plekha7	1.639416245	3.115397486	up	0.005487451	0.016361184
G10090_32056	233781	Xylt1	-1.974263909	0.254499741	down	6.74368E-24	2.66237E-22
G10090_8871	233826	Palb2	-1.255050665	0.418978853	down	3.74609E-06	2.25995E-05
G10090_13533	234258	Neil3	-1.869579789	0.27365312	down	0.007493078	0.021436399
G10090_14243	234797	6430548M08Rik	-1.054556938	0.481445054	down	3.97169E-09	3.78367E-08
G10090_32656	235041	Kank2	-1.147207767	0.451498229	down	1.71929E-10	1.92638E-09
G10090_1809	235493	Fam214a	-2.539529292	0.171998836	down	6.09834E-21	1.90811E-19
G10090_14914	235504	Slc17a5	-1.217173322	0.430124638	down	1.26941E-42	1.83219E-40
G10090_24173	235527	Plscr4	-2.431060278	0.185429119	down	7.60373E-07	5.20473E-06
G10090_2122	235633	Als2cl	-1.46700843	0.361731608	down	2.60032E-08	2.21895E-07
G10090_5303	236266	Alms1	-1.154310505	0.449280856	down	0.000425648	0.001693885
G10090_20606	236285	Lancl3	1.628508732	3.091932302	up	0.000102392	0.000474262
G10090_33464	236539	Phgdh	2.947383496	7.71348858	up	1.29142E-33	1.09024E-31
G10090_22277	236576	Spry3	-2.6234018	0.162284622	down	1.9733E-09	1.9548E-08
G10090_16495	236727	Slc9a7	-2.354234451	0.195571161	down	1.33359E-13	2.08877E-12
G10090_3913	236899	Pcyt1b	-2.630065892	0.161536726	down	6.7857E-15	1.186E-13
G10090_11457	236920	Stard8	-1.057313662	0.48052598	down	8.70642E-13	1.27305E-11
G10090_24409	236930	Ercc6l	-1.398297362	0.37937661	down	0.000753516	0.002802563
G10090_2635	237711	Eml6	2.529864855	5.775175764	up	2.67372E-08	2.27869E-07
G10090_6899	237860	Ssh2	-1.261090179	0.41722856	down	2.01857E-14	3.3954E-13
G10090_15229	237868	Sarm1	-1.652716073	0.318040837	down	0.001770006	0.006019454
G10090_3359	238161	Akap6	2.553938175	5.872350886	up	3.00383E-08	2.54067E-07
G10090_10116	238455	Macc1	5.911561174	60.19455867	up	0.000439768	0.001744388
G10090_1730	238871	Pde4d	1.581095117	2.991968772	up	5.94282E-08	4.80545E-07

G10090_7367	239027	Arhgap22	-2.004326069	0.249251472	down	7.17864E-15	1.24817E-13
G10090_12082	239273	Abcc4	1.179681715	2.265267955	up	1.45835E-09	1.47627E-08
G10090_8086	239827	Pigz	-1.281799371	0.411282226	down	0.00047761	0.001882255
G10090_25189	239849	Cd200r4	-1.594432408	0.331152486	down	0.00574867	0.017026567
G10090_18005	240041	Zfp945	-1.13904725	0.454059338	down	5.61915E-07	3.92843E-06
G10090_22501	240047	Mmp25	1.277566919	2.424297782	up	0.01154009	0.031180078
G10090_31850	240055	Neurl1b	-1.997242628	0.250478273	down	6.24477E-06	3.62715E-05
G10090_21139	240063	Zfp811	1.788415975	3.45435409	up	0.001165056	0.004156978
G10090_16576	240168	Rasgrp3	-3.26720808	0.103865751	down	7.16298E-26	3.29276E-24
G10090_25206	240216	E230025N22Rik	3.618055703	12.27844284	up	6.55646E-05	0.000316687
G10090_643	240354	Malt1	2.711877636	6.551737867	up	2.41456E-43	3.72536E-41
G10090_18676	240505	Cdc42bpg	1.02647878	2.037046312	up	7.20454E-15	1.25105E-13
G10090_13898	240641	Kif20b	-1.488988569	0.356262226	down	0.002817686	0.009158014
G10090_32306	240672	Dusp5	2.228505208	4.686481566	up	2.4853E-09	2.44397E-08
G10090_8550	240816	Rgsl1	7.204634584	147.5064862	up	1.03351E-08	9.30434E-08
G10090_16547	241062	Pgap1	-1.679578171	0.3121739	down	8.69617E-26	3.98392E-24
G10090_21882	241226	Itga8	-1.998325622	0.250290316	down	8.22915E-05	0.00039018
G10090_2499	241274	Pnpla7	-1.375382067	0.385450614	down	1.66347E-21	5.44604E-20
G10090_17336	241303	Fam78a	-1.579781034	0.334532659	down	2.97206E-19	8.04314E-18
G10090_588	241308	Ralgps1	1.147502199	2.215300176	up	7.7092E-10	8.05923E-09
G10090_13958	241576	Ldlrad3	-1.793087155	0.288553922	down	1.01908E-10	1.17308E-09
G10090_10404	241638	Lzts3	1.321488929	2.499239099	up	1.2197E-06	8.02663E-06
G10090_20440	241727	Snph	-4.092449764	0.058620547	down	0.000802344	0.002962008
G10090_26728	242100	Pglyrp3	5.130051474	35.01864786	up	0.000897521	0.003288052
G10090_20266	242126	Slc22a15	-1.04250387	0.48548416	down	2.38458E-05	0.000125868
G10090_4135	242202	Pde5a	3.236563966	9.425466117	up	9.71353E-20	2.75893E-18
G10090_17644	242297	Fam110b	-2.625596251	0.162037962	down	3.61679E-14	5.97884E-13
G10090_16205	242341	Atp6v0d2	-1.485804338	0.357049416	down	4.06444E-05	0.000205179
G10090_29914	242681	Rab42	-1.778623252	0.291461402	down	0.001729977	0.005901267
G10090_3961	242864	Napepld	1.419011954	2.674023147	up	2.02088E-06	1.27894E-05
G10090_6708	242960	Fbxl5	2.119810554	4.346368674	up	3.62761E-33	2.95111E-31
G10090_5605	243197	Mfsd7a	1.073849967	2.105043384	up	2.98867E-11	3.65031E-10
G10090_9105	243219	2900026A02Rik	-1.644178948	0.319928418	down	3.49295E-13	5.29784E-12
G10090_2256	243725	Ppp1r9a	1.188622096	2.279349407	up	1.54877E-05	8.46807E-05
G10090_15184	243816	Gp6	5.388426915	41.88689142	up	0.000119288	0.00054352
G10090_9673	243864	Mill2	-1.203578598	0.434196921	down	0.003996861	0.012416075
G10090_17920	243905	Zfp568	1.022570947	2.031536023	up	5.93615E-06	3.46288E-05
G10090_7339	243910	Nfkbid	1.333566234	2.520248922	up	3.43251E-09	3.29811E-08
G10090_15187	244144	Usp35	-1.028614079	0.490180814	down	2.21561E-15	4.09081E-14
G10090_11425	244202	Nlrp10	-1.854980556	0.276436389	down	2.90385E-18	7.12585E-17
G10090_32524	244238	Mrgpre	-1.521120829	0.348415128	down	0.000104094	0.000481481
G10090_25410	244556	Zfp791	-2.089161778	0.235017195	down	0.00041529	0.001656101
G10090_19840	244864	Layn	1.216038709	2.323079794	up	1.64084E-36	1.64366E-34
G10090_22965	244871	Zc3h12c	1.374516531	2.592810073	up	9.81975E-22	3.27073E-20
G10090_25170	244882	Tnfaip8l3	1.493017857	2.814771591	up	0.009929706	0.027397007
G10090_4455	245007	Zbtb38	-1.321404414	0.400145222	down	4.47396E-11	5.36077E-10
G10090_26046	245038	Dclk3	-2.901240437	0.133856543	down	8.03662E-07	5.47314E-06
G10090_14180	245126	Tarm1	4.671263537	25.47947306	up	1.74456E-18	4.41002E-17
G10090_23397	245240	9930111J21Rik2	-1.95110153	0.258618694	down	1.42339E-05	7.81758E-05
G10090_12436	245527	Eda2r	-2.475303909	0.179828812	down	0.007236966	0.020796788
G10090_3656	245671	Klf8	-1.059789505	0.479702045	down	0.000285875	0.001185386
G10090_6275	246049	Slc36a2	-3.406538722	0.094303902	down	4.05663E-27	2.0394E-25
G10090_10268	246102	Rttn	-1.657841467	0.316912953	down	1.68305E-09	1.68468E-08
G10090_139	246277	Csad	-1.088207155	0.470345512	down	3.42017E-13	5.19332E-12
G10090_24121	246278	Cd207	-3.035896679	0.121928164	down	1.78155E-11	2.24332E-10
G10090_13559	246738	Dnajc28	-1.271693746	0.414173242	down	4.3914E-12	5.95412E-11
G10090_15981	252967	Ropn1l	-1.362604645	0.388879572	down	3.72277E-06	2.24891E-05
G10090_11957	252972	Tpcn1	-2.262183375	0.208456263	down	1.80583E-30	1.21808E-28
G10090_5430	257632	Nod2	2.011032831	4.030706767	up	1.01216E-21	3.36292E-20

G10090_32744	258504	Olfr107	3.66578094	12.69141427	up	0.007739359	0.022046206
G10090_10760	258571	Olfr1033	1.373073863	2.590218608	up	0.003999467	0.012421298
G10090_3611	260315	Nav3	2.958306889	7.772113069	up	0.002967162	0.00956719
G10090_4263	260409	Cdc42ep3	-1.590832338	0.331979868	down	8.99004E-30	5.77384E-28
G10090_7412	263406	Plekhg3	-1.100009447	0.466513441	down	8.02597E-18	1.91695E-16
G10090_10406	268445	Ankrd13b	-1.046951004	0.48398995	down	3.14477E-06	1.92311E-05
G10090_581	268451	Rab11fip4	-1.437681691	0.369160042	down	0.014215481	0.037370623
G10090_28120	268481	Krt222	5.454436817	43.84792957	up	0.000788483	0.002920478
G10090_14350	268512	Slc26a11	-2.761728228	0.147447347	down	7.65713E-42	1.02782E-39
G10090_15849	268515	Bahcc1	-1.38811355	0.382064058	down	0.002141792	0.007146227
G10090_3462	268857	Nlrc3	-2.235444724	0.212355779	down	4.00372E-21	1.27049E-19
G10090_1399	268859	Rbfox1	2.411603974	5.320655421	up	3.94147E-26	1.8629E-24
G10090_930	268902	Robo2	4.34870678	20.37469814	up	1.3126E-25	5.93231E-24
G10090_2249	268977	Ltbp1	3.285429689	9.750185649	up	4.12077E-05	0.00020771
G10090_18761	269060	Dagla	-1.524621515	0.347570727	down	7.73207E-33	6.21482E-31
G10090_22356	269152	Kif26b	-4.311664937	0.050356962	down	5.63992E-18	1.35915E-16
G10090_450	269180	Inpp4a	-1.279611545	0.411906402	down	9.4141E-33	7.52175E-31
G10090_12584	269181	Mgat4a	1.322370421	2.50076661	up	5.55738E-14	9.02017E-13
G10090_8410	269233	Fam171a1	-3.256075894	0.104670305	down	1.9373E-07	1.44791E-06
G10090_7328	269295	Rtn4rl2	3.923150009	15.17000871	up	1.64894E-11	2.08612E-10
G10090_14898	269582	Clspn	-1.280249782	0.411724218	down	0.017542445	0.044698603
G10090_14160	269610	Chd5	-1.311503413	0.402900803	down	0.000238682	0.00100908
G10090_18091	269642	Nat8l	-5.590500071	0.020753521	down	1.00867E-06	6.73935E-06
G10090_19674	269701	Wdr66	1.059384574	2.08404232	up	0.00079641	0.002947399
G10090_20772	269717	Orai2	1.722863986	3.300910417	up	9.16668E-29	5.37312E-27
G10090_17059	269823	Pon3	1.420585376	2.676941062	up	1.2956E-17	3.06177E-16
G10090_3767	270035	Letm2	-1.445616716	0.367135184	down	0.003462145	0.010944977
G10090_5590	270118	Maml2	-2.047229803	0.241948214	down	2.10393E-17	4.886E-16
G10090_1406	270152	Jaml	-2.398067749	0.189718497	down	8.59835E-06	4.89879E-05
G10090_32285	270160	Rab39	-2.08867675	0.23509622	down	1.26648E-06	8.32105E-06
G10090_7803	270210	Zfp651	-1.326030997	0.39886405	down	0.00041412	0.001651925
G10090_11719	270685	Mthfd1l	1.279925792	2.428264863	up	1.35111E-16	2.83375E-15
G10090_641	270757	Bpifc	3.374315334	10.36979403	up	1.86618E-06	1.18607E-05
G10090_21640	270906	Prr11	-1.874068456	0.272803024	down	0.002076812	0.006953618
G10090_9421	271221	Rubcnl	-1.467763013	0.361542458	down	0.004007849	0.012438697
G10090_7106	271697	Cdk15	-3.613366824	0.081708681	down	0.006905781	0.019956147
G10090_12549	271849	Shc4	-1.476002353	0.359483547	down	0.019142794	0.048145722
G10090_31743	272396	Tarsl2	-1.402604683	0.378245629	down	6.88282E-07	4.74028E-06
G10090_24096	277154	Nynrin	-2.532525588	0.172835851	down	1.9838E-12	2.8132E-11
G10090_13173	279029	Stkld1	-1.712872477	0.305052091	down	0.000150664	0.000671211
G10090_638	279572	Tlr13	-1.048380049	0.483510777	down	1.39575E-09	1.41504E-08
G10090_8485	282619	Sbsn	-1.756707697	0.295922706	down	0.003547815	0.011176324
G10090_4800	286942	Kif19a	-1.840710263	0.279184303	down	0.004010357	0.012440726
G10090_23593	317758	Gimap9	4.204421726	18.43559055	up	0.008844572	0.024759268
G10090_5544	319430	C5ar2	-2.305125993	0.202342882	down	0.009547475	0.026472992
G10090_20130	319480	Itga11	2.216107689	4.646381742	up	0.006266813	0.018342657
G10090_5067	319504	Nrcam	-3.576059897	0.083849158	down	4.34849E-09	4.12216E-08
G10090_3957	319520	Dusp4	1.475707807	2.781200603	up	3.48998E-18	8.51746E-17
G10090_13464	319555	Nwd1	-4.801339198	0.035863517	down	0.0010316	0.003735409
G10090_11630	319581	Xkr5	-2.640167348	0.160409629	down	0.002185039	0.007275858
G10090_12925	319660	Agmo	-3.849951753	0.069350411	down	3.48687E-16	7.01712E-15
G10090_3544	319701	Fbxo48	-2.407128556	0.188530709	down	0.007176629	0.020641073
G10090_11005	319710	Frmd6	1.28223375	2.4321526	up	5.37515E-05	0.000265552
G10090_28414	319734	Cacna2d4	-4.218456733	0.053717772	down	0.007976532	0.022655308
G10090_13969	319909	Ism1	6.424108439	85.8715566	up	1.51004E-06	9.77302E-06
G10090_20340	320024	Nceh1	-1.823961122	0.282444415	down	3.8193E-26	1.81154E-24
G10090_7318	320100	Relt	1.601557786	3.034708167	up	4.68975E-18	1.13835E-16
G10090_8272	320159	Togaram2	3.389278494	10.47790582	up	7.42391E-05	0.000354252
G10090_1400	320365	Fry	-1.496381229	0.354441336	down	5.72304E-25	2.43874E-23

G10090_946	320405	Cadps2	2.154982276	4.45363176	up	0.005752716	0.017034791
G10090_23423	320472	Ppm1e	-2.293127553	0.204032722	down	0.011387872	0.030849528
G10090_1787	320484	Rasal3	-1.073926489	0.475024395	down	2.61312E-19	7.11478E-18
G10090_16288	320528	Vps13c	-1.04306756	0.485294508	down	7.69381E-11	9.00383E-10
G10090_4469	320560	Dennd5b	-1.568047315	0.337264572	down	0.017639229	0.044894079
G10090_26974	320782	Tmem154	-1.090264636	0.469675213	down	0.001051496	0.003803176
G10090_2550	320827	C530008M17Rik	1.041257254	2.058020359	up	0.004204662	0.012986466
G10090_4079	320878	Mical2	-1.177721344	0.442049139	down	1.07387E-07	8.3611E-07
G10090_16409	320910	Itgb8	5.795454354	55.53996467	up	3.52468E-31	2.45139E-29
G10090_8528	320916	Wscd2	4.692088873	25.84993724	up	0.000132403	0.000597596
G10090_18418	320924	Ccbe1	1.729166289	3.315361734	up	0.00307627	0.009878653
G10090_10353	321006	Dcaf1	1.533945417	2.895766784	up	5.44298E-13	8.1089E-12
G10090_26459	321019	Gpr183	-3.379236952	0.096105516	down	1.33723E-17	3.15459E-16
G10090_15377	326623	Tnfsf15	2.644120414	6.251144768	up	4.482E-15	8.03229E-14
G10090_21029	327957	Scimp	5.132077021	35.06784864	up	1.49053E-27	7.90807E-26
G10090_5425	327959	Xaf1	1.486042846	2.801195832	up	2.95914E-09	2.88457E-08
G10090_5764	328232	Gfod1	1.302648614	2.466813443	up	4.9531E-09	4.65911E-08
G10090_1653	328234	Rnf182	6.103623802	68.76601327	up	6.06998E-06	3.53021E-05
G10090_16049	329244	Il19	6.708167988	104.558605	up	3.39954E-05	0.000174168
G10090_1016	329251	Ppp1r12b	1.358395223	2.563998153	up	2.77357E-09	2.71551E-08
G10090_14199	329278	Tnn	4.550059966	23.42634483	up	0.005001101	0.015066248
G10090_8928	329421	Myo3b	2.582919449	5.991509199	up	0.003546161	0.011176324
G10090_19832	329436	Gm14461	-2.699234422	0.153974738	down	0.000497249	0.001951629
G10090_19124	329547	Bpi	4.573787877	23.81482227	up	0.019949123	0.049856095
G10090_16418	329679	Fnip2	-1.187021646	0.439208644	down	4.77424E-24	1.92446E-22
G10090_509	329727	Dennd2c	-1.087403188	0.470607694	down	0.00393951	0.012263462
G10090_5205	329739	Fam102b	1.370877859	2.586278896	up	8.99484E-35	8.21345E-33
G10090_9417	329877	Dennd4c	-1.548170139	0.341943498	down	7.05075E-36	6.76016E-34
G10090_20902	330096	Shisa3	4.967815509	31.29402903	up	0.006541999	0.019031914
G10090_2060	330122	Cxcl3	8.425282881	343.765959	up	1.69976E-32	1.3265E-30
G10090_26394	330267	Thsd7a	5.578252269	47.77726184	up	0.000530294	0.002066824
G10090_18421	330301	Zfp786	4.803962861	27.93424381	up	0.008954957	0.025026522
G10090_7959	330460	Tmem150b	-1.149657329	0.450732277	down	0.000112834	0.000517771
G10090_3432	330627	Trim66	-2.085582146	0.235601047	down	0.008230413	0.023287696
G10090_12919	330812	Rnf150	-4.382601153	0.047940835	down	6.3212E-23	2.29944E-21
G10090_107	330908	Opcml	-5.090380868	0.029352336	down	1.44882E-14	2.46797E-13
G10090_29385	331004	Slc9a9	-5.329693112	0.024865804	down	1.13446E-67	5.85687E-65
G10090_13073	331493	Gm5127	2.2757912	4.842631417	up	0.003861439	0.012051173
G10090_2749	332175	Zdhhc23	-3.240093792	0.105836283	down	0.0083661	0.023611892
G10090_13328	333050	Ksr2	-2.922206831	0.1319253	down	1.21865E-06	8.02644E-06
G10090_9747	338372	Map3k9	-1.274058619	0.413494883	down	4.79229E-06	2.8413E-05
G10090_16177	353047	Plekhm1	-1.227880807	0.426944129	down	1.58308E-18	4.01694E-17
G10090_16183	353187	Nr1d2	-1.414284292	0.375195833	down	9.13639E-24	3.56505E-22
G10090_4124	353346	Gpr141	1.137718526	2.200327887	up	7.62113E-10	7.99206E-09
G10090_32897	378431	Txlnb	2.470117181	5.540887904	up	5.5245E-07	3.86427E-06
G10090_10204	378460	Pram1	-1.835663916	0.280162561	down	0.000438068	0.00173867
G10090_25386	380686	Cnrip1	-1.04402821	0.484971472	down	0.005523062	0.016452744
G10090_13106	380711	Rap1gap2	1.975884328	3.933692889	up	1.25942E-09	1.28264E-08
G10090_9965	380773	Slirp	1.040281218	2.056628504	up	7.09411E-08	5.69182E-07
G10090_1340	380839	Serpinb1c	-1.371842587	0.386397432	down	0.001989348	0.006689133
G10090_12917	380912	Zfp395	-3.327508322	0.099613955	down	2.11918E-20	6.30727E-19
G10090_64	381062	Ermard	-1.472668836	0.360315136	down	4.11672E-12	5.59865E-11
G10090_9657	381126	Garem1	-1.090138885	0.469716154	down	0.004130277	0.012774357
G10090_10724	381199	Tmem151a	-1.252548034	0.419706283	down	0.002960118	0.009549067
G10090_6493	381269	Mreg	1.787886351	3.453086206	up	0.000255836	0.001072146
G10090_3643	381293	Kif14	-1.880410262	0.271606467	down	0.001182788	0.004216881
G10090_13487	381560	Xkr8	1.71298274	3.278379208	up	7.33475E-23	2.65375E-21
G10090_5626	381680	Nxpe5	-3.331267759	0.099354715	down	2.23542E-20	6.6385E-19
G10090_25085	381810	Lpar5	-2.212016726	0.215832387	down	7.73754E-16	1.50961E-14

G10090_5540	381836	Sbk2	-1.49079601	0.355816172	down	4.59715E-06	2.73648E-05
G10090_3216	381853	Gipr	4.13241006	17.5379724	up	1.76104E-05	9.51245E-05
G10090_29851	382019	Zfp882	-1.467355075	0.361644703	down	9.7465E-05	0.00045406
G10090_32227	382551	Cd300ld3	-2.721725377	0.151592956	down	2.17801E-19	5.99088E-18
G10090_21927	382913	Neil2	-1.873484858	0.272913401	down	5.23468E-05	0.000259185
G10090_18820	383435	Ms4a14	-1.245516643	0.421756836	down	0.00082723	0.003046341
G10090_26255	383548	Serpinb3b	7.51322704	182.6866042	up	2.36006E-07	1.74253E-06
G10090_6626	384009	Glipr2	2.345692015	5.083041518	up	5.47103E-08	4.44807E-07
G10090_27608	399603	Fam84b	-1.122652553	0.459248669	down	1.09854E-09	1.12562E-08
G10090_22391	404710	Iqgap3	-1.99804987	0.25033816	down	0.00466245	0.01418175
G10090_19172	407821	Znrf3	-1.19336032	0.437283155	down	2.31108E-08	1.98447E-07
G10090_33681	432779	Lrrc14b	-2.780034941	0.145588172	down	1.7991E-05	9.69854E-05
G10090_25688	433016	Gm5483	7.382050697	166.8086967	up	0.000346014	0.001411147
G10090_18338	433182	Eno1b	1.263843698	2.401346678	up	1.11089E-26	5.48216E-25
G10090_6224	433256	Acsl5	1.160355116	2.235124379	up	1.68619E-21	5.507E-20
G10090_20453	433470	AA467197	3.120153745	8.694805438	up	9.41919E-09	8.54282E-08
G10090_14791	433904	Ociad2	-1.79958163	0.287257879	down	6.35443E-06	3.68447E-05
G10090_2864	433940	Fam222a	-3.289112061	0.102300701	down	0.011453108	0.030988725
G10090_30305	435653	Fcrlb	1.39984375	2.638730021	up	2.69546E-05	0.000141002
G10090_11276	436440	Gpr31b	1.637481066	3.111221405	up	0.014425884	0.037842221
G10090_12932	494448	Cbx6	-1.641917926	0.32043021	down	2.03795E-12	2.8856E-11
G10090_31038	494504	Apcdd1	-3.189424318	0.109619448	down	1.05196E-10	1.20687E-09
G10090_20852	544922	Zkscan4	-1.073278946	0.475237654	down	0.000674923	0.002552687
G10090_11631	544971	Bdp1	1.576088884	2.981604464	up	5.39614E-44	8.52146E-42
G10090_904	545156	Kalrn	2.516360176	5.721368135	up	0.000995478	0.003618278
G10090_11428	545554	Ankrd34a	-2.391599722	0.190570971	down	0.00054791	0.002128064
G10090_15557	545812	Pilrb2	1.778401013	3.430457548	up	9.87583E-13	1.44091E-11
G10090_9189	546611	Klhl33	-1.40378139	0.377937246	down	0.011491402	0.031073548
G10090_24356	547347	Gm6034	2.625824697	6.172370647	up	1.17703E-05	6.57755E-05
G10090_29735	619441	Tnfsfm13	-1.348745206	0.392633396	down	1.50215E-09	1.51719E-08
G10090_9582	620235	Siglec15	-2.300169683	0.203039217	down	3.47737E-05	0.000177816
G10090_20049	620913	Gm12185	-1.872315817	0.273134637	down	0.014065741	0.037049537
G10090_30036	621823	Psme2b	-2.814755862	0.142126171	down	3.14436E-06	1.92311E-05
G10090_3003	622675	Zfp827	1.871018478	3.657907207	up	0.000169583	0.00074633
G10090_29586	622976	Gm6377	3.005679353	8.031555091	up	1.48162E-27	7.89198E-26
G10090_7257	625098	Slc38a6	1.061541778	2.087160835	up	4.53936E-14	7.43071E-13
G10090_17808	629378	Dact3	-1.889855122	0.269834155	down	0.0082759	0.023399328
G10090_33386	630146	Cd101	-1.438401022	0.368976024	down	0.008416595	0.023734444
G10090_10333	631323	Gm12250	1.310400167	2.480103222	up	0.001859189	0.006287705
G10090_18065	634731	Susd1	-1.010225204	0.496468743	down	0.00725004	0.0208299
G10090_28000	652925	Tmem243	1.762620713	3.393139428	up	5.69539E-24	2.25909E-22
G10090_25918	654812	Angptl7	-3.685731826	0.077711298	down	8.24021E-12	1.07491E-10
G10090_5421	654824	Ankrd37	2.046108984	4.12990611	up	1.89046E-12	2.69094E-11
G10090_11572	665433	Hist1h2ao	-1.535861664	0.344873298	down	0.010921147	0.029819885
G10090_4373	665563	Mthfd2l	2.232499466	4.699474579	up	7.93263E-13	1.16449E-11
G10090_23098	665700	Hmcn2	2.362927503	5.144131428	up	1.92148E-18	4.82094E-17
G10090_19338	667214	9930111J21Rik1	-2.415393883	0.187453688	down	1.11279E-11	1.42665E-10
G10090_760	668101	Sirpb1b	-1.080226227	0.472954654	down	3.46764E-06	2.10711E-05
G10090_11920	668212	Efr3b	-1.62274477	0.324717092	down	5.41684E-07	3.79688E-06
G10090_11065	668225	Fignl2	1.244019894	2.368575885	up	1.0596E-08	9.50105E-08
G10090_11860	668923	Zfp442	-1.014989366	0.494831975	down	2.13166E-06	1.34524E-05
G10090_7908	671535	Parp10	1.315216669	2.488396994	up	1.26409E-16	2.66372E-15
G10090_29184	751864	Gm9733	-2.988965722	0.125959713	down	2.4565E-06	1.53295E-05
G10090_5908	751865	Sap25	-1.147561092	0.451387668	down	3.74249E-12	5.11042E-11
G10090_22451	100034251	Wfdc17	2.352956747	5.108701873	up	1.61323E-19	4.47406E-18
G10090_15776	100037283	Rnaset2a	1.613495272	3.059922835	up	1.20616E-31	8.7991E-30
G10090_29284	100038882	Isg15	2.797955104	6.954540055	up	3.091E-17	7.00854E-16
G10090_26507	100038947	LOC100038947	-1.054105896	0.481595596	down	0.000449352	0.001777676
G10090_26876	100041194	Ahnak2	-1.409199418	0.376520568	down	6.6522E-10	7.03091E-09

G10090_5556	100041420	Gm3325	-1.158354025	0.448023395	down	0.004642065	0.014126149
G10090_11908	100041621	Gm3435	-1.177850199	0.442009659	down	0.001242578	0.004401204
G10090_225	100041734	4930522L14Rik	-1.255795077	0.418762721	down	4.47558E-06	2.66923E-05
G10090_32448	100042295	Gm3776	5.166725685	35.92025496	up	1.02165E-19	2.89316E-18
G10090_95	100042480	Nhsl2	-1.768722403	0.293468507	down	2.13897E-12	3.02544E-11
G10090_21392	100043123	Cd300ld4	-3.942703165	0.065032146	down	1.25638E-10	1.42435E-09
G10090_29151	100043125	Cd300ld5	-2.728976344	0.150832963	down	1.11284E-18	2.87817E-17
G10090_9466	100043332	Ankrd66	1.213039185	2.31825487	up	0.015459297	0.040121837
G10090_5358	100169864	Gm44504	1.396502097	2.632625115	up	0.000369001	0.00149145
G10090_9771	100502766	Kifc1	-1.310071308	0.403300945	down	0.017925246	0.045527071
G10090_14594	100503043	Armcx4	3.868341823	14.6045077	up	1.69067E-06	1.08118E-05
G10090_5932	100503085	Klhl3	1.295611134	2.454809606	up	0.011596632	0.031301345
G10090_27800	100503386	Tpbgl	-2.528081324	0.173369098	down	0.014098873	0.037129522
G10090_10385	102216272	Ak6	1.004279596	2.005941588	up	2.29423E-08	1.97533E-07
G10090_15730	102635944	Gm33153	1.812508228	3.512524353	up	3.70214E-11	4.48501E-10
G10090_5046	102638514	Gm35060	2.278036367	4.850173535	up	1.72549E-07	1.30119E-06
G10090_8057	105245043	LOC105245043	4.925500687	30.38949271	up	1.75118E-06	1.11774E-05
G10090_31017	105245097	Gm40595	-1.549993379	0.341511632	down	0.002045682	0.006854515
G10090_24021	105245547	Gm40991	-1.338484716	0.39543577	down	0.0007825	0.002899918
G10090_26604	105246138	Gm41476	-1.127162217	0.457815363	down	0.007521843	0.021500362
G10090_8841	105246572	Gm41844	-1.208828928	0.432619642	down	0.000907752	0.003321908
G10090_33782	105246872	Gm42078	-1.549200409	0.341699393	down	0.01171491	0.031550811
G10090_26347	108167700	Gm46139	1.227489915	2.341592314	up	0.000211475	0.000908362
G10090_29297	108167806	Gm46221	-1.386819131	0.382407008	down	0.014915603	0.038883695
G10090_10807	108168067	Gm46403	-1.407528109	0.376957006	down	0.003121027	0.009996073
G10090_26384	108169061	Gm46918	-1.150755929	0.450389179	down	0.012314154	0.032907206
