# Supplementary material for: Guanylate-Binding protein 2b regulates the AMPK/mTOR/ULK1 signalling pathway to induce autophagy during Mycobacterium bovis infection
Source: Virulence. 2022 May 21;13(1):875–89. doi: 10.1080/21505594.2022.2073024 (PMC9132469; doi:10.1080/21505594.2022.2073024)
Supplement: Supplemental Material [file KVIR_A_2073024_SM9990.zip › Supplementary table 3 .pdf]

Up regulation gene significance pathway

path_id	path_name	path_diffgene_count	path_gene_count	enrichment	pvalue	FDR
01100	Metabolic pathways	165	1572	5.304782606	1.97608E-68	6.24442E-66
04668	TNF signaling pathway	52	113	23.25739605	7.13226E-57	1.1269E-54
04060	Cytokine-cytokine receptor interaction	72	293	12.41941286	1.15099E-55	1.21237E-53
05200	Pathways in cancer	80	543	7.446056818	7.43951E-44	5.87721E-42
05169	Epstein-Barr virus infection	52	231	11.37699461	2.79444E-38	1.76609E-36
04064	NF-kappa B signaling pathway	38	105	18.29070671	7.1923E-37	3.72573E-35
04621	NOD-like receptor signaling pathway	49	211	11.73680295	8.2532E-37	3.72573E-35
04010	MAPK signaling pathway	53	294	9.110972328	8.74058E-34	3.45253E-32
05168	Herpes simplex virus 1 infection	63	458	6.952023954	7.083E-33	2.48692E-31
04151	PI3K-Akt signaling pathway	56	359	7.88369414	3.29921E-32	1.04255E-30
04657	IL-17 signaling pathway	33	91	18.32773243	3.70837E-32	1.06531E-30
05167	Kaposi sarcoma-associated herpesvirus infection	46	225	10.33264484	5.83147E-32	1.53562E-30
04061	Viral protein interaction with cytokine and cytokine receptor	33	95	17.55603844	1.96184E-31	4.76877E-30
05418	Fluid shear stress and atherosclerosis	38	146	13.15427537	9.88892E-31	2.23207E-29
05152	Tuberculosis	40	180	11.2311357	2.15881E-29	4.54789E-28
05164	Influenza A	39	173	11.39343535	6.23627E-29	1.23166E-27
04625	C-type lectin receptor signaling pathway	33	112	14.8912826	9.5585E-29	1.77676E-27
05323	Rheumatoid arthritis	29	87	16.84670355	4.03375E-27	7.08147E-26
05132	Salmonella infection	43	253	8.589821178	1.80436E-26	3.00094E-25
04062	Chemokine signaling pathway	38	192	10.00273023	5.35458E-26	8.46024E-25
04630	JAK-STAT signaling pathway	36	169	10.76594073	7.5243E-26	1.13223E-24
04066	HIF-1 signaling pathway	30	114	13.30002912	1.36396E-24	1.95914E-23
05163	Human cytomegalovirus infection	41	256	8.094314596	3.02966E-24	4.16249E-23
04620	Toll-like receptor signaling pathway	28	100	14.15123098	7.86568E-24	1.03565E-22
05171	Coronavirus disease - COVID-19	39	247	7.980017471	7.18588E-23	9.08296E-22
04380	Osteoclast differentiation	29	128	11.45049382	8.55881E-22	1.01329E-20
05133	Pertussis	24	77	15.75276176	8.65782E-22	1.01329E-20
05205	Proteoglycans in cancer	35	205	8.628799379	9.07942E-22	1.02468E-20
05146	Amoebiasis	27	107	12.75311203	1.09711E-21	1.19547E-20
05145	Toxoplasmosis	27	110	12.40529989	2.43504E-21	2.56491E-20
05022	Pathways of neurodegeneration - multiple diseases	50	472	5.353825281	4.26179E-21	4.34427E-20
05142	Chagas disease	26	103	12.75769783	6.42639E-21	6.34606E-20
05202	Transcriptional misregulation in cancer	35	223	7.932304362	1.59751E-20	1.52973E-19
04015	Rap1 signaling pathway	34	214	8.029737206	3.903E-20	3.62749E-19
04210	Apoptosis	28	136	10.4053169	7.2193E-20	6.518E-19
05160	Hepatitis C	30	165	9.189111027	1.36512E-19	1.19827E-18
05166	Human T-cell leukemia virus 1 infection	35	247	7.161554141	4.8385E-19	4.13234E-18
04510	Focal adhesion	32	201	8.04618677	4.98951E-19	4.14917E-18
05134	Legionellosis	20	61	16.57052808	8.59406E-19	6.9634E-18
05165	Human papillomavirus infection	41	362	5.724156179	1.68237E-18	1.32907E-17

04623	Cytosolic DNA-sensing pathway	20	63	16.04447957	1.79035E-18	1.37988E-17
05144	Malaria	19	57	16.84670355	4.7605E-18	3.58171E-17
05162	Measles	27	146	9.346458819	6.46079E-18	4.74793E-17
05235	PD-L1 expression and PD-1 checkpoint pathway in cancer	22	88	12.63502766	9.91663E-18	7.12195E-17
04933	AGE-RAGE signaling pathway in diabetic complications	23	101	11.50913411	1.63533E-17	1.14836E-16
05140	Leishmaniasis	20	70	14.44003161	1.8846E-17	1.29464E-16
04659	Th17 cell differentiation	23	104	11.17713986	3.29888E-17	2.21797E-16
04217	Necroptosis	28	174	8.132891369	6.97144E-17	4.58953E-16
05135	Yersinia infection	25	134	9.429125121	9.52173E-17	6.14055E-16
05161	Hepatitis B	27	163	8.37167477	1.2182E-16	7.69904E-16
04014	Ras signaling pathway	31	232	6.75320444	3.18253E-16	1.97192E-15
05321	Inflammatory bowel disease	18	62	14.67293535	6.33156E-16	3.84764E-15
05010	Alzheimer disease	38	369	5.204672641	7.96759E-16	4.75049E-15
05203	Viral carcinogenesis	30	229	6.620975194	1.72713E-15	1.01069E-14
01230	Biosynthesis of amino acids	19	79	12.15521649	4.37788E-15	2.51529E-14
04920	Adipocytokine signaling pathway	18	71	12.8129858	8.99599E-15	5.07631E-14
04145	Phagosome	26	182	7.220015807	1.88082E-14	1.0427E-13
01240	Biosynthesis of cofactors	24	154	7.876380881	2.85765E-14	1.55693E-13
04810	Regulation of actin cytoskeleton	28	220	6.432377719	3.44134E-14	1.84316E-13
04658	Th1 and Th2 cell differentiation	19	88	10.91206934	3.7305E-14	1.96473E-13
04020	Calcium signaling pathway	29	240	6.106930037	4.54499E-14	2.31648E-13
05170	Human immunodeficiency virus 1 infection	29	240	6.106930037	4.54499E-14	2.31648E-13
05143	African trypanosomiasis	14	39	18.14260382	5.0413E-14	2.52865E-13
05206	MicroRNAs in cancer	32	303	5.337569442	9.1078E-14	4.49698E-13
04622	RIG-I-like receptor signaling pathway	17	70	12.27402687	1.16908E-13	5.68354E-13
05014	Amyotrophic lateral sclerosis	35	369	4.793777433	1.43954E-13	6.89236E-13
04514	Cell adhesion molecules	24	174	6.971049745	4.61768E-13	2.17789E-12
04926	Relaxin signaling pathway	21	129	8.227459873	5.62026E-13	2.61177E-12
05020	Prion disease	29	268	5.46889257	7.9576E-13	3.64435E-12
03050	Proteasome	14	47	15.05450104	9.85322E-13	4.44802E-12
04360	Axon guidance	24	181	6.701451136	1.1159E-12	4.96656E-12
04080	Neuroactive ligand-receptor interaction	33	358	4.658725283	1.70335E-12	7.47582E-12
04940	Type I diabetes mellitus	16	70	11.55202529	1.85143E-12	8.01439E-12
04640	Hematopoietic cell lineage	18	95	9.576020965	2.05697E-12	8.78384E-12
04660	T cell receptor signaling pathway	18	103	8.832252347	8.75587E-12	3.68914E-11
04218	Cellular senescence	23	184	6.317513831	1.21251E-11	5.04148E-11
04666	Fc gamma R-mediated phagocytosis	17	92	9.33893349	1.38976E-11	5.70342E-11
00230	Purine metabolism	20	136	7.432369213	1.50079E-11	6.08014E-11
05222	Small cell lung cancer	17	93	9.23851485	1.66933E-11	6.67731E-11
05230	Central carbon metabolism in cancer	15	69	10.98698058	2.16831E-11	8.56481E-11
00480	Glutathione metabolism	15	71	10.67748817	3.372E-11	1.3155E-10
04216	Ferroptosis	12	40	15.1620332	4.61424E-11	1.77817E-10

05330	Allograft rejection	14	63	11.2311357	8.0322E-11	3.05804E-10
00270	Cysteine and methionine metabolism	13	53	12.39663091	1.09149E-10	4.10607E-10
04931	Insulin resistance	17	110	7.810744373	2.72472E-10	1.01295E-09
05221	Acute myeloid leukemia	14	70	10.10802213	3.65037E-10	1.3413E-09
05012	Parkinson disease	24	247	4.910779982	8.73275E-10	3.1719E-09
05332	Graft-versus-host disease	13	63	10.42891172	1.13082E-09	4.06069E-09
05410	Hypertrophic cardiomyopathy	15	91	8.33078747	1.39758E-09	4.96221E-09
01200	Carbon metabolism	17	124	6.928886138	1.87959E-09	6.59946E-09
04350	TGF-beta signaling pathway	15	95	7.980017471	2.6132E-09	9.07441E-09
04068	FoxO signaling pathway	17	131	6.558640313	4.47755E-09	1.53794E-08
04072	Phospholipase D signaling pathway	18	149	6.105516723	4.76015E-09	1.61743E-08
04670	Leukocyte transendothelial migration	16	118	6.852896359	7.1504E-09	2.40375E-08
04921	Oxytocin signaling pathway	18	153	5.945895371	7.32982E-09	2.43813E-08
05416	Viral myocarditis	14	88	8.040472149	8.72876E-09	2.87322E-08
00100	Steroid biosynthesis	8	20	20.21604426	9.39981E-09	3.06221E-08
04722	Neurotrophin signaling pathway	16	121	6.682989838	1.03923E-08	3.35097E-08
04611	Platelet activation	16	124	6.5213046	1.49353E-08	4.76722E-08
05219	Bladder cancer	10	41	12.32685626	2.29028E-08	7.23729E-08
00010	Glycolysis / Gluconeogenesis	12	67	9.051960116	3.02973E-08	9.47916E-08
05211	Renal cell carcinoma	12	68	8.918843056	3.61269E-08	1.11923E-07
05215	Prostate cancer	14	99	7.147086355	4.21457E-08	1.29301E-07
04024	cAMP signaling pathway	20	215	4.701405642	5.70498E-08	1.72286E-07
05225	Hepatocellular carcinoma	18	174	5.228287309	5.7247E-08	1.72286E-07
04115	p53 signaling pathway	12	72	8.423351775	7.08757E-08	2.1129E-07
04650	Natural killer cell mediated cytotoxicity	15	121	6.265302973	7.92067E-08	2.33919E-07
04512	ECM-receptor interaction	13	88	7.46615271	8.21283E-08	2.40301E-07
04917	Prolactin signaling pathway	12	74	8.195693619	9.76232E-08	2.83018E-07
04928	Parathyroid hormone synthesis, secretion and action	14	108	6.551495825	1.31409E-07	3.77503E-07
04610	Complement and coagulation cascades	13	93	7.06474665	1.62067E-07	4.61379E-07
04530	Tight junction	17	167	5.144801683	1.81686E-07	5.12614E-07
01524	Platinum drug resistance	12	80	7.581016598	2.40358E-07	6.72152E-07
04932	Non-alcoholic fatty liver disease	16	151	5.355243513	2.55912E-07	7.09371E-07
04662	B cell receptor signaling pathway	12	81	7.4874238	2.77085E-07	7.61381E-07
04141	Protein processing in endoplasmic reticulum	17	172	4.995243494	2.8053E-07	7.64201E-07
04935	Growth hormone synthesis, secretion and action	14	116	6.099668527	3.28804E-07	8.88053E-07
01523	Antifolate resistance	8	30	13.47736284	3.66319E-07	9.80989E-07
04012	ErbB signaling pathway	12	84	7.220015807	4.19233E-07	1.11326E-06
05017	Spinocerebellar ataxia	15	141	5.376607516	6.21445E-07	1.63647E-06
04613	Neutrophil extracellular trap formation	18	205	4.437668252	7.10421E-07	1.85531E-06
04071	Sphingolipid signaling pathway	14	124	5.706141525	7.6317E-07	1.97674E-06
00240	Pyrimidine metabolism	10	58	8.713812181	7.85995E-07	2.0193E-06
03320	PPAR signaling pathway	12	89	6.814396942	8.04385E-07	2.04988E-06

00900	Terpenoid backbone biosynthesis	7	23	15.38177281	8.69622E-07	2.1984E-06
05016	Huntington disease	22	302	3.681729915	8.77971E-07	2.20189E-06
04612	Antigen processing and presentation	12	90	6.73868142	9.11462E-07	2.25017E-06
04912	GnRH signaling pathway	12	90	6.73868142	9.11462E-07	2.25017E-06
04390	Hippo signaling pathway	15	157	4.828672992	2.51973E-06	6.17236E-06
04664	Fc epsilon RI signaling pathway	10	66	7.657592523	2.75433E-06	6.69515E-06
00380	Tryptophan metabolism	9	52	8.747326843	3.10612E-06	7.47976E-06
04910	Insulin signaling pathway	14	139	5.090370857	3.12446E-06	7.47976E-06
04270	Vascular smooth muscle contraction	14	143	4.947982861	4.40595E-06	1.04683E-05
04922	Glucagon signaling pathway	12	104	5.831551229	4.46395E-06	1.05269E-05
05150	Staphylococcus aureus infection	13	124	5.298559988	4.8705E-06	1.14006E-05
00760	Nicotinate and nicotinamide metabolism	8	41	9.861485005	4.93068E-06	1.14566E-05
05210	Colorectal cancer	11	88	6.317513831	5.57774E-06	1.28654E-05
04152	AMPK signaling pathway	13	126	5.214455861	5.84474E-06	1.33836E-05
04750	Inflammatory mediator regulation of TRP channels	13	127	5.173397153	6.39395E-06	1.45359E-05
00220	Arginine biosynthesis	6	20	15.1620332	7.25886E-06	1.62681E-05
00532	Glycosaminoglycan biosynthesis - chondroitin sulfate / dermatan sulfate	6	20	15.1620332	7.25886E-06	1.62681E-05
04370	VEGF signaling pathway	9	58	7.842430963	8.08422E-06	1.79902E-05
04740	Olfactory transduction	4	1167	0.173230885	8.24245E-06	1.82141E-05
01522	Endocrine resistance	11	93	5.97786255	9.71069E-06	2.13096E-05
05212	Pancreatic cancer	10	76	6.650014559	1.04231E-05	2.27151E-05
05414	Dilated cardiomyopathy	11	94	5.914268268	1.08018E-05	2.33792E-05
01521	EGFR tyrosine kinase inhibitor resistance	10	79	6.397482361	1.49118E-05	3.17748E-05
05320	Autoimmune thyroid disease	10	79	6.397482361	1.49118E-05	3.17748E-05
04371	Apelin signaling pathway	13	137	4.795776923	1.49824E-05	3.17748E-05
04140	Autophagy - animal	13	138	4.761024916	1.6243E-05	3.41408E-05
05231	Choline metabolism in cancer	11	98	5.672869563	1.63141E-05	3.41408E-05
05204	Chemical carcinogenesis	11	101	5.504368487	2.19286E-05	4.55883E-05
05224	Breast cancer	13	147	4.469533595	3.25394E-05	6.72056E-05
04978	Mineral absorption	8	53	7.628695947	3.70769E-05	7.60799E-05
05226	Gastric cancer	13	150	4.380142923	4.05258E-05	8.26203E-05
00330	Arginine and proline metabolism	8	54	7.4874238	4.27714E-05	8.66395E-05
00982	Drug metabolism - cytochrome P450	9	71	6.406492899	4.49311E-05	9.04345E-05
04261	Adrenergic signaling in cardiomyocytes	13	152	4.322509463	4.67641E-05	9.35282E-05
00260	Glycine, serine and threonine metabolism	7	40	8.844519364	4.93117E-05	9.80032E-05
00980	Metabolism of xenobiotics by cytochrome P450	9	73	6.230972546	5.65396E-05	0.000111666
04150	mTOR signaling pathway	13	156	4.211675888	6.18202E-05	0.000121337
00280	Valine, leucine and isoleucine degradation	8	57	7.093348863	6.44611E-05	0.000125739
04672	Intestinal immune network for IgA production	7	43	8.227459873	8.09422E-05	0.000155962
04975	Fat digestion and absorption	7	43	8.227459873	8.09422E-05	0.000155962
04934	Cushing syndrome	13	162	4.055687892	9.2365E-05	0.000176893
04550	Signaling pathways regulating pluripotency of stem cells	12	140	4.332009484	9.82581E-05	0.000187046

00564	Glycerophospholipid metabolism	10	98	5.157154148	0.000103355	0.000195571
04919	Thyroid hormone signaling pathway	11	120	4.632843476	0.000113914	0.000214266
00561	Glycerolipid metabolism	8	62	6.5213046	0.000120883	0.0002247
01212	Fatty acid metabolism	8	62	6.5213046	0.000120883	0.0002247
04144	Endocytosis	17	272	3.158756916	0.00014622	0.000270207
04930	Type II diabetes mellitus	7	48	7.370432803	0.000169711	0.000311795
04022	cGMP-PKG signaling pathway	13	173	3.797811783	0.000183648	0.000335449
00071	Fatty acid degradation	7	52	6.803476434	0.0002879	0.000522852
04211	Longevity regulating pathway	9	90	5.054011065	0.000304887	0.000550539
04915	Estrogen signaling pathway	11	134	4.148815053	0.000314089	0.000563932
04725	Cholinergic synapse	10	112	4.512509879	0.000325731	0.00058153
00983	Drug metabolism - other enzymes	9	92	4.944141259	0.000362084	0.000642801
04310	Wnt signaling pathway	12	162	3.7437119	0.00040786	0.000720022
00620	Pyruvate metabolism	6	39	7.775401638	0.000443261	0.000778169
00350	Tyrosine metabolism	6	40	7.581016598	0.000512781	0.000895242
05100	Bacterial invasion of epithelial cells	8	76	5.320011647	0.000528406	0.000917452
05412	Arrhythmogenic right ventricular cardiomyopathy	8	77	5.250920587	0.00057952	0.001000701
00450	Selenocompound metabolism	4	17	11.89179074	0.001182631	0.002031041
04974	Protein digestion and absorption	9	108	4.211675888	0.001231278	0.002103156
04146	Peroxisome	8	86	4.701405642	0.001250167	0.002112582
04540	Gap junction	8	86	4.701405642	0.001250167	0.002112582
00565	Ether lipid metabolism	6	48	6.317513831	0.001433245	0.002409071
04720	Long-term potentiation	7	67	5.280310068	0.001447669	0.002420442
04979	Cholesterol metabolism	6	49	6.188584978	0.001606076	0.002671157
02010	ABC transporters	6	50	6.064813278	0.001794751	0.002969326
04972	Pancreatic secretion	9	114	3.990008736	0.001837072	0.003023514
00061	Fatty acid biosynthesis	4	19	10.64002329	0.001866214	0.003039813
00670	One carbon pool by folate	4	19	10.64002329	0.001866214	0.003039813
05218	Melanoma	7	72	4.913621869	0.002251708	0.003648922
04120	Ubiquitin mediated proteolysis	10	145	3.485524872	0.002647891	0.004242948
04918	Thyroid hormone synthesis	7	74	4.780821278	0.002658556	0.004242948
05214	Glioma	7	74	4.780821278	0.002658556	0.004242948
00051	Fructose and mannose metabolism	5	36	7.019459813	0.002727821	0.004331615
03008	Ribosome biogenesis in eukaryotes	9	121	3.759181784	0.002833225	0.004476496
05322	Systemic lupus erythematosus	10	148	3.414872341	0.00310279	0.004878018
05220	Chronic myeloid leukemia	7	76	4.655010191	0.003121552	0.004883219
04721	Synaptic vesicle cycle	7	77	4.594555514	0.003375755	0.005254869
04925	Aldosterone synthesis and secretion	8	102	3.963930247	0.003918688	0.006070125
00250	Alanine, aspartate and glutamate metabolism	5	39	6.479501365	0.00396714	0.006111551
05213	Endometrial cancer	6	58	5.228287309	0.00399878	0.006111551
00360	Phenylalanine metabolism	4	23	8.789584461	0.004003453	0.006111551
05310	Asthma	4	25	8.086417704	0.005542428	0.008420226

00072	Synthesis and degradation of ketone bodies	3	12	12.63502766	0.005951234	0.008998039
05217	Basal cell carcinoma	6	63	4.813343871	0.006175453	0.009292587
00790	Folate biosynthesis	4	26	7.775401638	0.006448218	0.009648471
04911	Insulin secretion	7	86	4.113729937	0.006473025	0.009648471
00601	Glycosphingolipid biosynthesis - lacto and neolacto series	4	27	7.4874238	0.007451944	0.011003806
04392	Hippo signaling pathway - multiple species	4	27	7.4874238	0.007451944	0.011003806
04137	Mitophagy - animal	6	66	4.594555514	0.007855517	0.011545783
05030	Cocaine addiction	5	48	5.264594859	0.010192682	0.014911517
04520	Adherens junction	6	71	4.270995266	0.011394746	0.01659327
00600	Sphingolipid metabolism	5	50	5.054011065	0.012209954	0.017560882
00562	Inositol phosphate metabolism	6	72	4.211675888	0.012225931	0.017560882
05223	Non-small cell lung cancer	6	72	4.211675888	0.012225931	0.017560882
00052	Galactose metabolism	4	32	6.317513831	0.014120703	0.020009606
00630	Glyoxylate and dicarboxylate metabolism	4	32	6.317513831	0.014120703	0.020009606
04215	Apoptosis - multiple species	4	32	6.317513831	0.014120703	0.020009606
03013	RNA transport	10	182	2.776929157	0.014340703	0.020230635
04971	Gastric acid secretion	6	75	4.043208852	0.014991684	0.021054987
04916	Melanogenesis	7	100	3.537807746	0.015216178	0.021181993
04976	Bile secretion	7	100	3.537807746	0.015216178	0.021181993
00030	Pentose phosphate pathway	4	33	6.126074018	0.015819622	0.02192544
04330	Notch signaling pathway	5	54	4.679639875	0.01708286	0.023572855
00500	Starch and sucrose metabolism	4	34	5.945895371	0.017651541	0.024146697
00640	Propanoate metabolism	4	34	5.945895371	0.017651541	0.024146697
04726	Serotonergic synapse	8	131	3.086418971	0.018788181	0.025590798
04728	Dopaminergic synapse	8	135	2.99496952	0.02246831	0.030472043
05216	Thyroid cancer	4	37	5.463795746	0.023990887	0.03239795
00770	Pantothenate and CoA biosynthesis	3	20	7.581016598	0.027410129	0.036701698
01210	2-Oxocarboxylic acid metabolism	3	20	7.581016598	0.027410129	0.036701698
00590	Arachidonic acid metabolism	6	86	3.526054231	0.029182117	0.038746004
04970	Salivary secretion	6	86	3.526054231	0.029182117	0.038746004
04724	Glutamatergic synapse	7	113	3.130803315	0.029493593	0.038995713
04213	Longevity regulating pathway - multiple species	5	62	4.075815375	0.0307245	0.040453925
04913	Ovarian steroidogenesis	5	63	4.011119893	0.032840797	0.043060963
00860	Porphyrin and chlorophyll metabolism	4	42	4.813343871	0.037622727	0.049127197
00740	Riboflavin metabolism	2	8	12.63502766	0.04048015	0.052640854
04110	Cell cycle	7	123	2.87626646	0.045869332	0.059404545
05031	Amphetamine addiction	5	69	3.662326859	0.047689505	0.06150973
04070	Phosphatidylinositol signaling system	6	96	3.158756916	0.048809969	0.062698985
00400	Phenylalanine, tyrosine and tryptophan biosynthesis	2	9	11.2311357	0.051367176	0.065451725
00750	Vitamin B6 metabolism	2	9	11.2311357	0.051367176	0.065451725
04927	Cortisol synthesis and secretion	5	72	3.509729906	0.056588477	0.071815096
00650	Butanoate metabolism	3	28	5.415011855	0.07008234	0.088249733

04924	Renin secretion	5	76	3.32500728	0.070097098	0.088249733
00130	Ubiquinone and other terpenoid-quinone biosynthesis	2	11	9.189111027	0.076449284	0.095486062
00920	Sulfur metabolism	2	11	9.189111027	0.076449284	0.095486062
00410	beta-Alanine metabolism	3	31	4.89097845	0.092062164	0.114534031
03018	RNA degradation	5	84	3.00833992	0.103134586	0.127805997
04714	Thermogenesis	10	230	2.197396115	0.108331888	0.133722174
00533	Glycosaminoglycan biosynthesis - keratan sulfate	2	14	7.220015807	0.121637396	0.149561934
04730	Long-term depression	4	60	3.36934071	0.124268122	0.152204367
04727	GABAergic synapse	5	89	2.839332059	0.128103039	0.1562956
04961	Endocrine and other factor-regulated calcium reabsorption	4	61	3.314105616	0.130974314	0.159184167
00730	Thiamine metabolism	2	15	6.73868142	0.138539334	0.167733447
00140	Steroid hormone biosynthesis	5	92	2.746745144	0.144744841	0.174577747
00310	Lysine degradation	4	64	3.158756916	0.15234021	0.183039948
00603	Glycosphingolipid biosynthesis - globo and isoglobo series	2	16	6.317513831	0.156290662	0.187075186
00910	Nitrogen metabolism	2	17	5.945895371	0.174851199	0.208501807
00513	Various types of N-glycan biosynthesis	3	40	3.790508299	0.177726624	0.211133884
04142	Lysosome	6	131	2.314814228	0.186171368	0.220337649
00514	Other types of O-glycan biosynthesis	3	43	3.526054231	0.212662269	0.250751033
04973	Carbohydrate digestion and absorption	3	48	3.158756916	0.277549253	0.326042989
00520	Amino sugar and nucleotide sugar metabolism	3	50	3.032406639	0.305720124	0.357805775
00290	Valine, leucine and isoleucine biosynthesis	1	4	12.63502766	0.307313769	0.358343731
00534	Glycosaminoglycan biosynthesis - heparan sulfate / heparin	2	24	4.211675888	0.324313067	0.376775475
00592	alpha-Linolenic acid metabolism	2	25	4.043208852	0.348044581	0.402864789
04114	Oocyte meiosis	5	119	2.123534061	0.352349429	0.406359196
00340	Histidine metabolism	2	26	3.887700819	0.372276294	0.427779305
04260	Cardiac muscle contraction	4	87	2.323683248	0.37738729	0.4320811
00524	Neomycin, kanamycin and gentamicin biosynthesis	1	5	10.10802213	0.380382235	0.433937857
04744	Phototransduction	2	27	3.7437119	0.396979154	0.449625135
04966	Collecting duct acid secretion	2	27	3.7437119	0.396979154	0.449625135
04914	Progesterone-mediated oocyte maturation	4	90	2.24622714	0.414289355	0.467555129
00512	Mucin type O-glycan biosynthesis	2	28	3.610007904	0.422125063	0.474702918
05032	Morphine addiction	4	91	2.221543325	0.426943702	0.478419183
03060	Protein export	2	29	3.485524872	0.447686847	0.499890613
04710	Circadian rhythm	2	30	3.36934071	0.473638233	0.527005921
00830	Retinol metabolism	4	97	2.084128274	0.506412301	0.561495744
04713	Circadian entrainment	4	98	2.062861659	0.520223378	0.574792263
05034	Alcoholism	7	205	1.725759876	0.523027761	0.575807845
00020	Citrate cycle (TCA cycle)	2	32	3.158756916	0.526609074	0.575807845
04136	Autophagy - other	2	32	3.158756916	0.526609074	0.575807845
04130	SNARE interactions in vesicular transport	2	33	3.063037009	0.553580264	0.603211598
01040	Biosynthesis of unsaturated fatty acids	2	34	2.972947685	0.580844481	0.63074521
04960	Aldosterone-regulated sodium reabsorption	2	38	2.660005824	0.692400248	0.749309858

04962	Vasopressin-regulated water reabsorption	2	44	2.297277757	0.865093832	0.933002222
04723	Retrograde endocannabinoid signaling	5	148	1.707436171	0.890710676	0.957362496
00604	Glycosphingolipid biosynthesis - ganglio series	1	15	3.36934071	1	1
00510	N-Glycan biosynthesis	2	50	2.021604426	1	1
00591	Linoleic acid metabolism	2	50	2.021604426	1	1
03460	Fanconi anemia pathway	2	51	1.981965124	1	1
04340	Hedgehog signaling pathway	2	52	1.94385041	1	1
00120	Primary bile acid biosynthesis	1	18	2.807783925	1	1
04923	Regulation of lipolysis in adipocytes	2	56	1.805003952	1	1
04929	GnRH secretion	2	63	1.604447957	1	1
04964	Proximal tubule bicarbonate reclamation	1	22	2.297277757	1	1
00515	Mannose type O-glycan biosynthesis	1	23	2.197396115	1	1
04977	Vitamin digestion and absorption	1	24	2.105837944	1	1
04742	Taste transduction	3	92	1.648047086	1	1
00062	Fatty acid elongation	1	29	1.742762436	1	1
03020	RNA polymerase	1	29	1.742762436	1	1
03015	mRNA surveillance pathway	3	102	1.486473843	1	1
00040	Pentose and glucuronate interconversions	1	35	1.444003161	1	1
05340	Primary immunodeficiency	1	36	1.403891963	1	1
03040	Spliceosome	1	134	0.377165005	1	1
05033	Nicotine addiction	1	40	1.263502766	1	1
03022	Basal transcription factors	1	44	1.148638878	1	1
00190	Oxidative phosphorylation	3	133	1.140002496	1	1
00970	Aminoacyl-tRNA biosynthesis	1	66	0.765759252	1	1

Significant up-regulated genes included in the pathway

path_id	path_name	enrichment	pvalue	FDR	gene_id	gene_name
01100	Metabolic pathways	5.304782606	1.97608E-68	6.24442E-66	13121	Cyp51
01100	Metabolic pathways	5.304782606	1.97608E-68	6.24442E-66	66234	Msmo1
01100	Metabolic pathways	5.304782606	1.97608E-68	6.24442E-66	11846	Arg1
01100	Metabolic pathways	5.304782606	1.97608E-68	6.24442E-66	11847	Arg2
01100	Metabolic pathways	5.304782606	1.97608E-68	6.24442E-66	107869	Cth
01100	Metabolic pathways	5.304782606	1.97608E-68	6.24442E-66	229905	Kyat3
01100	Metabolic pathways	5.304782606	1.97608E-68	6.24442E-66	12040	Bckdhb
01100	Metabolic pathways	5.304782606	1.97608E-68	6.24442E-66	56348	Hsd17b12
01100	Metabolic pathways	5.304782606	1.97608E-68	6.24442E-66	20250	Scd2
01100	Metabolic pathways	5.304782606	1.97608E-68	6.24442E-66	100042295	Gm3776
01100	Metabolic pathways	5.304782606	1.97608E-68	6.24442E-66	14857	Gsta1
01100	Metabolic pathways	5.304782606	1.97608E-68	6.24442E-66	14858	Gsta2
01100	Metabolic pathways	5.304782606	1.97608E-68	6.24442E-66	14860	Gsta4
01100	Metabolic pathways	5.304782606	1.97608E-68	6.24442E-66	14863	Gstm2
01100	Metabolic pathways	5.304782606	1.97608E-68	6.24442E-66	11758	Prdx6
01100	Metabolic pathways	5.304782606	1.97608E-68	6.24442E-66	14782	Gsr

01100	Metabolic pathways	5.304782606	1.97608E-68	6.24442E-66	11670	Aldh3a1
01100	Metabolic pathways	5.304782606	1.97608E-68	6.24442E-66	66681	Pgm2
01100	Metabolic pathways	5.304782606	1.97608E-68	6.24442E-66	15277	Hk2
01100	Metabolic pathways	5.304782606	1.97608E-68	6.24442E-66	30955	Pik3cg
01100	Metabolic pathways	5.304782606	1.97608E-68	6.24442E-66	18719	Pip5k1b
01100	Metabolic pathways	5.304782606	1.97608E-68	6.24442E-66	18720	Pip5k1a
01100	Metabolic pathways	5.304782606	1.97608E-68	6.24442E-66	17772	Mtm1
01100	Metabolic pathways	5.304782606	1.97608E-68	6.24442E-66	18798	Plcb4
01100	Metabolic pathways	5.304782606	1.97608E-68	6.24442E-66	17319	Mif
01100	Metabolic pathways	5.304782606	1.97608E-68	6.24442E-66	18126	Nos2
01100	Metabolic pathways	5.304782606	1.97608E-68	6.24442E-66	11655	Alas1
01100	Metabolic pathways	5.304782606	1.97608E-68	6.24442E-66	16828	Ldha
01100	Metabolic pathways	5.304782606	1.97608E-68	6.24442E-66	14854	Gss
01100	Metabolic pathways	5.304782606	1.97608E-68	6.24442E-66	67417	Ears2
01100	Metabolic pathways	5.304782606	1.97608E-68	6.24442E-66	12035	Bcat1
01100	Metabolic pathways	5.304782606	1.97608E-68	6.24442E-66	18950	Pnp
01100	Metabolic pathways	5.304782606	1.97608E-68	6.24442E-66	22271	Upp1
01100	Metabolic pathways	5.304782606	1.97608E-68	6.24442E-66	107569	Nt5c3
01100	Metabolic pathways	5.304782606	1.97608E-68	6.24442E-66	13178	Dck
01100	Metabolic pathways	5.304782606	1.97608E-68	6.24442E-66	230718	Nt5c1a
01100	Metabolic pathways	5.304782606	1.97608E-68	6.24442E-66	23959	Nt5e
01100	Metabolic pathways	5.304782606	1.97608E-68	6.24442E-66	64705	Dpys
01100	Metabolic pathways	5.304782606	1.97608E-68	6.24442E-66	224794	Enpp4
01100	Metabolic pathways	5.304782606	1.97608E-68	6.24442E-66	18578	Pde4b
01100	Metabolic pathways	5.304782606	1.97608E-68	6.24442E-66	238871	Pde4d
01100	Metabolic pathways	5.304782606	1.97608E-68	6.24442E-66	23984	Pde10a
01100	Metabolic pathways	5.304782606	1.97608E-68	6.24442E-66	54369	Nme6
01100	Metabolic pathways	5.304782606	1.97608E-68	6.24442E-66	56738	Mocs1
01100	Metabolic pathways	5.304782606	1.97608E-68	6.24442E-66	18587	Pde6b
01100	Metabolic pathways	5.304782606	1.97608E-68	6.24442E-66	242202	Pde5a
01100	Metabolic pathways	5.304782606	1.97608E-68	6.24442E-66	73707	Gucy2g
01100	Metabolic pathways	5.304782606	1.97608E-68	6.24442E-66	12349	Car2
01100	Metabolic pathways	5.304782606	1.97608E-68	6.24442E-66	71934	Car13
01100	Metabolic pathways	5.304782606	1.97608E-68	6.24442E-66	17448	Mdh2
01100	Metabolic pathways	5.304782606	1.97608E-68	6.24442E-66	13885	Esd
01100	Metabolic pathways	5.304782606	1.97608E-68	6.24442E-66	16365	Acod1
01100	Metabolic pathways	5.304782606	1.97608E-68	6.24442E-66	76238	Grhpr
01100	Metabolic pathways	5.304782606	1.97608E-68	6.24442E-66	170768	Pfkfb3
01100	Metabolic pathways	5.304782606	1.97608E-68	6.24442E-66	14538	Gcnt2
01100	Metabolic pathways	5.304782606	1.97608E-68	6.24442E-66	269181	Mgat4a
01100	Metabolic pathways	5.304782606	1.97608E-68	6.24442E-66	227671	Gbgt1
01100	Metabolic pathways	5.304782606	1.97608E-68	6.24442E-66	69065	Chac1

01100	Metabolic pathways	5.304782606	1.97608E-68	6.24442E-66	15368	Hmox1
01100	Metabolic pathways	5.304782606	1.97608E-68	6.24442E-66	233016	Blvrb
01100	Metabolic pathways	5.304782606	1.97608E-68	6.24442E-66	20441	St3gal3
01100	Metabolic pathways	5.304782606	1.97608E-68	6.24442E-66	14629	Gclc
01100	Metabolic pathways	5.304782606	1.97608E-68	6.24442E-66	14630	Gclm
01100	Metabolic pathways	5.304782606	1.97608E-68	6.24442E-66	11637	Ak2
01100	Metabolic pathways	5.304782606	1.97608E-68	6.24442E-66	11639	Ak4
01100	Metabolic pathways	5.304782606	1.97608E-68	6.24442E-66	72947	Phykpl
01100	Metabolic pathways	5.304782606	1.97608E-68	6.24442E-66	14187	Akr1b8
01100	Metabolic pathways	5.304782606	1.97608E-68	6.24442E-66	18641	Pfkl
01100	Metabolic pathways	5.304782606	1.97608E-68	6.24442E-66	217119	Xylt2
01100	Metabolic pathways	5.304782606	1.97608E-68	6.24442E-66	208715	Hmgcs1
01100	Metabolic pathways	5.304782606	1.97608E-68	6.24442E-66	20810	Srm
01100	Metabolic pathways	5.304782606	1.97608E-68	6.24442E-66	19012	Plpp1
01100	Metabolic pathways	5.304782606	1.97608E-68	6.24442E-66	380921	Dgkh
01100	Metabolic pathways	5.304782606	1.97608E-68	6.24442E-66	67916	Plpp3
01100	Metabolic pathways	5.304782606	1.97608E-68	6.24442E-66	23972	Papss2
01100	Metabolic pathways	5.304782606	1.97608E-68	6.24442E-66	15483	Hsd11b1
01100	Metabolic pathways	5.304782606	1.97608E-68	6.24442E-66	14204	Il4i1
01100	Metabolic pathways	5.304782606	1.97608E-68	6.24442E-66	73988	4930438A08Rik
01100	Metabolic pathways	5.304782606	1.97608E-68	6.24442E-66	15930	Ido1
01100	Metabolic pathways	5.304782606	1.97608E-68	6.24442E-66	209176	Ido2
01100	Metabolic pathways	5.304782606	1.97608E-68	6.24442E-66	17768	Mthfd2
01100	Metabolic pathways	5.304782606	1.97608E-68	6.24442E-66	665563	Mthfd2l
01100	Metabolic pathways	5.304782606	1.97608E-68	6.24442E-66	270685	Mthfd1l
01100	Metabolic pathways	5.304782606	1.97608E-68	6.24442E-66	14137	Fdft1
01100	Metabolic pathways	5.304782606	1.97608E-68	6.24442E-66	18655	Pgk1
01100	Metabolic pathways	5.304782606	1.97608E-68	6.24442E-66	110208	Pgd
01100	Metabolic pathways	5.304782606	1.97608E-68	6.24442E-66	14584	Gfpt2
01100	Metabolic pathways	5.304782606	1.97608E-68	6.24442E-66	11898	Ass1
01100	Metabolic pathways	5.304782606	1.97608E-68	6.24442E-66	236539	Phgdh
01100	Metabolic pathways	5.304782606	1.97608E-68	6.24442E-66	107272	Psat1
01100	Metabolic pathways	5.304782606	1.97608E-68	6.24442E-66	15186	Hdc
01100	Metabolic pathways	5.304782606	1.97608E-68	6.24442E-66	11529	Adh7
01100	Metabolic pathways	5.304782606	1.97608E-68	6.24442E-66	11761	Aox1
01100	Metabolic pathways	5.304782606	1.97608E-68	6.24442E-66	27053	Asns
01100	Metabolic pathways	5.304782606	1.97608E-68	6.24442E-66	110460	Acat2
01100	Metabolic pathways	5.304782606	1.97608E-68	6.24442E-66	224530	Acat3
01100	Metabolic pathways	5.304782606	1.97608E-68	6.24442E-66	14081	Acsl1
01100	Metabolic pathways	5.304782606	1.97608E-68	6.24442E-66	433256	Acsl5
01100	Metabolic pathways	5.304782606	1.97608E-68	6.24442E-66	50790	Acsl4
01100	Metabolic pathways	5.304782606	1.97608E-68	6.24442E-66	94180	Acsbg1

01100	Metabolic pathways	5.304782606	1.97608E-68	6.24442E-66	192156	Mvd
01100	Metabolic pathways	5.304782606	1.97608E-68	6.24442E-66	20775	Sqle
01100	Metabolic pathways	5.304782606	1.97608E-68	6.24442E-66	15490	Hsd17b7
01100	Metabolic pathways	5.304782606	1.97608E-68	6.24442E-66	14433	Gapdh
01100	Metabolic pathways	5.304782606	1.97608E-68	6.24442E-66	22169	Cmpk2
01100	Metabolic pathways	5.304782606	1.97608E-68	6.24442E-66	80914	Uck2
01100	Metabolic pathways	5.304782606	1.97608E-68	6.24442E-66	18783	Pla2g4a
01100	Metabolic pathways	5.304782606	1.97608E-68	6.24442E-66	18784	Pla2g5
01100	Metabolic pathways	5.304782606	1.97608E-68	6.24442E-66	68262	Agpat4
01100	Metabolic pathways	5.304782606	1.97608E-68	6.24442E-66	270084	Lpcat2
01100	Metabolic pathways	5.304782606	1.97608E-68	6.24442E-66	241447	Cers6
01100	Metabolic pathways	5.304782606	1.97608E-68	6.24442E-66	20773	Sptlc2
01100	Metabolic pathways	5.304782606	1.97608E-68	6.24442E-66	11684	Alox12
01100	Metabolic pathways	5.304782606	1.97608E-68	6.24442E-66	13850	Ephx2
01100	Metabolic pathways	5.304782606	1.97608E-68	6.24442E-66	19225	Ptgs2
01100	Metabolic pathways	5.304782606	1.97608E-68	6.24442E-66	64292	Ptges
01100	Metabolic pathways	5.304782606	1.97608E-68	6.24442E-66	12182	Bst1
01100	Metabolic pathways	5.304782606	1.97608E-68	6.24442E-66	12494	Cd38
01100	Metabolic pathways	5.304782606	1.97608E-68	6.24442E-66	192185	Nadk
01100	Metabolic pathways	5.304782606	1.97608E-68	6.24442E-66	16891	Lipg
01100	Metabolic pathways	5.304782606	1.97608E-68	6.24442E-66	319554	Idi1
01100	Metabolic pathways	5.304782606	1.97608E-68	6.24442E-66	110196	Fdps
01100	Metabolic pathways	5.304782606	1.97608E-68	6.24442E-66	14425	Galnt3
01100	Metabolic pathways	5.304782606	1.97608E-68	6.24442E-66	20442	St3gal1
01100	Metabolic pathways	5.304782606	1.97608E-68	6.24442E-66	74241	Chpf
01100	Metabolic pathways	5.304782606	1.97608E-68	6.24442E-66	78923	Chsy3
01100	Metabolic pathways	5.304782606	1.97608E-68	6.24442E-66	108105	B3gnt5
01100	Metabolic pathways	5.304782606	1.97608E-68	6.24442E-66	26878	B3galt2
01100	Metabolic pathways	5.304782606	1.97608E-68	6.24442E-66	20698	Sphk1
01100	Metabolic pathways	5.304782606	1.97608E-68	6.24442E-66	18648	Pgam1
01100	Metabolic pathways	5.304782606	1.97608E-68	6.24442E-66	71776	Tha1
01100	Metabolic pathways	5.304782606	1.97608E-68	6.24442E-66	13806	Eno1
01100	Metabolic pathways	5.304782606	1.97608E-68	6.24442E-66	433182	Eno1b
01100	Metabolic pathways	5.304782606	1.97608E-68	6.24442E-66	217214	Nags
01100	Metabolic pathways	5.304782606	1.97608E-68	6.24442E-66	14933	Gk
01100	Metabolic pathways	5.304782606	1.97608E-68	6.24442E-66	18563	Pcx
01100	Metabolic pathways	5.304782606	1.97608E-68	6.24442E-66	102580	Alg9
01100	Metabolic pathways	5.304782606	1.97608E-68	6.24442E-66	21991	Tpi1
01100	Metabolic pathways	5.304782606	1.97608E-68	6.24442E-66	12850	Coq7
01100	Metabolic pathways	5.304782606	1.97608E-68	6.24442E-66	14042	Ext1
01100	Metabolic pathways	5.304782606	1.97608E-68	6.24442E-66	103142	Rdh9
01100	Metabolic pathways	5.304782606	1.97608E-68	6.24442E-66	77974	Rdh12

01100	Metabolic pathways	5.304782606	1.97608E-68	6.24442E-66	59027	Nampt
01100	Metabolic pathways	5.304782606	1.97608E-68	6.24442E-66	18263	Odc1
01100	Metabolic pathways	5.304782606	1.97608E-68	6.24442E-66	109652	Acy1
01100	Metabolic pathways	5.304782606	1.97608E-68	6.24442E-66	14936	Gys1
01100	Metabolic pathways	5.304782606	1.97608E-68	6.24442E-66	74185	Gbe1
01100	Metabolic pathways	5.304782606	1.97608E-68	6.24442E-66	18104	Nqo1
01100	Metabolic pathways	5.304782606	1.97608E-68	6.24442E-66	14528	Gch1
01100	Metabolic pathways	5.304782606	1.97608E-68	6.24442E-66	11717	Ampd3
01100	Metabolic pathways	5.304782606	1.97608E-68	6.24442E-66	102216272	Ak6
01100	Metabolic pathways	5.304782606	1.97608E-68	6.24442E-66	76974	Urah
01100	Metabolic pathways	5.304782606	1.97608E-68	6.24442E-66	11433	Acp5
01100	Metabolic pathways	5.304782606	1.97608E-68	6.24442E-66	268566	Gphn
01100	Metabolic pathways	5.304782606	1.97608E-68	6.24442E-66	14381	G6pdx
01100	Metabolic pathways	5.304782606	1.97608E-68	6.24442E-66	59010	Sqor
01100	Metabolic pathways	5.304782606	1.97608E-68	6.24442E-66	18451	P4ha1
01100	Metabolic pathways	5.304782606	1.97608E-68	6.24442E-66	18452	P4ha2
01100	Metabolic pathways	5.304782606	1.97608E-68	6.24442E-66	15357	Hmgcr
01100	Metabolic pathways	5.304782606	1.97608E-68	6.24442E-66	12856	Cox17
01100	Metabolic pathways	5.304782606	1.97608E-68	6.24442E-66	11973	Atp6v1e1
01100	Metabolic pathways	5.304782606	1.97608E-68	6.24442E-66	67956	Kmt5a
01100	Metabolic pathways	5.304782606	1.97608E-68	6.24442E-66	228608	Smox
01100	Metabolic pathways	5.304782606	1.97608E-68	6.24442E-66	74754	Dhcr24
01100	Metabolic pathways	5.304782606	1.97608E-68	6.24442E-66	235293	Sc5d
04668	TNF signaling pathway	23.25739605	7.13226E-57	1.1269E-54	22029	Traf1
04668	TNF signaling pathway	23.25739605	7.13226E-57	1.1269E-54	15953	Ifi47
04668	TNF signaling pathway	23.25739605	7.13226E-57	1.1269E-54	21929	Tnfaip3
04668	TNF signaling pathway	23.25739605	7.13226E-57	1.1269E-54	17395	Mmp9
04668	TNF signaling pathway	23.25739605	7.13226E-57	1.1269E-54	17387	Mmp14
04668	TNF signaling pathway	23.25739605	7.13226E-57	1.1269E-54	16477	Junb
04668	TNF signaling pathway	23.25739605	7.13226E-57	1.1269E-54	17392	Mmp3
04668	TNF signaling pathway	23.25739605	7.13226E-57	1.1269E-54	257632	Nod2
04668	TNF signaling pathway	23.25739605	7.13226E-57	1.1269E-54	15894	Icam1
04668	TNF signaling pathway	23.25739605	7.13226E-57	1.1269E-54	13614	Edn1
04668	TNF signaling pathway	23.25739605	7.13226E-57	1.1269E-54	22341	Vegfc
04668	TNF signaling pathway	23.25739605	7.13226E-57	1.1269E-54	21938	Tnfrsf1b
04668	TNF signaling pathway	23.25739605	7.13226E-57	1.1269E-54	18708	Pik3r1
04668	TNF signaling pathway	23.25739605	7.13226E-57	1.1269E-54	56532	Ripk3
04668	TNF signaling pathway	23.25739605	7.13226E-57	1.1269E-54	74568	Mlkl
04668	TNF signaling pathway	23.25739605	7.13226E-57	1.1269E-54	12633	Cflar
04668	TNF signaling pathway	23.25739605	7.13226E-57	1.1269E-54	69601	Dab2ip
04668	TNF signaling pathway	23.25739605	7.13226E-57	1.1269E-54	16362	Irf1
04668	TNF signaling pathway	23.25739605	7.13226E-57	1.1269E-54	231991	Creb5

04668	TNF signaling pathway	23.25739605	7.13226E-57	1.1269E-54	12608 Cebpb
04668	TNF signaling pathway	23.25739605	7.13226E-57	1.1269E-54	12369 Casp7
04668	TNF signaling pathway	23.25739605	7.13226E-57	1.1269E-54	20293 Ccl12
04668	TNF signaling pathway	23.25739605	7.13226E-57	1.1269E-54	22329 Vcam1
04668	TNF signaling pathway	23.25739605	7.13226E-57	1.1269E-54	19225 Ptgs2
04668	TNF signaling pathway	23.25739605	7.13226E-57	1.1269E-54	14825 Cxcl1
04668	TNF signaling pathway	23.25739605	7.13226E-57	1.1269E-54	20310 Cxcl2
04668	TNF signaling pathway	23.25739605	7.13226E-57	1.1269E-54	330122 Cxcl3
04668	TNF signaling pathway	23.25739605	7.13226E-57	1.1269E-54	20311 Cxcl5
04668	TNF signaling pathway	23.25739605	7.13226E-57	1.1269E-54	15945 Cxcl10
04668	TNF signaling pathway	23.25739605	7.13226E-57	1.1269E-54	12977 Csf1
04668	TNF signaling pathway	23.25739605	7.13226E-57	1.1269E-54	12981 Csf2
04668	TNF signaling pathway	23.25739605	7.13226E-57	1.1269E-54	14102 Fas
04668	TNF signaling pathway	23.25739605	7.13226E-57	1.1269E-54	16449 Jag1
04668	TNF signaling pathway	23.25739605	7.13226E-57	1.1269E-54	16176 Il1b
04668	TNF signaling pathway	23.25739605	7.13226E-57	1.1269E-54	16193 Il6
04668	TNF signaling pathway	23.25739605	7.13226E-57	1.1269E-54	20296 Ccl2
04668	TNF signaling pathway	23.25739605	7.13226E-57	1.1269E-54	20304 Ccl5
04668	TNF signaling pathway	23.25739605	7.13226E-57	1.1269E-54	12702 Socs3
04668	TNF signaling pathway	23.25739605	7.13226E-57	1.1269E-54	26399 Map2k6
04668	TNF signaling pathway	23.25739605	7.13226E-57	1.1269E-54	26415 Mapk13
04668	TNF signaling pathway	23.25739605	7.13226E-57	1.1269E-54	18035 Nfkbia
04668	TNF signaling pathway	23.25739605	7.13226E-57	1.1269E-54	18033 Nfkb1
04668	TNF signaling pathway	23.25739605	7.13226E-57	1.1269E-54	26410 Map3k8
04668	TNF signaling pathway	23.25739605	7.13226E-57	1.1269E-54	56613 Rps6ka4
04668	TNF signaling pathway	23.25739605	7.13226E-57	1.1269E-54	11911 Atf4
04668	TNF signaling pathway	23.25739605	7.13226E-57	1.1269E-54	22033 Traf5
04668	TNF signaling pathway	23.25739605	7.13226E-57	1.1269E-54	11796 Birc3
04668	TNF signaling pathway	23.25739605	7.13226E-57	1.1269E-54	21926 Tnf
04668	TNF signaling pathway	23.25739605	7.13226E-57	1.1269E-54	22030 Traf2
04668	TNF signaling pathway	23.25739605	7.13226E-57	1.1269E-54	16878 Lif
04668	TNF signaling pathway	23.25739605	7.13226E-57	1.1269E-54	12051 Bcl3
04668	TNF signaling pathway	23.25739605	7.13226E-57	1.1269E-54	16992 Lta
04060	Cytokine-cytokine receptor interaction	12.41941286	1.15099E-55	1.21237E-53	21938 Tnfrsf1b
04060	Cytokine-cytokine receptor interaction	12.41941286	1.15099E-55	1.21237E-53	56744 Pf4
04060	Cytokine-cytokine receptor interaction	12.41941286	1.15099E-55	1.21237E-53	66102 Cxcl16
04060	Cytokine-cytokine receptor interaction	12.41941286	1.15099E-55	1.21237E-53	18383 Tnfrsf11b
04060	Cytokine-cytokine receptor interaction	12.41941286	1.15099E-55	1.21237E-53	20311 Cxcl5
04060	Cytokine-cytokine receptor interaction	12.41941286	1.15099E-55	1.21237E-53	57349 Ppbp
04060	Cytokine-cytokine receptor interaction	12.41941286	1.15099E-55	1.21237E-53	15945 Cxcl10
04060	Cytokine-cytokine receptor interaction	12.41941286	1.15099E-55	1.21237E-53	17329 Cxcl9
04060	Cytokine-cytokine receptor interaction	12.41941286	1.15099E-55	1.21237E-53	50930 Tnfsf14

04060	Cytokine-cytokine receptor interaction	12.41941286	1.15099E-55	1.21237E-53	16992	Lta
04060	Cytokine-cytokine receptor interaction	12.41941286	1.15099E-55	1.21237E-53	22164	Tnfsf4
04060	Cytokine-cytokine receptor interaction	12.41941286	1.15099E-55	1.21237E-53	21950	Tnfsf9
04060	Cytokine-cytokine receptor interaction	12.41941286	1.15099E-55	1.21237E-53	23832	Xcr1
04060	Cytokine-cytokine receptor interaction	12.41941286	1.15099E-55	1.21237E-53	16190	Il4ra
04060	Cytokine-cytokine receptor interaction	12.41941286	1.15099E-55	1.21237E-53	12981	Csf2
04060	Cytokine-cytokine receptor interaction	12.41941286	1.15099E-55	1.21237E-53	16161	Il12rb1
04060	Cytokine-cytokine receptor interaction	12.41941286	1.15099E-55	1.21237E-53	209590	Il23r
04060	Cytokine-cytokine receptor interaction	12.41941286	1.15099E-55	1.21237E-53	16164	Il13ra1
04060	Cytokine-cytokine receptor interaction	12.41941286	1.15099E-55	1.21237E-53	12986	Csf3r
04060	Cytokine-cytokine receptor interaction	12.41941286	1.15099E-55	1.21237E-53	83430	Il23a
04060	Cytokine-cytokine receptor interaction	12.41941286	1.15099E-55	1.21237E-53	16159	Il12a
04060	Cytokine-cytokine receptor interaction	12.41941286	1.15099E-55	1.21237E-53	16160	Il12b
04060	Cytokine-cytokine receptor interaction	12.41941286	1.15099E-55	1.21237E-53	12985	Csf3
04060	Cytokine-cytokine receptor interaction	12.41941286	1.15099E-55	1.21237E-53	329244	Il19
04060	Cytokine-cytokine receptor interaction	12.41941286	1.15099E-55	1.21237E-53	16153	Il10
04060	Cytokine-cytokine receptor interaction	12.41941286	1.15099E-55	1.21237E-53	15978	Ifng
04060	Cytokine-cytokine receptor interaction	12.41941286	1.15099E-55	1.21237E-53	14825	Cxcl1
04060	Cytokine-cytokine receptor interaction	12.41941286	1.15099E-55	1.21237E-53	20310	Cxcl2
04060	Cytokine-cytokine receptor interaction	12.41941286	1.15099E-55	1.21237E-53	330122	Cxcl3
04060	Cytokine-cytokine receptor interaction	12.41941286	1.15099E-55	1.21237E-53	20303	Ccl4
04060	Cytokine-cytokine receptor interaction	12.41941286	1.15099E-55	1.21237E-53	20302	Ccl3
04060	Cytokine-cytokine receptor interaction	12.41941286	1.15099E-55	1.21237E-53	12775	Ccr7
04060	Cytokine-cytokine receptor interaction	12.41941286	1.15099E-55	1.21237E-53	213208	Il20rb
04060	Cytokine-cytokine receptor interaction	12.41941286	1.15099E-55	1.21237E-53	11479	Acvr1b
04060	Cytokine-cytokine receptor interaction	12.41941286	1.15099E-55	1.21237E-53	12983	Csf2rb
04060	Cytokine-cytokine receptor interaction	12.41941286	1.15099E-55	1.21237E-53	21933	Tnfrsf10b
04060	Cytokine-cytokine receptor interaction	12.41941286	1.15099E-55	1.21237E-53	22035	Tnfsf10
04060	Cytokine-cytokine receptor interaction	12.41941286	1.15099E-55	1.21237E-53	326623	Tnfsf15
04060	Cytokine-cytokine receptor interaction	12.41941286	1.15099E-55	1.21237E-53	21926	Tnf
04060	Cytokine-cytokine receptor interaction	12.41941286	1.15099E-55	1.21237E-53	20299	Ccl22
04060	Cytokine-cytokine receptor interaction	12.41941286	1.15099E-55	1.21237E-53	20304	Ccl5
04060	Cytokine-cytokine receptor interaction	12.41941286	1.15099E-55	1.21237E-53	246779	Il27
04060	Cytokine-cytokine receptor interaction	12.41941286	1.15099E-55	1.21237E-53	50498	Ebi3
04060	Cytokine-cytokine receptor interaction	12.41941286	1.15099E-55	1.21237E-53	16181	Il1rn
04060	Cytokine-cytokine receptor interaction	12.41941286	1.15099E-55	1.21237E-53	107527	Il1rl2
04060	Cytokine-cytokine receptor interaction	12.41941286	1.15099E-55	1.21237E-53	56221	Ccl24
04060	Cytokine-cytokine receptor interaction	12.41941286	1.15099E-55	1.21237E-53	20308	Ccl9
04060	Cytokine-cytokine receptor interaction	12.41941286	1.15099E-55	1.21237E-53	12768	Ccr1
04060	Cytokine-cytokine receptor interaction	12.41941286	1.15099E-55	1.21237E-53	20306	Ccl7
04060	Cytokine-cytokine receptor interaction	12.41941286	1.15099E-55	1.21237E-53	12769	Ccr9
04060	Cytokine-cytokine receptor interaction	12.41941286	1.15099E-55	1.21237E-53	20293	Ccl12

04060	Cytokine-cytokine receptor interaction	12.41941286	1.15099E-55	1.21237E-53	20296	Ccl2
04060	Cytokine-cytokine receptor interaction	12.41941286	1.15099E-55	1.21237E-53	16193	Il6
04060	Cytokine-cytokine receptor interaction	12.41941286	1.15099E-55	1.21237E-53	16175	Il1a
04060	Cytokine-cytokine receptor interaction	12.41941286	1.15099E-55	1.21237E-53	12156	Bmp2
04060	Cytokine-cytokine receptor interaction	12.41941286	1.15099E-55	1.21237E-53	16323	Inhba
04060	Cytokine-cytokine receptor interaction	12.41941286	1.15099E-55	1.21237E-53	320100	Relt
04060	Cytokine-cytokine receptor interaction	12.41941286	1.15099E-55	1.21237E-53	21942	Tnfrsf9
04060	Cytokine-cytokine receptor interaction	12.41941286	1.15099E-55	1.21237E-53	12977	Csf1
04060	Cytokine-cytokine receptor interaction	12.41941286	1.15099E-55	1.21237E-53	18049	Ngf
04060	Cytokine-cytokine receptor interaction	12.41941286	1.15099E-55	1.21237E-53	23886	Gdf15
04060	Cytokine-cytokine receptor interaction	12.41941286	1.15099E-55	1.21237E-53	16847	Lepr
04060	Cytokine-cytokine receptor interaction	12.41941286	1.15099E-55	1.21237E-53	16165	Il13ra2
04060	Cytokine-cytokine receptor interaction	12.41941286	1.15099E-55	1.21237E-53	16878	Lif
04060	Cytokine-cytokine receptor interaction	12.41941286	1.15099E-55	1.21237E-53	18413	Osm
04060	Cytokine-cytokine receptor interaction	12.41941286	1.15099E-55	1.21237E-53	16176	Il1b
04060	Cytokine-cytokine receptor interaction	12.41941286	1.15099E-55	1.21237E-53	21941	Tnfrsf8
04060	Cytokine-cytokine receptor interaction	12.41941286	1.15099E-55	1.21237E-53	21939	Cd40
04060	Cytokine-cytokine receptor interaction	12.41941286	1.15099E-55	1.21237E-53	14102	Fas
04060	Cytokine-cytokine receptor interaction	12.41941286	1.15099E-55	1.21237E-53	16177	Il1r1
04060	Cytokine-cytokine receptor interaction	12.41941286	1.15099E-55	1.21237E-53	12166	Bmpr1a
04060	Cytokine-cytokine receptor interaction	12.41941286	1.15099E-55	1.21237E-53	16173	Il18
05200	Pathways in cancer	7.446056818	7.43951E-44	5.87721E-42	22027	Hsp90b1
05200	Pathways in cancer	7.446056818	7.43951E-44	5.87721E-42	18033	Nfkb1
05200	Pathways in cancer	7.446056818	7.43951E-44	5.87721E-42	15368	Hmox1
05200	Pathways in cancer	7.446056818	7.43951E-44	5.87721E-42	56717	Mtor
05200	Pathways in cancer	7.446056818	7.43951E-44	5.87721E-42	20846	Stat1
05200	Pathways in cancer	7.446056818	7.43951E-44	5.87721E-42	18035	Nfkbia
05200	Pathways in cancer	7.446056818	7.43951E-44	5.87721E-42	15519	Hsp90aa1
05200	Pathways in cancer	7.446056818	7.43951E-44	5.87721E-42	14745	Lpar1
05200	Pathways in cancer	7.446056818	7.43951E-44	5.87721E-42	12062	Bdkrb2
05200	Pathways in cancer	7.446056818	7.43951E-44	5.87721E-42	13618	Ednrb
05200	Pathways in cancer	7.446056818	7.43951E-44	5.87721E-42	14701	Gng12
05200	Pathways in cancer	7.446056818	7.43951E-44	5.87721E-42	18798	Plcb4
05200	Pathways in cancer	7.446056818	7.43951E-44	5.87721E-42	19419	Rasgrp1
05200	Pathways in cancer	7.446056818	7.43951E-44	5.87721E-42	16449	Jag1
05200	Pathways in cancer	7.446056818	7.43951E-44	5.87721E-42	18128	Notch1
05200	Pathways in cancer	7.446056818	7.43951E-44	5.87721E-42	19225	Ptgs2
05200	Pathways in cancer	7.446056818	7.43951E-44	5.87721E-42	18126	Nos2
05200	Pathways in cancer	7.446056818	7.43951E-44	5.87721E-42	11796	Birc3
05200	Pathways in cancer	7.446056818	7.43951E-44	5.87721E-42	22029	Traf1
05200	Pathways in cancer	7.446056818	7.43951E-44	5.87721E-42	22030	Traf2
05200	Pathways in cancer	7.446056818	7.43951E-44	5.87721E-42	22033	Traf5

05200	Pathways in cancer	7.446056818	7.43951E-44	5.87721E-42	18654	Pgf
05200	Pathways in cancer	7.446056818	7.43951E-44	5.87721E-42	22339	Vegfa
05200	Pathways in cancer	7.446056818	7.43951E-44	5.87721E-42	22341	Vegfc
05200	Pathways in cancer	7.446056818	7.43951E-44	5.87721E-42	20525	Slc2a1
05200	Pathways in cancer	7.446056818	7.43951E-44	5.87721E-42	23871	Ets1
05200	Pathways in cancer	7.446056818	7.43951E-44	5.87721E-42	17390	Mmp2
05200	Pathways in cancer	7.446056818	7.43951E-44	5.87721E-42	17395	Mmp9
05200	Pathways in cancer	7.446056818	7.43951E-44	5.87721E-42	83996	Mmp1b
05200	Pathways in cancer	7.446056818	7.43951E-44	5.87721E-42	12986	Csf3r
05200	Pathways in cancer	7.446056818	7.43951E-44	5.87721E-42	16193	Il6
05200	Pathways in cancer	7.446056818	7.43951E-44	5.87721E-42	19015	Ppard
05200	Pathways in cancer	7.446056818	7.43951E-44	5.87721E-42	14164	Fgf1
05200	Pathways in cancer	7.446056818	7.43951E-44	5.87721E-42	14062	F2r
05200	Pathways in cancer	7.446056818	7.43951E-44	5.87721E-42	16452	Jak2
05200	Pathways in cancer	7.446056818	7.43951E-44	5.87721E-42	13649	Egfr
05200	Pathways in cancer	7.446056818	7.43951E-44	5.87721E-42	17295	Met
05200	Pathways in cancer	7.446056818	7.43951E-44	5.87721E-42	13197	Gadd45a
05200	Pathways in cancer	7.446056818	7.43951E-44	5.87721E-42	17873	Gadd45b
05200	Pathways in cancer	7.446056818	7.43951E-44	5.87721E-42	108058	Camk2d
05200	Pathways in cancer	7.446056818	7.43951E-44	5.87721E-42	12369	Casp7
05200	Pathways in cancer	7.446056818	7.43951E-44	5.87721E-42	18712	Pim1
05200	Pathways in cancer	7.446056818	7.43951E-44	5.87721E-42	18034	Nfkb2
05200	Pathways in cancer	7.446056818	7.43951E-44	5.87721E-42	18104	Nqo1
05200	Pathways in cancer	7.446056818	7.43951E-44	5.87721E-42	100042295	Gm3776
05200	Pathways in cancer	7.446056818	7.43951E-44	5.87721E-42	14857	Gsta1
05200	Pathways in cancer	7.446056818	7.43951E-44	5.87721E-42	14858	Gsta2
05200	Pathways in cancer	7.446056818	7.43951E-44	5.87721E-42	14860	Gsta4
05200	Pathways in cancer	7.446056818	7.43951E-44	5.87721E-42	14863	Gstm2
05200	Pathways in cancer	7.446056818	7.43951E-44	5.87721E-42	50493	Txnrd1
05200	Pathways in cancer	7.446056818	7.43951E-44	5.87721E-42	16190	Il4ra
05200	Pathways in cancer	7.446056818	7.43951E-44	5.87721E-42	209590	Il23r
05200	Pathways in cancer	7.446056818	7.43951E-44	5.87721E-42	20847	Stat2
05200	Pathways in cancer	7.446056818	7.43951E-44	5.87721E-42	13614	Edn1
05200	Pathways in cancer	7.446056818	7.43951E-44	5.87721E-42	14102	Fas
05200	Pathways in cancer	7.446056818	7.43951E-44	5.87721E-42	18715	Pim2
05200	Pathways in cancer	7.446056818	7.43951E-44	5.87721E-42	15978	Ifng
05200	Pathways in cancer	7.446056818	7.43951E-44	5.87721E-42	16159	Il12a
05200	Pathways in cancer	7.446056818	7.43951E-44	5.87721E-42	16160	Il12b
05200	Pathways in cancer	7.446056818	7.43951E-44	5.87721E-42	83430	Il23a
05200	Pathways in cancer	7.446056818	7.43951E-44	5.87721E-42	12983	Csf2rb
05200	Pathways in cancer	7.446056818	7.43951E-44	5.87721E-42	16161	Il12rb1
05200	Pathways in cancer	7.446056818	7.43951E-44	5.87721E-42	16164	Il13ra1

05200	Pathways in cancer	7.446056818	7.43951E-44	5.87721E-42	13063	Cycs
05200	Pathways in cancer	7.446056818	7.43951E-44	5.87721E-42	12444	Ccnd2
05200	Pathways in cancer	7.446056818	7.43951E-44	5.87721E-42	12575	Cdkn1a
05200	Pathways in cancer	7.446056818	7.43951E-44	5.87721E-42	12579	Cdkn2b
05200	Pathways in cancer	7.446056818	7.43951E-44	5.87721E-42	16782	Lamc2
05200	Pathways in cancer	7.446056818	7.43951E-44	5.87721E-42	12122	Bid
05200	Pathways in cancer	7.446056818	7.43951E-44	5.87721E-42	22420	Wnt6
05200	Pathways in cancer	7.446056818	7.43951E-44	5.87721E-42	12827	Col4a2
05200	Pathways in cancer	7.446056818	7.43951E-44	5.87721E-42	11864	Arnt2
05200	Pathways in cancer	7.446056818	7.43951E-44	5.87721E-42	14362	Fzd1
05200	Pathways in cancer	7.446056818	7.43951E-44	5.87721E-42	18708	Pik3r1
05200	Pathways in cancer	7.446056818	7.43951E-44	5.87721E-42	15251	Hif1a
05200	Pathways in cancer	7.446056818	7.43951E-44	5.87721E-42	19730	Ralgds
05200	Pathways in cancer	7.446056818	7.43951E-44	5.87721E-42	17869	Myc
05200	Pathways in cancer	7.446056818	7.43951E-44	5.87721E-42	112407	Egln3
05200	Pathways in cancer	7.446056818	7.43951E-44	5.87721E-42	12156	Bmp2
05200	Pathways in cancer	7.446056818	7.43951E-44	5.87721E-42	67923	Eloc
05169	Epstein-Barr virus infection	11.37699461	2.79444E-38	1.76609E-36	16408	Itgal
05169	Epstein-Barr virus infection	11.37699461	2.79444E-38	1.76609E-36	18033	Nfkb1
05169	Epstein-Barr virus infection	11.37699461	2.79444E-38	1.76609E-36	18034	Nfkb2
05169	Epstein-Barr virus infection	11.37699461	2.79444E-38	1.76609E-36	21939	Cd40
05169	Epstein-Barr virus infection	11.37699461	2.79444E-38	1.76609E-36	22030	Traf2
05169	Epstein-Barr virus infection	11.37699461	2.79444E-38	1.76609E-36	20846	Stat1
05169	Epstein-Barr virus infection	11.37699461	2.79444E-38	1.76609E-36	19698	Relb
05169	Epstein-Barr virus infection	11.37699461	2.79444E-38	1.76609E-36	24088	Tlr2
05169	Epstein-Barr virus infection	11.37699461	2.79444E-38	1.76609E-36	23960	Oas1g
05169	Epstein-Barr virus infection	11.37699461	2.79444E-38	1.76609E-36	15945	Cxcl10
05169	Epstein-Barr virus infection	11.37699461	2.79444E-38	1.76609E-36	100038882	Isg15
05169	Epstein-Barr virus infection	11.37699461	2.79444E-38	1.76609E-36	14102	Fas
05169	Epstein-Barr virus infection	11.37699461	2.79444E-38	1.76609E-36	13063	Cycs
05169	Epstein-Barr virus infection	11.37699461	2.79444E-38	1.76609E-36	12122	Bid
05169	Epstein-Barr virus infection	11.37699461	2.79444E-38	1.76609E-36	12503	Cd247
05169	Epstein-Barr virus infection	11.37699461	2.79444E-38	1.76609E-36	56436	Adrm1
05169	Epstein-Barr virus infection	11.37699461	2.79444E-38	1.76609E-36	14990	H2-M2
05169	Epstein-Barr virus infection	11.37699461	2.79444E-38	1.76609E-36	15018	H2-Q7
05169	Epstein-Barr virus infection	11.37699461	2.79444E-38	1.76609E-36	15024	H2-T10
05169	Epstein-Barr virus infection	11.37699461	2.79444E-38	1.76609E-36	15039	H2-T22
05169	Epstein-Barr virus infection	11.37699461	2.79444E-38	1.76609E-36	12317	Calr
05169	Epstein-Barr virus infection	11.37699461	2.79444E-38	1.76609E-36	60406	Sap30
05169	Epstein-Barr virus infection	11.37699461	2.79444E-38	1.76609E-36	12399	Runx3
05169	Epstein-Barr virus infection	11.37699461	2.79444E-38	1.76609E-36	12575	Cdkn1a
05169	Epstein-Barr virus infection	11.37699461	2.79444E-38	1.76609E-36	13197	Gadd45a

05169	Epstein-Barr virus infection	11.37699461	2.79444E-38	1.76609E-36	17873	Gadd45b
05169	Epstein-Barr virus infection	11.37699461	2.79444E-38	1.76609E-36	12444	Ccnd2
05169	Epstein-Barr virus infection	11.37699461	2.79444E-38	1.76609E-36	19181	Psmc2
05169	Epstein-Barr virus infection	11.37699461	2.79444E-38	1.76609E-36	21926	Tnf
05169	Epstein-Barr virus infection	11.37699461	2.79444E-38	1.76609E-36	56489	Ikbke
05169	Epstein-Barr virus infection	11.37699461	2.79444E-38	1.76609E-36	54123	Irf7
05169	Epstein-Barr virus infection	11.37699461	2.79444E-38	1.76609E-36	20847	Stat2
05169	Epstein-Barr virus infection	11.37699461	2.79444E-38	1.76609E-36	18036	Nfkbib
05169	Epstein-Barr virus infection	11.37699461	2.79444E-38	1.76609E-36	16193	Il6
05169	Epstein-Barr virus infection	11.37699461	2.79444E-38	1.76609E-36	22033	Traf5
05169	Epstein-Barr virus infection	11.37699461	2.79444E-38	1.76609E-36	18035	Nfkbia
05169	Epstein-Barr virus infection	11.37699461	2.79444E-38	1.76609E-36	21929	Tnfaip3
05169	Epstein-Barr virus infection	11.37699461	2.79444E-38	1.76609E-36	18037	Nfkbie
05169	Epstein-Barr virus infection	11.37699461	2.79444E-38	1.76609E-36	56480	Tbk1
05169	Epstein-Barr virus infection	11.37699461	2.79444E-38	1.76609E-36	19106	Eif2ak2
05169	Epstein-Barr virus infection	11.37699461	2.79444E-38	1.76609E-36	17096	Lyn
05169	Epstein-Barr virus infection	11.37699461	2.79444E-38	1.76609E-36	26399	Map2k6
05169	Epstein-Barr virus infection	11.37699461	2.79444E-38	1.76609E-36	59029	Psmd14
05169	Epstein-Barr virus infection	11.37699461	2.79444E-38	1.76609E-36	15894	Icam1
05169	Epstein-Barr virus infection	11.37699461	2.79444E-38	1.76609E-36	69077	Psmd11
05169	Epstein-Barr virus infection	11.37699461	2.79444E-38	1.76609E-36	20963	Syk
05169	Epstein-Barr virus infection	11.37699461	2.79444E-38	1.76609E-36	26415	Mapk13
05169	Epstein-Barr virus infection	11.37699461	2.79444E-38	1.76609E-36	18708	Pik3r1
05169	Epstein-Barr virus infection	11.37699461	2.79444E-38	1.76609E-36	17869	Myc
05169	Epstein-Barr virus infection	11.37699461	2.79444E-38	1.76609E-36	110558	H2-Q9
05169	Epstein-Barr virus infection	11.37699461	2.79444E-38	1.76609E-36	230073	Ddx58
05169	Epstein-Barr virus infection	11.37699461	2.79444E-38	1.76609E-36	15001	H2-Oa
04064	NF-kappa B signaling pathway	18.29070671	7.1923E-37	3.72573E-35	12475	Cd14
04064	NF-kappa B signaling pathway	18.29070671	7.1923E-37	3.72573E-35	16177	Il1r1
04064	NF-kappa B signaling pathway	18.29070671	7.1923E-37	3.72573E-35	16797	Lat
04064	NF-kappa B signaling pathway	18.29070671	7.1923E-37	3.72573E-35	22029	Traf1
04064	NF-kappa B signaling pathway	18.29070671	7.1923E-37	3.72573E-35	22033	Traf5
04064	NF-kappa B signaling pathway	18.29070671	7.1923E-37	3.72573E-35	225471	Ticam2
04064	NF-kappa B signaling pathway	18.29070671	7.1923E-37	3.72573E-35	330122	Cxcl3
04064	NF-kappa B signaling pathway	18.29070671	7.1923E-37	3.72573E-35	17087	Ly96
04064	NF-kappa B signaling pathway	18.29070671	7.1923E-37	3.72573E-35	230073	Ddx58
04064	NF-kappa B signaling pathway	18.29070671	7.1923E-37	3.72573E-35	18033	Nfkb1
04064	NF-kappa B signaling pathway	18.29070671	7.1923E-37	3.72573E-35	21939	Cd40
04064	NF-kappa B signaling pathway	18.29070671	7.1923E-37	3.72573E-35	240354	Malt1
04064	NF-kappa B signaling pathway	18.29070671	7.1923E-37	3.72573E-35	11796	Birc3
04064	NF-kappa B signaling pathway	18.29070671	7.1923E-37	3.72573E-35	18035	Nfkbia
04064	NF-kappa B signaling pathway	18.29070671	7.1923E-37	3.72573E-35	22030	Traf2

04064	NF-kappa B signaling pathway	18.29070671	7.1923E-37	3.72573E-35	19225	Ptgs2
04064	NF-kappa B signaling pathway	18.29070671	7.1923E-37	3.72573E-35	20303	Ccl4
04064	NF-kappa B signaling pathway	18.29070671	7.1923E-37	3.72573E-35	16176	Il1b
04064	NF-kappa B signaling pathway	18.29070671	7.1923E-37	3.72573E-35	21926	Tnf
04064	NF-kappa B signaling pathway	18.29070671	7.1923E-37	3.72573E-35	50930	Tnfsf14
04064	NF-kappa B signaling pathway	18.29070671	7.1923E-37	3.72573E-35	15894	Icam1
04064	NF-kappa B signaling pathway	18.29070671	7.1923E-37	3.72573E-35	16992	Lta
04064	NF-kappa B signaling pathway	18.29070671	7.1923E-37	3.72573E-35	12047	Bcl2a1d
04064	NF-kappa B signaling pathway	18.29070671	7.1923E-37	3.72573E-35	20963	Syk
04064	NF-kappa B signaling pathway	18.29070671	7.1923E-37	3.72573E-35	17096	Lyn
04064	NF-kappa B signaling pathway	18.29070671	7.1923E-37	3.72573E-35	21929	Tnfaip3
04064	NF-kappa B signaling pathway	18.29070671	7.1923E-37	3.72573E-35	13197	Gadd45a
04064	NF-kappa B signaling pathway	18.29070671	7.1923E-37	3.72573E-35	17873	Gadd45b
04064	NF-kappa B signaling pathway	18.29070671	7.1923E-37	3.72573E-35	12044	Bcl2a1a
04064	NF-kappa B signaling pathway	18.29070671	7.1923E-37	3.72573E-35	12045	Bcl2a1b
04064	NF-kappa B signaling pathway	18.29070671	7.1923E-37	3.72573E-35	22329	Vcam1
04064	NF-kappa B signaling pathway	18.29070671	7.1923E-37	3.72573E-35	18792	Plau
04064	NF-kappa B signaling pathway	18.29070671	7.1923E-37	3.72573E-35	14825	Cxcl1
04064	NF-kappa B signaling pathway	18.29070671	7.1923E-37	3.72573E-35	20310	Cxcl2
04064	NF-kappa B signaling pathway	18.29070671	7.1923E-37	3.72573E-35	12046	Bcl2a1c
04064	NF-kappa B signaling pathway	18.29070671	7.1923E-37	3.72573E-35	19698	Relb
04064	NF-kappa B signaling pathway	18.29070671	7.1923E-37	3.72573E-35	12633	Cflar
04064	NF-kappa B signaling pathway	18.29070671	7.1923E-37	3.72573E-35	18034	Nfkb2
04621	NOD-like receptor signaling pathway	11.73680295	8.2532E-37	3.72573E-35	54123	Irf7
04621	NOD-like receptor signaling pathway	11.73680295	8.2532E-37	3.72573E-35	18798	Plcb4
04621	NOD-like receptor signaling pathway	11.73680295	8.2532E-37	3.72573E-35	28240	Trpm2
04621	NOD-like receptor signaling pathway	11.73680295	8.2532E-37	3.72573E-35	18033	Nfkb1
04621	NOD-like receptor signaling pathway	11.73680295	8.2532E-37	3.72573E-35	22166	Txn1
04621	NOD-like receptor signaling pathway	11.73680295	8.2532E-37	3.72573E-35	18035	Nfkbia
04621	NOD-like receptor signaling pathway	11.73680295	8.2532E-37	3.72573E-35	18036	Nfkbib
04621	NOD-like receptor signaling pathway	11.73680295	8.2532E-37	3.72573E-35	11796	Birc3
04621	NOD-like receptor signaling pathway	11.73680295	8.2532E-37	3.72573E-35	15519	Hsp90aa1
04621	NOD-like receptor signaling pathway	11.73680295	8.2532E-37	3.72573E-35	21353	Tank
04621	NOD-like receptor signaling pathway	11.73680295	8.2532E-37	3.72573E-35	17948	Naip2
04621	NOD-like receptor signaling pathway	11.73680295	8.2532E-37	3.72573E-35	14469	Gbp2
04621	NOD-like receptor signaling pathway	11.73680295	8.2532E-37	3.72573E-35	229898	Gbp5
04621	NOD-like receptor signaling pathway	11.73680295	8.2532E-37	3.72573E-35	229900	Gbp7
04621	NOD-like receptor signaling pathway	11.73680295	8.2532E-37	3.72573E-35	55932	Gbp3
04621	NOD-like receptor signaling pathway	11.73680295	8.2532E-37	3.72573E-35	56480	Tbk1
04621	NOD-like receptor signaling pathway	11.73680295	8.2532E-37	3.72573E-35	56489	Ikbke
04621	NOD-like receptor signaling pathway	11.73680295	8.2532E-37	3.72573E-35	20310	Cxcl2
04621	NOD-like receptor signaling pathway	11.73680295	8.2532E-37	3.72573E-35	330122	Cxcl3

04621	NOD-like receptor signaling pathway	11.73680295	8.2532E-37	3.72573E-35	20846	Stat1
04621	NOD-like receptor signaling pathway	11.73680295	8.2532E-37	3.72573E-35	20847	Stat2
04621	NOD-like receptor signaling pathway	11.73680295	8.2532E-37	3.72573E-35	12363	Casp4
04621	NOD-like receptor signaling pathway	11.73680295	8.2532E-37	3.72573E-35	26415	Mapk13
04621	NOD-like receptor signaling pathway	11.73680295	8.2532E-37	3.72573E-35	54483	Mefv
04621	NOD-like receptor signaling pathway	11.73680295	8.2532E-37	3.72573E-35	56532	Ripk3
04621	NOD-like receptor signaling pathway	11.73680295	8.2532E-37	3.72573E-35	23960	Oas1g
04621	NOD-like receptor signaling pathway	11.73680295	8.2532E-37	3.72573E-35	24014	Rnasel
04621	NOD-like receptor signaling pathway	11.73680295	8.2532E-37	3.72573E-35	16193	Il6
04621	NOD-like receptor signaling pathway	11.73680295	8.2532E-37	3.72573E-35	21926	Tnf
04621	NOD-like receptor signaling pathway	11.73680295	8.2532E-37	3.72573E-35	14825	Cxcl1
04621	NOD-like receptor signaling pathway	11.73680295	8.2532E-37	3.72573E-35	21929	Tnfaip3
04621	NOD-like receptor signaling pathway	11.73680295	8.2532E-37	3.72573E-35	12362	Casp1
04621	NOD-like receptor signaling pathway	11.73680295	8.2532E-37	3.72573E-35	20293	Ccl12
04621	NOD-like receptor signaling pathway	11.73680295	8.2532E-37	3.72573E-35	15951	Ifi204
04621	NOD-like receptor signaling pathway	11.73680295	8.2532E-37	3.72573E-35	20296	Ccl2
04621	NOD-like receptor signaling pathway	11.73680295	8.2532E-37	3.72573E-35	22030	Traf2
04621	NOD-like receptor signaling pathway	11.73680295	8.2532E-37	3.72573E-35	22033	Traf5
04621	NOD-like receptor signaling pathway	11.73680295	8.2532E-37	3.72573E-35	14468	Gbp2b
04621	NOD-like receptor signaling pathway	11.73680295	8.2532E-37	3.72573E-35	69538	Antxr1
04621	NOD-like receptor signaling pathway	11.73680295	8.2532E-37	3.72573E-35	20304	Ccl5
04621	NOD-like receptor signaling pathway	11.73680295	8.2532E-37	3.72573E-35	12796	Camp
04621	NOD-like receptor signaling pathway	11.73680295	8.2532E-37	3.72573E-35	107607	Nod1
04621	NOD-like receptor signaling pathway	11.73680295	8.2532E-37	3.72573E-35	216799	Nlrp3
04621	NOD-like receptor signaling pathway	11.73680295	8.2532E-37	3.72573E-35	257632	Nod2
04621	NOD-like receptor signaling pathway	11.73680295	8.2532E-37	3.72573E-35	13058	Cybb
04621	NOD-like receptor signaling pathway	11.73680295	8.2532E-37	3.72573E-35	16173	Il18
04621	NOD-like receptor signaling pathway	11.73680295	8.2532E-37	3.72573E-35	16176	Il1b
04621	NOD-like receptor signaling pathway	11.73680295	8.2532E-37	3.72573E-35	59027	Nampt
04621	NOD-like receptor signaling pathway	11.73680295	8.2532E-37	3.72573E-35	192656	Ripk2
04010	MAPK signaling pathway	9.110972328	8.74058E-34	3.45253E-32	13649	Egfr
04010	MAPK signaling pathway	9.110972328	8.74058E-34	3.45253E-32	22341	Vegfc
04010	MAPK signaling pathway	9.110972328	8.74058E-34	3.45253E-32	18654	Pgf
04010	MAPK signaling pathway	9.110972328	8.74058E-34	3.45253E-32	22339	Vegfa
04010	MAPK signaling pathway	9.110972328	8.74058E-34	3.45253E-32	14254	Flt1
04010	MAPK signaling pathway	9.110972328	8.74058E-34	3.45253E-32	13163	Daxx
04010	MAPK signaling pathway	9.110972328	8.74058E-34	3.45253E-32	22030	Traf2
04010	MAPK signaling pathway	9.110972328	8.74058E-34	3.45253E-32	19698	Relb
04010	MAPK signaling pathway	9.110972328	8.74058E-34	3.45253E-32	17873	Gadd45b
04010	MAPK signaling pathway	9.110972328	8.74058E-34	3.45253E-32	18049	Ngf
04010	MAPK signaling pathway	9.110972328	8.74058E-34	3.45253E-32	18034	Nfkb2
04010	MAPK signaling pathway	9.110972328	8.74058E-34	3.45253E-32	19419	Rasgrp1

04010	MAPK signaling pathway	9.110972328	8.74058E-34	3.45253E-32	215449	Rap1b
04010	MAPK signaling pathway	9.110972328	8.74058E-34	3.45253E-32	76089	Rapgef2
04010	MAPK signaling pathway	9.110972328	8.74058E-34	3.45253E-32	114713	Rasa2
04010	MAPK signaling pathway	9.110972328	8.74058E-34	3.45253E-32	11911	Atf4
04010	MAPK signaling pathway	9.110972328	8.74058E-34	3.45253E-32	12475	Cd14
04010	MAPK signaling pathway	9.110972328	8.74058E-34	3.45253E-32	14102	Fas
04010	MAPK signaling pathway	9.110972328	8.74058E-34	3.45253E-32	14164	Fgf1
04010	MAPK signaling pathway	9.110972328	8.74058E-34	3.45253E-32	26410	Map3k8
04010	MAPK signaling pathway	9.110972328	8.74058E-34	3.45253E-32	26415	Mapk13
04010	MAPK signaling pathway	9.110972328	8.74058E-34	3.45253E-32	26399	Map2k6
04010	MAPK signaling pathway	9.110972328	8.74058E-34	3.45253E-32	13197	Gadd45a
04010	MAPK signaling pathway	9.110972328	8.74058E-34	3.45253E-32	17295	Met
04010	MAPK signaling pathway	9.110972328	8.74058E-34	3.45253E-32	17869	Myc
04010	MAPK signaling pathway	9.110972328	8.74058E-34	3.45253E-32	14701	Gng12
04010	MAPK signaling pathway	9.110972328	8.74058E-34	3.45253E-32	20112	Rps6ka2
04010	MAPK signaling pathway	9.110972328	8.74058E-34	3.45253E-32	16175	Il1a
04010	MAPK signaling pathway	9.110972328	8.74058E-34	3.45253E-32	16176	Il1b
04010	MAPK signaling pathway	9.110972328	8.74058E-34	3.45253E-32	21926	Tnf
04010	MAPK signaling pathway	9.110972328	8.74058E-34	3.45253E-32	19057	Ppp3cc
04010	MAPK signaling pathway	9.110972328	8.74058E-34	3.45253E-32	286940	Flnb
04010	MAPK signaling pathway	9.110972328	8.74058E-34	3.45253E-32	11600	Angpt1
04010	MAPK signaling pathway	9.110972328	8.74058E-34	3.45253E-32	54378	Cacng6
04010	MAPK signaling pathway	9.110972328	8.74058E-34	3.45253E-32	81905	Cacng8
04010	MAPK signaling pathway	9.110972328	8.74058E-34	3.45253E-32	16177	Il1r1
04010	MAPK signaling pathway	9.110972328	8.74058E-34	3.45253E-32	13874	Ereg
04010	MAPK signaling pathway	9.110972328	8.74058E-34	3.45253E-32	70686	Dusp16
04010	MAPK signaling pathway	9.110972328	8.74058E-34	3.45253E-32	18783	Pla2g4a
04010	MAPK signaling pathway	9.110972328	8.74058E-34	3.45253E-32	18033	Nfkb1
04010	MAPK signaling pathway	9.110972328	8.74058E-34	3.45253E-32	13637	Efna2
04010	MAPK signaling pathway	9.110972328	8.74058E-34	3.45253E-32	13640	Efna5
04010	MAPK signaling pathway	9.110972328	8.74058E-34	3.45253E-32	11839	Areg
04010	MAPK signaling pathway	9.110972328	8.74058E-34	3.45253E-32	12977	Csf1
04010	MAPK signaling pathway	9.110972328	8.74058E-34	3.45253E-32	17164	Mapkapk2
04010	MAPK signaling pathway	9.110972328	8.74058E-34	3.45253E-32	13537	Dusp2
04010	MAPK signaling pathway	9.110972328	8.74058E-34	3.45253E-32	15370	Nr4a1
04010	MAPK signaling pathway	9.110972328	8.74058E-34	3.45253E-32	56613	Rps6ka4
04010	MAPK signaling pathway	9.110972328	8.74058E-34	3.45253E-32	19417	Rasgrf1
04010	MAPK signaling pathway	9.110972328	8.74058E-34	3.45253E-32	18218	Dusp8
04010	MAPK signaling pathway	9.110972328	8.74058E-34	3.45253E-32	67603	Dusp6
04010	MAPK signaling pathway	9.110972328	8.74058E-34	3.45253E-32	319520	Dusp4
04010	MAPK signaling pathway	9.110972328	8.74058E-34	3.45253E-32	240672	Dusp5
05168	Herpes simplex virus 1 infection	6.952023954	7.083E-33	2.48692E-31	20847	Stat2

05168	Herpes simplex virus 1 infection	6.952023954	7.083E-33	2.48692E-31	243308	A430033K04Rik
05168	Herpes simplex virus 1 infection	6.952023954	7.083E-33	2.48692E-31	16452	Jak2
05168	Herpes simplex virus 1 infection	6.952023954	7.083E-33	2.48692E-31	13063	Cycs
05168	Herpes simplex virus 1 infection	6.952023954	7.083E-33	2.48692E-31	12702	Socs3
05168	Herpes simplex virus 1 infection	6.952023954	7.083E-33	2.48692E-31	72230	Zfp558
05168	Herpes simplex virus 1 infection	6.952023954	7.083E-33	2.48692E-31	74400	Zfp819
05168	Herpes simplex virus 1 infection	6.952023954	7.083E-33	2.48692E-31	20846	Stat1
05168	Herpes simplex virus 1 infection	6.952023954	7.083E-33	2.48692E-31	23960	Oas1g
05168	Herpes simplex virus 1 infection	6.952023954	7.083E-33	2.48692E-31	24014	Rnasel
05168	Herpes simplex virus 1 infection	6.952023954	7.083E-33	2.48692E-31	15018	H2-Q7
05168	Herpes simplex virus 1 infection	6.952023954	7.083E-33	2.48692E-31	24135	Zfp68
05168	Herpes simplex virus 1 infection	6.952023954	7.083E-33	2.48692E-31	69550	Bst2
05168	Herpes simplex virus 1 infection	6.952023954	7.083E-33	2.48692E-31	20304	Ccl5
05168	Herpes simplex virus 1 infection	6.952023954	7.083E-33	2.48692E-31	18987	Pou2f2
05168	Herpes simplex virus 1 infection	6.952023954	7.083E-33	2.48692E-31	15978	Ifng
05168	Herpes simplex virus 1 infection	6.952023954	7.083E-33	2.48692E-31	214763	Cgas
05168	Herpes simplex virus 1 infection	6.952023954	7.083E-33	2.48692E-31	110558	H2-Q9
05168	Herpes simplex virus 1 infection	6.952023954	7.083E-33	2.48692E-31	14990	H2-M2
05168	Herpes simplex virus 1 infection	6.952023954	7.083E-33	2.48692E-31	15024	H2-T10
05168	Herpes simplex virus 1 infection	6.952023954	7.083E-33	2.48692E-31	243905	Zfp568
05168	Herpes simplex virus 1 infection	6.952023954	7.083E-33	2.48692E-31	330301	Zfp786
05168	Herpes simplex virus 1 infection	6.952023954	7.083E-33	2.48692E-31	20293	Ccl12
05168	Herpes simplex virus 1 infection	6.952023954	7.083E-33	2.48692E-31	21926	Tnf
05168	Herpes simplex virus 1 infection	6.952023954	7.083E-33	2.48692E-31	56489	Ikbke
05168	Herpes simplex virus 1 infection	6.952023954	7.083E-33	2.48692E-31	56480	Tbk1
05168	Herpes simplex virus 1 infection	6.952023954	7.083E-33	2.48692E-31	71586	Ifih1
05168	Herpes simplex virus 1 infection	6.952023954	7.083E-33	2.48692E-31	20296	Ccl2
05168	Herpes simplex virus 1 infection	6.952023954	7.083E-33	2.48692E-31	11796	Birc3
05168	Herpes simplex virus 1 infection	6.952023954	7.083E-33	2.48692E-31	210105	Zfp719
05168	Herpes simplex virus 1 infection	6.952023954	7.083E-33	2.48692E-31	240063	Zfp811
05168	Herpes simplex virus 1 infection	6.952023954	7.083E-33	2.48692E-31	16160	Il12b
05168	Herpes simplex virus 1 infection	6.952023954	7.083E-33	2.48692E-31	19106	Eif2ak2
05168	Herpes simplex virus 1 infection	6.952023954	7.083E-33	2.48692E-31	50930	Tnfsf14
05168	Herpes simplex virus 1 infection	6.952023954	7.083E-33	2.48692E-31	170741	Pilrb1
05168	Herpes simplex virus 1 infection	6.952023954	7.083E-33	2.48692E-31	15039	H2-T22
05168	Herpes simplex virus 1 infection	6.952023954	7.083E-33	2.48692E-31	12317	Calr
05168	Herpes simplex virus 1 infection	6.952023954	7.083E-33	2.48692E-31	15001	H2-Oa
05168	Herpes simplex virus 1 infection	6.952023954	7.083E-33	2.48692E-31	14102	Fas
05168	Herpes simplex virus 1 infection	6.952023954	7.083E-33	2.48692E-31	108067	Eif2b3
05168	Herpes simplex virus 1 infection	6.952023954	7.083E-33	2.48692E-31	230073	Ddx58
05168	Herpes simplex virus 1 infection	6.952023954	7.083E-33	2.48692E-31	210104	Zfp658
05168	Herpes simplex virus 1 infection	6.952023954	7.083E-33	2.48692E-31	18636	Cfp

05168	Herpes simplex virus 1 infection	6.952023954	7.083E-33	2.48692E-31	24088	Tlr2
05168	Herpes simplex virus 1 infection	6.952023954	7.083E-33	2.48692E-31	13163	Daxx
05168	Herpes simplex virus 1 infection	6.952023954	7.083E-33	2.48692E-31	16992	Lta
05168	Herpes simplex virus 1 infection	6.952023954	7.083E-33	2.48692E-31	16159	Il12a
05168	Herpes simplex virus 1 infection	6.952023954	7.083E-33	2.48692E-31	16176	Il1b
05168	Herpes simplex virus 1 infection	6.952023954	7.083E-33	2.48692E-31	16193	Il6
05168	Herpes simplex virus 1 infection	6.952023954	7.083E-33	2.48692E-31	231805	Pilra
05168	Herpes simplex virus 1 infection	6.952023954	7.083E-33	2.48692E-31	545812	Pilrb2
05168	Herpes simplex virus 1 infection	6.952023954	7.083E-33	2.48692E-31	12122	Bid
05168	Herpes simplex virus 1 infection	6.952023954	7.083E-33	2.48692E-31	18708	Pik3r1
05168	Herpes simplex virus 1 infection	6.952023954	7.083E-33	2.48692E-31	16402	Itga5
05168	Herpes simplex virus 1 infection	6.952023954	7.083E-33	2.48692E-31	20779	Src
05168	Herpes simplex virus 1 infection	6.952023954	7.083E-33	2.48692E-31	20963	Syk
05168	Herpes simplex virus 1 infection	6.952023954	7.083E-33	2.48692E-31	12266	C3
05168	Herpes simplex virus 1 infection	6.952023954	7.083E-33	2.48692E-31	54123	Irf7
05168	Herpes simplex virus 1 infection	6.952023954	7.083E-33	2.48692E-31	18035	Nfkbia
05168	Herpes simplex virus 1 infection	6.952023954	7.083E-33	2.48692E-31	22030	Traf2
05168	Herpes simplex virus 1 infection	6.952023954	7.083E-33	2.48692E-31	56717	Mtor
05168	Herpes simplex virus 1 infection	6.952023954	7.083E-33	2.48692E-31	18033	Nfkb1
05168	Herpes simplex virus 1 infection	6.952023954	7.083E-33	2.48692E-31	22033	Traf5
04151	PI3K-Akt signaling pathway	7.88369414	3.29921E-32	1.04255E-30	13649	Egfr
04151	PI3K-Akt signaling pathway	7.88369414	3.29921E-32	1.04255E-30	13684	Eif4e
04151	PI3K-Akt signaling pathway	7.88369414	3.29921E-32	1.04255E-30	18708	Pik3r1
04151	PI3K-Akt signaling pathway	7.88369414	3.29921E-32	1.04255E-30	14745	Lpar1
04151	PI3K-Akt signaling pathway	7.88369414	3.29921E-32	1.04255E-30	320207	Pik3r5
04151	PI3K-Akt signaling pathway	7.88369414	3.29921E-32	1.04255E-30	56717	Mtor
04151	PI3K-Akt signaling pathway	7.88369414	3.29921E-32	1.04255E-30	14062	F2r
04151	PI3K-Akt signaling pathway	7.88369414	3.29921E-32	1.04255E-30	14936	Gys1
04151	PI3K-Akt signaling pathway	7.88369414	3.29921E-32	1.04255E-30	16782	Lamc2
04151	PI3K-Akt signaling pathway	7.88369414	3.29921E-32	1.04255E-30	12827	Col4a2
04151	PI3K-Akt signaling pathway	7.88369414	3.29921E-32	1.04255E-30	21825	Thbs1
04151	PI3K-Akt signaling pathway	7.88369414	3.29921E-32	1.04255E-30	21923	Tnc
04151	PI3K-Akt signaling pathway	7.88369414	3.29921E-32	1.04255E-30	329278	Tnn
04151	PI3K-Akt signaling pathway	7.88369414	3.29921E-32	1.04255E-30	24088	Tlr2
04151	PI3K-Akt signaling pathway	7.88369414	3.29921E-32	1.04255E-30	14254	Flt1
04151	PI3K-Akt signaling pathway	7.88369414	3.29921E-32	1.04255E-30	17295	Met
04151	PI3K-Akt signaling pathway	7.88369414	3.29921E-32	1.04255E-30	16190	Il4ra
04151	PI3K-Akt signaling pathway	7.88369414	3.29921E-32	1.04255E-30	104099	Itga9
04151	PI3K-Akt signaling pathway	7.88369414	3.29921E-32	1.04255E-30	109700	Itga1
04151	PI3K-Akt signaling pathway	7.88369414	3.29921E-32	1.04255E-30	16402	Itga5
04151	PI3K-Akt signaling pathway	7.88369414	3.29921E-32	1.04255E-30	16193	Il6
04151	PI3K-Akt signaling pathway	7.88369414	3.29921E-32	1.04255E-30	319480	Itga11

04151	PI3K-Akt signaling pathway	7.88369414	3.29921E-32	1.04255E-30	320910	Itgb8
04151	PI3K-Akt signaling pathway	7.88369414	3.29921E-32	1.04255E-30	11600	Angpt1
04151	PI3K-Akt signaling pathway	7.88369414	3.29921E-32	1.04255E-30	11839	Areg
04151	PI3K-Akt signaling pathway	7.88369414	3.29921E-32	1.04255E-30	12575	Cdkn1a
04151	PI3K-Akt signaling pathway	7.88369414	3.29921E-32	1.04255E-30	30955	Pik3cg
04151	PI3K-Akt signaling pathway	7.88369414	3.29921E-32	1.04255E-30	13637	Efna2
04151	PI3K-Akt signaling pathway	7.88369414	3.29921E-32	1.04255E-30	13640	Efna5
04151	PI3K-Akt signaling pathway	7.88369414	3.29921E-32	1.04255E-30	20963	Syk
04151	PI3K-Akt signaling pathway	7.88369414	3.29921E-32	1.04255E-30	16452	Jak2
04151	PI3K-Akt signaling pathway	7.88369414	3.29921E-32	1.04255E-30	12985	Csf3
04151	PI3K-Akt signaling pathway	7.88369414	3.29921E-32	1.04255E-30	17869	Myc
04151	PI3K-Akt signaling pathway	7.88369414	3.29921E-32	1.04255E-30	22341	Vegfc
04151	PI3K-Akt signaling pathway	7.88369414	3.29921E-32	1.04255E-30	18413	Osm
04151	PI3K-Akt signaling pathway	7.88369414	3.29921E-32	1.04255E-30	18033	Nfkb1
04151	PI3K-Akt signaling pathway	7.88369414	3.29921E-32	1.04255E-30	22027	Hsp90b1
04151	PI3K-Akt signaling pathway	7.88369414	3.29921E-32	1.04255E-30	105787	Prkaa1
04151	PI3K-Akt signaling pathway	7.88369414	3.29921E-32	1.04255E-30	108079	Prkaa2
04151	PI3K-Akt signaling pathway	7.88369414	3.29921E-32	1.04255E-30	74747	Ddit4
04151	PI3K-Akt signaling pathway	7.88369414	3.29921E-32	1.04255E-30	14164	Fgf1
04151	PI3K-Akt signaling pathway	7.88369414	3.29921E-32	1.04255E-30	83490	Pik3ap1
04151	PI3K-Akt signaling pathway	7.88369414	3.29921E-32	1.04255E-30	12986	Csf3r
04151	PI3K-Akt signaling pathway	7.88369414	3.29921E-32	1.04255E-30	14701	Gng12
04151	PI3K-Akt signaling pathway	7.88369414	3.29921E-32	1.04255E-30	15519	Hsp90aa1
04151	PI3K-Akt signaling pathway	7.88369414	3.29921E-32	1.04255E-30	18049	Ngf
04151	PI3K-Akt signaling pathway	7.88369414	3.29921E-32	1.04255E-30	22339	Vegfa
04151	PI3K-Akt signaling pathway	7.88369414	3.29921E-32	1.04255E-30	19053	Ppp2cb
04151	PI3K-Akt signaling pathway	7.88369414	3.29921E-32	1.04255E-30	12977	Csf1
04151	PI3K-Akt signaling pathway	7.88369414	3.29921E-32	1.04255E-30	11911	Atf4
04151	PI3K-Akt signaling pathway	7.88369414	3.29921E-32	1.04255E-30	231991	Creb5
04151	PI3K-Akt signaling pathway	7.88369414	3.29921E-32	1.04255E-30	17863	Myb
04151	PI3K-Akt signaling pathway	7.88369414	3.29921E-32	1.04255E-30	15370	Nr4a1
04151	PI3K-Akt signaling pathway	7.88369414	3.29921E-32	1.04255E-30	13874	Ereg
04151	PI3K-Akt signaling pathway	7.88369414	3.29921E-32	1.04255E-30	12444	Ccnd2
04151	PI3K-Akt signaling pathway	7.88369414	3.29921E-32	1.04255E-30	18654	Pgf
04657	IL-17 signaling pathway	18.32773243	3.70837E-32	1.06531E-30	22027	Hsp90b1
04657	IL-17 signaling pathway	18.32773243	3.70837E-32	1.06531E-30	22033	Traf5
04657	IL-17 signaling pathway	18.32773243	3.70837E-32	1.06531E-30	26415	Mapk13
04657	IL-17 signaling pathway	18.32773243	3.70837E-32	1.06531E-30	12608	Cebpb
04657	IL-17 signaling pathway	18.32773243	3.70837E-32	1.06531E-30	15978	Ifng
04657	IL-17 signaling pathway	18.32773243	3.70837E-32	1.06531E-30	14825	Cxcl1
04657	IL-17 signaling pathway	18.32773243	3.70837E-32	1.06531E-30	14283	Fosl1
04657	IL-17 signaling pathway	18.32773243	3.70837E-32	1.06531E-30	18033	Nfkb1

04657	IL-17 signaling pathway	18.32773243	3.70837E-32	1.06531E-30	17392	Mmp3
04657	IL-17 signaling pathway	18.32773243	3.70837E-32	1.06531E-30	15519	Hsp90aa1
04657	IL-17 signaling pathway	18.32773243	3.70837E-32	1.06531E-30	16176	Il1b
04657	IL-17 signaling pathway	18.32773243	3.70837E-32	1.06531E-30	20201	S100a8
04657	IL-17 signaling pathway	18.32773243	3.70837E-32	1.06531E-30	20311	Cxcl5
04657	IL-17 signaling pathway	18.32773243	3.70837E-32	1.06531E-30	56489	Ikbke
04657	IL-17 signaling pathway	18.32773243	3.70837E-32	1.06531E-30	17386	Mmp13
04657	IL-17 signaling pathway	18.32773243	3.70837E-32	1.06531E-30	56480	Tbk1
04657	IL-17 signaling pathway	18.32773243	3.70837E-32	1.06531E-30	16193	Il6
04657	IL-17 signaling pathway	18.32773243	3.70837E-32	1.06531E-30	83996	Mmp1b
04657	IL-17 signaling pathway	18.32773243	3.70837E-32	1.06531E-30	16819	Lcn2
04657	IL-17 signaling pathway	18.32773243	3.70837E-32	1.06531E-30	18035	Nfkbia
04657	IL-17 signaling pathway	18.32773243	3.70837E-32	1.06531E-30	21926	Tnf
04657	IL-17 signaling pathway	18.32773243	3.70837E-32	1.06531E-30	330122	Cxcl3
04657	IL-17 signaling pathway	18.32773243	3.70837E-32	1.06531E-30	15945	Cxcl10
04657	IL-17 signaling pathway	18.32773243	3.70837E-32	1.06531E-30	12985	Csf3
04657	IL-17 signaling pathway	18.32773243	3.70837E-32	1.06531E-30	19225	Ptgs2
04657	IL-17 signaling pathway	18.32773243	3.70837E-32	1.06531E-30	20306	Ccl7
04657	IL-17 signaling pathway	18.32773243	3.70837E-32	1.06531E-30	22030	Traf2
04657	IL-17 signaling pathway	18.32773243	3.70837E-32	1.06531E-30	20293	Ccl12
04657	IL-17 signaling pathway	18.32773243	3.70837E-32	1.06531E-30	20296	Ccl2
04657	IL-17 signaling pathway	18.32773243	3.70837E-32	1.06531E-30	21929	Tnfaip3
04657	IL-17 signaling pathway	18.32773243	3.70837E-32	1.06531E-30	12981	Csf2
04657	IL-17 signaling pathway	18.32773243	3.70837E-32	1.06531E-30	17395	Mmp9
04657	IL-17 signaling pathway	18.32773243	3.70837E-32	1.06531E-30	20310	Cxcl2
05167	Kaposi sarcoma-associated herpesvirus infection	10.33264484	5.83147E-32	1.53562E-30	19225	Ptgs2
05167	Kaposi sarcoma-associated herpesvirus infection	10.33264484	5.83147E-32	1.53562E-30	17096	Lyn
05167	Kaposi sarcoma-associated herpesvirus infection	10.33264484	5.83147E-32	1.53562E-30	330122	Cxcl3
05167	Kaposi sarcoma-associated herpesvirus infection	10.33264484	5.83147E-32	1.53562E-30	22339	Vegfa
05167	Kaposi sarcoma-associated herpesvirus infection	10.33264484	5.83147E-32	1.53562E-30	16193	Il6
05167	Kaposi sarcoma-associated herpesvirus infection	10.33264484	5.83147E-32	1.53562E-30	12524	Cd86
05167	Kaposi sarcoma-associated herpesvirus infection	10.33264484	5.83147E-32	1.53562E-30	20963	Syk
05167	Kaposi sarcoma-associated herpesvirus infection	10.33264484	5.83147E-32	1.53562E-30	20310	Cxcl2
05167	Kaposi sarcoma-associated herpesvirus infection	10.33264484	5.83147E-32	1.53562E-30	12981	Csf2
05167	Kaposi sarcoma-associated herpesvirus infection	10.33264484	5.83147E-32	1.53562E-30	22695	Zfp36
05167	Kaposi sarcoma-associated herpesvirus infection	10.33264484	5.83147E-32	1.53562E-30	56717	Mtor
05167	Kaposi sarcoma-associated herpesvirus infection	10.33264484	5.83147E-32	1.53562E-30	56489	Ikbke
05167	Kaposi sarcoma-associated herpesvirus infection	10.33264484	5.83147E-32	1.53562E-30	320207	Pik3r5
05167	Kaposi sarcoma-associated herpesvirus infection	10.33264484	5.83147E-32	1.53562E-30	15162	Hck
05167	Kaposi sarcoma-associated herpesvirus infection	10.33264484	5.83147E-32	1.53562E-30	20779	Src
05167	Kaposi sarcoma-associated herpesvirus infection	10.33264484	5.83147E-32	1.53562E-30	18708	Pik3r1
05167	Kaposi sarcoma-associated herpesvirus infection	10.33264484	5.83147E-32	1.53562E-30	17869	Myc

05167	Kaposi sarcoma-associated herpesvirus infection	10.33264484	5.83147E-32	1.53562E-30	15894	Icam1
05167	Kaposi sarcoma-associated herpesvirus infection	10.33264484	5.83147E-32	1.53562E-30	12122	Bid
05167	Kaposi sarcoma-associated herpesvirus infection	10.33264484	5.83147E-32	1.53562E-30	14825	Cxcl1
05167	Kaposi sarcoma-associated herpesvirus infection	10.33264484	5.83147E-32	1.53562E-30	26399	Map2k6
05167	Kaposi sarcoma-associated herpesvirus infection	10.33264484	5.83147E-32	1.53562E-30	26415	Mapk13
05167	Kaposi sarcoma-associated herpesvirus infection	10.33264484	5.83147E-32	1.53562E-30	54123	Irf7
05167	Kaposi sarcoma-associated herpesvirus infection	10.33264484	5.83147E-32	1.53562E-30	30955	Pik3cg
05167	Kaposi sarcoma-associated herpesvirus infection	10.33264484	5.83147E-32	1.53562E-30	14701	Gng12
05167	Kaposi sarcoma-associated herpesvirus infection	10.33264484	5.83147E-32	1.53562E-30	15251	Hif1a
05167	Kaposi sarcoma-associated herpesvirus infection	10.33264484	5.83147E-32	1.53562E-30	19057	Ppp3cc
05167	Kaposi sarcoma-associated herpesvirus infection	10.33264484	5.83147E-32	1.53562E-30	12768	Ccr1
05167	Kaposi sarcoma-associated herpesvirus infection	10.33264484	5.83147E-32	1.53562E-30	56480	Tbk1
05167	Kaposi sarcoma-associated herpesvirus infection	10.33264484	5.83147E-32	1.53562E-30	18033	Nfkb1
05167	Kaposi sarcoma-associated herpesvirus infection	10.33264484	5.83147E-32	1.53562E-30	22030	Traf2
05167	Kaposi sarcoma-associated herpesvirus infection	10.33264484	5.83147E-32	1.53562E-30	15018	H2-Q7
05167	Kaposi sarcoma-associated herpesvirus infection	10.33264484	5.83147E-32	1.53562E-30	18035	Nfkbia
05167	Kaposi sarcoma-associated herpesvirus infection	10.33264484	5.83147E-32	1.53562E-30	17164	Mapkapk2
05167	Kaposi sarcoma-associated herpesvirus infection	10.33264484	5.83147E-32	1.53562E-30	20847	Stat2
05167	Kaposi sarcoma-associated herpesvirus infection	10.33264484	5.83147E-32	1.53562E-30	19106	Eif2ak2
05167	Kaposi sarcoma-associated herpesvirus infection	10.33264484	5.83147E-32	1.53562E-30	20846	Stat1
05167	Kaposi sarcoma-associated herpesvirus infection	10.33264484	5.83147E-32	1.53562E-30	16452	Jak2
05167	Kaposi sarcoma-associated herpesvirus infection	10.33264484	5.83147E-32	1.53562E-30	12575	Cdkn1a
05167	Kaposi sarcoma-associated herpesvirus infection	10.33264484	5.83147E-32	1.53562E-30	14102	Fas
05167	Kaposi sarcoma-associated herpesvirus infection	10.33264484	5.83147E-32	1.53562E-30	15024	H2-T10
05167	Kaposi sarcoma-associated herpesvirus infection	10.33264484	5.83147E-32	1.53562E-30	13063	Cycs
05167	Kaposi sarcoma-associated herpesvirus infection	10.33264484	5.83147E-32	1.53562E-30	14990	H2-M2
05167	Kaposi sarcoma-associated herpesvirus infection	10.33264484	5.83147E-32	1.53562E-30	15039	H2-T22
05167	Kaposi sarcoma-associated herpesvirus infection	10.33264484	5.83147E-32	1.53562E-30	110558	H2-Q9
05167	Kaposi sarcoma-associated herpesvirus infection	10.33264484	5.83147E-32	1.53562E-30	12266	C3
04061	Viral protein interaction with cytokine and cytokine receptor	17.55603844	1.96184E-31	4.76877E-30	12768	Ccr1
04061	Viral protein interaction with cytokine and cytokine receptor	17.55603844	1.96184E-31	4.76877E-30	20293	Ccl12
04061	Viral protein interaction with cytokine and cytokine receptor	17.55603844	1.96184E-31	4.76877E-30	20306	Ccl7
04061	Viral protein interaction with cytokine and cytokine receptor	17.55603844	1.96184E-31	4.76877E-30	21933	Tnfrsf10b
04061	Viral protein interaction with cytokine and cytokine receptor	17.55603844	1.96184E-31	4.76877E-30	21926	Tnf
04061	Viral protein interaction with cytokine and cytokine receptor	17.55603844	1.96184E-31	4.76877E-30	22035	Tnfsf10
04061	Viral protein interaction with cytokine and cytokine receptor	17.55603844	1.96184E-31	4.76877E-30	20304	Ccl5
04061	Viral protein interaction with cytokine and cytokine receptor	17.55603844	1.96184E-31	4.76877E-30	20308	Ccl9
04061	Viral protein interaction with cytokine and cytokine receptor	17.55603844	1.96184E-31	4.76877E-30	50930	Tnfsf14
04061	Viral protein interaction with cytokine and cytokine receptor	17.55603844	1.96184E-31	4.76877E-30	20302	Ccl3
04061	Viral protein interaction with cytokine and cytokine receptor	17.55603844	1.96184E-31	4.76877E-30	20310	Cxcl2
04061	Viral protein interaction with cytokine and cytokine receptor	17.55603844	1.96184E-31	4.76877E-30	16992	Lta
04061	Viral protein interaction with cytokine and cytokine receptor	17.55603844	1.96184E-31	4.76877E-30	12977	Csf1

04061	Viral protein interaction with cytokine and cytokine receptor	17.55603844	1.96184E-31	4.76877E-30	14825	Cxcl1
04061	Viral protein interaction with cytokine and cytokine receptor	17.55603844	1.96184E-31	4.76877E-30	20296	Ccl2
04061	Viral protein interaction with cytokine and cytokine receptor	17.55603844	1.96184E-31	4.76877E-30	56221	Ccl24
04061	Viral protein interaction with cytokine and cytokine receptor	17.55603844	1.96184E-31	4.76877E-30	21938	Tnfrsf1b
04061	Viral protein interaction with cytokine and cytokine receptor	17.55603844	1.96184E-31	4.76877E-30	329244	Il19
04061	Viral protein interaction with cytokine and cytokine receptor	17.55603844	1.96184E-31	4.76877E-30	12769	Ccr9
04061	Viral protein interaction with cytokine and cytokine receptor	17.55603844	1.96184E-31	4.76877E-30	16173	Il18
04061	Viral protein interaction with cytokine and cytokine receptor	17.55603844	1.96184E-31	4.76877E-30	213208	Il20rb
04061	Viral protein interaction with cytokine and cytokine receptor	17.55603844	1.96184E-31	4.76877E-30	330122	Cxcl3
04061	Viral protein interaction with cytokine and cytokine receptor	17.55603844	1.96184E-31	4.76877E-30	20303	Ccl4
04061	Viral protein interaction with cytokine and cytokine receptor	17.55603844	1.96184E-31	4.76877E-30	57349	Ppbp
04061	Viral protein interaction with cytokine and cytokine receptor	17.55603844	1.96184E-31	4.76877E-30	20311	Cxcl5
04061	Viral protein interaction with cytokine and cytokine receptor	17.55603844	1.96184E-31	4.76877E-30	20299	Ccl22
04061	Viral protein interaction with cytokine and cytokine receptor	17.55603844	1.96184E-31	4.76877E-30	12775	Ccr7
04061	Viral protein interaction with cytokine and cytokine receptor	17.55603844	1.96184E-31	4.76877E-30	16193	Il6
04061	Viral protein interaction with cytokine and cytokine receptor	17.55603844	1.96184E-31	4.76877E-30	15945	Cxcl10
04061	Viral protein interaction with cytokine and cytokine receptor	17.55603844	1.96184E-31	4.76877E-30	23832	Xcr1
04061	Viral protein interaction with cytokine and cytokine receptor	17.55603844	1.96184E-31	4.76877E-30	17329	Cxcl9
04061	Viral protein interaction with cytokine and cytokine receptor	17.55603844	1.96184E-31	4.76877E-30	16153	Il10
04061	Viral protein interaction with cytokine and cytokine receptor	17.55603844	1.96184E-31	4.76877E-30	56744	Pf4
05418	Fluid shear stress and atherosclerosis	13.15427537	9.88892E-31	2.23207E-29	16175	Il1a
05418	Fluid shear stress and atherosclerosis	13.15427537	9.88892E-31	2.23207E-29	100042295	Gm3776
05418	Fluid shear stress and atherosclerosis	13.15427537	9.88892E-31	2.23207E-29	15368	Hmox1
05418	Fluid shear stress and atherosclerosis	13.15427537	9.88892E-31	2.23207E-29	16176	Il1b
05418	Fluid shear stress and atherosclerosis	13.15427537	9.88892E-31	2.23207E-29	11898	Ass1
05418	Fluid shear stress and atherosclerosis	13.15427537	9.88892E-31	2.23207E-29	14857	Gsta1
05418	Fluid shear stress and atherosclerosis	13.15427537	9.88892E-31	2.23207E-29	21926	Tnf
05418	Fluid shear stress and atherosclerosis	13.15427537	9.88892E-31	2.23207E-29	20293	Ccl12
05418	Fluid shear stress and atherosclerosis	13.15427537	9.88892E-31	2.23207E-29	15978	Ifng
05418	Fluid shear stress and atherosclerosis	13.15427537	9.88892E-31	2.23207E-29	14863	Gstm2
05418	Fluid shear stress and atherosclerosis	13.15427537	9.88892E-31	2.23207E-29	13614	Edn1
05418	Fluid shear stress and atherosclerosis	13.15427537	9.88892E-31	2.23207E-29	14858	Gsta2
05418	Fluid shear stress and atherosclerosis	13.15427537	9.88892E-31	2.23207E-29	14860	Gsta4
05418	Fluid shear stress and atherosclerosis	13.15427537	9.88892E-31	2.23207E-29	108079	Prkaa2
05418	Fluid shear stress and atherosclerosis	13.15427537	9.88892E-31	2.23207E-29	17390	Mmp2
05418	Fluid shear stress and atherosclerosis	13.15427537	9.88892E-31	2.23207E-29	18708	Pik3r1
05418	Fluid shear stress and atherosclerosis	13.15427537	9.88892E-31	2.23207E-29	17395	Mmp9
05418	Fluid shear stress and atherosclerosis	13.15427537	9.88892E-31	2.23207E-29	12389	Cav1
05418	Fluid shear stress and atherosclerosis	13.15427537	9.88892E-31	2.23207E-29	20296	Ccl2
05418	Fluid shear stress and atherosclerosis	13.15427537	9.88892E-31	2.23207E-29	26399	Map2k6
05418	Fluid shear stress and atherosclerosis	13.15427537	9.88892E-31	2.23207E-29	12562	Cdh5
05418	Fluid shear stress and atherosclerosis	13.15427537	9.88892E-31	2.23207E-29	105787	Prkaa1

05418	Fluid shear stress and atherosclerosis	13.15427537	9.88892E-31	2.23207E-29	22339	Vegfa
05418	Fluid shear stress and atherosclerosis	13.15427537	9.88892E-31	2.23207E-29	22166	Txn1
05418	Fluid shear stress and atherosclerosis	13.15427537	9.88892E-31	2.23207E-29	18412	Sqstm1
05418	Fluid shear stress and atherosclerosis	13.15427537	9.88892E-31	2.23207E-29	18033	Nfkb1
05418	Fluid shear stress and atherosclerosis	13.15427537	9.88892E-31	2.23207E-29	26415	Mapk13
05418	Fluid shear stress and atherosclerosis	13.15427537	9.88892E-31	2.23207E-29	22329	Vcam1
05418	Fluid shear stress and atherosclerosis	13.15427537	9.88892E-31	2.23207E-29	15894	Icam1
05418	Fluid shear stress and atherosclerosis	13.15427537	9.88892E-31	2.23207E-29	12166	Bmpr1a
05418	Fluid shear stress and atherosclerosis	13.15427537	9.88892E-31	2.23207E-29	20969	Sdc1
05418	Fluid shear stress and atherosclerosis	13.15427537	9.88892E-31	2.23207E-29	15519	Hsp90aa1
05418	Fluid shear stress and atherosclerosis	13.15427537	9.88892E-31	2.23207E-29	18104	Nqo1
05418	Fluid shear stress and atherosclerosis	13.15427537	9.88892E-31	2.23207E-29	20779	Src
05418	Fluid shear stress and atherosclerosis	13.15427537	9.88892E-31	2.23207E-29	14733	Gpc1
05418	Fluid shear stress and atherosclerosis	13.15427537	9.88892E-31	2.23207E-29	20971	Sdc4
05418	Fluid shear stress and atherosclerosis	13.15427537	9.88892E-31	2.23207E-29	16177	Il1r1
05418	Fluid shear stress and atherosclerosis	13.15427537	9.88892E-31	2.23207E-29	22027	Hsp90b1
05152	Tuberculosis	11.2311357	2.15881E-29	4.54789E-28	16175	Il1a
05152	Tuberculosis	11.2311357	2.15881E-29	4.54789E-28	83430	Il23a
05152	Tuberculosis	11.2311357	2.15881E-29	4.54789E-28	16160	Il12b
05152	Tuberculosis	11.2311357	2.15881E-29	4.54789E-28	16706	Ksr1
05152	Tuberculosis	11.2311357	2.15881E-29	4.54789E-28	16159	Il12a
05152	Tuberculosis	11.2311357	2.15881E-29	4.54789E-28	12796	Camp
05152	Tuberculosis	11.2311357	2.15881E-29	4.54789E-28	257632	Nod2
05152	Tuberculosis	11.2311357	2.15881E-29	4.54789E-28	15001	H2-Oa
05152	Tuberculosis	11.2311357	2.15881E-29	4.54789E-28	16173	Il18
05152	Tuberculosis	11.2311357	2.15881E-29	4.54789E-28	20963	Syk
05152	Tuberculosis	11.2311357	2.15881E-29	4.54789E-28	12122	Bid
05152	Tuberculosis	11.2311357	2.15881E-29	4.54789E-28	13063	Cycs
05152	Tuberculosis	11.2311357	2.15881E-29	4.54789E-28	192656	Ripk2
05152	Tuberculosis	11.2311357	2.15881E-29	4.54789E-28	16193	Il6
05152	Tuberculosis	11.2311357	2.15881E-29	4.54789E-28	240354	Malt1
05152	Tuberculosis	11.2311357	2.15881E-29	4.54789E-28	56619	Clec4e
05152	Tuberculosis	11.2311357	2.15881E-29	4.54789E-28	12795	Plk3
05152	Tuberculosis	11.2311357	2.15881E-29	4.54789E-28	24088	Tlr2
05152	Tuberculosis	11.2311357	2.15881E-29	4.54789E-28	12608	Cebpb
05152	Tuberculosis	11.2311357	2.15881E-29	4.54789E-28	12266	C3
05152	Tuberculosis	11.2311357	2.15881E-29	4.54789E-28	18033	Nfkb1
05152	Tuberculosis	11.2311357	2.15881E-29	4.54789E-28	21897	Tlr1
05152	Tuberculosis	11.2311357	2.15881E-29	4.54789E-28	21899	Tlr6
05152	Tuberculosis	11.2311357	2.15881E-29	4.54789E-28	21926	Tnf
05152	Tuberculosis	11.2311357	2.15881E-29	4.54789E-28	20779	Src
05152	Tuberculosis	11.2311357	2.15881E-29	4.54789E-28	16176	Il1b

05152	Tuberculosis	11.2311357	2.15881E-29	4.54789E-28	15978	Ifng
05152	Tuberculosis	11.2311357	2.15881E-29	4.54789E-28	16452	Jak2
05152	Tuberculosis	11.2311357	2.15881E-29	4.54789E-28	20846	Stat1
05152	Tuberculosis	11.2311357	2.15881E-29	4.54789E-28	16153	Il10
05152	Tuberculosis	11.2311357	2.15881E-29	4.54789E-28	26415	Mapk13
05152	Tuberculosis	11.2311357	2.15881E-29	4.54789E-28	22337	Vdr
05152	Tuberculosis	11.2311357	2.15881E-29	4.54789E-28	20698	Sphk1
05152	Tuberculosis	11.2311357	2.15881E-29	4.54789E-28	12475	Cd14
05152	Tuberculosis	11.2311357	2.15881E-29	4.54789E-28	108058	Camk2d
05152	Tuberculosis	11.2311357	2.15881E-29	4.54789E-28	19057	Ppp3cc
05152	Tuberculosis	11.2311357	2.15881E-29	4.54789E-28	14130	Fcgr2b
05152	Tuberculosis	11.2311357	2.15881E-29	4.54789E-28	108960	Irak2
05152	Tuberculosis	11.2311357	2.15881E-29	4.54789E-28	216238	Eea1
05152	Tuberculosis	11.2311357	2.15881E-29	4.54789E-28	18126	Nos2
05164	Influenza A	11.39343535	6.23627E-29	1.23166E-27	16160	Il12b
05164	Influenza A	11.39343535	6.23627E-29	1.23166E-27	15894	Icam1
05164	Influenza A	11.39343535	6.23627E-29	1.23166E-27	16193	Il6
05164	Influenza A	11.39343535	6.23627E-29	1.23166E-27	12362	Casp1
05164	Influenza A	11.39343535	6.23627E-29	1.23166E-27	16176	Il1b
05164	Influenza A	11.39343535	6.23627E-29	1.23166E-27	22035	Tnfsf10
05164	Influenza A	11.39343535	6.23627E-29	1.23166E-27	110196	Fdps
05164	Influenza A	11.39343535	6.23627E-29	1.23166E-27	19106	Eif2ak2
05164	Influenza A	11.39343535	6.23627E-29	1.23166E-27	21933	Tnfrsf10b
05164	Influenza A	11.39343535	6.23627E-29	1.23166E-27	20293	Ccl12
05164	Influenza A	11.39343535	6.23627E-29	1.23166E-27	20296	Ccl2
05164	Influenza A	11.39343535	6.23627E-29	1.23166E-27	16175	Il1a
05164	Influenza A	11.39343535	6.23627E-29	1.23166E-27	58185	Rsad2
05164	Influenza A	11.39343535	6.23627E-29	1.23166E-27	16159	Il12a
05164	Influenza A	11.39343535	6.23627E-29	1.23166E-27	16452	Jak2
05164	Influenza A	11.39343535	6.23627E-29	1.23166E-27	13063	Cycs
05164	Influenza A	11.39343535	6.23627E-29	1.23166E-27	21926	Tnf
05164	Influenza A	11.39343535	6.23627E-29	1.23166E-27	20846	Stat1
05164	Influenza A	11.39343535	6.23627E-29	1.23166E-27	20847	Stat2
05164	Influenza A	11.39343535	6.23627E-29	1.23166E-27	20304	Ccl5
05164	Influenza A	11.39343535	6.23627E-29	1.23166E-27	16173	Il18
05164	Influenza A	11.39343535	6.23627E-29	1.23166E-27	15001	H2-Oa
05164	Influenza A	11.39343535	6.23627E-29	1.23166E-27	12702	Socs3
05164	Influenza A	11.39343535	6.23627E-29	1.23166E-27	18708	Pik3r1
05164	Influenza A	11.39343535	6.23627E-29	1.23166E-27	14102	Fas
05164	Influenza A	11.39343535	6.23627E-29	1.23166E-27	15978	Ifng
05164	Influenza A	11.39343535	6.23627E-29	1.23166E-27	216799	Nlrp3
05164	Influenza A	11.39343535	6.23627E-29	1.23166E-27	24014	Rnasel

05164	Influenza A	11.39343535	6.23627E-29	1.23166E-27	18036	Nfkbib
05164	Influenza A	11.39343535	6.23627E-29	1.23166E-27	18035	Nfkbia
05164	Influenza A	11.39343535	6.23627E-29	1.23166E-27	12122	Bid
05164	Influenza A	11.39343535	6.23627E-29	1.23166E-27	71586	Ifih1
05164	Influenza A	11.39343535	6.23627E-29	1.23166E-27	56480	Tbk1
05164	Influenza A	11.39343535	6.23627E-29	1.23166E-27	18033	Nfkb1
05164	Influenza A	11.39343535	6.23627E-29	1.23166E-27	23960	Oas1g
05164	Influenza A	11.39343535	6.23627E-29	1.23166E-27	230073	Ddx58
05164	Influenza A	11.39343535	6.23627E-29	1.23166E-27	54123	Irf7
05164	Influenza A	11.39343535	6.23627E-29	1.23166E-27	56489	Ikbke
05164	Influenza A	11.39343535	6.23627E-29	1.23166E-27	15945	Cxcl10
04625	C-type lectin receptor signaling pathway	14.8912826	9.5585E-29	1.77676E-27	19057	Ppp3cc
04625	C-type lectin receptor signaling pathway	14.8912826	9.5585E-29	1.77676E-27	16153	Il10
04625	C-type lectin receptor signaling pathway	14.8912826	9.5585E-29	1.77676E-27	21926	Tnf
04625	C-type lectin receptor signaling pathway	14.8912826	9.5585E-29	1.77676E-27	20847	Stat2
04625	C-type lectin receptor signaling pathway	14.8912826	9.5585E-29	1.77676E-27	56489	Ikbke
04625	C-type lectin receptor signaling pathway	14.8912826	9.5585E-29	1.77676E-27	19225	Ptgs2
04625	C-type lectin receptor signaling pathway	14.8912826	9.5585E-29	1.77676E-27	16193	Il6
04625	C-type lectin receptor signaling pathway	14.8912826	9.5585E-29	1.77676E-27	18035	Nfkbia
04625	C-type lectin receptor signaling pathway	14.8912826	9.5585E-29	1.77676E-27	12051	Bcl3
04625	C-type lectin receptor signaling pathway	14.8912826	9.5585E-29	1.77676E-27	18708	Pik3r1
04625	C-type lectin receptor signaling pathway	14.8912826	9.5585E-29	1.77676E-27	20779	Src
04625	C-type lectin receptor signaling pathway	14.8912826	9.5585E-29	1.77676E-27	18034	Nfkb2
04625	C-type lectin receptor signaling pathway	14.8912826	9.5585E-29	1.77676E-27	16176	Il1b
04625	C-type lectin receptor signaling pathway	14.8912826	9.5585E-29	1.77676E-27	12795	Plk3
04625	C-type lectin receptor signaling pathway	14.8912826	9.5585E-29	1.77676E-27	16362	Irf1
04625	C-type lectin receptor signaling pathway	14.8912826	9.5585E-29	1.77676E-27	17474	Clec4d
04625	C-type lectin receptor signaling pathway	14.8912826	9.5585E-29	1.77676E-27	16706	Ksr1
04625	C-type lectin receptor signaling pathway	14.8912826	9.5585E-29	1.77676E-27	240354	Malt1
04625	C-type lectin receptor signaling pathway	14.8912826	9.5585E-29	1.77676E-27	19698	Relb
04625	C-type lectin receptor signaling pathway	14.8912826	9.5585E-29	1.77676E-27	17164	Mapkapk2
04625	C-type lectin receptor signaling pathway	14.8912826	9.5585E-29	1.77676E-27	18033	Nfkb1
04625	C-type lectin receptor signaling pathway	14.8912826	9.5585E-29	1.77676E-27	12362	Casp1
04625	C-type lectin receptor signaling pathway	14.8912826	9.5585E-29	1.77676E-27	83430	Il23a
04625	C-type lectin receptor signaling pathway	14.8912826	9.5585E-29	1.77676E-27	16159	Il12a
04625	C-type lectin receptor signaling pathway	14.8912826	9.5585E-29	1.77676E-27	16160	Il12b
04625	C-type lectin receptor signaling pathway	14.8912826	9.5585E-29	1.77676E-27	56620	Clec4n
04625	C-type lectin receptor signaling pathway	14.8912826	9.5585E-29	1.77676E-27	20846	Stat1
04625	C-type lectin receptor signaling pathway	14.8912826	9.5585E-29	1.77676E-27	56619	Clec4e
04625	C-type lectin receptor signaling pathway	14.8912826	9.5585E-29	1.77676E-27	13655	Egr3
04625	C-type lectin receptor signaling pathway	14.8912826	9.5585E-29	1.77676E-27	20963	Syk
04625	C-type lectin receptor signaling pathway	14.8912826	9.5585E-29	1.77676E-27	20299	Ccl22

04625	C-type lectin receptor signaling pathway	14.8912826	9.5585E-29	1.77676E-27	216799	Nlrp3
04625	C-type lectin receptor signaling pathway	14.8912826	9.5585E-29	1.77676E-27	26415	Mapk13
05323	Rheumatoid arthritis	16.84670355	4.03375E-27	7.08147E-26	11973	Atp6v1e1
05323	Rheumatoid arthritis	16.84670355	4.03375E-27	7.08147E-26	20293	Ccl12
05323	Rheumatoid arthritis	16.84670355	4.03375E-27	7.08147E-26	16193	Il6
05323	Rheumatoid arthritis	16.84670355	4.03375E-27	7.08147E-26	16176	Il1b
05323	Rheumatoid arthritis	16.84670355	4.03375E-27	7.08147E-26	17392	Mmp3
05323	Rheumatoid arthritis	16.84670355	4.03375E-27	7.08147E-26	15001	H2-Oa
05323	Rheumatoid arthritis	16.84670355	4.03375E-27	7.08147E-26	20302	Ccl3
05323	Rheumatoid arthritis	16.84670355	4.03375E-27	7.08147E-26	12981	Csf2
05323	Rheumatoid arthritis	16.84670355	4.03375E-27	7.08147E-26	11433	Acp5
05323	Rheumatoid arthritis	16.84670355	4.03375E-27	7.08147E-26	20304	Ccl5
05323	Rheumatoid arthritis	16.84670355	4.03375E-27	7.08147E-26	16175	Il1a
05323	Rheumatoid arthritis	16.84670355	4.03375E-27	7.08147E-26	24088	Tlr2
05323	Rheumatoid arthritis	16.84670355	4.03375E-27	7.08147E-26	16173	Il18
05323	Rheumatoid arthritis	16.84670355	4.03375E-27	7.08147E-26	21926	Tnf
05323	Rheumatoid arthritis	16.84670355	4.03375E-27	7.08147E-26	83430	Il23a
05323	Rheumatoid arthritis	16.84670355	4.03375E-27	7.08147E-26	20296	Ccl2
05323	Rheumatoid arthritis	16.84670355	4.03375E-27	7.08147E-26	330122	Cxcl3
05323	Rheumatoid arthritis	16.84670355	4.03375E-27	7.08147E-26	15978	Ifng
05323	Rheumatoid arthritis	16.84670355	4.03375E-27	7.08147E-26	83996	Mmp1b
05323	Rheumatoid arthritis	16.84670355	4.03375E-27	7.08147E-26	14254	Flt1
05323	Rheumatoid arthritis	16.84670355	4.03375E-27	7.08147E-26	12977	Csf1
05323	Rheumatoid arthritis	16.84670355	4.03375E-27	7.08147E-26	12524	Cd86
05323	Rheumatoid arthritis	16.84670355	4.03375E-27	7.08147E-26	14825	Cxcl1
05323	Rheumatoid arthritis	16.84670355	4.03375E-27	7.08147E-26	11600	Angpt1
05323	Rheumatoid arthritis	16.84670355	4.03375E-27	7.08147E-26	20310	Cxcl2
05323	Rheumatoid arthritis	16.84670355	4.03375E-27	7.08147E-26	16408	Itgal
05323	Rheumatoid arthritis	16.84670355	4.03375E-27	7.08147E-26	15894	Icam1
05323	Rheumatoid arthritis	16.84670355	4.03375E-27	7.08147E-26	22339	Vegfa
05323	Rheumatoid arthritis	16.84670355	4.03375E-27	7.08147E-26	20311	Cxcl5
05132	Salmonella infection	8.589821178	1.80436E-26	3.00094E-25	18035	Nfkbia
05132	Salmonella infection	8.589821178	1.80436E-26	3.00094E-25	16193	Il6
05132	Salmonella infection	8.589821178	1.80436E-26	3.00094E-25	21926	Tnf
05132	Salmonella infection	8.589821178	1.80436E-26	3.00094E-25	56532	Ripk3
05132	Salmonella infection	8.589821178	1.80436E-26	3.00094E-25	12475	Cd14
05132	Salmonella infection	8.589821178	1.80436E-26	3.00094E-25	15519	Hsp90aa1
05132	Salmonella infection	8.589821178	1.80436E-26	3.00094E-25	14433	Gapdh
05132	Salmonella infection	8.589821178	1.80436E-26	3.00094E-25	16176	Il1b
05132	Salmonella infection	8.589821178	1.80436E-26	3.00094E-25	17087	Ly96
05132	Salmonella infection	8.589821178	1.80436E-26	3.00094E-25	13063	Cycs
05132	Salmonella infection	8.589821178	1.80436E-26	3.00094E-25	22027	Hsp90b1

05132	Salmonella infection	8.589821178	1.80436E-26	3.00094E-25	12362	Casp1
05132	Salmonella infection	8.589821178	1.80436E-26	3.00094E-25	18033	Nfkb1
05132	Salmonella infection	8.589821178	1.80436E-26	3.00094E-25	21899	Tlr6
05132	Salmonella infection	8.589821178	1.80436E-26	3.00094E-25	21933	Tnfrsf10b
05132	Salmonella infection	8.589821178	1.80436E-26	3.00094E-25	17948	Naip2
05132	Salmonella infection	8.589821178	1.80436E-26	3.00094E-25	192656	Ripk2
05132	Salmonella infection	8.589821178	1.80436E-26	3.00094E-25	22035	Tnfsf10
05132	Salmonella infection	8.589821178	1.80436E-26	3.00094E-25	24088	Tlr2
05132	Salmonella infection	8.589821178	1.80436E-26	3.00094E-25	16648	Kpna3
05132	Salmonella infection	8.589821178	1.80436E-26	3.00094E-25	26415	Mapk13
05132	Salmonella infection	8.589821178	1.80436E-26	3.00094E-25	16173	Il18
05132	Salmonella infection	8.589821178	1.80436E-26	3.00094E-25	216799	Nlrp3
05132	Salmonella infection	8.589821178	1.80436E-26	3.00094E-25	22166	Txn1
05132	Salmonella infection	8.589821178	1.80436E-26	3.00094E-25	22142	Tuba1a
05132	Salmonella infection	8.589821178	1.80436E-26	3.00094E-25	12363	Casp4
05132	Salmonella infection	8.589821178	1.80436E-26	3.00094E-25	107607	Nod1
05132	Salmonella infection	8.589821178	1.80436E-26	3.00094E-25	18645	Pfn2
05132	Salmonella infection	8.589821178	1.80436E-26	3.00094E-25	76884	Cyfip2
05132	Salmonella infection	8.589821178	1.80436E-26	3.00094E-25	22030	Traf2
05132	Salmonella infection	8.589821178	1.80436E-26	3.00094E-25	12369	Casp7
05132	Salmonella infection	8.589821178	1.80436E-26	3.00094E-25	74568	Mlkl
05132	Salmonella infection	8.589821178	1.80436E-26	3.00094E-25	17113	M6pr
05132	Salmonella infection	8.589821178	1.80436E-26	3.00094E-25	16649	Kpna4
05132	Salmonella infection	8.589821178	1.80436E-26	3.00094E-25	26399	Map2k6
05132	Salmonella infection	8.589821178	1.80436E-26	3.00094E-25	286940	Flnb
05132	Salmonella infection	8.589821178	1.80436E-26	3.00094E-25	170625	Snx18
05132	Salmonella infection	8.589821178	1.80436E-26	3.00094E-25	50884	Nckap1
05132	Salmonella infection	8.589821178	1.80436E-26	3.00094E-25	11796	Birc3
05132	Salmonella infection	8.589821178	1.80436E-26	3.00094E-25	22153	Tubb4a
05132	Salmonella infection	8.589821178	1.80436E-26	3.00094E-25	17869	Myc
05132	Salmonella infection	8.589821178	1.80436E-26	3.00094E-25	30955	Pik3cg
05132	Salmonella infection	8.589821178	1.80436E-26	3.00094E-25	22145	Tuba4a
04062	Chemokine signaling pathway	10.00273023	5.35458E-26	8.46024E-25	18035	Nfkbia
04062	Chemokine signaling pathway	10.00273023	5.35458E-26	8.46024E-25	20302	Ccl3
04062	Chemokine signaling pathway	10.00273023	5.35458E-26	8.46024E-25	14825	Cxcl1
04062	Chemokine signaling pathway	10.00273023	5.35458E-26	8.46024E-25	12769	Ccr9
04062	Chemokine signaling pathway	10.00273023	5.35458E-26	8.46024E-25	215449	Rap1b
04062	Chemokine signaling pathway	10.00273023	5.35458E-26	8.46024E-25	23832	Xcr1
04062	Chemokine signaling pathway	10.00273023	5.35458E-26	8.46024E-25	18036	Nfkbib
04062	Chemokine signaling pathway	10.00273023	5.35458E-26	8.46024E-25	18033	Nfkb1
04062	Chemokine signaling pathway	10.00273023	5.35458E-26	8.46024E-25	320207	Pik3r5
04062	Chemokine signaling pathway	10.00273023	5.35458E-26	8.46024E-25	30955	Pik3cg

04062	Chemokine signaling pathway	10.00273023	5.35458E-26	8.46024E-25	20308	Ccl9
04062	Chemokine signaling pathway	10.00273023	5.35458E-26	8.46024E-25	20296	Ccl2
04062	Chemokine signaling pathway	10.00273023	5.35458E-26	8.46024E-25	20293	Ccl12
04062	Chemokine signaling pathway	10.00273023	5.35458E-26	8.46024E-25	18708	Pik3r1
04062	Chemokine signaling pathway	10.00273023	5.35458E-26	8.46024E-25	20847	Stat2
04062	Chemokine signaling pathway	10.00273023	5.35458E-26	8.46024E-25	12775	Ccr7
04062	Chemokine signaling pathway	10.00273023	5.35458E-26	8.46024E-25	20310	Cxcl2
04062	Chemokine signaling pathway	10.00273023	5.35458E-26	8.46024E-25	57349	Ppbp
04062	Chemokine signaling pathway	10.00273023	5.35458E-26	8.46024E-25	20306	Ccl7
04062	Chemokine signaling pathway	10.00273023	5.35458E-26	8.46024E-25	20846	Stat1
04062	Chemokine signaling pathway	10.00273023	5.35458E-26	8.46024E-25	14701	Gng12
04062	Chemokine signaling pathway	10.00273023	5.35458E-26	8.46024E-25	12768	Ccr1
04062	Chemokine signaling pathway	10.00273023	5.35458E-26	8.46024E-25	17096	Lyn
04062	Chemokine signaling pathway	10.00273023	5.35458E-26	8.46024E-25	15945	Cxcl10
04062	Chemokine signaling pathway	10.00273023	5.35458E-26	8.46024E-25	17329	Cxcl9
04062	Chemokine signaling pathway	10.00273023	5.35458E-26	8.46024E-25	18798	Plcb4
04062	Chemokine signaling pathway	10.00273023	5.35458E-26	8.46024E-25	20303	Ccl4
04062	Chemokine signaling pathway	10.00273023	5.35458E-26	8.46024E-25	20304	Ccl5
04062	Chemokine signaling pathway	10.00273023	5.35458E-26	8.46024E-25	16452	Jak2
04062	Chemokine signaling pathway	10.00273023	5.35458E-26	8.46024E-25	330122	Cxcl3
04062	Chemokine signaling pathway	10.00273023	5.35458E-26	8.46024E-25	56221	Ccl24
04062	Chemokine signaling pathway	10.00273023	5.35458E-26	8.46024E-25	15162	Hck
04062	Chemokine signaling pathway	10.00273023	5.35458E-26	8.46024E-25	12927	Bcar1
04062	Chemokine signaling pathway	10.00273023	5.35458E-26	8.46024E-25	20779	Src
04062	Chemokine signaling pathway	10.00273023	5.35458E-26	8.46024E-25	20311	Cxcl5
04062	Chemokine signaling pathway	10.00273023	5.35458E-26	8.46024E-25	66102	Cxcl16
04062	Chemokine signaling pathway	10.00273023	5.35458E-26	8.46024E-25	20299	Ccl22
04062	Chemokine signaling pathway	10.00273023	5.35458E-26	8.46024E-25	56744	Pf4
04630	JAK-STAT signaling pathway	10.76594073	7.5243E-26	1.13223E-24	12444	Ccnd2
04630	JAK-STAT signaling pathway	10.76594073	7.5243E-26	1.13223E-24	12575	Cdkn1a
04630	JAK-STAT signaling pathway	10.76594073	7.5243E-26	1.13223E-24	56717	Mtor
04630	JAK-STAT signaling pathway	10.76594073	7.5243E-26	1.13223E-24	12986	Csf3r
04630	JAK-STAT signaling pathway	10.76594073	7.5243E-26	1.13223E-24	83430	Il23a
04630	JAK-STAT signaling pathway	10.76594073	7.5243E-26	1.13223E-24	12985	Csf3
04630	JAK-STAT signaling pathway	10.76594073	7.5243E-26	1.13223E-24	16165	Il13ra2
04630	JAK-STAT signaling pathway	10.76594073	7.5243E-26	1.13223E-24	329244	Il19
04630	JAK-STAT signaling pathway	10.76594073	7.5243E-26	1.13223E-24	12983	Csf2rb
04630	JAK-STAT signaling pathway	10.76594073	7.5243E-26	1.13223E-24	20847	Stat2
04630	JAK-STAT signaling pathway	10.76594073	7.5243E-26	1.13223E-24	16161	Il12rb1
04630	JAK-STAT signaling pathway	10.76594073	7.5243E-26	1.13223E-24	16164	Il13ra1
04630	JAK-STAT signaling pathway	10.76594073	7.5243E-26	1.13223E-24	16190	Il4ra
04630	JAK-STAT signaling pathway	10.76594073	7.5243E-26	1.13223E-24	16847	Lepr

04630	JAK-STAT signaling pathway	10.76594073	7.5243E-26	1.13223E-24	18712	Pim1
04630	JAK-STAT signaling pathway	10.76594073	7.5243E-26	1.13223E-24	209590	Il23r
04630	JAK-STAT signaling pathway	10.76594073	7.5243E-26	1.13223E-24	213208	Il20rb
04630	JAK-STAT signaling pathway	10.76594073	7.5243E-26	1.13223E-24	19255	Ptpn2
04630	JAK-STAT signaling pathway	10.76594073	7.5243E-26	1.13223E-24	16452	Jak2
04630	JAK-STAT signaling pathway	10.76594073	7.5243E-26	1.13223E-24	20846	Stat1
04630	JAK-STAT signaling pathway	10.76594073	7.5243E-26	1.13223E-24	12700	Cish
04630	JAK-STAT signaling pathway	10.76594073	7.5243E-26	1.13223E-24	11761	Aox1
04630	JAK-STAT signaling pathway	10.76594073	7.5243E-26	1.13223E-24	16153	Il10
04630	JAK-STAT signaling pathway	10.76594073	7.5243E-26	1.13223E-24	16159	Il12a
04630	JAK-STAT signaling pathway	10.76594073	7.5243E-26	1.13223E-24	13649	Egfr
04630	JAK-STAT signaling pathway	10.76594073	7.5243E-26	1.13223E-24	15978	Ifng
04630	JAK-STAT signaling pathway	10.76594073	7.5243E-26	1.13223E-24	18413	Osm
04630	JAK-STAT signaling pathway	10.76594073	7.5243E-26	1.13223E-24	18708	Pik3r1
04630	JAK-STAT signaling pathway	10.76594073	7.5243E-26	1.13223E-24	16193	Il6
04630	JAK-STAT signaling pathway	10.76594073	7.5243E-26	1.13223E-24	16878	Lif
04630	JAK-STAT signaling pathway	10.76594073	7.5243E-26	1.13223E-24	12702	Socs3
04630	JAK-STAT signaling pathway	10.76594073	7.5243E-26	1.13223E-24	216233	Socs2
04630	JAK-STAT signaling pathway	10.76594073	7.5243E-26	1.13223E-24	12703	Socs1
04630	JAK-STAT signaling pathway	10.76594073	7.5243E-26	1.13223E-24	12981	Csf2
04630	JAK-STAT signaling pathway	10.76594073	7.5243E-26	1.13223E-24	17869	Myc
04630	JAK-STAT signaling pathway	10.76594073	7.5243E-26	1.13223E-24	16160	Il12b
04066	HIF-1 signaling pathway	13.30002912	1.36396E-24	1.95914E-23	15277	Hk2
04066	HIF-1 signaling pathway	13.30002912	1.36396E-24	1.95914E-23	108058	Camk2d
04066	HIF-1 signaling pathway	13.30002912	1.36396E-24	1.95914E-23	13649	Egfr
04066	HIF-1 signaling pathway	13.30002912	1.36396E-24	1.95914E-23	15368	Hmox1
04066	HIF-1 signaling pathway	13.30002912	1.36396E-24	1.95914E-23	56717	Mtor
04066	HIF-1 signaling pathway	13.30002912	1.36396E-24	1.95914E-23	13684	Eif4e
04066	HIF-1 signaling pathway	13.30002912	1.36396E-24	1.95914E-23	14254	Flt1
04066	HIF-1 signaling pathway	13.30002912	1.36396E-24	1.95914E-23	18708	Pik3r1
04066	HIF-1 signaling pathway	13.30002912	1.36396E-24	1.95914E-23	13614	Edn1
04066	HIF-1 signaling pathway	13.30002912	1.36396E-24	1.95914E-23	18126	Nos2
04066	HIF-1 signaling pathway	13.30002912	1.36396E-24	1.95914E-23	170768	Pfkfb3
04066	HIF-1 signaling pathway	13.30002912	1.36396E-24	1.95914E-23	12575	Cdkn1a
04066	HIF-1 signaling pathway	13.30002912	1.36396E-24	1.95914E-23	20525	Slc2a1
04066	HIF-1 signaling pathway	13.30002912	1.36396E-24	1.95914E-23	22339	Vegfa
04066	HIF-1 signaling pathway	13.30002912	1.36396E-24	1.95914E-23	228026	Pdk1
04066	HIF-1 signaling pathway	13.30002912	1.36396E-24	1.95914E-23	15251	Hif1a
04066	HIF-1 signaling pathway	13.30002912	1.36396E-24	1.95914E-23	13058	Cybb
04066	HIF-1 signaling pathway	13.30002912	1.36396E-24	1.95914E-23	18655	Pgk1
04066	HIF-1 signaling pathway	13.30002912	1.36396E-24	1.95914E-23	67923	Eloc
04066	HIF-1 signaling pathway	13.30002912	1.36396E-24	1.95914E-23	18033	Nfkb1

04066	HIF-1 signaling pathway	13.30002912	1.36396E-24	1.95914E-23	112407	Egln3
04066	HIF-1 signaling pathway	13.30002912	1.36396E-24	1.95914E-23	16828	Ldha
04066	HIF-1 signaling pathway	13.30002912	1.36396E-24	1.95914E-23	18641	Pfkl
04066	HIF-1 signaling pathway	13.30002912	1.36396E-24	1.95914E-23	14433	Gapdh
04066	HIF-1 signaling pathway	13.30002912	1.36396E-24	1.95914E-23	13806	Eno1
04066	HIF-1 signaling pathway	13.30002912	1.36396E-24	1.95914E-23	433182	Eno1b
04066	HIF-1 signaling pathway	13.30002912	1.36396E-24	1.95914E-23	21857	Timp1
04066	HIF-1 signaling pathway	13.30002912	1.36396E-24	1.95914E-23	16193	Il6
04066	HIF-1 signaling pathway	13.30002912	1.36396E-24	1.95914E-23	11600	Angpt1
04066	HIF-1 signaling pathway	13.30002912	1.36396E-24	1.95914E-23	15978	Ifng
05163	Human cytomegalovirus infection	8.094314596	3.02966E-24	4.16249E-23	14990	H2-M2
05163	Human cytomegalovirus infection	8.094314596	3.02966E-24	4.16249E-23	20296	Ccl2
05163	Human cytomegalovirus infection	8.094314596	3.02966E-24	4.16249E-23	20293	Ccl12
05163	Human cytomegalovirus infection	8.094314596	3.02966E-24	4.16249E-23	14102	Fas
05163	Human cytomegalovirus infection	8.094314596	3.02966E-24	4.16249E-23	15024	H2-T10
05163	Human cytomegalovirus infection	8.094314596	3.02966E-24	4.16249E-23	12575	Cdkn1a
05163	Human cytomegalovirus infection	8.094314596	3.02966E-24	4.16249E-23	18798	Plcb4
05163	Human cytomegalovirus infection	8.094314596	3.02966E-24	4.16249E-23	19057	Ppp3cc
05163	Human cytomegalovirus infection	8.094314596	3.02966E-24	4.16249E-23	15039	H2-T22
05163	Human cytomegalovirus infection	8.094314596	3.02966E-24	4.16249E-23	15018	H2-Q7
05163	Human cytomegalovirus infection	8.094314596	3.02966E-24	4.16249E-23	13063	Cycs
05163	Human cytomegalovirus infection	8.094314596	3.02966E-24	4.16249E-23	20302	Ccl3
05163	Human cytomegalovirus infection	8.094314596	3.02966E-24	4.16249E-23	12927	Bcar1
05163	Human cytomegalovirus infection	8.094314596	3.02966E-24	4.16249E-23	110558	H2-Q9
05163	Human cytomegalovirus infection	8.094314596	3.02966E-24	4.16249E-23	231991	Creb5
05163	Human cytomegalovirus infection	8.094314596	3.02966E-24	4.16249E-23	14701	Gng12
05163	Human cytomegalovirus infection	8.094314596	3.02966E-24	4.16249E-23	21926	Tnf
05163	Human cytomegalovirus infection	8.094314596	3.02966E-24	4.16249E-23	18035	Nfkbia
05163	Human cytomegalovirus infection	8.094314596	3.02966E-24	4.16249E-23	19225	Ptgs2
05163	Human cytomegalovirus infection	8.094314596	3.02966E-24	4.16249E-23	26399	Map2k6
05163	Human cytomegalovirus infection	8.094314596	3.02966E-24	4.16249E-23	16177	Il1r1
05163	Human cytomegalovirus infection	8.094314596	3.02966E-24	4.16249E-23	22033	Traf5
05163	Human cytomegalovirus infection	8.094314596	3.02966E-24	4.16249E-23	12122	Bid
05163	Human cytomegalovirus infection	8.094314596	3.02966E-24	4.16249E-23	18708	Pik3r1
05163	Human cytomegalovirus infection	8.094314596	3.02966E-24	4.16249E-23	22339	Vegfa
05163	Human cytomegalovirus infection	8.094314596	3.02966E-24	4.16249E-23	20779	Src
05163	Human cytomegalovirus infection	8.094314596	3.02966E-24	4.16249E-23	214763	Cgas
05163	Human cytomegalovirus infection	8.094314596	3.02966E-24	4.16249E-23	16193	Il6
05163	Human cytomegalovirus infection	8.094314596	3.02966E-24	4.16249E-23	22030	Traf2
05163	Human cytomegalovirus infection	8.094314596	3.02966E-24	4.16249E-23	12317	Calr
05163	Human cytomegalovirus infection	8.094314596	3.02966E-24	4.16249E-23	20303	Ccl4
05163	Human cytomegalovirus infection	8.094314596	3.02966E-24	4.16249E-23	56717	Mtor

05163	Human cytomegalovirus infection	8.094314596	3.02966E-24	4.16249E-23	11911	Atf4
05163	Human cytomegalovirus infection	8.094314596	3.02966E-24	4.16249E-23	18033	Nfkb1
05163	Human cytomegalovirus infection	8.094314596	3.02966E-24	4.16249E-23	17869	Myc
05163	Human cytomegalovirus infection	8.094314596	3.02966E-24	4.16249E-23	26415	Mapk13
05163	Human cytomegalovirus infection	8.094314596	3.02966E-24	4.16249E-23	16176	Il1b
05163	Human cytomegalovirus infection	8.094314596	3.02966E-24	4.16249E-23	12768	Ccr1
05163	Human cytomegalovirus infection	8.094314596	3.02966E-24	4.16249E-23	20304	Ccl5
05163	Human cytomegalovirus infection	8.094314596	3.02966E-24	4.16249E-23	56480	Tbk1
05163	Human cytomegalovirus infection	8.094314596	3.02966E-24	4.16249E-23	13649	Egfr
04620	Toll-like receptor signaling pathway	14.15123098	7.86568E-24	1.03565E-22	54123	Irf7
04620	Toll-like receptor signaling pathway	14.15123098	7.86568E-24	1.03565E-22	225471	Ticam2
04620	Toll-like receptor signaling pathway	14.15123098	7.86568E-24	1.03565E-22	26415	Mapk13
04620	Toll-like receptor signaling pathway	14.15123098	7.86568E-24	1.03565E-22	26410	Map3k8
04620	Toll-like receptor signaling pathway	14.15123098	7.86568E-24	1.03565E-22	21939	Cd40
04620	Toll-like receptor signaling pathway	14.15123098	7.86568E-24	1.03565E-22	15945	Cxcl10
04620	Toll-like receptor signaling pathway	14.15123098	7.86568E-24	1.03565E-22	18033	Nfkb1
04620	Toll-like receptor signaling pathway	14.15123098	7.86568E-24	1.03565E-22	26399	Map2k6
04620	Toll-like receptor signaling pathway	14.15123098	7.86568E-24	1.03565E-22	21899	Tlr6
04620	Toll-like receptor signaling pathway	14.15123098	7.86568E-24	1.03565E-22	21897	Tlr1
04620	Toll-like receptor signaling pathway	14.15123098	7.86568E-24	1.03565E-22	12524	Cd86
04620	Toll-like receptor signaling pathway	14.15123098	7.86568E-24	1.03565E-22	20302	Ccl3
04620	Toll-like receptor signaling pathway	14.15123098	7.86568E-24	1.03565E-22	16176	Il1b
04620	Toll-like receptor signaling pathway	14.15123098	7.86568E-24	1.03565E-22	24088	Tlr2
04620	Toll-like receptor signaling pathway	14.15123098	7.86568E-24	1.03565E-22	20303	Ccl4
04620	Toll-like receptor signaling pathway	14.15123098	7.86568E-24	1.03565E-22	18035	Nfkbia
04620	Toll-like receptor signaling pathway	14.15123098	7.86568E-24	1.03565E-22	20304	Ccl5
04620	Toll-like receptor signaling pathway	14.15123098	7.86568E-24	1.03565E-22	56489	Ikbke
04620	Toll-like receptor signaling pathway	14.15123098	7.86568E-24	1.03565E-22	17087	Ly96
04620	Toll-like receptor signaling pathway	14.15123098	7.86568E-24	1.03565E-22	20846	Stat1
04620	Toll-like receptor signaling pathway	14.15123098	7.86568E-24	1.03565E-22	16193	Il6
04620	Toll-like receptor signaling pathway	14.15123098	7.86568E-24	1.03565E-22	16160	Il12b
04620	Toll-like receptor signaling pathway	14.15123098	7.86568E-24	1.03565E-22	12475	Cd14
04620	Toll-like receptor signaling pathway	14.15123098	7.86568E-24	1.03565E-22	17329	Cxcl9
04620	Toll-like receptor signaling pathway	14.15123098	7.86568E-24	1.03565E-22	16159	Il12a
04620	Toll-like receptor signaling pathway	14.15123098	7.86568E-24	1.03565E-22	21926	Tnf
04620	Toll-like receptor signaling pathway	14.15123098	7.86568E-24	1.03565E-22	56480	Tbk1
04620	Toll-like receptor signaling pathway	14.15123098	7.86568E-24	1.03565E-22	18708	Pik3r1
05171	Coronavirus disease - COVID-19	7.980017471	7.18588E-23	9.08296E-22	16176	Il1b
05171	Coronavirus disease - COVID-19	7.980017471	7.18588E-23	9.08296E-22	16159	Il12a
05171	Coronavirus disease - COVID-19	7.980017471	7.18588E-23	9.08296E-22	15945	Cxcl10
05171	Coronavirus disease - COVID-19	7.980017471	7.18588E-23	9.08296E-22	12981	Csf2
05171	Coronavirus disease - COVID-19	7.980017471	7.18588E-23	9.08296E-22	19106	Eif2ak2

05171	Coronavirus disease - COVID-19	7.980017471	7.18588E-23	9.08296E-22	56480	Tbk1
05171	Coronavirus disease - COVID-19	7.980017471	7.18588E-23	9.08296E-22	15200	Hbegf
05171	Coronavirus disease - COVID-19	7.980017471	7.18588E-23	9.08296E-22	667277	C1rb
05171	Coronavirus disease - COVID-19	7.980017471	7.18588E-23	9.08296E-22	50908	C1s1
05171	Coronavirus disease - COVID-19	7.980017471	7.18588E-23	9.08296E-22	16193	Il6
05171	Coronavirus disease - COVID-19	7.980017471	7.18588E-23	9.08296E-22	14962	Cfb
05171	Coronavirus disease - COVID-19	7.980017471	7.18588E-23	9.08296E-22	12266	C3
05171	Coronavirus disease - COVID-19	7.980017471	7.18588E-23	9.08296E-22	56489	Ikbke
05171	Coronavirus disease - COVID-19	7.980017471	7.18588E-23	9.08296E-22	13058	Cybb
05171	Coronavirus disease - COVID-19	7.980017471	7.18588E-23	9.08296E-22	13649	Egfr
05171	Coronavirus disease - COVID-19	7.980017471	7.18588E-23	9.08296E-22	20963	Syk
05171	Coronavirus disease - COVID-19	7.980017471	7.18588E-23	9.08296E-22	100038882	Isg15
05171	Coronavirus disease - COVID-19	7.980017471	7.18588E-23	9.08296E-22	216799	Nlrp3
05171	Coronavirus disease - COVID-19	7.980017471	7.18588E-23	9.08296E-22	12985	Csf3
05171	Coronavirus disease - COVID-19	7.980017471	7.18588E-23	9.08296E-22	20847	Stat2
05171	Coronavirus disease - COVID-19	7.980017471	7.18588E-23	9.08296E-22	18708	Pik3r1
05171	Coronavirus disease - COVID-19	7.980017471	7.18588E-23	9.08296E-22	23960	Oas1g
05171	Coronavirus disease - COVID-19	7.980017471	7.18588E-23	9.08296E-22	20296	Ccl2
05171	Coronavirus disease - COVID-19	7.980017471	7.18588E-23	9.08296E-22	20293	Ccl12
05171	Coronavirus disease - COVID-19	7.980017471	7.18588E-23	9.08296E-22	214763	Cgas
05171	Coronavirus disease - COVID-19	7.980017471	7.18588E-23	9.08296E-22	16160	Il12b
05171	Coronavirus disease - COVID-19	7.980017471	7.18588E-23	9.08296E-22	21926	Tnf
05171	Coronavirus disease - COVID-19	7.980017471	7.18588E-23	9.08296E-22	20846	Stat1
05171	Coronavirus disease - COVID-19	7.980017471	7.18588E-23	9.08296E-22	83996	Mmp1b
05171	Coronavirus disease - COVID-19	7.980017471	7.18588E-23	9.08296E-22	18035	Nfkbia
05171	Coronavirus disease - COVID-19	7.980017471	7.18588E-23	9.08296E-22	26415	Mapk13
05171	Coronavirus disease - COVID-19	7.980017471	7.18588E-23	9.08296E-22	11491	Adam17
05171	Coronavirus disease - COVID-19	7.980017471	7.18588E-23	9.08296E-22	12362	Casp1
05171	Coronavirus disease - COVID-19	7.980017471	7.18588E-23	9.08296E-22	18033	Nfkb1
05171	Coronavirus disease - COVID-19	7.980017471	7.18588E-23	9.08296E-22	24088	Tlr2
05171	Coronavirus disease - COVID-19	7.980017471	7.18588E-23	9.08296E-22	230073	Ddx58
05171	Coronavirus disease - COVID-19	7.980017471	7.18588E-23	9.08296E-22	71586	Ifih1
05171	Coronavirus disease - COVID-19	7.980017471	7.18588E-23	9.08296E-22	18036	Nfkbib
05171	Coronavirus disease - COVID-19	7.980017471	7.18588E-23	9.08296E-22	17392	Mmp3
04380	Osteoclast differentiation	11.45049382	8.55881E-22	1.01329E-20	15978	Ifng
04380	Osteoclast differentiation	11.45049382	8.55881E-22	1.01329E-20	18708	Pik3r1
04380	Osteoclast differentiation	11.45049382	8.55881E-22	1.01329E-20	20847	Stat2
04380	Osteoclast differentiation	11.45049382	8.55881E-22	1.01329E-20	16175	Il1a
04380	Osteoclast differentiation	11.45049382	8.55881E-22	1.01329E-20	16177	Il1r1
04380	Osteoclast differentiation	11.45049382	8.55881E-22	1.01329E-20	26415	Mapk13
04380	Osteoclast differentiation	11.45049382	8.55881E-22	1.01329E-20	12311	Calcr
04380	Osteoclast differentiation	11.45049382	8.55881E-22	1.01329E-20	16176	Il1b

04380	Osteoclast differentiation	11.45049382	8.55881E-22	1.01329E-20	21926	Tnf
04380	Osteoclast differentiation	11.45049382	8.55881E-22	1.01329E-20	14284	Fosl2
04380	Osteoclast differentiation	11.45049382	8.55881E-22	1.01329E-20	14283	Fosl1
04380	Osteoclast differentiation	11.45049382	8.55881E-22	1.01329E-20	20846	Stat1
04380	Osteoclast differentiation	11.45049382	8.55881E-22	1.01329E-20	14130	Fcgr2b
04380	Osteoclast differentiation	11.45049382	8.55881E-22	1.01329E-20	11433	Acp5
04380	Osteoclast differentiation	11.45049382	8.55881E-22	1.01329E-20	18383	Tnfrsf11b
04380	Osteoclast differentiation	11.45049382	8.55881E-22	1.01329E-20	16477	Junb
04380	Osteoclast differentiation	11.45049382	8.55881E-22	1.01329E-20	26399	Map2k6
04380	Osteoclast differentiation	11.45049382	8.55881E-22	1.01329E-20	12703	Socs1
04380	Osteoclast differentiation	11.45049382	8.55881E-22	1.01329E-20	12702	Socs3
04380	Osteoclast differentiation	11.45049382	8.55881E-22	1.01329E-20	18035	Nfkbia
04380	Osteoclast differentiation	11.45049382	8.55881E-22	1.01329E-20	22030	Traf2
04380	Osteoclast differentiation	11.45049382	8.55881E-22	1.01329E-20	19698	Relb
04380	Osteoclast differentiation	11.45049382	8.55881E-22	1.01329E-20	19057	Ppp3cc
04380	Osteoclast differentiation	11.45049382	8.55881E-22	1.01329E-20	18034	Nfkb2
04380	Osteoclast differentiation	11.45049382	8.55881E-22	1.01329E-20	18033	Nfkb1
04380	Osteoclast differentiation	11.45049382	8.55881E-22	1.01329E-20	16822	Lcp2
04380	Osteoclast differentiation	11.45049382	8.55881E-22	1.01329E-20	12977	Csf1
04380	Osteoclast differentiation	11.45049382	8.55881E-22	1.01329E-20	20963	Syk
04380	Osteoclast differentiation	11.45049382	8.55881E-22	1.01329E-20	18412	Sqstm1
05133	Pertussis	15.75276176	8.65782E-22	1.01329E-20	216799	Nlrp3
05133	Pertussis	15.75276176	8.65782E-22	1.01329E-20	16362	Irf1
05133	Pertussis	15.75276176	8.65782E-22	1.01329E-20	16160	Il12b
05133	Pertussis	15.75276176	8.65782E-22	1.01329E-20	83430	Il23a
05133	Pertussis	15.75276176	8.65782E-22	1.01329E-20	20311	Cxcl5
05133	Pertussis	15.75276176	8.65782E-22	1.01329E-20	225471	Ticam2
05133	Pertussis	15.75276176	8.65782E-22	1.01329E-20	16153	Il10
05133	Pertussis	15.75276176	8.65782E-22	1.01329E-20	12475	Cd14
05133	Pertussis	15.75276176	8.65782E-22	1.01329E-20	16159	Il12a
05133	Pertussis	15.75276176	8.65782E-22	1.01329E-20	12362	Casp1
05133	Pertussis	15.75276176	8.65782E-22	1.01329E-20	16193	Il6
05133	Pertussis	15.75276176	8.65782E-22	1.01329E-20	18126	Nos2
05133	Pertussis	15.75276176	8.65782E-22	1.01329E-20	107607	Nod1
05133	Pertussis	15.75276176	8.65782E-22	1.01329E-20	50908	C1s1
05133	Pertussis	15.75276176	8.65782E-22	1.01329E-20	26415	Mapk13
05133	Pertussis	15.75276176	8.65782E-22	1.01329E-20	12266	C3
05133	Pertussis	15.75276176	8.65782E-22	1.01329E-20	21926	Tnf
05133	Pertussis	15.75276176	8.65782E-22	1.01329E-20	667277	C1rb
05133	Pertussis	15.75276176	8.65782E-22	1.01329E-20	17087	Ly96
05133	Pertussis	15.75276176	8.65782E-22	1.01329E-20	16175	Il1a
05133	Pertussis	15.75276176	8.65782E-22	1.01329E-20	16176	Il1b

05133	Pertussis	15.75276176	8.65782E-22	1.01329E-20	12369	Casp7
05133	Pertussis	15.75276176	8.65782E-22	1.01329E-20	16402	Itga5
05133	Pertussis	15.75276176	8.65782E-22	1.01329E-20	18033	Nfkb1
05205	Proteoglycans in cancer	8.628799379	9.07942E-22	1.02468E-20	18708	Pik3r1
05205	Proteoglycans in cancer	8.628799379	9.07942E-22	1.02468E-20	329251	Ppp1r12b
05205	Proteoglycans in cancer	8.628799379	9.07942E-22	1.02468E-20	17698	Msn
05205	Proteoglycans in cancer	8.628799379	9.07942E-22	1.02468E-20	26415	Mapk13
05205	Proteoglycans in cancer	8.628799379	9.07942E-22	1.02468E-20	16402	Itga5
05205	Proteoglycans in cancer	8.628799379	9.07942E-22	1.02468E-20	24088	Tlr2
05205	Proteoglycans in cancer	8.628799379	9.07942E-22	1.02468E-20	108058	Camk2d
05205	Proteoglycans in cancer	8.628799379	9.07942E-22	1.02468E-20	17395	Mmp9
05205	Proteoglycans in cancer	8.628799379	9.07942E-22	1.02468E-20	17869	Myc
05205	Proteoglycans in cancer	8.628799379	9.07942E-22	1.02468E-20	13179	Dcn
05205	Proteoglycans in cancer	8.628799379	9.07942E-22	1.02468E-20	22350	Ezr
05205	Proteoglycans in cancer	8.628799379	9.07942E-22	1.02468E-20	22339	Vegfa
05205	Proteoglycans in cancer	8.628799379	9.07942E-22	1.02468E-20	20971	Sdc4
05205	Proteoglycans in cancer	8.628799379	9.07942E-22	1.02468E-20	18792	Plau
05205	Proteoglycans in cancer	8.628799379	9.07942E-22	1.02468E-20	18793	Plaur
05205	Proteoglycans in cancer	8.628799379	9.07942E-22	1.02468E-20	20969	Sdc1
05205	Proteoglycans in cancer	8.628799379	9.07942E-22	1.02468E-20	13649	Egfr
05205	Proteoglycans in cancer	8.628799379	9.07942E-22	1.02468E-20	22420	Wnt6
05205	Proteoglycans in cancer	8.628799379	9.07942E-22	1.02468E-20	14362	Fzd1
05205	Proteoglycans in cancer	8.628799379	9.07942E-22	1.02468E-20	17022	Lum
05205	Proteoglycans in cancer	8.628799379	9.07942E-22	1.02468E-20	17390	Mmp2
05205	Proteoglycans in cancer	8.628799379	9.07942E-22	1.02468E-20	286940	Flnb
05205	Proteoglycans in cancer	8.628799379	9.07942E-22	1.02468E-20	12389	Cav1
05205	Proteoglycans in cancer	8.628799379	9.07942E-22	1.02468E-20	15251	Hif1a
05205	Proteoglycans in cancer	8.628799379	9.07942E-22	1.02468E-20	14102	Fas
05205	Proteoglycans in cancer	8.628799379	9.07942E-22	1.02468E-20	14733	Gpc1
05205	Proteoglycans in cancer	8.628799379	9.07942E-22	1.02468E-20	15200	Hbegf
05205	Proteoglycans in cancer	8.628799379	9.07942E-22	1.02468E-20	16160	Il12b
05205	Proteoglycans in cancer	8.628799379	9.07942E-22	1.02468E-20	15163	Hcls1
05205	Proteoglycans in cancer	8.628799379	9.07942E-22	1.02468E-20	17295	Met
05205	Proteoglycans in cancer	8.628799379	9.07942E-22	1.02468E-20	21825	Thbs1
05205	Proteoglycans in cancer	8.628799379	9.07942E-22	1.02468E-20	20779	Src
05205	Proteoglycans in cancer	8.628799379	9.07942E-22	1.02468E-20	56717	Mtor
05205	Proteoglycans in cancer	8.628799379	9.07942E-22	1.02468E-20	12575	Cdkn1a
05205	Proteoglycans in cancer	8.628799379	9.07942E-22	1.02468E-20	21926	Tnf
05146	Amoebiasis	12.75311203	1.09711E-21	1.19547E-20	12475	Cd14
05146	Amoebiasis	12.75311203	1.09711E-21	1.19547E-20	15978	Ifng
05146	Amoebiasis	12.75311203	1.09711E-21	1.19547E-20	16160	Il12b
05146	Amoebiasis	12.75311203	1.09711E-21	1.19547E-20	12479	Cd1d1

05146	Amoebiasis	12.75311203	1.09711E-21	1.19547E-20	18477	Prdx1
05146	Amoebiasis	12.75311203	1.09711E-21	1.19547E-20	16159	Il12a
05146	Amoebiasis	12.75311203	1.09711E-21	1.19547E-20	11847	Arg2
05146	Amoebiasis	12.75311203	1.09711E-21	1.19547E-20	11846	Arg1
05146	Amoebiasis	12.75311203	1.09711E-21	1.19547E-20	16176	Il1b
05146	Amoebiasis	12.75311203	1.09711E-21	1.19547E-20	24088	Tlr2
05146	Amoebiasis	12.75311203	1.09711E-21	1.19547E-20	16782	Lamc2
05146	Amoebiasis	12.75311203	1.09711E-21	1.19547E-20	18033	Nfkb1
05146	Amoebiasis	12.75311203	1.09711E-21	1.19547E-20	18708	Pik3r1
05146	Amoebiasis	12.75311203	1.09711E-21	1.19547E-20	18798	Plcb4
05146	Amoebiasis	12.75311203	1.09711E-21	1.19547E-20	383548	Serpinb3b
05146	Amoebiasis	12.75311203	1.09711E-21	1.19547E-20	16153	Il10
05146	Amoebiasis	12.75311203	1.09711E-21	1.19547E-20	12827	Col4a2
05146	Amoebiasis	12.75311203	1.09711E-21	1.19547E-20	20310	Cxcl2
05146	Amoebiasis	12.75311203	1.09711E-21	1.19547E-20	22330	Vcl
05146	Amoebiasis	12.75311203	1.09711E-21	1.19547E-20	20723	Serpinb9
05146	Amoebiasis	12.75311203	1.09711E-21	1.19547E-20	18126	Nos2
05146	Amoebiasis	12.75311203	1.09711E-21	1.19547E-20	330122	Cxcl3
05146	Amoebiasis	12.75311203	1.09711E-21	1.19547E-20	12981	Csf2
05146	Amoebiasis	12.75311203	1.09711E-21	1.19547E-20	16193	Il6
05146	Amoebiasis	12.75311203	1.09711E-21	1.19547E-20	16177	Il1r1
05146	Amoebiasis	12.75311203	1.09711E-21	1.19547E-20	14825	Cxcl1
05146	Amoebiasis	12.75311203	1.09711E-21	1.19547E-20	21926	Tnf
05145	Toxoplasmosis	12.40529989	2.43504E-21	2.56491E-20	18035	Nfkbia
05145	Toxoplasmosis	12.40529989	2.43504E-21	2.56491E-20	18126	Nos2
05145	Toxoplasmosis	12.40529989	2.43504E-21	2.56491E-20	18036	Nfkbib
05145	Toxoplasmosis	12.40529989	2.43504E-21	2.56491E-20	20846	Stat1
05145	Toxoplasmosis	12.40529989	2.43504E-21	2.56491E-20	16452	Jak2
05145	Toxoplasmosis	12.40529989	2.43504E-21	2.56491E-20	18033	Nfkb1
05145	Toxoplasmosis	12.40529989	2.43504E-21	2.56491E-20	16145	Igtp
05145	Toxoplasmosis	12.40529989	2.43504E-21	2.56491E-20	16835	Ldlr
05145	Toxoplasmosis	12.40529989	2.43504E-21	2.56491E-20	16160	Il12b
05145	Toxoplasmosis	12.40529989	2.43504E-21	2.56491E-20	16153	Il10
05145	Toxoplasmosis	12.40529989	2.43504E-21	2.56491E-20	11796	Birc3
05145	Toxoplasmosis	12.40529989	2.43504E-21	2.56491E-20	15944	Irgm1
05145	Toxoplasmosis	12.40529989	2.43504E-21	2.56491E-20	15001	H2-Oa
05145	Toxoplasmosis	12.40529989	2.43504E-21	2.56491E-20	26415	Mapk13
05145	Toxoplasmosis	12.40529989	2.43504E-21	2.56491E-20	12703	Socs1
05145	Toxoplasmosis	12.40529989	2.43504E-21	2.56491E-20	24088	Tlr2
05145	Toxoplasmosis	12.40529989	2.43504E-21	2.56491E-20	15978	Ifng
05145	Toxoplasmosis	12.40529989	2.43504E-21	2.56491E-20	54396	Irgm2
05145	Toxoplasmosis	12.40529989	2.43504E-21	2.56491E-20	21939	Cd40

05145	Toxoplasmosis	12.40529989	2.43504E-21	2.56491E-20	26399	Map2k6
05145	Toxoplasmosis	12.40529989	2.43504E-21	2.56491E-20	17087	Ly96
05145	Toxoplasmosis	12.40529989	2.43504E-21	2.56491E-20	16159	Il12a
05145	Toxoplasmosis	12.40529989	2.43504E-21	2.56491E-20	21926	Tnf
05145	Toxoplasmosis	12.40529989	2.43504E-21	2.56491E-20	30955	Pik3cg
05145	Toxoplasmosis	12.40529989	2.43504E-21	2.56491E-20	320207	Pik3r5
05145	Toxoplasmosis	12.40529989	2.43504E-21	2.56491E-20	13063	Cycs
05145	Toxoplasmosis	12.40529989	2.43504E-21	2.56491E-20	16782	Lamc2
05022	Pathways of neurodegeneration - multiple diseases	5.353825281	4.26179E-21	4.34427E-20	26415	Mapk13
05022	Pathways of neurodegeneration - multiple diseases	5.353825281	4.26179E-21	4.34427E-20	14362	Fzd1
05022	Pathways of neurodegeneration - multiple diseases	5.353825281	4.26179E-21	4.34427E-20	16193	Il6
05022	Pathways of neurodegeneration - multiple diseases	5.353825281	4.26179E-21	4.34427E-20	16175	Il1a
05022	Pathways of neurodegeneration - multiple diseases	5.353825281	4.26179E-21	4.34427E-20	21938	Tnfrsf1b
05022	Pathways of neurodegeneration - multiple diseases	5.353825281	4.26179E-21	4.34427E-20	19173	Psmb5
05022	Pathways of neurodegeneration - multiple diseases	5.353825281	4.26179E-21	4.34427E-20	56480	Tbk1
05022	Pathways of neurodegeneration - multiple diseases	5.353825281	4.26179E-21	4.34427E-20	18033	Nfkb1
05022	Pathways of neurodegeneration - multiple diseases	5.353825281	4.26179E-21	4.34427E-20	13058	Cybb
05022	Pathways of neurodegeneration - multiple diseases	5.353825281	4.26179E-21	4.34427E-20	12369	Casp7
05022	Pathways of neurodegeneration - multiple diseases	5.353825281	4.26179E-21	4.34427E-20	73205	C9orf72
05022	Pathways of neurodegeneration - multiple diseases	5.353825281	4.26179E-21	4.34427E-20	22420	Wnt6
05022	Pathways of neurodegeneration - multiple diseases	5.353825281	4.26179E-21	4.34427E-20	18412	Sqstm1
05022	Pathways of neurodegeneration - multiple diseases	5.353825281	4.26179E-21	4.34427E-20	21353	Tank
05022	Pathways of neurodegeneration - multiple diseases	5.353825281	4.26179E-21	4.34427E-20	13163	Daxx
05022	Pathways of neurodegeneration - multiple diseases	5.353825281	4.26179E-21	4.34427E-20	56717	Mtor
05022	Pathways of neurodegeneration - multiple diseases	5.353825281	4.26179E-21	4.34427E-20	26399	Map2k6
05022	Pathways of neurodegeneration - multiple diseases	5.353825281	4.26179E-21	4.34427E-20	66725	Lrrk2
05022	Pathways of neurodegeneration - multiple diseases	5.353825281	4.26179E-21	4.34427E-20	20617	Snca
05022	Pathways of neurodegeneration - multiple diseases	5.353825281	4.26179E-21	4.34427E-20	19166	Psma2
05022	Pathways of neurodegeneration - multiple diseases	5.353825281	4.26179E-21	4.34427E-20	19181	Psmc2
05022	Pathways of neurodegeneration - multiple diseases	5.353825281	4.26179E-21	4.34427E-20	19175	Psmb6
05022	Pathways of neurodegeneration - multiple diseases	5.353825281	4.26179E-21	4.34427E-20	22145	Tuba4a
05022	Pathways of neurodegeneration - multiple diseases	5.353825281	4.26179E-21	4.34427E-20	26442	Psma5
05022	Pathways of neurodegeneration - multiple diseases	5.353825281	4.26179E-21	4.34427E-20	19057	Ppp3cc
05022	Pathways of neurodegeneration - multiple diseases	5.353825281	4.26179E-21	4.34427E-20	12977	Csf1
05022	Pathways of neurodegeneration - multiple diseases	5.353825281	4.26179E-21	4.34427E-20	16176	Il1b
05022	Pathways of neurodegeneration - multiple diseases	5.353825281	4.26179E-21	4.34427E-20	13063	Cycs
05022	Pathways of neurodegeneration - multiple diseases	5.353825281	4.26179E-21	4.34427E-20	22223	Uchl1
05022	Pathways of neurodegeneration - multiple diseases	5.353825281	4.26179E-21	4.34427E-20	21926	Tnf
05022	Pathways of neurodegeneration - multiple diseases	5.353825281	4.26179E-21	4.34427E-20	22153	Tubb4a
05022	Pathways of neurodegeneration - multiple diseases	5.353825281	4.26179E-21	4.34427E-20	18126	Nos2
05022	Pathways of neurodegeneration - multiple diseases	5.353825281	4.26179E-21	4.34427E-20	12122	Bid
05022	Pathways of neurodegeneration - multiple diseases	5.353825281	4.26179E-21	4.34427E-20	26443	Psma6

05022	Pathways of neurodegeneration - multiple diseases	5.353825281	4.26179E-21	4.34427E-20	19225	Ptgs2
05022	Pathways of neurodegeneration - multiple diseases	5.353825281	4.26179E-21	4.34427E-20	73916	Ift57
05022	Pathways of neurodegeneration - multiple diseases	5.353825281	4.26179E-21	4.34427E-20	108058	Camk2d
05022	Pathways of neurodegeneration - multiple diseases	5.353825281	4.26179E-21	4.34427E-20	14102	Fas
05022	Pathways of neurodegeneration - multiple diseases	5.353825281	4.26179E-21	4.34427E-20	14828	Hspa5
05022	Pathways of neurodegeneration - multiple diseases	5.353825281	4.26179E-21	4.34427E-20	59029	Psmd14
05022	Pathways of neurodegeneration - multiple diseases	5.353825281	4.26179E-21	4.34427E-20	69077	Psmd11
05022	Pathways of neurodegeneration - multiple diseases	5.353825281	4.26179E-21	4.34427E-20	22201	Uba1
05022	Pathways of neurodegeneration - multiple diseases	5.353825281	4.26179E-21	4.34427E-20	11911	Atf4
05022	Pathways of neurodegeneration - multiple diseases	5.353825281	4.26179E-21	4.34427E-20	22142	Tuba1a
05022	Pathways of neurodegeneration - multiple diseases	5.353825281	4.26179E-21	4.34427E-20	18798	Plcb4
05022	Pathways of neurodegeneration - multiple diseases	5.353825281	4.26179E-21	4.34427E-20	69926	Dnah17
05022	Pathways of neurodegeneration - multiple diseases	5.353825281	4.26179E-21	4.34427E-20	56791	Ube2l6
05022	Pathways of neurodegeneration - multiple diseases	5.353825281	4.26179E-21	4.34427E-20	14814	Grin2d
05022	Pathways of neurodegeneration - multiple diseases	5.353825281	4.26179E-21	4.34427E-20	56436	Adrm1
05022	Pathways of neurodegeneration - multiple diseases	5.353825281	4.26179E-21	4.34427E-20	22030	Traf2
05142	Chagas disease	12.75769783	6.42639E-21	6.34606E-20	19053	Ppp2cb
05142	Chagas disease	12.75769783	6.42639E-21	6.34606E-20	12266	C3
05142	Chagas disease	12.75769783	6.42639E-21	6.34606E-20	16153	Il10
05142	Chagas disease	12.75769783	6.42639E-21	6.34606E-20	14102	Fas
05142	Chagas disease	12.75769783	6.42639E-21	6.34606E-20	20304	Ccl5
05142	Chagas disease	12.75769783	6.42639E-21	6.34606E-20	18798	Plcb4
05142	Chagas disease	12.75769783	6.42639E-21	6.34606E-20	18708	Pik3r1
05142	Chagas disease	12.75769783	6.42639E-21	6.34606E-20	12062	Bdkrb2
05142	Chagas disease	12.75769783	6.42639E-21	6.34606E-20	26415	Mapk13
05142	Chagas disease	12.75769783	6.42639E-21	6.34606E-20	21899	Tlr6
05142	Chagas disease	12.75769783	6.42639E-21	6.34606E-20	12503	Cd247
05142	Chagas disease	12.75769783	6.42639E-21	6.34606E-20	12317	Calr
05142	Chagas disease	12.75769783	6.42639E-21	6.34606E-20	18126	Nos2
05142	Chagas disease	12.75769783	6.42639E-21	6.34606E-20	18033	Nfkb1
05142	Chagas disease	12.75769783	6.42639E-21	6.34606E-20	15978	Ifng
05142	Chagas disease	12.75769783	6.42639E-21	6.34606E-20	24088	Tlr2
05142	Chagas disease	12.75769783	6.42639E-21	6.34606E-20	16176	Il1b
05142	Chagas disease	12.75769783	6.42639E-21	6.34606E-20	20293	Ccl12
05142	Chagas disease	12.75769783	6.42639E-21	6.34606E-20	20302	Ccl3
05142	Chagas disease	12.75769783	6.42639E-21	6.34606E-20	16193	Il6
05142	Chagas disease	12.75769783	6.42639E-21	6.34606E-20	16160	Il12b
05142	Chagas disease	12.75769783	6.42639E-21	6.34606E-20	18035	Nfkbia
05142	Chagas disease	12.75769783	6.42639E-21	6.34606E-20	20296	Ccl2
05142	Chagas disease	12.75769783	6.42639E-21	6.34606E-20	12633	Cflar
05142	Chagas disease	12.75769783	6.42639E-21	6.34606E-20	21926	Tnf
05142	Chagas disease	12.75769783	6.42639E-21	6.34606E-20	16159	Il12a

05202	Transcriptional misregulation in cancer	7.932304362	1.59751E-20	1.52973E-19	18612	Etv4
05202	Transcriptional misregulation in cancer	7.932304362	1.59751E-20	1.52973E-19	14254	Flt1
05202	Transcriptional misregulation in cancer	7.932304362	1.59751E-20	1.52973E-19	20474	Six4
05202	Transcriptional misregulation in cancer	7.932304362	1.59751E-20	1.52973E-19	12981	Csf2
05202	Transcriptional misregulation in cancer	7.932304362	1.59751E-20	1.52973E-19	20471	Six1
05202	Transcriptional misregulation in cancer	7.932304362	1.59751E-20	1.52973E-19	16193	Il6
05202	Transcriptional misregulation in cancer	7.932304362	1.59751E-20	1.52973E-19	14009	Etv1
05202	Transcriptional misregulation in cancer	7.932304362	1.59751E-20	1.52973E-19	18124	Nr4a3
05202	Transcriptional misregulation in cancer	7.932304362	1.59751E-20	1.52973E-19	18033	Nfkb1
05202	Transcriptional misregulation in cancer	7.932304362	1.59751E-20	1.52973E-19	21939	Cd40
05202	Transcriptional misregulation in cancer	7.932304362	1.59751E-20	1.52973E-19	56312	Nupr1
05202	Transcriptional misregulation in cancer	7.932304362	1.59751E-20	1.52973E-19	12045	Bcl2a1b
05202	Transcriptional misregulation in cancer	7.932304362	1.59751E-20	1.52973E-19	21417	Zeb1
05202	Transcriptional misregulation in cancer	7.932304362	1.59751E-20	1.52973E-19	67603	Dusp6
05202	Transcriptional misregulation in cancer	7.932304362	1.59751E-20	1.52973E-19	12575	Cdkn1a
05202	Transcriptional misregulation in cancer	7.932304362	1.59751E-20	1.52973E-19	17873	Gadd45b
05202	Transcriptional misregulation in cancer	7.932304362	1.59751E-20	1.52973E-19	12608	Cebpb
05202	Transcriptional misregulation in cancer	7.932304362	1.59751E-20	1.52973E-19	12044	Bcl2a1a
05202	Transcriptional misregulation in cancer	7.932304362	1.59751E-20	1.52973E-19	12524	Cd86
05202	Transcriptional misregulation in cancer	7.932304362	1.59751E-20	1.52973E-19	12444	Ccnd2
05202	Transcriptional misregulation in cancer	7.932304362	1.59751E-20	1.52973E-19	18792	Plau
05202	Transcriptional misregulation in cancer	7.932304362	1.59751E-20	1.52973E-19	12046	Bcl2a1c
05202	Transcriptional misregulation in cancer	7.932304362	1.59751E-20	1.52973E-19	11864	Arnt2
05202	Transcriptional misregulation in cancer	7.932304362	1.59751E-20	1.52973E-19	11796	Birc3
05202	Transcriptional misregulation in cancer	7.932304362	1.59751E-20	1.52973E-19	17295	Met
05202	Transcriptional misregulation in cancer	7.932304362	1.59751E-20	1.52973E-19	15902	Id2
05202	Transcriptional misregulation in cancer	7.932304362	1.59751E-20	1.52973E-19	17392	Mmp3
05202	Transcriptional misregulation in cancer	7.932304362	1.59751E-20	1.52973E-19	17869	Myc
05202	Transcriptional misregulation in cancer	7.932304362	1.59751E-20	1.52973E-19	17395	Mmp9
05202	Transcriptional misregulation in cancer	7.932304362	1.59751E-20	1.52973E-19	12047	Bcl2a1d
05202	Transcriptional misregulation in cancer	7.932304362	1.59751E-20	1.52973E-19	80859	Nfkbiz
05202	Transcriptional misregulation in cancer	7.932304362	1.59751E-20	1.52973E-19	12475	Cd14
05202	Transcriptional misregulation in cancer	7.932304362	1.59751E-20	1.52973E-19	19696	Rel
05202	Transcriptional misregulation in cancer	7.932304362	1.59751E-20	1.52973E-19	22029	Traf1
05202	Transcriptional misregulation in cancer	7.932304362	1.59751E-20	1.52973E-19	13197	Gadd45a
04015	Rap1 signaling pathway	8.029737206	3.903E-20	3.62749E-19	14062	F2r
04015	Rap1 signaling pathway	8.029737206	3.903E-20	3.62749E-19	215449	Rap1b
04015	Rap1 signaling pathway	8.029737206	3.903E-20	3.62749E-19	16822	Lcp2
04015	Rap1 signaling pathway	8.029737206	3.903E-20	3.62749E-19	14293	Fpr1
04015	Rap1 signaling pathway	8.029737206	3.903E-20	3.62749E-19	14745	Lpar1
04015	Rap1 signaling pathway	8.029737206	3.903E-20	3.62749E-19	12927	Bcar1
04015	Rap1 signaling pathway	8.029737206	3.903E-20	3.62749E-19	18708	Pik3r1

04015	Rap1 signaling pathway	8.029737206	3.903E-20	3.62749E-19	18049	Ngf
04015	Rap1 signaling pathway	8.029737206	3.903E-20	3.62749E-19	11600	Angpt1
04015	Rap1 signaling pathway	8.029737206	3.903E-20	3.62749E-19	14164	Fgf1
04015	Rap1 signaling pathway	8.029737206	3.903E-20	3.62749E-19	12977	Csf1
04015	Rap1 signaling pathway	8.029737206	3.903E-20	3.62749E-19	20779	Src
04015	Rap1 signaling pathway	8.029737206	3.903E-20	3.62749E-19	13640	Efna5
04015	Rap1 signaling pathway	8.029737206	3.903E-20	3.62749E-19	11540	Adora2a
04015	Rap1 signaling pathway	8.029737206	3.903E-20	3.62749E-19	11541	Adora2b
04015	Rap1 signaling pathway	8.029737206	3.903E-20	3.62749E-19	16408	Itgal
04015	Rap1 signaling pathway	8.029737206	3.903E-20	3.62749E-19	217692	Sipa1l1
04015	Rap1 signaling pathway	8.029737206	3.903E-20	3.62749E-19	13637	Efna2
04015	Rap1 signaling pathway	8.029737206	3.903E-20	3.62749E-19	18798	Plcb4
04015	Rap1 signaling pathway	8.029737206	3.903E-20	3.62749E-19	21825	Thbs1
04015	Rap1 signaling pathway	8.029737206	3.903E-20	3.62749E-19	18645	Pfn2
04015	Rap1 signaling pathway	8.029737206	3.903E-20	3.62749E-19	22323	Vasp
04015	Rap1 signaling pathway	8.029737206	3.903E-20	3.62749E-19	17295	Met
04015	Rap1 signaling pathway	8.029737206	3.903E-20	3.62749E-19	14254	Flt1
04015	Rap1 signaling pathway	8.029737206	3.903E-20	3.62749E-19	16797	Lat
04015	Rap1 signaling pathway	8.029737206	3.903E-20	3.62749E-19	26415	Mapk13
04015	Rap1 signaling pathway	8.029737206	3.903E-20	3.62749E-19	26399	Map2k6
04015	Rap1 signaling pathway	8.029737206	3.903E-20	3.62749E-19	19730	Ralgds
04015	Rap1 signaling pathway	8.029737206	3.903E-20	3.62749E-19	107746	Rapgef1
04015	Rap1 signaling pathway	8.029737206	3.903E-20	3.62749E-19	22341	Vegfc
04015	Rap1 signaling pathway	8.029737206	3.903E-20	3.62749E-19	18654	Pgf
04015	Rap1 signaling pathway	8.029737206	3.903E-20	3.62749E-19	22339	Vegfa
04015	Rap1 signaling pathway	8.029737206	3.903E-20	3.62749E-19	13649	Egfr
04015	Rap1 signaling pathway	8.029737206	3.903E-20	3.62749E-19	76089	Rapgef2
04210	Apoptosis	10.4053169	7.2193E-20	6.518E-19	12046	Bcl2a1c
04210	Apoptosis	10.4053169	7.2193E-20	6.518E-19	12045	Bcl2a1b
04210	Apoptosis	10.4053169	7.2193E-20	6.518E-19	12047	Bcl2a1d
04210	Apoptosis	10.4053169	7.2193E-20	6.518E-19	11911	Atf4
04210	Apoptosis	10.4053169	7.2193E-20	6.518E-19	11796	Birc3
04210	Apoptosis	10.4053169	7.2193E-20	6.518E-19	18035	Nfkbia
04210	Apoptosis	10.4053169	7.2193E-20	6.518E-19	18049	Ngf
04210	Apoptosis	10.4053169	7.2193E-20	6.518E-19	14102	Fas
04210	Apoptosis	10.4053169	7.2193E-20	6.518E-19	12369	Casp7
04210	Apoptosis	10.4053169	7.2193E-20	6.518E-19	13163	Daxx
04210	Apoptosis	10.4053169	7.2193E-20	6.518E-19	17873	Gadd45b
04210	Apoptosis	10.4053169	7.2193E-20	6.518E-19	12044	Bcl2a1a
04210	Apoptosis	10.4053169	7.2193E-20	6.518E-19	22142	Tuba1a
04210	Apoptosis	10.4053169	7.2193E-20	6.518E-19	21926	Tnf
04210	Apoptosis	10.4053169	7.2193E-20	6.518E-19	18033	Nfkb1

04210	Apoptosis	10.4053169	7.2193E-20	6.518E-19	13063	Cycs
04210	Apoptosis	10.4053169	7.2193E-20	6.518E-19	13197	Gadd45a
04210	Apoptosis	10.4053169	7.2193E-20	6.518E-19	22030	Traf2
04210	Apoptosis	10.4053169	7.2193E-20	6.518E-19	22145	Tuba4a
04210	Apoptosis	10.4053169	7.2193E-20	6.518E-19	22035	Tnfsf10
04210	Apoptosis	10.4053169	7.2193E-20	6.518E-19	22029	Traf1
04210	Apoptosis	10.4053169	7.2193E-20	6.518E-19	12122	Bid
04210	Apoptosis	10.4053169	7.2193E-20	6.518E-19	13032	Ctsc
04210	Apoptosis	10.4053169	7.2193E-20	6.518E-19	12633	Cflar
04210	Apoptosis	10.4053169	7.2193E-20	6.518E-19	12983	Csf2rb
04210	Apoptosis	10.4053169	7.2193E-20	6.518E-19	69601	Dab2ip
04210	Apoptosis	10.4053169	7.2193E-20	6.518E-19	21933	Tnfrsf10b
04210	Apoptosis	10.4053169	7.2193E-20	6.518E-19	18708	Pik3r1
05160	Hepatitis C	9.189111027	1.36512E-19	1.19827E-18	112419	Ifit1bl2
05160	Hepatitis C	9.189111027	1.36512E-19	1.19827E-18	13649	Egfr
05160	Hepatitis C	9.189111027	1.36512E-19	1.19827E-18	19053	Ppp2cb
05160	Hepatitis C	9.189111027	1.36512E-19	1.19827E-18	18033	Nfkb1
05160	Hepatitis C	9.189111027	1.36512E-19	1.19827E-18	23960	Oas1g
05160	Hepatitis C	9.189111027	1.36512E-19	1.19827E-18	12702	Socs3
05160	Hepatitis C	9.189111027	1.36512E-19	1.19827E-18	18708	Pik3r1
05160	Hepatitis C	9.189111027	1.36512E-19	1.19827E-18	667373	Ifit1bl1
05160	Hepatitis C	9.189111027	1.36512E-19	1.19827E-18	15945	Cxcl10
05160	Hepatitis C	9.189111027	1.36512E-19	1.19827E-18	56489	Ikbke
05160	Hepatitis C	9.189111027	1.36512E-19	1.19827E-18	15957	Ifit1
05160	Hepatitis C	9.189111027	1.36512E-19	1.19827E-18	20846	Stat1
05160	Hepatitis C	9.189111027	1.36512E-19	1.19827E-18	24014	Rnasel
05160	Hepatitis C	9.189111027	1.36512E-19	1.19827E-18	19106	Eif2ak2
05160	Hepatitis C	9.189111027	1.36512E-19	1.19827E-18	230073	Ddx58
05160	Hepatitis C	9.189111027	1.36512E-19	1.19827E-18	58185	Rsad2
05160	Hepatitis C	9.189111027	1.36512E-19	1.19827E-18	18035	Nfkbia
05160	Hepatitis C	9.189111027	1.36512E-19	1.19827E-18	56480	Tbk1
05160	Hepatitis C	9.189111027	1.36512E-19	1.19827E-18	20847	Stat2
05160	Hepatitis C	9.189111027	1.36512E-19	1.19827E-18	13063	Cycs
05160	Hepatitis C	9.189111027	1.36512E-19	1.19827E-18	21926	Tnf
05160	Hepatitis C	9.189111027	1.36512E-19	1.19827E-18	12122	Bid
05160	Hepatitis C	9.189111027	1.36512E-19	1.19827E-18	12575	Cdkn1a
05160	Hepatitis C	9.189111027	1.36512E-19	1.19827E-18	16835	Ldlr
05160	Hepatitis C	9.189111027	1.36512E-19	1.19827E-18	54123	Irf7
05160	Hepatitis C	9.189111027	1.36512E-19	1.19827E-18	15978	Ifng
05160	Hepatitis C	9.189111027	1.36512E-19	1.19827E-18	14102	Fas
05160	Hepatitis C	9.189111027	1.36512E-19	1.19827E-18	22030	Traf2
05160	Hepatitis C	9.189111027	1.36512E-19	1.19827E-18	17869	Myc

05160	Hepatitis C	9.189111027	1.36512E-19	1.19827E-18	12633	Cflar
05166	Human T-cell leukemia virus 1 infection	7.161554141	4.8385E-19	4.13234E-18	12981	Csf2
05166	Human T-cell leukemia virus 1 infection	7.161554141	4.8385E-19	4.13234E-18	110558	H2-Q9
05166	Human T-cell leukemia virus 1 infection	7.161554141	4.8385E-19	4.13234E-18	16992	Lta
05166	Human T-cell leukemia virus 1 infection	7.161554141	4.8385E-19	4.13234E-18	15024	H2-T10
05166	Human T-cell leukemia virus 1 infection	7.161554141	4.8385E-19	4.13234E-18	15018	H2-Q7
05166	Human T-cell leukemia virus 1 infection	7.161554141	4.8385E-19	4.13234E-18	15039	H2-T22
05166	Human T-cell leukemia virus 1 infection	7.161554141	4.8385E-19	4.13234E-18	18034	Nfkb2
05166	Human T-cell leukemia virus 1 infection	7.161554141	4.8385E-19	4.13234E-18	14990	H2-M2
05166	Human T-cell leukemia virus 1 infection	7.161554141	4.8385E-19	4.13234E-18	18035	Nfkbia
05166	Human T-cell leukemia virus 1 infection	7.161554141	4.8385E-19	4.13234E-18	19698	Relb
05166	Human T-cell leukemia virus 1 infection	7.161554141	4.8385E-19	4.13234E-18	16177	Il1r1
05166	Human T-cell leukemia virus 1 infection	7.161554141	4.8385E-19	4.13234E-18	12575	Cdkn1a
05166	Human T-cell leukemia virus 1 infection	7.161554141	4.8385E-19	4.13234E-18	16193	Il6
05166	Human T-cell leukemia virus 1 infection	7.161554141	4.8385E-19	4.13234E-18	15894	Icam1
05166	Human T-cell leukemia virus 1 infection	7.161554141	4.8385E-19	4.13234E-18	231991	Creb5
05166	Human T-cell leukemia virus 1 infection	7.161554141	4.8385E-19	4.13234E-18	22695	Zfp36
05166	Human T-cell leukemia virus 1 infection	7.161554141	4.8385E-19	4.13234E-18	19057	Ppp3cc
05166	Human T-cell leukemia virus 1 infection	7.161554141	4.8385E-19	4.13234E-18	18033	Nfkb1
05166	Human T-cell leukemia virus 1 infection	7.161554141	4.8385E-19	4.13234E-18	16408	Itgal
05166	Human T-cell leukemia virus 1 infection	7.161554141	4.8385E-19	4.13234E-18	17869	Myc
05166	Human T-cell leukemia virus 1 infection	7.161554141	4.8385E-19	4.13234E-18	12317	Calr
05166	Human T-cell leukemia virus 1 infection	7.161554141	4.8385E-19	4.13234E-18	20525	Slc2a1
05166	Human T-cell leukemia virus 1 infection	7.161554141	4.8385E-19	4.13234E-18	12444	Ccnd2
05166	Human T-cell leukemia virus 1 infection	7.161554141	4.8385E-19	4.13234E-18	21939	Cd40
05166	Human T-cell leukemia virus 1 infection	7.161554141	4.8385E-19	4.13234E-18	18708	Pik3r1
05166	Human T-cell leukemia virus 1 infection	7.161554141	4.8385E-19	4.13234E-18	15001	H2-Oa
05166	Human T-cell leukemia virus 1 infection	7.161554141	4.8385E-19	4.13234E-18	12257	Tspo
05166	Human T-cell leukemia virus 1 infection	7.161554141	4.8385E-19	4.13234E-18	13653	Egr1
05166	Human T-cell leukemia virus 1 infection	7.161554141	4.8385E-19	4.13234E-18	21926	Tnf
05166	Human T-cell leukemia virus 1 infection	7.161554141	4.8385E-19	4.13234E-18	14283	Fosl1
05166	Human T-cell leukemia virus 1 infection	7.161554141	4.8385E-19	4.13234E-18	12579	Cdkn2b
05166	Human T-cell leukemia virus 1 infection	7.161554141	4.8385E-19	4.13234E-18	23872	Ets2
05166	Human T-cell leukemia virus 1 infection	7.161554141	4.8385E-19	4.13234E-18	110196	Fdps
05166	Human T-cell leukemia virus 1 infection	7.161554141	4.8385E-19	4.13234E-18	23871	Ets1
05166	Human T-cell leukemia virus 1 infection	7.161554141	4.8385E-19	4.13234E-18	11911	Atf4
04510	Focal adhesion	8.04618677	4.98951E-19	4.14917E-18	19417	Rasgrf1
04510	Focal adhesion	8.04618677	4.98951E-19	4.14917E-18	20779	Src
04510	Focal adhesion	8.04618677	4.98951E-19	4.14917E-18	215449	Rap1b
04510	Focal adhesion	8.04618677	4.98951E-19	4.14917E-18	18720	Pip5k1a
04510	Focal adhesion	8.04618677	4.98951E-19	4.14917E-18	18719	Pip5k1b
04510	Focal adhesion	8.04618677	4.98951E-19	4.14917E-18	16402	Itga5

04510	Focal adhesion	8.04618677	4.98951E-19	4.14917E-18	12827	Col4a2
04510	Focal adhesion	8.04618677	4.98951E-19	4.14917E-18	12444	Ccnd2
04510	Focal adhesion	8.04618677	4.98951E-19	4.14917E-18	18708	Pik3r1
04510	Focal adhesion	8.04618677	4.98951E-19	4.14917E-18	11796	Birc3
04510	Focal adhesion	8.04618677	4.98951E-19	4.14917E-18	228785	Mylk2
04510	Focal adhesion	8.04618677	4.98951E-19	4.14917E-18	107746	Rapgef1
04510	Focal adhesion	8.04618677	4.98951E-19	4.14917E-18	22323	Vasp
04510	Focal adhesion	8.04618677	4.98951E-19	4.14917E-18	12389	Cav1
04510	Focal adhesion	8.04618677	4.98951E-19	4.14917E-18	14254	Flt1
04510	Focal adhesion	8.04618677	4.98951E-19	4.14917E-18	21923	Tnc
04510	Focal adhesion	8.04618677	4.98951E-19	4.14917E-18	22330	Vcl
04510	Focal adhesion	8.04618677	4.98951E-19	4.14917E-18	286940	Flnb
04510	Focal adhesion	8.04618677	4.98951E-19	4.14917E-18	319480	Itga11
04510	Focal adhesion	8.04618677	4.98951E-19	4.14917E-18	329251	Ppp1r12b
04510	Focal adhesion	8.04618677	4.98951E-19	4.14917E-18	17295	Met
04510	Focal adhesion	8.04618677	4.98951E-19	4.14917E-18	12927	Bcar1
04510	Focal adhesion	8.04618677	4.98951E-19	4.14917E-18	329278	Tnn
04510	Focal adhesion	8.04618677	4.98951E-19	4.14917E-18	109700	Itga1
04510	Focal adhesion	8.04618677	4.98951E-19	4.14917E-18	22341	Vegfc
04510	Focal adhesion	8.04618677	4.98951E-19	4.14917E-18	13649	Egfr
04510	Focal adhesion	8.04618677	4.98951E-19	4.14917E-18	16782	Lamc2
04510	Focal adhesion	8.04618677	4.98951E-19	4.14917E-18	104099	Itga9
04510	Focal adhesion	8.04618677	4.98951E-19	4.14917E-18	320910	Itgb8
04510	Focal adhesion	8.04618677	4.98951E-19	4.14917E-18	21825	Thbs1
04510	Focal adhesion	8.04618677	4.98951E-19	4.14917E-18	22339	Vegfa
04510	Focal adhesion	8.04618677	4.98951E-19	4.14917E-18	18654	Pgf
05134	Legionellosis	16.57052808	8.59406E-19	6.9634E-18	12362	Casp1
05134	Legionellosis	16.57052808	8.59406E-19	6.9634E-18	18035	Nfkbia
05134	Legionellosis	16.57052808	8.59406E-19	6.9634E-18	12266	C3
05134	Legionellosis	16.57052808	8.59406E-19	6.9634E-18	13063	Cycs
05134	Legionellosis	16.57052808	8.59406E-19	6.9634E-18	16193	Il6
05134	Legionellosis	16.57052808	8.59406E-19	6.9634E-18	16176	Il1b
05134	Legionellosis	16.57052808	8.59406E-19	6.9634E-18	18033	Nfkb1
05134	Legionellosis	16.57052808	8.59406E-19	6.9634E-18	12176	Bnip3
05134	Legionellosis	16.57052808	8.59406E-19	6.9634E-18	18034	Nfkb2
05134	Legionellosis	16.57052808	8.59406E-19	6.9634E-18	16173	Il18
05134	Legionellosis	16.57052808	8.59406E-19	6.9634E-18	17948	Naip2
05134	Legionellosis	16.57052808	8.59406E-19	6.9634E-18	24088	Tlr2
05134	Legionellosis	16.57052808	8.59406E-19	6.9634E-18	12369	Casp7
05134	Legionellosis	16.57052808	8.59406E-19	6.9634E-18	20310	Cxcl2
05134	Legionellosis	16.57052808	8.59406E-19	6.9634E-18	12475	Cd14
05134	Legionellosis	16.57052808	8.59406E-19	6.9634E-18	330122	Cxcl3

05134	Legionellosis	16.57052808	8.59406E-19	6.9634E-18	14825	Cxcl1
05134	Legionellosis	16.57052808	8.59406E-19	6.9634E-18	21926	Tnf
05134	Legionellosis	16.57052808	8.59406E-19	6.9634E-18	16159	Il12a
05134	Legionellosis	16.57052808	8.59406E-19	6.9634E-18	16160	Il12b
05165	Human papillomavirus infection	5.724156179	1.68237E-18	1.32907E-17	12444	Ccnd2
05165	Human papillomavirus infection	5.724156179	1.68237E-18	1.32907E-17	100038882	Isg15
05165	Human papillomavirus infection	5.724156179	1.68237E-18	1.32907E-17	16449	Jag1
05165	Human papillomavirus infection	5.724156179	1.68237E-18	1.32907E-17	18128	Notch1
05165	Human papillomavirus infection	5.724156179	1.68237E-18	1.32907E-17	18033	Nfkb1
05165	Human papillomavirus infection	5.724156179	1.68237E-18	1.32907E-17	22339	Vegfa
05165	Human papillomavirus infection	5.724156179	1.68237E-18	1.32907E-17	231655	Oasl1
05165	Human papillomavirus infection	5.724156179	1.68237E-18	1.32907E-17	12575	Cdkn1a
05165	Human papillomavirus infection	5.724156179	1.68237E-18	1.32907E-17	23962	Oasl2
05165	Human papillomavirus infection	5.724156179	1.68237E-18	1.32907E-17	21926	Tnf
05165	Human papillomavirus infection	5.724156179	1.68237E-18	1.32907E-17	14102	Fas
05165	Human papillomavirus infection	5.724156179	1.68237E-18	1.32907E-17	320910	Itgb8
05165	Human papillomavirus infection	5.724156179	1.68237E-18	1.32907E-17	104099	Itga9
05165	Human papillomavirus infection	5.724156179	1.68237E-18	1.32907E-17	231991	Creb5
05165	Human papillomavirus infection	5.724156179	1.68237E-18	1.32907E-17	56489	Ikbke
05165	Human papillomavirus infection	5.724156179	1.68237E-18	1.32907E-17	12827	Col4a2
05165	Human papillomavirus infection	5.724156179	1.68237E-18	1.32907E-17	19106	Eif2ak2
05165	Human papillomavirus infection	5.724156179	1.68237E-18	1.32907E-17	13649	Egfr
05165	Human papillomavirus infection	5.724156179	1.68237E-18	1.32907E-17	16782	Lamc2
05165	Human papillomavirus infection	5.724156179	1.68237E-18	1.32907E-17	14362	Fzd1
05165	Human papillomavirus infection	5.724156179	1.68237E-18	1.32907E-17	20846	Stat1
05165	Human papillomavirus infection	5.724156179	1.68237E-18	1.32907E-17	18708	Pik3r1
05165	Human papillomavirus infection	5.724156179	1.68237E-18	1.32907E-17	110558	H2-Q9
05165	Human papillomavirus infection	5.724156179	1.68237E-18	1.32907E-17	21923	Tnc
05165	Human papillomavirus infection	5.724156179	1.68237E-18	1.32907E-17	22420	Wnt6
05165	Human papillomavirus infection	5.724156179	1.68237E-18	1.32907E-17	109700	Itga1
05165	Human papillomavirus infection	5.724156179	1.68237E-18	1.32907E-17	329278	Tnn
05165	Human papillomavirus infection	5.724156179	1.68237E-18	1.32907E-17	16402	Itga5
05165	Human papillomavirus infection	5.724156179	1.68237E-18	1.32907E-17	14990	H2-M2
05165	Human papillomavirus infection	5.724156179	1.68237E-18	1.32907E-17	21825	Thbs1
05165	Human papillomavirus infection	5.724156179	1.68237E-18	1.32907E-17	15039	H2-T22
05165	Human papillomavirus infection	5.724156179	1.68237E-18	1.32907E-17	19053	Ppp2cb
05165	Human papillomavirus infection	5.724156179	1.68237E-18	1.32907E-17	11973	Atp6v1e1
05165	Human papillomavirus infection	5.724156179	1.68237E-18	1.32907E-17	319480	Itga11
05165	Human papillomavirus infection	5.724156179	1.68237E-18	1.32907E-17	56717	Mtor
05165	Human papillomavirus infection	5.724156179	1.68237E-18	1.32907E-17	20847	Stat2
05165	Human papillomavirus infection	5.724156179	1.68237E-18	1.32907E-17	15018	H2-Q7
05165	Human papillomavirus infection	5.724156179	1.68237E-18	1.32907E-17	15024	H2-T10

05165	Human papillomavirus infection	5.724156179	1.68237E-18	1.32907E-17	19225	Ptgs2
05165	Human papillomavirus infection	5.724156179	1.68237E-18	1.32907E-17	16362	Irf1
05165	Human papillomavirus infection	5.724156179	1.68237E-18	1.32907E-17	56480	Tbk1
04623	Cytosolic DNA-sensing pathway	16.04447957	1.79035E-18	1.37988E-17	20304	Ccl5
04623	Cytosolic DNA-sensing pathway	16.04447957	1.79035E-18	1.37988E-17	22040	Trex1
04623	Cytosolic DNA-sensing pathway	16.04447957	1.79035E-18	1.37988E-17	16193	Il6
04623	Cytosolic DNA-sensing pathway	16.04447957	1.79035E-18	1.37988E-17	18033	Nfkb1
04623	Cytosolic DNA-sensing pathway	16.04447957	1.79035E-18	1.37988E-17	67065	Polr3d
04623	Cytosolic DNA-sensing pathway	16.04447957	1.79035E-18	1.37988E-17	214763	Cgas
04623	Cytosolic DNA-sensing pathway	16.04447957	1.79035E-18	1.37988E-17	20303	Ccl4
04623	Cytosolic DNA-sensing pathway	16.04447957	1.79035E-18	1.37988E-17	15945	Cxcl10
04623	Cytosolic DNA-sensing pathway	16.04447957	1.79035E-18	1.37988E-17	58203	Zbp1
04623	Cytosolic DNA-sensing pathway	16.04447957	1.79035E-18	1.37988E-17	56532	Ripk3
04623	Cytosolic DNA-sensing pathway	16.04447957	1.79035E-18	1.37988E-17	18036	Nfkbib
04623	Cytosolic DNA-sensing pathway	16.04447957	1.79035E-18	1.37988E-17	16173	Il18
04623	Cytosolic DNA-sensing pathway	16.04447957	1.79035E-18	1.37988E-17	12362	Casp1
04623	Cytosolic DNA-sensing pathway	16.04447957	1.79035E-18	1.37988E-17	16176	Il1b
04623	Cytosolic DNA-sensing pathway	16.04447957	1.79035E-18	1.37988E-17	18035	Nfkbia
04623	Cytosolic DNA-sensing pathway	16.04447957	1.79035E-18	1.37988E-17	230073	Ddx58
04623	Cytosolic DNA-sensing pathway	16.04447957	1.79035E-18	1.37988E-17	54123	Irf7
04623	Cytosolic DNA-sensing pathway	16.04447957	1.79035E-18	1.37988E-17	56489	Ikbke
04623	Cytosolic DNA-sensing pathway	16.04447957	1.79035E-18	1.37988E-17	56480	Tbk1
04623	Cytosolic DNA-sensing pathway	16.04447957	1.79035E-18	1.37988E-17	26388	Ifi202b
05144	Malaria	16.84670355	4.7605E-18	3.58171E-17	80782	Klrb1b
05144	Malaria	16.84670355	4.7605E-18	3.58171E-17	16173	Il18
05144	Malaria	16.84670355	4.7605E-18	3.58171E-17	21926	Tnf
05144	Malaria	16.84670355	4.7605E-18	3.58171E-17	12985	Csf3
05144	Malaria	16.84670355	4.7605E-18	3.58171E-17	16176	Il1b
05144	Malaria	16.84670355	4.7605E-18	3.58171E-17	15894	Icam1
05144	Malaria	16.84670355	4.7605E-18	3.58171E-17	21939	Cd40
05144	Malaria	16.84670355	4.7605E-18	3.58171E-17	20293	Ccl12
05144	Malaria	16.84670355	4.7605E-18	3.58171E-17	16193	Il6
05144	Malaria	16.84670355	4.7605E-18	3.58171E-17	16159	Il12a
05144	Malaria	16.84670355	4.7605E-18	3.58171E-17	17295	Met
05144	Malaria	16.84670355	4.7605E-18	3.58171E-17	16408	Itgal
05144	Malaria	16.84670355	4.7605E-18	3.58171E-17	21825	Thbs1
05144	Malaria	16.84670355	4.7605E-18	3.58171E-17	20969	Sdc1
05144	Malaria	16.84670355	4.7605E-18	3.58171E-17	24088	Tlr2
05144	Malaria	16.84670355	4.7605E-18	3.58171E-17	15978	Ifng
05144	Malaria	16.84670355	4.7605E-18	3.58171E-17	16153	Il10
05144	Malaria	16.84670355	4.7605E-18	3.58171E-17	20296	Ccl2
05144	Malaria	16.84670355	4.7605E-18	3.58171E-17	22329	Vcam1

05162	Measles	9.346458819	6.46079E-18	4.74793E-17	20847	Stat2
05162	Measles	9.346458819	6.46079E-18	4.74793E-17	18033	Nfkb1
05162	Measles	9.346458819	6.46079E-18	4.74793E-17	20846	Stat1
05162	Measles	9.346458819	6.46079E-18	4.74793E-17	71586	Ifih1
05162	Measles	9.346458819	6.46079E-18	4.74793E-17	18035	Nfkbia
05162	Measles	9.346458819	6.46079E-18	4.74793E-17	16176	Il1b
05162	Measles	9.346458819	6.46079E-18	4.74793E-17	14130	Fcgr2b
05162	Measles	9.346458819	6.46079E-18	4.74793E-17	21929	Tnfaip3
05162	Measles	9.346458819	6.46079E-18	4.74793E-17	12444	Ccnd2
05162	Measles	9.346458819	6.46079E-18	4.74793E-17	56489	Ikbke
05162	Measles	9.346458819	6.46079E-18	4.74793E-17	56480	Tbk1
05162	Measles	9.346458819	6.46079E-18	4.74793E-17	16175	Il1a
05162	Measles	9.346458819	6.46079E-18	4.74793E-17	24088	Tlr2
05162	Measles	9.346458819	6.46079E-18	4.74793E-17	18036	Nfkbib
05162	Measles	9.346458819	6.46079E-18	4.74793E-17	18708	Pik3r1
05162	Measles	9.346458819	6.46079E-18	4.74793E-17	17698	Msn
05162	Measles	9.346458819	6.46079E-18	4.74793E-17	23960	Oas1g
05162	Measles	9.346458819	6.46079E-18	4.74793E-17	16160	Il12b
05162	Measles	9.346458819	6.46079E-18	4.74793E-17	54123	Irf7
05162	Measles	9.346458819	6.46079E-18	4.74793E-17	12122	Bid
05162	Measles	9.346458819	6.46079E-18	4.74793E-17	14102	Fas
05162	Measles	9.346458819	6.46079E-18	4.74793E-17	19106	Eif2ak2
05162	Measles	9.346458819	6.46079E-18	4.74793E-17	13063	Cycs
05162	Measles	9.346458819	6.46079E-18	4.74793E-17	230073	Ddx58
05162	Measles	9.346458819	6.46079E-18	4.74793E-17	27218	Slamf1
05162	Measles	9.346458819	6.46079E-18	4.74793E-17	16159	Il12a
05162	Measles	9.346458819	6.46079E-18	4.74793E-17	16193	Il6
05235	PD-L1 expression and PD-1 checkpoint pathway in cancer	12.63502766	9.91663E-18	7.12195E-17	26399	Map2k6
05235	PD-L1 expression and PD-1 checkpoint pathway in cancer	12.63502766	9.91663E-18	7.12195E-17	24088	Tlr2
05235	PD-L1 expression and PD-1 checkpoint pathway in cancer	12.63502766	9.91663E-18	7.12195E-17	53314	Batf
05235	PD-L1 expression and PD-1 checkpoint pathway in cancer	12.63502766	9.91663E-18	7.12195E-17	60533	Cd274
05235	PD-L1 expression and PD-1 checkpoint pathway in cancer	12.63502766	9.91663E-18	7.12195E-17	225471	Ticam2
05235	PD-L1 expression and PD-1 checkpoint pathway in cancer	12.63502766	9.91663E-18	7.12195E-17	74481	Batf2
05235	PD-L1 expression and PD-1 checkpoint pathway in cancer	12.63502766	9.91663E-18	7.12195E-17	16452	Jak2
05235	PD-L1 expression and PD-1 checkpoint pathway in cancer	12.63502766	9.91663E-18	7.12195E-17	16797	Lat
05235	PD-L1 expression and PD-1 checkpoint pathway in cancer	12.63502766	9.91663E-18	7.12195E-17	18035	Nfkbia
05235	PD-L1 expression and PD-1 checkpoint pathway in cancer	12.63502766	9.91663E-18	7.12195E-17	13649	Egfr
05235	PD-L1 expression and PD-1 checkpoint pathway in cancer	12.63502766	9.91663E-18	7.12195E-17	18036	Nfkbib
05235	PD-L1 expression and PD-1 checkpoint pathway in cancer	12.63502766	9.91663E-18	7.12195E-17	15251	Hif1a
05235	PD-L1 expression and PD-1 checkpoint pathway in cancer	12.63502766	9.91663E-18	7.12195E-17	26415	Mapk13
05235	PD-L1 expression and PD-1 checkpoint pathway in cancer	12.63502766	9.91663E-18	7.12195E-17	20846	Stat1
05235	PD-L1 expression and PD-1 checkpoint pathway in cancer	12.63502766	9.91663E-18	7.12195E-17	12503	Cd247

05235	PD-L1 expression and PD-1 checkpoint pathway in cancer	12.63502766	9.91663E-18	7.12195E-17	18037	Nfkbie
05235	PD-L1 expression and PD-1 checkpoint pathway in cancer	12.63502766	9.91663E-18	7.12195E-17	19419	Rasgrp1
05235	PD-L1 expression and PD-1 checkpoint pathway in cancer	12.63502766	9.91663E-18	7.12195E-17	19057	Ppp3cc
05235	PD-L1 expression and PD-1 checkpoint pathway in cancer	12.63502766	9.91663E-18	7.12195E-17	18708	Pik3r1
05235	PD-L1 expression and PD-1 checkpoint pathway in cancer	12.63502766	9.91663E-18	7.12195E-17	56717	Mtor
05235	PD-L1 expression and PD-1 checkpoint pathway in cancer	12.63502766	9.91663E-18	7.12195E-17	18033	Nfkb1
05235	PD-L1 expression and PD-1 checkpoint pathway in cancer	12.63502766	9.91663E-18	7.12195E-17	15978	Ifng
04933	AGE-RAGE signaling pathway in diabetic complications	11.50913411	1.63533E-17	1.14836E-16	17390	Mmp2
04933	AGE-RAGE signaling pathway in diabetic complications	11.50913411	1.63533E-17	1.14836E-16	18798	Plcb4
04933	AGE-RAGE signaling pathway in diabetic complications	11.50913411	1.63533E-17	1.14836E-16	20296	Ccl2
04933	AGE-RAGE signaling pathway in diabetic complications	11.50913411	1.63533E-17	1.14836E-16	16452	Jak2
04933	AGE-RAGE signaling pathway in diabetic complications	11.50913411	1.63533E-17	1.14836E-16	22339	Vegfa
04933	AGE-RAGE signaling pathway in diabetic complications	11.50913411	1.63533E-17	1.14836E-16	13058	Cybb
04933	AGE-RAGE signaling pathway in diabetic complications	11.50913411	1.63533E-17	1.14836E-16	15894	Icam1
04933	AGE-RAGE signaling pathway in diabetic complications	11.50913411	1.63533E-17	1.14836E-16	20293	Ccl12
04933	AGE-RAGE signaling pathway in diabetic complications	11.50913411	1.63533E-17	1.14836E-16	26415	Mapk13
04933	AGE-RAGE signaling pathway in diabetic complications	11.50913411	1.63533E-17	1.14836E-16	22329	Vcam1
04933	AGE-RAGE signaling pathway in diabetic complications	11.50913411	1.63533E-17	1.14836E-16	21926	Tnf
04933	AGE-RAGE signaling pathway in diabetic complications	11.50913411	1.63533E-17	1.14836E-16	18712	Pim1
04933	AGE-RAGE signaling pathway in diabetic complications	11.50913411	1.63533E-17	1.14836E-16	13614	Edn1
04933	AGE-RAGE signaling pathway in diabetic complications	11.50913411	1.63533E-17	1.14836E-16	22341	Vegfc
04933	AGE-RAGE signaling pathway in diabetic complications	11.50913411	1.63533E-17	1.14836E-16	16176	Il1b
04933	AGE-RAGE signaling pathway in diabetic complications	11.50913411	1.63533E-17	1.14836E-16	16193	Il6
04933	AGE-RAGE signaling pathway in diabetic complications	11.50913411	1.63533E-17	1.14836E-16	18708	Pik3r1
04933	AGE-RAGE signaling pathway in diabetic complications	11.50913411	1.63533E-17	1.14836E-16	14066	F3
04933	AGE-RAGE signaling pathway in diabetic complications	11.50913411	1.63533E-17	1.14836E-16	20846	Stat1
04933	AGE-RAGE signaling pathway in diabetic complications	11.50913411	1.63533E-17	1.14836E-16	12827	Col4a2
04933	AGE-RAGE signaling pathway in diabetic complications	11.50913411	1.63533E-17	1.14836E-16	16175	Il1a
04933	AGE-RAGE signaling pathway in diabetic complications	11.50913411	1.63533E-17	1.14836E-16	13653	Egr1
04933	AGE-RAGE signaling pathway in diabetic complications	11.50913411	1.63533E-17	1.14836E-16	18033	Nfkb1
05140	Leishmaniasis	14.44003161	1.8846E-17	1.29464E-16	20846	Stat1
05140	Leishmaniasis	14.44003161	1.8846E-17	1.29464E-16	18033	Nfkb1
05140	Leishmaniasis	14.44003161	1.8846E-17	1.29464E-16	18035	Nfkbia
05140	Leishmaniasis	14.44003161	1.8846E-17	1.29464E-16	16176	Il1b
05140	Leishmaniasis	14.44003161	1.8846E-17	1.29464E-16	17357	Marcksl1
05140	Leishmaniasis	14.44003161	1.8846E-17	1.29464E-16	21926	Tnf
05140	Leishmaniasis	14.44003161	1.8846E-17	1.29464E-16	16159	Il12a
05140	Leishmaniasis	14.44003161	1.8846E-17	1.29464E-16	24088	Tlr2
05140	Leishmaniasis	14.44003161	1.8846E-17	1.29464E-16	16452	Jak2
05140	Leishmaniasis	14.44003161	1.8846E-17	1.29464E-16	26415	Mapk13
05140	Leishmaniasis	14.44003161	1.8846E-17	1.29464E-16	15978	Ifng
05140	Leishmaniasis	14.44003161	1.8846E-17	1.29464E-16	16153	Il10

05140	Leishmaniasis	14.44003161	1.8846E-17	1.29464E-16	15001	H2-Oa
05140	Leishmaniasis	14.44003161	1.8846E-17	1.29464E-16	18126	Nos2
05140	Leishmaniasis	14.44003161	1.8846E-17	1.29464E-16	13058	Cybb
05140	Leishmaniasis	14.44003161	1.8846E-17	1.29464E-16	12266	C3
05140	Leishmaniasis	14.44003161	1.8846E-17	1.29464E-16	16160	Il12b
05140	Leishmaniasis	14.44003161	1.8846E-17	1.29464E-16	19225	Ptgs2
05140	Leishmaniasis	14.44003161	1.8846E-17	1.29464E-16	16175	Il1a
05140	Leishmaniasis	14.44003161	1.8846E-17	1.29464E-16	18036	Nfkbib
04659	Th17 cell differentiation	11.17713986	3.29888E-17	2.21797E-16	16452	Jak2
04659	Th17 cell differentiation	11.17713986	3.29888E-17	2.21797E-16	16190	Il4ra
04659	Th17 cell differentiation	11.17713986	3.29888E-17	2.21797E-16	56717	Mtor
04659	Th17 cell differentiation	11.17713986	3.29888E-17	2.21797E-16	15519	Hsp90aa1
04659	Th17 cell differentiation	11.17713986	3.29888E-17	2.21797E-16	16161	Il12rb1
04659	Th17 cell differentiation	11.17713986	3.29888E-17	2.21797E-16	16176	Il1b
04659	Th17 cell differentiation	11.17713986	3.29888E-17	2.21797E-16	11622	Ahr
04659	Th17 cell differentiation	11.17713986	3.29888E-17	2.21797E-16	209590	Il23r
04659	Th17 cell differentiation	11.17713986	3.29888E-17	2.21797E-16	20846	Stat1
04659	Th17 cell differentiation	11.17713986	3.29888E-17	2.21797E-16	16193	Il6
04659	Th17 cell differentiation	11.17713986	3.29888E-17	2.21797E-16	16177	Il1r1
04659	Th17 cell differentiation	11.17713986	3.29888E-17	2.21797E-16	83430	Il23a
04659	Th17 cell differentiation	11.17713986	3.29888E-17	2.21797E-16	15251	Hif1a
04659	Th17 cell differentiation	11.17713986	3.29888E-17	2.21797E-16	15978	Ifng
04659	Th17 cell differentiation	11.17713986	3.29888E-17	2.21797E-16	15001	H2-Oa
04659	Th17 cell differentiation	11.17713986	3.29888E-17	2.21797E-16	12503	Cd247
04659	Th17 cell differentiation	11.17713986	3.29888E-17	2.21797E-16	18033	Nfkb1
04659	Th17 cell differentiation	11.17713986	3.29888E-17	2.21797E-16	16797	Lat
04659	Th17 cell differentiation	11.17713986	3.29888E-17	2.21797E-16	19057	Ppp3cc
04659	Th17 cell differentiation	11.17713986	3.29888E-17	2.21797E-16	26415	Mapk13
04659	Th17 cell differentiation	11.17713986	3.29888E-17	2.21797E-16	18035	Nfkbia
04659	Th17 cell differentiation	11.17713986	3.29888E-17	2.21797E-16	18036	Nfkbib
04659	Th17 cell differentiation	11.17713986	3.29888E-17	2.21797E-16	18037	Nfkbie
04217	Necroptosis	8.132891369	6.97144E-17	4.58953E-16	225471	Ticam2
04217	Necroptosis	8.132891369	6.97144E-17	4.58953E-16	56532	Ripk3
04217	Necroptosis	8.132891369	6.97144E-17	4.58953E-16	13058	Cybb
04217	Necroptosis	8.132891369	6.97144E-17	4.58953E-16	12362	Casp1
04217	Necroptosis	8.132891369	6.97144E-17	4.58953E-16	20846	Stat1
04217	Necroptosis	8.132891369	6.97144E-17	4.58953E-16	18412	Sqstm1
04217	Necroptosis	8.132891369	6.97144E-17	4.58953E-16	22030	Traf2
04217	Necroptosis	8.132891369	6.97144E-17	4.58953E-16	19106	Eif2ak2
04217	Necroptosis	8.132891369	6.97144E-17	4.58953E-16	15519	Hsp90aa1
04217	Necroptosis	8.132891369	6.97144E-17	4.58953E-16	16176	Il1b
04217	Necroptosis	8.132891369	6.97144E-17	4.58953E-16	108058	Camk2d

04217	Necroptosis	8.132891369	6.97144E-17	4.58953E-16	16175	Il1a
04217	Necroptosis	8.132891369	6.97144E-17	4.58953E-16	216799	Nlrp3
04217	Necroptosis	8.132891369	6.97144E-17	4.58953E-16	21926	Tnf
04217	Necroptosis	8.132891369	6.97144E-17	4.58953E-16	21929	Tnfaip3
04217	Necroptosis	8.132891369	6.97144E-17	4.58953E-16	18783	Pla2g4a
04217	Necroptosis	8.132891369	6.97144E-17	4.58953E-16	74568	Mlkl
04217	Necroptosis	8.132891369	6.97144E-17	4.58953E-16	58203	Zbp1
04217	Necroptosis	8.132891369	6.97144E-17	4.58953E-16	16452	Jak2
04217	Necroptosis	8.132891369	6.97144E-17	4.58953E-16	22033	Traf5
04217	Necroptosis	8.132891369	6.97144E-17	4.58953E-16	15978	Ifng
04217	Necroptosis	8.132891369	6.97144E-17	4.58953E-16	12633	Cflar
04217	Necroptosis	8.132891369	6.97144E-17	4.58953E-16	12122	Bid
04217	Necroptosis	8.132891369	6.97144E-17	4.58953E-16	22035	Tnfsf10
04217	Necroptosis	8.132891369	6.97144E-17	4.58953E-16	14102	Fas
04217	Necroptosis	8.132891369	6.97144E-17	4.58953E-16	20847	Stat2
04217	Necroptosis	8.132891369	6.97144E-17	4.58953E-16	11796	Birc3
04217	Necroptosis	8.132891369	6.97144E-17	4.58953E-16	21933	Tnfrsf10b
05135	Yersinia infection	9.429125121	9.52173E-17	6.14055E-16	12362	Casp1
05135	Yersinia infection	9.429125121	9.52173E-17	6.14055E-16	216799	Nlrp3
05135	Yersinia infection	9.429125121	9.52173E-17	6.14055E-16	26415	Mapk13
05135	Yersinia infection	9.429125121	9.52173E-17	6.14055E-16	18719	Pip5k1b
05135	Yersinia infection	9.429125121	9.52173E-17	6.14055E-16	12927	Bcar1
05135	Yersinia infection	9.429125121	9.52173E-17	6.14055E-16	16153	Il10
05135	Yersinia infection	9.429125121	9.52173E-17	6.14055E-16	18720	Pip5k1a
05135	Yersinia infection	9.429125121	9.52173E-17	6.14055E-16	16402	Itga5
05135	Yersinia infection	9.429125121	9.52173E-17	6.14055E-16	20112	Rps6ka2
05135	Yersinia infection	9.429125121	9.52173E-17	6.14055E-16	54483	Mefv
05135	Yersinia infection	9.429125121	9.52173E-17	6.14055E-16	56480	Tbk1
05135	Yersinia infection	9.429125121	9.52173E-17	6.14055E-16	16176	Il1b
05135	Yersinia infection	9.429125121	9.52173E-17	6.14055E-16	16173	Il18
05135	Yersinia infection	9.429125121	9.52173E-17	6.14055E-16	16797	Lat
05135	Yersinia infection	9.429125121	9.52173E-17	6.14055E-16	18033	Nfkb1
05135	Yersinia infection	9.429125121	9.52173E-17	6.14055E-16	20779	Src
05135	Yersinia infection	9.429125121	9.52173E-17	6.14055E-16	16822	Lcp2
05135	Yersinia infection	9.429125121	9.52173E-17	6.14055E-16	20296	Ccl2
05135	Yersinia infection	9.429125121	9.52173E-17	6.14055E-16	18708	Pik3r1
05135	Yersinia infection	9.429125121	9.52173E-17	6.14055E-16	18035	Nfkbia
05135	Yersinia infection	9.429125121	9.52173E-17	6.14055E-16	22030	Traf2
05135	Yersinia infection	9.429125121	9.52173E-17	6.14055E-16	20293	Ccl12
05135	Yersinia infection	9.429125121	9.52173E-17	6.14055E-16	26399	Map2k6
05135	Yersinia infection	9.429125121	9.52173E-17	6.14055E-16	21926	Tnf
05135	Yersinia infection	9.429125121	9.52173E-17	6.14055E-16	16193	Il6

05161	Hepatitis B	8.37167477	1.2182E-16	7.69904E-16	12575	Cdkn1a
05161	Hepatitis B	8.37167477	1.2182E-16	7.69904E-16	18708	Pik3r1
05161	Hepatitis B	8.37167477	1.2182E-16	7.69904E-16	21926	Tnf
05161	Hepatitis B	8.37167477	1.2182E-16	7.69904E-16	14102	Fas
05161	Hepatitis B	8.37167477	1.2182E-16	7.69904E-16	20779	Src
05161	Hepatitis B	8.37167477	1.2182E-16	7.69904E-16	11911	Atf4
05161	Hepatitis B	8.37167477	1.2182E-16	7.69904E-16	18035	Nfkbia
05161	Hepatitis B	8.37167477	1.2182E-16	7.69904E-16	16452	Jak2
05161	Hepatitis B	8.37167477	1.2182E-16	7.69904E-16	71586	Ifih1
05161	Hepatitis B	8.37167477	1.2182E-16	7.69904E-16	20846	Stat1
05161	Hepatitis B	8.37167477	1.2182E-16	7.69904E-16	231991	Creb5
05161	Hepatitis B	8.37167477	1.2182E-16	7.69904E-16	13063	Cycs
05161	Hepatitis B	8.37167477	1.2182E-16	7.69904E-16	24088	Tlr2
05161	Hepatitis B	8.37167477	1.2182E-16	7.69904E-16	56489	Ikbke
05161	Hepatitis B	8.37167477	1.2182E-16	7.69904E-16	17869	Myc
05161	Hepatitis B	8.37167477	1.2182E-16	7.69904E-16	56480	Tbk1
05161	Hepatitis B	8.37167477	1.2182E-16	7.69904E-16	12122	Bid
05161	Hepatitis B	8.37167477	1.2182E-16	7.69904E-16	16193	Il6
05161	Hepatitis B	8.37167477	1.2182E-16	7.69904E-16	13655	Egr3
05161	Hepatitis B	8.37167477	1.2182E-16	7.69904E-16	17395	Mmp9
05161	Hepatitis B	8.37167477	1.2182E-16	7.69904E-16	225471	Ticam2
05161	Hepatitis B	8.37167477	1.2182E-16	7.69904E-16	20847	Stat2
05161	Hepatitis B	8.37167477	1.2182E-16	7.69904E-16	18033	Nfkb1
05161	Hepatitis B	8.37167477	1.2182E-16	7.69904E-16	26399	Map2k6
05161	Hepatitis B	8.37167477	1.2182E-16	7.69904E-16	230073	Ddx58
05161	Hepatitis B	8.37167477	1.2182E-16	7.69904E-16	54123	Irf7
05161	Hepatitis B	8.37167477	1.2182E-16	7.69904E-16	26415	Mapk13
04014	Ras signaling pathway	6.75320444	3.18253E-16	1.97192E-15	215449	Rap1b
04014	Ras signaling pathway	6.75320444	3.18253E-16	1.97192E-15	16706	Ksr1
04014	Ras signaling pathway	6.75320444	3.18253E-16	1.97192E-15	15566	Htr7
04014	Ras signaling pathway	6.75320444	3.18253E-16	1.97192E-15	114713	Rasa2
04014	Ras signaling pathway	6.75320444	3.18253E-16	1.97192E-15	19696	Rel
04014	Ras signaling pathway	6.75320444	3.18253E-16	1.97192E-15	14701	Gng12
04014	Ras signaling pathway	6.75320444	3.18253E-16	1.97192E-15	16797	Lat
04014	Ras signaling pathway	6.75320444	3.18253E-16	1.97192E-15	17295	Met
04014	Ras signaling pathway	6.75320444	3.18253E-16	1.97192E-15	56480	Tbk1
04014	Ras signaling pathway	6.75320444	3.18253E-16	1.97192E-15	12977	Csf1
04014	Ras signaling pathway	6.75320444	3.18253E-16	1.97192E-15	19417	Rasgrf1
04014	Ras signaling pathway	6.75320444	3.18253E-16	1.97192E-15	18654	Pgf
04014	Ras signaling pathway	6.75320444	3.18253E-16	1.97192E-15	22339	Vegfa
04014	Ras signaling pathway	6.75320444	3.18253E-16	1.97192E-15	11600	Angpt1
04014	Ras signaling pathway	6.75320444	3.18253E-16	1.97192E-15	18049	Ngf

04014	Ras signaling pathway	6.75320444	3.18253E-16	1.97192E-15	14164	Fgf1
04014	Ras signaling pathway	6.75320444	3.18253E-16	1.97192E-15	19731	Rgl1
04014	Ras signaling pathway	6.75320444	3.18253E-16	1.97192E-15	18708	Pik3r1
04014	Ras signaling pathway	6.75320444	3.18253E-16	1.97192E-15	22341	Vegfc
04014	Ras signaling pathway	6.75320444	3.18253E-16	1.97192E-15	19419	Rasgrp1
04014	Ras signaling pathway	6.75320444	3.18253E-16	1.97192E-15	85031	Pla1a
04014	Ras signaling pathway	6.75320444	3.18253E-16	1.97192E-15	19730	Ralgds
04014	Ras signaling pathway	6.75320444	3.18253E-16	1.97192E-15	13640	Efna5
04014	Ras signaling pathway	6.75320444	3.18253E-16	1.97192E-15	23872	Ets2
04014	Ras signaling pathway	6.75320444	3.18253E-16	1.97192E-15	18784	Pla2g5
04014	Ras signaling pathway	6.75320444	3.18253E-16	1.97192E-15	18783	Pla2g4a
04014	Ras signaling pathway	6.75320444	3.18253E-16	1.97192E-15	13637	Efna2
04014	Ras signaling pathway	6.75320444	3.18253E-16	1.97192E-15	23871	Ets1
04014	Ras signaling pathway	6.75320444	3.18253E-16	1.97192E-15	13649	Egfr
04014	Ras signaling pathway	6.75320444	3.18253E-16	1.97192E-15	14254	Flt1
04014	Ras signaling pathway	6.75320444	3.18253E-16	1.97192E-15	18033	Nfkb1
05321	Inflammatory bowel disease	14.67293535	6.33156E-16	3.84764E-15	209590	Il23r
05321	Inflammatory bowel disease	14.67293535	6.33156E-16	3.84764E-15	16190	Il4ra
05321	Inflammatory bowel disease	14.67293535	6.33156E-16	3.84764E-15	16159	Il12a
05321	Inflammatory bowel disease	14.67293535	6.33156E-16	3.84764E-15	16175	Il1a
05321	Inflammatory bowel disease	14.67293535	6.33156E-16	3.84764E-15	16173	Il18
05321	Inflammatory bowel disease	14.67293535	6.33156E-16	3.84764E-15	20846	Stat1
05321	Inflammatory bowel disease	14.67293535	6.33156E-16	3.84764E-15	18033	Nfkb1
05321	Inflammatory bowel disease	14.67293535	6.33156E-16	3.84764E-15	15001	H2-Oa
05321	Inflammatory bowel disease	14.67293535	6.33156E-16	3.84764E-15	16161	Il12rb1
05321	Inflammatory bowel disease	14.67293535	6.33156E-16	3.84764E-15	16153	Il10
05321	Inflammatory bowel disease	14.67293535	6.33156E-16	3.84764E-15	24088	Tlr2
05321	Inflammatory bowel disease	14.67293535	6.33156E-16	3.84764E-15	257632	Nod2
05321	Inflammatory bowel disease	14.67293535	6.33156E-16	3.84764E-15	16193	Il6
05321	Inflammatory bowel disease	14.67293535	6.33156E-16	3.84764E-15	16176	Il1b
05321	Inflammatory bowel disease	14.67293535	6.33156E-16	3.84764E-15	16160	Il12b
05321	Inflammatory bowel disease	14.67293535	6.33156E-16	3.84764E-15	21926	Tnf
05321	Inflammatory bowel disease	14.67293535	6.33156E-16	3.84764E-15	83430	Il23a
05321	Inflammatory bowel disease	14.67293535	6.33156E-16	3.84764E-15	15978	Ifng
05010	Alzheimer disease	5.204672641	7.96759E-16	4.75049E-15	12122	Bid
05010	Alzheimer disease	5.204672641	7.96759E-16	4.75049E-15	12369	Casp7
05010	Alzheimer disease	5.204672641	7.96759E-16	4.75049E-15	14433	Gapdh
05010	Alzheimer disease	5.204672641	7.96759E-16	4.75049E-15	19225	Ptgs2
05010	Alzheimer disease	5.204672641	7.96759E-16	4.75049E-15	14102	Fas
05010	Alzheimer disease	5.204672641	7.96759E-16	4.75049E-15	18798	Plcb4
05010	Alzheimer disease	5.204672641	7.96759E-16	4.75049E-15	59029	Psmd14
05010	Alzheimer disease	5.204672641	7.96759E-16	4.75049E-15	19175	Psmb6

05010	Alzheimer disease	5.204672641	7.96759E-16	4.75049E-15	19057	Ppp3cc
05010	Alzheimer disease	5.204672641	7.96759E-16	4.75049E-15	26442	Psma5
05010	Alzheimer disease	5.204672641	7.96759E-16	4.75049E-15	11911	Atf4
05010	Alzheimer disease	5.204672641	7.96759E-16	4.75049E-15	13058	Cybb
05010	Alzheimer disease	5.204672641	7.96759E-16	4.75049E-15	12977	Csf1
05010	Alzheimer disease	5.204672641	7.96759E-16	4.75049E-15	18708	Pik3r1
05010	Alzheimer disease	5.204672641	7.96759E-16	4.75049E-15	19106	Eif2ak2
05010	Alzheimer disease	5.204672641	7.96759E-16	4.75049E-15	69077	Psmd11
05010	Alzheimer disease	5.204672641	7.96759E-16	4.75049E-15	56436	Adrm1
05010	Alzheimer disease	5.204672641	7.96759E-16	4.75049E-15	19181	Psmc2
05010	Alzheimer disease	5.204672641	7.96759E-16	4.75049E-15	14814	Grin2d
05010	Alzheimer disease	5.204672641	7.96759E-16	4.75049E-15	18126	Nos2
05010	Alzheimer disease	5.204672641	7.96759E-16	4.75049E-15	14362	Fzd1
05010	Alzheimer disease	5.204672641	7.96759E-16	4.75049E-15	22145	Tuba4a
05010	Alzheimer disease	5.204672641	7.96759E-16	4.75049E-15	22030	Traf2
05010	Alzheimer disease	5.204672641	7.96759E-16	4.75049E-15	16176	Il1b
05010	Alzheimer disease	5.204672641	7.96759E-16	4.75049E-15	19166	Psma2
05010	Alzheimer disease	5.204672641	7.96759E-16	4.75049E-15	22420	Wnt6
05010	Alzheimer disease	5.204672641	7.96759E-16	4.75049E-15	26443	Psma6
05010	Alzheimer disease	5.204672641	7.96759E-16	4.75049E-15	19173	Psmb5
05010	Alzheimer disease	5.204672641	7.96759E-16	4.75049E-15	18033	Nfkb1
05010	Alzheimer disease	5.204672641	7.96759E-16	4.75049E-15	16193	Il6
05010	Alzheimer disease	5.204672641	7.96759E-16	4.75049E-15	56717	Mtor
05010	Alzheimer disease	5.204672641	7.96759E-16	4.75049E-15	20617	Snca
05010	Alzheimer disease	5.204672641	7.96759E-16	4.75049E-15	22142	Tuba1a
05010	Alzheimer disease	5.204672641	7.96759E-16	4.75049E-15	13063	Cycs
05010	Alzheimer disease	5.204672641	7.96759E-16	4.75049E-15	22153	Tubb4a
05010	Alzheimer disease	5.204672641	7.96759E-16	4.75049E-15	16175	Il1a
05010	Alzheimer disease	5.204672641	7.96759E-16	4.75049E-15	21926	Tnf
05010	Alzheimer disease	5.204672641	7.96759E-16	4.75049E-15	11491	Adam17
05203	Viral carcinogenesis	6.620975194	1.72713E-15	1.01069E-14	20259	Scin
05203	Viral carcinogenesis	6.620975194	1.72713E-15	1.01069E-14	114713	Rasa2
05203	Viral carcinogenesis	6.620975194	1.72713E-15	1.01069E-14	18035	Nfkbia
05203	Viral carcinogenesis	6.620975194	1.72713E-15	1.01069E-14	12575	Cdkn1a
05203	Viral carcinogenesis	6.620975194	1.72713E-15	1.01069E-14	15039	H2-T22
05203	Viral carcinogenesis	6.620975194	1.72713E-15	1.01069E-14	12266	C3
05203	Viral carcinogenesis	6.620975194	1.72713E-15	1.01069E-14	19696	Rel
05203	Viral carcinogenesis	6.620975194	1.72713E-15	1.01069E-14	110558	H2-Q9
05203	Viral carcinogenesis	6.620975194	1.72713E-15	1.01069E-14	20963	Syk
05203	Viral carcinogenesis	6.620975194	1.72713E-15	1.01069E-14	18033	Nfkb1
05203	Viral carcinogenesis	6.620975194	1.72713E-15	1.01069E-14	19106	Eif2ak2
05203	Viral carcinogenesis	6.620975194	1.72713E-15	1.01069E-14	231991	Creb5

05203	Viral carcinogenesis	6.620975194	1.72713E-15	1.01069E-14	11911	Atf4
05203	Viral carcinogenesis	6.620975194	1.72713E-15	1.01069E-14	66973	Mrps18b
05203	Viral carcinogenesis	6.620975194	1.72713E-15	1.01069E-14	15018	H2-Q7
05203	Viral carcinogenesis	6.620975194	1.72713E-15	1.01069E-14	14990	H2-M2
05203	Viral carcinogenesis	6.620975194	1.72713E-15	1.01069E-14	12444	Ccnd2
05203	Viral carcinogenesis	6.620975194	1.72713E-15	1.01069E-14	18034	Nfkb2
05203	Viral carcinogenesis	6.620975194	1.72713E-15	1.01069E-14	17164	Mapkapk2
05203	Viral carcinogenesis	6.620975194	1.72713E-15	1.01069E-14	20779	Src
05203	Viral carcinogenesis	6.620975194	1.72713E-15	1.01069E-14	18708	Pik3r1
05203	Viral carcinogenesis	6.620975194	1.72713E-15	1.01069E-14	68153	Gtf2e2
05203	Viral carcinogenesis	6.620975194	1.72713E-15	1.01069E-14	17096	Lyn
05203	Viral carcinogenesis	6.620975194	1.72713E-15	1.01069E-14	12579	Cdkn2b
05203	Viral carcinogenesis	6.620975194	1.72713E-15	1.01069E-14	22030	Traf2
05203	Viral carcinogenesis	6.620975194	1.72713E-15	1.01069E-14	54123	Irf7
05203	Viral carcinogenesis	6.620975194	1.72713E-15	1.01069E-14	13655	Egr3
05203	Viral carcinogenesis	6.620975194	1.72713E-15	1.01069E-14	22033	Traf5
05203	Viral carcinogenesis	6.620975194	1.72713E-15	1.01069E-14	22029	Traf1
05203	Viral carcinogenesis	6.620975194	1.72713E-15	1.01069E-14	15024	H2-T10
01230	Biosynthesis of amino acids	12.15521649	4.37788E-15	2.51529E-14	27053	Asns
01230	Biosynthesis of amino acids	12.15521649	4.37788E-15	2.51529E-14	109652	Acy1
01230	Biosynthesis of amino acids	12.15521649	4.37788E-15	2.51529E-14	11846	Arg1
01230	Biosynthesis of amino acids	12.15521649	4.37788E-15	2.51529E-14	18563	Pcx
01230	Biosynthesis of amino acids	12.15521649	4.37788E-15	2.51529E-14	217214	Nags
01230	Biosynthesis of amino acids	12.15521649	4.37788E-15	2.51529E-14	236539	Phgdh
01230	Biosynthesis of amino acids	12.15521649	4.37788E-15	2.51529E-14	12035	Bcat1
01230	Biosynthesis of amino acids	12.15521649	4.37788E-15	2.51529E-14	11898	Ass1
01230	Biosynthesis of amino acids	12.15521649	4.37788E-15	2.51529E-14	71776	Tha1
01230	Biosynthesis of amino acids	12.15521649	4.37788E-15	2.51529E-14	107272	Psat1
01230	Biosynthesis of amino acids	12.15521649	4.37788E-15	2.51529E-14	18641	Pfkl
01230	Biosynthesis of amino acids	12.15521649	4.37788E-15	2.51529E-14	11847	Arg2
01230	Biosynthesis of amino acids	12.15521649	4.37788E-15	2.51529E-14	107869	Cth
01230	Biosynthesis of amino acids	12.15521649	4.37788E-15	2.51529E-14	18648	Pgam1
01230	Biosynthesis of amino acids	12.15521649	4.37788E-15	2.51529E-14	13806	Eno1
01230	Biosynthesis of amino acids	12.15521649	4.37788E-15	2.51529E-14	433182	Eno1b
01230	Biosynthesis of amino acids	12.15521649	4.37788E-15	2.51529E-14	21991	Tpi1
01230	Biosynthesis of amino acids	12.15521649	4.37788E-15	2.51529E-14	14433	Gapdh
01230	Biosynthesis of amino acids	12.15521649	4.37788E-15	2.51529E-14	18655	Pgk1
04920	Adipocytokine signaling pathway	12.8129858	8.99599E-15	5.07631E-14	21938	Tnfrsf1b
04920	Adipocytokine signaling pathway	12.8129858	8.99599E-15	5.07631E-14	433256	Acsl5
04920	Adipocytokine signaling pathway	12.8129858	8.99599E-15	5.07631E-14	18035	Nfkbia
04920	Adipocytokine signaling pathway	12.8129858	8.99599E-15	5.07631E-14	20525	Slc2a1
04920	Adipocytokine signaling pathway	12.8129858	8.99599E-15	5.07631E-14	14081	Acsl1

04920	Adipocytokine signaling pathway	12.8129858	8.99599E-15	5.07631E-14	105787	Prkaa1
04920	Adipocytokine signaling pathway	12.8129858	8.99599E-15	5.07631E-14	12702	Socs3
04920	Adipocytokine signaling pathway	12.8129858	8.99599E-15	5.07631E-14	56717	Mtor
04920	Adipocytokine signaling pathway	12.8129858	8.99599E-15	5.07631E-14	18037	Nfkbie
04920	Adipocytokine signaling pathway	12.8129858	8.99599E-15	5.07631E-14	108079	Prkaa2
04920	Adipocytokine signaling pathway	12.8129858	8.99599E-15	5.07631E-14	22030	Traf2
04920	Adipocytokine signaling pathway	12.8129858	8.99599E-15	5.07631E-14	18033	Nfkb1
04920	Adipocytokine signaling pathway	12.8129858	8.99599E-15	5.07631E-14	18036	Nfkbib
04920	Adipocytokine signaling pathway	12.8129858	8.99599E-15	5.07631E-14	16452	Jak2
04920	Adipocytokine signaling pathway	12.8129858	8.99599E-15	5.07631E-14	94180	Acsbg1
04920	Adipocytokine signaling pathway	12.8129858	8.99599E-15	5.07631E-14	16847	Lepr
04920	Adipocytokine signaling pathway	12.8129858	8.99599E-15	5.07631E-14	50790	Acsl4
04920	Adipocytokine signaling pathway	12.8129858	8.99599E-15	5.07631E-14	21926	Tnf
04145	Phagosome	7.220015807	1.88082E-14	1.0427E-13	20288	Msr1
04145	Phagosome	7.220015807	1.88082E-14	1.0427E-13	15018	H2-Q7
04145	Phagosome	7.220015807	1.88082E-14	1.0427E-13	11973	Atp6v1e1
04145	Phagosome	7.220015807	1.88082E-14	1.0427E-13	110558	H2-Q9
04145	Phagosome	7.220015807	1.88082E-14	1.0427E-13	12266	C3
04145	Phagosome	7.220015807	1.88082E-14	1.0427E-13	15001	H2-Oa
04145	Phagosome	7.220015807	1.88082E-14	1.0427E-13	21825	Thbs1
04145	Phagosome	7.220015807	1.88082E-14	1.0427E-13	12475	Cd14
04145	Phagosome	7.220015807	1.88082E-14	1.0427E-13	15024	H2-T10
04145	Phagosome	7.220015807	1.88082E-14	1.0427E-13	108078	Olr1
04145	Phagosome	7.220015807	1.88082E-14	1.0427E-13	17167	Marco
04145	Phagosome	7.220015807	1.88082E-14	1.0427E-13	21899	Tlr6
04145	Phagosome	7.220015807	1.88082E-14	1.0427E-13	22153	Tubb4a
04145	Phagosome	7.220015807	1.88082E-14	1.0427E-13	14130	Fcgr2b
04145	Phagosome	7.220015807	1.88082E-14	1.0427E-13	16402	Itga5
04145	Phagosome	7.220015807	1.88082E-14	1.0427E-13	216238	Eea1
04145	Phagosome	7.220015807	1.88082E-14	1.0427E-13	14990	H2-M2
04145	Phagosome	7.220015807	1.88082E-14	1.0427E-13	22145	Tuba4a
04145	Phagosome	7.220015807	1.88082E-14	1.0427E-13	17113	M6pr
04145	Phagosome	7.220015807	1.88082E-14	1.0427E-13	24088	Tlr2
04145	Phagosome	7.220015807	1.88082E-14	1.0427E-13	667277	C1rb
04145	Phagosome	7.220015807	1.88082E-14	1.0427E-13	12317	Calr
04145	Phagosome	7.220015807	1.88082E-14	1.0427E-13	20335	Sec61g
04145	Phagosome	7.220015807	1.88082E-14	1.0427E-13	15039	H2-T22
04145	Phagosome	7.220015807	1.88082E-14	1.0427E-13	22142	Tuba1a
04145	Phagosome	7.220015807	1.88082E-14	1.0427E-13	13058	Cybb
01240	Biosynthesis of cofactors	7.876380881	2.85765E-14	1.55693E-13	12035	Bcat1
01240	Biosynthesis of cofactors	7.876380881	2.85765E-14	1.55693E-13	107272	Psat1
01240	Biosynthesis of cofactors	7.876380881	2.85765E-14	1.55693E-13	22169	Cmpk2

01240	Biosynthesis of cofactors	7.876380881	2.85765E-14	1.55693E-13	209176	Ido2
01240	Biosynthesis of cofactors	7.876380881	2.85765E-14	1.55693E-13	67417	Ears2
01240	Biosynthesis of cofactors	7.876380881	2.85765E-14	1.55693E-13	14854	Gss
01240	Biosynthesis of cofactors	7.876380881	2.85765E-14	1.55693E-13	11639	Ak4
01240	Biosynthesis of cofactors	7.876380881	2.85765E-14	1.55693E-13	11637	Ak2
01240	Biosynthesis of cofactors	7.876380881	2.85765E-14	1.55693E-13	15930	Ido1
01240	Biosynthesis of cofactors	7.876380881	2.85765E-14	1.55693E-13	665563	Mthfd2l
01240	Biosynthesis of cofactors	7.876380881	2.85765E-14	1.55693E-13	12850	Coq7
01240	Biosynthesis of cofactors	7.876380881	2.85765E-14	1.55693E-13	102216272	Ak6
01240	Biosynthesis of cofactors	7.876380881	2.85765E-14	1.55693E-13	56738	Mocs1
01240	Biosynthesis of cofactors	7.876380881	2.85765E-14	1.55693E-13	270685	Mthfd1l
01240	Biosynthesis of cofactors	7.876380881	2.85765E-14	1.55693E-13	17768	Mthfd2
01240	Biosynthesis of cofactors	7.876380881	2.85765E-14	1.55693E-13	77974	Rdh12
01240	Biosynthesis of cofactors	7.876380881	2.85765E-14	1.55693E-13	18104	Nqo1
01240	Biosynthesis of cofactors	7.876380881	2.85765E-14	1.55693E-13	14629	Gclc
01240	Biosynthesis of cofactors	7.876380881	2.85765E-14	1.55693E-13	54369	Nme6
01240	Biosynthesis of cofactors	7.876380881	2.85765E-14	1.55693E-13	14528	Gch1
01240	Biosynthesis of cofactors	7.876380881	2.85765E-14	1.55693E-13	192185	Nadk
01240	Biosynthesis of cofactors	7.876380881	2.85765E-14	1.55693E-13	14630	Gclm
01240	Biosynthesis of cofactors	7.876380881	2.85765E-14	1.55693E-13	11655	Alas1
01240	Biosynthesis of cofactors	7.876380881	2.85765E-14	1.55693E-13	268566	Gphn
04810	Regulation of actin cytoskeleton	6.432377719	3.44134E-14	1.84316E-13	18708	Pik3r1
04810	Regulation of actin cytoskeleton	6.432377719	3.44134E-14	1.84316E-13	109700	Itga1
04810	Regulation of actin cytoskeleton	6.432377719	3.44134E-14	1.84316E-13	14062	F2r
04810	Regulation of actin cytoskeleton	6.432377719	3.44134E-14	1.84316E-13	104099	Itga9
04810	Regulation of actin cytoskeleton	6.432377719	3.44134E-14	1.84316E-13	12927	Bcar1
04810	Regulation of actin cytoskeleton	6.432377719	3.44134E-14	1.84316E-13	22350	Ezr
04810	Regulation of actin cytoskeleton	6.432377719	3.44134E-14	1.84316E-13	14701	Gng12
04810	Regulation of actin cytoskeleton	6.432377719	3.44134E-14	1.84316E-13	14164	Fgf1
04810	Regulation of actin cytoskeleton	6.432377719	3.44134E-14	1.84316E-13	228785	Mylk2
04810	Regulation of actin cytoskeleton	6.432377719	3.44134E-14	1.84316E-13	219140	Spata13
04810	Regulation of actin cytoskeleton	6.432377719	3.44134E-14	1.84316E-13	14745	Lpar1
04810	Regulation of actin cytoskeleton	6.432377719	3.44134E-14	1.84316E-13	13649	Egfr
04810	Regulation of actin cytoskeleton	6.432377719	3.44134E-14	1.84316E-13	22330	Vcl
04810	Regulation of actin cytoskeleton	6.432377719	3.44134E-14	1.84316E-13	17698	Msn
04810	Regulation of actin cytoskeleton	6.432377719	3.44134E-14	1.84316E-13	17880	Myh11
04810	Regulation of actin cytoskeleton	6.432377719	3.44134E-14	1.84316E-13	320910	Itgb8
04810	Regulation of actin cytoskeleton	6.432377719	3.44134E-14	1.84316E-13	329251	Ppp1r12b
04810	Regulation of actin cytoskeleton	6.432377719	3.44134E-14	1.84316E-13	50884	Nckap1
04810	Regulation of actin cytoskeleton	6.432377719	3.44134E-14	1.84316E-13	18719	Pip5k1b
04810	Regulation of actin cytoskeleton	6.432377719	3.44134E-14	1.84316E-13	20259	Scin
04810	Regulation of actin cytoskeleton	6.432377719	3.44134E-14	1.84316E-13	319480	Itga11

04810	Regulation of actin cytoskeleton	6.432377719	3.44134E-14	1.84316E-13	18720	Pip5k1a
04810	Regulation of actin cytoskeleton	6.432377719	3.44134E-14	1.84316E-13	16402	Itga5
04810	Regulation of actin cytoskeleton	6.432377719	3.44134E-14	1.84316E-13	76884	Cyfip2
04810	Regulation of actin cytoskeleton	6.432377719	3.44134E-14	1.84316E-13	18645	Pfn2
04810	Regulation of actin cytoskeleton	6.432377719	3.44134E-14	1.84316E-13	20779	Src
04810	Regulation of actin cytoskeleton	6.432377719	3.44134E-14	1.84316E-13	12062	Bdkrb2
04810	Regulation of actin cytoskeleton	6.432377719	3.44134E-14	1.84316E-13	16408	Itgal
04658	Th1 and Th2 cell differentiation	10.91206934	3.7305E-14	1.96473E-13	16797	Lat
04658	Th1 and Th2 cell differentiation	10.91206934	3.7305E-14	1.96473E-13	19057	Ppp3cc
04658	Th1 and Th2 cell differentiation	10.91206934	3.7305E-14	1.96473E-13	16190	Il4ra
04658	Th1 and Th2 cell differentiation	10.91206934	3.7305E-14	1.96473E-13	20846	Stat1
04658	Th1 and Th2 cell differentiation	10.91206934	3.7305E-14	1.96473E-13	26415	Mapk13
04658	Th1 and Th2 cell differentiation	10.91206934	3.7305E-14	1.96473E-13	18033	Nfkb1
04658	Th1 and Th2 cell differentiation	10.91206934	3.7305E-14	1.96473E-13	18035	Nfkbia
04658	Th1 and Th2 cell differentiation	10.91206934	3.7305E-14	1.96473E-13	16449	Jag1
04658	Th1 and Th2 cell differentiation	10.91206934	3.7305E-14	1.96473E-13	15978	Ifng
04658	Th1 and Th2 cell differentiation	10.91206934	3.7305E-14	1.96473E-13	18036	Nfkbib
04658	Th1 and Th2 cell differentiation	10.91206934	3.7305E-14	1.96473E-13	18128	Notch1
04658	Th1 and Th2 cell differentiation	10.91206934	3.7305E-14	1.96473E-13	12503	Cd247
04658	Th1 and Th2 cell differentiation	10.91206934	3.7305E-14	1.96473E-13	16161	Il12rb1
04658	Th1 and Th2 cell differentiation	10.91206934	3.7305E-14	1.96473E-13	16159	Il12a
04658	Th1 and Th2 cell differentiation	10.91206934	3.7305E-14	1.96473E-13	16452	Jak2
04658	Th1 and Th2 cell differentiation	10.91206934	3.7305E-14	1.96473E-13	18037	Nfkbie
04658	Th1 and Th2 cell differentiation	10.91206934	3.7305E-14	1.96473E-13	12399	Runx3
04658	Th1 and Th2 cell differentiation	10.91206934	3.7305E-14	1.96473E-13	16160	Il12b
04658	Th1 and Th2 cell differentiation	10.91206934	3.7305E-14	1.96473E-13	15001	H2-Oa
04020	Calcium signaling pathway	6.106930037	4.54499E-14	2.31648E-13	94045	P2rx5
04020	Calcium signaling pathway	6.106930037	4.54499E-14	2.31648E-13	14829	Grpr
04020	Calcium signaling pathway	6.106930037	4.54499E-14	2.31648E-13	20698	Sphk1
04020	Calcium signaling pathway	6.106930037	4.54499E-14	2.31648E-13	13649	Egfr
04020	Calcium signaling pathway	6.106930037	4.54499E-14	2.31648E-13	14062	F2r
04020	Calcium signaling pathway	6.106930037	4.54499E-14	2.31648E-13	13618	Ednrb
04020	Calcium signaling pathway	6.106930037	4.54499E-14	2.31648E-13	14814	Grin2d
04020	Calcium signaling pathway	6.106930037	4.54499E-14	2.31648E-13	17295	Met
04020	Calcium signaling pathway	6.106930037	4.54499E-14	2.31648E-13	14254	Flt1
04020	Calcium signaling pathway	6.106930037	4.54499E-14	2.31648E-13	12494	Cd38
04020	Calcium signaling pathway	6.106930037	4.54499E-14	2.31648E-13	15464	Hrc
04020	Calcium signaling pathway	6.106930037	4.54499E-14	2.31648E-13	58861	Cysltr1
04020	Calcium signaling pathway	6.106930037	4.54499E-14	2.31648E-13	68279	Mcoln2
04020	Calcium signaling pathway	6.106930037	4.54499E-14	2.31648E-13	11540	Adora2a
04020	Calcium signaling pathway	6.106930037	4.54499E-14	2.31648E-13	381290	Atp2b4
04020	Calcium signaling pathway	6.106930037	4.54499E-14	2.31648E-13	269717	Orai2

04020	Calcium signaling pathway	6.106930037	4.54499E-14	2.31648E-13	228785	Mylk2
04020	Calcium signaling pathway	6.106930037	4.54499E-14	2.31648E-13	19057	Ppp3cc
04020	Calcium signaling pathway	6.106930037	4.54499E-14	2.31648E-13	108058	Camk2d
04020	Calcium signaling pathway	6.106930037	4.54499E-14	2.31648E-13	22341	Vegfc
04020	Calcium signaling pathway	6.106930037	4.54499E-14	2.31648E-13	15566	Htr7
04020	Calcium signaling pathway	6.106930037	4.54499E-14	2.31648E-13	12062	Bdkrb2
04020	Calcium signaling pathway	6.106930037	4.54499E-14	2.31648E-13	14164	Fgf1
04020	Calcium signaling pathway	6.106930037	4.54499E-14	2.31648E-13	18798	Plcb4
04020	Calcium signaling pathway	6.106930037	4.54499E-14	2.31648E-13	11541	Adora2b
04020	Calcium signaling pathway	6.106930037	4.54499E-14	2.31648E-13	18126	Nos2
04020	Calcium signaling pathway	6.106930037	4.54499E-14	2.31648E-13	15466	Hrh2
04020	Calcium signaling pathway	6.106930037	4.54499E-14	2.31648E-13	22339	Vegfa
04020	Calcium signaling pathway	6.106930037	4.54499E-14	2.31648E-13	18049	Ngf
05170	Human immunodeficiency virus 1 infection	6.106930037	4.54499E-14	2.31648E-13	24088	Tlr2
05170	Human immunodeficiency virus 1 infection	6.106930037	4.54499E-14	2.31648E-13	209387	Trim30d
05170	Human immunodeficiency virus 1 infection	6.106930037	4.54499E-14	2.31648E-13	18035	Nfkbia
05170	Human immunodeficiency virus 1 infection	6.106930037	4.54499E-14	2.31648E-13	214763	Cgas
05170	Human immunodeficiency virus 1 infection	6.106930037	4.54499E-14	2.31648E-13	26415	Mapk13
05170	Human immunodeficiency virus 1 infection	6.106930037	4.54499E-14	2.31648E-13	26399	Map2k6
05170	Human immunodeficiency virus 1 infection	6.106930037	4.54499E-14	2.31648E-13	15024	H2-T10
05170	Human immunodeficiency virus 1 infection	6.106930037	4.54499E-14	2.31648E-13	13063	Cycs
05170	Human immunodeficiency virus 1 infection	6.106930037	4.54499E-14	2.31648E-13	12503	Cd247
05170	Human immunodeficiency virus 1 infection	6.106930037	4.54499E-14	2.31648E-13	69550	Bst2
05170	Human immunodeficiency virus 1 infection	6.106930037	4.54499E-14	2.31648E-13	56480	Tbk1
05170	Human immunodeficiency virus 1 infection	6.106930037	4.54499E-14	2.31648E-13	22030	Traf2
05170	Human immunodeficiency virus 1 infection	6.106930037	4.54499E-14	2.31648E-13	15018	H2-Q7
05170	Human immunodeficiency virus 1 infection	6.106930037	4.54499E-14	2.31648E-13	321006	Dcaf1
05170	Human immunodeficiency virus 1 infection	6.106930037	4.54499E-14	2.31648E-13	67923	Eloc
05170	Human immunodeficiency virus 1 infection	6.106930037	4.54499E-14	2.31648E-13	22033	Traf5
05170	Human immunodeficiency virus 1 infection	6.106930037	4.54499E-14	2.31648E-13	110558	H2-Q9
05170	Human immunodeficiency virus 1 infection	6.106930037	4.54499E-14	2.31648E-13	14990	H2-M2
05170	Human immunodeficiency virus 1 infection	6.106930037	4.54499E-14	2.31648E-13	21938	Tnfrsf1b
05170	Human immunodeficiency virus 1 infection	6.106930037	4.54499E-14	2.31648E-13	14701	Gng12
05170	Human immunodeficiency virus 1 infection	6.106930037	4.54499E-14	2.31648E-13	14102	Fas
05170	Human immunodeficiency virus 1 infection	6.106930037	4.54499E-14	2.31648E-13	21926	Tnf
05170	Human immunodeficiency virus 1 infection	6.106930037	4.54499E-14	2.31648E-13	18708	Pik3r1
05170	Human immunodeficiency virus 1 infection	6.106930037	4.54499E-14	2.31648E-13	12317	Calr
05170	Human immunodeficiency virus 1 infection	6.106930037	4.54499E-14	2.31648E-13	56717	Mtor
05170	Human immunodeficiency virus 1 infection	6.106930037	4.54499E-14	2.31648E-13	19057	Ppp3cc
05170	Human immunodeficiency virus 1 infection	6.106930037	4.54499E-14	2.31648E-13	15039	H2-T22
05170	Human immunodeficiency virus 1 infection	6.106930037	4.54499E-14	2.31648E-13	18033	Nfkb1
05170	Human immunodeficiency virus 1 infection	6.106930037	4.54499E-14	2.31648E-13	12122	Bid

05143	African trypanosomiasis	18.14260382	5.0413E-14	2.52865E-13	21926	Tnf
05143	African trypanosomiasis	18.14260382	5.0413E-14	2.52865E-13	16176	Il1b
05143	African trypanosomiasis	18.14260382	5.0413E-14	2.52865E-13	22329	Vcam1
05143	African trypanosomiasis	18.14260382	5.0413E-14	2.52865E-13	16193	Il6
05143	African trypanosomiasis	18.14260382	5.0413E-14	2.52865E-13	14102	Fas
05143	African trypanosomiasis	18.14260382	5.0413E-14	2.52865E-13	16173	Il18
05143	African trypanosomiasis	18.14260382	5.0413E-14	2.52865E-13	16153	Il10
05143	African trypanosomiasis	18.14260382	5.0413E-14	2.52865E-13	16160	Il12b
05143	African trypanosomiasis	18.14260382	5.0413E-14	2.52865E-13	16159	Il12a
05143	African trypanosomiasis	18.14260382	5.0413E-14	2.52865E-13	18798	Plcb4
05143	African trypanosomiasis	18.14260382	5.0413E-14	2.52865E-13	15978	Ifng
05143	African trypanosomiasis	18.14260382	5.0413E-14	2.52865E-13	209176	Ido2
05143	African trypanosomiasis	18.14260382	5.0413E-14	2.52865E-13	15894	Icam1
05143	African trypanosomiasis	18.14260382	5.0413E-14	2.52865E-13	15930	Ido1
05206	MicroRNAs in cancer	5.337569442	9.1078E-14	4.49698E-13	21923	Tnc
05206	MicroRNAs in cancer	5.337569442	9.1078E-14	4.49698E-13	16402	Itga5
05206	MicroRNAs in cancer	5.337569442	9.1078E-14	4.49698E-13	18712	Pim1
05206	MicroRNAs in cancer	5.337569442	9.1078E-14	4.49698E-13	17118	Marcks
05206	MicroRNAs in cancer	5.337569442	9.1078E-14	4.49698E-13	12444	Ccnd2
05206	MicroRNAs in cancer	5.337569442	9.1078E-14	4.49698E-13	17395	Mmp9
05206	MicroRNAs in cancer	5.337569442	9.1078E-14	4.49698E-13	13649	Egfr
05206	MicroRNAs in cancer	5.337569442	9.1078E-14	4.49698E-13	329278	Tnn
05206	MicroRNAs in cancer	5.337569442	9.1078E-14	4.49698E-13	21417	Zeb1
05206	MicroRNAs in cancer	5.337569442	9.1078E-14	4.49698E-13	12575	Cdkn1a
05206	MicroRNAs in cancer	5.337569442	9.1078E-14	4.49698E-13	74747	Ddit4
05206	MicroRNAs in cancer	5.337569442	9.1078E-14	4.49698E-13	11987	Slc7a1
05206	MicroRNAs in cancer	5.337569442	9.1078E-14	4.49698E-13	18033	Nfkb1
05206	MicroRNAs in cancer	5.337569442	9.1078E-14	4.49698E-13	18792	Plau
05206	MicroRNAs in cancer	5.337569442	9.1078E-14	4.49698E-13	13078	Cyp1b1
05206	MicroRNAs in cancer	5.337569442	9.1078E-14	4.49698E-13	12703	Socs1
05206	MicroRNAs in cancer	5.337569442	9.1078E-14	4.49698E-13	17295	Met
05206	MicroRNAs in cancer	5.337569442	9.1078E-14	4.49698E-13	17869	Myc
05206	MicroRNAs in cancer	5.337569442	9.1078E-14	4.49698E-13	15368	Hmox1
05206	MicroRNAs in cancer	5.337569442	9.1078E-14	4.49698E-13	18671	Abcb1a
05206	MicroRNAs in cancer	5.337569442	9.1078E-14	4.49698E-13	22350	Ezr
05206	MicroRNAs in cancer	5.337569442	9.1078E-14	4.49698E-13	13637	Efna2
05206	MicroRNAs in cancer	5.337569442	9.1078E-14	4.49698E-13	14086	Fscn1
05206	MicroRNAs in cancer	5.337569442	9.1078E-14	4.49698E-13	18708	Pik3r1
05206	MicroRNAs in cancer	5.337569442	9.1078E-14	4.49698E-13	18128	Notch1
05206	MicroRNAs in cancer	5.337569442	9.1078E-14	4.49698E-13	140486	Igf2bp1
05206	MicroRNAs in cancer	5.337569442	9.1078E-14	4.49698E-13	56717	Mtor
05206	MicroRNAs in cancer	5.337569442	9.1078E-14	4.49698E-13	22339	Vegfa

05206	MicroRNAs in cancer	5.337569442	9.1078E-14	4.49698E-13	19225	Ptgs2
05206	MicroRNAs in cancer	5.337569442	9.1078E-14	4.49698E-13	13640	Efna5
05206	MicroRNAs in cancer	5.337569442	9.1078E-14	4.49698E-13	21825	Thbs1
05206	MicroRNAs in cancer	5.337569442	9.1078E-14	4.49698E-13	17250	Abcc1
04622	RIG-I-like receptor signaling pathway	12.27402687	1.16908E-13	5.68354E-13	18035	Nfkbia
04622	RIG-I-like receptor signaling pathway	12.27402687	1.16908E-13	5.68354E-13	21926	Tnf
04622	RIG-I-like receptor signaling pathway	12.27402687	1.16908E-13	5.68354E-13	18036	Nfkbib
04622	RIG-I-like receptor signaling pathway	12.27402687	1.16908E-13	5.68354E-13	15945	Cxcl10
04622	RIG-I-like receptor signaling pathway	12.27402687	1.16908E-13	5.68354E-13	16159	Il12a
04622	RIG-I-like receptor signaling pathway	12.27402687	1.16908E-13	5.68354E-13	21353	Tank
04622	RIG-I-like receptor signaling pathway	12.27402687	1.16908E-13	5.68354E-13	18033	Nfkb1
04622	RIG-I-like receptor signaling pathway	12.27402687	1.16908E-13	5.68354E-13	54123	Irf7
04622	RIG-I-like receptor signaling pathway	12.27402687	1.16908E-13	5.68354E-13	56489	Ikbke
04622	RIG-I-like receptor signaling pathway	12.27402687	1.16908E-13	5.68354E-13	26415	Mapk13
04622	RIG-I-like receptor signaling pathway	12.27402687	1.16908E-13	5.68354E-13	16160	Il12b
04622	RIG-I-like receptor signaling pathway	12.27402687	1.16908E-13	5.68354E-13	100038882	Isg15
04622	RIG-I-like receptor signaling pathway	12.27402687	1.16908E-13	5.68354E-13	22030	Traf2
04622	RIG-I-like receptor signaling pathway	12.27402687	1.16908E-13	5.68354E-13	230073	Ddx58
04622	RIG-I-like receptor signaling pathway	12.27402687	1.16908E-13	5.68354E-13	71586	Ifih1
04622	RIG-I-like receptor signaling pathway	12.27402687	1.16908E-13	5.68354E-13	56480	Tbk1
04622	RIG-I-like receptor signaling pathway	12.27402687	1.16908E-13	5.68354E-13	80861	Dhx58
05014	Amyotrophic lateral sclerosis	4.793777433	1.43954E-13	6.89236E-13	13163	Daxx
05014	Amyotrophic lateral sclerosis	4.793777433	1.43954E-13	6.89236E-13	12362	Casp1
05014	Amyotrophic lateral sclerosis	4.793777433	1.43954E-13	6.89236E-13	21938	Tnfrsf1b
05014	Amyotrophic lateral sclerosis	4.793777433	1.43954E-13	6.89236E-13	22142	Tuba1a
05014	Amyotrophic lateral sclerosis	4.793777433	1.43954E-13	6.89236E-13	56717	Mtor
05014	Amyotrophic lateral sclerosis	4.793777433	1.43954E-13	6.89236E-13	19181	Psmc2
05014	Amyotrophic lateral sclerosis	4.793777433	1.43954E-13	6.89236E-13	26442	Psma5
05014	Amyotrophic lateral sclerosis	4.793777433	1.43954E-13	6.89236E-13	19166	Psma2
05014	Amyotrophic lateral sclerosis	4.793777433	1.43954E-13	6.89236E-13	26443	Psma6
05014	Amyotrophic lateral sclerosis	4.793777433	1.43954E-13	6.89236E-13	26399	Map2k6
05014	Amyotrophic lateral sclerosis	4.793777433	1.43954E-13	6.89236E-13	69077	Psmd11
05014	Amyotrophic lateral sclerosis	4.793777433	1.43954E-13	6.89236E-13	73205	C9orf72
05014	Amyotrophic lateral sclerosis	4.793777433	1.43954E-13	6.89236E-13	56480	Tbk1
05014	Amyotrophic lateral sclerosis	4.793777433	1.43954E-13	6.89236E-13	14828	Hspa5
05014	Amyotrophic lateral sclerosis	4.793777433	1.43954E-13	6.89236E-13	22030	Traf2
05014	Amyotrophic lateral sclerosis	4.793777433	1.43954E-13	6.89236E-13	19175	Psmb6
05014	Amyotrophic lateral sclerosis	4.793777433	1.43954E-13	6.89236E-13	18645	Pfn2
05014	Amyotrophic lateral sclerosis	4.793777433	1.43954E-13	6.89236E-13	59029	Psmd14
05014	Amyotrophic lateral sclerosis	4.793777433	1.43954E-13	6.89236E-13	21353	Tank
05014	Amyotrophic lateral sclerosis	4.793777433	1.43954E-13	6.89236E-13	20511	Slc1a2
05014	Amyotrophic lateral sclerosis	4.793777433	1.43954E-13	6.89236E-13	13063	Cycs

05014	Amyotrophic lateral sclerosis	4.793777433	1.43954E-13	6.89236E-13	12122	Bid
05014	Amyotrophic lateral sclerosis	4.793777433	1.43954E-13	6.89236E-13	11911	Atf4
05014	Amyotrophic lateral sclerosis	4.793777433	1.43954E-13	6.89236E-13	19173	Psmb5
05014	Amyotrophic lateral sclerosis	4.793777433	1.43954E-13	6.89236E-13	19057	Ppp3cc
05014	Amyotrophic lateral sclerosis	4.793777433	1.43954E-13	6.89236E-13	22153	Tubb4a
05014	Amyotrophic lateral sclerosis	4.793777433	1.43954E-13	6.89236E-13	22145	Tuba4a
05014	Amyotrophic lateral sclerosis	4.793777433	1.43954E-13	6.89236E-13	56436	Adrm1
05014	Amyotrophic lateral sclerosis	4.793777433	1.43954E-13	6.89236E-13	26415	Mapk13
05014	Amyotrophic lateral sclerosis	4.793777433	1.43954E-13	6.89236E-13	211323	Nrg1
05014	Amyotrophic lateral sclerosis	4.793777433	1.43954E-13	6.89236E-13	14814	Grin2d
05014	Amyotrophic lateral sclerosis	4.793777433	1.43954E-13	6.89236E-13	18412	Sqstm1
05014	Amyotrophic lateral sclerosis	4.793777433	1.43954E-13	6.89236E-13	21926	Tnf
05014	Amyotrophic lateral sclerosis	4.793777433	1.43954E-13	6.89236E-13	69926	Dnah17
05014	Amyotrophic lateral sclerosis	4.793777433	1.43954E-13	6.89236E-13	18126	Nos2
04514	Cell adhesion molecules	6.971049745	4.61768E-13	2.17789E-12	110558	H2-Q9
04514	Cell adhesion molecules	6.971049745	4.61768E-13	2.17789E-12	15018	H2-Q7
04514	Cell adhesion molecules	6.971049745	4.61768E-13	2.17789E-12	69524	Esam
04514	Cell adhesion molecules	6.971049745	4.61768E-13	2.17789E-12	16408	Itgal
04514	Cell adhesion molecules	6.971049745	4.61768E-13	2.17789E-12	14990	H2-M2
04514	Cell adhesion molecules	6.971049745	4.61768E-13	2.17789E-12	12562	Cdh5
04514	Cell adhesion molecules	6.971049745	4.61768E-13	2.17789E-12	13003	Vcan
04514	Cell adhesion molecules	6.971049745	4.61768E-13	2.17789E-12	12524	Cd86
04514	Cell adhesion molecules	6.971049745	4.61768E-13	2.17789E-12	52118	Pvr
04514	Cell adhesion molecules	6.971049745	4.61768E-13	2.17789E-12	20737	Spn
04514	Cell adhesion molecules	6.971049745	4.61768E-13	2.17789E-12	171171	Ntng2
04514	Cell adhesion molecules	6.971049745	4.61768E-13	2.17789E-12	15894	Icam1
04514	Cell adhesion molecules	6.971049745	4.61768E-13	2.17789E-12	104099	Itga9
04514	Cell adhesion molecules	6.971049745	4.61768E-13	2.17789E-12	68481	Mpzl1
04514	Cell adhesion molecules	6.971049745	4.61768E-13	2.17789E-12	320910	Itgb8
04514	Cell adhesion molecules	6.971049745	4.61768E-13	2.17789E-12	22329	Vcam1
04514	Cell adhesion molecules	6.971049745	4.61768E-13	2.17789E-12	15001	H2-Oa
04514	Cell adhesion molecules	6.971049745	4.61768E-13	2.17789E-12	20971	Sdc4
04514	Cell adhesion molecules	6.971049745	4.61768E-13	2.17789E-12	60533	Cd274
04514	Cell adhesion molecules	6.971049745	4.61768E-13	2.17789E-12	15024	H2-T10
04514	Cell adhesion molecules	6.971049745	4.61768E-13	2.17789E-12	15039	H2-T22
04514	Cell adhesion molecules	6.971049745	4.61768E-13	2.17789E-12	50723	Icosl
04514	Cell adhesion molecules	6.971049745	4.61768E-13	2.17789E-12	20969	Sdc1
04514	Cell adhesion molecules	6.971049745	4.61768E-13	2.17789E-12	21939	Cd40
04926	Relaxin signaling pathway	8.227459873	5.62026E-13	2.61177E-12	13618	Ednrb
04926	Relaxin signaling pathway	8.227459873	5.62026E-13	2.61177E-12	13649	Egfr
04926	Relaxin signaling pathway	8.227459873	5.62026E-13	2.61177E-12	13614	Edn1
04926	Relaxin signaling pathway	8.227459873	5.62026E-13	2.61177E-12	26415	Mapk13

04926	Relaxin signaling pathway	8.227459873	5.62026E-13	2.61177E-12	20779	Src
04926	Relaxin signaling pathway	8.227459873	5.62026E-13	2.61177E-12	11911	Atf4
04926	Relaxin signaling pathway	8.227459873	5.62026E-13	2.61177E-12	18798	Plcb4
04926	Relaxin signaling pathway	8.227459873	5.62026E-13	2.61177E-12	17386	Mmp13
04926	Relaxin signaling pathway	8.227459873	5.62026E-13	2.61177E-12	18126	Nos2
04926	Relaxin signaling pathway	8.227459873	5.62026E-13	2.61177E-12	22341	Vegfc
04926	Relaxin signaling pathway	8.227459873	5.62026E-13	2.61177E-12	11475	Acta2
04926	Relaxin signaling pathway	8.227459873	5.62026E-13	2.61177E-12	231991	Creb5
04926	Relaxin signaling pathway	8.227459873	5.62026E-13	2.61177E-12	18033	Nfkb1
04926	Relaxin signaling pathway	8.227459873	5.62026E-13	2.61177E-12	18708	Pik3r1
04926	Relaxin signaling pathway	8.227459873	5.62026E-13	2.61177E-12	14701	Gng12
04926	Relaxin signaling pathway	8.227459873	5.62026E-13	2.61177E-12	12827	Col4a2
04926	Relaxin signaling pathway	8.227459873	5.62026E-13	2.61177E-12	17395	Mmp9
04926	Relaxin signaling pathway	8.227459873	5.62026E-13	2.61177E-12	17390	Mmp2
04926	Relaxin signaling pathway	8.227459873	5.62026E-13	2.61177E-12	22339	Vegfa
04926	Relaxin signaling pathway	8.227459873	5.62026E-13	2.61177E-12	18035	Nfkbia
04926	Relaxin signaling pathway	8.227459873	5.62026E-13	2.61177E-12	83996	Mmp1b
05020	Prion disease	5.46889257	7.9576E-13	3.64435E-12	26442	Psma5
05020	Prion disease	5.46889257	7.9576E-13	3.64435E-12	26443	Psma6
05020	Prion disease	5.46889257	7.9576E-13	3.64435E-12	56436	Adrm1
05020	Prion disease	5.46889257	7.9576E-13	3.64435E-12	19181	Psmc2
05020	Prion disease	5.46889257	7.9576E-13	3.64435E-12	21926	Tnf
05020	Prion disease	5.46889257	7.9576E-13	3.64435E-12	26415	Mapk13
05020	Prion disease	5.46889257	7.9576E-13	3.64435E-12	19173	Psmb5
05020	Prion disease	5.46889257	7.9576E-13	3.64435E-12	20304	Ccl5
05020	Prion disease	5.46889257	7.9576E-13	3.64435E-12	14814	Grin2d
05020	Prion disease	5.46889257	7.9576E-13	3.64435E-12	11911	Atf4
05020	Prion disease	5.46889257	7.9576E-13	3.64435E-12	231991	Creb5
05020	Prion disease	5.46889257	7.9576E-13	3.64435E-12	13058	Cybb
05020	Prion disease	5.46889257	7.9576E-13	3.64435E-12	19057	Ppp3cc
05020	Prion disease	5.46889257	7.9576E-13	3.64435E-12	22142	Tuba1a
05020	Prion disease	5.46889257	7.9576E-13	3.64435E-12	19166	Psma2
05020	Prion disease	5.46889257	7.9576E-13	3.64435E-12	18128	Notch1
05020	Prion disease	5.46889257	7.9576E-13	3.64435E-12	19175	Psmb6
05020	Prion disease	5.46889257	7.9576E-13	3.64435E-12	16176	Il1b
05020	Prion disease	5.46889257	7.9576E-13	3.64435E-12	22145	Tuba4a
05020	Prion disease	5.46889257	7.9576E-13	3.64435E-12	12389	Cav1
05020	Prion disease	5.46889257	7.9576E-13	3.64435E-12	16193	Il6
05020	Prion disease	5.46889257	7.9576E-13	3.64435E-12	18708	Pik3r1
05020	Prion disease	5.46889257	7.9576E-13	3.64435E-12	59029	Psmd14
05020	Prion disease	5.46889257	7.9576E-13	3.64435E-12	69077	Psmd11
05020	Prion disease	5.46889257	7.9576E-13	3.64435E-12	13063	Cycs

05020	Prion disease	5.46889257	7.9576E-13	3.64435E-12	16175	Il1a
05020	Prion disease	5.46889257	7.9576E-13	3.64435E-12	22153	Tubb4a
05020	Prion disease	5.46889257	7.9576E-13	3.64435E-12	13653	Egr1
05020	Prion disease	5.46889257	7.9576E-13	3.64435E-12	14828	Hspa5
03050	Proteasome	15.05450104	9.85322E-13	4.44802E-12	19175	Psmb6
03050	Proteasome	15.05450104	9.85322E-13	4.44802E-12	26442	Psma5
03050	Proteasome	15.05450104	9.85322E-13	4.44802E-12	19188	Psme2
03050	Proteasome	15.05450104	9.85322E-13	4.44802E-12	19166	Psma2
03050	Proteasome	15.05450104	9.85322E-13	4.44802E-12	26443	Psma6
03050	Proteasome	15.05450104	9.85322E-13	4.44802E-12	66537	Pomp
03050	Proteasome	15.05450104	9.85322E-13	4.44802E-12	19173	Psmb5
03050	Proteasome	15.05450104	9.85322E-13	4.44802E-12	69077	Psmd11
03050	Proteasome	15.05450104	9.85322E-13	4.44802E-12	15978	Ifng
03050	Proteasome	15.05450104	9.85322E-13	4.44802E-12	59029	Psmd14
03050	Proteasome	15.05450104	9.85322E-13	4.44802E-12	19181	Psmc2
03050	Proteasome	15.05450104	9.85322E-13	4.44802E-12	56436	Adrm1
03050	Proteasome	15.05450104	9.85322E-13	4.44802E-12	19171	Psmb10
03050	Proteasome	15.05450104	9.85322E-13	4.44802E-12	16913	Psmb8
04360	Axon guidance	6.701451136	1.1159E-12	4.96656E-12	171171	Ntng2
04360	Axon guidance	6.701451136	1.1159E-12	4.96656E-12	18708	Pik3r1
04360	Axon guidance	6.701451136	1.1159E-12	4.96656E-12	117600	Srgap1
04360	Axon guidance	6.701451136	1.1159E-12	4.96656E-12	228026	Pdk1
04360	Axon guidance	6.701451136	1.1159E-12	4.96656E-12	13838	Epha4
04360	Axon guidance	6.701451136	1.1159E-12	4.96656E-12	268902	Robo2
04360	Axon guidance	6.701451136	1.1159E-12	4.96656E-12	17973	Nck1
04360	Axon guidance	6.701451136	1.1159E-12	4.96656E-12	20563	Slit2
04360	Axon guidance	6.701451136	1.1159E-12	4.96656E-12	108058	Camk2d
04360	Axon guidance	6.701451136	1.1159E-12	4.96656E-12	19057	Ppp3cc
04360	Axon guidance	6.701451136	1.1159E-12	4.96656E-12	17295	Met
04360	Axon guidance	6.701451136	1.1159E-12	4.96656E-12	13637	Efna2
04360	Axon guidance	6.701451136	1.1159E-12	4.96656E-12	20351	Sema4a
04360	Axon guidance	6.701451136	1.1159E-12	4.96656E-12	22253	Unc5c
04360	Axon guidance	6.701451136	1.1159E-12	4.96656E-12	20349	Sema3e
04360	Axon guidance	6.701451136	1.1159E-12	4.96656E-12	223881	Rnd1
04360	Axon guidance	6.701451136	1.1159E-12	4.96656E-12	107449	Unc5b
04360	Axon guidance	6.701451136	1.1159E-12	4.96656E-12	231148	Ablim2
04360	Axon guidance	6.701451136	1.1159E-12	4.96656E-12	20779	Src
04360	Axon guidance	6.701451136	1.1159E-12	4.96656E-12	20361	Sema7a
04360	Axon guidance	6.701451136	1.1159E-12	4.96656E-12	13642	Efnb2
04360	Axon guidance	6.701451136	1.1159E-12	4.96656E-12	20348	Sema3c
04360	Axon guidance	6.701451136	1.1159E-12	4.96656E-12	13640	Efna5
04360	Axon guidance	6.701451136	1.1159E-12	4.96656E-12	117606	Boc

04080	Neuroactive ligand-receptor interaction	4.658725283	1.70335E-12	7.47582E-12	12062	Bdkrb2
04080	Neuroactive ligand-receptor interaction	4.658725283	1.70335E-12	7.47582E-12	58861	Cysltr1
04080	Neuroactive ligand-receptor interaction	4.658725283	1.70335E-12	7.47582E-12	13610	S1pr3
04080	Neuroactive ligand-receptor interaction	4.658725283	1.70335E-12	7.47582E-12	16995	Ltb4r1
04080	Neuroactive ligand-receptor interaction	4.658725283	1.70335E-12	7.47582E-12	381853	Gipr
04080	Neuroactive ligand-receptor interaction	4.658725283	1.70335E-12	7.47582E-12	15551	Htr1b
04080	Neuroactive ligand-receptor interaction	4.658725283	1.70335E-12	7.47582E-12	54598	Calcrl
04080	Neuroactive ligand-receptor interaction	4.658725283	1.70335E-12	7.47582E-12	14745	Lpar1
04080	Neuroactive ligand-receptor interaction	4.658725283	1.70335E-12	7.47582E-12	14829	Grpr
04080	Neuroactive ligand-receptor interaction	4.658725283	1.70335E-12	7.47582E-12	18619	Penk
04080	Neuroactive ligand-receptor interaction	4.658725283	1.70335E-12	7.47582E-12	13618	Ednrb
04080	Neuroactive ligand-receptor interaction	4.658725283	1.70335E-12	7.47582E-12	15557	Htr1f
04080	Neuroactive ligand-receptor interaction	4.658725283	1.70335E-12	7.47582E-12	140795	P2ry14
04080	Neuroactive ligand-receptor interaction	4.658725283	1.70335E-12	7.47582E-12	14427	Galr1
04080	Neuroactive ligand-receptor interaction	4.658725283	1.70335E-12	7.47582E-12	16847	Lepr
04080	Neuroactive ligand-receptor interaction	4.658725283	1.70335E-12	7.47582E-12	14294	Fpr3
04080	Neuroactive ligand-receptor interaction	4.658725283	1.70335E-12	7.47582E-12	94045	P2rx5
04080	Neuroactive ligand-receptor interaction	4.658725283	1.70335E-12	7.47582E-12	14293	Fpr1
04080	Neuroactive ligand-receptor interaction	4.658725283	1.70335E-12	7.47582E-12	11540	Adora2a
04080	Neuroactive ligand-receptor interaction	4.658725283	1.70335E-12	7.47582E-12	14289	Fpr2
04080	Neuroactive ligand-receptor interaction	4.658725283	1.70335E-12	7.47582E-12	12311	Calcr
04080	Neuroactive ligand-receptor interaction	4.658725283	1.70335E-12	7.47582E-12	15566	Htr7
04080	Neuroactive ligand-receptor interaction	4.658725283	1.70335E-12	7.47582E-12	74191	P2ry13
04080	Neuroactive ligand-receptor interaction	4.658725283	1.70335E-12	7.47582E-12	14062	F2r
04080	Neuroactive ligand-receptor interaction	4.658725283	1.70335E-12	7.47582E-12	14064	F2rl2
04080	Neuroactive ligand-receptor interaction	4.658725283	1.70335E-12	7.47582E-12	11541	Adora2b
04080	Neuroactive ligand-receptor interaction	4.658725283	1.70335E-12	7.47582E-12	12257	Tspo
04080	Neuroactive ligand-receptor interaction	4.658725283	1.70335E-12	7.47582E-12	15466	Hrh2
04080	Neuroactive ligand-receptor interaction	4.658725283	1.70335E-12	7.47582E-12	11535	Adm
04080	Neuroactive ligand-receptor interaction	4.658725283	1.70335E-12	7.47582E-12	13614	Edn1
04080	Neuroactive ligand-receptor interaction	4.658725283	1.70335E-12	7.47582E-12	18442	P2ry2
04080	Neuroactive ligand-receptor interaction	4.658725283	1.70335E-12	7.47582E-12	12266	C3
04080	Neuroactive ligand-receptor interaction	4.658725283	1.70335E-12	7.47582E-12	14814	Grin2d
04940	Type I diabetes mellitus	11.55202529	1.85143E-12	8.01439E-12	16176	Il1b
04940	Type I diabetes mellitus	11.55202529	1.85143E-12	8.01439E-12	16175	Il1a
04940	Type I diabetes mellitus	11.55202529	1.85143E-12	8.01439E-12	16992	Lta
04940	Type I diabetes mellitus	11.55202529	1.85143E-12	8.01439E-12	21926	Tnf
04940	Type I diabetes mellitus	11.55202529	1.85143E-12	8.01439E-12	15978	Ifng
04940	Type I diabetes mellitus	11.55202529	1.85143E-12	8.01439E-12	110558	H2-Q9
04940	Type I diabetes mellitus	11.55202529	1.85143E-12	8.01439E-12	15024	H2-T10
04940	Type I diabetes mellitus	11.55202529	1.85143E-12	8.01439E-12	14102	Fas
04940	Type I diabetes mellitus	11.55202529	1.85143E-12	8.01439E-12	15018	H2-Q7

04940	Type I diabetes mellitus	11.55202529	1.85143E-12	8.01439E-12	15039	H2-T22
04940	Type I diabetes mellitus	11.55202529	1.85143E-12	8.01439E-12	16160	Il12b
04940	Type I diabetes mellitus	11.55202529	1.85143E-12	8.01439E-12	19275	Ptprn
04940	Type I diabetes mellitus	11.55202529	1.85143E-12	8.01439E-12	15001	H2-Oa
04940	Type I diabetes mellitus	11.55202529	1.85143E-12	8.01439E-12	14990	H2-M2
04940	Type I diabetes mellitus	11.55202529	1.85143E-12	8.01439E-12	16159	Il12a
04940	Type I diabetes mellitus	11.55202529	1.85143E-12	8.01439E-12	12524	Cd86
04640	Hematopoietic cell lineage	9.576020965	2.05697E-12	8.78384E-12	12981	Csf2
04640	Hematopoietic cell lineage	9.576020965	2.05697E-12	8.78384E-12	16193	Il6
04640	Hematopoietic cell lineage	9.576020965	2.05697E-12	8.78384E-12	15001	H2-Oa
04640	Hematopoietic cell lineage	9.576020965	2.05697E-12	8.78384E-12	16177	Il1r1
04640	Hematopoietic cell lineage	9.576020965	2.05697E-12	8.78384E-12	12985	Csf3
04640	Hematopoietic cell lineage	9.576020965	2.05697E-12	8.78384E-12	12494	Cd38
04640	Hematopoietic cell lineage	9.576020965	2.05697E-12	8.78384E-12	16176	Il1b
04640	Hematopoietic cell lineage	9.576020965	2.05697E-12	8.78384E-12	12977	Csf1
04640	Hematopoietic cell lineage	9.576020965	2.05697E-12	8.78384E-12	16402	Itga5
04640	Hematopoietic cell lineage	9.576020965	2.05697E-12	8.78384E-12	109700	Itga1
04640	Hematopoietic cell lineage	9.576020965	2.05697E-12	8.78384E-12	12489	Cd33
04640	Hematopoietic cell lineage	9.576020965	2.05697E-12	8.78384E-12	16175	Il1a
04640	Hematopoietic cell lineage	9.576020965	2.05697E-12	8.78384E-12	12475	Cd14
04640	Hematopoietic cell lineage	9.576020965	2.05697E-12	8.78384E-12	16190	Il4ra
04640	Hematopoietic cell lineage	9.576020965	2.05697E-12	8.78384E-12	12484	Cd24a
04640	Hematopoietic cell lineage	9.576020965	2.05697E-12	8.78384E-12	21926	Tnf
04640	Hematopoietic cell lineage	9.576020965	2.05697E-12	8.78384E-12	12986	Csf3r
04640	Hematopoietic cell lineage	9.576020965	2.05697E-12	8.78384E-12	12479	Cd1d1
04660	T cell receptor signaling pathway	8.832252347	8.75587E-12	3.68914E-11	16797	Lat
04660	T cell receptor signaling pathway	8.832252347	8.75587E-12	3.68914E-11	12981	Csf2
04660	T cell receptor signaling pathway	8.832252347	8.75587E-12	3.68914E-11	18708	Pik3r1
04660	T cell receptor signaling pathway	8.832252347	8.75587E-12	3.68914E-11	26410	Map3k8
04660	T cell receptor signaling pathway	8.832252347	8.75587E-12	3.68914E-11	26415	Mapk13
04660	T cell receptor signaling pathway	8.832252347	8.75587E-12	3.68914E-11	19057	Ppp3cc
04660	T cell receptor signaling pathway	8.832252347	8.75587E-12	3.68914E-11	16822	Lcp2
04660	T cell receptor signaling pathway	8.832252347	8.75587E-12	3.68914E-11	12503	Cd247
04660	T cell receptor signaling pathway	8.832252347	8.75587E-12	3.68914E-11	18033	Nfkb1
04660	T cell receptor signaling pathway	8.832252347	8.75587E-12	3.68914E-11	15978	Ifng
04660	T cell receptor signaling pathway	8.832252347	8.75587E-12	3.68914E-11	240354	Malt1
04660	T cell receptor signaling pathway	8.832252347	8.75587E-12	3.68914E-11	21926	Tnf
04660	T cell receptor signaling pathway	8.832252347	8.75587E-12	3.68914E-11	18035	Nfkbia
04660	T cell receptor signaling pathway	8.832252347	8.75587E-12	3.68914E-11	16153	Il10
04660	T cell receptor signaling pathway	8.832252347	8.75587E-12	3.68914E-11	18036	Nfkbib
04660	T cell receptor signaling pathway	8.832252347	8.75587E-12	3.68914E-11	17973	Nck1
04660	T cell receptor signaling pathway	8.832252347	8.75587E-12	3.68914E-11	19419	Rasgrp1

04660	T cell receptor signaling pathway	8.832252347	8.75587E-12	3.68914E-11	18037	Nfkbie
04218	Cellular senescence	6.317513831	1.21251E-11	5.04148E-11	56717	Mtor
04218	Cellular senescence	6.317513831	1.21251E-11	5.04148E-11	18708	Pik3r1
04218	Cellular senescence	6.317513831	1.21251E-11	5.04148E-11	12575	Cdkn1a
04218	Cellular senescence	6.317513831	1.21251E-11	5.04148E-11	12579	Cdkn2b
04218	Cellular senescence	6.317513831	1.21251E-11	5.04148E-11	16175	Il1a
04218	Cellular senescence	6.317513831	1.21251E-11	5.04148E-11	17869	Myc
04218	Cellular senescence	6.317513831	1.21251E-11	5.04148E-11	17164	Mapkapk2
04218	Cellular senescence	6.317513831	1.21251E-11	5.04148E-11	18412	Sqstm1
04218	Cellular senescence	6.317513831	1.21251E-11	5.04148E-11	14990	H2-M2
04218	Cellular senescence	6.317513831	1.21251E-11	5.04148E-11	13197	Gadd45a
04218	Cellular senescence	6.317513831	1.21251E-11	5.04148E-11	233020	Hipk4
04218	Cellular senescence	6.317513831	1.21251E-11	5.04148E-11	15024	H2-T10
04218	Cellular senescence	6.317513831	1.21251E-11	5.04148E-11	18033	Nfkb1
04218	Cellular senescence	6.317513831	1.21251E-11	5.04148E-11	12444	Ccnd2
04218	Cellular senescence	6.317513831	1.21251E-11	5.04148E-11	15039	H2-T22
04218	Cellular senescence	6.317513831	1.21251E-11	5.04148E-11	26399	Map2k6
04218	Cellular senescence	6.317513831	1.21251E-11	5.04148E-11	110558	H2-Q9
04218	Cellular senescence	6.317513831	1.21251E-11	5.04148E-11	16193	Il6
04218	Cellular senescence	6.317513831	1.21251E-11	5.04148E-11	19057	Ppp3cc
04218	Cellular senescence	6.317513831	1.21251E-11	5.04148E-11	17873	Gadd45b
04218	Cellular senescence	6.317513831	1.21251E-11	5.04148E-11	26415	Mapk13
04218	Cellular senescence	6.317513831	1.21251E-11	5.04148E-11	23871	Ets1
04218	Cellular senescence	6.317513831	1.21251E-11	5.04148E-11	15018	H2-Q7
04666	Fc gamma R-mediated phagocytosis	9.33893349	1.38976E-11	5.70342E-11	67916	Plpp3
04666	Fc gamma R-mediated phagocytosis	9.33893349	1.38976E-11	5.70342E-11	17909	Myo10
04666	Fc gamma R-mediated phagocytosis	9.33893349	1.38976E-11	5.70342E-11	14130	Fcgr2b
04666	Fc gamma R-mediated phagocytosis	9.33893349	1.38976E-11	5.70342E-11	22323	Vasp
04666	Fc gamma R-mediated phagocytosis	9.33893349	1.38976E-11	5.70342E-11	17357	Marcksl1
04666	Fc gamma R-mediated phagocytosis	9.33893349	1.38976E-11	5.70342E-11	18720	Pip5k1a
04666	Fc gamma R-mediated phagocytosis	9.33893349	1.38976E-11	5.70342E-11	18783	Pla2g4a
04666	Fc gamma R-mediated phagocytosis	9.33893349	1.38976E-11	5.70342E-11	15162	Hck
04666	Fc gamma R-mediated phagocytosis	9.33893349	1.38976E-11	5.70342E-11	19012	Plpp1
04666	Fc gamma R-mediated phagocytosis	9.33893349	1.38976E-11	5.70342E-11	16797	Lat
04666	Fc gamma R-mediated phagocytosis	9.33893349	1.38976E-11	5.70342E-11	18708	Pik3r1
04666	Fc gamma R-mediated phagocytosis	9.33893349	1.38976E-11	5.70342E-11	20698	Sphk1
04666	Fc gamma R-mediated phagocytosis	9.33893349	1.38976E-11	5.70342E-11	20963	Syk
04666	Fc gamma R-mediated phagocytosis	9.33893349	1.38976E-11	5.70342E-11	20259	Scin
04666	Fc gamma R-mediated phagocytosis	9.33893349	1.38976E-11	5.70342E-11	18719	Pip5k1b
04666	Fc gamma R-mediated phagocytosis	9.33893349	1.38976E-11	5.70342E-11	17096	Lyn
04666	Fc gamma R-mediated phagocytosis	9.33893349	1.38976E-11	5.70342E-11	17118	Marcks
00230	Purine metabolism	7.432369213	1.50079E-11	6.08014E-11	18950	Pnp

00230	Purine metabolism	7.432369213	1.50079E-11	6.08014E-11	11637	Ak2
00230	Purine metabolism	7.432369213	1.50079E-11	6.08014E-11	11717	Ampd3
00230	Purine metabolism	7.432369213	1.50079E-11	6.08014E-11	11639	Ak4
00230	Purine metabolism	7.432369213	1.50079E-11	6.08014E-11	23984	Pde10a
00230	Purine metabolism	7.432369213	1.50079E-11	6.08014E-11	23959	Nt5e
00230	Purine metabolism	7.432369213	1.50079E-11	6.08014E-11	76974	Urah
00230	Purine metabolism	7.432369213	1.50079E-11	6.08014E-11	54369	Nme6
00230	Purine metabolism	7.432369213	1.50079E-11	6.08014E-11	18578	Pde4b
00230	Purine metabolism	7.432369213	1.50079E-11	6.08014E-11	230718	Nt5c1a
00230	Purine metabolism	7.432369213	1.50079E-11	6.08014E-11	13178	Dck
00230	Purine metabolism	7.432369213	1.50079E-11	6.08014E-11	238871	Pde4d
00230	Purine metabolism	7.432369213	1.50079E-11	6.08014E-11	66681	Pgm2
00230	Purine metabolism	7.432369213	1.50079E-11	6.08014E-11	102216272	Ak6
00230	Purine metabolism	7.432369213	1.50079E-11	6.08014E-11	18587	Pde6b
00230	Purine metabolism	7.432369213	1.50079E-11	6.08014E-11	224794	Enpp4
00230	Purine metabolism	7.432369213	1.50079E-11	6.08014E-11	107569	Nt5c3
00230	Purine metabolism	7.432369213	1.50079E-11	6.08014E-11	23972	Papss2
00230	Purine metabolism	7.432369213	1.50079E-11	6.08014E-11	242202	Pde5a
00230	Purine metabolism	7.432369213	1.50079E-11	6.08014E-11	73707	Gucy2g
05222	Small cell lung cancer	9.23851485	1.66933E-11	6.67731E-11	18708	Pik3r1
05222	Small cell lung cancer	9.23851485	1.66933E-11	6.67731E-11	13063	Cycs
05222	Small cell lung cancer	9.23851485	1.66933E-11	6.67731E-11	18033	Nfkb1
05222	Small cell lung cancer	9.23851485	1.66933E-11	6.67731E-11	17869	Myc
05222	Small cell lung cancer	9.23851485	1.66933E-11	6.67731E-11	19225	Ptgs2
05222	Small cell lung cancer	9.23851485	1.66933E-11	6.67731E-11	12579	Cdkn2b
05222	Small cell lung cancer	9.23851485	1.66933E-11	6.67731E-11	18035	Nfkbia
05222	Small cell lung cancer	9.23851485	1.66933E-11	6.67731E-11	17873	Gadd45b
05222	Small cell lung cancer	9.23851485	1.66933E-11	6.67731E-11	11796	Birc3
05222	Small cell lung cancer	9.23851485	1.66933E-11	6.67731E-11	13197	Gadd45a
05222	Small cell lung cancer	9.23851485	1.66933E-11	6.67731E-11	12575	Cdkn1a
05222	Small cell lung cancer	9.23851485	1.66933E-11	6.67731E-11	18126	Nos2
05222	Small cell lung cancer	9.23851485	1.66933E-11	6.67731E-11	22029	Traf1
05222	Small cell lung cancer	9.23851485	1.66933E-11	6.67731E-11	16782	Lamc2
05222	Small cell lung cancer	9.23851485	1.66933E-11	6.67731E-11	22033	Traf5
05222	Small cell lung cancer	9.23851485	1.66933E-11	6.67731E-11	22030	Traf2
05222	Small cell lung cancer	9.23851485	1.66933E-11	6.67731E-11	12827	Col4a2
05230	Central carbon metabolism in cancer	10.98698058	2.16831E-11	8.56481E-11	18708	Pik3r1
05230	Central carbon metabolism in cancer	10.98698058	2.16831E-11	8.56481E-11	17295	Met
05230	Central carbon metabolism in cancer	10.98698058	2.16831E-11	8.56481E-11	20525	Slc2a1
05230	Central carbon metabolism in cancer	10.98698058	2.16831E-11	8.56481E-11	228026	Pdk1
05230	Central carbon metabolism in cancer	10.98698058	2.16831E-11	8.56481E-11	17869	Myc
05230	Central carbon metabolism in cancer	10.98698058	2.16831E-11	8.56481E-11	13649	Egfr

05230	Central carbon metabolism in cancer	10.98698058	2.16831E-11	8.56481E-11	56717	Mtor
05230	Central carbon metabolism in cancer	10.98698058	2.16831E-11	8.56481E-11	14381	G6pdx
05230	Central carbon metabolism in cancer	10.98698058	2.16831E-11	8.56481E-11	80879	Slc16a3
05230	Central carbon metabolism in cancer	10.98698058	2.16831E-11	8.56481E-11	18648	Pgam1
05230	Central carbon metabolism in cancer	10.98698058	2.16831E-11	8.56481E-11	15251	Hif1a
05230	Central carbon metabolism in cancer	10.98698058	2.16831E-11	8.56481E-11	16828	Ldha
05230	Central carbon metabolism in cancer	10.98698058	2.16831E-11	8.56481E-11	18641	Pfkl
05230	Central carbon metabolism in cancer	10.98698058	2.16831E-11	8.56481E-11	20539	Slc7a5
05230	Central carbon metabolism in cancer	10.98698058	2.16831E-11	8.56481E-11	15277	Hk2
00480	Glutathione metabolism	10.67748817	3.372E-11	1.3155E-10	14381	G6pdx
00480	Glutathione metabolism	10.67748817	3.372E-11	1.3155E-10	110208	Pgd
00480	Glutathione metabolism	10.67748817	3.372E-11	1.3155E-10	18263	Odc1
00480	Glutathione metabolism	10.67748817	3.372E-11	1.3155E-10	14863	Gstm2
00480	Glutathione metabolism	10.67748817	3.372E-11	1.3155E-10	69065	Chac1
00480	Glutathione metabolism	10.67748817	3.372E-11	1.3155E-10	14860	Gsta4
00480	Glutathione metabolism	10.67748817	3.372E-11	1.3155E-10	14782	Gsr
00480	Glutathione metabolism	10.67748817	3.372E-11	1.3155E-10	14630	Gclm
00480	Glutathione metabolism	10.67748817	3.372E-11	1.3155E-10	14854	Gss
00480	Glutathione metabolism	10.67748817	3.372E-11	1.3155E-10	14629	Gclc
00480	Glutathione metabolism	10.67748817	3.372E-11	1.3155E-10	14857	Gsta1
00480	Glutathione metabolism	10.67748817	3.372E-11	1.3155E-10	20810	Srm
00480	Glutathione metabolism	10.67748817	3.372E-11	1.3155E-10	11758	Prdx6
00480	Glutathione metabolism	10.67748817	3.372E-11	1.3155E-10	100042295	Gm3776
00480	Glutathione metabolism	10.67748817	3.372E-11	1.3155E-10	14858	Gsta2
04216	Ferroptosis	15.1620332	4.61424E-11	1.77817E-10	14629	Gclc
04216	Ferroptosis	15.1620332	4.61424E-11	1.77817E-10	14630	Gclm
04216	Ferroptosis	15.1620332	4.61424E-11	1.77817E-10	14854	Gss
04216	Ferroptosis	15.1620332	4.61424E-11	1.77817E-10	433256	Acsl5
04216	Ferroptosis	15.1620332	4.61424E-11	1.77817E-10	50790	Acsl4
04216	Ferroptosis	15.1620332	4.61424E-11	1.77817E-10	213053	Slc39a14
04216	Ferroptosis	15.1620332	4.61424E-11	1.77817E-10	18174	Slc11a2
04216	Ferroptosis	15.1620332	4.61424E-11	1.77817E-10	17254	Slc3a2
04216	Ferroptosis	15.1620332	4.61424E-11	1.77817E-10	26570	Slc7a11
04216	Ferroptosis	15.1620332	4.61424E-11	1.77817E-10	15368	Hmox1
04216	Ferroptosis	15.1620332	4.61424E-11	1.77817E-10	13058	Cybb
04216	Ferroptosis	15.1620332	4.61424E-11	1.77817E-10	14081	Acsl1
05330	Allograft rejection	11.2311357	8.0322E-11	3.05804E-10	21939	Cd40
05330	Allograft rejection	11.2311357	8.0322E-11	3.05804E-10	16153	Il10
05330	Allograft rejection	11.2311357	8.0322E-11	3.05804E-10	15001	H2-Oa
05330	Allograft rejection	11.2311357	8.0322E-11	3.05804E-10	12524	Cd86
05330	Allograft rejection	11.2311357	8.0322E-11	3.05804E-10	15024	H2-T10
05330	Allograft rejection	11.2311357	8.0322E-11	3.05804E-10	16160	Il12b

05330	Allograft rejection	11.2311357	8.0322E-11	3.05804E-10	14102	Fas
05330	Allograft rejection	11.2311357	8.0322E-11	3.05804E-10	16159	Il12a
05330	Allograft rejection	11.2311357	8.0322E-11	3.05804E-10	110558	H2-Q9
05330	Allograft rejection	11.2311357	8.0322E-11	3.05804E-10	14990	H2-M2
05330	Allograft rejection	11.2311357	8.0322E-11	3.05804E-10	15039	H2-T22
05330	Allograft rejection	11.2311357	8.0322E-11	3.05804E-10	15978	Ifng
05330	Allograft rejection	11.2311357	8.0322E-11	3.05804E-10	15018	H2-Q7
05330	Allograft rejection	11.2311357	8.0322E-11	3.05804E-10	21926	Tnf
00270	Cysteine and methionine metabolism	12.39663091	1.09149E-10	4.10607E-10	16828	Ldha
00270	Cysteine and methionine metabolism	12.39663091	1.09149E-10	4.10607E-10	73988	4930438A08Rik
00270	Cysteine and methionine metabolism	12.39663091	1.09149E-10	4.10607E-10	107869	Cth
00270	Cysteine and methionine metabolism	12.39663091	1.09149E-10	4.10607E-10	12035	Bcat1
00270	Cysteine and methionine metabolism	12.39663091	1.09149E-10	4.10607E-10	236539	Phgdh
00270	Cysteine and methionine metabolism	12.39663091	1.09149E-10	4.10607E-10	17448	Mdh2
00270	Cysteine and methionine metabolism	12.39663091	1.09149E-10	4.10607E-10	229905	Kyat3
00270	Cysteine and methionine metabolism	12.39663091	1.09149E-10	4.10607E-10	14630	Gclm
00270	Cysteine and methionine metabolism	12.39663091	1.09149E-10	4.10607E-10	14204	Il4i1
00270	Cysteine and methionine metabolism	12.39663091	1.09149E-10	4.10607E-10	14854	Gss
00270	Cysteine and methionine metabolism	12.39663091	1.09149E-10	4.10607E-10	14629	Gclc
00270	Cysteine and methionine metabolism	12.39663091	1.09149E-10	4.10607E-10	107272	Psat1
00270	Cysteine and methionine metabolism	12.39663091	1.09149E-10	4.10607E-10	20810	Srm
04931	Insulin resistance	7.810744373	2.72472E-10	1.01295E-09	108079	Prkaa2
04931	Insulin resistance	7.810744373	2.72472E-10	1.01295E-09	20112	Rps6ka2
04931	Insulin resistance	7.810744373	2.72472E-10	1.01295E-09	26569	Slc27a4
04931	Insulin resistance	7.810744373	2.72472E-10	1.01295E-09	56717	Mtor
04931	Insulin resistance	7.810744373	2.72472E-10	1.01295E-09	18708	Pik3r1
04931	Insulin resistance	7.810744373	2.72472E-10	1.01295E-09	18035	Nfkbia
04931	Insulin resistance	7.810744373	2.72472E-10	1.01295E-09	14584	Gfpt2
04931	Insulin resistance	7.810744373	2.72472E-10	1.01295E-09	16193	Il6
04931	Insulin resistance	7.810744373	2.72472E-10	1.01295E-09	228775	Trib3
04931	Insulin resistance	7.810744373	2.72472E-10	1.01295E-09	18033	Nfkb1
04931	Insulin resistance	7.810744373	2.72472E-10	1.01295E-09	19246	Ptpn1
04931	Insulin resistance	7.810744373	2.72472E-10	1.01295E-09	105787	Prkaa1
04931	Insulin resistance	7.810744373	2.72472E-10	1.01295E-09	231991	Creb5
04931	Insulin resistance	7.810744373	2.72472E-10	1.01295E-09	12702	Socs3
04931	Insulin resistance	7.810744373	2.72472E-10	1.01295E-09	20525	Slc2a1
04931	Insulin resistance	7.810744373	2.72472E-10	1.01295E-09	21926	Tnf
04931	Insulin resistance	7.810744373	2.72472E-10	1.01295E-09	14936	Gys1
05221	Acute myeloid leukemia	10.10802213	3.65037E-10	1.3413E-09	67603	Dusp6
05221	Acute myeloid leukemia	10.10802213	3.65037E-10	1.3413E-09	18708	Pik3r1
05221	Acute myeloid leukemia	10.10802213	3.65037E-10	1.3413E-09	56717	Mtor
05221	Acute myeloid leukemia	10.10802213	3.65037E-10	1.3413E-09	17869	Myc

05221	Acute myeloid leukemia	10.10802213	3.65037E-10	1.3413E-09	12044	Bcl2a1a
05221	Acute myeloid leukemia	10.10802213	3.65037E-10	1.3413E-09	18715	Pim2
05221	Acute myeloid leukemia	10.10802213	3.65037E-10	1.3413E-09	12981	Csf2
05221	Acute myeloid leukemia	10.10802213	3.65037E-10	1.3413E-09	12047	Bcl2a1d
05221	Acute myeloid leukemia	10.10802213	3.65037E-10	1.3413E-09	12046	Bcl2a1c
05221	Acute myeloid leukemia	10.10802213	3.65037E-10	1.3413E-09	12475	Cd14
05221	Acute myeloid leukemia	10.10802213	3.65037E-10	1.3413E-09	18033	Nfkb1
05221	Acute myeloid leukemia	10.10802213	3.65037E-10	1.3413E-09	18712	Pim1
05221	Acute myeloid leukemia	10.10802213	3.65037E-10	1.3413E-09	19015	Ppard
05221	Acute myeloid leukemia	10.10802213	3.65037E-10	1.3413E-09	12045	Bcl2a1b
05012	Parkinson disease	4.910779982	8.73275E-10	3.1719E-09	22166	Txn1
05012	Parkinson disease	4.910779982	8.73275E-10	3.1719E-09	59029	Psmd14
05012	Parkinson disease	4.910779982	8.73275E-10	3.1719E-09	56791	Ube2l6
05012	Parkinson disease	4.910779982	8.73275E-10	3.1719E-09	14828	Hspa5
05012	Parkinson disease	4.910779982	8.73275E-10	3.1719E-09	108058	Camk2d
05012	Parkinson disease	4.910779982	8.73275E-10	3.1719E-09	69077	Psmd11
05012	Parkinson disease	4.910779982	8.73275E-10	3.1719E-09	13163	Daxx
05012	Parkinson disease	4.910779982	8.73275E-10	3.1719E-09	19181	Psmc2
05012	Parkinson disease	4.910779982	8.73275E-10	3.1719E-09	22153	Tubb4a
05012	Parkinson disease	4.910779982	8.73275E-10	3.1719E-09	66725	Lrrk2
05012	Parkinson disease	4.910779982	8.73275E-10	3.1719E-09	26443	Psma6
05012	Parkinson disease	4.910779982	8.73275E-10	3.1719E-09	11911	Atf4
05012	Parkinson disease	4.910779982	8.73275E-10	3.1719E-09	56436	Adrm1
05012	Parkinson disease	4.910779982	8.73275E-10	3.1719E-09	26442	Psma5
05012	Parkinson disease	4.910779982	8.73275E-10	3.1719E-09	22201	Uba1
05012	Parkinson disease	4.910779982	8.73275E-10	3.1719E-09	19166	Psma2
05012	Parkinson disease	4.910779982	8.73275E-10	3.1719E-09	22145	Tuba4a
05012	Parkinson disease	4.910779982	8.73275E-10	3.1719E-09	22223	Uchl1
05012	Parkinson disease	4.910779982	8.73275E-10	3.1719E-09	20617	Snca
05012	Parkinson disease	4.910779982	8.73275E-10	3.1719E-09	22142	Tuba1a
05012	Parkinson disease	4.910779982	8.73275E-10	3.1719E-09	19173	Psmb5
05012	Parkinson disease	4.910779982	8.73275E-10	3.1719E-09	13063	Cycs
05012	Parkinson disease	4.910779982	8.73275E-10	3.1719E-09	19175	Psmb6
05012	Parkinson disease	4.910779982	8.73275E-10	3.1719E-09	11540	Adora2a
05332	Graft-versus-host disease	10.42891172	1.13082E-09	4.06069E-09	16193	Il6
05332	Graft-versus-host disease	10.42891172	1.13082E-09	4.06069E-09	12524	Cd86
05332	Graft-versus-host disease	10.42891172	1.13082E-09	4.06069E-09	16175	Il1a
05332	Graft-versus-host disease	10.42891172	1.13082E-09	4.06069E-09	15039	H2-T22
05332	Graft-versus-host disease	10.42891172	1.13082E-09	4.06069E-09	16176	Il1b
05332	Graft-versus-host disease	10.42891172	1.13082E-09	4.06069E-09	15024	H2-T10
05332	Graft-versus-host disease	10.42891172	1.13082E-09	4.06069E-09	15001	H2-Oa
05332	Graft-versus-host disease	10.42891172	1.13082E-09	4.06069E-09	110558	H2-Q9

05332	Graft-versus-host disease	10.42891172	1.13082E-09	4.06069E-09	21926	Tnf
05332	Graft-versus-host disease	10.42891172	1.13082E-09	4.06069E-09	14990	H2-M2
05332	Graft-versus-host disease	10.42891172	1.13082E-09	4.06069E-09	15978	Ifng
05332	Graft-versus-host disease	10.42891172	1.13082E-09	4.06069E-09	14102	Fas
05332	Graft-versus-host disease	10.42891172	1.13082E-09	4.06069E-09	15018	H2-Q7
05410	Hypertrophic cardiomyopathy	8.33078747	1.39758E-09	4.96221E-09	13614	Edn1
05410	Hypertrophic cardiomyopathy	8.33078747	1.39758E-09	4.96221E-09	105787	Prkaa1
05410	Hypertrophic cardiomyopathy	8.33078747	1.39758E-09	4.96221E-09	17868	Mybpc3
05410	Hypertrophic cardiomyopathy	8.33078747	1.39758E-09	4.96221E-09	21926	Tnf
05410	Hypertrophic cardiomyopathy	8.33078747	1.39758E-09	4.96221E-09	320910	Itgb8
05410	Hypertrophic cardiomyopathy	8.33078747	1.39758E-09	4.96221E-09	22138	Ttn
05410	Hypertrophic cardiomyopathy	8.33078747	1.39758E-09	4.96221E-09	319480	Itga11
05410	Hypertrophic cardiomyopathy	8.33078747	1.39758E-09	4.96221E-09	16193	Il6
05410	Hypertrophic cardiomyopathy	8.33078747	1.39758E-09	4.96221E-09	326618	Tpm4
05410	Hypertrophic cardiomyopathy	8.33078747	1.39758E-09	4.96221E-09	81905	Cacng8
05410	Hypertrophic cardiomyopathy	8.33078747	1.39758E-09	4.96221E-09	16402	Itga5
05410	Hypertrophic cardiomyopathy	8.33078747	1.39758E-09	4.96221E-09	108079	Prkaa2
05410	Hypertrophic cardiomyopathy	8.33078747	1.39758E-09	4.96221E-09	54378	Cacng6
05410	Hypertrophic cardiomyopathy	8.33078747	1.39758E-09	4.96221E-09	104099	Itga9
05410	Hypertrophic cardiomyopathy	8.33078747	1.39758E-09	4.96221E-09	109700	Itga1
01200	Carbon metabolism	6.928886138	1.87959E-09	6.59946E-09	18655	Pgk1
01200	Carbon metabolism	6.928886138	1.87959E-09	6.59946E-09	18648	Pgam1
01200	Carbon metabolism	6.928886138	1.87959E-09	6.59946E-09	110460	Acat2
01200	Carbon metabolism	6.928886138	1.87959E-09	6.59946E-09	224530	Acat3
01200	Carbon metabolism	6.928886138	1.87959E-09	6.59946E-09	110208	Pgd
01200	Carbon metabolism	6.928886138	1.87959E-09	6.59946E-09	236539	Phgdh
01200	Carbon metabolism	6.928886138	1.87959E-09	6.59946E-09	107272	Psat1
01200	Carbon metabolism	6.928886138	1.87959E-09	6.59946E-09	18563	Pcx
01200	Carbon metabolism	6.928886138	1.87959E-09	6.59946E-09	15277	Hk2
01200	Carbon metabolism	6.928886138	1.87959E-09	6.59946E-09	18641	Pfkl
01200	Carbon metabolism	6.928886138	1.87959E-09	6.59946E-09	14381	G6pdx
01200	Carbon metabolism	6.928886138	1.87959E-09	6.59946E-09	433182	Eno1b
01200	Carbon metabolism	6.928886138	1.87959E-09	6.59946E-09	13885	Esd
01200	Carbon metabolism	6.928886138	1.87959E-09	6.59946E-09	14433	Gapdh
01200	Carbon metabolism	6.928886138	1.87959E-09	6.59946E-09	17448	Mdh2
01200	Carbon metabolism	6.928886138	1.87959E-09	6.59946E-09	21991	Tpi1
01200	Carbon metabolism	6.928886138	1.87959E-09	6.59946E-09	13806	Eno1
04350	TGF-beta signaling pathway	7.980017471	2.6132E-09	9.07441E-09	12166	Bmpr1a
04350	TGF-beta signaling pathway	7.980017471	2.6132E-09	9.07441E-09	15978	Ifng
04350	TGF-beta signaling pathway	7.980017471	2.6132E-09	9.07441E-09	17869	Myc
04350	TGF-beta signaling pathway	7.980017471	2.6132E-09	9.07441E-09	19053	Ppp2cb
04350	TGF-beta signaling pathway	7.980017471	2.6132E-09	9.07441E-09	21825	Thbs1

04350	TGF-beta signaling pathway	7.980017471	2.6132E-09	9.07441E-09	12156	Bmp2
04350	TGF-beta signaling pathway	7.980017471	2.6132E-09	9.07441E-09	16323	Inhba
04350	TGF-beta signaling pathway	7.980017471	2.6132E-09	9.07441E-09	11479	Acvr1b
04350	TGF-beta signaling pathway	7.980017471	2.6132E-09	9.07441E-09	12579	Cdkn2b
04350	TGF-beta signaling pathway	7.980017471	2.6132E-09	9.07441E-09	13179	Dcn
04350	TGF-beta signaling pathway	7.980017471	2.6132E-09	9.07441E-09	23893	Grem2
04350	TGF-beta signaling pathway	7.980017471	2.6132E-09	9.07441E-09	21926	Tnf
04350	TGF-beta signaling pathway	7.980017471	2.6132E-09	9.07441E-09	268977	Ltbp1
04350	TGF-beta signaling pathway	7.980017471	2.6132E-09	9.07441E-09	15902	Id2
04350	TGF-beta signaling pathway	7.980017471	2.6132E-09	9.07441E-09	14313	Fst
04068	FoxO signaling pathway	6.558640313	4.47755E-09	1.53794E-08	22035	Tnfsf10
04068	FoxO signaling pathway	6.558640313	4.47755E-09	1.53794E-08	18708	Pik3r1
04068	FoxO signaling pathway	6.558640313	4.47755E-09	1.53794E-08	17873	Gadd45b
04068	FoxO signaling pathway	6.558640313	4.47755E-09	1.53794E-08	12579	Cdkn2b
04068	FoxO signaling pathway	6.558640313	4.47755E-09	1.53794E-08	12575	Cdkn1a
04068	FoxO signaling pathway	6.558640313	4.47755E-09	1.53794E-08	105787	Prkaa1
04068	FoxO signaling pathway	6.558640313	4.47755E-09	1.53794E-08	13649	Egfr
04068	FoxO signaling pathway	6.558640313	4.47755E-09	1.53794E-08	12444	Ccnd2
04068	FoxO signaling pathway	6.558640313	4.47755E-09	1.53794E-08	16193	Il6
04068	FoxO signaling pathway	6.558640313	4.47755E-09	1.53794E-08	13197	Gadd45a
04068	FoxO signaling pathway	6.558640313	4.47755E-09	1.53794E-08	26415	Mapk13
04068	FoxO signaling pathway	6.558640313	4.47755E-09	1.53794E-08	12176	Bnip3
04068	FoxO signaling pathway	6.558640313	4.47755E-09	1.53794E-08	12795	Plk3
04068	FoxO signaling pathway	6.558640313	4.47755E-09	1.53794E-08	108079	Prkaa2
04068	FoxO signaling pathway	6.558640313	4.47755E-09	1.53794E-08	20656	Sod2
04068	FoxO signaling pathway	6.558640313	4.47755E-09	1.53794E-08	16153	Il10
04068	FoxO signaling pathway	6.558640313	4.47755E-09	1.53794E-08	20620	Plk2
04072	Phospholipase D signaling pathway	6.105516723	4.76015E-09	1.61743E-08	67916	Plpp3
04072	Phospholipase D signaling pathway	6.105516723	4.76015E-09	1.61743E-08	380921	Dgkh
04072	Phospholipase D signaling pathway	6.105516723	4.76015E-09	1.61743E-08	56717	Mtor
04072	Phospholipase D signaling pathway	6.105516723	4.76015E-09	1.61743E-08	19012	Plpp1
04072	Phospholipase D signaling pathway	6.105516723	4.76015E-09	1.61743E-08	18720	Pip5k1a
04072	Phospholipase D signaling pathway	6.105516723	4.76015E-09	1.61743E-08	14745	Lpar1
04072	Phospholipase D signaling pathway	6.105516723	4.76015E-09	1.61743E-08	18719	Pip5k1b
04072	Phospholipase D signaling pathway	6.105516723	4.76015E-09	1.61743E-08	18798	Plcb4
04072	Phospholipase D signaling pathway	6.105516723	4.76015E-09	1.61743E-08	20963	Syk
04072	Phospholipase D signaling pathway	6.105516723	4.76015E-09	1.61743E-08	14062	F2r
04072	Phospholipase D signaling pathway	6.105516723	4.76015E-09	1.61743E-08	20698	Sphk1
04072	Phospholipase D signaling pathway	6.105516723	4.76015E-09	1.61743E-08	320207	Pik3r5
04072	Phospholipase D signaling pathway	6.105516723	4.76015E-09	1.61743E-08	68262	Agpat4
04072	Phospholipase D signaling pathway	6.105516723	4.76015E-09	1.61743E-08	30955	Pik3cg
04072	Phospholipase D signaling pathway	6.105516723	4.76015E-09	1.61743E-08	18783	Pla2g4a

04072	Phospholipase D signaling pathway	6.105516723	4.76015E-09	1.61743E-08	13649	Egfr
04072	Phospholipase D signaling pathway	6.105516723	4.76015E-09	1.61743E-08	19730	Ralgds
04072	Phospholipase D signaling pathway	6.105516723	4.76015E-09	1.61743E-08	18708	Pik3r1
04670	Leukocyte transendothelial migration	6.852896359	7.1504E-09	2.40375E-08	22323	Vasp
04670	Leukocyte transendothelial migration	6.852896359	7.1504E-09	2.40375E-08	13058	Cybb
04670	Leukocyte transendothelial migration	6.852896359	7.1504E-09	2.40375E-08	26415	Mapk13
04670	Leukocyte transendothelial migration	6.852896359	7.1504E-09	2.40375E-08	17698	Msn
04670	Leukocyte transendothelial migration	6.852896359	7.1504E-09	2.40375E-08	215449	Rap1b
04670	Leukocyte transendothelial migration	6.852896359	7.1504E-09	2.40375E-08	17395	Mmp9
04670	Leukocyte transendothelial migration	6.852896359	7.1504E-09	2.40375E-08	22330	Vcl
04670	Leukocyte transendothelial migration	6.852896359	7.1504E-09	2.40375E-08	16408	Itgal
04670	Leukocyte transendothelial migration	6.852896359	7.1504E-09	2.40375E-08	12562	Cdh5
04670	Leukocyte transendothelial migration	6.852896359	7.1504E-09	2.40375E-08	12927	Bcar1
04670	Leukocyte transendothelial migration	6.852896359	7.1504E-09	2.40375E-08	69524	Esam
04670	Leukocyte transendothelial migration	6.852896359	7.1504E-09	2.40375E-08	22329	Vcam1
04670	Leukocyte transendothelial migration	6.852896359	7.1504E-09	2.40375E-08	22350	Ezr
04670	Leukocyte transendothelial migration	6.852896359	7.1504E-09	2.40375E-08	18708	Pik3r1
04670	Leukocyte transendothelial migration	6.852896359	7.1504E-09	2.40375E-08	17390	Mmp2
04670	Leukocyte transendothelial migration	6.852896359	7.1504E-09	2.40375E-08	15894	Icam1
04921	Oxytocin signaling pathway	5.945895371	7.32982E-09	2.43813E-08	320207	Pik3r5
04921	Oxytocin signaling pathway	5.945895371	7.32982E-09	2.43813E-08	18783	Pla2g4a
04921	Oxytocin signaling pathway	5.945895371	7.32982E-09	2.43813E-08	228785	Mylk2
04921	Oxytocin signaling pathway	5.945895371	7.32982E-09	2.43813E-08	20779	Src
04921	Oxytocin signaling pathway	5.945895371	7.32982E-09	2.43813E-08	18798	Plcb4
04921	Oxytocin signaling pathway	5.945895371	7.32982E-09	2.43813E-08	81905	Cacng8
04921	Oxytocin signaling pathway	5.945895371	7.32982E-09	2.43813E-08	13649	Egfr
04921	Oxytocin signaling pathway	5.945895371	7.32982E-09	2.43813E-08	28240	Trpm2
04921	Oxytocin signaling pathway	5.945895371	7.32982E-09	2.43813E-08	19057	Ppp3cc
04921	Oxytocin signaling pathway	5.945895371	7.32982E-09	2.43813E-08	54378	Cacng6
04921	Oxytocin signaling pathway	5.945895371	7.32982E-09	2.43813E-08	19225	Ptgs2
04921	Oxytocin signaling pathway	5.945895371	7.32982E-09	2.43813E-08	108058	Camk2d
04921	Oxytocin signaling pathway	5.945895371	7.32982E-09	2.43813E-08	30955	Pik3cg
04921	Oxytocin signaling pathway	5.945895371	7.32982E-09	2.43813E-08	108079	Prkaa2
04921	Oxytocin signaling pathway	5.945895371	7.32982E-09	2.43813E-08	105787	Prkaa1
04921	Oxytocin signaling pathway	5.945895371	7.32982E-09	2.43813E-08	12575	Cdkn1a
04921	Oxytocin signaling pathway	5.945895371	7.32982E-09	2.43813E-08	329251	Ppp1r12b
04921	Oxytocin signaling pathway	5.945895371	7.32982E-09	2.43813E-08	12494	Cd38
05416	Viral myocarditis	8.040472149	8.72876E-09	2.87322E-08	14990	H2-M2
05416	Viral myocarditis	8.040472149	8.72876E-09	2.87322E-08	21939	Cd40
05416	Viral myocarditis	8.040472149	8.72876E-09	2.87322E-08	15001	H2-Oa
05416	Viral myocarditis	8.040472149	8.72876E-09	2.87322E-08	13690	Eif4g2
05416	Viral myocarditis	8.040472149	8.72876E-09	2.87322E-08	110558	H2-Q9

05416	Viral myocarditis	8.040472149	8.72876E-09	2.87322E-08	15894	Icam1
05416	Viral myocarditis	8.040472149	8.72876E-09	2.87322E-08	13063	Cycs
05416	Viral myocarditis	8.040472149	8.72876E-09	2.87322E-08	12389	Cav1
05416	Viral myocarditis	8.040472149	8.72876E-09	2.87322E-08	12122	Bid
05416	Viral myocarditis	8.040472149	8.72876E-09	2.87322E-08	12524	Cd86
05416	Viral myocarditis	8.040472149	8.72876E-09	2.87322E-08	16408	Itgal
05416	Viral myocarditis	8.040472149	8.72876E-09	2.87322E-08	15024	H2-T10
05416	Viral myocarditis	8.040472149	8.72876E-09	2.87322E-08	15039	H2-T22
05416	Viral myocarditis	8.040472149	8.72876E-09	2.87322E-08	15018	H2-Q7
00100	Steroid biosynthesis	20.21604426	9.39981E-09	3.06221E-08	223920	Soat2
00100	Steroid biosynthesis	20.21604426	9.39981E-09	3.06221E-08	235293	Sc5d
00100	Steroid biosynthesis	20.21604426	9.39981E-09	3.06221E-08	14137	Fdft1
00100	Steroid biosynthesis	20.21604426	9.39981E-09	3.06221E-08	15490	Hsd17b7
00100	Steroid biosynthesis	20.21604426	9.39981E-09	3.06221E-08	13121	Cyp51
00100	Steroid biosynthesis	20.21604426	9.39981E-09	3.06221E-08	74754	Dhcr24
00100	Steroid biosynthesis	20.21604426	9.39981E-09	3.06221E-08	20775	Sqle
00100	Steroid biosynthesis	20.21604426	9.39981E-09	3.06221E-08	66234	Msmo1
04722	Neurotrophin signaling pathway	6.682989838	1.03923E-08	3.35097E-08	108960	Irak2
04722	Neurotrophin signaling pathway	6.682989838	1.03923E-08	3.35097E-08	18049	Ngf
04722	Neurotrophin signaling pathway	6.682989838	1.03923E-08	3.35097E-08	20112	Rps6ka2
04722	Neurotrophin signaling pathway	6.682989838	1.03923E-08	3.35097E-08	192656	Ripk2
04722	Neurotrophin signaling pathway	6.682989838	1.03923E-08	3.35097E-08	26415	Mapk13
04722	Neurotrophin signaling pathway	6.682989838	1.03923E-08	3.35097E-08	11911	Atf4
04722	Neurotrophin signaling pathway	6.682989838	1.03923E-08	3.35097E-08	18708	Pik3r1
04722	Neurotrophin signaling pathway	6.682989838	1.03923E-08	3.35097E-08	73914	Irak3
04722	Neurotrophin signaling pathway	6.682989838	1.03923E-08	3.35097E-08	18033	Nfkb1
04722	Neurotrophin signaling pathway	6.682989838	1.03923E-08	3.35097E-08	17164	Mapkapk2
04722	Neurotrophin signaling pathway	6.682989838	1.03923E-08	3.35097E-08	108058	Camk2d
04722	Neurotrophin signaling pathway	6.682989838	1.03923E-08	3.35097E-08	18036	Nfkbib
04722	Neurotrophin signaling pathway	6.682989838	1.03923E-08	3.35097E-08	18035	Nfkbia
04722	Neurotrophin signaling pathway	6.682989838	1.03923E-08	3.35097E-08	107746	Rapgef1
04722	Neurotrophin signaling pathway	6.682989838	1.03923E-08	3.35097E-08	18037	Nfkbie
04722	Neurotrophin signaling pathway	6.682989838	1.03923E-08	3.35097E-08	215449	Rap1b
04611	Platelet activation	6.5213046	1.49353E-08	4.76722E-08	22323	Vasp
04611	Platelet activation	6.5213046	1.49353E-08	4.76722E-08	18708	Pik3r1
04611	Platelet activation	6.5213046	1.49353E-08	4.76722E-08	228785	Mylk2
04611	Platelet activation	6.5213046	1.49353E-08	4.76722E-08	18783	Pla2g4a
04611	Platelet activation	6.5213046	1.49353E-08	4.76722E-08	18798	Plcb4
04611	Platelet activation	6.5213046	1.49353E-08	4.76722E-08	20963	Syk
04611	Platelet activation	6.5213046	1.49353E-08	4.76722E-08	320207	Pik3r5
04611	Platelet activation	6.5213046	1.49353E-08	4.76722E-08	215449	Rap1b
04611	Platelet activation	6.5213046	1.49353E-08	4.76722E-08	19419	Rasgrp1

04611	Platelet activation	6.5213046	1.49353E-08	4.76722E-08	16822	Lcp2
04611	Platelet activation	6.5213046	1.49353E-08	4.76722E-08	26415	Mapk13
04611	Platelet activation	6.5213046	1.49353E-08	4.76722E-08	17096	Lyn
04611	Platelet activation	6.5213046	1.49353E-08	4.76722E-08	14062	F2r
04611	Platelet activation	6.5213046	1.49353E-08	4.76722E-08	30955	Pik3cg
04611	Platelet activation	6.5213046	1.49353E-08	4.76722E-08	20779	Src
04611	Platelet activation	6.5213046	1.49353E-08	4.76722E-08	243816	Gp6
05219	Bladder cancer	12.32685626	2.29028E-08	7.23729E-08	83996	Mmp1b
05219	Bladder cancer	12.32685626	2.29028E-08	7.23729E-08	13649	Egfr
05219	Bladder cancer	12.32685626	2.29028E-08	7.23729E-08	17395	Mmp9
05219	Bladder cancer	12.32685626	2.29028E-08	7.23729E-08	20779	Src
05219	Bladder cancer	12.32685626	2.29028E-08	7.23729E-08	17869	Myc
05219	Bladder cancer	12.32685626	2.29028E-08	7.23729E-08	21825	Thbs1
05219	Bladder cancer	12.32685626	2.29028E-08	7.23729E-08	22339	Vegfa
05219	Bladder cancer	12.32685626	2.29028E-08	7.23729E-08	15200	Hbegf
05219	Bladder cancer	12.32685626	2.29028E-08	7.23729E-08	17390	Mmp2
05219	Bladder cancer	12.32685626	2.29028E-08	7.23729E-08	12575	Cdkn1a
00010	Glycolysis / Gluconeogenesis	9.051960116	3.02973E-08	9.47916E-08	21991	Tpi1
00010	Glycolysis / Gluconeogenesis	9.051960116	3.02973E-08	9.47916E-08	66681	Pgm2
00010	Glycolysis / Gluconeogenesis	9.051960116	3.02973E-08	9.47916E-08	11670	Aldh3a1
00010	Glycolysis / Gluconeogenesis	9.051960116	3.02973E-08	9.47916E-08	18641	Pfkl
00010	Glycolysis / Gluconeogenesis	9.051960116	3.02973E-08	9.47916E-08	18648	Pgam1
00010	Glycolysis / Gluconeogenesis	9.051960116	3.02973E-08	9.47916E-08	13806	Eno1
00010	Glycolysis / Gluconeogenesis	9.051960116	3.02973E-08	9.47916E-08	433182	Eno1b
00010	Glycolysis / Gluconeogenesis	9.051960116	3.02973E-08	9.47916E-08	14433	Gapdh
00010	Glycolysis / Gluconeogenesis	9.051960116	3.02973E-08	9.47916E-08	15277	Hk2
00010	Glycolysis / Gluconeogenesis	9.051960116	3.02973E-08	9.47916E-08	18655	Pgk1
00010	Glycolysis / Gluconeogenesis	9.051960116	3.02973E-08	9.47916E-08	16828	Ldha
00010	Glycolysis / Gluconeogenesis	9.051960116	3.02973E-08	9.47916E-08	11529	Adh7
05211	Renal cell carcinoma	8.918843056	3.61269E-08	1.11923E-07	215449	Rap1b
05211	Renal cell carcinoma	8.918843056	3.61269E-08	1.11923E-07	12575	Cdkn1a
05211	Renal cell carcinoma	8.918843056	3.61269E-08	1.11923E-07	18708	Pik3r1
05211	Renal cell carcinoma	8.918843056	3.61269E-08	1.11923E-07	15251	Hif1a
05211	Renal cell carcinoma	8.918843056	3.61269E-08	1.11923E-07	107746	Rapgef1
05211	Renal cell carcinoma	8.918843056	3.61269E-08	1.11923E-07	22339	Vegfa
05211	Renal cell carcinoma	8.918843056	3.61269E-08	1.11923E-07	112407	Egln3
05211	Renal cell carcinoma	8.918843056	3.61269E-08	1.11923E-07	20525	Slc2a1
05211	Renal cell carcinoma	8.918843056	3.61269E-08	1.11923E-07	23871	Ets1
05211	Renal cell carcinoma	8.918843056	3.61269E-08	1.11923E-07	17295	Met
05211	Renal cell carcinoma	8.918843056	3.61269E-08	1.11923E-07	11864	Arnt2
05211	Renal cell carcinoma	8.918843056	3.61269E-08	1.11923E-07	67923	Eloc
05215	Prostate cancer	7.147086355	4.21457E-08	1.29301E-07	17395	Mmp9

05215	Prostate cancer	7.147086355	4.21457E-08	1.29301E-07	18708	Pik3r1
05215	Prostate cancer	7.147086355	4.21457E-08	1.29301E-07	18792	Plau
05215	Prostate cancer	7.147086355	4.21457E-08	1.29301E-07	11911	Atf4
05215	Prostate cancer	7.147086355	4.21457E-08	1.29301E-07	12575	Cdkn1a
05215	Prostate cancer	7.147086355	4.21457E-08	1.29301E-07	17392	Mmp3
05215	Prostate cancer	7.147086355	4.21457E-08	1.29301E-07	15519	Hsp90aa1
05215	Prostate cancer	7.147086355	4.21457E-08	1.29301E-07	231991	Creb5
05215	Prostate cancer	7.147086355	4.21457E-08	1.29301E-07	18035	Nfkbia
05215	Prostate cancer	7.147086355	4.21457E-08	1.29301E-07	13649	Egfr
05215	Prostate cancer	7.147086355	4.21457E-08	1.29301E-07	21417	Zeb1
05215	Prostate cancer	7.147086355	4.21457E-08	1.29301E-07	18033	Nfkb1
05215	Prostate cancer	7.147086355	4.21457E-08	1.29301E-07	22027	Hsp90b1
05215	Prostate cancer	7.147086355	4.21457E-08	1.29301E-07	56717	Mtor
04024	cAMP signaling pathway	4.701405642	5.70498E-08	1.72286E-07	15551	Htr1b
04024	cAMP signaling pathway	4.701405642	5.70498E-08	1.72286E-07	233079	Ffar2
04024	cAMP signaling pathway	4.701405642	5.70498E-08	1.72286E-07	18708	Pik3r1
04024	cAMP signaling pathway	4.701405642	5.70498E-08	1.72286E-07	108058	Camk2d
04024	cAMP signaling pathway	4.701405642	5.70498E-08	1.72286E-07	381853	Gipr
04024	cAMP signaling pathway	4.701405642	5.70498E-08	1.72286E-07	381290	Atp2b4
04024	cAMP signaling pathway	4.701405642	5.70498E-08	1.72286E-07	23984	Pde10a
04024	cAMP signaling pathway	4.701405642	5.70498E-08	1.72286E-07	11540	Adora2a
04024	cAMP signaling pathway	4.701405642	5.70498E-08	1.72286E-07	80885	Hcar2
04024	cAMP signaling pathway	4.701405642	5.70498E-08	1.72286E-07	231991	Creb5
04024	cAMP signaling pathway	4.701405642	5.70498E-08	1.72286E-07	15557	Htr1f
04024	cAMP signaling pathway	4.701405642	5.70498E-08	1.72286E-07	14814	Grin2d
04024	cAMP signaling pathway	4.701405642	5.70498E-08	1.72286E-07	215449	Rap1b
04024	cAMP signaling pathway	4.701405642	5.70498E-08	1.72286E-07	14062	F2r
04024	cAMP signaling pathway	4.701405642	5.70498E-08	1.72286E-07	13614	Edn1
04024	cAMP signaling pathway	4.701405642	5.70498E-08	1.72286E-07	18035	Nfkbia
04024	cAMP signaling pathway	4.701405642	5.70498E-08	1.72286E-07	238871	Pde4d
04024	cAMP signaling pathway	4.701405642	5.70498E-08	1.72286E-07	18033	Nfkb1
04024	cAMP signaling pathway	4.701405642	5.70498E-08	1.72286E-07	18578	Pde4b
04024	cAMP signaling pathway	4.701405642	5.70498E-08	1.72286E-07	239273	Abcc4
05225	Hepatocellular carcinoma	5.228287309	5.7247E-08	1.72286E-07	100042295	Gm3776
05225	Hepatocellular carcinoma	5.228287309	5.7247E-08	1.72286E-07	14362	Fzd1
05225	Hepatocellular carcinoma	5.228287309	5.7247E-08	1.72286E-07	14860	Gsta4
05225	Hepatocellular carcinoma	5.228287309	5.7247E-08	1.72286E-07	14858	Gsta2
05225	Hepatocellular carcinoma	5.228287309	5.7247E-08	1.72286E-07	17873	Gadd45b
05225	Hepatocellular carcinoma	5.228287309	5.7247E-08	1.72286E-07	14857	Gsta1
05225	Hepatocellular carcinoma	5.228287309	5.7247E-08	1.72286E-07	18708	Pik3r1
05225	Hepatocellular carcinoma	5.228287309	5.7247E-08	1.72286E-07	17295	Met
05225	Hepatocellular carcinoma	5.228287309	5.7247E-08	1.72286E-07	14863	Gstm2

05225	Hepatocellular carcinoma	5.228287309	5.7247E-08	1.72286E-07	22420	Wnt6
05225	Hepatocellular carcinoma	5.228287309	5.7247E-08	1.72286E-07	18104	Nqo1
05225	Hepatocellular carcinoma	5.228287309	5.7247E-08	1.72286E-07	15368	Hmox1
05225	Hepatocellular carcinoma	5.228287309	5.7247E-08	1.72286E-07	13197	Gadd45a
05225	Hepatocellular carcinoma	5.228287309	5.7247E-08	1.72286E-07	50493	Txnrd1
05225	Hepatocellular carcinoma	5.228287309	5.7247E-08	1.72286E-07	13649	Egfr
05225	Hepatocellular carcinoma	5.228287309	5.7247E-08	1.72286E-07	56717	Mtor
05225	Hepatocellular carcinoma	5.228287309	5.7247E-08	1.72286E-07	17869	Myc
05225	Hepatocellular carcinoma	5.228287309	5.7247E-08	1.72286E-07	12575	Cdkn1a
04115	p53 signaling pathway	8.423351775	7.08757E-08	2.1129E-07	17873	Gadd45b
04115	p53 signaling pathway	8.423351775	7.08757E-08	2.1129E-07	12521	Cd82
04115	p53 signaling pathway	8.423351775	7.08757E-08	2.1129E-07	12122	Bid
04115	p53 signaling pathway	8.423351775	7.08757E-08	2.1129E-07	13063	Cycs
04115	p53 signaling pathway	8.423351775	7.08757E-08	2.1129E-07	12444	Ccnd2
04115	p53 signaling pathway	8.423351775	7.08757E-08	2.1129E-07	26374	Cop1
04115	p53 signaling pathway	8.423351775	7.08757E-08	2.1129E-07	21933	Tnfrsf10b
04115	p53 signaling pathway	8.423351775	7.08757E-08	2.1129E-07	14102	Fas
04115	p53 signaling pathway	8.423351775	7.08757E-08	2.1129E-07	13197	Gadd45a
04115	p53 signaling pathway	8.423351775	7.08757E-08	2.1129E-07	12575	Cdkn1a
04115	p53 signaling pathway	8.423351775	7.08757E-08	2.1129E-07	21825	Thbs1
04115	p53 signaling pathway	8.423351775	7.08757E-08	2.1129E-07	55948	Sfn
04650	Natural killer cell mediated cytotoxicity	6.265302973	7.92067E-08	2.33919E-07	15894	Icam1
04650	Natural killer cell mediated cytotoxicity	6.265302973	7.92067E-08	2.33919E-07	15978	Ifng
04650	Natural killer cell mediated cytotoxicity	6.265302973	7.92067E-08	2.33919E-07	16822	Lcp2
04650	Natural killer cell mediated cytotoxicity	6.265302973	7.92067E-08	2.33919E-07	21933	Tnfrsf10b
04650	Natural killer cell mediated cytotoxicity	6.265302973	7.92067E-08	2.33919E-07	20963	Syk
04650	Natural killer cell mediated cytotoxicity	6.265302973	7.92067E-08	2.33919E-07	12122	Bid
04650	Natural killer cell mediated cytotoxicity	6.265302973	7.92067E-08	2.33919E-07	12503	Cd247
04650	Natural killer cell mediated cytotoxicity	6.265302973	7.92067E-08	2.33919E-07	18708	Pik3r1
04650	Natural killer cell mediated cytotoxicity	6.265302973	7.92067E-08	2.33919E-07	22035	Tnfsf10
04650	Natural killer cell mediated cytotoxicity	6.265302973	7.92067E-08	2.33919E-07	12981	Csf2
04650	Natural killer cell mediated cytotoxicity	6.265302973	7.92067E-08	2.33919E-07	21926	Tnf
04650	Natural killer cell mediated cytotoxicity	6.265302973	7.92067E-08	2.33919E-07	16797	Lat
04650	Natural killer cell mediated cytotoxicity	6.265302973	7.92067E-08	2.33919E-07	16408	Itgal
04650	Natural killer cell mediated cytotoxicity	6.265302973	7.92067E-08	2.33919E-07	14102	Fas
04650	Natural killer cell mediated cytotoxicity	6.265302973	7.92067E-08	2.33919E-07	19057	Ppp3cc
04512	ECM-receptor interaction	7.46615271	8.21283E-08	2.40301E-07	109700	Itga1
04512	ECM-receptor interaction	7.46615271	8.21283E-08	2.40301E-07	104099	Itga9
04512	ECM-receptor interaction	7.46615271	8.21283E-08	2.40301E-07	16782	Lamc2
04512	ECM-receptor interaction	7.46615271	8.21283E-08	2.40301E-07	320910	Itgb8
04512	ECM-receptor interaction	7.46615271	8.21283E-08	2.40301E-07	12827	Col4a2
04512	ECM-receptor interaction	7.46615271	8.21283E-08	2.40301E-07	319480	Itga11

04512	ECM-receptor interaction	7.46615271	8.21283E-08	2.40301E-07	20971	Sdc4
04512	ECM-receptor interaction	7.46615271	8.21283E-08	2.40301E-07	16402	Itga5
04512	ECM-receptor interaction	7.46615271	8.21283E-08	2.40301E-07	329278	Tnn
04512	ECM-receptor interaction	7.46615271	8.21283E-08	2.40301E-07	21825	Thbs1
04512	ECM-receptor interaction	7.46615271	8.21283E-08	2.40301E-07	243816	Gp6
04512	ECM-receptor interaction	7.46615271	8.21283E-08	2.40301E-07	20969	Sdc1
04512	ECM-receptor interaction	7.46615271	8.21283E-08	2.40301E-07	21923	Tnc
04917	Prolactin signaling pathway	8.195693619	9.76232E-08	2.83018E-07	18708	Pik3r1
04917	Prolactin signaling pathway	8.195693619	9.76232E-08	2.83018E-07	16452	Jak2
04917	Prolactin signaling pathway	8.195693619	9.76232E-08	2.83018E-07	20779	Src
04917	Prolactin signaling pathway	8.195693619	9.76232E-08	2.83018E-07	216233	Socs2
04917	Prolactin signaling pathway	8.195693619	9.76232E-08	2.83018E-07	18033	Nfkb1
04917	Prolactin signaling pathway	8.195693619	9.76232E-08	2.83018E-07	12700	Cish
04917	Prolactin signaling pathway	8.195693619	9.76232E-08	2.83018E-07	20846	Stat1
04917	Prolactin signaling pathway	8.195693619	9.76232E-08	2.83018E-07	12703	Socs1
04917	Prolactin signaling pathway	8.195693619	9.76232E-08	2.83018E-07	26415	Mapk13
04917	Prolactin signaling pathway	8.195693619	9.76232E-08	2.83018E-07	16362	Irf1
04917	Prolactin signaling pathway	8.195693619	9.76232E-08	2.83018E-07	12444	Ccnd2
04917	Prolactin signaling pathway	8.195693619	9.76232E-08	2.83018E-07	12702	Socs3
04928	Parathyroid hormone synthesis, secretion and action	6.551495825	1.31409E-07	3.77503E-07	238871	Pde4d
04928	Parathyroid hormone synthesis, secretion and action	6.551495825	1.31409E-07	3.77503E-07	13653	Egr1
04928	Parathyroid hormone synthesis, secretion and action	6.551495825	1.31409E-07	3.77503E-07	22337	Vdr
04928	Parathyroid hormone synthesis, secretion and action	6.551495825	1.31409E-07	3.77503E-07	13649	Egfr
04928	Parathyroid hormone synthesis, secretion and action	6.551495825	1.31409E-07	3.77503E-07	15200	Hbegf
04928	Parathyroid hormone synthesis, secretion and action	6.551495825	1.31409E-07	3.77503E-07	17386	Mmp13
04928	Parathyroid hormone synthesis, secretion and action	6.551495825	1.31409E-07	3.77503E-07	18227	Nr4a2
04928	Parathyroid hormone synthesis, secretion and action	6.551495825	1.31409E-07	3.77503E-07	12575	Cdkn1a
04928	Parathyroid hormone synthesis, secretion and action	6.551495825	1.31409E-07	3.77503E-07	231991	Creb5
04928	Parathyroid hormone synthesis, secretion and action	6.551495825	1.31409E-07	3.77503E-07	17387	Mmp14
04928	Parathyroid hormone synthesis, secretion and action	6.551495825	1.31409E-07	3.77503E-07	18578	Pde4b
04928	Parathyroid hormone synthesis, secretion and action	6.551495825	1.31409E-07	3.77503E-07	11911	Atf4
04928	Parathyroid hormone synthesis, secretion and action	6.551495825	1.31409E-07	3.77503E-07	18798	Plcb4
04928	Parathyroid hormone synthesis, secretion and action	6.551495825	1.31409E-07	3.77503E-07	240047	Mmp25
04610	Complement and coagulation cascades	7.06474665	1.62067E-07	4.61379E-07	50908	C1s1
04610	Complement and coagulation cascades	7.06474665	1.62067E-07	4.61379E-07	18793	Plaur
04610	Complement and coagulation cascades	7.06474665	1.62067E-07	4.61379E-07	14066	F3
04610	Complement and coagulation cascades	7.06474665	1.62067E-07	4.61379E-07	667277	C1rb
04610	Complement and coagulation cascades	7.06474665	1.62067E-07	4.61379E-07	14058	F10
04610	Complement and coagulation cascades	7.06474665	1.62067E-07	4.61379E-07	14962	Cfb
04610	Complement and coagulation cascades	7.06474665	1.62067E-07	4.61379E-07	18792	Plau
04610	Complement and coagulation cascades	7.06474665	1.62067E-07	4.61379E-07	18788	Serpinb2
04610	Complement and coagulation cascades	7.06474665	1.62067E-07	4.61379E-07	14064	F2rl2

04610	Complement and coagulation cascades	7.06474665	1.62067E-07	4.61379E-07	12062	Bdkrb2
04610	Complement and coagulation cascades	7.06474665	1.62067E-07	4.61379E-07	14062	F2r
04610	Complement and coagulation cascades	7.06474665	1.62067E-07	4.61379E-07	12266	C3
04610	Complement and coagulation cascades	7.06474665	1.62067E-07	4.61379E-07	19124	Procr
04530	Tight junction	5.144801683	1.81686E-07	5.12614E-07	15163	Hcls1
04530	Tight junction	5.144801683	1.81686E-07	5.12614E-07	104027	Synpo
04530	Tight junction	5.144801683	1.81686E-07	5.12614E-07	12479	Cd1d1
04530	Tight junction	5.144801683	1.81686E-07	5.12614E-07	19053	Ppp2cb
04530	Tight junction	5.144801683	1.81686E-07	5.12614E-07	22350	Ezr
04530	Tight junction	5.144801683	1.81686E-07	5.12614E-07	22323	Vasp
04530	Tight junction	5.144801683	1.81686E-07	5.12614E-07	17698	Msn
04530	Tight junction	5.144801683	1.81686E-07	5.12614E-07	17880	Myh11
04530	Tight junction	5.144801683	1.81686E-07	5.12614E-07	75723	Amotl1
04530	Tight junction	5.144801683	1.81686E-07	5.12614E-07	231830	Micall2
04530	Tight junction	5.144801683	1.81686E-07	5.12614E-07	105787	Prkaa1
04530	Tight junction	5.144801683	1.81686E-07	5.12614E-07	22142	Tuba1a
04530	Tight junction	5.144801683	1.81686E-07	5.12614E-07	108079	Prkaa2
04530	Tight junction	5.144801683	1.81686E-07	5.12614E-07	20779	Src
04530	Tight junction	5.144801683	1.81686E-07	5.12614E-07	22145	Tuba4a
04530	Tight junction	5.144801683	1.81686E-07	5.12614E-07	76089	Rapgef2
04530	Tight junction	5.144801683	1.81686E-07	5.12614E-07	434204	Whamm
01524	Platinum drug resistance	7.581016598	2.40358E-07	6.72152E-07	12122	Bid
01524	Platinum drug resistance	7.581016598	2.40358E-07	6.72152E-07	14863	Gstm2
01524	Platinum drug resistance	7.581016598	2.40358E-07	6.72152E-07	14102	Fas
01524	Platinum drug resistance	7.581016598	2.40358E-07	6.72152E-07	100042295	Gm3776
01524	Platinum drug resistance	7.581016598	2.40358E-07	6.72152E-07	11796	Birc3
01524	Platinum drug resistance	7.581016598	2.40358E-07	6.72152E-07	12575	Cdkn1a
01524	Platinum drug resistance	7.581016598	2.40358E-07	6.72152E-07	13063	Cycs
01524	Platinum drug resistance	7.581016598	2.40358E-07	6.72152E-07	14857	Gsta1
01524	Platinum drug resistance	7.581016598	2.40358E-07	6.72152E-07	14858	Gsta2
01524	Platinum drug resistance	7.581016598	2.40358E-07	6.72152E-07	18708	Pik3r1
01524	Platinum drug resistance	7.581016598	2.40358E-07	6.72152E-07	14860	Gsta4
01524	Platinum drug resistance	7.581016598	2.40358E-07	6.72152E-07	20529	Slc31a1
04932	Non-alcoholic fatty liver disease	5.355243513	2.55912E-07	7.09371E-07	21926	Tnf
04932	Non-alcoholic fatty liver disease	5.355243513	2.55912E-07	7.09371E-07	12702	Socs3
04932	Non-alcoholic fatty liver disease	5.355243513	2.55912E-07	7.09371E-07	18708	Pik3r1
04932	Non-alcoholic fatty liver disease	5.355243513	2.55912E-07	7.09371E-07	16193	Il6
04932	Non-alcoholic fatty liver disease	5.355243513	2.55912E-07	7.09371E-07	108079	Prkaa2
04932	Non-alcoholic fatty liver disease	5.355243513	2.55912E-07	7.09371E-07	18033	Nfkb1
04932	Non-alcoholic fatty liver disease	5.355243513	2.55912E-07	7.09371E-07	105787	Prkaa1
04932	Non-alcoholic fatty liver disease	5.355243513	2.55912E-07	7.09371E-07	12122	Bid
04932	Non-alcoholic fatty liver disease	5.355243513	2.55912E-07	7.09371E-07	16176	Il1b

04932	Non-alcoholic fatty liver disease	5.355243513	2.55912E-07	7.09371E-07	16847	Lepr
04932	Non-alcoholic fatty liver disease	5.355243513	2.55912E-07	7.09371E-07	12369	Casp7
04932	Non-alcoholic fatty liver disease	5.355243513	2.55912E-07	7.09371E-07	13063	Cycs
04932	Non-alcoholic fatty liver disease	5.355243513	2.55912E-07	7.09371E-07	11911	Atf4
04932	Non-alcoholic fatty liver disease	5.355243513	2.55912E-07	7.09371E-07	16175	Il1a
04932	Non-alcoholic fatty liver disease	5.355243513	2.55912E-07	7.09371E-07	22030	Traf2
04932	Non-alcoholic fatty liver disease	5.355243513	2.55912E-07	7.09371E-07	14102	Fas
04662	B cell receptor signaling pathway	7.4874238	2.77085E-07	7.61381E-07	83490	Pik3ap1
04662	B cell receptor signaling pathway	7.4874238	2.77085E-07	7.61381E-07	20963	Syk
04662	B cell receptor signaling pathway	7.4874238	2.77085E-07	7.61381E-07	17096	Lyn
04662	B cell receptor signaling pathway	7.4874238	2.77085E-07	7.61381E-07	18033	Nfkb1
04662	B cell receptor signaling pathway	7.4874238	2.77085E-07	7.61381E-07	18036	Nfkbib
04662	B cell receptor signaling pathway	7.4874238	2.77085E-07	7.61381E-07	18035	Nfkbia
04662	B cell receptor signaling pathway	7.4874238	2.77085E-07	7.61381E-07	19057	Ppp3cc
04662	B cell receptor signaling pathway	7.4874238	2.77085E-07	7.61381E-07	68713	Ifitm1
04662	B cell receptor signaling pathway	7.4874238	2.77085E-07	7.61381E-07	18037	Nfkbie
04662	B cell receptor signaling pathway	7.4874238	2.77085E-07	7.61381E-07	14130	Fcgr2b
04662	B cell receptor signaling pathway	7.4874238	2.77085E-07	7.61381E-07	18708	Pik3r1
04662	B cell receptor signaling pathway	7.4874238	2.77085E-07	7.61381E-07	240354	Malt1
04141	Protein processing in endoplasmic reticulum	4.995243494	2.8053E-07	7.64201E-07	14828	Hspa5
04141	Protein processing in endoplasmic reticulum	4.995243494	2.8053E-07	7.64201E-07	71853	Pdia6
04141	Protein processing in endoplasmic reticulum	4.995243494	2.8053E-07	7.64201E-07	20335	Sec61g
04141	Protein processing in endoplasmic reticulum	4.995243494	2.8053E-07	7.64201E-07	18453	P4hb
04141	Protein processing in endoplasmic reticulum	4.995243494	2.8053E-07	7.64201E-07	13418	Dnajc1
04141	Protein processing in endoplasmic reticulum	4.995243494	2.8053E-07	7.64201E-07	66861	Dnajc10
04141	Protein processing in endoplasmic reticulum	4.995243494	2.8053E-07	7.64201E-07	12304	Pdia4
04141	Protein processing in endoplasmic reticulum	4.995243494	2.8053E-07	7.64201E-07	19106	Eif2ak2
04141	Protein processing in endoplasmic reticulum	4.995243494	2.8053E-07	7.64201E-07	22030	Traf2
04141	Protein processing in endoplasmic reticulum	4.995243494	2.8053E-07	7.64201E-07	18415	Hspa4l
04141	Protein processing in endoplasmic reticulum	4.995243494	2.8053E-07	7.64201E-07	12317	Calr
04141	Protein processing in endoplasmic reticulum	4.995243494	2.8053E-07	7.64201E-07	11911	Atf4
04141	Protein processing in endoplasmic reticulum	4.995243494	2.8053E-07	7.64201E-07	108159	Ubxn8
04141	Protein processing in endoplasmic reticulum	4.995243494	2.8053E-07	7.64201E-07	22027	Hsp90b1
04141	Protein processing in endoplasmic reticulum	4.995243494	2.8053E-07	7.64201E-07	17872	Ppp1r15a
04141	Protein processing in endoplasmic reticulum	4.995243494	2.8053E-07	7.64201E-07	12282	Hyou1
04141	Protein processing in endoplasmic reticulum	4.995243494	2.8053E-07	7.64201E-07	15519	Hsp90aa1
04935	Growth hormone synthesis, secretion and action	6.099668527	3.28804E-07	8.88053E-07	12927	Bcar1
04935	Growth hormone synthesis, secretion and action	6.099668527	3.28804E-07	8.88053E-07	26415	Mapk13
04935	Growth hormone synthesis, secretion and action	6.099668527	3.28804E-07	8.88053E-07	20846	Stat1
04935	Growth hormone synthesis, secretion and action	6.099668527	3.28804E-07	8.88053E-07	11911	Atf4
04935	Growth hormone synthesis, secretion and action	6.099668527	3.28804E-07	8.88053E-07	231991	Creb5
04935	Growth hormone synthesis, secretion and action	6.099668527	3.28804E-07	8.88053E-07	12702	Socs3

04935	Growth hormone synthesis, secretion and action	6.099668527	3.28804E-07	8.88053E-07	26399	Map2k6
04935	Growth hormone synthesis, secretion and action	6.099668527	3.28804E-07	8.88053E-07	16452	Jak2
04935	Growth hormone synthesis, secretion and action	6.099668527	3.28804E-07	8.88053E-07	12703	Socs1
04935	Growth hormone synthesis, secretion and action	6.099668527	3.28804E-07	8.88053E-07	16477	Junb
04935	Growth hormone synthesis, secretion and action	6.099668527	3.28804E-07	8.88053E-07	18798	Plcb4
04935	Growth hormone synthesis, secretion and action	6.099668527	3.28804E-07	8.88053E-07	216233	Socs2
04935	Growth hormone synthesis, secretion and action	6.099668527	3.28804E-07	8.88053E-07	18708	Pik3r1
04935	Growth hormone synthesis, secretion and action	6.099668527	3.28804E-07	8.88053E-07	56717	Mtor
01523	Antifolate resistance	13.47736284	3.66319E-07	9.80989E-07	16193	Il6
01523	Antifolate resistance	13.47736284	3.66319E-07	9.80989E-07	239273	Abcc4
01523	Antifolate resistance	13.47736284	3.66319E-07	9.80989E-07	27416	Abcc5
01523	Antifolate resistance	13.47736284	3.66319E-07	9.80989E-07	21926	Tnf
01523	Antifolate resistance	13.47736284	3.66319E-07	9.80989E-07	17250	Abcc1
01523	Antifolate resistance	13.47736284	3.66319E-07	9.80989E-07	18033	Nfkb1
01523	Antifolate resistance	13.47736284	3.66319E-07	9.80989E-07	11684	Alox12
01523	Antifolate resistance	13.47736284	3.66319E-07	9.80989E-07	16176	Il1b
04012	ErbB signaling pathway	7.220015807	4.19233E-07	1.11326E-06	13649	Egfr
04012	ErbB signaling pathway	7.220015807	4.19233E-07	1.11326E-06	17973	Nck1
04012	ErbB signaling pathway	7.220015807	4.19233E-07	1.11326E-06	56717	Mtor
04012	ErbB signaling pathway	7.220015807	4.19233E-07	1.11326E-06	12575	Cdkn1a
04012	ErbB signaling pathway	7.220015807	4.19233E-07	1.11326E-06	13874	Ereg
04012	ErbB signaling pathway	7.220015807	4.19233E-07	1.11326E-06	20779	Src
04012	ErbB signaling pathway	7.220015807	4.19233E-07	1.11326E-06	108058	Camk2d
04012	ErbB signaling pathway	7.220015807	4.19233E-07	1.11326E-06	15200	Hbegf
04012	ErbB signaling pathway	7.220015807	4.19233E-07	1.11326E-06	18708	Pik3r1
04012	ErbB signaling pathway	7.220015807	4.19233E-07	1.11326E-06	211323	Nrg1
04012	ErbB signaling pathway	7.220015807	4.19233E-07	1.11326E-06	11839	Areg
04012	ErbB signaling pathway	7.220015807	4.19233E-07	1.11326E-06	17869	Myc
05017	Spinocerebellar ataxia	5.376607516	6.21445E-07	1.63647E-06	56436	Adrm1
05017	Spinocerebellar ataxia	5.376607516	6.21445E-07	1.63647E-06	56717	Mtor
05017	Spinocerebellar ataxia	5.376607516	6.21445E-07	1.63647E-06	13063	Cycs
05017	Spinocerebellar ataxia	5.376607516	6.21445E-07	1.63647E-06	19166	Psma2
05017	Spinocerebellar ataxia	5.376607516	6.21445E-07	1.63647E-06	19181	Psmc2
05017	Spinocerebellar ataxia	5.376607516	6.21445E-07	1.63647E-06	19175	Psmb6
05017	Spinocerebellar ataxia	5.376607516	6.21445E-07	1.63647E-06	19173	Psmb5
05017	Spinocerebellar ataxia	5.376607516	6.21445E-07	1.63647E-06	26443	Psma6
05017	Spinocerebellar ataxia	5.376607516	6.21445E-07	1.63647E-06	26442	Psma5
05017	Spinocerebellar ataxia	5.376607516	6.21445E-07	1.63647E-06	18708	Pik3r1
05017	Spinocerebellar ataxia	5.376607516	6.21445E-07	1.63647E-06	59029	Psmd14
05017	Spinocerebellar ataxia	5.376607516	6.21445E-07	1.63647E-06	22030	Traf2
05017	Spinocerebellar ataxia	5.376607516	6.21445E-07	1.63647E-06	69077	Psmd11
05017	Spinocerebellar ataxia	5.376607516	6.21445E-07	1.63647E-06	18798	Plcb4

05017	Spinocerebellar ataxia	5.376607516	6.21445E-07	1.63647E-06	14814	Grin2d
04613	Neutrophil extracellular trap formation	4.437668252	7.10421E-07	1.85531E-06	18708	Pik3r1
04613	Neutrophil extracellular trap formation	4.437668252	7.10421E-07	1.85531E-06	14294	Fpr3
04613	Neutrophil extracellular trap formation	4.437668252	7.10421E-07	1.85531E-06	16408	Itgal
04613	Neutrophil extracellular trap formation	4.437668252	7.10421E-07	1.85531E-06	24088	Tlr2
04613	Neutrophil extracellular trap formation	4.437668252	7.10421E-07	1.85531E-06	12363	Casp4
04613	Neutrophil extracellular trap formation	4.437668252	7.10421E-07	1.85531E-06	18798	Plcb4
04613	Neutrophil extracellular trap formation	4.437668252	7.10421E-07	1.85531E-06	12362	Casp1
04613	Neutrophil extracellular trap formation	4.437668252	7.10421E-07	1.85531E-06	14293	Fpr1
04613	Neutrophil extracellular trap formation	4.437668252	7.10421E-07	1.85531E-06	18033	Nfkb1
04613	Neutrophil extracellular trap formation	4.437668252	7.10421E-07	1.85531E-06	20779	Src
04613	Neutrophil extracellular trap formation	4.437668252	7.10421E-07	1.85531E-06	12796	Camp
04613	Neutrophil extracellular trap formation	4.437668252	7.10421E-07	1.85531E-06	13058	Cybb
04613	Neutrophil extracellular trap formation	4.437668252	7.10421E-07	1.85531E-06	26415	Mapk13
04613	Neutrophil extracellular trap formation	4.437668252	7.10421E-07	1.85531E-06	14289	Fpr2
04613	Neutrophil extracellular trap formation	4.437668252	7.10421E-07	1.85531E-06	56717	Mtor
04613	Neutrophil extracellular trap formation	4.437668252	7.10421E-07	1.85531E-06	12266	C3
04613	Neutrophil extracellular trap formation	4.437668252	7.10421E-07	1.85531E-06	20963	Syk
04613	Neutrophil extracellular trap formation	4.437668252	7.10421E-07	1.85531E-06	64008	Aqp9
04071	Sphingolipid signaling pathway	5.706141525	7.6317E-07	1.97674E-06	18798	Plcb4
04071	Sphingolipid signaling pathway	5.706141525	7.6317E-07	1.97674E-06	18033	Nfkb1
04071	Sphingolipid signaling pathway	5.706141525	7.6317E-07	1.97674E-06	21926	Tnf
04071	Sphingolipid signaling pathway	5.706141525	7.6317E-07	1.97674E-06	20698	Sphk1
04071	Sphingolipid signaling pathway	5.706141525	7.6317E-07	1.97674E-06	26415	Mapk13
04071	Sphingolipid signaling pathway	5.706141525	7.6317E-07	1.97674E-06	13610	S1pr3
04071	Sphingolipid signaling pathway	5.706141525	7.6317E-07	1.97674E-06	17250	Abcc1
04071	Sphingolipid signaling pathway	5.706141525	7.6317E-07	1.97674E-06	20773	Sptlc2
04071	Sphingolipid signaling pathway	5.706141525	7.6317E-07	1.97674E-06	12122	Bid
04071	Sphingolipid signaling pathway	5.706141525	7.6317E-07	1.97674E-06	12062	Bdkrb2
04071	Sphingolipid signaling pathway	5.706141525	7.6317E-07	1.97674E-06	18708	Pik3r1
04071	Sphingolipid signaling pathway	5.706141525	7.6317E-07	1.97674E-06	22030	Traf2
04071	Sphingolipid signaling pathway	5.706141525	7.6317E-07	1.97674E-06	241447	Cers6
04071	Sphingolipid signaling pathway	5.706141525	7.6317E-07	1.97674E-06	19053	Ppp2cb
00240	Pyrimidine metabolism	8.713812181	7.85995E-07	2.0193E-06	64705	Dpys
00240	Pyrimidine metabolism	8.713812181	7.85995E-07	2.0193E-06	80914	Uck2
00240	Pyrimidine metabolism	8.713812181	7.85995E-07	2.0193E-06	18950	Pnp
00240	Pyrimidine metabolism	8.713812181	7.85995E-07	2.0193E-06	23959	Nt5e
00240	Pyrimidine metabolism	8.713812181	7.85995E-07	2.0193E-06	22271	Upp1
00240	Pyrimidine metabolism	8.713812181	7.85995E-07	2.0193E-06	13178	Dck
00240	Pyrimidine metabolism	8.713812181	7.85995E-07	2.0193E-06	54369	Nme6
00240	Pyrimidine metabolism	8.713812181	7.85995E-07	2.0193E-06	230718	Nt5c1a
00240	Pyrimidine metabolism	8.713812181	7.85995E-07	2.0193E-06	22169	Cmpk2

00240	Pyrimidine metabolism	8.713812181	7.85995E-07	2.0193E-06	107569	Nt5c3
03320	PPAR signaling pathway	6.814396942	8.04385E-07	2.04988E-06	19015	Ppard
03320	PPAR signaling pathway	6.814396942	8.04385E-07	2.04988E-06	14077	Fabp3
03320	PPAR signaling pathway	6.814396942	8.04385E-07	2.04988E-06	14933	Gk
03320	PPAR signaling pathway	6.814396942	8.04385E-07	2.04988E-06	20250	Scd2
03320	PPAR signaling pathway	6.814396942	8.04385E-07	2.04988E-06	14081	Acsl1
03320	PPAR signaling pathway	6.814396942	8.04385E-07	2.04988E-06	108078	Olr1
03320	PPAR signaling pathway	6.814396942	8.04385E-07	2.04988E-06	26569	Slc27a4
03320	PPAR signaling pathway	6.814396942	8.04385E-07	2.04988E-06	83996	Mmp1b
03320	PPAR signaling pathway	6.814396942	8.04385E-07	2.04988E-06	94180	Acsbg1
03320	PPAR signaling pathway	6.814396942	8.04385E-07	2.04988E-06	433256	Acsl5
03320	PPAR signaling pathway	6.814396942	8.04385E-07	2.04988E-06	50790	Acsl4
03320	PPAR signaling pathway	6.814396942	8.04385E-07	2.04988E-06	208715	Hmgcs1
00900	Terpenoid backbone biosynthesis	15.38177281	8.69622E-07	2.1984E-06	110196	Fdps
00900	Terpenoid backbone biosynthesis	15.38177281	8.69622E-07	2.1984E-06	192156	Mvd
00900	Terpenoid backbone biosynthesis	15.38177281	8.69622E-07	2.1984E-06	224530	Acat3
00900	Terpenoid backbone biosynthesis	15.38177281	8.69622E-07	2.1984E-06	110460	Acat2
00900	Terpenoid backbone biosynthesis	15.38177281	8.69622E-07	2.1984E-06	319554	Idi1
00900	Terpenoid backbone biosynthesis	15.38177281	8.69622E-07	2.1984E-06	15357	Hmgcr
00900	Terpenoid backbone biosynthesis	15.38177281	8.69622E-07	2.1984E-06	208715	Hmgcs1
05016	Huntington disease	3.681729915	8.77971E-07	2.20189E-06	56436	Adrm1
05016	Huntington disease	3.681729915	8.77971E-07	2.20189E-06	26442	Psma5
05016	Huntington disease	3.681729915	8.77971E-07	2.20189E-06	59029	Psmd14
05016	Huntington disease	3.681729915	8.77971E-07	2.20189E-06	18798	Plcb4
05016	Huntington disease	3.681729915	8.77971E-07	2.20189E-06	22145	Tuba4a
05016	Huntington disease	3.681729915	8.77971E-07	2.20189E-06	19181	Psmc2
05016	Huntington disease	3.681729915	8.77971E-07	2.20189E-06	13063	Cycs
05016	Huntington disease	3.681729915	8.77971E-07	2.20189E-06	69077	Psmd11
05016	Huntington disease	3.681729915	8.77971E-07	2.20189E-06	26443	Psma6
05016	Huntington disease	3.681729915	8.77971E-07	2.20189E-06	22030	Traf2
05016	Huntington disease	3.681729915	8.77971E-07	2.20189E-06	22153	Tubb4a
05016	Huntington disease	3.681729915	8.77971E-07	2.20189E-06	19173	Psmb5
05016	Huntington disease	3.681729915	8.77971E-07	2.20189E-06	231991	Creb5
05016	Huntington disease	3.681729915	8.77971E-07	2.20189E-06	19166	Psma2
05016	Huntington disease	3.681729915	8.77971E-07	2.20189E-06	21817	Tgm2
05016	Huntington disease	3.681729915	8.77971E-07	2.20189E-06	19175	Psmb6
05016	Huntington disease	3.681729915	8.77971E-07	2.20189E-06	56717	Mtor
05016	Huntington disease	3.681729915	8.77971E-07	2.20189E-06	73916	Ift57
05016	Huntington disease	3.681729915	8.77971E-07	2.20189E-06	20511	Slc1a2
05016	Huntington disease	3.681729915	8.77971E-07	2.20189E-06	22142	Tuba1a
05016	Huntington disease	3.681729915	8.77971E-07	2.20189E-06	69926	Dnah17
05016	Huntington disease	3.681729915	8.77971E-07	2.20189E-06	20656	Sod2

04612	Antigen processing and presentation	6.73868142	9.11462E-07	2.25017E-06	19188	Psme2
04612	Antigen processing and presentation	6.73868142	9.11462E-07	2.25017E-06	15039	H2-T22
04612	Antigen processing and presentation	6.73868142	9.11462E-07	2.25017E-06	15024	H2-T10
04612	Antigen processing and presentation	6.73868142	9.11462E-07	2.25017E-06	15018	H2-Q7
04612	Antigen processing and presentation	6.73868142	9.11462E-07	2.25017E-06	21926	Tnf
04612	Antigen processing and presentation	6.73868142	9.11462E-07	2.25017E-06	15519	Hsp90aa1
04612	Antigen processing and presentation	6.73868142	9.11462E-07	2.25017E-06	14828	Hspa5
04612	Antigen processing and presentation	6.73868142	9.11462E-07	2.25017E-06	14990	H2-M2
04612	Antigen processing and presentation	6.73868142	9.11462E-07	2.25017E-06	110558	H2-Q9
04612	Antigen processing and presentation	6.73868142	9.11462E-07	2.25017E-06	15978	Ifng
04612	Antigen processing and presentation	6.73868142	9.11462E-07	2.25017E-06	15001	H2-Oa
04612	Antigen processing and presentation	6.73868142	9.11462E-07	2.25017E-06	12317	Calr
04912	GnRH signaling pathway	6.73868142	9.11462E-07	2.25017E-06	11911	Atf4
04912	GnRH signaling pathway	6.73868142	9.11462E-07	2.25017E-06	108058	Camk2d
04912	GnRH signaling pathway	6.73868142	9.11462E-07	2.25017E-06	18798	Plcb4
04912	GnRH signaling pathway	6.73868142	9.11462E-07	2.25017E-06	26399	Map2k6
04912	GnRH signaling pathway	6.73868142	9.11462E-07	2.25017E-06	17390	Mmp2
04912	GnRH signaling pathway	6.73868142	9.11462E-07	2.25017E-06	17387	Mmp14
04912	GnRH signaling pathway	6.73868142	9.11462E-07	2.25017E-06	13649	Egfr
04912	GnRH signaling pathway	6.73868142	9.11462E-07	2.25017E-06	20779	Src
04912	GnRH signaling pathway	6.73868142	9.11462E-07	2.25017E-06	18783	Pla2g4a
04912	GnRH signaling pathway	6.73868142	9.11462E-07	2.25017E-06	13653	Egr1
04912	GnRH signaling pathway	6.73868142	9.11462E-07	2.25017E-06	15200	Hbegf
04912	GnRH signaling pathway	6.73868142	9.11462E-07	2.25017E-06	26415	Mapk13
04390	Hippo signaling pathway	4.828672992	2.51973E-06	6.17236E-06	12444	Ccnd2
04390	Hippo signaling pathway	4.828672992	2.51973E-06	6.17236E-06	11796	Birc3
04390	Hippo signaling pathway	4.828672992	2.51973E-06	6.17236E-06	15902	Id2
04390	Hippo signaling pathway	4.828672992	2.51973E-06	6.17236E-06	17869	Myc
04390	Hippo signaling pathway	4.828672992	2.51973E-06	6.17236E-06	12166	Bmpr1a
04390	Hippo signaling pathway	4.828672992	2.51973E-06	6.17236E-06	14164	Fgf1
04390	Hippo signaling pathway	4.828672992	2.51973E-06	6.17236E-06	11839	Areg
04390	Hippo signaling pathway	4.828672992	2.51973E-06	6.17236E-06	12156	Bmp2
04390	Hippo signaling pathway	4.828672992	2.51973E-06	6.17236E-06	64010	Sav1
04390	Hippo signaling pathway	4.828672992	2.51973E-06	6.17236E-06	73246	Rassf6
04390	Hippo signaling pathway	4.828672992	2.51973E-06	6.17236E-06	14362	Fzd1
04390	Hippo signaling pathway	4.828672992	2.51973E-06	6.17236E-06	22420	Wnt6
04390	Hippo signaling pathway	4.828672992	2.51973E-06	6.17236E-06	319710	Frmd6
04390	Hippo signaling pathway	4.828672992	2.51973E-06	6.17236E-06	11576	Afp
04390	Hippo signaling pathway	4.828672992	2.51973E-06	6.17236E-06	19053	Ppp2cb
04664	Fc epsilon RI signaling pathway	7.657592523	2.75433E-06	6.69515E-06	20963	Syk
04664	Fc epsilon RI signaling pathway	7.657592523	2.75433E-06	6.69515E-06	26399	Map2k6
04664	Fc epsilon RI signaling pathway	7.657592523	2.75433E-06	6.69515E-06	17096	Lyn

04664	Fc epsilon RI signaling pathway	7.657592523	2.75433E-06	6.69515E-06	16822	Lcp2
04664	Fc epsilon RI signaling pathway	7.657592523	2.75433E-06	6.69515E-06	18783	Pla2g4a
04664	Fc epsilon RI signaling pathway	7.657592523	2.75433E-06	6.69515E-06	26415	Mapk13
04664	Fc epsilon RI signaling pathway	7.657592523	2.75433E-06	6.69515E-06	18708	Pik3r1
04664	Fc epsilon RI signaling pathway	7.657592523	2.75433E-06	6.69515E-06	12981	Csf2
04664	Fc epsilon RI signaling pathway	7.657592523	2.75433E-06	6.69515E-06	21926	Tnf
04664	Fc epsilon RI signaling pathway	7.657592523	2.75433E-06	6.69515E-06	16797	Lat
00380	Tryptophan metabolism	8.747326843	3.10612E-06	7.47976E-06	224530	Acat3
00380	Tryptophan metabolism	8.747326843	3.10612E-06	7.47976E-06	229905	Kyat3
00380	Tryptophan metabolism	8.747326843	3.10612E-06	7.47976E-06	14204	Il4i1
00380	Tryptophan metabolism	8.747326843	3.10612E-06	7.47976E-06	11761	Aox1
00380	Tryptophan metabolism	8.747326843	3.10612E-06	7.47976E-06	110460	Acat2
00380	Tryptophan metabolism	8.747326843	3.10612E-06	7.47976E-06	73988	4930438A08Rik
00380	Tryptophan metabolism	8.747326843	3.10612E-06	7.47976E-06	209176	Ido2
00380	Tryptophan metabolism	8.747326843	3.10612E-06	7.47976E-06	13078	Cyp1b1
00380	Tryptophan metabolism	8.747326843	3.10612E-06	7.47976E-06	15930	Ido1
04910	Insulin signaling pathway	5.090370857	3.12446E-06	7.47976E-06	15277	Hk2
04910	Insulin signaling pathway	5.090370857	3.12446E-06	7.47976E-06	13684	Eif4e
04910	Insulin signaling pathway	5.090370857	3.12446E-06	7.47976E-06	108079	Prkaa2
04910	Insulin signaling pathway	5.090370857	3.12446E-06	7.47976E-06	12703	Socs1
04910	Insulin signaling pathway	5.090370857	3.12446E-06	7.47976E-06	107746	Rapgef1
04910	Insulin signaling pathway	5.090370857	3.12446E-06	7.47976E-06	18708	Pik3r1
04910	Insulin signaling pathway	5.090370857	3.12446E-06	7.47976E-06	14936	Gys1
04910	Insulin signaling pathway	5.090370857	3.12446E-06	7.47976E-06	12702	Socs3
04910	Insulin signaling pathway	5.090370857	3.12446E-06	7.47976E-06	104215	Rhoq
04910	Insulin signaling pathway	5.090370857	3.12446E-06	7.47976E-06	105787	Prkaa1
04910	Insulin signaling pathway	5.090370857	3.12446E-06	7.47976E-06	19088	Prkar2b
04910	Insulin signaling pathway	5.090370857	3.12446E-06	7.47976E-06	56717	Mtor
04910	Insulin signaling pathway	5.090370857	3.12446E-06	7.47976E-06	19246	Ptpn1
04910	Insulin signaling pathway	5.090370857	3.12446E-06	7.47976E-06	216233	Socs2
04270	Vascular smooth muscle contraction	4.947982861	4.40595E-06	1.04683E-05	11540	Adora2a
04270	Vascular smooth muscle contraction	4.947982861	4.40595E-06	1.04683E-05	54598	Calcrl
04270	Vascular smooth muscle contraction	4.947982861	4.40595E-06	1.04683E-05	16531	Kcnma1
04270	Vascular smooth muscle contraction	4.947982861	4.40595E-06	1.04683E-05	17880	Myh11
04270	Vascular smooth muscle contraction	4.947982861	4.40595E-06	1.04683E-05	329251	Ppp1r12b
04270	Vascular smooth muscle contraction	4.947982861	4.40595E-06	1.04683E-05	109624	Cald1
04270	Vascular smooth muscle contraction	4.947982861	4.40595E-06	1.04683E-05	18783	Pla2g4a
04270	Vascular smooth muscle contraction	4.947982861	4.40595E-06	1.04683E-05	18798	Plcb4
04270	Vascular smooth muscle contraction	4.947982861	4.40595E-06	1.04683E-05	11535	Adm
04270	Vascular smooth muscle contraction	4.947982861	4.40595E-06	1.04683E-05	18784	Pla2g5
04270	Vascular smooth muscle contraction	4.947982861	4.40595E-06	1.04683E-05	228785	Mylk2
04270	Vascular smooth muscle contraction	4.947982861	4.40595E-06	1.04683E-05	11541	Adora2b

04270	Vascular smooth muscle contraction	4.947982861	4.40595E-06	1.04683E-05	13614	Edn1
04270	Vascular smooth muscle contraction	4.947982861	4.40595E-06	1.04683E-05	11475	Acta2
04922	Glucagon signaling pathway	5.831551229	4.46395E-06	1.05269E-05	108079	Prkaa2
04922	Glucagon signaling pathway	5.831551229	4.46395E-06	1.05269E-05	11911	Atf4
04922	Glucagon signaling pathway	5.831551229	4.46395E-06	1.05269E-05	105787	Prkaa1
04922	Glucagon signaling pathway	5.831551229	4.46395E-06	1.05269E-05	18648	Pgam1
04922	Glucagon signaling pathway	5.831551229	4.46395E-06	1.05269E-05	19057	Ppp3cc
04922	Glucagon signaling pathway	5.831551229	4.46395E-06	1.05269E-05	231991	Creb5
04922	Glucagon signaling pathway	5.831551229	4.46395E-06	1.05269E-05	14936	Gys1
04922	Glucagon signaling pathway	5.831551229	4.46395E-06	1.05269E-05	108058	Camk2d
04922	Glucagon signaling pathway	5.831551229	4.46395E-06	1.05269E-05	16828	Ldha
04922	Glucagon signaling pathway	5.831551229	4.46395E-06	1.05269E-05	20525	Slc2a1
04922	Glucagon signaling pathway	5.831551229	4.46395E-06	1.05269E-05	18641	Pfkl
04922	Glucagon signaling pathway	5.831551229	4.46395E-06	1.05269E-05	18798	Plcb4
05150	Staphylococcus aureus infection	5.298559988	4.8705E-06	1.14006E-05	15894	Icam1
05150	Staphylococcus aureus infection	5.298559988	4.8705E-06	1.14006E-05	12796	Camp
05150	Staphylococcus aureus infection	5.298559988	4.8705E-06	1.14006E-05	16153	Il10
05150	Staphylococcus aureus infection	5.298559988	4.8705E-06	1.14006E-05	12266	C3
05150	Staphylococcus aureus infection	5.298559988	4.8705E-06	1.14006E-05	14294	Fpr3
05150	Staphylococcus aureus infection	5.298559988	4.8705E-06	1.14006E-05	16408	Itgal
05150	Staphylococcus aureus infection	5.298559988	4.8705E-06	1.14006E-05	667277	C1rb
05150	Staphylococcus aureus infection	5.298559988	4.8705E-06	1.14006E-05	15001	H2-Oa
05150	Staphylococcus aureus infection	5.298559988	4.8705E-06	1.14006E-05	14962	Cfb
05150	Staphylococcus aureus infection	5.298559988	4.8705E-06	1.14006E-05	14293	Fpr1
05150	Staphylococcus aureus infection	5.298559988	4.8705E-06	1.14006E-05	14130	Fcgr2b
05150	Staphylococcus aureus infection	5.298559988	4.8705E-06	1.14006E-05	14289	Fpr2
05150	Staphylococcus aureus infection	5.298559988	4.8705E-06	1.14006E-05	50908	C1s1
00760	Nicotinate and nicotinamide metabolism	9.861485005	4.93068E-06	1.14566E-05	12182	Bst1
00760	Nicotinate and nicotinamide metabolism	9.861485005	4.93068E-06	1.14566E-05	12494	Cd38
00760	Nicotinate and nicotinamide metabolism	9.861485005	4.93068E-06	1.14566E-05	192185	Nadk
00760	Nicotinate and nicotinamide metabolism	9.861485005	4.93068E-06	1.14566E-05	23959	Nt5e
00760	Nicotinate and nicotinamide metabolism	9.861485005	4.93068E-06	1.14566E-05	59027	Nampt
00760	Nicotinate and nicotinamide metabolism	9.861485005	4.93068E-06	1.14566E-05	18950	Pnp
00760	Nicotinate and nicotinamide metabolism	9.861485005	4.93068E-06	1.14566E-05	230718	Nt5c1a
00760	Nicotinate and nicotinamide metabolism	9.861485005	4.93068E-06	1.14566E-05	11761	Aox1
05210	Colorectal cancer	6.317513831	5.57774E-06	1.28654E-05	17869	Myc
05210	Colorectal cancer	6.317513831	5.57774E-06	1.28654E-05	13197	Gadd45a
05210	Colorectal cancer	6.317513831	5.57774E-06	1.28654E-05	56717	Mtor
05210	Colorectal cancer	6.317513831	5.57774E-06	1.28654E-05	12575	Cdkn1a
05210	Colorectal cancer	6.317513831	5.57774E-06	1.28654E-05	13874	Ereg
05210	Colorectal cancer	6.317513831	5.57774E-06	1.28654E-05	13063	Cycs
05210	Colorectal cancer	6.317513831	5.57774E-06	1.28654E-05	11839	Areg

05210	Colorectal cancer	6.317513831	5.57774E-06	1.28654E-05	17873	Gadd45b
05210	Colorectal cancer	6.317513831	5.57774E-06	1.28654E-05	13649	Egfr
05210	Colorectal cancer	6.317513831	5.57774E-06	1.28654E-05	18708	Pik3r1
05210	Colorectal cancer	6.317513831	5.57774E-06	1.28654E-05	19730	Ralgds
04152	AMPK signaling pathway	5.214455861	5.84474E-06	1.33836E-05	108079	Prkaa2
04152	AMPK signaling pathway	5.214455861	5.84474E-06	1.33836E-05	56717	Mtor
04152	AMPK signaling pathway	5.214455861	5.84474E-06	1.33836E-05	170768	Pfkfb3
04152	AMPK signaling pathway	5.214455861	5.84474E-06	1.33836E-05	14936	Gys1
04152	AMPK signaling pathway	5.214455861	5.84474E-06	1.33836E-05	15357	Hmgcr
04152	AMPK signaling pathway	5.214455861	5.84474E-06	1.33836E-05	19325	Rab10
04152	AMPK signaling pathway	5.214455861	5.84474E-06	1.33836E-05	20250	Scd2
04152	AMPK signaling pathway	5.214455861	5.84474E-06	1.33836E-05	18708	Pik3r1
04152	AMPK signaling pathway	5.214455861	5.84474E-06	1.33836E-05	231991	Creb5
04152	AMPK signaling pathway	5.214455861	5.84474E-06	1.33836E-05	16847	Lepr
04152	AMPK signaling pathway	5.214455861	5.84474E-06	1.33836E-05	105787	Prkaa1
04152	AMPK signaling pathway	5.214455861	5.84474E-06	1.33836E-05	19053	Ppp2cb
04152	AMPK signaling pathway	5.214455861	5.84474E-06	1.33836E-05	18641	Pfkl
04750	Inflammatory mediator regulation of TRP channels	5.173397153	6.39395E-06	1.45359E-05	20779	Src
04750	Inflammatory mediator regulation of TRP channels	5.173397153	6.39395E-06	1.45359E-05	12062	Bdkrb2
04750	Inflammatory mediator regulation of TRP channels	5.173397153	6.39395E-06	1.45359E-05	18049	Ngf
04750	Inflammatory mediator regulation of TRP channels	5.173397153	6.39395E-06	1.45359E-05	16177	Il1r1
04750	Inflammatory mediator regulation of TRP channels	5.173397153	6.39395E-06	1.45359E-05	16176	Il1b
04750	Inflammatory mediator regulation of TRP channels	5.173397153	6.39395E-06	1.45359E-05	18798	Plcb4
04750	Inflammatory mediator regulation of TRP channels	5.173397153	6.39395E-06	1.45359E-05	18442	P2ry2
04750	Inflammatory mediator regulation of TRP channels	5.173397153	6.39395E-06	1.45359E-05	26399	Map2k6
04750	Inflammatory mediator regulation of TRP channels	5.173397153	6.39395E-06	1.45359E-05	18708	Pik3r1
04750	Inflammatory mediator regulation of TRP channels	5.173397153	6.39395E-06	1.45359E-05	26415	Mapk13
04750	Inflammatory mediator regulation of TRP channels	5.173397153	6.39395E-06	1.45359E-05	18783	Pla2g4a
04750	Inflammatory mediator regulation of TRP channels	5.173397153	6.39395E-06	1.45359E-05	108058	Camk2d
04750	Inflammatory mediator regulation of TRP channels	5.173397153	6.39395E-06	1.45359E-05	11684	Alox12
00220	Arginine biosynthesis	15.1620332	7.25886E-06	1.62681E-05	11847	Arg2
00220	Arginine biosynthesis	15.1620332	7.25886E-06	1.62681E-05	11898	Ass1
00220	Arginine biosynthesis	15.1620332	7.25886E-06	1.62681E-05	217214	Nags
00220	Arginine biosynthesis	15.1620332	7.25886E-06	1.62681E-05	109652	Acy1
00220	Arginine biosynthesis	15.1620332	7.25886E-06	1.62681E-05	11846	Arg1
00220	Arginine biosynthesis	15.1620332	7.25886E-06	1.62681E-05	18126	Nos2
00532	Glycosaminoglycan biosynthesis - chondroitin sulfate / dermatan sulfate	15.1620332	7.25886E-06	1.62681E-05	338362	Ust
00532	Glycosaminoglycan biosynthesis - chondroitin sulfate / dermatan sulfate	15.1620332	7.25886E-06	1.62681E-05	60322	Chst7
00532	Glycosaminoglycan biosynthesis - chondroitin sulfate / dermatan sulfate	15.1620332	7.25886E-06	1.62681E-05	217119	Xylt2
00532	Glycosaminoglycan biosynthesis - chondroitin sulfate / dermatan sulfate	15.1620332	7.25886E-06	1.62681E-05	78923	Chsy3
00532	Glycosaminoglycan biosynthesis - chondroitin sulfate / dermatan sulfate	15.1620332	7.25886E-06	1.62681E-05	74241	Chpf
00532	Glycosaminoglycan biosynthesis - chondroitin sulfate / dermatan sulfate	15.1620332	7.25886E-06	1.62681E-05	58250	Chst11

04370	VEGF signaling pathway	7.842430963	8.08422E-06	1.79902E-05	18708	Pik3r1
04370	VEGF signaling pathway	7.842430963	8.08422E-06	1.79902E-05	18783	Pla2g4a
04370	VEGF signaling pathway	7.842430963	8.08422E-06	1.79902E-05	20779	Src
04370	VEGF signaling pathway	7.842430963	8.08422E-06	1.79902E-05	22339	Vegfa
04370	VEGF signaling pathway	7.842430963	8.08422E-06	1.79902E-05	26415	Mapk13
04370	VEGF signaling pathway	7.842430963	8.08422E-06	1.79902E-05	17164	Mapkapk2
04370	VEGF signaling pathway	7.842430963	8.08422E-06	1.79902E-05	19225	Ptgs2
04370	VEGF signaling pathway	7.842430963	8.08422E-06	1.79902E-05	20698	Sphk1
04370	VEGF signaling pathway	7.842430963	8.08422E-06	1.79902E-05	19057	Ppp3cc
04740	Olfactory transduction	0.173230885	8.24245E-06	1.82141E-05	18356	Olfr56
04740	Olfactory transduction	0.173230885	8.24245E-06	1.82141E-05	258504	Olfr107
04740	Olfactory transduction	0.173230885	8.24245E-06	1.82141E-05	258571	Olfr1033
04740	Olfactory transduction	0.173230885	8.24245E-06	1.82141E-05	108058	Camk2d
01522	Endocrine resistance	5.97786255	9.71069E-06	2.13096E-05	13649	Egfr
01522	Endocrine resistance	5.97786255	9.71069E-06	2.13096E-05	17390	Mmp2
01522	Endocrine resistance	5.97786255	9.71069E-06	2.13096E-05	56717	Mtor
01522	Endocrine resistance	5.97786255	9.71069E-06	2.13096E-05	17395	Mmp9
01522	Endocrine resistance	5.97786255	9.71069E-06	2.13096E-05	15200	Hbegf
01522	Endocrine resistance	5.97786255	9.71069E-06	2.13096E-05	18708	Pik3r1
01522	Endocrine resistance	5.97786255	9.71069E-06	2.13096E-05	26415	Mapk13
01522	Endocrine resistance	5.97786255	9.71069E-06	2.13096E-05	12575	Cdkn1a
01522	Endocrine resistance	5.97786255	9.71069E-06	2.13096E-05	20779	Src
01522	Endocrine resistance	5.97786255	9.71069E-06	2.13096E-05	16449	Jag1
01522	Endocrine resistance	5.97786255	9.71069E-06	2.13096E-05	18128	Notch1
05212	Pancreatic cancer	6.650014559	1.04231E-05	2.27151E-05	19730	Ralgds
05212	Pancreatic cancer	6.650014559	1.04231E-05	2.27151E-05	13649	Egfr
05212	Pancreatic cancer	6.650014559	1.04231E-05	2.27151E-05	22339	Vegfa
05212	Pancreatic cancer	6.650014559	1.04231E-05	2.27151E-05	18033	Nfkb1
05212	Pancreatic cancer	6.650014559	1.04231E-05	2.27151E-05	56717	Mtor
05212	Pancreatic cancer	6.650014559	1.04231E-05	2.27151E-05	12575	Cdkn1a
05212	Pancreatic cancer	6.650014559	1.04231E-05	2.27151E-05	20846	Stat1
05212	Pancreatic cancer	6.650014559	1.04231E-05	2.27151E-05	13197	Gadd45a
05212	Pancreatic cancer	6.650014559	1.04231E-05	2.27151E-05	17873	Gadd45b
05212	Pancreatic cancer	6.650014559	1.04231E-05	2.27151E-05	18708	Pik3r1
05414	Dilated cardiomyopathy	5.914268268	1.08018E-05	2.33792E-05	81905	Cacng8
05414	Dilated cardiomyopathy	5.914268268	1.08018E-05	2.33792E-05	54378	Cacng6
05414	Dilated cardiomyopathy	5.914268268	1.08018E-05	2.33792E-05	319480	Itga11
05414	Dilated cardiomyopathy	5.914268268	1.08018E-05	2.33792E-05	109700	Itga1
05414	Dilated cardiomyopathy	5.914268268	1.08018E-05	2.33792E-05	17868	Mybpc3
05414	Dilated cardiomyopathy	5.914268268	1.08018E-05	2.33792E-05	21926	Tnf
05414	Dilated cardiomyopathy	5.914268268	1.08018E-05	2.33792E-05	326618	Tpm4
05414	Dilated cardiomyopathy	5.914268268	1.08018E-05	2.33792E-05	104099	Itga9

05414	Dilated cardiomyopathy	5.914268268	1.08018E-05	2.33792E-05	320910	Itgb8
05414	Dilated cardiomyopathy	5.914268268	1.08018E-05	2.33792E-05	22138	Ttn
05414	Dilated cardiomyopathy	5.914268268	1.08018E-05	2.33792E-05	16402	Itga5
01521	EGFR tyrosine kinase inhibitor resistance	6.397482361	1.49118E-05	3.17748E-05	17295	Met
01521	EGFR tyrosine kinase inhibitor resistance	6.397482361	1.49118E-05	3.17748E-05	16193	Il6
01521	EGFR tyrosine kinase inhibitor resistance	6.397482361	1.49118E-05	3.17748E-05	13649	Egfr
01521	EGFR tyrosine kinase inhibitor resistance	6.397482361	1.49118E-05	3.17748E-05	13684	Eif4e
01521	EGFR tyrosine kinase inhibitor resistance	6.397482361	1.49118E-05	3.17748E-05	56717	Mtor
01521	EGFR tyrosine kinase inhibitor resistance	6.397482361	1.49118E-05	3.17748E-05	22339	Vegfa
01521	EGFR tyrosine kinase inhibitor resistance	6.397482361	1.49118E-05	3.17748E-05	211323	Nrg1
01521	EGFR tyrosine kinase inhibitor resistance	6.397482361	1.49118E-05	3.17748E-05	20779	Src
01521	EGFR tyrosine kinase inhibitor resistance	6.397482361	1.49118E-05	3.17748E-05	18708	Pik3r1
01521	EGFR tyrosine kinase inhibitor resistance	6.397482361	1.49118E-05	3.17748E-05	16452	Jak2
05320	Autoimmune thyroid disease	6.397482361	1.49118E-05	3.17748E-05	15001	H2-Oa
05320	Autoimmune thyroid disease	6.397482361	1.49118E-05	3.17748E-05	21939	Cd40
05320	Autoimmune thyroid disease	6.397482361	1.49118E-05	3.17748E-05	110558	H2-Q9
05320	Autoimmune thyroid disease	6.397482361	1.49118E-05	3.17748E-05	14102	Fas
05320	Autoimmune thyroid disease	6.397482361	1.49118E-05	3.17748E-05	12524	Cd86
05320	Autoimmune thyroid disease	6.397482361	1.49118E-05	3.17748E-05	15024	H2-T10
05320	Autoimmune thyroid disease	6.397482361	1.49118E-05	3.17748E-05	15039	H2-T22
05320	Autoimmune thyroid disease	6.397482361	1.49118E-05	3.17748E-05	14990	H2-M2
05320	Autoimmune thyroid disease	6.397482361	1.49118E-05	3.17748E-05	16153	Il10
05320	Autoimmune thyroid disease	6.397482361	1.49118E-05	3.17748E-05	15018	H2-Q7
04371	Apelin signaling pathway	4.795776923	1.49824E-05	3.17748E-05	105787	Prkaa1
04371	Apelin signaling pathway	4.795776923	1.49824E-05	3.17748E-05	11475	Acta2
04371	Apelin signaling pathway	4.795776923	1.49824E-05	3.17748E-05	320207	Pik3r5
04371	Apelin signaling pathway	4.795776923	1.49824E-05	3.17748E-05	228785	Mylk2
04371	Apelin signaling pathway	4.795776923	1.49824E-05	3.17748E-05	108079	Prkaa2
04371	Apelin signaling pathway	4.795776923	1.49824E-05	3.17748E-05	18126	Nos2
04371	Apelin signaling pathway	4.795776923	1.49824E-05	3.17748E-05	13653	Egr1
04371	Apelin signaling pathway	4.795776923	1.49824E-05	3.17748E-05	16449	Jag1
04371	Apelin signaling pathway	4.795776923	1.49824E-05	3.17748E-05	14701	Gng12
04371	Apelin signaling pathway	4.795776923	1.49824E-05	3.17748E-05	18798	Plcb4
04371	Apelin signaling pathway	4.795776923	1.49824E-05	3.17748E-05	56717	Mtor
04371	Apelin signaling pathway	4.795776923	1.49824E-05	3.17748E-05	30955	Pik3cg
04371	Apelin signaling pathway	4.795776923	1.49824E-05	3.17748E-05	20698	Sphk1
04140	Autophagy - animal	4.761024916	1.6243E-05	3.41408E-05	73205	C9orf72
04140	Autophagy - animal	4.761024916	1.6243E-05	3.41408E-05	18708	Pik3r1
04140	Autophagy - animal	4.761024916	1.6243E-05	3.41408E-05	15251	Hif1a
04140	Autophagy - animal	4.761024916	1.6243E-05	3.41408E-05	19053	Ppp2cb
04140	Autophagy - animal	4.761024916	1.6243E-05	3.41408E-05	108079	Prkaa2
04140	Autophagy - animal	4.761024916	1.6243E-05	3.41408E-05	105787	Prkaa1

04140	Autophagy - animal	4.761024916	1.6243E-05	3.41408E-05	12633	Cflar
04140	Autophagy - animal	4.761024916	1.6243E-05	3.41408E-05	56717	Mtor
04140	Autophagy - animal	4.761024916	1.6243E-05	3.41408E-05	18412	Sqstm1
04140	Autophagy - animal	4.761024916	1.6243E-05	3.41408E-05	56480	Tbk1
04140	Autophagy - animal	4.761024916	1.6243E-05	3.41408E-05	21353	Tank
04140	Autophagy - animal	4.761024916	1.6243E-05	3.41408E-05	12176	Bnip3
04140	Autophagy - animal	4.761024916	1.6243E-05	3.41408E-05	74747	Ddit4
05231	Choline metabolism in cancer	5.672869563	1.63141E-05	3.41408E-05	67916	Plpp3
05231	Choline metabolism in cancer	5.672869563	1.63141E-05	3.41408E-05	18720	Pip5k1a
05231	Choline metabolism in cancer	5.672869563	1.63141E-05	3.41408E-05	15251	Hif1a
05231	Choline metabolism in cancer	5.672869563	1.63141E-05	3.41408E-05	56717	Mtor
05231	Choline metabolism in cancer	5.672869563	1.63141E-05	3.41408E-05	18783	Pla2g4a
05231	Choline metabolism in cancer	5.672869563	1.63141E-05	3.41408E-05	18719	Pip5k1b
05231	Choline metabolism in cancer	5.672869563	1.63141E-05	3.41408E-05	19012	Plpp1
05231	Choline metabolism in cancer	5.672869563	1.63141E-05	3.41408E-05	18708	Pik3r1
05231	Choline metabolism in cancer	5.672869563	1.63141E-05	3.41408E-05	380921	Dgkh
05231	Choline metabolism in cancer	5.672869563	1.63141E-05	3.41408E-05	19730	Ralgds
05231	Choline metabolism in cancer	5.672869563	1.63141E-05	3.41408E-05	13649	Egfr
05204	Chemical carcinogenesis	5.504368487	2.19286E-05	4.55883E-05	100042295	Gm3776
05204	Chemical carcinogenesis	5.504368487	2.19286E-05	4.55883E-05	11529	Adh7
05204	Chemical carcinogenesis	5.504368487	2.19286E-05	4.55883E-05	11670	Aldh3a1
05204	Chemical carcinogenesis	5.504368487	2.19286E-05	4.55883E-05	19225	Ptgs2
05204	Chemical carcinogenesis	5.504368487	2.19286E-05	4.55883E-05	14858	Gsta2
05204	Chemical carcinogenesis	5.504368487	2.19286E-05	4.55883E-05	14860	Gsta4
05204	Chemical carcinogenesis	5.504368487	2.19286E-05	4.55883E-05	13078	Cyp1b1
05204	Chemical carcinogenesis	5.504368487	2.19286E-05	4.55883E-05	15483	Hsd11b1
05204	Chemical carcinogenesis	5.504368487	2.19286E-05	4.55883E-05	14857	Gsta1
05204	Chemical carcinogenesis	5.504368487	2.19286E-05	4.55883E-05	229905	Kyat3
05204	Chemical carcinogenesis	5.504368487	2.19286E-05	4.55883E-05	14863	Gstm2
05224	Breast cancer	4.469533595	3.25394E-05	6.72056E-05	14164	Fgf1
05224	Breast cancer	4.469533595	3.25394E-05	6.72056E-05	22420	Wnt6
05224	Breast cancer	4.469533595	3.25394E-05	6.72056E-05	13649	Egfr
05224	Breast cancer	4.469533595	3.25394E-05	6.72056E-05	18034	Nfkb2
05224	Breast cancer	4.469533595	3.25394E-05	6.72056E-05	17873	Gadd45b
05224	Breast cancer	4.469533595	3.25394E-05	6.72056E-05	14362	Fzd1
05224	Breast cancer	4.469533595	3.25394E-05	6.72056E-05	18708	Pik3r1
05224	Breast cancer	4.469533595	3.25394E-05	6.72056E-05	12575	Cdkn1a
05224	Breast cancer	4.469533595	3.25394E-05	6.72056E-05	17869	Myc
05224	Breast cancer	4.469533595	3.25394E-05	6.72056E-05	56717	Mtor
05224	Breast cancer	4.469533595	3.25394E-05	6.72056E-05	13197	Gadd45a
05224	Breast cancer	4.469533595	3.25394E-05	6.72056E-05	16449	Jag1
05224	Breast cancer	4.469533595	3.25394E-05	6.72056E-05	18128	Notch1

04978	Mineral absorption	7.628695947	3.70769E-05	7.60799E-05	15368	Hmox1
04978	Mineral absorption	7.628695947	3.70769E-05	7.60799E-05	17750	Mt2
04978	Mineral absorption	7.628695947	3.70769E-05	7.60799E-05	18174	Slc11a2
04978	Mineral absorption	7.628695947	3.70769E-05	7.60799E-05	22337	Vdr
04978	Mineral absorption	7.628695947	3.70769E-05	7.60799E-05	381290	Atp2b4
04978	Mineral absorption	7.628695947	3.70769E-05	7.60799E-05	20529	Slc31a1
04978	Mineral absorption	7.628695947	3.70769E-05	7.60799E-05	72027	Slc39a4
04978	Mineral absorption	7.628695947	3.70769E-05	7.60799E-05	17748	Mt1
05226	Gastric cancer	4.380142923	4.05258E-05	8.26203E-05	18671	Abcb1a
05226	Gastric cancer	4.380142923	4.05258E-05	8.26203E-05	18708	Pik3r1
05226	Gastric cancer	4.380142923	4.05258E-05	8.26203E-05	12579	Cdkn2b
05226	Gastric cancer	4.380142923	4.05258E-05	8.26203E-05	17873	Gadd45b
05226	Gastric cancer	4.380142923	4.05258E-05	8.26203E-05	14164	Fgf1
05226	Gastric cancer	4.380142923	4.05258E-05	8.26203E-05	17869	Myc
05226	Gastric cancer	4.380142923	4.05258E-05	8.26203E-05	14362	Fzd1
05226	Gastric cancer	4.380142923	4.05258E-05	8.26203E-05	56717	Mtor
05226	Gastric cancer	4.380142923	4.05258E-05	8.26203E-05	13197	Gadd45a
05226	Gastric cancer	4.380142923	4.05258E-05	8.26203E-05	17295	Met
05226	Gastric cancer	4.380142923	4.05258E-05	8.26203E-05	22420	Wnt6
05226	Gastric cancer	4.380142923	4.05258E-05	8.26203E-05	12575	Cdkn1a
05226	Gastric cancer	4.380142923	4.05258E-05	8.26203E-05	13649	Egfr
00330	Arginine and proline metabolism	7.4874238	4.27714E-05	8.66395E-05	11847	Arg2
00330	Arginine and proline metabolism	7.4874238	4.27714E-05	8.66395E-05	18126	Nos2
00330	Arginine and proline metabolism	7.4874238	4.27714E-05	8.66395E-05	20810	Srm
00330	Arginine and proline metabolism	7.4874238	4.27714E-05	8.66395E-05	228608	Smox
00330	Arginine and proline metabolism	7.4874238	4.27714E-05	8.66395E-05	18452	P4ha2
00330	Arginine and proline metabolism	7.4874238	4.27714E-05	8.66395E-05	18451	P4ha1
00330	Arginine and proline metabolism	7.4874238	4.27714E-05	8.66395E-05	18263	Odc1
00330	Arginine and proline metabolism	7.4874238	4.27714E-05	8.66395E-05	11846	Arg1
00982	Drug metabolism - cytochrome P450	6.406492899	4.49311E-05	9.04345E-05	14261	Fmo1
00982	Drug metabolism - cytochrome P450	6.406492899	4.49311E-05	9.04345E-05	14858	Gsta2
00982	Drug metabolism - cytochrome P450	6.406492899	4.49311E-05	9.04345E-05	14860	Gsta4
00982	Drug metabolism - cytochrome P450	6.406492899	4.49311E-05	9.04345E-05	100042295	Gm3776
00982	Drug metabolism - cytochrome P450	6.406492899	4.49311E-05	9.04345E-05	11670	Aldh3a1
00982	Drug metabolism - cytochrome P450	6.406492899	4.49311E-05	9.04345E-05	14857	Gsta1
00982	Drug metabolism - cytochrome P450	6.406492899	4.49311E-05	9.04345E-05	14863	Gstm2
00982	Drug metabolism - cytochrome P450	6.406492899	4.49311E-05	9.04345E-05	11761	Aox1
00982	Drug metabolism - cytochrome P450	6.406492899	4.49311E-05	9.04345E-05	11529	Adh7
04261	Adrenergic signaling in cardiomyocytes	4.322509463	4.67641E-05	9.35282E-05	19053	Ppp2cb
04261	Adrenergic signaling in cardiomyocytes	4.322509463	4.67641E-05	9.35282E-05	231991	Creb5
04261	Adrenergic signaling in cardiomyocytes	4.322509463	4.67641E-05	9.35282E-05	11911	Atf4
04261	Adrenergic signaling in cardiomyocytes	4.322509463	4.67641E-05	9.35282E-05	30955	Pik3cg

04261	Adrenergic signaling in cardiomyocytes	4.322509463	4.67641E-05	9.35282E-05	381290	Atp2b4
04261	Adrenergic signaling in cardiomyocytes	4.322509463	4.67641E-05	9.35282E-05	320207	Pik3r5
04261	Adrenergic signaling in cardiomyocytes	4.322509463	4.67641E-05	9.35282E-05	54378	Cacng6
04261	Adrenergic signaling in cardiomyocytes	4.322509463	4.67641E-05	9.35282E-05	26415	Mapk13
04261	Adrenergic signaling in cardiomyocytes	4.322509463	4.67641E-05	9.35282E-05	108058	Camk2d
04261	Adrenergic signaling in cardiomyocytes	4.322509463	4.67641E-05	9.35282E-05	18798	Plcb4
04261	Adrenergic signaling in cardiomyocytes	4.322509463	4.67641E-05	9.35282E-05	326618	Tpm4
04261	Adrenergic signaling in cardiomyocytes	4.322509463	4.67641E-05	9.35282E-05	81905	Cacng8
04261	Adrenergic signaling in cardiomyocytes	4.322509463	4.67641E-05	9.35282E-05	12916	Crem
00260	Glycine, serine and threonine metabolism	8.844519364	4.93117E-05	9.80032E-05	71776	Tha1
00260	Glycine, serine and threonine metabolism	8.844519364	4.93117E-05	9.80032E-05	11655	Alas1
00260	Glycine, serine and threonine metabolism	8.844519364	4.93117E-05	9.80032E-05	236539	Phgdh
00260	Glycine, serine and threonine metabolism	8.844519364	4.93117E-05	9.80032E-05	107272	Psat1
00260	Glycine, serine and threonine metabolism	8.844519364	4.93117E-05	9.80032E-05	76238	Grhpr
00260	Glycine, serine and threonine metabolism	8.844519364	4.93117E-05	9.80032E-05	18648	Pgam1
00260	Glycine, serine and threonine metabolism	8.844519364	4.93117E-05	9.80032E-05	107869	Cth
00980	Metabolism of xenobiotics by cytochrome P450	6.230972546	5.65396E-05	0.000111666	14863	Gstm2
00980	Metabolism of xenobiotics by cytochrome P450	6.230972546	5.65396E-05	0.000111666	14857	Gsta1
00980	Metabolism of xenobiotics by cytochrome P450	6.230972546	5.65396E-05	0.000111666	13078	Cyp1b1
00980	Metabolism of xenobiotics by cytochrome P450	6.230972546	5.65396E-05	0.000111666	14860	Gsta4
00980	Metabolism of xenobiotics by cytochrome P450	6.230972546	5.65396E-05	0.000111666	14858	Gsta2
00980	Metabolism of xenobiotics by cytochrome P450	6.230972546	5.65396E-05	0.000111666	15483	Hsd11b1
00980	Metabolism of xenobiotics by cytochrome P450	6.230972546	5.65396E-05	0.000111666	100042295	Gm3776
00980	Metabolism of xenobiotics by cytochrome P450	6.230972546	5.65396E-05	0.000111666	11529	Adh7
00980	Metabolism of xenobiotics by cytochrome P450	6.230972546	5.65396E-05	0.000111666	11670	Aldh3a1
04150	mTOR signaling pathway	4.211675888	6.18202E-05	0.000121337	74747	Ddit4
04150	mTOR signaling pathway	4.211675888	6.18202E-05	0.000121337	108079	Prkaa2
04150	mTOR signaling pathway	4.211675888	6.18202E-05	0.000121337	105787	Prkaa1
04150	mTOR signaling pathway	4.211675888	6.18202E-05	0.000121337	56717	Mtor
04150	mTOR signaling pathway	4.211675888	6.18202E-05	0.000121337	11973	Atp6v1e1
04150	mTOR signaling pathway	4.211675888	6.18202E-05	0.000121337	14362	Fzd1
04150	mTOR signaling pathway	4.211675888	6.18202E-05	0.000121337	20539	Slc7a5
04150	mTOR signaling pathway	4.211675888	6.18202E-05	0.000121337	17254	Slc3a2
04150	mTOR signaling pathway	4.211675888	6.18202E-05	0.000121337	22420	Wnt6
04150	mTOR signaling pathway	4.211675888	6.18202E-05	0.000121337	13684	Eif4e
04150	mTOR signaling pathway	4.211675888	6.18202E-05	0.000121337	21926	Tnf
04150	mTOR signaling pathway	4.211675888	6.18202E-05	0.000121337	20112	Rps6ka2
04150	mTOR signaling pathway	4.211675888	6.18202E-05	0.000121337	18708	Pik3r1
00280	Valine, leucine and isoleucine degradation	7.093348863	6.44611E-05	0.000125739	110460	Acat2
00280	Valine, leucine and isoleucine degradation	7.093348863	6.44611E-05	0.000125739	14204	Il4i1
00280	Valine, leucine and isoleucine degradation	7.093348863	6.44611E-05	0.000125739	12040	Bckdhb
00280	Valine, leucine and isoleucine degradation	7.093348863	6.44611E-05	0.000125739	11761	Aox1

00280	Valine, leucine and isoleucine degradation	7.093348863	6.44611E-05	0.000125739	12035	Bcat1
00280	Valine, leucine and isoleucine degradation	7.093348863	6.44611E-05	0.000125739	224530	Acat3
00280	Valine, leucine and isoleucine degradation	7.093348863	6.44611E-05	0.000125739	208715	Hmgcs1
00280	Valine, leucine and isoleucine degradation	7.093348863	6.44611E-05	0.000125739	73988	4930438A08Rik
04672	Intestinal immune network for IgA production	8.227459873	8.09422E-05	0.000155962	16153	Il10
04672	Intestinal immune network for IgA production	8.227459873	8.09422E-05	0.000155962	21939	Cd40
04672	Intestinal immune network for IgA production	8.227459873	8.09422E-05	0.000155962	12769	Ccr9
04672	Intestinal immune network for IgA production	8.227459873	8.09422E-05	0.000155962	50723	Icosl
04672	Intestinal immune network for IgA production	8.227459873	8.09422E-05	0.000155962	16193	Il6
04672	Intestinal immune network for IgA production	8.227459873	8.09422E-05	0.000155962	15001	H2-Oa
04672	Intestinal immune network for IgA production	8.227459873	8.09422E-05	0.000155962	12524	Cd86
04975	Fat digestion and absorption	8.227459873	8.09422E-05	0.000155962	11303	Abca1
04975	Fat digestion and absorption	8.227459873	8.09422E-05	0.000155962	110460	Acat2
04975	Fat digestion and absorption	8.227459873	8.09422E-05	0.000155962	19012	Plpp1
04975	Fat digestion and absorption	8.227459873	8.09422E-05	0.000155962	26569	Slc27a4
04975	Fat digestion and absorption	8.227459873	8.09422E-05	0.000155962	67916	Plpp3
04975	Fat digestion and absorption	8.227459873	8.09422E-05	0.000155962	18784	Pla2g5
04975	Fat digestion and absorption	8.227459873	8.09422E-05	0.000155962	224530	Acat3
04934	Cushing syndrome	4.055687892	9.2365E-05	0.000176893	22420	Wnt6
04934	Cushing syndrome	4.055687892	9.2365E-05	0.000176893	12575	Cdkn1a
04934	Cushing syndrome	4.055687892	9.2365E-05	0.000176893	16835	Ldlr
04934	Cushing syndrome	4.055687892	9.2365E-05	0.000176893	11622	Ahr
04934	Cushing syndrome	4.055687892	9.2365E-05	0.000176893	215449	Rap1b
04934	Cushing syndrome	4.055687892	9.2365E-05	0.000176893	12579	Cdkn2b
04934	Cushing syndrome	4.055687892	9.2365E-05	0.000176893	11911	Atf4
04934	Cushing syndrome	4.055687892	9.2365E-05	0.000176893	18798	Plcb4
04934	Cushing syndrome	4.055687892	9.2365E-05	0.000176893	13649	Egfr
04934	Cushing syndrome	4.055687892	9.2365E-05	0.000176893	15370	Nr4a1
04934	Cushing syndrome	4.055687892	9.2365E-05	0.000176893	14362	Fzd1
04934	Cushing syndrome	4.055687892	9.2365E-05	0.000176893	108058	Camk2d
04934	Cushing syndrome	4.055687892	9.2365E-05	0.000176893	231991	Creb5
04550	Signaling pathways regulating pluripotency of stem cells	4.332009484	9.82581E-05	0.000187046	15902	Id2
04550	Signaling pathways regulating pluripotency of stem cells	4.332009484	9.82581E-05	0.000187046	16452	Jak2
04550	Signaling pathways regulating pluripotency of stem cells	4.332009484	9.82581E-05	0.000187046	16323	Inhba
04550	Signaling pathways regulating pluripotency of stem cells	4.332009484	9.82581E-05	0.000187046	17869	Myc
04550	Signaling pathways regulating pluripotency of stem cells	4.332009484	9.82581E-05	0.000187046	22420	Wnt6
04550	Signaling pathways regulating pluripotency of stem cells	4.332009484	9.82581E-05	0.000187046	20482	Skil
04550	Signaling pathways regulating pluripotency of stem cells	4.332009484	9.82581E-05	0.000187046	18708	Pik3r1
04550	Signaling pathways regulating pluripotency of stem cells	4.332009484	9.82581E-05	0.000187046	16878	Lif
04550	Signaling pathways regulating pluripotency of stem cells	4.332009484	9.82581E-05	0.000187046	26415	Mapk13
04550	Signaling pathways regulating pluripotency of stem cells	4.332009484	9.82581E-05	0.000187046	12166	Bmpr1a
04550	Signaling pathways regulating pluripotency of stem cells	4.332009484	9.82581E-05	0.000187046	11479	Acvr1b

04550	Signaling pathways regulating pluripotency of stem cells	4.332009484	9.82581E-05	0.000187046	14362	Fzd1
00564	Glycerophospholipid metabolism	5.157154148	0.000103355	0.000195571	71910	Plpp5
00564	Glycerophospholipid metabolism	5.157154148	0.000103355	0.000195571	18784	Pla2g5
00564	Glycerophospholipid metabolism	5.157154148	0.000103355	0.000195571	270084	Lpcat2
00564	Glycerophospholipid metabolism	5.157154148	0.000103355	0.000195571	19012	Plpp1
00564	Glycerophospholipid metabolism	5.157154148	0.000103355	0.000195571	67916	Plpp3
00564	Glycerophospholipid metabolism	5.157154148	0.000103355	0.000195571	18783	Pla2g4a
00564	Glycerophospholipid metabolism	5.157154148	0.000103355	0.000195571	85031	Pla1a
00564	Glycerophospholipid metabolism	5.157154148	0.000103355	0.000195571	14571	Gpd2
00564	Glycerophospholipid metabolism	5.157154148	0.000103355	0.000195571	380921	Dgkh
00564	Glycerophospholipid metabolism	5.157154148	0.000103355	0.000195571	68262	Agpat4
04919	Thyroid hormone signaling pathway	4.632843476	0.000113914	0.000214266	20846	Stat1
04919	Thyroid hormone signaling pathway	4.632843476	0.000113914	0.000214266	18708	Pik3r1
04919	Thyroid hormone signaling pathway	4.632843476	0.000113914	0.000214266	72472	Slc16a10
04919	Thyroid hormone signaling pathway	4.632843476	0.000113914	0.000214266	20779	Src
04919	Thyroid hormone signaling pathway	4.632843476	0.000113914	0.000214266	17869	Myc
04919	Thyroid hormone signaling pathway	4.632843476	0.000113914	0.000214266	56717	Mtor
04919	Thyroid hormone signaling pathway	4.632843476	0.000113914	0.000214266	18641	Pfkl
04919	Thyroid hormone signaling pathway	4.632843476	0.000113914	0.000214266	18128	Notch1
04919	Thyroid hormone signaling pathway	4.632843476	0.000113914	0.000214266	18798	Plcb4
04919	Thyroid hormone signaling pathway	4.632843476	0.000113914	0.000214266	15251	Hif1a
04919	Thyroid hormone signaling pathway	4.632843476	0.000113914	0.000214266	20525	Slc2a1
00561	Glycerolipid metabolism	6.5213046	0.000120883	0.0002247	68262	Agpat4
00561	Glycerolipid metabolism	6.5213046	0.000120883	0.0002247	71910	Plpp5
00561	Glycerolipid metabolism	6.5213046	0.000120883	0.0002247	19012	Plpp1
00561	Glycerolipid metabolism	6.5213046	0.000120883	0.0002247	16891	Lipg
00561	Glycerolipid metabolism	6.5213046	0.000120883	0.0002247	14187	Akr1b8
00561	Glycerolipid metabolism	6.5213046	0.000120883	0.0002247	380921	Dgkh
00561	Glycerolipid metabolism	6.5213046	0.000120883	0.0002247	67916	Plpp3
00561	Glycerolipid metabolism	6.5213046	0.000120883	0.0002247	14933	Gk
01212	Fatty acid metabolism	6.5213046	0.000120883	0.0002247	224530	Acat3
01212	Fatty acid metabolism	6.5213046	0.000120883	0.0002247	110460	Acat2
01212	Fatty acid metabolism	6.5213046	0.000120883	0.0002247	20250	Scd2
01212	Fatty acid metabolism	6.5213046	0.000120883	0.0002247	50790	Acsl4
01212	Fatty acid metabolism	6.5213046	0.000120883	0.0002247	56348	Hsd17b12
01212	Fatty acid metabolism	6.5213046	0.000120883	0.0002247	14081	Acsl1
01212	Fatty acid metabolism	6.5213046	0.000120883	0.0002247	433256	Acsl5
01212	Fatty acid metabolism	6.5213046	0.000120883	0.0002247	94180	Acsbg1
04144	Endocytosis	3.158756916	0.00014622	0.000270207	16835	Ldlr
04144	Endocytosis	3.158756916	0.00014622	0.000270207	13649	Egfr
04144	Endocytosis	3.158756916	0.00014622	0.000270207	14990	H2-M2
04144	Endocytosis	3.158756916	0.00014622	0.000270207	211673	Arfgef1

04144	Endocytosis	3.158756916	0.00014622	0.000270207	75767	Rab11fip1
04144	Endocytosis	3.158756916	0.00014622	0.000270207	18719	Pip5k1b
04144	Endocytosis	3.158756916	0.00014622	0.000270207	15024	H2-T10
04144	Endocytosis	3.158756916	0.00014622	0.000270207	73728	Psd
04144	Endocytosis	3.158756916	0.00014622	0.000270207	110558	H2-Q9
04144	Endocytosis	3.158756916	0.00014622	0.000270207	15018	H2-Q7
04144	Endocytosis	3.158756916	0.00014622	0.000270207	19325	Rab10
04144	Endocytosis	3.158756916	0.00014622	0.000270207	15039	H2-T22
04144	Endocytosis	3.158756916	0.00014622	0.000270207	20779	Src
04144	Endocytosis	3.158756916	0.00014622	0.000270207	13660	Ehd1
04144	Endocytosis	3.158756916	0.00014622	0.000270207	12389	Cav1
04144	Endocytosis	3.158756916	0.00014622	0.000270207	216238	Eea1
04144	Endocytosis	3.158756916	0.00014622	0.000270207	18720	Pip5k1a
04930	Type II diabetes mellitus	7.370432803	0.000169711	0.000311795	15277	Hk2
04930	Type II diabetes mellitus	7.370432803	0.000169711	0.000311795	18708	Pik3r1
04930	Type II diabetes mellitus	7.370432803	0.000169711	0.000311795	12702	Socs3
04930	Type II diabetes mellitus	7.370432803	0.000169711	0.000311795	21926	Tnf
04930	Type II diabetes mellitus	7.370432803	0.000169711	0.000311795	12703	Socs1
04930	Type II diabetes mellitus	7.370432803	0.000169711	0.000311795	216233	Socs2
04930	Type II diabetes mellitus	7.370432803	0.000169711	0.000311795	56717	Mtor
04022	cGMP-PKG signaling pathway	3.797811783	0.000183648	0.000335449	381290	Atp2b4
04022	cGMP-PKG signaling pathway	3.797811783	0.000183648	0.000335449	11911	Atf4
04022	cGMP-PKG signaling pathway	3.797811783	0.000183648	0.000335449	22323	Vasp
04022	cGMP-PKG signaling pathway	3.797811783	0.000183648	0.000335449	12062	Bdkrb2
04022	cGMP-PKG signaling pathway	3.797811783	0.000183648	0.000335449	30955	Pik3cg
04022	cGMP-PKG signaling pathway	3.797811783	0.000183648	0.000335449	16531	Kcnma1
04022	cGMP-PKG signaling pathway	3.797811783	0.000183648	0.000335449	19057	Ppp3cc
04022	cGMP-PKG signaling pathway	3.797811783	0.000183648	0.000335449	228785	Mylk2
04022	cGMP-PKG signaling pathway	3.797811783	0.000183648	0.000335449	242202	Pde5a
04022	cGMP-PKG signaling pathway	3.797811783	0.000183648	0.000335449	18798	Plcb4
04022	cGMP-PKG signaling pathway	3.797811783	0.000183648	0.000335449	13618	Ednrb
04022	cGMP-PKG signaling pathway	3.797811783	0.000183648	0.000335449	320207	Pik3r5
04022	cGMP-PKG signaling pathway	3.797811783	0.000183648	0.000335449	231991	Creb5
00071	Fatty acid degradation	6.803476434	0.0002879	0.000522852	433256	Acsl5
00071	Fatty acid degradation	6.803476434	0.0002879	0.000522852	224530	Acat3
00071	Fatty acid degradation	6.803476434	0.0002879	0.000522852	14081	Acsl1
00071	Fatty acid degradation	6.803476434	0.0002879	0.000522852	110460	Acat2
00071	Fatty acid degradation	6.803476434	0.0002879	0.000522852	11529	Adh7
00071	Fatty acid degradation	6.803476434	0.0002879	0.000522852	94180	Acsbg1
00071	Fatty acid degradation	6.803476434	0.0002879	0.000522852	50790	Acsl4
04211	Longevity regulating pathway	5.054011065	0.000304887	0.000550539	20656	Sod2
04211	Longevity regulating pathway	5.054011065	0.000304887	0.000550539	56717	Mtor

04211	Longevity regulating pathway	5.054011065	0.000304887	0.000550539	108079	Prkaa2
04211	Longevity regulating pathway	5.054011065	0.000304887	0.000550539	11911	Atf4
04211	Longevity regulating pathway	5.054011065	0.000304887	0.000550539	105787	Prkaa1
04211	Longevity regulating pathway	5.054011065	0.000304887	0.000550539	13684	Eif4e
04211	Longevity regulating pathway	5.054011065	0.000304887	0.000550539	231991	Creb5
04211	Longevity regulating pathway	5.054011065	0.000304887	0.000550539	18708	Pik3r1
04211	Longevity regulating pathway	5.054011065	0.000304887	0.000550539	18033	Nfkb1
04915	Estrogen signaling pathway	4.148815053	0.000314089	0.000563932	231991	Creb5
04915	Estrogen signaling pathway	4.148815053	0.000314089	0.000563932	18798	Plcb4
04915	Estrogen signaling pathway	4.148815053	0.000314089	0.000563932	18708	Pik3r1
04915	Estrogen signaling pathway	4.148815053	0.000314089	0.000563932	15200	Hbegf
04915	Estrogen signaling pathway	4.148815053	0.000314089	0.000563932	15519	Hsp90aa1
04915	Estrogen signaling pathway	4.148815053	0.000314089	0.000563932	17390	Mmp2
04915	Estrogen signaling pathway	4.148815053	0.000314089	0.000563932	17395	Mmp9
04915	Estrogen signaling pathway	4.148815053	0.000314089	0.000563932	22027	Hsp90b1
04915	Estrogen signaling pathway	4.148815053	0.000314089	0.000563932	13649	Egfr
04915	Estrogen signaling pathway	4.148815053	0.000314089	0.000563932	11911	Atf4
04915	Estrogen signaling pathway	4.148815053	0.000314089	0.000563932	20779	Src
04725	Cholinergic synapse	4.512509879	0.000325731	0.00058153	30955	Pik3cg
04725	Cholinergic synapse	4.512509879	0.000325731	0.00058153	14701	Gng12
04725	Cholinergic synapse	4.512509879	0.000325731	0.00058153	11911	Atf4
04725	Cholinergic synapse	4.512509879	0.000325731	0.00058153	231991	Creb5
04725	Cholinergic synapse	4.512509879	0.000325731	0.00058153	16452	Jak2
04725	Cholinergic synapse	4.512509879	0.000325731	0.00058153	18708	Pik3r1
04725	Cholinergic synapse	4.512509879	0.000325731	0.00058153	18798	Plcb4
04725	Cholinergic synapse	4.512509879	0.000325731	0.00058153	108058	Camk2d
04725	Cholinergic synapse	4.512509879	0.000325731	0.00058153	320207	Pik3r5
04725	Cholinergic synapse	4.512509879	0.000325731	0.00058153	226922	Kcnq5
00983	Drug metabolism - other enzymes	4.944141259	0.000362084	0.000642801	54369	Nme6
00983	Drug metabolism - other enzymes	4.944141259	0.000362084	0.000642801	100042295	Gm3776
00983	Drug metabolism - other enzymes	4.944141259	0.000362084	0.000642801	14858	Gsta2
00983	Drug metabolism - other enzymes	4.944141259	0.000362084	0.000642801	14863	Gstm2
00983	Drug metabolism - other enzymes	4.944141259	0.000362084	0.000642801	80914	Uck2
00983	Drug metabolism - other enzymes	4.944141259	0.000362084	0.000642801	14857	Gsta1
00983	Drug metabolism - other enzymes	4.944141259	0.000362084	0.000642801	22271	Upp1
00983	Drug metabolism - other enzymes	4.944141259	0.000362084	0.000642801	14860	Gsta4
00983	Drug metabolism - other enzymes	4.944141259	0.000362084	0.000642801	64705	Dpys
04310	Wnt signaling pathway	3.7437119	0.00040786	0.000720022	231201	AF366264
04310	Wnt signaling pathway	3.7437119	0.00040786	0.000720022	12444	Ccnd2
04310	Wnt signaling pathway	3.7437119	0.00040786	0.000720022	208846	Daam1
04310	Wnt signaling pathway	3.7437119	0.00040786	0.000720022	19057	Ppp3cc
04310	Wnt signaling pathway	3.7437119	0.00040786	0.000720022	18798	Plcb4

04310	Wnt signaling pathway	3.7437119	0.00040786	0.000720022	114671	4930444G20Rik
04310	Wnt signaling pathway	3.7437119	0.00040786	0.000720022	14362	Fzd1
04310	Wnt signaling pathway	3.7437119	0.00040786	0.000720022	19015	Ppard
04310	Wnt signaling pathway	3.7437119	0.00040786	0.000720022	22420	Wnt6
04310	Wnt signaling pathway	3.7437119	0.00040786	0.000720022	14283	Fosl1
04310	Wnt signaling pathway	3.7437119	0.00040786	0.000720022	17869	Myc
04310	Wnt signaling pathway	3.7437119	0.00040786	0.000720022	108058	Camk2d
00620	Pyruvate metabolism	7.775401638	0.000443261	0.000778169	17448	Mdh2
00620	Pyruvate metabolism	7.775401638	0.000443261	0.000778169	76238	Grhpr
00620	Pyruvate metabolism	7.775401638	0.000443261	0.000778169	16828	Ldha
00620	Pyruvate metabolism	7.775401638	0.000443261	0.000778169	110460	Acat2
00620	Pyruvate metabolism	7.775401638	0.000443261	0.000778169	224530	Acat3
00620	Pyruvate metabolism	7.775401638	0.000443261	0.000778169	18563	Pcx
00350	Tyrosine metabolism	7.581016598	0.000512781	0.000895242	17319	Mif
00350	Tyrosine metabolism	7.581016598	0.000512781	0.000895242	11670	Aldh3a1
00350	Tyrosine metabolism	7.581016598	0.000512781	0.000895242	11761	Aox1
00350	Tyrosine metabolism	7.581016598	0.000512781	0.000895242	11529	Adh7
00350	Tyrosine metabolism	7.581016598	0.000512781	0.000895242	14204	Il4i1
00350	Tyrosine metabolism	7.581016598	0.000512781	0.000895242	73988	4930438A08Rik
05100	Bacterial invasion of epithelial cells	5.320011647	0.000528406	0.000917452	12389	Cav1
05100	Bacterial invasion of epithelial cells	5.320011647	0.000528406	0.000917452	12927	Bcar1
05100	Bacterial invasion of epithelial cells	5.320011647	0.000528406	0.000917452	16402	Itga5
05100	Bacterial invasion of epithelial cells	5.320011647	0.000528406	0.000917452	15163	Hcls1
05100	Bacterial invasion of epithelial cells	5.320011647	0.000528406	0.000917452	20779	Src
05100	Bacterial invasion of epithelial cells	5.320011647	0.000528406	0.000917452	17295	Met
05100	Bacterial invasion of epithelial cells	5.320011647	0.000528406	0.000917452	18708	Pik3r1
05100	Bacterial invasion of epithelial cells	5.320011647	0.000528406	0.000917452	22330	Vcl
05412	Arrhythmogenic right ventricular cardiomyopathy	5.250920587	0.00057952	0.001000701	319480	Itga11
05412	Arrhythmogenic right ventricular cardiomyopathy	5.250920587	0.00057952	0.001000701	16402	Itga5
05412	Arrhythmogenic right ventricular cardiomyopathy	5.250920587	0.00057952	0.001000701	14609	Gja1
05412	Arrhythmogenic right ventricular cardiomyopathy	5.250920587	0.00057952	0.001000701	54378	Cacng6
05412	Arrhythmogenic right ventricular cardiomyopathy	5.250920587	0.00057952	0.001000701	109700	Itga1
05412	Arrhythmogenic right ventricular cardiomyopathy	5.250920587	0.00057952	0.001000701	81905	Cacng8
05412	Arrhythmogenic right ventricular cardiomyopathy	5.250920587	0.00057952	0.001000701	320910	Itgb8
05412	Arrhythmogenic right ventricular cardiomyopathy	5.250920587	0.00057952	0.001000701	104099	Itga9
00450	Selenocompound metabolism	11.89179074	0.001182631	0.002031041	50493	Txnrd1
00450	Selenocompound metabolism	11.89179074	0.001182631	0.002031041	23972	Papss2
00450	Selenocompound metabolism	11.89179074	0.001182631	0.002031041	229905	Kyat3
00450	Selenocompound metabolism	11.89179074	0.001182631	0.002031041	107869	Cth
04974	Protein digestion and absorption	4.211675888	0.001231278	0.002103156	20510	Slc1a1
04974	Protein digestion and absorption	4.211675888	0.001231278	0.002103156	72472	Slc16a10
04974	Protein digestion and absorption	4.211675888	0.001231278	0.002103156	53867	Col5a3

04974	Protein digestion and absorption	4.211675888	0.001231278	0.002103156	12831	Col5a1
04974	Protein digestion and absorption	4.211675888	0.001231278	0.002103156	50934	Slc7a8
04974	Protein digestion and absorption	4.211675888	0.001231278	0.002103156	12827	Col4a2
04974	Protein digestion and absorption	4.211675888	0.001231278	0.002103156	12823	Col19a1
04974	Protein digestion and absorption	4.211675888	0.001231278	0.002103156	17254	Slc3a2
04974	Protein digestion and absorption	4.211675888	0.001231278	0.002103156	373864	Col27a1
04146	Peroxisome	4.701405642	0.001250167	0.002112582	54683	Prdx5
04146	Peroxisome	4.701405642	0.001250167	0.002112582	18477	Prdx1
04146	Peroxisome	4.701405642	0.001250167	0.002112582	14081	Acsl1
04146	Peroxisome	4.701405642	0.001250167	0.002112582	20656	Sod2
04146	Peroxisome	4.701405642	0.001250167	0.002112582	50790	Acsl4
04146	Peroxisome	4.701405642	0.001250167	0.002112582	433256	Acsl5
04146	Peroxisome	4.701405642	0.001250167	0.002112582	13850	Ephx2
04146	Peroxisome	4.701405642	0.001250167	0.002112582	18126	Nos2
04540	Gap junction	4.701405642	0.001250167	0.002112582	13649	Egfr
04540	Gap junction	4.701405642	0.001250167	0.002112582	14745	Lpar1
04540	Gap junction	4.701405642	0.001250167	0.002112582	20779	Src
04540	Gap junction	4.701405642	0.001250167	0.002112582	14609	Gja1
04540	Gap junction	4.701405642	0.001250167	0.002112582	22142	Tuba1a
04540	Gap junction	4.701405642	0.001250167	0.002112582	18798	Plcb4
04540	Gap junction	4.701405642	0.001250167	0.002112582	22145	Tuba4a
04540	Gap junction	4.701405642	0.001250167	0.002112582	22153	Tubb4a
00565	Ether lipid metabolism	6.317513831	0.001433245	0.002409071	270084	Lpcat2
00565	Ether lipid metabolism	6.317513831	0.001433245	0.002409071	18606	Enpp2
00565	Ether lipid metabolism	6.317513831	0.001433245	0.002409071	18783	Pla2g4a
00565	Ether lipid metabolism	6.317513831	0.001433245	0.002409071	18784	Pla2g5
00565	Ether lipid metabolism	6.317513831	0.001433245	0.002409071	19012	Plpp1
00565	Ether lipid metabolism	6.317513831	0.001433245	0.002409071	67916	Plpp3
04720	Long-term potentiation	5.280310068	0.001447669	0.002420442	19057	Ppp3cc
04720	Long-term potentiation	5.280310068	0.001447669	0.002420442	18798	Plcb4
04720	Long-term potentiation	5.280310068	0.001447669	0.002420442	108058	Camk2d
04720	Long-term potentiation	5.280310068	0.001447669	0.002420442	11911	Atf4
04720	Long-term potentiation	5.280310068	0.001447669	0.002420442	215449	Rap1b
04720	Long-term potentiation	5.280310068	0.001447669	0.002420442	20112	Rps6ka2
04720	Long-term potentiation	5.280310068	0.001447669	0.002420442	14814	Grin2d
04979	Cholesterol metabolism	6.188584978	0.001606076	0.002671157	16976	Lrpap1
04979	Cholesterol metabolism	6.188584978	0.001606076	0.002671157	16835	Ldlr
04979	Cholesterol metabolism	6.188584978	0.001606076	0.002671157	16891	Lipg
04979	Cholesterol metabolism	6.188584978	0.001606076	0.002671157	11303	Abca1
04979	Cholesterol metabolism	6.188584978	0.001606076	0.002671157	12257	Tspo
04979	Cholesterol metabolism	6.188584978	0.001606076	0.002671157	223920	Soat2
02010	ABC transporters	6.064813278	0.001794751	0.002969326	11303	Abca1

02010	ABC transporters	6.064813278	0.001794751	0.002969326	18671	Abcb1a
02010	ABC transporters	6.064813278	0.001794751	0.002969326	192663	Abcg4
02010	ABC transporters	6.064813278	0.001794751	0.002969326	17250	Abcc1
02010	ABC transporters	6.064813278	0.001794751	0.002969326	239273	Abcc4
02010	ABC transporters	6.064813278	0.001794751	0.002969326	27416	Abcc5
04972	Pancreatic secretion	3.990008736	0.001837072	0.003023514	215449	Rap1b
04972	Pancreatic secretion	3.990008736	0.001837072	0.003023514	12494	Cd38
04972	Pancreatic secretion	3.990008736	0.001837072	0.003023514	18798	Plcb4
04972	Pancreatic secretion	3.990008736	0.001837072	0.003023514	381290	Atp2b4
04972	Pancreatic secretion	3.990008736	0.001837072	0.003023514	12349	Car2
04972	Pancreatic secretion	3.990008736	0.001837072	0.003023514	229933	Clca2
04972	Pancreatic secretion	3.990008736	0.001837072	0.003023514	18784	Pla2g5
04972	Pancreatic secretion	3.990008736	0.001837072	0.003023514	12182	Bst1
04972	Pancreatic secretion	3.990008736	0.001837072	0.003023514	16531	Kcnma1
00061	Fatty acid biosynthesis	10.64002329	0.001866214	0.003039813	433256	Acsl5
00061	Fatty acid biosynthesis	10.64002329	0.001866214	0.003039813	14081	Acsl1
00061	Fatty acid biosynthesis	10.64002329	0.001866214	0.003039813	50790	Acsl4
00061	Fatty acid biosynthesis	10.64002329	0.001866214	0.003039813	94180	Acsbg1
00670	One carbon pool by folate	10.64002329	0.001866214	0.003039813	270685	Mthfd1l
00670	One carbon pool by folate	10.64002329	0.001866214	0.003039813	17768	Mthfd2
00670	One carbon pool by folate	10.64002329	0.001866214	0.003039813	216188	Aldh1l2
00670	One carbon pool by folate	10.64002329	0.001866214	0.003039813	665563	Mthfd2l
05218	Melanoma	4.913621869	0.002251708	0.003648922	13649	Egfr
05218	Melanoma	4.913621869	0.002251708	0.003648922	14164	Fgf1
05218	Melanoma	4.913621869	0.002251708	0.003648922	17873	Gadd45b
05218	Melanoma	4.913621869	0.002251708	0.003648922	13197	Gadd45a
05218	Melanoma	4.913621869	0.002251708	0.003648922	12575	Cdkn1a
05218	Melanoma	4.913621869	0.002251708	0.003648922	18708	Pik3r1
05218	Melanoma	4.913621869	0.002251708	0.003648922	17295	Met
04120	Ubiquitin mediated proteolysis	3.485524872	0.002647891	0.004242948	22201	Uba1
04120	Ubiquitin mediated proteolysis	3.485524872	0.002647891	0.004242948	12702	Socs3
04120	Ubiquitin mediated proteolysis	3.485524872	0.002647891	0.004242948	56791	Ube2l6
04120	Ubiquitin mediated proteolysis	3.485524872	0.002647891	0.004242948	67921	Ube2f
04120	Ubiquitin mediated proteolysis	3.485524872	0.002647891	0.004242948	12703	Socs1
04120	Ubiquitin mediated proteolysis	3.485524872	0.002647891	0.004242948	26374	Cop1
04120	Ubiquitin mediated proteolysis	3.485524872	0.002647891	0.004242948	77891	Ube2s
04120	Ubiquitin mediated proteolysis	3.485524872	0.002647891	0.004242948	67923	Eloc
04120	Ubiquitin mediated proteolysis	3.485524872	0.002647891	0.004242948	11796	Birc3
04120	Ubiquitin mediated proteolysis	3.485524872	0.002647891	0.004242948	218793	Ube2e2
04918	Thyroid hormone synthesis	4.780821278	0.002658556	0.004242948	22027	Hsp90b1
04918	Thyroid hormone synthesis	4.780821278	0.002658556	0.004242948	14828	Hspa5
04918	Thyroid hormone synthesis	4.780821278	0.002658556	0.004242948	14782	Gsr

04918	Thyroid hormone synthesis	4.780821278	0.002658556	0.004242948	231991	Creb5
04918	Thyroid hormone synthesis	4.780821278	0.002658556	0.004242948	12304	Pdia4
04918	Thyroid hormone synthesis	4.780821278	0.002658556	0.004242948	18798	Plcb4
04918	Thyroid hormone synthesis	4.780821278	0.002658556	0.004242948	11911	Atf4
05214	Glioma	4.780821278	0.002658556	0.004242948	108058	Camk2d
05214	Glioma	4.780821278	0.002658556	0.004242948	18708	Pik3r1
05214	Glioma	4.780821278	0.002658556	0.004242948	17873	Gadd45b
05214	Glioma	4.780821278	0.002658556	0.004242948	13197	Gadd45a
05214	Glioma	4.780821278	0.002658556	0.004242948	56717	Mtor
05214	Glioma	4.780821278	0.002658556	0.004242948	12575	Cdkn1a
05214	Glioma	4.780821278	0.002658556	0.004242948	13649	Egfr
00051	Fructose and mannose metabolism	7.019459813	0.002727821	0.004331615	14187	Akr1b8
00051	Fructose and mannose metabolism	7.019459813	0.002727821	0.004331615	170768	Pfkfb3
00051	Fructose and mannose metabolism	7.019459813	0.002727821	0.004331615	18641	Pfkl
00051	Fructose and mannose metabolism	7.019459813	0.002727821	0.004331615	15277	Hk2
00051	Fructose and mannose metabolism	7.019459813	0.002727821	0.004331615	21991	Tpi1
03008	Ribosome biogenesis in eukaryotes	3.759181784	0.002833225	0.004476496	55989	Nop58
03008	Ribosome biogenesis in eukaryotes	3.759181784	0.002833225	0.004476496	30877	Gnl3
03008	Ribosome biogenesis in eukaryotes	3.759181784	0.002833225	0.004476496	68147	Gar1
03008	Ribosome biogenesis in eukaryotes	3.759181784	0.002833225	0.004476496	59028	Rcl1
03008	Ribosome biogenesis in eukaryotes	3.759181784	0.002833225	0.004476496	69237	Gtpbp4
03008	Ribosome biogenesis in eukaryotes	3.759181784	0.002833225	0.004476496	67724	Pop1
03008	Ribosome biogenesis in eukaryotes	3.759181784	0.002833225	0.004476496	16418	Eif6
03008	Ribosome biogenesis in eukaryotes	3.759181784	0.002833225	0.004476496	102216272	Ak6
03008	Ribosome biogenesis in eukaryotes	3.759181784	0.002833225	0.004476496	72515	Wdr43
05322	Systemic lupus erythematosus	3.414872341	0.00310279	0.004878018	15001	H2-Oa
05322	Systemic lupus erythematosus	3.414872341	0.00310279	0.004878018	21926	Tnf
05322	Systemic lupus erythematosus	3.414872341	0.00310279	0.004878018	20821	Trim21
05322	Systemic lupus erythematosus	3.414872341	0.00310279	0.004878018	16153	Il10
05322	Systemic lupus erythematosus	3.414872341	0.00310279	0.004878018	21939	Cd40
05322	Systemic lupus erythematosus	3.414872341	0.00310279	0.004878018	667277	C1rb
05322	Systemic lupus erythematosus	3.414872341	0.00310279	0.004878018	12524	Cd86
05322	Systemic lupus erythematosus	3.414872341	0.00310279	0.004878018	50908	C1s1
05322	Systemic lupus erythematosus	3.414872341	0.00310279	0.004878018	15978	Ifng
05322	Systemic lupus erythematosus	3.414872341	0.00310279	0.004878018	12266	C3
05220	Chronic myeloid leukemia	4.655010191	0.003121552	0.004883219	18708	Pik3r1
05220	Chronic myeloid leukemia	4.655010191	0.003121552	0.004883219	17869	Myc
05220	Chronic myeloid leukemia	4.655010191	0.003121552	0.004883219	13197	Gadd45a
05220	Chronic myeloid leukemia	4.655010191	0.003121552	0.004883219	18033	Nfkb1
05220	Chronic myeloid leukemia	4.655010191	0.003121552	0.004883219	18035	Nfkbia
05220	Chronic myeloid leukemia	4.655010191	0.003121552	0.004883219	12575	Cdkn1a
05220	Chronic myeloid leukemia	4.655010191	0.003121552	0.004883219	17873	Gadd45b

04721	Synaptic vesicle cycle	4.594555514	0.003375755	0.005254869	12890	Cplx2
04721	Synaptic vesicle cycle	4.594555514	0.003375755	0.005254869	20510	Slc1a1
04721	Synaptic vesicle cycle	4.594555514	0.003375755	0.005254869	11973	Atp6v1e1
04721	Synaptic vesicle cycle	4.594555514	0.003375755	0.005254869	20511	Slc1a2
04721	Synaptic vesicle cycle	4.594555514	0.003375755	0.005254869	14412	Slc6a13
04721	Synaptic vesicle cycle	4.594555514	0.003375755	0.005254869	14411	Slc6a12
04721	Synaptic vesicle cycle	4.594555514	0.003375755	0.005254869	14664	Slc6a9
04925	Aldosterone synthesis and secretion	3.963930247	0.003918688	0.006070125	231991	Creb5
04925	Aldosterone synthesis and secretion	3.963930247	0.003918688	0.006070125	18798	Plcb4
04925	Aldosterone synthesis and secretion	3.963930247	0.003918688	0.006070125	11911	Atf4
04925	Aldosterone synthesis and secretion	3.963930247	0.003918688	0.006070125	381290	Atp2b4
04925	Aldosterone synthesis and secretion	3.963930247	0.003918688	0.006070125	16835	Ldlr
04925	Aldosterone synthesis and secretion	3.963930247	0.003918688	0.006070125	18227	Nr4a2
04925	Aldosterone synthesis and secretion	3.963930247	0.003918688	0.006070125	15370	Nr4a1
04925	Aldosterone synthesis and secretion	3.963930247	0.003918688	0.006070125	108058	Camk2d
00250	Alanine, aspartate and glutamate metabolism	6.479501365	0.00396714	0.006111551	14584	Gfpt2
00250	Alanine, aspartate and glutamate metabolism	6.479501365	0.00396714	0.006111551	73988	4930438A08Rik
00250	Alanine, aspartate and glutamate metabolism	6.479501365	0.00396714	0.006111551	11898	Ass1
00250	Alanine, aspartate and glutamate metabolism	6.479501365	0.00396714	0.006111551	27053	Asns
00250	Alanine, aspartate and glutamate metabolism	6.479501365	0.00396714	0.006111551	14204	Il4i1
05213	Endometrial cancer	5.228287309	0.00399878	0.006111551	17873	Gadd45b
05213	Endometrial cancer	5.228287309	0.00399878	0.006111551	13197	Gadd45a
05213	Endometrial cancer	5.228287309	0.00399878	0.006111551	17869	Myc
05213	Endometrial cancer	5.228287309	0.00399878	0.006111551	13649	Egfr
05213	Endometrial cancer	5.228287309	0.00399878	0.006111551	18708	Pik3r1
05213	Endometrial cancer	5.228287309	0.00399878	0.006111551	12575	Cdkn1a
00360	Phenylalanine metabolism	8.789584461	0.004003453	0.006111551	11670	Aldh3a1
00360	Phenylalanine metabolism	8.789584461	0.004003453	0.006111551	17319	Mif
00360	Phenylalanine metabolism	8.789584461	0.004003453	0.006111551	73988	4930438A08Rik
00360	Phenylalanine metabolism	8.789584461	0.004003453	0.006111551	14204	Il4i1
05310	Asthma	8.086417704	0.005542428	0.008420226	21926	Tnf
05310	Asthma	8.086417704	0.005542428	0.008420226	15001	H2-Oa
05310	Asthma	8.086417704	0.005542428	0.008420226	21939	Cd40
05310	Asthma	8.086417704	0.005542428	0.008420226	16153	Il10
00072	Synthesis and degradation of ketone bodies	12.63502766	0.005951234	0.008998039	208715	Hmgcs1
00072	Synthesis and degradation of ketone bodies	12.63502766	0.005951234	0.008998039	224530	Acat3
00072	Synthesis and degradation of ketone bodies	12.63502766	0.005951234	0.008998039	110460	Acat2
05217	Basal cell carcinoma	4.813343871	0.006175453	0.009292587	12156	Bmp2
05217	Basal cell carcinoma	4.813343871	0.006175453	0.009292587	22420	Wnt6
05217	Basal cell carcinoma	4.813343871	0.006175453	0.009292587	12575	Cdkn1a
05217	Basal cell carcinoma	4.813343871	0.006175453	0.009292587	14362	Fzd1
05217	Basal cell carcinoma	4.813343871	0.006175453	0.009292587	13197	Gadd45a

05217	Basal cell carcinoma	4.813343871	0.006175453	0.009292587	17873	Gadd45b
00790	Folate biosynthesis	7.775401638	0.006448218	0.009648471	14187	Akr1b8
00790	Folate biosynthesis	7.775401638	0.006448218	0.009648471	56738	Mocs1
00790	Folate biosynthesis	7.775401638	0.006448218	0.009648471	268566	Gphn
00790	Folate biosynthesis	7.775401638	0.006448218	0.009648471	14528	Gch1
04911	Insulin secretion	4.113729937	0.006473025	0.009648471	116838	Rims2
04911	Insulin secretion	4.113729937	0.006473025	0.009648471	108058	Camk2d
04911	Insulin secretion	4.113729937	0.006473025	0.009648471	231991	Creb5
04911	Insulin secretion	4.113729937	0.006473025	0.009648471	16531	Kcnma1
04911	Insulin secretion	4.113729937	0.006473025	0.009648471	18798	Plcb4
04911	Insulin secretion	4.113729937	0.006473025	0.009648471	20525	Slc2a1
04911	Insulin secretion	4.113729937	0.006473025	0.009648471	11911	Atf4
00601	Glycosphingolipid biosynthesis - lacto and neolacto series	7.4874238	0.007451944	0.011003806	14538	Gcnt2
00601	Glycosphingolipid biosynthesis - lacto and neolacto series	7.4874238	0.007451944	0.011003806	108105	B3gnt5
00601	Glycosphingolipid biosynthesis - lacto and neolacto series	7.4874238	0.007451944	0.011003806	26878	B3galt2
00601	Glycosphingolipid biosynthesis - lacto and neolacto series	7.4874238	0.007451944	0.011003806	20441	St3gal3
04392	Hippo signaling pathway - multiple species	7.4874238	0.007451944	0.011003806	319710	Frmd6
04392	Hippo signaling pathway - multiple species	7.4874238	0.007451944	0.011003806	213391	Rassf4
04392	Hippo signaling pathway - multiple species	7.4874238	0.007451944	0.011003806	64010	Sav1
04392	Hippo signaling pathway - multiple species	7.4874238	0.007451944	0.011003806	73246	Rassf6
04137	Mitophagy - animal	4.594555514	0.007855517	0.011545783	18412	Sqstm1
04137	Mitophagy - animal	4.594555514	0.007855517	0.011545783	12176	Bnip3
04137	Mitophagy - animal	4.594555514	0.007855517	0.011545783	56480	Tbk1
04137	Mitophagy - animal	4.594555514	0.007855517	0.011545783	20779	Src
04137	Mitophagy - animal	4.594555514	0.007855517	0.011545783	15251	Hif1a
04137	Mitophagy - animal	4.594555514	0.007855517	0.011545783	11911	Atf4
05030	Cocaine addiction	5.264594859	0.010192682	0.014911517	18033	Nfkb1
05030	Cocaine addiction	5.264594859	0.010192682	0.014911517	19739	Rgs9
05030	Cocaine addiction	5.264594859	0.010192682	0.014911517	14814	Grin2d
05030	Cocaine addiction	5.264594859	0.010192682	0.014911517	11911	Atf4
05030	Cocaine addiction	5.264594859	0.010192682	0.014911517	231991	Creb5
04520	Adherens junction	4.270995266	0.011394746	0.01659327	13649	Egfr
04520	Adherens junction	4.270995266	0.011394746	0.01659327	19246	Ptpn1
04520	Adherens junction	4.270995266	0.011394746	0.01659327	22330	Vcl
04520	Adherens junction	4.270995266	0.011394746	0.01659327	20779	Src
04520	Adherens junction	4.270995266	0.011394746	0.01659327	17295	Met
04520	Adherens junction	4.270995266	0.011394746	0.01659327	19271	Ptprj
00600	Sphingolipid metabolism	5.054011065	0.012209954	0.017560882	67916	Plpp3
00600	Sphingolipid metabolism	5.054011065	0.012209954	0.017560882	19012	Plpp1
00600	Sphingolipid metabolism	5.054011065	0.012209954	0.017560882	20698	Sphk1
00600	Sphingolipid metabolism	5.054011065	0.012209954	0.017560882	241447	Cers6
00600	Sphingolipid metabolism	5.054011065	0.012209954	0.017560882	20773	Sptlc2

00562	Inositol phosphate metabolism	4.211675888	0.012225931	0.017560882	18720	Pip5k1a
00562	Inositol phosphate metabolism	4.211675888	0.012225931	0.017560882	18719	Pip5k1b
00562	Inositol phosphate metabolism	4.211675888	0.012225931	0.017560882	17772	Mtm1
00562	Inositol phosphate metabolism	4.211675888	0.012225931	0.017560882	18798	Plcb4
00562	Inositol phosphate metabolism	4.211675888	0.012225931	0.017560882	21991	Tpi1
00562	Inositol phosphate metabolism	4.211675888	0.012225931	0.017560882	30955	Pik3cg
05223	Non-small cell lung cancer	4.211675888	0.012225931	0.017560882	17295	Met
05223	Non-small cell lung cancer	4.211675888	0.012225931	0.017560882	17873	Gadd45b
05223	Non-small cell lung cancer	4.211675888	0.012225931	0.017560882	13649	Egfr
05223	Non-small cell lung cancer	4.211675888	0.012225931	0.017560882	13197	Gadd45a
05223	Non-small cell lung cancer	4.211675888	0.012225931	0.017560882	12575	Cdkn1a
05223	Non-small cell lung cancer	4.211675888	0.012225931	0.017560882	18708	Pik3r1
00052	Galactose metabolism	6.317513831	0.014120703	0.020009606	66681	Pgm2
00052	Galactose metabolism	6.317513831	0.014120703	0.020009606	15277	Hk2
00052	Galactose metabolism	6.317513831	0.014120703	0.020009606	18641	Pfkl
00052	Galactose metabolism	6.317513831	0.014120703	0.020009606	14187	Akr1b8
00630	Glyoxylate and dicarboxylate metabolism	6.317513831	0.014120703	0.020009606	110460	Acat2
00630	Glyoxylate and dicarboxylate metabolism	6.317513831	0.014120703	0.020009606	76238	Grhpr
00630	Glyoxylate and dicarboxylate metabolism	6.317513831	0.014120703	0.020009606	17448	Mdh2
00630	Glyoxylate and dicarboxylate metabolism	6.317513831	0.014120703	0.020009606	224530	Acat3
04215	Apoptosis - multiple species	6.317513831	0.014120703	0.020009606	13063	Cycs
04215	Apoptosis - multiple species	6.317513831	0.014120703	0.020009606	12369	Casp7
04215	Apoptosis - multiple species	6.317513831	0.014120703	0.020009606	12122	Bid
04215	Apoptosis - multiple species	6.317513831	0.014120703	0.020009606	11796	Birc3
03013	RNA transport	2.776929157	0.014340703	0.020230635	67724	Pop1
03013	RNA transport	2.776929157	0.014340703	0.020230635	13684	Eif4e
03013	RNA transport	2.776929157	0.014340703	0.020230635	66235	Eif1ax
03013	RNA transport	2.776929157	0.014340703	0.020230635	114671	4930444G20Rik
03013	RNA transport	2.776929157	0.014340703	0.020230635	66441	Magohb
03013	RNA transport	2.776929157	0.014340703	0.020230635	231201	AF366264
03013	RNA transport	2.776929157	0.014340703	0.020230635	108067	Eif2b3
03013	RNA transport	2.776929157	0.014340703	0.020230635	13690	Eif4g2
03013	RNA transport	2.776929157	0.014340703	0.020230635	13664	Eif1a
03013	RNA transport	2.776929157	0.014340703	0.020230635	76884	Cyfip2
04971	Gastric acid secretion	4.043208852	0.014991684	0.021054987	12349	Car2
04971	Gastric acid secretion	4.043208852	0.014991684	0.021054987	228785	Mylk2
04971	Gastric acid secretion	4.043208852	0.014991684	0.021054987	15466	Hrh2
04971	Gastric acid secretion	4.043208852	0.014991684	0.021054987	108058	Camk2d
04971	Gastric acid secretion	4.043208852	0.014991684	0.021054987	22350	Ezr
04971	Gastric acid secretion	4.043208852	0.014991684	0.021054987	18798	Plcb4
04916	Melanogenesis	3.537807746	0.015216178	0.021181993	13618	Ednrb
04916	Melanogenesis	3.537807746	0.015216178	0.021181993	13614	Edn1

04916	Melanogenesis	3.537807746	0.015216178	0.021181993	22420	Wnt6
04916	Melanogenesis	3.537807746	0.015216178	0.021181993	18798	Plcb4
04916	Melanogenesis	3.537807746	0.015216178	0.021181993	14362	Fzd1
04916	Melanogenesis	3.537807746	0.015216178	0.021181993	108058	Camk2d
04916	Melanogenesis	3.537807746	0.015216178	0.021181993	50518	a
04976	Bile secretion	3.537807746	0.015216178	0.021181993	64008	Aqp9
04976	Bile secretion	3.537807746	0.015216178	0.021181993	12349	Car2
04976	Bile secretion	3.537807746	0.015216178	0.021181993	15357	Hmgcr
04976	Bile secretion	3.537807746	0.015216178	0.021181993	239273	Abcc4
04976	Bile secretion	3.537807746	0.015216178	0.021181993	18671	Abcb1a
04976	Bile secretion	3.537807746	0.015216178	0.021181993	16835	Ldlr
04976	Bile secretion	3.537807746	0.015216178	0.021181993	20525	Slc2a1
00030	Pentose phosphate pathway	6.126074018	0.015819622	0.02192544	66681	Pgm2
00030	Pentose phosphate pathway	6.126074018	0.015819622	0.02192544	14381	G6pdx
00030	Pentose phosphate pathway	6.126074018	0.015819622	0.02192544	18641	Pfkl
00030	Pentose phosphate pathway	6.126074018	0.015819622	0.02192544	110208	Pgd
04330	Notch signaling pathway	4.679639875	0.01708286	0.023572855	18128	Notch1
04330	Notch signaling pathway	4.679639875	0.01708286	0.023572855	16449	Jag1
04330	Notch signaling pathway	4.679639875	0.01708286	0.023572855	11491	Adam17
04330	Notch signaling pathway	4.679639875	0.01708286	0.023572855	209200	Dtx3l
04330	Notch signaling pathway	4.679639875	0.01708286	0.023572855	74198	Dtx2
00500	Starch and sucrose metabolism	5.945895371	0.017651541	0.024146697	14936	Gys1
00500	Starch and sucrose metabolism	5.945895371	0.017651541	0.024146697	74185	Gbe1
00500	Starch and sucrose metabolism	5.945895371	0.017651541	0.024146697	15277	Hk2
00500	Starch and sucrose metabolism	5.945895371	0.017651541	0.024146697	66681	Pgm2
00640	Propanoate metabolism	5.945895371	0.017651541	0.024146697	16828	Ldha
00640	Propanoate metabolism	5.945895371	0.017651541	0.024146697	12040	Bckdhb
00640	Propanoate metabolism	5.945895371	0.017651541	0.024146697	110460	Acat2
00640	Propanoate metabolism	5.945895371	0.017651541	0.024146697	224530	Acat3
04726	Serotonergic synapse	3.086418971	0.018788181	0.025590798	18783	Pla2g4a
04726	Serotonergic synapse	3.086418971	0.018788181	0.025590798	11684	Alox12
04726	Serotonergic synapse	3.086418971	0.018788181	0.025590798	15557	Htr1f
04726	Serotonergic synapse	3.086418971	0.018788181	0.025590798	19225	Ptgs2
04726	Serotonergic synapse	3.086418971	0.018788181	0.025590798	18798	Plcb4
04726	Serotonergic synapse	3.086418971	0.018788181	0.025590798	14701	Gng12
04726	Serotonergic synapse	3.086418971	0.018788181	0.025590798	15551	Htr1b
04726	Serotonergic synapse	3.086418971	0.018788181	0.025590798	15566	Htr7
04728	Dopaminergic synapse	2.99496952	0.02246831	0.030472043	19053	Ppp2cb
04728	Dopaminergic synapse	2.99496952	0.02246831	0.030472043	108058	Camk2d
04728	Dopaminergic synapse	2.99496952	0.02246831	0.030472043	19057	Ppp3cc
04728	Dopaminergic synapse	2.99496952	0.02246831	0.030472043	18798	Plcb4
04728	Dopaminergic synapse	2.99496952	0.02246831	0.030472043	231991	Creb5

04728	Dopaminergic synapse	2.99496952	0.02246831	0.030472043	11911	Atf4
04728	Dopaminergic synapse	2.99496952	0.02246831	0.030472043	26415	Mapk13
04728	Dopaminergic synapse	2.99496952	0.02246831	0.030472043	14701	Gng12
05216	Thyroid cancer	5.463795746	0.023990887	0.03239795	13197	Gadd45a
05216	Thyroid cancer	5.463795746	0.023990887	0.03239795	17873	Gadd45b
05216	Thyroid cancer	5.463795746	0.023990887	0.03239795	12575	Cdkn1a
05216	Thyroid cancer	5.463795746	0.023990887	0.03239795	17869	Myc
00770	Pantothenate and CoA biosynthesis	7.581016598	0.027410129	0.036701698	12035	Bcat1
00770	Pantothenate and CoA biosynthesis	7.581016598	0.027410129	0.036701698	64705	Dpys
00770	Pantothenate and CoA biosynthesis	7.581016598	0.027410129	0.036701698	26464	Vnn3
01210	2-Oxocarboxylic acid metabolism	7.581016598	0.027410129	0.036701698	217214	Nags
01210	2-Oxocarboxylic acid metabolism	7.581016598	0.027410129	0.036701698	109652	Acy1
01210	2-Oxocarboxylic acid metabolism	7.581016598	0.027410129	0.036701698	12035	Bcat1
00590	Arachidonic acid metabolism	3.526054231	0.029182117	0.038746004	18784	Pla2g5
00590	Arachidonic acid metabolism	3.526054231	0.029182117	0.038746004	18783	Pla2g4a
00590	Arachidonic acid metabolism	3.526054231	0.029182117	0.038746004	19225	Ptgs2
00590	Arachidonic acid metabolism	3.526054231	0.029182117	0.038746004	11684	Alox12
00590	Arachidonic acid metabolism	3.526054231	0.029182117	0.038746004	13850	Ephx2
00590	Arachidonic acid metabolism	3.526054231	0.029182117	0.038746004	64292	Ptges
04970	Salivary secretion	3.526054231	0.029182117	0.038746004	18798	Plcb4
04970	Salivary secretion	3.526054231	0.029182117	0.038746004	12182	Bst1
04970	Salivary secretion	3.526054231	0.029182117	0.038746004	12494	Cd38
04970	Salivary secretion	3.526054231	0.029182117	0.038746004	16531	Kcnma1
04970	Salivary secretion	3.526054231	0.029182117	0.038746004	12796	Camp
04970	Salivary secretion	3.526054231	0.029182117	0.038746004	381290	Atp2b4
04724	Glutamatergic synapse	3.130803315	0.029493593	0.038995713	18798	Plcb4
04724	Glutamatergic synapse	3.130803315	0.029493593	0.038995713	20510	Slc1a1
04724	Glutamatergic synapse	3.130803315	0.029493593	0.038995713	18783	Pla2g4a
04724	Glutamatergic synapse	3.130803315	0.029493593	0.038995713	14701	Gng12
04724	Glutamatergic synapse	3.130803315	0.029493593	0.038995713	14814	Grin2d
04724	Glutamatergic synapse	3.130803315	0.029493593	0.038995713	20511	Slc1a2
04724	Glutamatergic synapse	3.130803315	0.029493593	0.038995713	19057	Ppp3cc
04213	Longevity regulating pathway - multiple species	4.075815375	0.0307245	0.040453925	105787	Prkaa1
04213	Longevity regulating pathway - multiple species	4.075815375	0.0307245	0.040453925	108079	Prkaa2
04213	Longevity regulating pathway - multiple species	4.075815375	0.0307245	0.040453925	18708	Pik3r1
04213	Longevity regulating pathway - multiple species	4.075815375	0.0307245	0.040453925	20656	Sod2
04213	Longevity regulating pathway - multiple species	4.075815375	0.0307245	0.040453925	56717	Mtor
04913	Ovarian steroidogenesis	4.011119893	0.032840797	0.043060963	19225	Ptgs2
04913	Ovarian steroidogenesis	4.011119893	0.032840797	0.043060963	18783	Pla2g4a
04913	Ovarian steroidogenesis	4.011119893	0.032840797	0.043060963	16835	Ldlr
04913	Ovarian steroidogenesis	4.011119893	0.032840797	0.043060963	13078	Cyp1b1
04913	Ovarian steroidogenesis	4.011119893	0.032840797	0.043060963	15490	Hsd17b7

00860	Porphyrin and chlorophyll metabolism	4.813343871	0.037622727	0.049127197	233016	Blvrb
00860	Porphyrin and chlorophyll metabolism	4.813343871	0.037622727	0.049127197	15368	Hmox1
00860	Porphyrin and chlorophyll metabolism	4.813343871	0.037622727	0.049127197	11655	Alas1
00860	Porphyrin and chlorophyll metabolism	4.813343871	0.037622727	0.049127197	67417	Ears2
00740	Riboflavin metabolism	12.63502766	0.04048015	0.052640854	11433	Acp5
00740	Riboflavin metabolism	12.63502766	0.04048015	0.052640854	233016	Blvrb
04110	Cell cycle	2.87626646	0.045869332	0.059404545	13197	Gadd45a
04110	Cell cycle	2.87626646	0.045869332	0.059404545	12579	Cdkn2b
04110	Cell cycle	2.87626646	0.045869332	0.059404545	17873	Gadd45b
04110	Cell cycle	2.87626646	0.045869332	0.059404545	55948	Sfn
04110	Cell cycle	2.87626646	0.045869332	0.059404545	12575	Cdkn1a
04110	Cell cycle	2.87626646	0.045869332	0.059404545	12444	Ccnd2
04110	Cell cycle	2.87626646	0.045869332	0.059404545	17869	Myc
05031	Amphetamine addiction	3.662326859	0.047689505	0.06150973	19057	Ppp3cc
05031	Amphetamine addiction	3.662326859	0.047689505	0.06150973	108058	Camk2d
05031	Amphetamine addiction	3.662326859	0.047689505	0.06150973	11911	Atf4
05031	Amphetamine addiction	3.662326859	0.047689505	0.06150973	14814	Grin2d
05031	Amphetamine addiction	3.662326859	0.047689505	0.06150973	231991	Creb5
04070	Phosphatidylinositol signaling system	3.158756916	0.048809969	0.062698985	17772	Mtm1
04070	Phosphatidylinositol signaling system	3.158756916	0.048809969	0.062698985	18798	Plcb4
04070	Phosphatidylinositol signaling system	3.158756916	0.048809969	0.062698985	18720	Pip5k1a
04070	Phosphatidylinositol signaling system	3.158756916	0.048809969	0.062698985	380921	Dgkh
04070	Phosphatidylinositol signaling system	3.158756916	0.048809969	0.062698985	18708	Pik3r1
04070	Phosphatidylinositol signaling system	3.158756916	0.048809969	0.062698985	18719	Pip5k1b
00400	Phenylalanine, tyrosine and tryptophan biosynthesis	11.2311357	0.051367176	0.065451725	73988	4930438A08Rik
00400	Phenylalanine, tyrosine and tryptophan biosynthesis	11.2311357	0.051367176	0.065451725	14204	Il4i1
00750	Vitamin B6 metabolism	11.2311357	0.051367176	0.065451725	11761	Aox1
00750	Vitamin B6 metabolism	11.2311357	0.051367176	0.065451725	107272	Psat1
04927	Cortisol synthesis and secretion	3.509729906	0.056588477	0.071815096	231991	Creb5
04927	Cortisol synthesis and secretion	3.509729906	0.056588477	0.071815096	16835	Ldlr
04927	Cortisol synthesis and secretion	3.509729906	0.056588477	0.071815096	15370	Nr4a1
04927	Cortisol synthesis and secretion	3.509729906	0.056588477	0.071815096	18798	Plcb4
04927	Cortisol synthesis and secretion	3.509729906	0.056588477	0.071815096	11911	Atf4
00650	Butanoate metabolism	5.415011855	0.07008234	0.088249733	224530	Acat3
00650	Butanoate metabolism	5.415011855	0.07008234	0.088249733	110460	Acat2
00650	Butanoate metabolism	5.415011855	0.07008234	0.088249733	208715	Hmgcs1
04924	Renin secretion	3.32500728	0.070097098	0.088249733	13614	Edn1
04924	Renin secretion	3.32500728	0.070097098	0.088249733	16531	Kcnma1
04924	Renin secretion	3.32500728	0.070097098	0.088249733	229933	Clca2
04924	Renin secretion	3.32500728	0.070097098	0.088249733	18798	Plcb4
04924	Renin secretion	3.32500728	0.070097098	0.088249733	19057	Ppp3cc
00130	Ubiquinone and other terpenoid-quinone biosynthesis	9.189111027	0.076449284	0.095486062	18104	Nqo1

00130	Ubiquinone and other terpenoid-quinone biosynthesis	9.189111027	0.076449284	0.095486062	12850	Coq7
00920	Sulfur metabolism	9.189111027	0.076449284	0.095486062	59010	Sqor
00920	Sulfur metabolism	9.189111027	0.076449284	0.095486062	23972	Papss2
00410	beta-Alanine metabolism	4.89097845	0.092062164	0.114534031	228608	Smox
00410	beta-Alanine metabolism	4.89097845	0.092062164	0.114534031	64705	Dpys
00410	beta-Alanine metabolism	4.89097845	0.092062164	0.114534031	11670	Aldh3a1
03018	RNA degradation	3.00833992	0.103134586	0.127805997	13806	Eno1
03018	RNA degradation	3.00833992	0.103134586	0.127805997	12228	Btg3
03018	RNA degradation	3.00833992	0.103134586	0.127805997	210106	Tent4a
03018	RNA degradation	3.00833992	0.103134586	0.127805997	18641	Pfkl
03018	RNA degradation	3.00833992	0.103134586	0.127805997	433182	Eno1b
04714	Thermogenesis	2.197396115	0.108331888	0.133722174	433256	Acsl5
04714	Thermogenesis	2.197396115	0.108331888	0.133722174	56717	Mtor
04714	Thermogenesis	2.197396115	0.108331888	0.133722174	14081	Acsl1
04714	Thermogenesis	2.197396115	0.108331888	0.133722174	12856	Cox17
04714	Thermogenesis	2.197396115	0.108331888	0.133722174	20112	Rps6ka2
04714	Thermogenesis	2.197396115	0.108331888	0.133722174	108079	Prkaa2
04714	Thermogenesis	2.197396115	0.108331888	0.133722174	50790	Acsl4
04714	Thermogenesis	2.197396115	0.108331888	0.133722174	26415	Mapk13
04714	Thermogenesis	2.197396115	0.108331888	0.133722174	105787	Prkaa1
04714	Thermogenesis	2.197396115	0.108331888	0.133722174	231991	Creb5
00533	Glycosaminoglycan biosynthesis - keratan sulfate	7.220015807	0.121637396	0.149561934	20442	St3gal1
00533	Glycosaminoglycan biosynthesis - keratan sulfate	7.220015807	0.121637396	0.149561934	20441	St3gal3
04730	Long-term depression	3.36934071	0.124268122	0.152204367	17096	Lyn
04730	Long-term depression	3.36934071	0.124268122	0.152204367	19053	Ppp2cb
04730	Long-term depression	3.36934071	0.124268122	0.152204367	18783	Pla2g4a
04730	Long-term depression	3.36934071	0.124268122	0.152204367	18798	Plcb4
04727	GABAergic synapse	2.839332059	0.128103039	0.1562956	14411	Slc6a12
04727	GABAergic synapse	2.839332059	0.128103039	0.1562956	14701	Gng12
04727	GABAergic synapse	2.839332059	0.128103039	0.1562956	20779	Src
04727	GABAergic synapse	2.839332059	0.128103039	0.1562956	14412	Slc6a13
04727	GABAergic synapse	2.839332059	0.128103039	0.1562956	268566	Gphn
04961	Endocrine and other factor-regulated calcium reabsorption	3.314105616	0.130974314	0.159184167	22337	Vdr
04961	Endocrine and other factor-regulated calcium reabsorption	3.314105616	0.130974314	0.159184167	381290	Atp2b4
04961	Endocrine and other factor-regulated calcium reabsorption	3.314105616	0.130974314	0.159184167	18798	Plcb4
04961	Endocrine and other factor-regulated calcium reabsorption	3.314105616	0.130974314	0.159184167	12062	Bdkrb2
00730	Thiamine metabolism	6.73868142	0.138539334	0.167733447	11639	Ak4
00730	Thiamine metabolism	6.73868142	0.138539334	0.167733447	11637	Ak2
00140	Steroid hormone biosynthesis	2.746745144	0.144744841	0.174577747	105349	Akr1c18
00140	Steroid hormone biosynthesis	2.746745144	0.144744841	0.174577747	15483	Hsd11b1
00140	Steroid hormone biosynthesis	2.746745144	0.144744841	0.174577747	15490	Hsd17b7
00140	Steroid hormone biosynthesis	2.746745144	0.144744841	0.174577747	56348	Hsd17b12

00140	Steroid hormone biosynthesis	2.746745144	0.144744841	0.174577747	13078	Cyp1b1
00310	Lysine degradation	3.158756916	0.15234021	0.183039948	110460	Acat2
00310	Lysine degradation	3.158756916	0.15234021	0.183039948	72947	Phykpl
00310	Lysine degradation	3.158756916	0.15234021	0.183039948	224530	Acat3
00310	Lysine degradation	3.158756916	0.15234021	0.183039948	67956	Kmt5a
00603	Glycosphingolipid biosynthesis - globo and isoglobo series	6.317513831	0.156290662	0.187075186	227671	Gbgt1
00603	Glycosphingolipid biosynthesis - globo and isoglobo series	6.317513831	0.156290662	0.187075186	20442	St3gal1
00910	Nitrogen metabolism	5.945895371	0.174851199	0.208501807	71934	Car13
00910	Nitrogen metabolism	5.945895371	0.174851199	0.208501807	12349	Car2
00513	Various types of N-glycan biosynthesis	3.790508299	0.177726624	0.211133884	20441	St3gal3
00513	Various types of N-glycan biosynthesis	3.790508299	0.177726624	0.211133884	102580	Alg9
00513	Various types of N-glycan biosynthesis	3.790508299	0.177726624	0.211133884	269181	Mgat4a
04142	Lysosome	2.314814228	0.186171368	0.220337649	17113	M6pr
04142	Lysosome	2.314814228	0.186171368	0.220337649	11433	Acp5
04142	Lysosome	2.314814228	0.186171368	0.220337649	18173	Slc11a1
04142	Lysosome	2.314814228	0.186171368	0.220337649	13032	Ctsc
04142	Lysosome	2.314814228	0.186171368	0.220337649	53599	Cd164
04142	Lysosome	2.314814228	0.186171368	0.220337649	18174	Slc11a2
00514	Other types of O-glycan biosynthesis	3.526054231	0.212662269	0.250751033	80294	Pofut2
00514	Other types of O-glycan biosynthesis	3.526054231	0.212662269	0.250751033	14425	Galnt3
00514	Other types of O-glycan biosynthesis	3.526054231	0.212662269	0.250751033	20441	St3gal3
04973	Carbohydrate digestion and absorption	3.158756916	0.277549253	0.326042989	15277	Hk2
04973	Carbohydrate digestion and absorption	3.158756916	0.277549253	0.326042989	18798	Plcb4
04973	Carbohydrate digestion and absorption	3.158756916	0.277549253	0.326042989	18708	Pik3r1
00520	Amino sugar and nucleotide sugar metabolism	3.032406639	0.305720124	0.357805775	66681	Pgm2
00520	Amino sugar and nucleotide sugar metabolism	3.032406639	0.305720124	0.357805775	15277	Hk2
00520	Amino sugar and nucleotide sugar metabolism	3.032406639	0.305720124	0.357805775	14584	Gfpt2
00290	Valine, leucine and isoleucine biosynthesis	12.63502766	0.307313769	0.358343731	12035	Bcat1
00534	Glycosaminoglycan biosynthesis - heparan sulfate / heparin	4.211675888	0.324313067	0.376775475	14042	Ext1
00534	Glycosaminoglycan biosynthesis - heparan sulfate / heparin	4.211675888	0.324313067	0.376775475	217119	Xylt2
00592	alpha-Linolenic acid metabolism	4.043208852	0.348044581	0.402864789	18784	Pla2g5
00592	alpha-Linolenic acid metabolism	4.043208852	0.348044581	0.402864789	18783	Pla2g4a
04114	Oocyte meiosis	2.123534061	0.352349429	0.406359196	19057	Ppp3cc
04114	Oocyte meiosis	2.123534061	0.352349429	0.406359196	26415	Mapk13
04114	Oocyte meiosis	2.123534061	0.352349429	0.406359196	19053	Ppp2cb
04114	Oocyte meiosis	2.123534061	0.352349429	0.406359196	108058	Camk2d
04114	Oocyte meiosis	2.123534061	0.352349429	0.406359196	20112	Rps6ka2
00340	Histidine metabolism	3.887700819	0.372276294	0.427779305	11670	Aldh3a1
00340	Histidine metabolism	3.887700819	0.372276294	0.427779305	15186	Hdc
04260	Cardiac muscle contraction	2.323683248	0.37738729	0.4320811	326618	Tpm4
04260	Cardiac muscle contraction	2.323683248	0.37738729	0.4320811	81905	Cacng8
04260	Cardiac muscle contraction	2.323683248	0.37738729	0.4320811	54378	Cacng6

04260	Cardiac muscle contraction	2.323683248	0.37738729	0.4320811	15464	Hrc
00524	Neomycin, kanamycin and gentamicin biosynthesis	10.10802213	0.380382235	0.433937857	15277	Hk2
04744	Phototransduction	3.7437119	0.396979154	0.449625135	18587	Pde6b
04744	Phototransduction	3.7437119	0.396979154	0.449625135	19739	Rgs9
04966	Collecting duct acid secretion	3.7437119	0.396979154	0.449625135	11973	Atp6v1e1
04966	Collecting duct acid secretion	3.7437119	0.396979154	0.449625135	12349	Car2
04914	Progesterone-mediated oocyte maturation	2.24622714	0.414289355	0.467555129	26415	Mapk13
04914	Progesterone-mediated oocyte maturation	2.24622714	0.414289355	0.467555129	20112	Rps6ka2
04914	Progesterone-mediated oocyte maturation	2.24622714	0.414289355	0.467555129	18708	Pik3r1
04914	Progesterone-mediated oocyte maturation	2.24622714	0.414289355	0.467555129	15519	Hsp90aa1
00512	Mucin type O-glycan biosynthesis	3.610007904	0.422125063	0.474702918	14425	Galnt3
00512	Mucin type O-glycan biosynthesis	3.610007904	0.422125063	0.474702918	20442	St3gal1
05032	Morphine addiction	2.221543325	0.426943702	0.478419183	238871	Pde4d
05032	Morphine addiction	2.221543325	0.426943702	0.478419183	23984	Pde10a
05032	Morphine addiction	2.221543325	0.426943702	0.478419183	18578	Pde4b
05032	Morphine addiction	2.221543325	0.426943702	0.478419183	14701	Gng12
03060	Protein export	3.485524872	0.447686847	0.499890613	20335	Sec61g
03060	Protein export	3.485524872	0.447686847	0.499890613	14828	Hspa5
04710	Circadian rhythm	3.36934071	0.473638233	0.527005921	108079	Prkaa2
04710	Circadian rhythm	3.36934071	0.473638233	0.527005921	105787	Prkaa1
00830	Retinol metabolism	2.084128274	0.506412301	0.561495744	103142	Rdh9
00830	Retinol metabolism	2.084128274	0.506412301	0.561495744	77974	Rdh12
00830	Retinol metabolism	2.084128274	0.506412301	0.561495744	11529	Adh7
00830	Retinol metabolism	2.084128274	0.506412301	0.561495744	11761	Aox1
04713	Circadian entrainment	2.062861659	0.520223378	0.574792263	18798	Plcb4
04713	Circadian entrainment	2.062861659	0.520223378	0.574792263	14814	Grin2d
04713	Circadian entrainment	2.062861659	0.520223378	0.574792263	108058	Camk2d
04713	Circadian entrainment	2.062861659	0.520223378	0.574792263	14701	Gng12
05034	Alcoholism	1.725759876	0.523027761	0.575807845	14814	Grin2d
05034	Alcoholism	1.725759876	0.523027761	0.575807845	11540	Adora2a
05034	Alcoholism	1.725759876	0.523027761	0.575807845	55984	Camkk1
05034	Alcoholism	1.725759876	0.523027761	0.575807845	14701	Gng12
05034	Alcoholism	1.725759876	0.523027761	0.575807845	231991	Creb5
05034	Alcoholism	1.725759876	0.523027761	0.575807845	11541	Adora2b
05034	Alcoholism	1.725759876	0.523027761	0.575807845	11911	Atf4
00020	Citrate cycle (TCA cycle)	3.158756916	0.526609074	0.575807845	18563	Pcx
00020	Citrate cycle (TCA cycle)	3.158756916	0.526609074	0.575807845	17448	Mdh2
04136	Autophagy - other	3.158756916	0.526609074	0.575807845	19053	Ppp2cb
04136	Autophagy - other	3.158756916	0.526609074	0.575807845	56717	Mtor
04130	SNARE interactions in vesicular transport	3.063037009	0.553580264	0.603211598	74732	Stx11
04130	SNARE interactions in vesicular transport	3.063037009	0.553580264	0.603211598	58244	Stx6
01040	Biosynthesis of unsaturated fatty acids	2.972947685	0.580844481	0.63074521	56348	Hsd17b12

01040	Biosynthesis of unsaturated fatty acids	2.972947685	0.580844481	0.63074521	20250	Scd2
04960	Aldosterone-regulated sodium reabsorption	2.660005824	0.692400248	0.749309858	55948	Sfn
04960	Aldosterone-regulated sodium reabsorption	2.660005824	0.692400248	0.749309858	18708	Pik3r1
04962	Vasopressin-regulated water reabsorption	2.297277757	0.865093832	0.933002222	231991	Creb5
04962	Vasopressin-regulated water reabsorption	2.297277757	0.865093832	0.933002222	11828	Aqp3
04723	Retrograde endocannabinoid signaling	1.707436171	0.890710676	0.957362496	19225	Ptgs2
04723	Retrograde endocannabinoid signaling	1.707436171	0.890710676	0.957362496	26415	Mapk13
04723	Retrograde endocannabinoid signaling	1.707436171	0.890710676	0.957362496	14701	Gng12
04723	Retrograde endocannabinoid signaling	1.707436171	0.890710676	0.957362496	18798	Plcb4
04723	Retrograde endocannabinoid signaling	1.707436171	0.890710676	0.957362496	242864	Napepld
00604	Glycosphingolipid biosynthesis - ganglio series	3.36934071	1	1	20442	St3gal1
00510	N-Glycan biosynthesis	2.021604426	1	1	102580	Alg9
00510	N-Glycan biosynthesis	2.021604426	1	1	269181	Mgat4a
00591	Linoleic acid metabolism	2.021604426	1	1	18784	Pla2g5
00591	Linoleic acid metabolism	2.021604426	1	1	18783	Pla2g4a
03460	Fanconi anemia pathway	1.981965124	1	1	193838	Eme2
03460	Fanconi anemia pathway	1.981965124	1	1	223970	Rmi2
04340	Hedgehog signaling pathway	1.94385041	1	1	117606	Boc
04340	Hedgehog signaling pathway	1.94385041	1	1	12444	Ccnd2
00120	Primary bile acid biosynthesis	2.807783925	1	1	12642	Ch25h
04923	Regulation of lipolysis in adipocytes	1.805003952	1	1	19225	Ptgs2
04923	Regulation of lipolysis in adipocytes	1.805003952	1	1	18708	Pik3r1
04929	GnRH secretion	1.604447957	1	1	18708	Pik3r1
04929	GnRH secretion	1.604447957	1	1	18798	Plcb4
04964	Proximal tubule bicarbonate reclamation	2.297277757	1	1	12349	Car2
00515	Mannose type O-glycan biosynthesis	2.197396115	1	1	20441	St3gal3
04977	Vitamin digestion and absorption	2.105837944	1	1	17250	Abcc1
04742	Taste transduction	1.648047086	1	1	15551	Htr1b
04742	Taste transduction	1.648047086	1	1	18798	Plcb4
04742	Taste transduction	1.648047086	1	1	15557	Htr1f
00062	Fatty acid elongation	1.742762436	1	1	56348	Hsd17b12
03020	RNA polymerase	1.742762436	1	1	67065	Polr3d
03015	mRNA surveillance pathway	1.486473843	1	1	19053	Ppp2cb
03015	mRNA surveillance pathway	1.486473843	1	1	14852	Gspt1
03015	mRNA surveillance pathway	1.486473843	1	1	66441	Magohb
00040	Pentose and glucuronate interconversions	1.444003161	1	1	14187	Akr1b8
05340	Primary immunodeficiency	1.403891963	1	1	21939	Cd40
03040	Spliceosome	0.377165005	1	1	66441	Magohb
05033	Nicotine addiction	1.263502766	1	1	14814	Grin2d
03022	Basal transcription factors	1.148638878	1	1	68153	Gtf2e2
00190	Oxidative phosphorylation	1.140002496	1	1	11973	Atp6v1e1
00190	Oxidative phosphorylation	1.140002496	1	1	67895	Ppa1

00190	Oxidative phosphorylation	1.140002496	1	1	12856	Cox17
00970	Aminoacyl-tRNA biosynthesis	0.765759252	1	1	67417	Ears2

Down regulated gene significance pathway

path_id	path_name	path_diffgene_count	path_gene_count	enrichment	pvalue	FDR
1100	Metabolic pathways	133	1572	4.105618662	2.38385E-42	7.62832E-40
05200	Pathways in cancer	74	543	6.61319608	4.62129E-37	7.39407E-35
04010	MAPK signaling pathway	45	294	7.427534759	5.8609E-25	6.25163E-23
04020	Calcium signaling pathway	36	240	7.278984064	7.80084E-20	6.24068E-18
04014	Ras signaling pathway	33	232	6.902484888	1.55422E-17	9.94698E-16
04015	Rap1 signaling pathway	31	214	7.029548473	9.36186E-17	4.99299E-15
04068	FoxO signaling pathway	23	131	8.519930456	1.682E-14	7.27229E-13
05202	Transcriptional misregulation in cancer	29	223	6.310628934	1.81807E-14	7.27229E-13
05224	Breast cancer	24	147	7.922703743	2.35067E-14	8.35793E-13
05214	Glioma	18	74	11.80375794	3.97311E-14	1.27139E-12
04218	Cellular senescence	26	184	6.857013973	6.28893E-14	1.82951E-12
04151	PI3K-Akt signaling pathway	35	359	4.731001713	2.05622E-13	5.48325E-12
05210	Colorectal cancer	18	88	9.92588736	1.00746E-12	2.47989E-11
05225	Hepatocellular carcinoma	24	174	6.693318679	1.09493E-12	2.5027E-11
05168	Herpes simplex virus 1 infection	38	458	4.026221171	2.73846E-12	5.84206E-11
04659	Th17 cell differentiation	18	104	8.398827766	2.02734E-11	4.05469E-10
04072	Phospholipase D signaling pathway	21	149	6.839313885	2.16878E-11	4.08241E-10
05226	Gastric cancer	21	150	6.793718459	2.47311E-11	4.20023E-10
01521	EGFR tyrosine kinase inhibitor resistance	16	79	9.828164137	2.49389E-11	4.20023E-10
04371	Apelin signaling pathway	20	137	7.084169405	3.56334E-11	5.70134E-10
05218	Melanoma	15	72	10.10970009	7.38074E-11	1.07854E-09
04725	Cholinergic synapse	18	112	7.798911497	7.41499E-11	1.07854E-09
04926	Relaxin signaling pathway	19	129	7.147322853	1.00001E-10	1.39132E-09
04510	Focal adhesion	23	201	5.552790496	1.69895E-10	2.26527E-09
05205	Proteoglycans in cancer	23	205	5.444443365	2.53849E-10	3.24927E-09
05167	Kaposi sarcoma-associated herpesvirus infection	24	225	5.176166445	2.84873E-10	3.50613E-09
05166	Human T-cell leukemia virus 1 infection	25	247	4.911595185	3.58587E-10	4.23489E-09
04110	Cell cycle	18	123	7.101447867	3.70553E-10	4.23489E-09
04514	Cell adhesion molecules	21	174	5.856653844	4.31858E-10	4.76533E-09
04070	Phosphatidylinositol signaling system	16	96	8.087760071	5.51638E-10	5.75404E-09
04152	AMPK signaling pathway	18	126	6.932365775	5.57423E-10	5.75404E-09
04310	Wnt signaling pathway	20	162	5.990933386	7.91381E-10	7.91381E-09
04115	p53 signaling pathway	14	72	9.435720083	9.21235E-10	8.93318E-09
04810	Regulation of actin cytoskeleton	23	220	5.073231317	1.05195E-09	9.90072E-09
04935	Growth hormone synthesis, secretion and action	17	116	7.111651097	1.19651E-09	1.09395E-08
04658	Th1 and Th2 cell differentiation	15	88	8.2715728	1.49009E-09	1.32453E-08
04921	Oxytocin signaling pathway	19	153	6.02617417	2.01666E-09	1.74414E-08
04022	cGMP-PKG signaling pathway	20	173	5.610006986	2.57772E-09	2.17071E-08

04062	Chemokine signaling pathway	21	192	5.307592546	2.71631E-09	2.2062E-08
04144	Endocytosis	25	272	4.460161804	2.75775E-09	2.2062E-08
05222	Small cell lung cancer	15	93	7.826864585	3.3323E-09	2.56319E-08
04928	Parathyroid hormone synthesis, secretion and action	16	108	7.189120063	3.36419E-09	2.56319E-08
05206	MicroRNAs in cancer	26	303	4.163995284	5.52871E-09	4.11439E-08
04713	Circadian entrainment	15	98	7.427534759	7.0805E-09	5.14945E-08
04066	HIF-1 signaling pathway	16	114	6.810745323	7.5931E-09	5.39954E-08
04142	Lysosome	17	131	6.297339902	8.20308E-09	5.70649E-08
05223	Non-small cell lung cancer	13	72	8.761740077	1.05535E-08	7.18535E-08
04060	Cytokine-cytokine receptor interaction	25	293	4.140491504	1.27647E-08	8.50978E-08
04922	Glucagon signaling pathway	15	104	6.999023138	1.6477E-08	1.07605E-07
05163	Human cytomegalovirus infection	23	256	4.359808163	2.02089E-08	1.29337E-07
05170	Human immunodeficiency virus 1 infection	22	240	4.448268039	2.99303E-08	1.87798E-07
01522	Endocrine resistance	14	93	7.305073612	3.0594E-08	1.88271E-07
04360	Axon guidance	19	181	5.093948332	3.4411E-08	2.07764E-07
04640	Hematopoietic cell lineage	14	95	7.151282589	4.05797E-08	2.40472E-07
04672	Intestinal immune network for IgA production	10	43	11.28524661	5.54144E-08	3.22411E-07
05215	Prostate cancer	14	99	6.862341878	6.98916E-08	3.9938E-07
04728	Dopaminergic synapse	16	135	5.75129605	9.06629E-08	5.01021E-07
04933	AGE-RAGE signaling pathway in diabetic complications	14	101	6.72645392	9.08101E-08	5.01021E-07
04660	T cell receptor signaling pathway	14	103	6.595843165	1.17249E-07	6.35924E-07
04650	Natural killer cell mediated cytotoxicity	15	121	6.015689309	1.34604E-07	7.17887E-07
03320	PPAR signaling pathway	13	89	7.088149276	1.51415E-07	7.94307E-07
04211	Longevity regulating pathway	13	90	7.009392061	1.73645E-07	8.82005E-07
04914	Progesterone-mediated oocyte maturation	13	90	7.009392061	1.73645E-07	8.82005E-07
05212	Pancreatic cancer	12	76	7.662088488	2.06776E-07	1.00638E-06
05220	Chronic myeloid leukemia	12	76	7.662088488	2.06776E-07	1.00638E-06
04979	Cholesterol metabolism	10	49	9.903379679	2.12322E-07	1.00638E-06
05321	Inflammatory bowel disease	11	62	8.609551043	2.13209E-07	1.00638E-06
05165	Human papillomavirus infection	26	362	3.485333069	2.13856E-07	1.00638E-06
05145	Toxoplasmosis	14	110	6.17610769	2.73867E-07	1.27011E-06
03460	Fanconi anemia pathway	10	51	9.515011848	3.18033E-07	1.45387E-06
01524	Platinum drug resistance	12	80	7.278984064	3.72311E-07	1.67802E-06
05231	Choline metabolism in cancer	13	98	6.437196791	4.87723E-07	2.16766E-06
04915	Estrogen signaling pathway	15	134	5.43207766	5.31253E-07	2.32878E-06
04024	cAMP signaling pathway	19	215	4.288393712	5.50128E-07	2.37893E-06
04923	Regulation of lipolysis in adipocytes	10	56	8.665457219	8.07296E-07	3.44446E-06
04919	Thyroid hormone signaling pathway	14	120	5.66143205	8.246E-07	3.472E-06
04550	Signaling pathways regulating pluripotency of stem cells	15	140	5.199274331	9.47281E-07	3.93675E-06
00562	Inositol phosphate metabolism	11	72	7.413780065	1.05057E-06	4.31002E-06
05235	PD-L1 expression and PD-1 checkpoint pathway in cancer	12	88	6.61725824	1.09106E-06	4.41949E-06
03030	DNA replication	8	35	11.09178524	1.83242E-06	7.26607E-06

04380	Osteoclast differentiation	14	128	5.307592546	1.83922E-06	7.26607E-06
04213	Longevity regulating pathway - multiple species	10	62	7.826864585	2.17972E-06	8.50622E-06
04726	Serotonergic synapse	14	131	5.186044626	2.44499E-06	9.42647E-06
04929	GnRH secretion	10	63	7.702628639	2.54316E-06	9.68822E-06
04261	Adrenergic signaling in cardiomyocytes	15	152	4.788805305	2.75488E-06	1.03713E-05
04925	Aldosterone synthesis and secretion	12	102	5.709007109	5.51076E-06	2.05051E-05
05142	Chagas disease	12	103	5.653579855	6.12095E-06	2.25138E-05
04934	Cushing syndrome	15	162	4.493200039	6.19364E-06	2.25223E-05
05132	Salmonella infection	19	253	3.644287147	6.59684E-06	2.3719E-05
05161	Hepatitis B	15	163	4.465634395	6.69235E-06	2.3795E-05
05418	Fluid shear stress and atherosclerosis	14	146	4.653231822	9.04918E-06	3.18213E-05
04750	Inflammatory mediator regulation of TRP channels	13	127	4.967285713	9.95956E-06	3.4642E-05
04612	Antigen processing and presentation	11	90	5.931024052	1.03184E-05	3.55043E-05
04630	JAK-STAT signaling pathway	15	169	4.307091162	1.05166E-05	3.58012E-05
04723	Retrograde endocannabinoid signaling	14	148	4.59035031	1.06344E-05	3.58213E-05
04931	Insulin resistance	12	110	5.293806592	1.23394E-05	4.11314E-05
05414	Dilated cardiomyopathy	11	94	5.67864005	1.58877E-05	5.24129E-05
05412	Arrhythmogenic right ventricular cardiomyopathy	10	77	6.302150705	1.68224E-05	5.49302E-05
03430	Mismatch repair	6	22	13.23451648	1.70511E-05	5.51145E-05
05135	Yersinia infection	13	134	4.707800638	1.81347E-05	5.80309E-05
03410	Base excision repair	7	34	9.99076244	2.05791E-05	6.52012E-05
00230	Purine metabolism	13	136	4.638568276	2.13642E-05	6.63742E-05
04210	Apoptosis	13	136	4.638568276	2.13642E-05	6.63742E-05
00564	Glycerophospholipid metabolism	11	98	5.446858823	2.39238E-05	7.36116E-05
04662	B cell receptor signaling pathway	10	81	5.990933386	2.67421E-05	8.14997E-05
04910	Insulin signaling pathway	13	139	4.538455292	2.71619E-05	8.19982E-05
04114	Oocyte meiosis	12	119	4.893434665	2.81001E-05	8.40376E-05
05169	Epstein-Barr virus infection	17	231	3.571218733	2.98573E-05	8.8466E-05
05216	Thyroid cancer	7	37	9.180700621	3.7318E-05	0.000109557
04611	Platelet activation	12	124	4.696118751	4.2928E-05	0.000124882
05010	Alzheimer disease	22	369	2.893182464	4.48283E-05	0.000128961
04540	Gap junction	10	86	5.642623305	4.59423E-05	0.000128961
04911	Insulin secretion	10	86	5.642623305	4.59423E-05	0.000128961
04970	Salivary secretion	10	86	5.642623305	4.59423E-05	0.000128961
05230	Central carbon metabolism in cancer	9	69	6.32955136	4.88888E-05	0.000136038
05221	Acute myeloid leukemia	9	70	6.239129197	5.50795E-05	0.000151944
05146	Amoebiasis	11	107	4.988711819	5.59141E-05	0.000152927
04520	Adherens junction	9	71	6.151254138	6.19258E-05	0.000167934
04740	Olfactory transduction	6	1167	0.249493884	6.71828E-05	0.00018066
04912	GnRH signaling pathway	10	90	5.391840047	6.89167E-05	0.000183757
04927	Cortisol synthesis and secretion	9	72	6.065820053	6.94832E-05	0.000183757
05032	Morphine addiction	10	91	5.332589058	7.60028E-05	0.000197731

05410	Hypertrophic cardiomyopathy	10	91	5.332589058	7.60028E-05	0.000197731
04150	mTOR signaling pathway	13	156	4.043880035	9.4213E-05	0.00024313
00062	Fatty acid elongation	6	29	10.03997802	9.59555E-05	0.000245646
05213	Endometrial cancer	8	58	6.693318679	9.81345E-05	0.00024923
03420	Nucleotide excision repair	7	43	7.899672627	0.000104884	0.000264275
04710	Circadian rhythm	6	30	9.705312085	0.000117852	0.000294631
04730	Long-term depression	8	60	6.470208057	0.00012632	0.000313352
00920	Sulfur metabolism	4	11	17.64602197	0.000211184	0.000519839
05022	Pathways of neurodegeneration - multiple diseases	24	472	2.467452225	0.000240614	0.000587759
00600	Sphingolipid metabolism	7	50	6.793718459	0.000286818	0.000695316
05323	Rheumatoid arthritis	9	87	5.019989009	0.000317972	0.000765045
04512	ECM-receptor interaction	9	88	4.96294368	0.000347767	0.000830489
05152	Tuberculosis	13	180	3.504696031	0.00041322	0.000979484
00270	Cysteine and methionine metabolism	7	53	6.409168358	0.000419515	0.000987093
04390	Hippo signaling pathway	12	157	3.709036466	0.000441731	0.001031781
00330	Arginine and proline metabolism	7	54	6.290480055	0.000473463	0.001089988
04330	Notch signaling pathway	7	54	6.290480055	0.000473463	0.001089988
04724	Glutamatergic synapse	10	113	4.294385878	0.000488766	0.00111718
00980	Metabolism of xenobiotics by cytochrome P450	8	73	5.317979225	0.000524288	0.001189872
04350	TGF-beta signaling pathway	9	95	4.597253093	0.000629199	0.001417912
04971	Gastric acid secretion	8	75	5.176166445	0.00063464	0.001420174
04216	Ferroptosis	6	40	7.278984064	0.000639164	0.001420365
05020	Prion disease	16	268	2.897108085	0.000662254	0.001461527
04924	Renin secretion	8	76	5.108058992	0.00069659	0.001526774
03440	Homologous recombination	6	41	7.101447867	0.000735733	0.001601596
04530	Tight junction	12	167	3.486938474	0.000789331	0.001706663
04913	Ovarian steroidogenesis	7	63	5.391840047	0.001259828	0.002705672
05203	Viral carcinogenesis	14	229	2.966689284	0.001301578	0.002776699
04012	ErbB signaling pathway	8	84	4.621577183	0.00139383	0.002953813
00511	Other glycan degradation	4	18	10.78368009	0.001745102	0.003673898
04930	Type II diabetes mellitus	6	48	6.065820053	0.001776591	0.003715746
04727	GABAergic synapse	8	89	4.361938016	0.002063386	0.004287555
04625	C-type lectin receptor signaling pathway	9	112	3.899455748	0.002157556	0.00445431
02010	ABC transporters	6	50	5.823187251	0.002221625	0.00455718
05140	Leishmaniasis	7	70	4.852656042	0.002410204	0.004912517
01040	Biosynthesis of unsaturated fatty acids	5	34	7.136258886	0.002499628	0.005062538
04610	Complement and coagulation cascades	8	93	4.174327778	0.00276863	0.005572085
04061	Viral protein interaction with cytokine and cytokine receptor	8	95	4.086447194	0.003187663	0.006375326
00531	Glycosaminoglycan degradation	4	21	9.243154367	0.003249315	0.006458266
04917	Prolactin signaling pathway	7	74	4.59035031	0.003370206	0.006616356
04918	Thyroid hormone synthesis	7	74	4.59035031	0.003370206	0.006616356
04722	Neurotrophin signaling pathway	9	121	3.609413585	0.003766097	0.007348482

05162	Measles	10	146	3.323737015	0.003796419	0.007362752
04960	Aldosterone-regulated sodium reabsorption	5	38	6.38507374	0.004212741	0.008120947
04976	Bile secretion	8	100	3.882124834	0.004460748	0.008453418
04071	Sphingolipid signaling pathway	9	124	3.522089063	0.004479557	0.008453418
05150	Staphylococcus aureus infection	9	124	3.522089063	0.004479557	0.008453418
05144	Malaria	6	57	5.108058992	0.004490878	0.008453418
00515	Mannose type O-glycan biosynthesis	4	23	8.439401813	0.004652591	0.008706603
05204	Chemical carcinogenesis	8	101	3.843687954	0.00475826	0.008852578
04370	VEGF signaling pathway	6	58	5.019989009	0.004922697	0.009105567
04145	Phagosome	11	182	2.932923982	0.005993866	0.011023201
04064	NF-kappa B signaling pathway	8	105	3.697261747	0.00611007	0.0111727
05134	Legionellosis	6	61	4.773104304	0.006410111	0.011629829
05310	Asthma	4	25	7.764249668	0.006432749	0.011629829
01212	Fatty acid metabolism	6	62	4.696118751	0.00697477	0.012538912
04974	Protein digestion and absorption	8	108	3.594560031	0.007310513	0.013069073
05217	Basal cell carcinoma	6	63	4.621577183	0.007576342	0.013469053
04080	Neuroactive ligand-receptor interaction	17	358	2.304333875	0.007933037	0.014025258
04146	Peroxisome	7	86	3.949836314	0.008137529	0.014307743
04392	Hippo signaling pathway - multiple species	4	27	7.189120063	0.008637826	0.015104395
04260	Cardiac muscle contraction	7	87	3.904435896	0.008694701	0.015121218
05416	Viral myocarditis	7	88	3.860067307	0.009280855	0.01605337
04140	Autophagy - animal	9	138	3.16477568	0.009387513	0.016150559
04664	Fc epsilon RI signaling pathway	6	66	4.411505493	0.009617854	0.01645836
04972	Pancreatic secretion	8	114	3.405372661	0.010265231	0.017472733
05211	Renal cell carcinoma	6	68	4.281755332	0.011191782	0.01894905
04270	Vascular smooth muscle contraction	9	143	3.054119188	0.011927353	0.019996214
00983	Drug metabolism - other enzymes	7	92	3.692238293	0.01193524	0.019996214
04973	Carbohydrate digestion and absorption	5	48	5.054850044	0.012133422	0.020222369
04940	Type I diabetes mellitus	6	70	4.159419465	0.01295086	0.021472928
05164	Influenza A	10	173	2.805003493	0.013299049	0.021936575
00480	Glutathione metabolism	6	71	4.100836092	0.013903854	0.02258494
00982	Drug metabolism - cytochrome P450	6	71	4.100836092	0.013903854	0.02258494
04920	Adipocytokine signaling pathway	6	71	4.100836092	0.013903854	0.02258494
01200	Carbon metabolism	8	124	3.130745834	0.017177458	0.027761549
04978	Mineral absorption	5	53	4.577977399	0.018691373	0.030056479
04620	Toll-like receptor signaling pathway	7	100	3.39685923	0.0189456	0.030162149
04916	Melanogenesis	7	100	3.39685923	0.0189456	0.030162149
05100	Bacterial invasion of epithelial cells	6	76	3.831044244	0.019468523	0.030841225
00500	Starch and sucrose metabolism	4	34	5.709007109	0.0203688	0.031951059
00640	Propanoate metabolism	4	34	5.709007109	0.0203688	0.031951059
04721	Synaptic vesicle cycle	6	77	3.781290423	0.020752869	0.032237467
05133	Pertussis	6	77	3.781290423	0.020752869	0.032237467

00440	Phosphonate and phosphinate metabolism	2	6	16.17552014	0.024097547	0.037252247
05016	Huntington disease	14	302	2.249575649	0.024962407	0.038165798
00051	Fructose and mannose metabolism	4	36	5.391840047	0.025046305	0.038165798
05340	Primary immunodeficiency	4	36	5.391840047	0.025046305	0.038165798
00670	One carbon pool by folate	3	19	7.662088488	0.026450507	0.040114513
00240	Pyrimidine metabolism	5	58	4.183324175	0.027440123	0.041419053
00250	Alanine, aspartate and glutamate metabolism	4	39	4.977083121	0.033316044	0.049818384
00620	Pyruvate metabolism	4	39	4.977083121	0.033316044	0.049818384
04668	TNF signaling pathway	7	113	3.006070115	0.036399453	0.054090427
05017	Spinocerebellar ataxia	8	141	2.753280024	0.036511038	0.054090427
05330	Allograft rejection	5	63	3.851314319	0.038710376	0.056822571
05332	Graft-versus-host disease	5	63	3.851314319	0.038710376	0.056822571
00760	Nicotinate and nicotinamide metabolism	4	41	4.734298578	0.039716252	0.05803288
00310	Lysine degradation	5	64	3.791137533	0.041294616	0.060064896
04613	Neutrophil extracellular trap formation	10	205	2.367149289	0.042369991	0.06107386
05034	Alcoholism	10	205	2.367149289	0.042369991	0.06107386
04670	Leukocyte transendothelial migration	7	118	2.878694262	0.045537878	0.065345834
04137	Mitophagy - animal	5	66	3.676254578	0.046812228	0.066650449
00514	Other types of O-glycan biosynthesis	4	43	4.514098644	0.046863597	0.066650449
05322	Systemic lupus erythematosus	8	148	2.62305732	0.047994213	0.067956407
04666	Fc gamma R-mediated phagocytosis	6	92	3.16477568	0.048240918	0.068004818
00010	Glycolysis / Gluconeogenesis	5	67	3.621385106	0.049750111	0.069519806
04720	Long-term potentiation	5	67	3.621385106	0.049750111	0.069519806
04932	Non-alcoholic fatty liver disease	8	151	2.570943599	0.053651626	0.07464574
01240	Biosynthesis of cofactors	8	154	2.520860282	0.059781017	0.082813531
00650	Butanoate metabolism	3	28	5.199274331	0.07798322	0.107563062
05171	Coronavirus disease - COVID-19	11	247	2.161101881	0.080149258	0.110076234
00071	Fatty acid degradation	4	52	3.73281234	0.088979072	0.121162991
04340	Hedgehog signaling pathway	4	52	3.73281234	0.088979072	0.121162991
01230	Biosynthesis of amino acids	5	79	3.071301293	0.095105944	0.128413089
05320	Autoimmune thyroid disease	5	79	3.071301293	0.095105944	0.128413089
04714	Thermogenesis	10	230	2.109850453	0.120529652	0.162056674
04120	Ubiquitin mediated proteolysis	7	145	2.342661538	0.124853521	0.167167894
00040	Pentose and glucuronate interconversions	3	35	4.159419465	0.140201263	0.186935018
04961	Endocrine and other factor-regulated calcium reabsorption	4	61	3.182069536	0.148613747	0.197317822
00604	Glycosphingolipid biosynthesis - ganglio series	2	15	6.470208057	0.149221603	0.197317822
00561	Glycerolipid metabolism	4	62	3.130745834	0.156363701	0.205510775
05012	Parkinson disease	10	247	1.964638074	0.156701966	0.205510775
04742	Taste transduction	5	92	2.637313067	0.167492752	0.218766043
00450	Selenocompound metabolism	2	17	5.709007109	0.188134422	0.244727704
00350	Tyrosine metabolism	3	40	3.639492032	0.196384723	0.254425552
00830	Retinol metabolism	5	97	2.501369094	0.202233851	0.260946904

05219	Bladder cancer	3	41	3.550723934	0.208753286	0.267294777
00120	Primary bile acid biosynthesis	2	18	5.391840047	0.208824044	0.267294777
05031	Amphetamine addiction	4	69	2.813133938	0.216946011	0.276584555
04962	Vasopressin-regulated water reabsorption	3	44	3.30862912	0.248038524	0.31313022
05014	Amyotrophic lateral sclerosis	13	369	1.70960782	0.248293124	0.31313022
00100	Steroid biosynthesis	2	20	4.852656042	0.25246124	0.31313022
00220	Arginine biosynthesis	2	20	4.852656042	0.25246124	0.31313022
00532	Glycosaminoglycan biosynthesis - chondroitin sulfate / dermatan sulfate	2	20	4.852656042	0.25246124	0.31313022
00770	Pantothenate and CoA biosynthesis	2	20	4.852656042	0.25246124	0.31313022
01210	2-Oxocarboxylic acid metabolism	2	20	4.852656042	0.25246124	0.31313022
04964	Proximal tubule bicarbonate reclamation	2	22	4.411505493	0.298846917	0.369231713
00510	N-Glycan biosynthesis	3	50	2.911593625	0.335908396	0.413425718
04621	NOD-like receptor signaling pathway	8	211	1.83986959	0.338066346	0.414487474
00534	Glycosaminoglycan biosynthesis - heparan sulfate / heparin	2	24	4.043880035	0.347684581	0.424652923
00380	Tryptophan metabolism	3	52	2.799609255	0.367770625	0.447477566
00563	Glycosylphosphatidylinositol (GPI)-anchor biosynthesis	2	25	3.882124834	0.372935639	0.452043199
04141	Protein processing in endoplasmic reticulum	7	172	1.974918157	0.376897077	0.455120999
03018	RNA degradation	4	84	2.310788592	0.382938359	0.460677725
04217	Necroptosis	7	174	1.952217948	0.385548397	0.462080476
00590	Arachidonic acid metabolism	4	86	2.257049322	0.408572822	0.487848146
00601	Glycosphingolipid biosynthesis - lacto and neolacto series	2	27	3.594560031	0.424937897	0.505502331
00512	Mucin type O-glycan biosynthesis	2	28	3.466182887	0.451626718	0.534359799
00280	Valine, leucine and isoleucine degradation	3	57	2.554029496	0.452535955	0.534359799
00232	Caffeine metabolism	1	6	8.087760071	0.469799537	0.552705337
00020	Citrate cycle (TCA cycle)	2	32	3.032910027	0.56228798	0.651928093
00052	Galactose metabolism	2	32	3.032910027	0.56228798	0.651928093
00630	Glyoxylate and dicarboxylate metabolism	2	32	3.032910027	0.56228798	0.651928093
04215	Apoptosis - multiple species	2	32	3.032910027	0.56228798	0.651928093
00030	Pentose phosphate pathway	2	33	2.941003662	0.590794351	0.682506109
00740	Riboflavin metabolism	1	8	6.065820053	0.613808676	0.704009951
04122	Sulfur relay system	1	8	6.065820053	0.613808676	0.704009951
05160	Hepatitis C	6	165	1.764602197	0.636850781	0.727829464
00400	Phenylalanine, tyrosine and tryptophan biosynthesis	1	9	5.391840047	0.683596479	0.775712317
00750	Vitamin B6 metabolism	1	9	5.391840047	0.683596479	0.775712317
05143	African trypanosomiasis	2	39	2.48854156	0.767024108	0.867306411
00260	Glycine, serine and threonine metabolism	2	40	2.426328021	0.797057759	0.891812878
00513	Various types of N-glycan biosynthesis	2	40	2.426328021	0.797057759	0.891812878
05033	Nicotine addiction	2	40	2.426328021	0.797057759	0.891812878
00072	Synthesis and degradation of ketone bodies	1	12	4.043880035	0.884453469	0.98615021
00430	Taurine and hypotaurine metabolism	1	13	3.73281234	0.948666926	1
03450	Non-homologous end-joining	1	13	3.73281234	0.948666926	1
00565	Ether lipid metabolism	2	48	2.021940018	1	1

00730	Thiamine metabolism	1	15	3.235104028	1	1
03010	Ribosome	1	179	0.271098103	1	1
00910	Nitrogen metabolism	1	17	2.854503554	1	1
00061	Fatty acid biosynthesis	1	19	2.554029496	1	1
04623	Cytosolic DNA-sensing pathway	2	63	1.540525728	1	1
00360	Phenylalanine metabolism	1	23	2.109850453	1	1
00900	Terpenoid backbone biosynthesis	1	23	2.109850453	1	1
00970	Aminoacyl-tRNA biosynthesis	2	66	1.470501831	1	1
00592	alpha-Linolenic acid metabolism	1	25	1.941062417	1	1
00340	Histidine metabolism	1	26	1.86640617	1	1
04950	Maturity onset diabetes of the young	1	27	1.797280016	1	1
04966	Collecting duct acid secretion	1	27	1.797280016	1	1
04657	IL-17 signaling pathway	3	91	1.599776717	1	1
03020	RNA polymerase	1	29	1.67332967	1	1
01523	Antifolate resistance	1	30	1.617552014	1	1
00053	Ascorbate and aldarate metabolism	1	31	1.565372917	1	1
00410	beta-Alanine metabolism	1	31	1.565372917	1	1
04136	Autophagy - other	1	32	1.516455013	1	1
04130	SNARE interactions in vesicular transport	1	33	1.470501831	1	1
00190	Oxidative phosphorylation	1	133	0.364861357	1	1
00860	Porphyrin and chlorophyll metabolism	1	42	1.155394296	1	1
03013	RNA transport	2	182	0.533258906	1	1
03022	Basal transcription factors	1	44	1.102876373	1	1
03050	Proteasome	1	47	1.032480009	1	1
00140	Steroid hormone biosynthesis	2	92	1.054925227	1	1
03015	mRNA surveillance pathway	1	102	0.475750592	1	1
03040	Spliceosome	3	134	1.086415532	1	1
00520	Amino sugar and nucleotide sugar metabolism	1	50	0.970531208	1	1
04622	RIG-I-like receptor signaling pathway	1	70	0.693236577	1	1
05030	Cocaine addiction	1	48	1.010970009	1	1

Significance down regulated genes included in the pathway

path_id	path_name	enrichment	pvalue	FDR	gene_id	gene_name
01100	Metabolic pathways	4.105618662	2.38385E-42	7.62832E-40	171210	Acot2
01100	Metabolic pathways	4.105618662	2.38385E-42	7.62832E-40	26897	Acot1
01100	Metabolic pathways	4.105618662	2.38385E-42	7.62832E-40	20148	Dhrs3
01100	Metabolic pathways	4.105618662	2.38385E-42	7.62832E-40	241452	Dhrs9
01100	Metabolic pathways	4.105618662	2.38385E-42	7.62832E-40	11881	Arsb
01100	Metabolic pathways	4.105618662	2.38385E-42	7.62832E-40	15586	Hyal1
01100	Metabolic pathways	4.105618662	2.38385E-42	7.62832E-40	241062	Pgap1
01100	Metabolic pathways	4.105618662	2.38385E-42	7.62832E-40	27357	Gyg
01100	Metabolic pathways	4.105618662	2.38385E-42	7.62832E-40	110078	Pygb
01100	Metabolic pathways	4.105618662	2.38385E-42	7.62832E-40	14537	Gcnt1

01100	Metabolic pathways	4.105618662	2.38385E-42	7.62832E-40	14347	Fut7
01100	Metabolic pathways	4.105618662	2.38385E-42	7.62832E-40	11565	Adssl1
01100	Metabolic pathways	4.105618662	2.38385E-42	7.62832E-40	107885	Mthfs
01100	Metabolic pathways	4.105618662	2.38385E-42	7.62832E-40	211389	Suox
01100	Metabolic pathways	4.105618662	2.38385E-42	7.62832E-40	19125	Prodh
01100	Metabolic pathways	4.105618662	2.38385E-42	7.62832E-40	242341	Atp6v0d2
01100	Metabolic pathways	4.105618662	2.38385E-42	7.62832E-40	69726	Smyd3
01100	Metabolic pathways	4.105618662	2.38385E-42	7.62832E-40	73251	Setd7
01100	Metabolic pathways	4.105618662	2.38385E-42	7.62832E-40	232811	Kmt5c
01100	Metabolic pathways	4.105618662	2.38385E-42	7.62832E-40	192289	Tmlhe
01100	Metabolic pathways	4.105618662	2.38385E-42	7.62832E-40	20135	Rrm2
01100	Metabolic pathways	4.105618662	2.38385E-42	7.62832E-40	103140	Gstt3
01100	Metabolic pathways	4.105618662	2.38385E-42	7.62832E-40	54486	Hpgds
01100	Metabolic pathways	4.105618662	2.38385E-42	7.62832E-40	66447	Mgst3
01100	Metabolic pathways	4.105618662	2.38385E-42	7.62832E-40	76263	Gstk1
01100	Metabolic pathways	4.105618662	2.38385E-42	7.62832E-40	16332	Inppl1
01100	Metabolic pathways	4.105618662	2.38385E-42	7.62832E-40	101490	Inpp5f
01100	Metabolic pathways	4.105618662	2.38385E-42	7.62832E-40	20975	Synj2
01100	Metabolic pathways	4.105618662	2.38385E-42	7.62832E-40	269180	Inpp4a
01100	Metabolic pathways	4.105618662	2.38385E-42	7.62832E-40	18718	Pip4k2a
01100	Metabolic pathways	4.105618662	2.38385E-42	7.62832E-40	18796	Plcb2
01100	Metabolic pathways	4.105618662	2.38385E-42	7.62832E-40	18803	Plcg1
01100	Metabolic pathways	4.105618662	2.38385E-42	7.62832E-40	170835	Inpp5j
01100	Metabolic pathways	4.105618662	2.38385E-42	7.62832E-40	16329	Inpp1
01100	Metabolic pathways	4.105618662	2.38385E-42	7.62832E-40	114663	Impa2
01100	Metabolic pathways	4.105618662	2.38385E-42	7.62832E-40	320404	Itpkb
01100	Metabolic pathways	4.105618662	2.38385E-42	7.62832E-40	18125	Nos1
01100	Metabolic pathways	4.105618662	2.38385E-42	7.62832E-40	14718	Got1
01100	Metabolic pathways	4.105618662	2.38385E-42	7.62832E-40	16832	Ldhb
01100	Metabolic pathways	4.105618662	2.38385E-42	7.62832E-40	22236	Ugt1a2
01100	Metabolic pathways	4.105618662	2.38385E-42	7.62832E-40	18605	Enpp1
01100	Metabolic pathways	4.105618662	2.38385E-42	7.62832E-40	12495	Entpd1
01100	Metabolic pathways	4.105618662	2.38385E-42	7.62832E-40	18575	Pde1c
01100	Metabolic pathways	4.105618662	2.38385E-42	7.62832E-40	18576	Pde3b
01100	Metabolic pathways	4.105618662	2.38385E-42	7.62832E-40	104111	Adcy3
01100	Metabolic pathways	4.105618662	2.38385E-42	7.62832E-40	11513	Adcy7
01100	Metabolic pathways	4.105618662	2.38385E-42	7.62832E-40	66355	Gmpr
01100	Metabolic pathways	4.105618662	2.38385E-42	7.62832E-40	11486	Ada
01100	Metabolic pathways	4.105618662	2.38385E-42	7.62832E-40	20341	Selenbp1
01100	Metabolic pathways	4.105618662	2.38385E-42	7.62832E-40	20342	Selenbp2
01100	Metabolic pathways	4.105618662	2.38385E-42	7.62832E-40	22117	Tst
01100	Metabolic pathways	4.105618662	2.38385E-42	7.62832E-40	56078	Car5b

01100	Metabolic pathways	4.105618662	2.38385E-42	7.62832E-40	74637	Shpk
01100	Metabolic pathways	4.105618662	2.38385E-42	7.62832E-40	217666	L2hgdh
01100	Metabolic pathways	4.105618662	2.38385E-42	7.62832E-40	67041	Oxct1
01100	Metabolic pathways	4.105618662	2.38385E-42	7.62832E-40	68738	Acss1
01100	Metabolic pathways	4.105618662	2.38385E-42	7.62832E-40	18640	Pfkfb2
01100	Metabolic pathways	4.105618662	2.38385E-42	7.62832E-40	52538	Acaa2
01100	Metabolic pathways	4.105618662	2.38385E-42	7.62832E-40	100705	Acacb
01100	Metabolic pathways	4.105618662	2.38385E-42	7.62832E-40	16548	Khk
01100	Metabolic pathways	4.105618662	2.38385E-42	7.62832E-40	14345	Fut4
01100	Metabolic pathways	4.105618662	2.38385E-42	7.62832E-40	76051	Ganc
01100	Metabolic pathways	4.105618662	2.38385E-42	7.62832E-40	13026	Pcyt1a
01100	Metabolic pathways	4.105618662	2.38385E-42	7.62832E-40	236899	Pcyt1b
01100	Metabolic pathways	4.105618662	2.38385E-42	7.62832E-40	216395	Rxylt1
01100	Metabolic pathways	4.105618662	2.38385E-42	7.62832E-40	74653	Pomk
01100	Metabolic pathways	4.105618662	2.38385E-42	7.62832E-40	108902	B4gat1
01100	Metabolic pathways	4.105618662	2.38385E-42	7.62832E-40	13086	Cyp2a4
01100	Metabolic pathways	4.105618662	2.38385E-42	7.62832E-40	74134	Cyp2s1
01100	Metabolic pathways	4.105618662	2.38385E-42	7.62832E-40	68870	Ak8
01100	Metabolic pathways	4.105618662	2.38385E-42	7.62832E-40	269642	Nat8l
01100	Metabolic pathways	4.105618662	2.38385E-42	7.62832E-40	18642	Pfkm
01100	Metabolic pathways	4.105618662	2.38385E-42	7.62832E-40	233781	Xylt1
01100	Metabolic pathways	4.105618662	2.38385E-42	7.62832E-40	110197	Dgkg
01100	Metabolic pathways	4.105618662	2.38385E-42	7.62832E-40	13139	Dgka
01100	Metabolic pathways	4.105618662	2.38385E-42	7.62832E-40	102448	Xylb
01100	Metabolic pathways	4.105618662	2.38385E-42	7.62832E-40	12846	Comt
01100	Metabolic pathways	4.105618662	2.38385E-42	7.62832E-40	14431	Gamt
01100	Metabolic pathways	4.105618662	2.38385E-42	7.62832E-40	26384	Gnpda1
01100	Metabolic pathways	4.105618662	2.38385E-42	7.62832E-40	68636	Fahd1
01100	Metabolic pathways	4.105618662	2.38385E-42	7.62832E-40	104923	Adi1
01100	Metabolic pathways	4.105618662	2.38385E-42	7.62832E-40	13433	Dnmt1
01100	Metabolic pathways	4.105618662	2.38385E-42	7.62832E-40	13435	Dnmt3a
01100	Metabolic pathways	4.105618662	2.38385E-42	7.62832E-40	12709	Ckb
01100	Metabolic pathways	4.105618662	2.38385E-42	7.62832E-40	107766	Haao
01100	Metabolic pathways	4.105618662	2.38385E-42	7.62832E-40	66514	Asrgl1
01100	Metabolic pathways	4.105618662	2.38385E-42	7.62832E-40	20229	Sat1
01100	Metabolic pathways	4.105618662	2.38385E-42	7.62832E-40	71761	Amdhd1
01100	Metabolic pathways	4.105618662	2.38385E-42	7.62832E-40	104086	Cyp27a1
01100	Metabolic pathways	4.105618662	2.38385E-42	7.62832E-40	246277	Csad
01100	Metabolic pathways	4.105618662	2.38385E-42	7.62832E-40	218121	Mboat1
01100	Metabolic pathways	4.105618662	2.38385E-42	7.62832E-40	210992	Lpcat1
01100	Metabolic pathways	4.105618662	2.38385E-42	7.62832E-40	20598	Smpd2
01100	Metabolic pathways	4.105618662	2.38385E-42	7.62832E-40	58994	Smpd3

01100	Metabolic pathways	4.105618662	2.38385E-42	7.62832E-40	14420	Galc
01100	Metabolic pathways	4.105618662	2.38385E-42	7.62832E-40	67260	Cers4
01100	Metabolic pathways	4.105618662	2.38385E-42	7.62832E-40	11689	Alox5
01100	Metabolic pathways	4.105618662	2.38385E-42	7.62832E-40	19224	Ptgs1
01100	Metabolic pathways	4.105618662	2.38385E-42	7.62832E-40	12409	Cbr2
01100	Metabolic pathways	4.105618662	2.38385E-42	7.62832E-40	64384	Sirt3
01100	Metabolic pathways	4.105618662	2.38385E-42	7.62832E-40	68346	Sirt5
01100	Metabolic pathways	4.105618662	2.38385E-42	7.62832E-40	223646	Naprt
01100	Metabolic pathways	4.105618662	2.38385E-42	7.62832E-40	23945	Mgll
01100	Metabolic pathways	4.105618662	2.38385E-42	7.62832E-40	140481	Man2a2
01100	Metabolic pathways	4.105618662	2.38385E-42	7.62832E-40	20440	St6gal1
01100	Metabolic pathways	4.105618662	2.38385E-42	7.62832E-40	230145	Galnt12
01100	Metabolic pathways	4.105618662	2.38385E-42	7.62832E-40	230101	Gba2
01100	Metabolic pathways	4.105618662	2.38385E-42	7.62832E-40	20454	St3gal5
01100	Metabolic pathways	4.105618662	2.38385E-42	7.62832E-40	14421	B4galnt1
01100	Metabolic pathways	4.105618662	2.38385E-42	7.62832E-40	69574	Cmbl
01100	Metabolic pathways	4.105618662	2.38385E-42	7.62832E-40	75735	Pank1
01100	Metabolic pathways	4.105618662	2.38385E-42	7.62832E-40	81535	Sgpp1
01100	Metabolic pathways	4.105618662	2.38385E-42	7.62832E-40	226265	Eno4
01100	Metabolic pathways	4.105618662	2.38385E-42	7.62832E-40	74551	Pck2
01100	Metabolic pathways	4.105618662	2.38385E-42	7.62832E-40	331487	Uprt
01100	Metabolic pathways	4.105618662	2.38385E-42	7.62832E-40	110074	Dut
01100	Metabolic pathways	4.105618662	2.38385E-42	7.62832E-40	110639	Prps2
01100	Metabolic pathways	4.105618662	2.38385E-42	7.62832E-40	20322	Sord
01100	Metabolic pathways	4.105618662	2.38385E-42	7.62832E-40	15531	Ndst1
01100	Metabolic pathways	4.105618662	2.38385E-42	7.62832E-40	15442	Hpse
01100	Metabolic pathways	4.105618662	2.38385E-42	7.62832E-40	27029	Sgsh
01100	Metabolic pathways	4.105618662	2.38385E-42	7.62832E-40	15926	Idh1
01100	Metabolic pathways	4.105618662	2.38385E-42	7.62832E-40	216134	Pdxk
01100	Metabolic pathways	4.105618662	2.38385E-42	7.62832E-40	104759	Pld4
01100	Metabolic pathways	4.105618662	2.38385E-42	7.62832E-40	66775	Hacd4
01100	Metabolic pathways	4.105618662	2.38385E-42	7.62832E-40	68801	Elovl5
01100	Metabolic pathways	4.105618662	2.38385E-42	7.62832E-40	80911	Acox3
01100	Metabolic pathways	4.105618662	2.38385E-42	7.62832E-40	434437	Amt
01100	Metabolic pathways	4.105618662	2.38385E-42	7.62832E-40	230815	Man1c1
01100	Metabolic pathways	4.105618662	2.38385E-42	7.62832E-40	70266	Kyat1
01100	Metabolic pathways	4.105618662	2.38385E-42	7.62832E-40	22436	Xdh
01100	Metabolic pathways	4.105618662	2.38385E-42	7.62832E-40	13360	Dhcr7
01100	Metabolic pathways	4.105618662	2.38385E-42	7.62832E-40	15107	Hadh
05200	Pathways in cancer	6.61319608	4.62129E-37	7.39407E-35	12830	Col4a5
05200	Pathways in cancer	6.61319608	4.62129E-37	7.39407E-35	14268	Fn1
05200	Pathways in cancer	6.61319608	4.62129E-37	7.39407E-35	12428	Ccna2

05200	Pathways in cancer	6.61319608	4.62129E-37	7.39407E-35	22032	Traf4
05200	Pathways in cancer	6.61319608	4.62129E-37	7.39407E-35	22340	Vegfb
05200	Pathways in cancer	6.61319608	4.62129E-37	7.39407E-35	17311	Kitl
05200	Pathways in cancer	6.61319608	4.62129E-37	7.39407E-35	14256	Flt3l
05200	Pathways in cancer	6.61319608	4.62129E-37	7.39407E-35	12367	Casp3
05200	Pathways in cancer	6.61319608	4.62129E-37	7.39407E-35	23805	Apc2
05200	Pathways in cancer	6.61319608	4.62129E-37	7.39407E-35	56717	Mtor
05200	Pathways in cancer	6.61319608	4.62129E-37	7.39407E-35	54354	Rassf5
05200	Pathways in cancer	6.61319608	4.62129E-37	7.39407E-35	12443	Ccnd1
05200	Pathways in cancer	6.61319608	4.62129E-37	7.39407E-35	18803	Plcg1
05200	Pathways in cancer	6.61319608	4.62129E-37	7.39407E-35	14281	Fos
05200	Pathways in cancer	6.61319608	4.62129E-37	7.39407E-35	16842	Lef1
05200	Pathways in cancer	6.61319608	4.62129E-37	7.39407E-35	13819	Epas1
05200	Pathways in cancer	6.61319608	4.62129E-37	7.39407E-35	12190	Brca2
05200	Pathways in cancer	6.61319608	4.62129E-37	7.39407E-35	17685	Msh2
05200	Pathways in cancer	6.61319608	4.62129E-37	7.39407E-35	21812	Tgfbr1
05200	Pathways in cancer	6.61319608	4.62129E-37	7.39407E-35	21813	Tgfbr2
05200	Pathways in cancer	6.61319608	4.62129E-37	7.39407E-35	17127	Smad3
05200	Pathways in cancer	6.61319608	4.62129E-37	7.39407E-35	17686	Msh3
05200	Pathways in cancer	6.61319608	4.62129E-37	7.39407E-35	17688	Msh6
05200	Pathways in cancer	6.61319608	4.62129E-37	7.39407E-35	19016	Pparg
05200	Pathways in cancer	6.61319608	4.62129E-37	7.39407E-35	20181	Rxra
05200	Pathways in cancer	6.61319608	4.62129E-37	7.39407E-35	16480	Jup
05200	Pathways in cancer	6.61319608	4.62129E-37	7.39407E-35	12606	Cebpa
05200	Pathways in cancer	6.61319608	4.62129E-37	7.39407E-35	242705	E2f2
05200	Pathways in cancer	6.61319608	4.62129E-37	7.39407E-35	27401	Skp2
05200	Pathways in cancer	6.61319608	4.62129E-37	7.39407E-35	14673	Gna12
05200	Pathways in cancer	6.61319608	4.62129E-37	7.39407E-35	269608	Plekhg5
05200	Pathways in cancer	6.61319608	4.62129E-37	7.39407E-35	18796	Plcb2
05200	Pathways in cancer	6.61319608	4.62129E-37	7.39407E-35	19395	Rasgrp2
05200	Pathways in cancer	6.61319608	4.62129E-37	7.39407E-35	233046	Rasgrp4
05200	Pathways in cancer	6.61319608	4.62129E-37	7.39407E-35	381810	Lpar5
05200	Pathways in cancer	6.61319608	4.62129E-37	7.39407E-35	67168	Lpar6
05200	Pathways in cancer	6.61319608	4.62129E-37	7.39407E-35	19218	Ptger3
05200	Pathways in cancer	6.61319608	4.62129E-37	7.39407E-35	104111	Adcy3
05200	Pathways in cancer	6.61319608	4.62129E-37	7.39407E-35	11513	Adcy7
05200	Pathways in cancer	6.61319608	4.62129E-37	7.39407E-35	14697	Gnb5
05200	Pathways in cancer	6.61319608	4.62129E-37	7.39407E-35	14702	Gng2
05200	Pathways in cancer	6.61319608	4.62129E-37	7.39407E-35	66066	Gng11
05200	Pathways in cancer	6.61319608	4.62129E-37	7.39407E-35	27015	Polk
05200	Pathways in cancer	6.61319608	4.62129E-37	7.39407E-35	17977	Ncoa1
05200	Pathways in cancer	6.61319608	4.62129E-37	7.39407E-35	17979	Ncoa3

05200	Pathways in cancer	6.61319608	4.62129E-37	7.39407E-35	16973	Lrp5
05200	Pathways in cancer	6.61319608	4.62129E-37	7.39407E-35	14296	Frat1
05200	Pathways in cancer	6.61319608	4.62129E-37	7.39407E-35	240168	Rasgrp3
05200	Pathways in cancer	6.61319608	4.62129E-37	7.39407E-35	12767	Cxcr4
05200	Pathways in cancer	6.61319608	4.62129E-37	7.39407E-35	16576	Kif7
05200	Pathways in cancer	6.61319608	4.62129E-37	7.39407E-35	15213	Hey1
05200	Pathways in cancer	6.61319608	4.62129E-37	7.39407E-35	103140	Gstt3
05200	Pathways in cancer	6.61319608	4.62129E-37	7.39407E-35	66447	Mgst3
05200	Pathways in cancer	6.61319608	4.62129E-37	7.39407E-35	23882	Gadd45g
05200	Pathways in cancer	6.61319608	4.62129E-37	7.39407E-35	107986	Ddb2
05200	Pathways in cancer	6.61319608	4.62129E-37	7.39407E-35	18710	Pik3r3
05200	Pathways in cancer	6.61319608	4.62129E-37	7.39407E-35	18596	Pdgfrb
05200	Pathways in cancer	6.61319608	4.62129E-37	7.39407E-35	16001	Igf1r
05200	Pathways in cancer	6.61319608	4.62129E-37	7.39407E-35	14182	Fgfr1
05200	Pathways in cancer	6.61319608	4.62129E-37	7.39407E-35	15234	Hgf
05200	Pathways in cancer	6.61319608	4.62129E-37	7.39407E-35	212398	Frat2
05200	Pathways in cancer	6.61319608	4.62129E-37	7.39407E-35	73086	Rps6ka5
05200	Pathways in cancer	6.61319608	4.62129E-37	7.39407E-35	12322	Camk2a
05200	Pathways in cancer	6.61319608	4.62129E-37	7.39407E-35	170770	Bbc3
05200	Pathways in cancer	6.61319608	4.62129E-37	7.39407E-35	15979	Ifngr1
05200	Pathways in cancer	6.61319608	4.62129E-37	7.39407E-35	16162	Il12rb2
05200	Pathways in cancer	6.61319608	4.62129E-37	7.39407E-35	16194	Il6ra
05200	Pathways in cancer	6.61319608	4.62129E-37	7.39407E-35	16195	Il6st
05200	Pathways in cancer	6.61319608	4.62129E-37	7.39407E-35	18709	Pik3r2
05200	Pathways in cancer	6.61319608	4.62129E-37	7.39407E-35	18590	Pdgfa
05200	Pathways in cancer	6.61319608	4.62129E-37	7.39407E-35	18591	Pdgfb
05200	Pathways in cancer	6.61319608	4.62129E-37	7.39407E-35	16000	Igf1
05200	Pathways in cancer	6.61319608	4.62129E-37	7.39407E-35	16774	Lama3
05200	Pathways in cancer	6.61319608	4.62129E-37	7.39407E-35	16403	Itga6
04010	MAPK signaling pathway	7.427534759	5.8609E-25	6.25163E-23	18018	Nfatc1
04010	MAPK signaling pathway	7.427534759	5.8609E-25	6.25163E-23	26401	Map3k1
04010	MAPK signaling pathway	7.427534759	5.8609E-25	6.25163E-23	11601	Angpt2
04010	MAPK signaling pathway	7.427534759	5.8609E-25	6.25163E-23	17311	Kitl
04010	MAPK signaling pathway	7.427534759	5.8609E-25	6.25163E-23	26411	Map4k1
04010	MAPK signaling pathway	7.427534759	5.8609E-25	6.25163E-23	17347	Mknk2
04010	MAPK signaling pathway	7.427534759	5.8609E-25	6.25163E-23	16000	Igf1
04010	MAPK signaling pathway	7.427534759	5.8609E-25	6.25163E-23	14281	Fos
04010	MAPK signaling pathway	7.427534759	5.8609E-25	6.25163E-23	193740	Hspa1a
04010	MAPK signaling pathway	7.427534759	5.8609E-25	6.25163E-23	73086	Rps6ka5
04010	MAPK signaling pathway	7.427534759	5.8609E-25	6.25163E-23	17260	Mef2c
04010	MAPK signaling pathway	7.427534759	5.8609E-25	6.25163E-23	26399	Map2k6
04010	MAPK signaling pathway	7.427534759	5.8609E-25	6.25163E-23	26407	Map3k4

04010	MAPK signaling pathway	7.427534759	5.8609E-25	6.25163E-23	14256	Flt3l
04010	MAPK signaling pathway	7.427534759	5.8609E-25	6.25163E-23	15234	Hgf
04010	MAPK signaling pathway	7.427534759	5.8609E-25	6.25163E-23	18596	Pdgfrb
04010	MAPK signaling pathway	7.427534759	5.8609E-25	6.25163E-23	12286	Cacna1a
04010	MAPK signaling pathway	7.427534759	5.8609E-25	6.25163E-23	12287	Cacna1b
04010	MAPK signaling pathway	7.427534759	5.8609E-25	6.25163E-23	12289	Cacna1d
04010	MAPK signaling pathway	7.427534759	5.8609E-25	6.25163E-23	12298	Cacnb4
04010	MAPK signaling pathway	7.427534759	5.8609E-25	6.25163E-23	319734	Cacna2d4
04010	MAPK signaling pathway	7.427534759	5.8609E-25	6.25163E-23	12531	Cdc25b
04010	MAPK signaling pathway	7.427534759	5.8609E-25	6.25163E-23	19395	Rasgrp2
04010	MAPK signaling pathway	7.427534759	5.8609E-25	6.25163E-23	233046	Rasgrp4
04010	MAPK signaling pathway	7.427534759	5.8609E-25	6.25163E-23	240168	Rasgrp3
04010	MAPK signaling pathway	7.427534759	5.8609E-25	6.25163E-23	14673	Gna12
04010	MAPK signaling pathway	7.427534759	5.8609E-25	6.25163E-23	26404	Map3k12
04010	MAPK signaling pathway	7.427534759	5.8609E-25	6.25163E-23	71751	Map3k13
04010	MAPK signaling pathway	7.427534759	5.8609E-25	6.25163E-23	23882	Gadd45g
04010	MAPK signaling pathway	7.427534759	5.8609E-25	6.25163E-23	12367	Casp3
04010	MAPK signaling pathway	7.427534759	5.8609E-25	6.25163E-23	21812	Tgfbr1
04010	MAPK signaling pathway	7.427534759	5.8609E-25	6.25163E-23	21813	Tgfbr2
04010	MAPK signaling pathway	7.427534759	5.8609E-25	6.25163E-23	19099	Mapk8ip1
04010	MAPK signaling pathway	7.427534759	5.8609E-25	6.25163E-23	109689	Arrb1
04010	MAPK signaling pathway	7.427534759	5.8609E-25	6.25163E-23	15511	Hspa1b
04010	MAPK signaling pathway	7.427534759	5.8609E-25	6.25163E-23	16765	Stmn1
04010	MAPK signaling pathway	7.427534759	5.8609E-25	6.25163E-23	16001	Igf1r
04010	MAPK signaling pathway	7.427534759	5.8609E-25	6.25163E-23	16542	Kdr
04010	MAPK signaling pathway	7.427534759	5.8609E-25	6.25163E-23	22340	Vegfb
04010	MAPK signaling pathway	7.427534759	5.8609E-25	6.25163E-23	14182	Fgfr1
04010	MAPK signaling pathway	7.427534759	5.8609E-25	6.25163E-23	18590	Pdgfa
04010	MAPK signaling pathway	7.427534759	5.8609E-25	6.25163E-23	18591	Pdgfb
04010	MAPK signaling pathway	7.427534759	5.8609E-25	6.25163E-23	54635	Pdgfc
04010	MAPK signaling pathway	7.427534759	5.8609E-25	6.25163E-23	75590	Dusp9
04010	MAPK signaling pathway	7.427534759	5.8609E-25	6.25163E-23	235584	Dusp7
04020	Calcium signaling pathway	7.278984064	7.80084E-20	6.24068E-18	11549	Adra1a
04020	Calcium signaling pathway	7.278984064	7.80084E-20	6.24068E-18	12287	Cacna1b
04020	Calcium signaling pathway	7.278984064	7.80084E-20	6.24068E-18	104111	Adcy3
04020	Calcium signaling pathway	7.278984064	7.80084E-20	6.24068E-18	22340	Vegfb
04020	Calcium signaling pathway	7.278984064	7.80084E-20	6.24068E-18	12286	Cacna1a
04020	Calcium signaling pathway	7.278984064	7.80084E-20	6.24068E-18	252972	Tpcn1
04020	Calcium signaling pathway	7.278984064	7.80084E-20	6.24068E-18	18575	Pde1c
04020	Calcium signaling pathway	7.278984064	7.80084E-20	6.24068E-18	54635	Pdgfc
04020	Calcium signaling pathway	7.278984064	7.80084E-20	6.24068E-18	15465	Hrh1
04020	Calcium signaling pathway	7.278984064	7.80084E-20	6.24068E-18	320404	Itpkb

04020	Calcium signaling pathway	7.278984064	7.80084E-20	6.24068E-18	14182	Fgfr1
04020	Calcium signaling pathway	7.278984064	7.80084E-20	6.24068E-18	12767	Cxcr4
04020	Calcium signaling pathway	7.278984064	7.80084E-20	6.24068E-18	16438	Itpr1
04020	Calcium signaling pathway	7.278984064	7.80084E-20	6.24068E-18	16440	Itpr3
04020	Calcium signaling pathway	7.278984064	7.80084E-20	6.24068E-18	18440	P2rx6
04020	Calcium signaling pathway	7.278984064	7.80084E-20	6.24068E-18	18803	Plcg1
04020	Calcium signaling pathway	7.278984064	7.80084E-20	6.24068E-18	18591	Pdgfb
04020	Calcium signaling pathway	7.278984064	7.80084E-20	6.24068E-18	52163	Camk1
04020	Calcium signaling pathway	7.278984064	7.80084E-20	6.24068E-18	15559	Htr2b
04020	Calcium signaling pathway	7.278984064	7.80084E-20	6.24068E-18	19218	Ptger3
04020	Calcium signaling pathway	7.278984064	7.80084E-20	6.24068E-18	16542	Kdr
04020	Calcium signaling pathway	7.278984064	7.80084E-20	6.24068E-18	18596	Pdgfrb
04020	Calcium signaling pathway	7.278984064	7.80084E-20	6.24068E-18	67972	Atp2b1
04020	Calcium signaling pathway	7.278984064	7.80084E-20	6.24068E-18	110094	Phka2
04020	Calcium signaling pathway	7.278984064	7.80084E-20	6.24068E-18	11513	Adcy7
04020	Calcium signaling pathway	7.278984064	7.80084E-20	6.24068E-18	11555	Adrb2
04020	Calcium signaling pathway	7.278984064	7.80084E-20	6.24068E-18	12322	Camk2a
04020	Calcium signaling pathway	7.278984064	7.80084E-20	6.24068E-18	14676	Gna15
04020	Calcium signaling pathway	7.278984064	7.80084E-20	6.24068E-18	18796	Plcb2
04020	Calcium signaling pathway	7.278984064	7.80084E-20	6.24068E-18	12289	Cacna1d
04020	Calcium signaling pathway	7.278984064	7.80084E-20	6.24068E-18	18590	Pdgfa
04020	Calcium signaling pathway	7.278984064	7.80084E-20	6.24068E-18	18125	Nos1
04020	Calcium signaling pathway	7.278984064	7.80084E-20	6.24068E-18	18439	P2rx7
04020	Calcium signaling pathway	7.278984064	7.80084E-20	6.24068E-18	15234	Hgf
04020	Calcium signaling pathway	7.278984064	7.80084E-20	6.24068E-18	20541	Slc8a1
04020	Calcium signaling pathway	7.278984064	7.80084E-20	6.24068E-18	65973	Asph
04014	Ras signaling pathway	6.902484888	1.55422E-17	9.94698E-16	19415	Rasal1
04014	Ras signaling pathway	6.902484888	1.55422E-17	9.94698E-16	17311	Kitl
04014	Ras signaling pathway	6.902484888	1.55422E-17	9.94698E-16	18590	Pdgfa
04014	Ras signaling pathway	6.902484888	1.55422E-17	9.94698E-16	226525	Rasal2
04014	Ras signaling pathway	6.902484888	1.55422E-17	9.94698E-16	16000	Igf1
04014	Ras signaling pathway	6.902484888	1.55422E-17	9.94698E-16	54354	Rassf5
04014	Ras signaling pathway	6.902484888	1.55422E-17	9.94698E-16	217944	Rapgef5
04014	Ras signaling pathway	6.902484888	1.55422E-17	9.94698E-16	240168	Rasgrp3
04014	Ras signaling pathway	6.902484888	1.55422E-17	9.94698E-16	15234	Hgf
04014	Ras signaling pathway	6.902484888	1.55422E-17	9.94698E-16	18803	Plcg1
04014	Ras signaling pathway	6.902484888	1.55422E-17	9.94698E-16	333050	Ksr2
04014	Ras signaling pathway	6.902484888	1.55422E-17	9.94698E-16	19414	Rasa3
04014	Ras signaling pathway	6.902484888	1.55422E-17	9.94698E-16	18591	Pdgfb
04014	Ras signaling pathway	6.902484888	1.55422E-17	9.94698E-16	320484	Rasal3
04014	Ras signaling pathway	6.902484888	1.55422E-17	9.94698E-16	54635	Pdgfc
04014	Ras signaling pathway	6.902484888	1.55422E-17	9.94698E-16	18709	Pik3r2

04014	Ras signaling pathway	6.902484888	1.55422E-17	9.94698E-16	18710	Pik3r3
04014	Ras signaling pathway	6.902484888	1.55422E-17	9.94698E-16	216148	Shc2
04014	Ras signaling pathway	6.902484888	1.55422E-17	9.94698E-16	271849	Shc4
04014	Ras signaling pathway	6.902484888	1.55422E-17	9.94698E-16	18596	Pdgfrb
04014	Ras signaling pathway	6.902484888	1.55422E-17	9.94698E-16	233046	Rasgrp4
04014	Ras signaling pathway	6.902484888	1.55422E-17	9.94698E-16	19395	Rasgrp2
04014	Ras signaling pathway	6.902484888	1.55422E-17	9.94698E-16	22340	Vegfb
04014	Ras signaling pathway	6.902484888	1.55422E-17	9.94698E-16	14702	Gng2
04014	Ras signaling pathway	6.902484888	1.55422E-17	9.94698E-16	14182	Fgfr1
04014	Ras signaling pathway	6.902484888	1.55422E-17	9.94698E-16	16001	Igf1r
04014	Ras signaling pathway	6.902484888	1.55422E-17	9.94698E-16	16542	Kdr
04014	Ras signaling pathway	6.902484888	1.55422E-17	9.94698E-16	14697	Gnb5
04014	Ras signaling pathway	6.902484888	1.55422E-17	9.94698E-16	11601	Angpt2
04014	Ras signaling pathway	6.902484888	1.55422E-17	9.94698E-16	14256	Flt3l
04014	Ras signaling pathway	6.902484888	1.55422E-17	9.94698E-16	66066	Gng11
04014	Ras signaling pathway	6.902484888	1.55422E-17	9.94698E-16	21844	Tiam1
04014	Ras signaling pathway	6.902484888	1.55422E-17	9.94698E-16	225870	Rin1
04015	Rap1 signaling pathway	7.029548473	9.36186E-17	4.99299E-15	51791	Rgs14
04015	Rap1 signaling pathway	7.029548473	9.36186E-17	4.99299E-15	11601	Angpt2
04015	Rap1 signaling pathway	7.029548473	9.36186E-17	4.99299E-15	18803	Plcg1
04015	Rap1 signaling pathway	7.029548473	9.36186E-17	4.99299E-15	26399	Map2k6
04015	Rap1 signaling pathway	7.029548473	9.36186E-17	4.99299E-15	15234	Hgf
04015	Rap1 signaling pathway	7.029548473	9.36186E-17	4.99299E-15	244668	Sipa1l2
04015	Rap1 signaling pathway	7.029548473	9.36186E-17	4.99299E-15	18591	Pdgfb
04015	Rap1 signaling pathway	7.029548473	9.36186E-17	4.99299E-15	17311	Kitl
04015	Rap1 signaling pathway	7.029548473	9.36186E-17	4.99299E-15	223864	Rapgef3
04015	Rap1 signaling pathway	7.029548473	9.36186E-17	4.99299E-15	18441	P2ry1
04015	Rap1 signaling pathway	7.029548473	9.36186E-17	4.99299E-15	18709	Pik3r2
04015	Rap1 signaling pathway	7.029548473	9.36186E-17	4.99299E-15	14182	Fgfr1
04015	Rap1 signaling pathway	7.029548473	9.36186E-17	4.99299E-15	18590	Pdgfa
04015	Rap1 signaling pathway	7.029548473	9.36186E-17	4.99299E-15	22340	Vegfb
04015	Rap1 signaling pathway	7.029548473	9.36186E-17	4.99299E-15	16000	Igf1
04015	Rap1 signaling pathway	7.029548473	9.36186E-17	4.99299E-15	54354	Rassf5
04015	Rap1 signaling pathway	7.029548473	9.36186E-17	4.99299E-15	18796	Plcb2
04015	Rap1 signaling pathway	7.029548473	9.36186E-17	4.99299E-15	22325	Vav2
04015	Rap1 signaling pathway	7.029548473	9.36186E-17	4.99299E-15	54635	Pdgfc
04015	Rap1 signaling pathway	7.029548473	9.36186E-17	4.99299E-15	381810	Lpar5
04015	Rap1 signaling pathway	7.029548473	9.36186E-17	4.99299E-15	217944	Rapgef5
04015	Rap1 signaling pathway	7.029548473	9.36186E-17	4.99299E-15	16001	Igf1r
04015	Rap1 signaling pathway	7.029548473	9.36186E-17	4.99299E-15	21844	Tiam1
04015	Rap1 signaling pathway	7.029548473	9.36186E-17	4.99299E-15	106952	Arap3
04015	Rap1 signaling pathway	7.029548473	9.36186E-17	4.99299E-15	11513	Adcy7

04015	Rap1 signaling pathway	7.029548473	9.36186E-17	4.99299E-15	104111	Adcy3
04015	Rap1 signaling pathway	7.029548473	9.36186E-17	4.99299E-15	240168	Rasgrp3
04015	Rap1 signaling pathway	7.029548473	9.36186E-17	4.99299E-15	18710	Pik3r3
04015	Rap1 signaling pathway	7.029548473	9.36186E-17	4.99299E-15	18596	Pdgfrb
04015	Rap1 signaling pathway	7.029548473	9.36186E-17	4.99299E-15	16542	Kdr
04015	Rap1 signaling pathway	7.029548473	9.36186E-17	4.99299E-15	19395	Rasgrp2
04068	FoxO signaling pathway	8.519930456	1.682E-14	7.27229E-13	12443	Ccnd1
04068	FoxO signaling pathway	8.519930456	1.682E-14	7.27229E-13	19651	Rbl2
04068	FoxO signaling pathway	8.519930456	1.682E-14	7.27229E-13	16000	Igf1
04068	FoxO signaling pathway	8.519930456	1.682E-14	7.27229E-13	27401	Skp2
04068	FoxO signaling pathway	8.519930456	1.682E-14	7.27229E-13	73251	Setd7
04068	FoxO signaling pathway	8.519930456	1.682E-14	7.27229E-13	12442	Ccnb2
04068	FoxO signaling pathway	8.519930456	1.682E-14	7.27229E-13	18817	Plk1
04068	FoxO signaling pathway	8.519930456	1.682E-14	7.27229E-13	23882	Gadd45g
04068	FoxO signaling pathway	8.519930456	1.682E-14	7.27229E-13	12053	Bcl6
04068	FoxO signaling pathway	8.519930456	1.682E-14	7.27229E-13	67731	Fbxo32
04068	FoxO signaling pathway	8.519930456	1.682E-14	7.27229E-13	16598	Klf2
04068	FoxO signaling pathway	8.519930456	1.682E-14	7.27229E-13	18710	Pik3r3
04068	FoxO signaling pathway	8.519930456	1.682E-14	7.27229E-13	17127	Smad3
04068	FoxO signaling pathway	8.519930456	1.682E-14	7.27229E-13	11920	Atm
04068	FoxO signaling pathway	8.519930456	1.682E-14	7.27229E-13	16001	Igf1r
04068	FoxO signaling pathway	8.519930456	1.682E-14	7.27229E-13	21812	Tgfbr1
04068	FoxO signaling pathway	8.519930456	1.682E-14	7.27229E-13	21813	Tgfbr2
04068	FoxO signaling pathway	8.519930456	1.682E-14	7.27229E-13	18709	Pik3r2
04068	FoxO signaling pathway	8.519930456	1.682E-14	7.27229E-13	27373	Csnk1e
04068	FoxO signaling pathway	8.519930456	1.682E-14	7.27229E-13	108099	Prkag2
04068	FoxO signaling pathway	8.519930456	1.682E-14	7.27229E-13	74551	Pck2
04068	FoxO signaling pathway	8.519930456	1.682E-14	7.27229E-13	13611	S1pr4
04068	FoxO signaling pathway	8.519930456	1.682E-14	7.27229E-13	13609	S1pr1
05202	Transcriptional misregulation in cancer	6.310628934	1.81807E-14	7.27229E-13	21813	Tgfbr2
05202	Transcriptional misregulation in cancer	6.310628934	1.81807E-14	7.27229E-13	70122	Mllt3
05202	Transcriptional misregulation in cancer	6.310628934	1.81807E-14	7.27229E-13	19016	Pparg
05202	Transcriptional misregulation in cancer	6.310628934	1.81807E-14	7.27229E-13	20181	Rxra
05202	Transcriptional misregulation in cancer	6.310628934	1.81807E-14	7.27229E-13	12428	Ccna2
05202	Transcriptional misregulation in cancer	6.310628934	1.81807E-14	7.27229E-13	16000	Igf1
05202	Transcriptional misregulation in cancer	6.310628934	1.81807E-14	7.27229E-13	212980	Slc45a3
05202	Transcriptional misregulation in cancer	6.310628934	1.81807E-14	7.27229E-13	11920	Atm
05202	Transcriptional misregulation in cancer	6.310628934	1.81807E-14	7.27229E-13	16001	Igf1r
05202	Transcriptional misregulation in cancer	6.310628934	1.81807E-14	7.27229E-13	16480	Jup
05202	Transcriptional misregulation in cancer	6.310628934	1.81807E-14	7.27229E-13	18590	Pdgfa
05202	Transcriptional misregulation in cancer	6.310628934	1.81807E-14	7.27229E-13	12580	Cdkn2c
05202	Transcriptional misregulation in cancer	6.310628934	1.81807E-14	7.27229E-13	27015	Polk

05202	Transcriptional misregulation in cancer	6.310628934	1.81807E-14	7.27229E-13	17132	Maf
05202	Transcriptional misregulation in cancer	6.310628934	1.81807E-14	7.27229E-13	14048	Eya1
05202	Transcriptional misregulation in cancer	6.310628934	1.81807E-14	7.27229E-13	12053	Bcl6
05202	Transcriptional misregulation in cancer	6.310628934	1.81807E-14	7.27229E-13	17260	Mef2c
05202	Transcriptional misregulation in cancer	6.310628934	1.81807E-14	7.27229E-13	12393	Runx2
05202	Transcriptional misregulation in cancer	6.310628934	1.81807E-14	7.27229E-13	15446	Hpgd
05202	Transcriptional misregulation in cancer	6.310628934	1.81807E-14	7.27229E-13	107986	Ddb2
05202	Transcriptional misregulation in cancer	6.310628934	1.81807E-14	7.27229E-13	23882	Gadd45g
05202	Transcriptional misregulation in cancer	6.310628934	1.81807E-14	7.27229E-13	15364	Hmga2
05202	Transcriptional misregulation in cancer	6.310628934	1.81807E-14	7.27229E-13	17095	Lyl1
05202	Transcriptional misregulation in cancer	6.310628934	1.81807E-14	7.27229E-13	16421	Itgb7
05202	Transcriptional misregulation in cancer	6.310628934	1.81807E-14	7.27229E-13	12606	Cebpa
05202	Transcriptional misregulation in cancer	6.310628934	1.81807E-14	7.27229E-13	18514	Pbx1
05202	Transcriptional misregulation in cancer	6.310628934	1.81807E-14	7.27229E-13	16909	Lmo2
05202	Transcriptional misregulation in cancer	6.310628934	1.81807E-14	7.27229E-13	15242	Hhex
05202	Transcriptional misregulation in cancer	6.310628934	1.81807E-14	7.27229E-13	18627	Per2
05224	Breast cancer	7.922703743	2.35067E-14	8.35793E-13	18709	Pik3r2
05224	Breast cancer	7.922703743	2.35067E-14	8.35793E-13	12189	Brca1
05224	Breast cancer	7.922703743	2.35067E-14	8.35793E-13	27015	Polk
05224	Breast cancer	7.922703743	2.35067E-14	8.35793E-13	15213	Hey1
05224	Breast cancer	7.922703743	2.35067E-14	8.35793E-13	18710	Pik3r3
05224	Breast cancer	7.922703743	2.35067E-14	8.35793E-13	107986	Ddb2
05224	Breast cancer	7.922703743	2.35067E-14	8.35793E-13	242705	E2f2
05224	Breast cancer	7.922703743	2.35067E-14	8.35793E-13	12190	Brca2
05224	Breast cancer	7.922703743	2.35067E-14	8.35793E-13	23882	Gadd45g
05224	Breast cancer	7.922703743	2.35067E-14	8.35793E-13	14182	Fgfr1
05224	Breast cancer	7.922703743	2.35067E-14	8.35793E-13	16000	Igf1
05224	Breast cancer	7.922703743	2.35067E-14	8.35793E-13	17979	Ncoa3
05224	Breast cancer	7.922703743	2.35067E-14	8.35793E-13	16001	Igf1r
05224	Breast cancer	7.922703743	2.35067E-14	8.35793E-13	14281	Fos
05224	Breast cancer	7.922703743	2.35067E-14	8.35793E-13	16973	Lrp5
05224	Breast cancer	7.922703743	2.35067E-14	8.35793E-13	216148	Shc2
05224	Breast cancer	7.922703743	2.35067E-14	8.35793E-13	271849	Shc4
05224	Breast cancer	7.922703743	2.35067E-14	8.35793E-13	56717	Mtor
05224	Breast cancer	7.922703743	2.35067E-14	8.35793E-13	16842	Lef1
05224	Breast cancer	7.922703743	2.35067E-14	8.35793E-13	14296	Frat1
05224	Breast cancer	7.922703743	2.35067E-14	8.35793E-13	12443	Ccnd1
05224	Breast cancer	7.922703743	2.35067E-14	8.35793E-13	17977	Ncoa1
05224	Breast cancer	7.922703743	2.35067E-14	8.35793E-13	212398	Frat2
05224	Breast cancer	7.922703743	2.35067E-14	8.35793E-13	23805	Apc2
05214	Glioma	11.80375794	3.97311E-14	1.27139E-12	242705	E2f2
05214	Glioma	11.80375794	3.97311E-14	1.27139E-12	18710	Pik3r3

05214	Glioma	11.80375794	3.97311E-14	1.27139E-12	18709	Pik3r2
05214	Glioma	11.80375794	3.97311E-14	1.27139E-12	56717	Mtor
05214	Glioma	11.80375794	3.97311E-14	1.27139E-12	16000	Igf1
05214	Glioma	11.80375794	3.97311E-14	1.27139E-12	18596	Pdgfrb
05214	Glioma	11.80375794	3.97311E-14	1.27139E-12	216148	Shc2
05214	Glioma	11.80375794	3.97311E-14	1.27139E-12	271849	Shc4
05214	Glioma	11.80375794	3.97311E-14	1.27139E-12	18590	Pdgfa
05214	Glioma	11.80375794	3.97311E-14	1.27139E-12	18803	Plcg1
05214	Glioma	11.80375794	3.97311E-14	1.27139E-12	27015	Polk
05214	Glioma	11.80375794	3.97311E-14	1.27139E-12	12443	Ccnd1
05214	Glioma	11.80375794	3.97311E-14	1.27139E-12	52163	Camk1
05214	Glioma	11.80375794	3.97311E-14	1.27139E-12	18591	Pdgfb
05214	Glioma	11.80375794	3.97311E-14	1.27139E-12	23882	Gadd45g
05214	Glioma	11.80375794	3.97311E-14	1.27139E-12	12322	Camk2a
05214	Glioma	11.80375794	3.97311E-14	1.27139E-12	107986	Ddb2
05214	Glioma	11.80375794	3.97311E-14	1.27139E-12	16001	Igf1r
04218	Cellular senescence	6.857013973	6.28893E-14	1.82951E-12	14235	Foxm1
04218	Cellular senescence	6.857013973	6.28893E-14	1.82951E-12	11920	Atm
04218	Cellular senescence	6.857013973	6.28893E-14	1.82951E-12	12428	Ccna2
04218	Cellular senescence	6.857013973	6.28893E-14	1.82951E-12	16440	Itpr3
04218	Cellular senescence	6.857013973	6.28893E-14	1.82951E-12	18709	Pik3r2
04218	Cellular senescence	6.857013973	6.28893E-14	1.82951E-12	18710	Pik3r3
04218	Cellular senescence	6.857013973	6.28893E-14	1.82951E-12	17127	Smad3
04218	Cellular senescence	6.857013973	6.28893E-14	1.82951E-12	18019	Nfatc2
04218	Cellular senescence	6.857013973	6.28893E-14	1.82951E-12	16438	Itpr1
04218	Cellular senescence	6.857013973	6.28893E-14	1.82951E-12	26399	Map2k6
04218	Cellular senescence	6.857013973	6.28893E-14	1.82951E-12	21812	Tgfbr1
04218	Cellular senescence	6.857013973	6.28893E-14	1.82951E-12	12192	Zfp36l1
04218	Cellular senescence	6.857013973	6.28893E-14	1.82951E-12	12193	Zfp36l2
04218	Cellular senescence	6.857013973	6.28893E-14	1.82951E-12	23882	Gadd45g
04218	Cellular senescence	6.857013973	6.28893E-14	1.82951E-12	15258	Hipk2
04218	Cellular senescence	6.857013973	6.28893E-14	1.82951E-12	19651	Rbl2
04218	Cellular senescence	6.857013973	6.28893E-14	1.82951E-12	242705	E2f2
04218	Cellular senescence	6.857013973	6.28893E-14	1.82951E-12	21813	Tgfbr2
04218	Cellular senescence	6.857013973	6.28893E-14	1.82951E-12	12443	Ccnd1
04218	Cellular senescence	6.857013973	6.28893E-14	1.82951E-12	12442	Ccnb2
04218	Cellular senescence	6.857013973	6.28893E-14	1.82951E-12	50883	Chek2
04218	Cellular senescence	6.857013973	6.28893E-14	1.82951E-12	12289	Cacna1d
04218	Cellular senescence	6.857013973	6.28893E-14	1.82951E-12	12334	Capn2
04218	Cellular senescence	6.857013973	6.28893E-14	1.82951E-12	18018	Nfatc1
04218	Cellular senescence	6.857013973	6.28893E-14	1.82951E-12	54354	Rassf5
04218	Cellular senescence	6.857013973	6.28893E-14	1.82951E-12	56717	Mtor

04151	PI3K-Akt signaling pathway	4.731001713	2.05622E-13	5.48325E-12	22340	Vegfb
04151	PI3K-Akt signaling pathway	4.731001713	2.05622E-13	5.48325E-12	18591	Pdgfb
04151	PI3K-Akt signaling pathway	4.731001713	2.05622E-13	5.48325E-12	12189	Brca1
04151	PI3K-Akt signaling pathway	4.731001713	2.05622E-13	5.48325E-12	15234	Hgf
04151	PI3K-Akt signaling pathway	4.731001713	2.05622E-13	5.48325E-12	16194	Il6ra
04151	PI3K-Akt signaling pathway	4.731001713	2.05622E-13	5.48325E-12	208647	Creb3l2
04151	PI3K-Akt signaling pathway	4.731001713	2.05622E-13	5.48325E-12	14256	Flt3l
04151	PI3K-Akt signaling pathway	4.731001713	2.05622E-13	5.48325E-12	18590	Pdgfa
04151	PI3K-Akt signaling pathway	4.731001713	2.05622E-13	5.48325E-12	16542	Kdr
04151	PI3K-Akt signaling pathway	4.731001713	2.05622E-13	5.48325E-12	16774	Lama3
04151	PI3K-Akt signaling pathway	4.731001713	2.05622E-13	5.48325E-12	18710	Pik3r3
04151	PI3K-Akt signaling pathway	4.731001713	2.05622E-13	5.48325E-12	12443	Ccnd1
04151	PI3K-Akt signaling pathway	4.731001713	2.05622E-13	5.48325E-12	74551	Pck2
04151	PI3K-Akt signaling pathway	4.731001713	2.05622E-13	5.48325E-12	11601	Angpt2
04151	PI3K-Akt signaling pathway	4.731001713	2.05622E-13	5.48325E-12	19651	Rbl2
04151	PI3K-Akt signaling pathway	4.731001713	2.05622E-13	5.48325E-12	17311	Kitl
04151	PI3K-Akt signaling pathway	4.731001713	2.05622E-13	5.48325E-12	16421	Itgb7
04151	PI3K-Akt signaling pathway	4.731001713	2.05622E-13	5.48325E-12	56717	Mtor
04151	PI3K-Akt signaling pathway	4.731001713	2.05622E-13	5.48325E-12	18709	Pik3r2
04151	PI3K-Akt signaling pathway	4.731001713	2.05622E-13	5.48325E-12	67168	Lpar6
04151	PI3K-Akt signaling pathway	4.731001713	2.05622E-13	5.48325E-12	14182	Fgfr1
04151	PI3K-Akt signaling pathway	4.731001713	2.05622E-13	5.48325E-12	54635	Pdgfc
04151	PI3K-Akt signaling pathway	4.731001713	2.05622E-13	5.48325E-12	18596	Pdgfrb
04151	PI3K-Akt signaling pathway	4.731001713	2.05622E-13	5.48325E-12	16000	Igf1
04151	PI3K-Akt signaling pathway	4.731001713	2.05622E-13	5.48325E-12	14702	Gng2
04151	PI3K-Akt signaling pathway	4.731001713	2.05622E-13	5.48325E-12	66066	Gng11
04151	PI3K-Akt signaling pathway	4.731001713	2.05622E-13	5.48325E-12	16403	Itga6
04151	PI3K-Akt signaling pathway	4.731001713	2.05622E-13	5.48325E-12	14268	Fn1
04151	PI3K-Akt signaling pathway	4.731001713	2.05622E-13	5.48325E-12	16419	Itgb5
04151	PI3K-Akt signaling pathway	4.731001713	2.05622E-13	5.48325E-12	241226	Itga8
04151	PI3K-Akt signaling pathway	4.731001713	2.05622E-13	5.48325E-12	12830	Col4a5
04151	PI3K-Akt signaling pathway	4.731001713	2.05622E-13	5.48325E-12	20181	Rxra
04151	PI3K-Akt signaling pathway	4.731001713	2.05622E-13	5.48325E-12	381810	Lpar5
04151	PI3K-Akt signaling pathway	4.731001713	2.05622E-13	5.48325E-12	14697	Gnb5
04151	PI3K-Akt signaling pathway	4.731001713	2.05622E-13	5.48325E-12	16001	Igf1r
05210	Colorectal cancer	9.92588736	1.00746E-12	2.47989E-11	17127	Smad3
05210	Colorectal cancer	9.92588736	1.00746E-12	2.47989E-11	21812	Tgfbr1
05210	Colorectal cancer	9.92588736	1.00746E-12	2.47989E-11	16842	Lef1
05210	Colorectal cancer	9.92588736	1.00746E-12	2.47989E-11	56717	Mtor
05210	Colorectal cancer	9.92588736	1.00746E-12	2.47989E-11	12367	Casp3
05210	Colorectal cancer	9.92588736	1.00746E-12	2.47989E-11	21813	Tgfbr2
05210	Colorectal cancer	9.92588736	1.00746E-12	2.47989E-11	170770	Bbc3

05210	Colorectal cancer	9.92588736	1.00746E-12	2.47989E-11	14281	Fos
05210	Colorectal cancer	9.92588736	1.00746E-12	2.47989E-11	23805	Apc2
05210	Colorectal cancer	9.92588736	1.00746E-12	2.47989E-11	17688	Msh6
05210	Colorectal cancer	9.92588736	1.00746E-12	2.47989E-11	17685	Msh2
05210	Colorectal cancer	9.92588736	1.00746E-12	2.47989E-11	27015	Polk
05210	Colorectal cancer	9.92588736	1.00746E-12	2.47989E-11	12443	Ccnd1
05210	Colorectal cancer	9.92588736	1.00746E-12	2.47989E-11	18710	Pik3r3
05210	Colorectal cancer	9.92588736	1.00746E-12	2.47989E-11	17686	Msh3
05210	Colorectal cancer	9.92588736	1.00746E-12	2.47989E-11	23882	Gadd45g
05210	Colorectal cancer	9.92588736	1.00746E-12	2.47989E-11	107986	Ddb2
05210	Colorectal cancer	9.92588736	1.00746E-12	2.47989E-11	18709	Pik3r2
05225	Hepatocellular carcinoma	6.693318679	1.09493E-12	2.5027E-11	66447	Mgst3
05225	Hepatocellular carcinoma	6.693318679	1.09493E-12	2.5027E-11	67155	Smarca2
05225	Hepatocellular carcinoma	6.693318679	1.09493E-12	2.5027E-11	103140	Gstt3
05225	Hepatocellular carcinoma	6.693318679	1.09493E-12	2.5027E-11	16973	Lrp5
05225	Hepatocellular carcinoma	6.693318679	1.09493E-12	2.5027E-11	14296	Frat1
05225	Hepatocellular carcinoma	6.693318679	1.09493E-12	2.5027E-11	17127	Smad3
05225	Hepatocellular carcinoma	6.693318679	1.09493E-12	2.5027E-11	242705	E2f2
05225	Hepatocellular carcinoma	6.693318679	1.09493E-12	2.5027E-11	23805	Apc2
05225	Hepatocellular carcinoma	6.693318679	1.09493E-12	2.5027E-11	15234	Hgf
05225	Hepatocellular carcinoma	6.693318679	1.09493E-12	2.5027E-11	18803	Plcg1
05225	Hepatocellular carcinoma	6.693318679	1.09493E-12	2.5027E-11	212398	Frat2
05225	Hepatocellular carcinoma	6.693318679	1.09493E-12	2.5027E-11	21813	Tgfbr2
05225	Hepatocellular carcinoma	6.693318679	1.09493E-12	2.5027E-11	16842	Lef1
05225	Hepatocellular carcinoma	6.693318679	1.09493E-12	2.5027E-11	18709	Pik3r2
05225	Hepatocellular carcinoma	6.693318679	1.09493E-12	2.5027E-11	16001	Igf1r
05225	Hepatocellular carcinoma	6.693318679	1.09493E-12	2.5027E-11	12443	Ccnd1
05225	Hepatocellular carcinoma	6.693318679	1.09493E-12	2.5027E-11	21812	Tgfbr1
05225	Hepatocellular carcinoma	6.693318679	1.09493E-12	2.5027E-11	56717	Mtor
05225	Hepatocellular carcinoma	6.693318679	1.09493E-12	2.5027E-11	107986	Ddb2
05225	Hepatocellular carcinoma	6.693318679	1.09493E-12	2.5027E-11	216148	Shc2
05225	Hepatocellular carcinoma	6.693318679	1.09493E-12	2.5027E-11	18710	Pik3r3
05225	Hepatocellular carcinoma	6.693318679	1.09493E-12	2.5027E-11	23882	Gadd45g
05225	Hepatocellular carcinoma	6.693318679	1.09493E-12	2.5027E-11	27015	Polk
05225	Hepatocellular carcinoma	6.693318679	1.09493E-12	2.5027E-11	271849	Shc4
05168	Herpes simplex virus 1 infection	4.026221171	2.73846E-12	5.84206E-11	18709	Pik3r2
05168	Herpes simplex virus 1 infection	4.026221171	2.73846E-12	5.84206E-11	56717	Mtor
05168	Herpes simplex virus 1 infection	4.026221171	2.73846E-12	5.84206E-11	14961	H2-Ab1
05168	Herpes simplex virus 1 infection	4.026221171	2.73846E-12	5.84206E-11	14998	H2-DMa
05168	Herpes simplex virus 1 infection	4.026221171	2.73846E-12	5.84206E-11	22751	Zfp90
05168	Herpes simplex virus 1 infection	4.026221171	2.73846E-12	5.84206E-11	14999	H2-DMb1
05168	Herpes simplex virus 1 infection	4.026221171	2.73846E-12	5.84206E-11	22775	Zik1

05168	Herpes simplex virus 1 infection	4.026221171	2.73846E-12	5.84206E-11	231866	Zfp12
05168	Herpes simplex virus 1 infection	4.026221171	2.73846E-12	5.84206E-11	272347	Zfp398
05168	Herpes simplex virus 1 infection	4.026221171	2.73846E-12	5.84206E-11	668039	Gm14434
05168	Herpes simplex virus 1 infection	4.026221171	2.73846E-12	5.84206E-11	16149	Cd74
05168	Herpes simplex virus 1 infection	4.026221171	2.73846E-12	5.84206E-11	14960	H2-Aa
05168	Herpes simplex virus 1 infection	4.026221171	2.73846E-12	5.84206E-11	22719	Zfp61
05168	Herpes simplex virus 1 infection	4.026221171	2.73846E-12	5.84206E-11	384763	Zfp667
05168	Herpes simplex virus 1 infection	4.026221171	2.73846E-12	5.84206E-11	54678	Zfp108
05168	Herpes simplex virus 1 infection	4.026221171	2.73846E-12	5.84206E-11	619331	Zfp551
05168	Herpes simplex virus 1 infection	4.026221171	2.73846E-12	5.84206E-11	244556	Zfp791
05168	Herpes simplex virus 1 infection	4.026221171	2.73846E-12	5.84206E-11	105246961	AB010352
05168	Herpes simplex virus 1 infection	4.026221171	2.73846E-12	5.84206E-11	30944	Zfp354c
05168	Herpes simplex virus 1 infection	4.026221171	2.73846E-12	5.84206E-11	15979	Ifngr1
05168	Herpes simplex virus 1 infection	4.026221171	2.73846E-12	5.84206E-11	22700	Zfp40
05168	Herpes simplex virus 1 infection	4.026221171	2.73846E-12	5.84206E-11	12367	Casp3
05168	Herpes simplex virus 1 infection	4.026221171	2.73846E-12	5.84206E-11	233057	Zfp940
05168	Herpes simplex virus 1 infection	4.026221171	2.73846E-12	5.84206E-11	22643	Zfp101
05168	Herpes simplex virus 1 infection	4.026221171	2.73846E-12	5.84206E-11	668923	Zfp442
05168	Herpes simplex virus 1 infection	4.026221171	2.73846E-12	5.84206E-11	243834	Zfp324
05168	Herpes simplex virus 1 infection	4.026221171	2.73846E-12	5.84206E-11	101835	AW146154
05168	Herpes simplex virus 1 infection	4.026221171	2.73846E-12	5.84206E-11	235050	Zfp810
05168	Herpes simplex virus 1 infection	4.026221171	2.73846E-12	5.84206E-11	56009	Alyref2
05168	Herpes simplex virus 1 infection	4.026221171	2.73846E-12	5.84206E-11	58235	Nectin1
05168	Herpes simplex virus 1 infection	4.026221171	2.73846E-12	5.84206E-11	81897	Tlr9
05168	Herpes simplex virus 1 infection	4.026221171	2.73846E-12	5.84206E-11	67911	Zfp169
05168	Herpes simplex virus 1 infection	4.026221171	2.73846E-12	5.84206E-11	212569	Zfp273
05168	Herpes simplex virus 1 infection	4.026221171	2.73846E-12	5.84206E-11	76373	Zfp773
05168	Herpes simplex virus 1 infection	4.026221171	2.73846E-12	5.84206E-11	239546	Zfp647
05168	Herpes simplex virus 1 infection	4.026221171	2.73846E-12	5.84206E-11	18710	Pik3r3
05168	Herpes simplex virus 1 infection	4.026221171	2.73846E-12	5.84206E-11	72128	2610008E11Rik
05168	Herpes simplex virus 1 infection	4.026221171	2.73846E-12	5.84206E-11	73451	Zfp763
04659	Th17 cell differentiation	8.398827766	2.02734E-11	4.05469E-10	21812	Tgfbr1
04659	Th17 cell differentiation	8.398827766	2.02734E-11	4.05469E-10	18018	Nfatc1
04659	Th17 cell differentiation	8.398827766	2.02734E-11	4.05469E-10	56717	Mtor
04659	Th17 cell differentiation	8.398827766	2.02734E-11	4.05469E-10	16194	Il6ra
04659	Th17 cell differentiation	8.398827766	2.02734E-11	4.05469E-10	14960	H2-Aa
04659	Th17 cell differentiation	8.398827766	2.02734E-11	4.05469E-10	12504	Cd4
04659	Th17 cell differentiation	8.398827766	2.02734E-11	4.05469E-10	21813	Tgfbr2
04659	Th17 cell differentiation	8.398827766	2.02734E-11	4.05469E-10	14999	H2-DMb1
04659	Th17 cell differentiation	8.398827766	2.02734E-11	4.05469E-10	14961	H2-Ab1
04659	Th17 cell differentiation	8.398827766	2.02734E-11	4.05469E-10	15979	Ifngr1
04659	Th17 cell differentiation	8.398827766	2.02734E-11	4.05469E-10	16195	Il6st

04659	Th17 cell differentiation	8.398827766	2.02734E-11	4.05469E-10	18019	Nfatc2
04659	Th17 cell differentiation	8.398827766	2.02734E-11	4.05469E-10	14281	Fos
04659	Th17 cell differentiation	8.398827766	2.02734E-11	4.05469E-10	14998	H2-DMa
04659	Th17 cell differentiation	8.398827766	2.02734E-11	4.05469E-10	50931	Il27ra
04659	Th17 cell differentiation	8.398827766	2.02734E-11	4.05469E-10	18803	Plcg1
04659	Th17 cell differentiation	8.398827766	2.02734E-11	4.05469E-10	20181	Rxra
04659	Th17 cell differentiation	8.398827766	2.02734E-11	4.05469E-10	17127	Smad3
04072	Phospholipase D signaling pathway	6.839313885	2.16878E-11	4.08241E-10	223864	Rapgef3
04072	Phospholipase D signaling pathway	6.839313885	2.16878E-11	4.08241E-10	216148	Shc2
04072	Phospholipase D signaling pathway	6.839313885	2.16878E-11	4.08241E-10	18591	Pdgfb
04072	Phospholipase D signaling pathway	6.839313885	2.16878E-11	4.08241E-10	18710	Pik3r3
04072	Phospholipase D signaling pathway	6.839313885	2.16878E-11	4.08241E-10	271849	Shc4
04072	Phospholipase D signaling pathway	6.839313885	2.16878E-11	4.08241E-10	17311	Kitl
04072	Phospholipase D signaling pathway	6.839313885	2.16878E-11	4.08241E-10	13139	Dgka
04072	Phospholipase D signaling pathway	6.839313885	2.16878E-11	4.08241E-10	18796	Plcb2
04072	Phospholipase D signaling pathway	6.839313885	2.16878E-11	4.08241E-10	18709	Pik3r2
04072	Phospholipase D signaling pathway	6.839313885	2.16878E-11	4.08241E-10	18803	Plcg1
04072	Phospholipase D signaling pathway	6.839313885	2.16878E-11	4.08241E-10	54635	Pdgfc
04072	Phospholipase D signaling pathway	6.839313885	2.16878E-11	4.08241E-10	18590	Pdgfa
04072	Phospholipase D signaling pathway	6.839313885	2.16878E-11	4.08241E-10	110197	Dgkg
04072	Phospholipase D signaling pathway	6.839313885	2.16878E-11	4.08241E-10	67168	Lpar6
04072	Phospholipase D signaling pathway	6.839313885	2.16878E-11	4.08241E-10	18596	Pdgfrb
04072	Phospholipase D signaling pathway	6.839313885	2.16878E-11	4.08241E-10	19157	Cyth1
04072	Phospholipase D signaling pathway	6.839313885	2.16878E-11	4.08241E-10	14673	Gna12
04072	Phospholipase D signaling pathway	6.839313885	2.16878E-11	4.08241E-10	56717	Mtor
04072	Phospholipase D signaling pathway	6.839313885	2.16878E-11	4.08241E-10	381810	Lpar5
04072	Phospholipase D signaling pathway	6.839313885	2.16878E-11	4.08241E-10	11513	Adcy7
04072	Phospholipase D signaling pathway	6.839313885	2.16878E-11	4.08241E-10	104111	Adcy3
05226	Gastric cancer	6.793718459	2.47311E-11	4.20023E-10	16973	Lrp5
05226	Gastric cancer	6.793718459	2.47311E-11	4.20023E-10	23882	Gadd45g
05226	Gastric cancer	6.793718459	2.47311E-11	4.20023E-10	107986	Ddb2
05226	Gastric cancer	6.793718459	2.47311E-11	4.20023E-10	14296	Frat1
05226	Gastric cancer	6.793718459	2.47311E-11	4.20023E-10	212398	Frat2
05226	Gastric cancer	6.793718459	2.47311E-11	4.20023E-10	15234	Hgf
05226	Gastric cancer	6.793718459	2.47311E-11	4.20023E-10	27015	Polk
05226	Gastric cancer	6.793718459	2.47311E-11	4.20023E-10	21813	Tgfbr2
05226	Gastric cancer	6.793718459	2.47311E-11	4.20023E-10	16842	Lef1
05226	Gastric cancer	6.793718459	2.47311E-11	4.20023E-10	23805	Apc2
05226	Gastric cancer	6.793718459	2.47311E-11	4.20023E-10	242705	E2f2
05226	Gastric cancer	6.793718459	2.47311E-11	4.20023E-10	56717	Mtor
05226	Gastric cancer	6.793718459	2.47311E-11	4.20023E-10	271849	Shc4
05226	Gastric cancer	6.793718459	2.47311E-11	4.20023E-10	216148	Shc2

05226	Gastric cancer	6.793718459	2.47311E-11	4.20023E-10	21812	Tgfbr1
05226	Gastric cancer	6.793718459	2.47311E-11	4.20023E-10	16480	Jup
05226	Gastric cancer	6.793718459	2.47311E-11	4.20023E-10	20181	Rxra
05226	Gastric cancer	6.793718459	2.47311E-11	4.20023E-10	12443	Ccnd1
05226	Gastric cancer	6.793718459	2.47311E-11	4.20023E-10	17127	Smad3
05226	Gastric cancer	6.793718459	2.47311E-11	4.20023E-10	18710	Pik3r3
05226	Gastric cancer	6.793718459	2.47311E-11	4.20023E-10	18709	Pik3r2
01521	EGFR tyrosine kinase inhibitor resistance	9.828164137	2.49389E-11	4.20023E-10	16194	Il6ra
01521	EGFR tyrosine kinase inhibitor resistance	9.828164137	2.49389E-11	4.20023E-10	14456	Gas6
01521	EGFR tyrosine kinase inhibitor resistance	9.828164137	2.49389E-11	4.20023E-10	271849	Shc4
01521	EGFR tyrosine kinase inhibitor resistance	9.828164137	2.49389E-11	4.20023E-10	18709	Pik3r2
01521	EGFR tyrosine kinase inhibitor resistance	9.828164137	2.49389E-11	4.20023E-10	216148	Shc2
01521	EGFR tyrosine kinase inhibitor resistance	9.828164137	2.49389E-11	4.20023E-10	54635	Pdgfc
01521	EGFR tyrosine kinase inhibitor resistance	9.828164137	2.49389E-11	4.20023E-10	18591	Pdgfb
01521	EGFR tyrosine kinase inhibitor resistance	9.828164137	2.49389E-11	4.20023E-10	18590	Pdgfa
01521	EGFR tyrosine kinase inhibitor resistance	9.828164137	2.49389E-11	4.20023E-10	18596	Pdgfrb
01521	EGFR tyrosine kinase inhibitor resistance	9.828164137	2.49389E-11	4.20023E-10	18803	Plcg1
01521	EGFR tyrosine kinase inhibitor resistance	9.828164137	2.49389E-11	4.20023E-10	15234	Hgf
01521	EGFR tyrosine kinase inhibitor resistance	9.828164137	2.49389E-11	4.20023E-10	16542	Kdr
01521	EGFR tyrosine kinase inhibitor resistance	9.828164137	2.49389E-11	4.20023E-10	18710	Pik3r3
01521	EGFR tyrosine kinase inhibitor resistance	9.828164137	2.49389E-11	4.20023E-10	16001	Igf1r
01521	EGFR tyrosine kinase inhibitor resistance	9.828164137	2.49389E-11	4.20023E-10	56717	Mtor
01521	EGFR tyrosine kinase inhibitor resistance	9.828164137	2.49389E-11	4.20023E-10	16000	Igf1
04371	Apelin signaling pathway	7.084169405	3.56334E-11	5.70134E-10	18125	Nos1
04371	Apelin signaling pathway	7.084169405	3.56334E-11	5.70134E-10	18576	Pde3b
04371	Apelin signaling pathway	7.084169405	3.56334E-11	5.70134E-10	17258	Mef2a
04371	Apelin signaling pathway	7.084169405	3.56334E-11	5.70134E-10	20541	Slc8a1
04371	Apelin signaling pathway	7.084169405	3.56334E-11	5.70134E-10	21812	Tgfbr1
04371	Apelin signaling pathway	7.084169405	3.56334E-11	5.70134E-10	11513	Adcy7
04371	Apelin signaling pathway	7.084169405	3.56334E-11	5.70134E-10	104111	Adcy3
04371	Apelin signaling pathway	7.084169405	3.56334E-11	5.70134E-10	17260	Mef2c
04371	Apelin signaling pathway	7.084169405	3.56334E-11	5.70134E-10	108099	Prkag2
04371	Apelin signaling pathway	7.084169405	3.56334E-11	5.70134E-10	12443	Ccnd1
04371	Apelin signaling pathway	7.084169405	3.56334E-11	5.70134E-10	14697	Gnb5
04371	Apelin signaling pathway	7.084169405	3.56334E-11	5.70134E-10	14702	Gng2
04371	Apelin signaling pathway	7.084169405	3.56334E-11	5.70134E-10	17127	Smad3
04371	Apelin signaling pathway	7.084169405	3.56334E-11	5.70134E-10	16598	Klf2
04371	Apelin signaling pathway	7.084169405	3.56334E-11	5.70134E-10	56717	Mtor
04371	Apelin signaling pathway	7.084169405	3.56334E-11	5.70134E-10	18796	Plcb2
04371	Apelin signaling pathway	7.084169405	3.56334E-11	5.70134E-10	16440	Itpr3
04371	Apelin signaling pathway	7.084169405	3.56334E-11	5.70134E-10	16438	Itpr1
04371	Apelin signaling pathway	7.084169405	3.56334E-11	5.70134E-10	15184	Hdac5

04371	Apelin signaling pathway	7.084169405	3.56334E-11	5.70134E-10	66066	Gng11
05218	Melanoma	10.10970009	7.38074E-11	1.07854E-09	15234	Hgf
05218	Melanoma	10.10970009	7.38074E-11	1.07854E-09	16000	Igf1
05218	Melanoma	10.10970009	7.38074E-11	1.07854E-09	54635	Pdgfc
05218	Melanoma	10.10970009	7.38074E-11	1.07854E-09	16001	Igf1r
05218	Melanoma	10.10970009	7.38074E-11	1.07854E-09	27015	Polk
05218	Melanoma	10.10970009	7.38074E-11	1.07854E-09	18590	Pdgfa
05218	Melanoma	10.10970009	7.38074E-11	1.07854E-09	14182	Fgfr1
05218	Melanoma	10.10970009	7.38074E-11	1.07854E-09	12443	Ccnd1
05218	Melanoma	10.10970009	7.38074E-11	1.07854E-09	18709	Pik3r2
05218	Melanoma	10.10970009	7.38074E-11	1.07854E-09	18596	Pdgfrb
05218	Melanoma	10.10970009	7.38074E-11	1.07854E-09	107986	Ddb2
05218	Melanoma	10.10970009	7.38074E-11	1.07854E-09	18591	Pdgfb
05218	Melanoma	10.10970009	7.38074E-11	1.07854E-09	242705	E2f2
05218	Melanoma	10.10970009	7.38074E-11	1.07854E-09	23882	Gadd45g
05218	Melanoma	10.10970009	7.38074E-11	1.07854E-09	18710	Pik3r3
04725	Cholinergic synapse	7.798911497	7.41499E-11	1.07854E-09	12287	Cacna1b
04725	Cholinergic synapse	7.798911497	7.41499E-11	1.07854E-09	12286	Cacna1a
04725	Cholinergic synapse	7.798911497	7.41499E-11	1.07854E-09	16438	Itpr1
04725	Cholinergic synapse	7.798911497	7.41499E-11	1.07854E-09	18796	Plcb2
04725	Cholinergic synapse	7.798911497	7.41499E-11	1.07854E-09	11513	Adcy7
04725	Cholinergic synapse	7.798911497	7.41499E-11	1.07854E-09	14702	Gng2
04725	Cholinergic synapse	7.798911497	7.41499E-11	1.07854E-09	208647	Creb3l2
04725	Cholinergic synapse	7.798911497	7.41499E-11	1.07854E-09	12322	Camk2a
04725	Cholinergic synapse	7.798911497	7.41499E-11	1.07854E-09	12289	Cacna1d
04725	Cholinergic synapse	7.798911497	7.41499E-11	1.07854E-09	14697	Gnb5
04725	Cholinergic synapse	7.798911497	7.41499E-11	1.07854E-09	66066	Gng11
04725	Cholinergic synapse	7.798911497	7.41499E-11	1.07854E-09	18710	Pik3r3
04725	Cholinergic synapse	7.798911497	7.41499E-11	1.07854E-09	16440	Itpr3
04725	Cholinergic synapse	7.798911497	7.41499E-11	1.07854E-09	18709	Pik3r2
04725	Cholinergic synapse	7.798911497	7.41499E-11	1.07854E-09	16518	Kcnj2
04725	Cholinergic synapse	7.798911497	7.41499E-11	1.07854E-09	14281	Fos
04725	Cholinergic synapse	7.798911497	7.41499E-11	1.07854E-09	104111	Adcy3
04725	Cholinergic synapse	7.798911497	7.41499E-11	1.07854E-09	110862	Kcnq3
04926	Relaxin signaling pathway	7.147322853	1.00001E-10	1.39132E-09	18796	Plcb2
04926	Relaxin signaling pathway	7.147322853	1.00001E-10	1.39132E-09	12830	Col4a5
04926	Relaxin signaling pathway	7.147322853	1.00001E-10	1.39132E-09	14702	Gng2
04926	Relaxin signaling pathway	7.147322853	1.00001E-10	1.39132E-09	14697	Gnb5
04926	Relaxin signaling pathway	7.147322853	1.00001E-10	1.39132E-09	208647	Creb3l2
04926	Relaxin signaling pathway	7.147322853	1.00001E-10	1.39132E-09	22340	Vegfb
04926	Relaxin signaling pathway	7.147322853	1.00001E-10	1.39132E-09	18125	Nos1
04926	Relaxin signaling pathway	7.147322853	1.00001E-10	1.39132E-09	104111	Adcy3

04926	Relaxin signaling pathway	7.147322853	1.00001E-10	1.39132E-09	21812	Tgfbr1
04926	Relaxin signaling pathway	7.147322853	1.00001E-10	1.39132E-09	21813	Tgfbr2
04926	Relaxin signaling pathway	7.147322853	1.00001E-10	1.39132E-09	11513	Adcy7
04926	Relaxin signaling pathway	7.147322853	1.00001E-10	1.39132E-09	271849	Shc4
04926	Relaxin signaling pathway	7.147322853	1.00001E-10	1.39132E-09	109689	Arrb1
04926	Relaxin signaling pathway	7.147322853	1.00001E-10	1.39132E-09	18709	Pik3r2
04926	Relaxin signaling pathway	7.147322853	1.00001E-10	1.39132E-09	14281	Fos
04926	Relaxin signaling pathway	7.147322853	1.00001E-10	1.39132E-09	14676	Gna15
04926	Relaxin signaling pathway	7.147322853	1.00001E-10	1.39132E-09	18710	Pik3r3
04926	Relaxin signaling pathway	7.147322853	1.00001E-10	1.39132E-09	66066	Gng11
04926	Relaxin signaling pathway	7.147322853	1.00001E-10	1.39132E-09	216148	Shc2
04510	Focal adhesion	5.552790496	1.69895E-10	2.26527E-09	16774	Lama3
04510	Focal adhesion	5.552790496	1.69895E-10	2.26527E-09	12443	Ccnd1
04510	Focal adhesion	5.552790496	1.69895E-10	2.26527E-09	18709	Pik3r2
04510	Focal adhesion	5.552790496	1.69895E-10	2.26527E-09	14268	Fn1
04510	Focal adhesion	5.552790496	1.69895E-10	2.26527E-09	18590	Pdgfa
04510	Focal adhesion	5.552790496	1.69895E-10	2.26527E-09	16403	Itga6
04510	Focal adhesion	5.552790496	1.69895E-10	2.26527E-09	12830	Col4a5
04510	Focal adhesion	5.552790496	1.69895E-10	2.26527E-09	22325	Vav2
04510	Focal adhesion	5.552790496	1.69895E-10	2.26527E-09	271849	Shc4
04510	Focal adhesion	5.552790496	1.69895E-10	2.26527E-09	16542	Kdr
04510	Focal adhesion	5.552790496	1.69895E-10	2.26527E-09	18710	Pik3r3
04510	Focal adhesion	5.552790496	1.69895E-10	2.26527E-09	16001	Igf1r
04510	Focal adhesion	5.552790496	1.69895E-10	2.26527E-09	15234	Hgf
04510	Focal adhesion	5.552790496	1.69895E-10	2.26527E-09	16419	Itgb5
04510	Focal adhesion	5.552790496	1.69895E-10	2.26527E-09	18591	Pdgfb
04510	Focal adhesion	5.552790496	1.69895E-10	2.26527E-09	16000	Igf1
04510	Focal adhesion	5.552790496	1.69895E-10	2.26527E-09	22340	Vegfb
04510	Focal adhesion	5.552790496	1.69895E-10	2.26527E-09	241226	Itga8
04510	Focal adhesion	5.552790496	1.69895E-10	2.26527E-09	16421	Itgb7
04510	Focal adhesion	5.552790496	1.69895E-10	2.26527E-09	12334	Capn2
04510	Focal adhesion	5.552790496	1.69895E-10	2.26527E-09	216148	Shc2
04510	Focal adhesion	5.552790496	1.69895E-10	2.26527E-09	54635	Pdgfc
04510	Focal adhesion	5.552790496	1.69895E-10	2.26527E-09	18596	Pdgfrb
05205	Proteoglycans in cancer	5.444443365	2.53849E-10	3.24927E-09	16001	Igf1r
05205	Proteoglycans in cancer	5.444443365	2.53849E-10	3.24927E-09	15442	Hpse
05205	Proteoglycans in cancer	5.444443365	2.53849E-10	3.24927E-09	14268	Fn1
05205	Proteoglycans in cancer	5.444443365	2.53849E-10	3.24927E-09	18803	Plcg1
05205	Proteoglycans in cancer	5.444443365	2.53849E-10	3.24927E-09	18569	Pdcd4
05205	Proteoglycans in cancer	5.444443365	2.53849E-10	3.24927E-09	16419	Itgb5
05205	Proteoglycans in cancer	5.444443365	2.53849E-10	3.24927E-09	14182	Fgfr1
05205	Proteoglycans in cancer	5.444443365	2.53849E-10	3.24927E-09	12322	Camk2a

05205	Proteoglycans in cancer	5.444443365	2.53849E-10	3.24927E-09	109676	Ank2
05205	Proteoglycans in cancer	5.444443365	2.53849E-10	3.24927E-09	56717	Mtor
05205	Proteoglycans in cancer	5.444443365	2.53849E-10	3.24927E-09	12443	Ccnd1
05205	Proteoglycans in cancer	5.444443365	2.53849E-10	3.24927E-09	15234	Hgf
05205	Proteoglycans in cancer	5.444443365	2.53849E-10	3.24927E-09	18710	Pik3r3
05205	Proteoglycans in cancer	5.444443365	2.53849E-10	3.24927E-09	16542	Kdr
05205	Proteoglycans in cancer	5.444443365	2.53849E-10	3.24927E-09	83383	Tfap4
05205	Proteoglycans in cancer	5.444443365	2.53849E-10	3.24927E-09	12367	Casp3
05205	Proteoglycans in cancer	5.444443365	2.53849E-10	3.24927E-09	21844	Tiam1
05205	Proteoglycans in cancer	5.444443365	2.53849E-10	3.24927E-09	16438	Itpr1
05205	Proteoglycans in cancer	5.444443365	2.53849E-10	3.24927E-09	18709	Pik3r2
05205	Proteoglycans in cancer	5.444443365	2.53849E-10	3.24927E-09	16000	Igf1
05205	Proteoglycans in cancer	5.444443365	2.53849E-10	3.24927E-09	22325	Vav2
05205	Proteoglycans in cancer	5.444443365	2.53849E-10	3.24927E-09	16440	Itpr3
05205	Proteoglycans in cancer	5.444443365	2.53849E-10	3.24927E-09	11733	Ank1
05167	Kaposi sarcoma-associated herpesvirus infection	5.176166445	2.84873E-10	3.50613E-09	271375	Cd200r2
05167	Kaposi sarcoma-associated herpesvirus infection	5.176166445	2.84873E-10	3.50613E-09	12774	Ccr5
05167	Kaposi sarcoma-associated herpesvirus infection	5.176166445	2.84873E-10	3.50613E-09	18018	Nfatc1
05167	Kaposi sarcoma-associated herpesvirus infection	5.176166445	2.84873E-10	3.50613E-09	239849	Cd200r4
05167	Kaposi sarcoma-associated herpesvirus infection	5.176166445	2.84873E-10	3.50613E-09	26399	Map2k6
05167	Kaposi sarcoma-associated herpesvirus infection	5.176166445	2.84873E-10	3.50613E-09	16842	Lef1
05167	Kaposi sarcoma-associated herpesvirus infection	5.176166445	2.84873E-10	3.50613E-09	12443	Ccnd1
05167	Kaposi sarcoma-associated herpesvirus infection	5.176166445	2.84873E-10	3.50613E-09	18019	Nfatc2
05167	Kaposi sarcoma-associated herpesvirus infection	5.176166445	2.84873E-10	3.50613E-09	14281	Fos
05167	Kaposi sarcoma-associated herpesvirus infection	5.176166445	2.84873E-10	3.50613E-09	16438	Itpr1
05167	Kaposi sarcoma-associated herpesvirus infection	5.176166445	2.84873E-10	3.50613E-09	18591	Pdgfb
05167	Kaposi sarcoma-associated herpesvirus infection	5.176166445	2.84873E-10	3.50613E-09	15979	Ifngr1
05167	Kaposi sarcoma-associated herpesvirus infection	5.176166445	2.84873E-10	3.50613E-09	56717	Mtor
05167	Kaposi sarcoma-associated herpesvirus infection	5.176166445	2.84873E-10	3.50613E-09	14702	Gng2
05167	Kaposi sarcoma-associated herpesvirus infection	5.176166445	2.84873E-10	3.50613E-09	66066	Gng11
05167	Kaposi sarcoma-associated herpesvirus infection	5.176166445	2.84873E-10	3.50613E-09	18803	Plcg1
05167	Kaposi sarcoma-associated herpesvirus infection	5.176166445	2.84873E-10	3.50613E-09	14697	Gnb5
05167	Kaposi sarcoma-associated herpesvirus infection	5.176166445	2.84873E-10	3.50613E-09	16195	Il6st
05167	Kaposi sarcoma-associated herpesvirus infection	5.176166445	2.84873E-10	3.50613E-09	12367	Casp3
05167	Kaposi sarcoma-associated herpesvirus infection	5.176166445	2.84873E-10	3.50613E-09	11601	Angpt2
05167	Kaposi sarcoma-associated herpesvirus infection	5.176166445	2.84873E-10	3.50613E-09	16440	Itpr3
05167	Kaposi sarcoma-associated herpesvirus infection	5.176166445	2.84873E-10	3.50613E-09	18709	Pik3r2
05167	Kaposi sarcoma-associated herpesvirus infection	5.176166445	2.84873E-10	3.50613E-09	242705	E2f2
05167	Kaposi sarcoma-associated herpesvirus infection	5.176166445	2.84873E-10	3.50613E-09	18710	Pik3r3
05166	Human T-cell leukemia virus 1 infection	4.911595185	3.58587E-10	4.23489E-09	14960	H2-Aa
05166	Human T-cell leukemia virus 1 infection	4.911595185	3.58587E-10	4.23489E-09	14281	Fos
05166	Human T-cell leukemia virus 1 infection	4.911595185	3.58587E-10	4.23489E-09	242705	E2f2

05166	Human T-cell leukemia virus 1 infection	4.911595185	3.58587E-10	4.23489E-09	18709	Pik3r2
05166	Human T-cell leukemia virus 1 infection	4.911595185	3.58587E-10	4.23489E-09	11920	Atm
05166	Human T-cell leukemia virus 1 infection	4.911595185	3.58587E-10	4.23489E-09	17127	Smad3
05166	Human T-cell leukemia virus 1 infection	4.911595185	3.58587E-10	4.23489E-09	12442	Ccnb2
05166	Human T-cell leukemia virus 1 infection	4.911595185	3.58587E-10	4.23489E-09	12580	Cdkn2c
05166	Human T-cell leukemia virus 1 infection	4.911595185	3.58587E-10	4.23489E-09	21813	Tgfbr2
05166	Human T-cell leukemia virus 1 infection	4.911595185	3.58587E-10	4.23489E-09	12443	Ccnd1
05166	Human T-cell leukemia virus 1 infection	4.911595185	3.58587E-10	4.23489E-09	50883	Chek2
05166	Human T-cell leukemia virus 1 infection	4.911595185	3.58587E-10	4.23489E-09	26401	Map3k1
05166	Human T-cell leukemia virus 1 infection	4.911595185	3.58587E-10	4.23489E-09	12504	Cd4
05166	Human T-cell leukemia virus 1 infection	4.911595185	3.58587E-10	4.23489E-09	21812	Tgfbr1
05166	Human T-cell leukemia virus 1 infection	4.911595185	3.58587E-10	4.23489E-09	14961	H2-Ab1
05166	Human T-cell leukemia virus 1 infection	4.911595185	3.58587E-10	4.23489E-09	14999	H2-DMb1
05166	Human T-cell leukemia virus 1 infection	4.911595185	3.58587E-10	4.23489E-09	18710	Pik3r3
05166	Human T-cell leukemia virus 1 infection	4.911595185	3.58587E-10	4.23489E-09	208647	Creb3l2
05166	Human T-cell leukemia virus 1 infection	4.911595185	3.58587E-10	4.23489E-09	30939	Pttg1
05166	Human T-cell leukemia virus 1 infection	4.911595185	3.58587E-10	4.23489E-09	12428	Ccna2
05166	Human T-cell leukemia virus 1 infection	4.911595185	3.58587E-10	4.23489E-09	11513	Adcy7
05166	Human T-cell leukemia virus 1 infection	4.911595185	3.58587E-10	4.23489E-09	18019	Nfatc2
05166	Human T-cell leukemia virus 1 infection	4.911595185	3.58587E-10	4.23489E-09	14998	H2-DMa
05166	Human T-cell leukemia virus 1 infection	4.911595185	3.58587E-10	4.23489E-09	104111	Adcy3
05166	Human T-cell leukemia virus 1 infection	4.911595185	3.58587E-10	4.23489E-09	18018	Nfatc1
04110	Cell cycle	7.101447867	3.70553E-10	4.23489E-09	19090	Prkdc
04110	Cell cycle	7.101447867	3.70553E-10	4.23489E-09	211586	Tfdp2
04110	Cell cycle	7.101447867	3.70553E-10	4.23489E-09	50883	Chek2
04110	Cell cycle	7.101447867	3.70553E-10	4.23489E-09	18817	Plk1
04110	Cell cycle	7.101447867	3.70553E-10	4.23489E-09	27401	Skp2
04110	Cell cycle	7.101447867	3.70553E-10	4.23489E-09	12580	Cdkn2c
04110	Cell cycle	7.101447867	3.70553E-10	4.23489E-09	17127	Smad3
04110	Cell cycle	7.101447867	3.70553E-10	4.23489E-09	242705	E2f2
04110	Cell cycle	7.101447867	3.70553E-10	4.23489E-09	17219	Mcm6
04110	Cell cycle	7.101447867	3.70553E-10	4.23489E-09	12428	Ccna2
04110	Cell cycle	7.101447867	3.70553E-10	4.23489E-09	12443	Ccnd1
04110	Cell cycle	7.101447867	3.70553E-10	4.23489E-09	11920	Atm
04110	Cell cycle	7.101447867	3.70553E-10	4.23489E-09	23882	Gadd45g
04110	Cell cycle	7.101447867	3.70553E-10	4.23489E-09	30939	Pttg1
04110	Cell cycle	7.101447867	3.70553E-10	4.23489E-09	12442	Ccnb2
04110	Cell cycle	7.101447867	3.70553E-10	4.23489E-09	22390	Wee1
04110	Cell cycle	7.101447867	3.70553E-10	4.23489E-09	12531	Cdc25b
04110	Cell cycle	7.101447867	3.70553E-10	4.23489E-09	19651	Rbl2
04514	Cell adhesion molecules	5.856653844	4.31858E-10	4.76533E-09	14998	H2-DMa
04514	Cell adhesion molecules	5.856653844	4.31858E-10	4.76533E-09	14961	H2-Ab1

04514	Cell adhesion molecules	5.856653844	4.31858E-10	4.76533E-09	319504	Nrcam
04514	Cell adhesion molecules	5.856653844	4.31858E-10	4.76533E-09	12490	Cd34
04514	Cell adhesion molecules	5.856653844	4.31858E-10	4.76533E-09	16728	L1cam
04514	Cell adhesion molecules	5.856653844	4.31858E-10	4.76533E-09	12504	Cd4
04514	Cell adhesion molecules	5.856653844	4.31858E-10	4.76533E-09	16421	Itgb7
04514	Cell adhesion molecules	5.856653844	4.31858E-10	4.76533E-09	58235	Nectin1
04514	Cell adhesion molecules	5.856653844	4.31858E-10	4.76533E-09	14960	H2-Aa
04514	Cell adhesion molecules	5.856653844	4.31858E-10	4.76533E-09	17528	Mpz
04514	Cell adhesion molecules	5.856653844	4.31858E-10	4.76533E-09	18007	Neo1
04514	Cell adhesion molecules	5.856653844	4.31858E-10	4.76533E-09	14999	H2-DMb1
04514	Cell adhesion molecules	5.856653844	4.31858E-10	4.76533E-09	54725	Cadm1
04514	Cell adhesion molecules	5.856653844	4.31858E-10	4.76533E-09	241226	Itga8
04514	Cell adhesion molecules	5.856653844	4.31858E-10	4.76533E-09	12483	Cd22
04514	Cell adhesion molecules	5.856653844	4.31858E-10	4.76533E-09	12481	Cd2
04514	Cell adhesion molecules	5.856653844	4.31858E-10	4.76533E-09	16403	Itga6
04514	Cell adhesion molecules	5.856653844	4.31858E-10	4.76533E-09	74048	Vsir
04514	Cell adhesion molecules	5.856653844	4.31858E-10	4.76533E-09	54167	Icos
04514	Cell adhesion molecules	5.856653844	4.31858E-10	4.76533E-09	207683	Igsf11
04514	Cell adhesion molecules	5.856653844	4.31858E-10	4.76533E-09	12487	Cd28
04070	Phosphatidylinositol signaling system	8.087760071	5.51638E-10	5.75404E-09	20975	Synj2
04070	Phosphatidylinositol signaling system	8.087760071	5.51638E-10	5.75404E-09	13139	Dgka
04070	Phosphatidylinositol signaling system	8.087760071	5.51638E-10	5.75404E-09	18709	Pik3r2
04070	Phosphatidylinositol signaling system	8.087760071	5.51638E-10	5.75404E-09	18803	Plcg1
04070	Phosphatidylinositol signaling system	8.087760071	5.51638E-10	5.75404E-09	110197	Dgkg
04070	Phosphatidylinositol signaling system	8.087760071	5.51638E-10	5.75404E-09	114663	Impa2
04070	Phosphatidylinositol signaling system	8.087760071	5.51638E-10	5.75404E-09	101490	Inpp5f
04070	Phosphatidylinositol signaling system	8.087760071	5.51638E-10	5.75404E-09	16329	Inpp1
04070	Phosphatidylinositol signaling system	8.087760071	5.51638E-10	5.75404E-09	320404	Itpkb
04070	Phosphatidylinositol signaling system	8.087760071	5.51638E-10	5.75404E-09	18710	Pik3r3
04070	Phosphatidylinositol signaling system	8.087760071	5.51638E-10	5.75404E-09	269180	Inpp4a
04070	Phosphatidylinositol signaling system	8.087760071	5.51638E-10	5.75404E-09	18718	Pip4k2a
04070	Phosphatidylinositol signaling system	8.087760071	5.51638E-10	5.75404E-09	18796	Plcb2
04070	Phosphatidylinositol signaling system	8.087760071	5.51638E-10	5.75404E-09	16440	Itpr3
04070	Phosphatidylinositol signaling system	8.087760071	5.51638E-10	5.75404E-09	16438	Itpr1
04070	Phosphatidylinositol signaling system	8.087760071	5.51638E-10	5.75404E-09	16332	Inppl1
04152	AMPK signaling pathway	6.932365775	5.57423E-10	5.75404E-09	208647	Creb3l2
04152	AMPK signaling pathway	6.932365775	5.57423E-10	5.75404E-09	18642	Pfkm
04152	AMPK signaling pathway	6.932365775	5.57423E-10	5.75404E-09	18640	Pfkfb2
04152	AMPK signaling pathway	6.932365775	5.57423E-10	5.75404E-09	12428	Ccna2
04152	AMPK signaling pathway	6.932365775	5.57423E-10	5.75404E-09	108099	Prkag2
04152	AMPK signaling pathway	6.932365775	5.57423E-10	5.75404E-09	16000	Igf1
04152	AMPK signaling pathway	6.932365775	5.57423E-10	5.75404E-09	16001	Igf1r

04152	AMPK signaling pathway	6.932365775	5.57423E-10	5.75404E-09	22241	Ulk1
04152	AMPK signaling pathway	6.932365775	5.57423E-10	5.75404E-09	100705	Acacb
04152	AMPK signaling pathway	6.932365775	5.57423E-10	5.75404E-09	11549	Adra1a
04152	AMPK signaling pathway	6.932365775	5.57423E-10	5.75404E-09	18710	Pik3r3
04152	AMPK signaling pathway	6.932365775	5.57423E-10	5.75404E-09	12894	Cpt1a
04152	AMPK signaling pathway	6.932365775	5.57423E-10	5.75404E-09	19016	Pparg
04152	AMPK signaling pathway	6.932365775	5.57423E-10	5.75404E-09	74551	Pck2
04152	AMPK signaling pathway	6.932365775	5.57423E-10	5.75404E-09	13631	Eef2k
04152	AMPK signaling pathway	6.932365775	5.57423E-10	5.75404E-09	12443	Ccnd1
04152	AMPK signaling pathway	6.932365775	5.57423E-10	5.75404E-09	18709	Pik3r2
04152	AMPK signaling pathway	6.932365775	5.57423E-10	5.75404E-09	56717	Mtor
04310	Wnt signaling pathway	5.990933386	7.91381E-10	7.91381E-09	68010	Bambi
04310	Wnt signaling pathway	5.990933386	7.91381E-10	7.91381E-09	407821	Znrf3
04310	Wnt signaling pathway	5.990933386	7.91381E-10	7.91381E-09	14296	Frat1
04310	Wnt signaling pathway	5.990933386	7.91381E-10	7.91381E-09	81004	Tbl1xr1
04310	Wnt signaling pathway	5.990933386	7.91381E-10	7.91381E-09	212398	Frat2
04310	Wnt signaling pathway	5.990933386	7.91381E-10	7.91381E-09	27373	Csnk1e
04310	Wnt signaling pathway	5.990933386	7.91381E-10	7.91381E-09	18018	Nfatc1
04310	Wnt signaling pathway	5.990933386	7.91381E-10	7.91381E-09	73739	Cby1
04310	Wnt signaling pathway	5.990933386	7.91381E-10	7.91381E-09	16973	Lrp5
04310	Wnt signaling pathway	5.990933386	7.91381E-10	7.91381E-09	23805	Apc2
04310	Wnt signaling pathway	5.990933386	7.91381E-10	7.91381E-09	21372	Tbl1x
04310	Wnt signaling pathway	5.990933386	7.91381E-10	7.91381E-09	18019	Nfatc2
04310	Wnt signaling pathway	5.990933386	7.91381E-10	7.91381E-09	12443	Ccnd1
04310	Wnt signaling pathway	5.990933386	7.91381E-10	7.91381E-09	16842	Lef1
04310	Wnt signaling pathway	5.990933386	7.91381E-10	7.91381E-09	67087	Ctnnbip1
04310	Wnt signaling pathway	5.990933386	7.91381E-10	7.91381E-09	17127	Smad3
04310	Wnt signaling pathway	5.990933386	7.91381E-10	7.91381E-09	243548	Prickle2
04310	Wnt signaling pathway	5.990933386	7.91381E-10	7.91381E-09	93840	Vangl2
04310	Wnt signaling pathway	5.990933386	7.91381E-10	7.91381E-09	12322	Camk2a
04310	Wnt signaling pathway	5.990933386	7.91381E-10	7.91381E-09	18796	Plcb2
04115	p53 signaling pathway	9.435720083	9.21235E-10	8.93318E-09	12442	Ccnb2
04115	p53 signaling pathway	9.435720083	9.21235E-10	8.93318E-09	12367	Casp3
04115	p53 signaling pathway	9.435720083	9.21235E-10	8.93318E-09	75747	Sesn3
04115	p53 signaling pathway	9.435720083	9.21235E-10	8.93318E-09	140742	Sesn1
04115	p53 signaling pathway	9.435720083	9.21235E-10	8.93318E-09	29870	Gtse1
04115	p53 signaling pathway	9.435720083	9.21235E-10	8.93318E-09	16000	Igf1
04115	p53 signaling pathway	9.435720083	9.21235E-10	8.93318E-09	23882	Gadd45g
04115	p53 signaling pathway	9.435720083	9.21235E-10	8.93318E-09	68428	Steap3
04115	p53 signaling pathway	9.435720083	9.21235E-10	8.93318E-09	12443	Ccnd1
04115	p53 signaling pathway	9.435720083	9.21235E-10	8.93318E-09	170770	Bbc3
04115	p53 signaling pathway	9.435720083	9.21235E-10	8.93318E-09	50883	Chek2

04115	p53 signaling pathway	9.435720083	9.21235E-10	8.93318E-09	107986	Ddb2
04115	p53 signaling pathway	9.435720083	9.21235E-10	8.93318E-09	20135	Rrm2
04115	p53 signaling pathway	9.435720083	9.21235E-10	8.93318E-09	11920	Atm
04810	Regulation of actin cytoskeleton	5.073231317	1.05195E-09	9.90072E-09	21844	Tiam1
04810	Regulation of actin cytoskeleton	5.073231317	1.05195E-09	9.90072E-09	226970	Arhgef4
04810	Regulation of actin cytoskeleton	5.073231317	1.05195E-09	9.90072E-09	18710	Pik3r3
04810	Regulation of actin cytoskeleton	5.073231317	1.05195E-09	9.90072E-09	22325	Vav2
04810	Regulation of actin cytoskeleton	5.073231317	1.05195E-09	9.90072E-09	23805	Apc2
04810	Regulation of actin cytoskeleton	5.073231317	1.05195E-09	9.90072E-09	18709	Pik3r2
04810	Regulation of actin cytoskeleton	5.073231317	1.05195E-09	9.90072E-09	14182	Fgfr1
04810	Regulation of actin cytoskeleton	5.073231317	1.05195E-09	9.90072E-09	18596	Pdgfrb
04810	Regulation of actin cytoskeleton	5.073231317	1.05195E-09	9.90072E-09	54635	Pdgfc
04810	Regulation of actin cytoskeleton	5.073231317	1.05195E-09	9.90072E-09	108100	Baiap2
04810	Regulation of actin cytoskeleton	5.073231317	1.05195E-09	9.90072E-09	18591	Pdgfb
04810	Regulation of actin cytoskeleton	5.073231317	1.05195E-09	9.90072E-09	14673	Gna12
04810	Regulation of actin cytoskeleton	5.073231317	1.05195E-09	9.90072E-09	404710	Iqgap3
04810	Regulation of actin cytoskeleton	5.073231317	1.05195E-09	9.90072E-09	18718	Pip4k2a
04810	Regulation of actin cytoskeleton	5.073231317	1.05195E-09	9.90072E-09	18590	Pdgfa
04810	Regulation of actin cytoskeleton	5.073231317	1.05195E-09	9.90072E-09	237860	Ssh2
04810	Regulation of actin cytoskeleton	5.073231317	1.05195E-09	9.90072E-09	381810	Lpar5
04810	Regulation of actin cytoskeleton	5.073231317	1.05195E-09	9.90072E-09	16403	Itga6
04810	Regulation of actin cytoskeleton	5.073231317	1.05195E-09	9.90072E-09	241226	Itga8
04810	Regulation of actin cytoskeleton	5.073231317	1.05195E-09	9.90072E-09	14268	Fn1
04810	Regulation of actin cytoskeleton	5.073231317	1.05195E-09	9.90072E-09	16421	Itgb7
04810	Regulation of actin cytoskeleton	5.073231317	1.05195E-09	9.90072E-09	12767	Cxcr4
04810	Regulation of actin cytoskeleton	5.073231317	1.05195E-09	9.90072E-09	16419	Itgb5
04935	Growth hormone synthesis, secretion and action	7.111651097	1.19651E-09	1.09395E-08	14281	Fos
04935	Growth hormone synthesis, secretion and action	7.111651097	1.19651E-09	1.09395E-08	26401	Map3k1
04935	Growth hormone synthesis, secretion and action	7.111651097	1.19651E-09	1.09395E-08	16000	Igf1
04935	Growth hormone synthesis, secretion and action	7.111651097	1.19651E-09	1.09395E-08	104111	Adcy3
04935	Growth hormone synthesis, secretion and action	7.111651097	1.19651E-09	1.09395E-08	216148	Shc2
04935	Growth hormone synthesis, secretion and action	7.111651097	1.19651E-09	1.09395E-08	12289	Cacna1d
04935	Growth hormone synthesis, secretion and action	7.111651097	1.19651E-09	1.09395E-08	56717	Mtor
04935	Growth hormone synthesis, secretion and action	7.111651097	1.19651E-09	1.09395E-08	26399	Map2k6
04935	Growth hormone synthesis, secretion and action	7.111651097	1.19651E-09	1.09395E-08	271849	Shc4
04935	Growth hormone synthesis, secretion and action	7.111651097	1.19651E-09	1.09395E-08	208647	Creb3l2
04935	Growth hormone synthesis, secretion and action	7.111651097	1.19651E-09	1.09395E-08	18803	Plcg1
04935	Growth hormone synthesis, secretion and action	7.111651097	1.19651E-09	1.09395E-08	18710	Pik3r3
04935	Growth hormone synthesis, secretion and action	7.111651097	1.19651E-09	1.09395E-08	11513	Adcy7
04935	Growth hormone synthesis, secretion and action	7.111651097	1.19651E-09	1.09395E-08	16440	Itpr3
04935	Growth hormone synthesis, secretion and action	7.111651097	1.19651E-09	1.09395E-08	18709	Pik3r2
04935	Growth hormone synthesis, secretion and action	7.111651097	1.19651E-09	1.09395E-08	18796	Plcb2

04935	Growth hormone synthesis, secretion and action	7.111651097	1.19651E-09	1.09395E-08	16438	Itpr1
04658	Th1 and Th2 cell differentiation	8.2715728	1.49009E-09	1.32453E-08	14960	H2-Aa
04658	Th1 and Th2 cell differentiation	8.2715728	1.49009E-09	1.32453E-08	16162	Il12rb2
04658	Th1 and Th2 cell differentiation	8.2715728	1.49009E-09	1.32453E-08	270118	Maml2
04658	Th1 and Th2 cell differentiation	8.2715728	1.49009E-09	1.32453E-08	433586	Maml3
04658	Th1 and Th2 cell differentiation	8.2715728	1.49009E-09	1.32453E-08	15979	Ifngr1
04658	Th1 and Th2 cell differentiation	8.2715728	1.49009E-09	1.32453E-08	14999	H2-DMb1
04658	Th1 and Th2 cell differentiation	8.2715728	1.49009E-09	1.32453E-08	12504	Cd4
04658	Th1 and Th2 cell differentiation	8.2715728	1.49009E-09	1.32453E-08	14961	H2-Ab1
04658	Th1 and Th2 cell differentiation	8.2715728	1.49009E-09	1.32453E-08	18803	Plcg1
04658	Th1 and Th2 cell differentiation	8.2715728	1.49009E-09	1.32453E-08	14998	H2-DMa
04658	Th1 and Th2 cell differentiation	8.2715728	1.49009E-09	1.32453E-08	17132	Maf
04658	Th1 and Th2 cell differentiation	8.2715728	1.49009E-09	1.32453E-08	14281	Fos
04658	Th1 and Th2 cell differentiation	8.2715728	1.49009E-09	1.32453E-08	12399	Runx3
04658	Th1 and Th2 cell differentiation	8.2715728	1.49009E-09	1.32453E-08	18018	Nfatc1
04658	Th1 and Th2 cell differentiation	8.2715728	1.49009E-09	1.32453E-08	18019	Nfatc2
04921	Oxytocin signaling pathway	6.02617417	2.01666E-09	1.74414E-08	12298	Cacnb4
04921	Oxytocin signaling pathway	6.02617417	2.01666E-09	1.74414E-08	12322	Camk2a
04921	Oxytocin signaling pathway	6.02617417	2.01666E-09	1.74414E-08	13631	Eef2k
04921	Oxytocin signaling pathway	6.02617417	2.01666E-09	1.74414E-08	12443	Ccnd1
04921	Oxytocin signaling pathway	6.02617417	2.01666E-09	1.74414E-08	12289	Cacna1d
04921	Oxytocin signaling pathway	6.02617417	2.01666E-09	1.74414E-08	319734	Cacna2d4
04921	Oxytocin signaling pathway	6.02617417	2.01666E-09	1.74414E-08	52163	Camk1
04921	Oxytocin signaling pathway	6.02617417	2.01666E-09	1.74414E-08	104111	Adcy3
04921	Oxytocin signaling pathway	6.02617417	2.01666E-09	1.74414E-08	18019	Nfatc2
04921	Oxytocin signaling pathway	6.02617417	2.01666E-09	1.74414E-08	14281	Fos
04921	Oxytocin signaling pathway	6.02617417	2.01666E-09	1.74414E-08	16440	Itpr3
04921	Oxytocin signaling pathway	6.02617417	2.01666E-09	1.74414E-08	108099	Prkag2
04921	Oxytocin signaling pathway	6.02617417	2.01666E-09	1.74414E-08	16518	Kcnj2
04921	Oxytocin signaling pathway	6.02617417	2.01666E-09	1.74414E-08	19735	Rgs2
04921	Oxytocin signaling pathway	6.02617417	2.01666E-09	1.74414E-08	17260	Mef2c
04921	Oxytocin signaling pathway	6.02617417	2.01666E-09	1.74414E-08	18018	Nfatc1
04921	Oxytocin signaling pathway	6.02617417	2.01666E-09	1.74414E-08	11513	Adcy7
04921	Oxytocin signaling pathway	6.02617417	2.01666E-09	1.74414E-08	16438	Itpr1
04921	Oxytocin signaling pathway	6.02617417	2.01666E-09	1.74414E-08	18796	Plcb2
04022	cGMP-PKG signaling pathway	5.610006986	2.57772E-09	2.17071E-08	11936	Fxyd2
04022	cGMP-PKG signaling pathway	5.610006986	2.57772E-09	2.17071E-08	12289	Cacna1d
04022	cGMP-PKG signaling pathway	5.610006986	2.57772E-09	2.17071E-08	18018	Nfatc1
04022	cGMP-PKG signaling pathway	5.610006986	2.57772E-09	2.17071E-08	20541	Slc8a1
04022	cGMP-PKG signaling pathway	5.610006986	2.57772E-09	2.17071E-08	19735	Rgs2
04022	cGMP-PKG signaling pathway	5.610006986	2.57772E-09	2.17071E-08	67972	Atp2b1
04022	cGMP-PKG signaling pathway	5.610006986	2.57772E-09	2.17071E-08	11549	Adra1a

04022	cGMP-PKG signaling pathway	5.610006986	2.57772E-09	2.17071E-08	208647	Creb3l2
04022	cGMP-PKG signaling pathway	5.610006986	2.57772E-09	2.17071E-08	14886	Gtf2i
04022	cGMP-PKG signaling pathway	5.610006986	2.57772E-09	2.17071E-08	16440	Itpr3
04022	cGMP-PKG signaling pathway	5.610006986	2.57772E-09	2.17071E-08	16438	Itpr1
04022	cGMP-PKG signaling pathway	5.610006986	2.57772E-09	2.17071E-08	18796	Plcb2
04022	cGMP-PKG signaling pathway	5.610006986	2.57772E-09	2.17071E-08	11555	Adrb2
04022	cGMP-PKG signaling pathway	5.610006986	2.57772E-09	2.17071E-08	17258	Mef2a
04022	cGMP-PKG signaling pathway	5.610006986	2.57772E-09	2.17071E-08	11513	Adcy7
04022	cGMP-PKG signaling pathway	5.610006986	2.57772E-09	2.17071E-08	14673	Gna12
04022	cGMP-PKG signaling pathway	5.610006986	2.57772E-09	2.17071E-08	17260	Mef2c
04022	cGMP-PKG signaling pathway	5.610006986	2.57772E-09	2.17071E-08	18576	Pde3b
04022	cGMP-PKG signaling pathway	5.610006986	2.57772E-09	2.17071E-08	18019	Nfatc2
04022	cGMP-PKG signaling pathway	5.610006986	2.57772E-09	2.17071E-08	104111	Adcy3
04062	Chemokine signaling pathway	5.307592546	2.71631E-09	2.2062E-08	18803	Plcg1
04062	Chemokine signaling pathway	5.307592546	2.71631E-09	2.2062E-08	11513	Adcy7
04062	Chemokine signaling pathway	5.307592546	2.71631E-09	2.2062E-08	22325	Vav2
04062	Chemokine signaling pathway	5.307592546	2.71631E-09	2.2062E-08	21844	Tiam1
04062	Chemokine signaling pathway	5.307592546	2.71631E-09	2.2062E-08	104111	Adcy3
04062	Chemokine signaling pathway	5.307592546	2.71631E-09	2.2062E-08	14702	Gng2
04062	Chemokine signaling pathway	5.307592546	2.71631E-09	2.2062E-08	66066	Gng11
04062	Chemokine signaling pathway	5.307592546	2.71631E-09	2.2062E-08	94176	Dock2
04062	Chemokine signaling pathway	5.307592546	2.71631E-09	2.2062E-08	109689	Arrb1
04062	Chemokine signaling pathway	5.307592546	2.71631E-09	2.2062E-08	12772	Ccr2
04062	Chemokine signaling pathway	5.307592546	2.71631E-09	2.2062E-08	14697	Gnb5
04062	Chemokine signaling pathway	5.307592546	2.71631E-09	2.2062E-08	216148	Shc2
04062	Chemokine signaling pathway	5.307592546	2.71631E-09	2.2062E-08	271849	Shc4
04062	Chemokine signaling pathway	5.307592546	2.71631E-09	2.2062E-08	12774	Ccr5
04062	Chemokine signaling pathway	5.307592546	2.71631E-09	2.2062E-08	13051	Cx3cr1
04062	Chemokine signaling pathway	5.307592546	2.71631E-09	2.2062E-08	18796	Plcb2
04062	Chemokine signaling pathway	5.307592546	2.71631E-09	2.2062E-08	18709	Pik3r2
04062	Chemokine signaling pathway	5.307592546	2.71631E-09	2.2062E-08	19395	Rasgrp2
04062	Chemokine signaling pathway	5.307592546	2.71631E-09	2.2062E-08	12766	Cxcr3
04062	Chemokine signaling pathway	5.307592546	2.71631E-09	2.2062E-08	18710	Pik3r3
04062	Chemokine signaling pathway	5.307592546	2.71631E-09	2.2062E-08	12767	Cxcr4
04144	Endocytosis	4.460161804	2.75775E-09	2.2062E-08	230837	Asap3
04144	Endocytosis	4.460161804	2.75775E-09	2.2062E-08	21812	Tgfbr1
04144	Endocytosis	4.460161804	2.75775E-09	2.2062E-08	106952	Arap3
04144	Endocytosis	4.460161804	2.75775E-09	2.2062E-08	21813	Tgfbr2
04144	Endocytosis	4.460161804	2.75775E-09	2.2062E-08	16572	Kif5a
04144	Endocytosis	4.460161804	2.75775E-09	2.2062E-08	98878	Ehd4
04144	Endocytosis	4.460161804	2.75775E-09	2.2062E-08	232227	Iqsec1
04144	Endocytosis	4.460161804	2.75775E-09	2.2062E-08	12767	Cxcr4

04144	Endocytosis	4.460161804	2.75775E-09	2.2062E-08	12774	Ccr5
04144	Endocytosis	4.460161804	2.75775E-09	2.2062E-08	69178	Snx5
04144	Endocytosis	4.460161804	2.75775E-09	2.2062E-08	212285	Arap2
04144	Endocytosis	4.460161804	2.75775E-09	2.2062E-08	17127	Smad3
04144	Endocytosis	4.460161804	2.75775E-09	2.2062E-08	107568	Wwp1
04144	Endocytosis	4.460161804	2.75775E-09	2.2062E-08	19157	Cyth1
04144	Endocytosis	4.460161804	2.75775E-09	2.2062E-08	52055	Rab11fip5
04144	Endocytosis	4.460161804	2.75775E-09	2.2062E-08	347722	Agap1
04144	Endocytosis	4.460161804	2.75775E-09	2.2062E-08	16574	Kif5c
04144	Endocytosis	4.460161804	2.75775E-09	2.2062E-08	100017	Ldlrap1
04144	Endocytosis	4.460161804	2.75775E-09	2.2062E-08	268451	Rab11fip4
04144	Endocytosis	4.460161804	2.75775E-09	2.2062E-08	109689	Arrb1
04144	Endocytosis	4.460161804	2.75775E-09	2.2062E-08	16001	Igf1r
04144	Endocytosis	4.460161804	2.75775E-09	2.2062E-08	19341	Rab4a
04144	Endocytosis	4.460161804	2.75775E-09	2.2062E-08	15511	Hspa1b
04144	Endocytosis	4.460161804	2.75775E-09	2.2062E-08	22042	Tfrc
04144	Endocytosis	4.460161804	2.75775E-09	2.2062E-08	193740	Hspa1a
05222	Small cell lung cancer	7.826864585	3.3323E-09	2.56319E-08	12443	Ccnd1
05222	Small cell lung cancer	7.826864585	3.3323E-09	2.56319E-08	12367	Casp3
05222	Small cell lung cancer	7.826864585	3.3323E-09	2.56319E-08	16403	Itga6
05222	Small cell lung cancer	7.826864585	3.3323E-09	2.56319E-08	27015	Polk
05222	Small cell lung cancer	7.826864585	3.3323E-09	2.56319E-08	20181	Rxra
05222	Small cell lung cancer	7.826864585	3.3323E-09	2.56319E-08	22032	Traf4
05222	Small cell lung cancer	7.826864585	3.3323E-09	2.56319E-08	16774	Lama3
05222	Small cell lung cancer	7.826864585	3.3323E-09	2.56319E-08	18709	Pik3r2
05222	Small cell lung cancer	7.826864585	3.3323E-09	2.56319E-08	12830	Col4a5
05222	Small cell lung cancer	7.826864585	3.3323E-09	2.56319E-08	27401	Skp2
05222	Small cell lung cancer	7.826864585	3.3323E-09	2.56319E-08	242705	E2f2
05222	Small cell lung cancer	7.826864585	3.3323E-09	2.56319E-08	23882	Gadd45g
05222	Small cell lung cancer	7.826864585	3.3323E-09	2.56319E-08	18710	Pik3r3
05222	Small cell lung cancer	7.826864585	3.3323E-09	2.56319E-08	14268	Fn1
05222	Small cell lung cancer	7.826864585	3.3323E-09	2.56319E-08	107986	Ddb2
04928	Parathyroid hormone synthesis, secretion and action	7.189120063	3.36419E-09	2.56319E-08	12393	Runx2
04928	Parathyroid hormone synthesis, secretion and action	7.189120063	3.36419E-09	2.56319E-08	16973	Lrp5
04928	Parathyroid hormone synthesis, secretion and action	7.189120063	3.36419E-09	2.56319E-08	109689	Arrb1
04928	Parathyroid hormone synthesis, secretion and action	7.189120063	3.36419E-09	2.56319E-08	17258	Mef2a
04928	Parathyroid hormone synthesis, secretion and action	7.189120063	3.36419E-09	2.56319E-08	208647	Creb3l2
04928	Parathyroid hormone synthesis, secretion and action	7.189120063	3.36419E-09	2.56319E-08	16438	Itpr1
04928	Parathyroid hormone synthesis, secretion and action	7.189120063	3.36419E-09	2.56319E-08	14281	Fos
04928	Parathyroid hormone synthesis, secretion and action	7.189120063	3.36419E-09	2.56319E-08	104111	Adcy3
04928	Parathyroid hormone synthesis, secretion and action	7.189120063	3.36419E-09	2.56319E-08	20181	Rxra
04928	Parathyroid hormone synthesis, secretion and action	7.189120063	3.36419E-09	2.56319E-08	14673	Gna12

04928	Parathyroid hormone synthesis, secretion and action	7.189120063	3.36419E-09	2.56319E-08	11513	Adcy7
04928	Parathyroid hormone synthesis, secretion and action	7.189120063	3.36419E-09	2.56319E-08	16440	Itpr3
04928	Parathyroid hormone synthesis, secretion and action	7.189120063	3.36419E-09	2.56319E-08	14182	Fgfr1
04928	Parathyroid hormone synthesis, secretion and action	7.189120063	3.36419E-09	2.56319E-08	17260	Mef2c
04928	Parathyroid hormone synthesis, secretion and action	7.189120063	3.36419E-09	2.56319E-08	18796	Plcb2
04928	Parathyroid hormone synthesis, secretion and action	7.189120063	3.36419E-09	2.56319E-08	16658	Mafb
05206	MicroRNAs in cancer	4.163995284	5.52871E-09	4.11439E-08	18803	Plcg1
05206	MicroRNAs in cancer	4.163995284	5.52871E-09	4.11439E-08	71819	Kif23
05206	MicroRNAs in cancer	4.163995284	5.52871E-09	4.11439E-08	18569	Pdcd4
05206	MicroRNAs in cancer	4.163995284	5.52871E-09	4.11439E-08	12443	Ccnd1
05206	MicroRNAs in cancer	4.163995284	5.52871E-09	4.11439E-08	15364	Hmga2
05206	MicroRNAs in cancer	4.163995284	5.52871E-09	4.11439E-08	212980	Slc45a3
05206	MicroRNAs in cancer	4.163995284	5.52871E-09	4.11439E-08	73086	Rps6ka5
05206	MicroRNAs in cancer	4.163995284	5.52871E-09	4.11439E-08	12189	Brca1
05206	MicroRNAs in cancer	4.163995284	5.52871E-09	4.11439E-08	16765	Stmn1
05206	MicroRNAs in cancer	4.163995284	5.52871E-09	4.11439E-08	12531	Cdc25b
05206	MicroRNAs in cancer	4.163995284	5.52871E-09	4.11439E-08	13433	Dnmt1
05206	MicroRNAs in cancer	4.163995284	5.52871E-09	4.11439E-08	242705	E2f2
05206	MicroRNAs in cancer	4.163995284	5.52871E-09	4.11439E-08	23805	Apc2
05206	MicroRNAs in cancer	4.163995284	5.52871E-09	4.11439E-08	13435	Dnmt3a
05206	MicroRNAs in cancer	4.163995284	5.52871E-09	4.11439E-08	18590	Pdgfa
05206	MicroRNAs in cancer	4.163995284	5.52871E-09	4.11439E-08	18710	Pik3r3
05206	MicroRNAs in cancer	4.163995284	5.52871E-09	4.11439E-08	56717	Mtor
05206	MicroRNAs in cancer	4.163995284	5.52871E-09	4.11439E-08	18709	Pik3r2
05206	MicroRNAs in cancer	4.163995284	5.52871E-09	4.11439E-08	18591	Pdgfb
05206	MicroRNAs in cancer	4.163995284	5.52871E-09	4.11439E-08	11920	Atm
05206	MicroRNAs in cancer	4.163995284	5.52871E-09	4.11439E-08	20677	Sox4
05206	MicroRNAs in cancer	4.163995284	5.52871E-09	4.11439E-08	271849	Shc4
05206	MicroRNAs in cancer	4.163995284	5.52871E-09	4.11439E-08	12367	Casp3
05206	MicroRNAs in cancer	4.163995284	5.52871E-09	4.11439E-08	171543	Bmf
05206	MicroRNAs in cancer	4.163995284	5.52871E-09	4.11439E-08	18596	Pdgfrb
05206	MicroRNAs in cancer	4.163995284	5.52871E-09	4.11439E-08	15184	Hdac5
04713	Circadian entrainment	7.427534759	7.0805E-09	5.14945E-08	18627	Per2
04713	Circadian entrainment	7.427534759	7.0805E-09	5.14945E-08	18628	Per3
04713	Circadian entrainment	7.427534759	7.0805E-09	5.14945E-08	14281	Fos
04713	Circadian entrainment	7.427534759	7.0805E-09	5.14945E-08	12289	Cacna1d
04713	Circadian entrainment	7.427534759	7.0805E-09	5.14945E-08	73086	Rps6ka5
04713	Circadian entrainment	7.427534759	7.0805E-09	5.14945E-08	12322	Camk2a
04713	Circadian entrainment	7.427534759	7.0805E-09	5.14945E-08	16440	Itpr3
04713	Circadian entrainment	7.427534759	7.0805E-09	5.14945E-08	18796	Plcb2
04713	Circadian entrainment	7.427534759	7.0805E-09	5.14945E-08	11513	Adcy7
04713	Circadian entrainment	7.427534759	7.0805E-09	5.14945E-08	18125	Nos1

04713	Circadian entrainment	7.427534759	7.0805E-09	5.14945E-08	66066	Gng11
04713	Circadian entrainment	7.427534759	7.0805E-09	5.14945E-08	16438	Itpr1
04713	Circadian entrainment	7.427534759	7.0805E-09	5.14945E-08	104111	Adcy3
04713	Circadian entrainment	7.427534759	7.0805E-09	5.14945E-08	14697	Gnb5
04713	Circadian entrainment	7.427534759	7.0805E-09	5.14945E-08	14702	Gng2
04066	HIF-1 signaling pathway	6.810745323	7.5931E-09	5.39954E-08	18803	Plcg1
04066	HIF-1 signaling pathway	6.810745323	7.5931E-09	5.39954E-08	16832	Ldhb
04066	HIF-1 signaling pathway	6.810745323	7.5931E-09	5.39954E-08	11601	Angpt2
04066	HIF-1 signaling pathway	6.810745323	7.5931E-09	5.39954E-08	56717	Mtor
04066	HIF-1 signaling pathway	6.810745323	7.5931E-09	5.39954E-08	16194	Il6ra
04066	HIF-1 signaling pathway	6.810745323	7.5931E-09	5.39954E-08	18709	Pik3r2
04066	HIF-1 signaling pathway	6.810745323	7.5931E-09	5.39954E-08	18710	Pik3r3
04066	HIF-1 signaling pathway	6.810745323	7.5931E-09	5.39954E-08	16000	Igf1
04066	HIF-1 signaling pathway	6.810745323	7.5931E-09	5.39954E-08	15979	Ifngr1
04066	HIF-1 signaling pathway	6.810745323	7.5931E-09	5.39954E-08	12322	Camk2a
04066	HIF-1 signaling pathway	6.810745323	7.5931E-09	5.39954E-08	22042	Tfrc
04066	HIF-1 signaling pathway	6.810745323	7.5931E-09	5.39954E-08	22041	Trf
04066	HIF-1 signaling pathway	6.810745323	7.5931E-09	5.39954E-08	18642	Pfkm
04066	HIF-1 signaling pathway	6.810745323	7.5931E-09	5.39954E-08	17347	Mknk2
04066	HIF-1 signaling pathway	6.810745323	7.5931E-09	5.39954E-08	226265	Eno4
04066	HIF-1 signaling pathway	6.810745323	7.5931E-09	5.39954E-08	16001	Igf1r
04142	Lysosome	6.297339902	8.20308E-09	5.70649E-08	235504	Slc17a5
04142	Lysosome	6.297339902	8.20308E-09	5.70649E-08	11881	Arsb
04142	Lysosome	6.297339902	8.20308E-09	5.70649E-08	12492	Scarb2
04142	Lysosome	6.297339902	8.20308E-09	5.70649E-08	16889	Lipa
04142	Lysosome	6.297339902	8.20308E-09	5.70649E-08	17159	Man2b1
04142	Lysosome	6.297339902	8.20308E-09	5.70649E-08	110173	Manba
04142	Lysosome	6.297339902	8.20308E-09	5.70649E-08	13034	Ctse
04142	Lysosome	6.297339902	8.20308E-09	5.70649E-08	108012	Ap1s2
04142	Lysosome	6.297339902	8.20308E-09	5.70649E-08	19141	Lgmn
04142	Lysosome	6.297339902	8.20308E-09	5.70649E-08	56464	Ctsf
04142	Lysosome	6.297339902	8.20308E-09	5.70649E-08	242341	Atp6v0d2
04142	Lysosome	6.297339902	8.20308E-09	5.70649E-08	27029	Sgsh
04142	Lysosome	6.297339902	8.20308E-09	5.70649E-08	14420	Galc
04142	Lysosome	6.297339902	8.20308E-09	5.70649E-08	15586	Hyal1
04142	Lysosome	6.297339902	8.20308E-09	5.70649E-08	192654	Pla2g15
04142	Lysosome	6.297339902	8.20308E-09	5.70649E-08	11764	Ap1b1
04142	Lysosome	6.297339902	8.20308E-09	5.70649E-08	14667	Gm2a
05223	Non-small cell lung cancer	8.761740077	1.05535E-08	7.18535E-08	107986	Ddb2
05223	Non-small cell lung cancer	8.761740077	1.05535E-08	7.18535E-08	18709	Pik3r2
05223	Non-small cell lung cancer	8.761740077	1.05535E-08	7.18535E-08	23882	Gadd45g
05223	Non-small cell lung cancer	8.761740077	1.05535E-08	7.18535E-08	12443	Ccnd1

05223	Non-small cell lung cancer	8.761740077	1.05535E-08	7.18535E-08	20181	Rxra
05223	Non-small cell lung cancer	8.761740077	1.05535E-08	7.18535E-08	242705	E2f2
05223	Non-small cell lung cancer	8.761740077	1.05535E-08	7.18535E-08	18803	Plcg1
05223	Non-small cell lung cancer	8.761740077	1.05535E-08	7.18535E-08	16572	Kif5a
05223	Non-small cell lung cancer	8.761740077	1.05535E-08	7.18535E-08	27015	Polk
05223	Non-small cell lung cancer	8.761740077	1.05535E-08	7.18535E-08	54354	Rassf5
05223	Non-small cell lung cancer	8.761740077	1.05535E-08	7.18535E-08	18710	Pik3r3
05223	Non-small cell lung cancer	8.761740077	1.05535E-08	7.18535E-08	15234	Hgf
05223	Non-small cell lung cancer	8.761740077	1.05535E-08	7.18535E-08	16574	Kif5c
04060	Cytokine-cytokine receptor interaction	4.140491504	1.27647E-08	8.50978E-08	21949	Tnfsf8
04060	Cytokine-cytokine receptor interaction	4.140491504	1.27647E-08	8.50978E-08	16195	Il6st
04060	Cytokine-cytokine receptor interaction	4.140491504	1.27647E-08	8.50978E-08	15979	Ifngr1
04060	Cytokine-cytokine receptor interaction	4.140491504	1.27647E-08	8.50978E-08	16880	Lifr
04060	Cytokine-cytokine receptor interaction	4.140491504	1.27647E-08	8.50978E-08	21813	Tgfbr2
04060	Cytokine-cytokine receptor interaction	4.140491504	1.27647E-08	8.50978E-08	12766	Cxcr3
04060	Cytokine-cytokine receptor interaction	4.140491504	1.27647E-08	8.50978E-08	16162	Il12rb2
04060	Cytokine-cytokine receptor interaction	4.140491504	1.27647E-08	8.50978E-08	16174	Il18rap
04060	Cytokine-cytokine receptor interaction	4.140491504	1.27647E-08	8.50978E-08	94185	Tnfrsf21
04060	Cytokine-cytokine receptor interaction	4.140491504	1.27647E-08	8.50978E-08	245527	Eda2r
04060	Cytokine-cytokine receptor interaction	4.140491504	1.27647E-08	8.50978E-08	24099	Tnfsf13b
04060	Cytokine-cytokine receptor interaction	4.140491504	1.27647E-08	8.50978E-08	69583	Tnfsf13
04060	Cytokine-cytokine receptor interaction	4.140491504	1.27647E-08	8.50978E-08	11481	Acvr2b
04060	Cytokine-cytokine receptor interaction	4.140491504	1.27647E-08	8.50978E-08	16194	Il6ra
04060	Cytokine-cytokine receptor interaction	4.140491504	1.27647E-08	8.50978E-08	21944	Tnfsf12
04060	Cytokine-cytokine receptor interaction	4.140491504	1.27647E-08	8.50978E-08	21812	Tgfbr1
04060	Cytokine-cytokine receptor interaction	4.140491504	1.27647E-08	8.50978E-08	16170	Il16
04060	Cytokine-cytokine receptor interaction	4.140491504	1.27647E-08	8.50978E-08	12767	Cxcr4
04060	Cytokine-cytokine receptor interaction	4.140491504	1.27647E-08	8.50978E-08	13051	Cx3cr1
04060	Cytokine-cytokine receptor interaction	4.140491504	1.27647E-08	8.50978E-08	14562	Gdf3
04060	Cytokine-cytokine receptor interaction	4.140491504	1.27647E-08	8.50978E-08	50931	Il27ra
04060	Cytokine-cytokine receptor interaction	4.140491504	1.27647E-08	8.50978E-08	12774	Ccr5
04060	Cytokine-cytokine receptor interaction	4.140491504	1.27647E-08	8.50978E-08	12504	Cd4
04060	Cytokine-cytokine receptor interaction	4.140491504	1.27647E-08	8.50978E-08	12772	Ccr2
04060	Cytokine-cytokine receptor interaction	4.140491504	1.27647E-08	8.50978E-08	17082	Il1rl1
04922	Glucagon signaling pathway	6.999023138	1.6477E-08	1.07605E-07	16438	Itpr1
04922	Glucagon signaling pathway	6.999023138	1.6477E-08	1.07605E-07	12322	Camk2a
04922	Glucagon signaling pathway	6.999023138	1.6477E-08	1.07605E-07	18576	Pde3b
04922	Glucagon signaling pathway	6.999023138	1.6477E-08	1.07605E-07	17691	Sik1
04922	Glucagon signaling pathway	6.999023138	1.6477E-08	1.07605E-07	74551	Pck2
04922	Glucagon signaling pathway	6.999023138	1.6477E-08	1.07605E-07	208647	Creb3l2
04922	Glucagon signaling pathway	6.999023138	1.6477E-08	1.07605E-07	12894	Cpt1a
04922	Glucagon signaling pathway	6.999023138	1.6477E-08	1.07605E-07	108099	Prkag2

04922	Glucagon signaling pathway	6.999023138	1.6477E-08	1.07605E-07	16440	Itpr3
04922	Glucagon signaling pathway	6.999023138	1.6477E-08	1.07605E-07	18796	Plcb2
04922	Glucagon signaling pathway	6.999023138	1.6477E-08	1.07605E-07	16832	Ldhb
04922	Glucagon signaling pathway	6.999023138	1.6477E-08	1.07605E-07	18642	Pfkm
04922	Glucagon signaling pathway	6.999023138	1.6477E-08	1.07605E-07	110094	Phka2
04922	Glucagon signaling pathway	6.999023138	1.6477E-08	1.07605E-07	100705	Acacb
04922	Glucagon signaling pathway	6.999023138	1.6477E-08	1.07605E-07	110078	Pygb
05163	Human cytomegalovirus infection	4.359808163	2.02089E-08	1.29337E-07	242705	E2f2
05163	Human cytomegalovirus infection	4.359808163	2.02089E-08	1.29337E-07	26399	Map2k6
05163	Human cytomegalovirus infection	4.359808163	2.02089E-08	1.29337E-07	208647	Creb3l2
05163	Human cytomegalovirus infection	4.359808163	2.02089E-08	1.29337E-07	11513	Adcy7
05163	Human cytomegalovirus infection	4.359808163	2.02089E-08	1.29337E-07	18796	Plcb2
05163	Human cytomegalovirus infection	4.359808163	2.02089E-08	1.29337E-07	18019	Nfatc2
05163	Human cytomegalovirus infection	4.359808163	2.02089E-08	1.29337E-07	56717	Mtor
05163	Human cytomegalovirus infection	4.359808163	2.02089E-08	1.29337E-07	12767	Cxcr4
05163	Human cytomegalovirus infection	4.359808163	2.02089E-08	1.29337E-07	16438	Itpr1
05163	Human cytomegalovirus infection	4.359808163	2.02089E-08	1.29337E-07	66066	Gng11
05163	Human cytomegalovirus infection	4.359808163	2.02089E-08	1.29337E-07	12443	Ccnd1
05163	Human cytomegalovirus infection	4.359808163	2.02089E-08	1.29337E-07	104111	Adcy3
05163	Human cytomegalovirus infection	4.359808163	2.02089E-08	1.29337E-07	18709	Pik3r2
05163	Human cytomegalovirus infection	4.359808163	2.02089E-08	1.29337E-07	16440	Itpr3
05163	Human cytomegalovirus infection	4.359808163	2.02089E-08	1.29337E-07	14702	Gng2
05163	Human cytomegalovirus infection	4.359808163	2.02089E-08	1.29337E-07	14673	Gna12
05163	Human cytomegalovirus infection	4.359808163	2.02089E-08	1.29337E-07	19218	Ptger3
05163	Human cytomegalovirus infection	4.359808163	2.02089E-08	1.29337E-07	18018	Nfatc1
05163	Human cytomegalovirus infection	4.359808163	2.02089E-08	1.29337E-07	18710	Pik3r3
05163	Human cytomegalovirus infection	4.359808163	2.02089E-08	1.29337E-07	12367	Casp3
05163	Human cytomegalovirus infection	4.359808163	2.02089E-08	1.29337E-07	16194	Il6ra
05163	Human cytomegalovirus infection	4.359808163	2.02089E-08	1.29337E-07	12774	Ccr5
05163	Human cytomegalovirus infection	4.359808163	2.02089E-08	1.29337E-07	14697	Gnb5
05170	Human immunodeficiency virus 1 infection	4.448268039	2.99303E-08	1.87798E-07	16438	Itpr1
05170	Human immunodeficiency virus 1 infection	4.448268039	2.99303E-08	1.87798E-07	108012	Ap1s2
05170	Human immunodeficiency virus 1 infection	4.448268039	2.99303E-08	1.87798E-07	12504	Cd4
05170	Human immunodeficiency virus 1 infection	4.448268039	2.99303E-08	1.87798E-07	14281	Fos
05170	Human immunodeficiency virus 1 infection	4.448268039	2.99303E-08	1.87798E-07	12767	Cxcr4
05170	Human immunodeficiency virus 1 infection	4.448268039	2.99303E-08	1.87798E-07	18709	Pik3r2
05170	Human immunodeficiency virus 1 infection	4.448268039	2.99303E-08	1.87798E-07	12774	Ccr5
05170	Human immunodeficiency virus 1 infection	4.448268039	2.99303E-08	1.87798E-07	56717	Mtor
05170	Human immunodeficiency virus 1 infection	4.448268039	2.99303E-08	1.87798E-07	16440	Itpr3
05170	Human immunodeficiency virus 1 infection	4.448268039	2.99303E-08	1.87798E-07	11920	Atm
05170	Human immunodeficiency virus 1 infection	4.448268039	2.99303E-08	1.87798E-07	12442	Ccnb2
05170	Human immunodeficiency virus 1 infection	4.448268039	2.99303E-08	1.87798E-07	11764	Ap1b1

05170	Human immunodeficiency virus 1 infection	4.448268039	2.99303E-08	1.87798E-07	22390	Wee1
05170	Human immunodeficiency virus 1 infection	4.448268039	2.99303E-08	1.87798E-07	66066	Gng11
05170	Human immunodeficiency virus 1 infection	4.448268039	2.99303E-08	1.87798E-07	18018	Nfatc1
05170	Human immunodeficiency virus 1 infection	4.448268039	2.99303E-08	1.87798E-07	26399	Map2k6
05170	Human immunodeficiency virus 1 infection	4.448268039	2.99303E-08	1.87798E-07	18710	Pik3r3
05170	Human immunodeficiency virus 1 infection	4.448268039	2.99303E-08	1.87798E-07	18019	Nfatc2
05170	Human immunodeficiency virus 1 infection	4.448268039	2.99303E-08	1.87798E-07	14702	Gng2
05170	Human immunodeficiency virus 1 infection	4.448268039	2.99303E-08	1.87798E-07	18803	Plcg1
05170	Human immunodeficiency virus 1 infection	4.448268039	2.99303E-08	1.87798E-07	12367	Casp3
05170	Human immunodeficiency virus 1 infection	4.448268039	2.99303E-08	1.87798E-07	14697	Gnb5
01522	Endocrine resistance	7.305073612	3.0594E-08	1.88271E-07	12580	Cdkn2c
01522	Endocrine resistance	7.305073612	3.0594E-08	1.88271E-07	17979	Ncoa3
01522	Endocrine resistance	7.305073612	3.0594E-08	1.88271E-07	14281	Fos
01522	Endocrine resistance	7.305073612	3.0594E-08	1.88271E-07	16001	Igf1r
01522	Endocrine resistance	7.305073612	3.0594E-08	1.88271E-07	12443	Ccnd1
01522	Endocrine resistance	7.305073612	3.0594E-08	1.88271E-07	16000	Igf1
01522	Endocrine resistance	7.305073612	3.0594E-08	1.88271E-07	216148	Shc2
01522	Endocrine resistance	7.305073612	3.0594E-08	1.88271E-07	11513	Adcy7
01522	Endocrine resistance	7.305073612	3.0594E-08	1.88271E-07	242705	E2f2
01522	Endocrine resistance	7.305073612	3.0594E-08	1.88271E-07	56717	Mtor
01522	Endocrine resistance	7.305073612	3.0594E-08	1.88271E-07	104111	Adcy3
01522	Endocrine resistance	7.305073612	3.0594E-08	1.88271E-07	271849	Shc4
01522	Endocrine resistance	7.305073612	3.0594E-08	1.88271E-07	18710	Pik3r3
01522	Endocrine resistance	7.305073612	3.0594E-08	1.88271E-07	18709	Pik3r2
04360	Axon guidance	5.093948332	3.4411E-08	2.07764E-07	11854	Rhod
04360	Axon guidance	5.093948332	3.4411E-08	2.07764E-07	13848	Ephb6
04360	Axon guidance	5.093948332	3.4411E-08	2.07764E-07	18710	Pik3r3
04360	Axon guidance	5.093948332	3.4411E-08	2.07764E-07	18709	Pik3r2
04360	Axon guidance	5.093948332	3.4411E-08	2.07764E-07	12322	Camk2a
04360	Axon guidance	5.093948332	3.4411E-08	2.07764E-07	20562	Slit1
04360	Axon guidance	5.093948332	3.4411E-08	2.07764E-07	18019	Nfatc2
04360	Axon guidance	5.093948332	3.4411E-08	2.07764E-07	22066	Trpc4
04360	Axon guidance	5.093948332	3.4411E-08	2.07764E-07	259302	Srgap3
04360	Axon guidance	5.093948332	3.4411E-08	2.07764E-07	18007	Neo1
04360	Axon guidance	5.093948332	3.4411E-08	2.07764E-07	12767	Cxcr4
04360	Axon guidance	5.093948332	3.4411E-08	2.07764E-07	54712	Plxnc1
04360	Axon guidance	5.093948332	3.4411E-08	2.07764E-07	140570	Plxnb2
04360	Axon guidance	5.093948332	3.4411E-08	2.07764E-07	16728	L1cam
04360	Axon guidance	5.093948332	3.4411E-08	2.07764E-07	18803	Plcg1
04360	Axon guidance	5.093948332	3.4411E-08	2.07764E-07	17974	Nck2
04360	Axon guidance	5.093948332	3.4411E-08	2.07764E-07	237860	Ssh2
04360	Axon guidance	5.093948332	3.4411E-08	2.07764E-07	18846	Plxna3

04360	Axon guidance	5.093948332	3.4411E-08	2.07764E-07	140571	Plxnb3
04640	Hematopoietic cell lineage	7.151282589	4.05797E-08	2.40472E-07	14960	H2-Aa
04640	Hematopoietic cell lineage	7.151282589	4.05797E-08	2.40472E-07	14723	Gp1ba
04640	Hematopoietic cell lineage	7.151282589	4.05797E-08	2.40472E-07	22042	Tfrc
04640	Hematopoietic cell lineage	7.151282589	4.05797E-08	2.40472E-07	12504	Cd4
04640	Hematopoietic cell lineage	7.151282589	4.05797E-08	2.40472E-07	14999	H2-DMb1
04640	Hematopoietic cell lineage	7.151282589	4.05797E-08	2.40472E-07	17311	Kitl
04640	Hematopoietic cell lineage	7.151282589	4.05797E-08	2.40472E-07	12481	Cd2
04640	Hematopoietic cell lineage	7.151282589	4.05797E-08	2.40472E-07	14961	H2-Ab1
04640	Hematopoietic cell lineage	7.151282589	4.05797E-08	2.40472E-07	12490	Cd34
04640	Hematopoietic cell lineage	7.151282589	4.05797E-08	2.40472E-07	16403	Itga6
04640	Hematopoietic cell lineage	7.151282589	4.05797E-08	2.40472E-07	14998	H2-DMa
04640	Hematopoietic cell lineage	7.151282589	4.05797E-08	2.40472E-07	16194	Il6ra
04640	Hematopoietic cell lineage	7.151282589	4.05797E-08	2.40472E-07	14256	Flt3l
04640	Hematopoietic cell lineage	7.151282589	4.05797E-08	2.40472E-07	12483	Cd22
04672	Intestinal immune network for IgA production	11.28524661	5.54144E-08	3.22411E-07	14960	H2-Aa
04672	Intestinal immune network for IgA production	11.28524661	5.54144E-08	3.22411E-07	69583	Tnfsf13
04672	Intestinal immune network for IgA production	11.28524661	5.54144E-08	3.22411E-07	12487	Cd28
04672	Intestinal immune network for IgA production	11.28524661	5.54144E-08	3.22411E-07	54167	Icos
04672	Intestinal immune network for IgA production	11.28524661	5.54144E-08	3.22411E-07	16421	Itgb7
04672	Intestinal immune network for IgA production	11.28524661	5.54144E-08	3.22411E-07	12767	Cxcr4
04672	Intestinal immune network for IgA production	11.28524661	5.54144E-08	3.22411E-07	14961	H2-Ab1
04672	Intestinal immune network for IgA production	11.28524661	5.54144E-08	3.22411E-07	14999	H2-DMb1
04672	Intestinal immune network for IgA production	11.28524661	5.54144E-08	3.22411E-07	24099	Tnfsf13b
04672	Intestinal immune network for IgA production	11.28524661	5.54144E-08	3.22411E-07	14998	H2-DMa
05215	Prostate cancer	6.862341878	6.98916E-08	3.9938E-07	18590	Pdgfa
05215	Prostate cancer	6.862341878	6.98916E-08	3.9938E-07	16000	Igf1
05215	Prostate cancer	6.862341878	6.98916E-08	3.9938E-07	12443	Ccnd1
05215	Prostate cancer	6.862341878	6.98916E-08	3.9938E-07	56717	Mtor
05215	Prostate cancer	6.862341878	6.98916E-08	3.9938E-07	18591	Pdgfb
05215	Prostate cancer	6.862341878	6.98916E-08	3.9938E-07	208647	Creb3l2
05215	Prostate cancer	6.862341878	6.98916E-08	3.9938E-07	16842	Lef1
05215	Prostate cancer	6.862341878	6.98916E-08	3.9938E-07	54635	Pdgfc
05215	Prostate cancer	6.862341878	6.98916E-08	3.9938E-07	18596	Pdgfrb
05215	Prostate cancer	6.862341878	6.98916E-08	3.9938E-07	18710	Pik3r3
05215	Prostate cancer	6.862341878	6.98916E-08	3.9938E-07	16001	Igf1r
05215	Prostate cancer	6.862341878	6.98916E-08	3.9938E-07	14182	Fgfr1
05215	Prostate cancer	6.862341878	6.98916E-08	3.9938E-07	242705	E2f2
05215	Prostate cancer	6.862341878	6.98916E-08	3.9938E-07	18709	Pik3r2
04728	Dopaminergic synapse	5.75129605	9.06629E-08	5.01021E-07	14281	Fos
04728	Dopaminergic synapse	5.75129605	9.06629E-08	5.01021E-07	14697	Gnb5
04728	Dopaminergic synapse	5.75129605	9.06629E-08	5.01021E-07	12846	Comt

04728	Dopaminergic synapse	5.75129605	9.06629E-08	5.01021E-07	12322	Camk2a
04728	Dopaminergic synapse	5.75129605	9.06629E-08	5.01021E-07	66066	Gng11
04728	Dopaminergic synapse	5.75129605	9.06629E-08	5.01021E-07	109689	Arrb1
04728	Dopaminergic synapse	5.75129605	9.06629E-08	5.01021E-07	12286	Cacna1a
04728	Dopaminergic synapse	5.75129605	9.06629E-08	5.01021E-07	14702	Gng2
04728	Dopaminergic synapse	5.75129605	9.06629E-08	5.01021E-07	18796	Plcb2
04728	Dopaminergic synapse	5.75129605	9.06629E-08	5.01021E-07	12287	Cacna1b
04728	Dopaminergic synapse	5.75129605	9.06629E-08	5.01021E-07	16574	Kif5c
04728	Dopaminergic synapse	5.75129605	9.06629E-08	5.01021E-07	16572	Kif5a
04728	Dopaminergic synapse	5.75129605	9.06629E-08	5.01021E-07	16438	Itpr1
04728	Dopaminergic synapse	5.75129605	9.06629E-08	5.01021E-07	16440	Itpr3
04728	Dopaminergic synapse	5.75129605	9.06629E-08	5.01021E-07	12289	Cacna1d
04728	Dopaminergic synapse	5.75129605	9.06629E-08	5.01021E-07	208647	Creb3l2
04933	AGE-RAGE signaling pathway in diabetic complications	6.72645392	9.08101E-08	5.01021E-07	22340	Vegfb
04933	AGE-RAGE signaling pathway in diabetic complications	6.72645392	9.08101E-08	5.01021E-07	18018	Nfatc1
04933	AGE-RAGE signaling pathway in diabetic complications	6.72645392	9.08101E-08	5.01021E-07	18710	Pik3r3
04933	AGE-RAGE signaling pathway in diabetic complications	6.72645392	9.08101E-08	5.01021E-07	18709	Pik3r2
04933	AGE-RAGE signaling pathway in diabetic complications	6.72645392	9.08101E-08	5.01021E-07	18796	Plcb2
04933	AGE-RAGE signaling pathway in diabetic complications	6.72645392	9.08101E-08	5.01021E-07	12443	Ccnd1
04933	AGE-RAGE signaling pathway in diabetic complications	6.72645392	9.08101E-08	5.01021E-07	17127	Smad3
04933	AGE-RAGE signaling pathway in diabetic complications	6.72645392	9.08101E-08	5.01021E-07	21812	Tgfbr1
04933	AGE-RAGE signaling pathway in diabetic complications	6.72645392	9.08101E-08	5.01021E-07	12830	Col4a5
04933	AGE-RAGE signaling pathway in diabetic complications	6.72645392	9.08101E-08	5.01021E-07	12367	Casp3
04933	AGE-RAGE signaling pathway in diabetic complications	6.72645392	9.08101E-08	5.01021E-07	21813	Tgfbr2
04933	AGE-RAGE signaling pathway in diabetic complications	6.72645392	9.08101E-08	5.01021E-07	21824	Thbd
04933	AGE-RAGE signaling pathway in diabetic complications	6.72645392	9.08101E-08	5.01021E-07	18803	Plcg1
04933	AGE-RAGE signaling pathway in diabetic complications	6.72645392	9.08101E-08	5.01021E-07	14268	Fn1
04660	T cell receptor signaling pathway	6.595843165	1.17249E-07	6.35924E-07	18710	Pik3r3
04660	T cell receptor signaling pathway	6.595843165	1.17249E-07	6.35924E-07	12487	Cd28
04660	T cell receptor signaling pathway	6.595843165	1.17249E-07	6.35924E-07	18018	Nfatc1
04660	T cell receptor signaling pathway	6.595843165	1.17249E-07	6.35924E-07	54167	Icos
04660	T cell receptor signaling pathway	6.595843165	1.17249E-07	6.35924E-07	12504	Cd4
04660	T cell receptor signaling pathway	6.595843165	1.17249E-07	6.35924E-07	21682	Tec
04660	T cell receptor signaling pathway	6.595843165	1.17249E-07	6.35924E-07	18019	Nfatc2
04660	T cell receptor signaling pathway	6.595843165	1.17249E-07	6.35924E-07	18709	Pik3r2
04660	T cell receptor signaling pathway	6.595843165	1.17249E-07	6.35924E-07	14281	Fos
04660	T cell receptor signaling pathway	6.595843165	1.17249E-07	6.35924E-07	22325	Vav2
04660	T cell receptor signaling pathway	6.595843165	1.17249E-07	6.35924E-07	18803	Plcg1
04660	T cell receptor signaling pathway	6.595843165	1.17249E-07	6.35924E-07	108723	Card11
04660	T cell receptor signaling pathway	6.595843165	1.17249E-07	6.35924E-07	17444	Grap2
04660	T cell receptor signaling pathway	6.595843165	1.17249E-07	6.35924E-07	17974	Nck2
04650	Natural killer cell mediated cytotoxicity	6.015689309	1.34604E-07	7.17887E-07	18018	Nfatc1

04650	Natural killer cell mediated cytotoxicity	6.015689309	1.34604E-07	7.17887E-07	16627	Klra1
04650	Natural killer cell mediated cytotoxicity	6.015689309	1.34604E-07	7.17887E-07	77777	Ulbp1
04650	Natural killer cell mediated cytotoxicity	6.015689309	1.34604E-07	7.17887E-07	18803	Plcg1
04650	Natural killer cell mediated cytotoxicity	6.015689309	1.34604E-07	7.17887E-07	15979	Ifngr1
04650	Natural killer cell mediated cytotoxicity	6.015689309	1.34604E-07	7.17887E-07	18019	Nfatc2
04650	Natural killer cell mediated cytotoxicity	6.015689309	1.34604E-07	7.17887E-07	12367	Casp3
04650	Natural killer cell mediated cytotoxicity	6.015689309	1.34604E-07	7.17887E-07	22325	Vav2
04650	Natural killer cell mediated cytotoxicity	6.015689309	1.34604E-07	7.17887E-07	18709	Pik3r2
04650	Natural killer cell mediated cytotoxicity	6.015689309	1.34604E-07	7.17887E-07	271849	Shc4
04650	Natural killer cell mediated cytotoxicity	6.015689309	1.34604E-07	7.17887E-07	26904	Sh2d1b1
04650	Natural killer cell mediated cytotoxicity	6.015689309	1.34604E-07	7.17887E-07	216148	Shc2
04650	Natural killer cell mediated cytotoxicity	6.015689309	1.34604E-07	7.17887E-07	16635	Klra4
04650	Natural killer cell mediated cytotoxicity	6.015689309	1.34604E-07	7.17887E-07	18710	Pik3r3
04650	Natural killer cell mediated cytotoxicity	6.015689309	1.34604E-07	7.17887E-07	17059	Klrb1c
03320	PPAR signaling pathway	7.088149276	1.51415E-07	7.94307E-07	20181	Rxra
03320	PPAR signaling pathway	7.088149276	1.51415E-07	7.94307E-07	12140	Fabp7
03320	PPAR signaling pathway	7.088149276	1.51415E-07	7.94307E-07	80911	Acox3
03320	PPAR signaling pathway	7.088149276	1.51415E-07	7.94307E-07	12894	Cpt1a
03320	PPAR signaling pathway	7.088149276	1.51415E-07	7.94307E-07	57875	Angptl4
03320	PPAR signaling pathway	7.088149276	1.51415E-07	7.94307E-07	74551	Pck2
03320	PPAR signaling pathway	7.088149276	1.51415E-07	7.94307E-07	11520	Plin2
03320	PPAR signaling pathway	7.088149276	1.51415E-07	7.94307E-07	19016	Pparg
03320	PPAR signaling pathway	7.088149276	1.51415E-07	7.94307E-07	16592	Fabp5
03320	PPAR signaling pathway	7.088149276	1.51415E-07	7.94307E-07	104086	Cyp27a1
03320	PPAR signaling pathway	7.088149276	1.51415E-07	7.94307E-07	11770	Fabp4
03320	PPAR signaling pathway	7.088149276	1.51415E-07	7.94307E-07	225579	Slc27a6
03320	PPAR signaling pathway	7.088149276	1.51415E-07	7.94307E-07	18830	Pltp
04211	Longevity regulating pathway	7.009392061	1.73645E-07	8.82005E-07	75747	Sesn3
04211	Longevity regulating pathway	7.009392061	1.73645E-07	8.82005E-07	108099	Prkag2
04211	Longevity regulating pathway	7.009392061	1.73645E-07	8.82005E-07	104111	Adcy3
04211	Longevity regulating pathway	7.009392061	1.73645E-07	8.82005E-07	19016	Pparg
04211	Longevity regulating pathway	7.009392061	1.73645E-07	8.82005E-07	140742	Sesn1
04211	Longevity regulating pathway	7.009392061	1.73645E-07	8.82005E-07	11513	Adcy7
04211	Longevity regulating pathway	7.009392061	1.73645E-07	8.82005E-07	16000	Igf1
04211	Longevity regulating pathway	7.009392061	1.73645E-07	8.82005E-07	56717	Mtor
04211	Longevity regulating pathway	7.009392061	1.73645E-07	8.82005E-07	22241	Ulk1
04211	Longevity regulating pathway	7.009392061	1.73645E-07	8.82005E-07	18709	Pik3r2
04211	Longevity regulating pathway	7.009392061	1.73645E-07	8.82005E-07	18710	Pik3r3
04211	Longevity regulating pathway	7.009392061	1.73645E-07	8.82005E-07	208647	Creb3l2
04211	Longevity regulating pathway	7.009392061	1.73645E-07	8.82005E-07	16001	Igf1r
04914	Progesterone-mediated oocyte maturation	7.009392061	1.73645E-07	8.82005E-07	12531	Cdc25b
04914	Progesterone-mediated oocyte maturation	7.009392061	1.73645E-07	8.82005E-07	208922	Cpeb3

04914	Progesterone-mediated oocyte maturation	7.009392061	1.73645E-07	8.82005E-07	18817	Plk1
04914	Progesterone-mediated oocyte maturation	7.009392061	1.73645E-07	8.82005E-07	16000	Igf1
04914	Progesterone-mediated oocyte maturation	7.009392061	1.73645E-07	8.82005E-07	12877	Cpeb1
04914	Progesterone-mediated oocyte maturation	7.009392061	1.73645E-07	8.82005E-07	18576	Pde3b
04914	Progesterone-mediated oocyte maturation	7.009392061	1.73645E-07	8.82005E-07	18710	Pik3r3
04914	Progesterone-mediated oocyte maturation	7.009392061	1.73645E-07	8.82005E-07	16001	Igf1r
04914	Progesterone-mediated oocyte maturation	7.009392061	1.73645E-07	8.82005E-07	12428	Ccna2
04914	Progesterone-mediated oocyte maturation	7.009392061	1.73645E-07	8.82005E-07	104111	Adcy3
04914	Progesterone-mediated oocyte maturation	7.009392061	1.73645E-07	8.82005E-07	18709	Pik3r2
04914	Progesterone-mediated oocyte maturation	7.009392061	1.73645E-07	8.82005E-07	12442	Ccnb2
04914	Progesterone-mediated oocyte maturation	7.009392061	1.73645E-07	8.82005E-07	11513	Adcy7
05212	Pancreatic cancer	7.662088488	2.06776E-07	1.00638E-06	23882	Gadd45g
05212	Pancreatic cancer	7.662088488	2.06776E-07	1.00638E-06	21813	Tgfbr2
05212	Pancreatic cancer	7.662088488	2.06776E-07	1.00638E-06	12190	Brca2
05212	Pancreatic cancer	7.662088488	2.06776E-07	1.00638E-06	12443	Ccnd1
05212	Pancreatic cancer	7.662088488	2.06776E-07	1.00638E-06	17127	Smad3
05212	Pancreatic cancer	7.662088488	2.06776E-07	1.00638E-06	18710	Pik3r3
05212	Pancreatic cancer	7.662088488	2.06776E-07	1.00638E-06	21812	Tgfbr1
05212	Pancreatic cancer	7.662088488	2.06776E-07	1.00638E-06	242705	E2f2
05212	Pancreatic cancer	7.662088488	2.06776E-07	1.00638E-06	56717	Mtor
05212	Pancreatic cancer	7.662088488	2.06776E-07	1.00638E-06	107986	Ddb2
05212	Pancreatic cancer	7.662088488	2.06776E-07	1.00638E-06	27015	Polk
05212	Pancreatic cancer	7.662088488	2.06776E-07	1.00638E-06	18709	Pik3r2
05220	Chronic myeloid leukemia	7.662088488	2.06776E-07	1.00638E-06	242705	E2f2
05220	Chronic myeloid leukemia	7.662088488	2.06776E-07	1.00638E-06	18709	Pik3r2
05220	Chronic myeloid leukemia	7.662088488	2.06776E-07	1.00638E-06	18710	Pik3r3
05220	Chronic myeloid leukemia	7.662088488	2.06776E-07	1.00638E-06	27015	Polk
05220	Chronic myeloid leukemia	7.662088488	2.06776E-07	1.00638E-06	271849	Shc4
05220	Chronic myeloid leukemia	7.662088488	2.06776E-07	1.00638E-06	21813	Tgfbr2
05220	Chronic myeloid leukemia	7.662088488	2.06776E-07	1.00638E-06	17127	Smad3
05220	Chronic myeloid leukemia	7.662088488	2.06776E-07	1.00638E-06	107986	Ddb2
05220	Chronic myeloid leukemia	7.662088488	2.06776E-07	1.00638E-06	23882	Gadd45g
05220	Chronic myeloid leukemia	7.662088488	2.06776E-07	1.00638E-06	12443	Ccnd1
05220	Chronic myeloid leukemia	7.662088488	2.06776E-07	1.00638E-06	21812	Tgfbr1
05220	Chronic myeloid leukemia	7.662088488	2.06776E-07	1.00638E-06	216148	Shc2
04979	Cholesterol metabolism	9.903379679	2.12322E-07	1.00638E-06	16971	Lrp1
04979	Cholesterol metabolism	9.903379679	2.12322E-07	1.00638E-06	104086	Cyp27a1
04979	Cholesterol metabolism	9.903379679	2.12322E-07	1.00638E-06	218203	Mylip
04979	Cholesterol metabolism	9.903379679	2.12322E-07	1.00638E-06	79196	Osbpl5
04979	Cholesterol metabolism	9.903379679	2.12322E-07	1.00638E-06	320024	Nceh1
04979	Cholesterol metabolism	9.903379679	2.12322E-07	1.00638E-06	18830	Pltp
04979	Cholesterol metabolism	9.903379679	2.12322E-07	1.00638E-06	11813	Apoc2

04979	Cholesterol metabolism	9.903379679	2.12322E-07	1.00638E-06	100017	Ldlrap1
04979	Cholesterol metabolism	9.903379679	2.12322E-07	1.00638E-06	16889	Lipa
04979	Cholesterol metabolism	9.903379679	2.12322E-07	1.00638E-06	57875	Angptl4
05321	Inflammatory bowel disease	8.609551043	2.13209E-07	1.00638E-06	18018	Nfatc1
05321	Inflammatory bowel disease	8.609551043	2.13209E-07	1.00638E-06	16174	Il18rap
05321	Inflammatory bowel disease	8.609551043	2.13209E-07	1.00638E-06	15979	Ifngr1
05321	Inflammatory bowel disease	8.609551043	2.13209E-07	1.00638E-06	16162	Il12rb2
05321	Inflammatory bowel disease	8.609551043	2.13209E-07	1.00638E-06	53791	Tlr5
05321	Inflammatory bowel disease	8.609551043	2.13209E-07	1.00638E-06	17132	Maf
05321	Inflammatory bowel disease	8.609551043	2.13209E-07	1.00638E-06	14999	H2-DMb1
05321	Inflammatory bowel disease	8.609551043	2.13209E-07	1.00638E-06	14998	H2-DMa
05321	Inflammatory bowel disease	8.609551043	2.13209E-07	1.00638E-06	14961	H2-Ab1
05321	Inflammatory bowel disease	8.609551043	2.13209E-07	1.00638E-06	14960	H2-Aa
05321	Inflammatory bowel disease	8.609551043	2.13209E-07	1.00638E-06	17127	Smad3
05165	Human papillomavirus infection	3.485333069	2.13856E-07	1.00638E-06	17305	Mfng
05165	Human papillomavirus infection	3.485333069	2.13856E-07	1.00638E-06	15213	Hey1
05165	Human papillomavirus infection	3.485333069	2.13856E-07	1.00638E-06	16848	Lfng
05165	Human papillomavirus infection	3.485333069	2.13856E-07	1.00638E-06	18596	Pdgfrb
05165	Human papillomavirus infection	3.485333069	2.13856E-07	1.00638E-06	270118	Maml2
05165	Human papillomavirus infection	3.485333069	2.13856E-07	1.00638E-06	19651	Rbl2
05165	Human papillomavirus infection	3.485333069	2.13856E-07	1.00638E-06	16403	Itga6
05165	Human papillomavirus infection	3.485333069	2.13856E-07	1.00638E-06	56717	Mtor
05165	Human papillomavirus infection	3.485333069	2.13856E-07	1.00638E-06	14268	Fn1
05165	Human papillomavirus infection	3.485333069	2.13856E-07	1.00638E-06	16421	Itgb7
05165	Human papillomavirus infection	3.485333069	2.13856E-07	1.00638E-06	12367	Casp3
05165	Human papillomavirus infection	3.485333069	2.13856E-07	1.00638E-06	433586	Maml3
05165	Human papillomavirus infection	3.485333069	2.13856E-07	1.00638E-06	18709	Pik3r2
05165	Human papillomavirus infection	3.485333069	2.13856E-07	1.00638E-06	12695	Patj
05165	Human papillomavirus infection	3.485333069	2.13856E-07	1.00638E-06	241226	Itga8
05165	Human papillomavirus infection	3.485333069	2.13856E-07	1.00638E-06	242341	Atp6v0d2
05165	Human papillomavirus infection	3.485333069	2.13856E-07	1.00638E-06	53310	Dlg3
05165	Human papillomavirus infection	3.485333069	2.13856E-07	1.00638E-06	18710	Pik3r3
05165	Human papillomavirus infection	3.485333069	2.13856E-07	1.00638E-06	11920	Atm
05165	Human papillomavirus infection	3.485333069	2.13856E-07	1.00638E-06	23805	Apc2
05165	Human papillomavirus infection	3.485333069	2.13856E-07	1.00638E-06	12443	Ccnd1
05165	Human papillomavirus infection	3.485333069	2.13856E-07	1.00638E-06	16419	Itgb5
05165	Human papillomavirus infection	3.485333069	2.13856E-07	1.00638E-06	12428	Ccna2
05165	Human papillomavirus infection	3.485333069	2.13856E-07	1.00638E-06	208647	Creb3l2
05165	Human papillomavirus infection	3.485333069	2.13856E-07	1.00638E-06	16774	Lama3
05165	Human papillomavirus infection	3.485333069	2.13856E-07	1.00638E-06	12830	Col4a5
05145	Toxoplasmosis	6.17610769	2.73867E-07	1.27011E-06	12367	Casp3
05145	Toxoplasmosis	6.17610769	2.73867E-07	1.27011E-06	16403	Itga6

05145	Toxoplasmosis	6.17610769	2.73867E-07	1.27011E-06	15511	Hspa1b
05145	Toxoplasmosis	6.17610769	2.73867E-07	1.27011E-06	11689	Alox5
05145	Toxoplasmosis	6.17610769	2.73867E-07	1.27011E-06	16774	Lama3
05145	Toxoplasmosis	6.17610769	2.73867E-07	1.27011E-06	15979	Ifngr1
05145	Toxoplasmosis	6.17610769	2.73867E-07	1.27011E-06	14999	H2-DMb1
05145	Toxoplasmosis	6.17610769	2.73867E-07	1.27011E-06	14961	H2-Ab1
05145	Toxoplasmosis	6.17610769	2.73867E-07	1.27011E-06	14998	H2-DMa
05145	Toxoplasmosis	6.17610769	2.73867E-07	1.27011E-06	12265	Ciita
05145	Toxoplasmosis	6.17610769	2.73867E-07	1.27011E-06	26399	Map2k6
05145	Toxoplasmosis	6.17610769	2.73867E-07	1.27011E-06	193740	Hspa1a
05145	Toxoplasmosis	6.17610769	2.73867E-07	1.27011E-06	14960	H2-Aa
05145	Toxoplasmosis	6.17610769	2.73867E-07	1.27011E-06	12774	Ccr5
03460	Fanconi anemia pathway	9.515011848	3.18033E-07	1.45387E-06	19714	Rev3l
03460	Fanconi anemia pathway	9.515011848	3.18033E-07	1.45387E-06	75764	Slx1b
03460	Fanconi anemia pathway	9.515011848	3.18033E-07	1.45387E-06	233826	Palb2
03460	Fanconi anemia pathway	9.515011848	3.18033E-07	1.45387E-06	12189	Brca1
03460	Fanconi anemia pathway	9.515011848	3.18033E-07	1.45387E-06	114714	Rad51c
03460	Fanconi anemia pathway	9.515011848	3.18033E-07	1.45387E-06	27015	Polk
03460	Fanconi anemia pathway	9.515011848	3.18033E-07	1.45387E-06	12190	Brca2
03460	Fanconi anemia pathway	9.515011848	3.18033E-07	1.45387E-06	211651	Fancd2
03460	Fanconi anemia pathway	9.515011848	3.18033E-07	1.45387E-06	60534	Fancg
03460	Fanconi anemia pathway	9.515011848	3.18033E-07	1.45387E-06	104806	Fancm
01524	Platinum drug resistance	7.278984064	3.72311E-07	1.67802E-06	18709	Pik3r2
01524	Platinum drug resistance	7.278984064	3.72311E-07	1.67802E-06	12367	Casp3
01524	Platinum drug resistance	7.278984064	3.72311E-07	1.67802E-06	17688	Msh6
01524	Platinum drug resistance	7.278984064	3.72311E-07	1.67802E-06	17686	Msh3
01524	Platinum drug resistance	7.278984064	3.72311E-07	1.67802E-06	19714	Rev3l
01524	Platinum drug resistance	7.278984064	3.72311E-07	1.67802E-06	12189	Brca1
01524	Platinum drug resistance	7.278984064	3.72311E-07	1.67802E-06	17685	Msh2
01524	Platinum drug resistance	7.278984064	3.72311E-07	1.67802E-06	11920	Atm
01524	Platinum drug resistance	7.278984064	3.72311E-07	1.67802E-06	170770	Bbc3
01524	Platinum drug resistance	7.278984064	3.72311E-07	1.67802E-06	103140	Gstt3
01524	Platinum drug resistance	7.278984064	3.72311E-07	1.67802E-06	66447	Mgst3
01524	Platinum drug resistance	7.278984064	3.72311E-07	1.67802E-06	18710	Pik3r3
05231	Choline metabolism in cancer	6.437196791	4.87723E-07	2.16766E-06	13139	Dgka
05231	Choline metabolism in cancer	6.437196791	4.87723E-07	2.16766E-06	14281	Fos
05231	Choline metabolism in cancer	6.437196791	4.87723E-07	2.16766E-06	56717	Mtor
05231	Choline metabolism in cancer	6.437196791	4.87723E-07	2.16766E-06	13026	Pcyt1a
05231	Choline metabolism in cancer	6.437196791	4.87723E-07	2.16766E-06	18710	Pik3r3
05231	Choline metabolism in cancer	6.437196791	4.87723E-07	2.16766E-06	54635	Pdgfc
05231	Choline metabolism in cancer	6.437196791	4.87723E-07	2.16766E-06	18590	Pdgfa
05231	Choline metabolism in cancer	6.437196791	4.87723E-07	2.16766E-06	110197	Dgkg

05231	Choline metabolism in cancer	6.437196791	4.87723E-07	2.16766E-06	18596	Pdgfrb
05231	Choline metabolism in cancer	6.437196791	4.87723E-07	2.16766E-06	18803	Plcg1
05231	Choline metabolism in cancer	6.437196791	4.87723E-07	2.16766E-06	236899	Pcyt1b
05231	Choline metabolism in cancer	6.437196791	4.87723E-07	2.16766E-06	18591	Pdgfb
05231	Choline metabolism in cancer	6.437196791	4.87723E-07	2.16766E-06	18709	Pik3r2
04915	Estrogen signaling pathway	5.43207766	5.31253E-07	2.32878E-06	11513	Adcy7
04915	Estrogen signaling pathway	5.43207766	5.31253E-07	2.32878E-06	15511	Hspa1b
04915	Estrogen signaling pathway	5.43207766	5.31253E-07	2.32878E-06	18710	Pik3r3
04915	Estrogen signaling pathway	5.43207766	5.31253E-07	2.32878E-06	18709	Pik3r2
04915	Estrogen signaling pathway	5.43207766	5.31253E-07	2.32878E-06	17979	Ncoa3
04915	Estrogen signaling pathway	5.43207766	5.31253E-07	2.32878E-06	16438	Itpr1
04915	Estrogen signaling pathway	5.43207766	5.31253E-07	2.32878E-06	16440	Itpr3
04915	Estrogen signaling pathway	5.43207766	5.31253E-07	2.32878E-06	271849	Shc4
04915	Estrogen signaling pathway	5.43207766	5.31253E-07	2.32878E-06	193740	Hspa1a
04915	Estrogen signaling pathway	5.43207766	5.31253E-07	2.32878E-06	17977	Ncoa1
04915	Estrogen signaling pathway	5.43207766	5.31253E-07	2.32878E-06	104111	Adcy3
04915	Estrogen signaling pathway	5.43207766	5.31253E-07	2.32878E-06	216148	Shc2
04915	Estrogen signaling pathway	5.43207766	5.31253E-07	2.32878E-06	208647	Creb3l2
04915	Estrogen signaling pathway	5.43207766	5.31253E-07	2.32878E-06	14281	Fos
04915	Estrogen signaling pathway	5.43207766	5.31253E-07	2.32878E-06	18796	Plcb2
04024	cAMP signaling pathway	4.288393712	5.50128E-07	2.37893E-06	18018	Nfatc1
04024	cAMP signaling pathway	4.288393712	5.50128E-07	2.37893E-06	11555	Adrb2
04024	cAMP signaling pathway	4.288393712	5.50128E-07	2.37893E-06	14281	Fos
04024	cAMP signaling pathway	4.288393712	5.50128E-07	2.37893E-06	80911	Acox3
04024	cAMP signaling pathway	4.288393712	5.50128E-07	2.37893E-06	208647	Creb3l2
04024	cAMP signaling pathway	4.288393712	5.50128E-07	2.37893E-06	11936	Fxyd2
04024	cAMP signaling pathway	4.288393712	5.50128E-07	2.37893E-06	12322	Camk2a
04024	cAMP signaling pathway	4.288393712	5.50128E-07	2.37893E-06	18709	Pik3r2
04024	cAMP signaling pathway	4.288393712	5.50128E-07	2.37893E-06	18576	Pde3b
04024	cAMP signaling pathway	4.288393712	5.50128E-07	2.37893E-06	12289	Cacna1d
04024	cAMP signaling pathway	4.288393712	5.50128E-07	2.37893E-06	21844	Tiam1
04024	cAMP signaling pathway	4.288393712	5.50128E-07	2.37893E-06	19218	Ptger3
04024	cAMP signaling pathway	4.288393712	5.50128E-07	2.37893E-06	106952	Arap3
04024	cAMP signaling pathway	4.288393712	5.50128E-07	2.37893E-06	223864	Rapgef3
04024	cAMP signaling pathway	4.288393712	5.50128E-07	2.37893E-06	67972	Atp2b1
04024	cAMP signaling pathway	4.288393712	5.50128E-07	2.37893E-06	22325	Vav2
04024	cAMP signaling pathway	4.288393712	5.50128E-07	2.37893E-06	104111	Adcy3
04024	cAMP signaling pathway	4.288393712	5.50128E-07	2.37893E-06	18710	Pik3r3
04024	cAMP signaling pathway	4.288393712	5.50128E-07	2.37893E-06	11513	Adcy7
04923	Regulation of lipolysis in adipocytes	8.665457219	8.07296E-07	3.44446E-06	104111	Adcy3
04923	Regulation of lipolysis in adipocytes	8.665457219	8.07296E-07	3.44446E-06	11555	Adrb2
04923	Regulation of lipolysis in adipocytes	8.665457219	8.07296E-07	3.44446E-06	18576	Pde3b

04923	Regulation of lipolysis in adipocytes	8.665457219	8.07296E-07	3.44446E-06	11513	Adcy7
04923	Regulation of lipolysis in adipocytes	8.665457219	8.07296E-07	3.44446E-06	19224	Ptgs1
04923	Regulation of lipolysis in adipocytes	8.665457219	8.07296E-07	3.44446E-06	19218	Ptger3
04923	Regulation of lipolysis in adipocytes	8.665457219	8.07296E-07	3.44446E-06	23945	Mgll
04923	Regulation of lipolysis in adipocytes	8.665457219	8.07296E-07	3.44446E-06	18710	Pik3r3
04923	Regulation of lipolysis in adipocytes	8.665457219	8.07296E-07	3.44446E-06	11770	Fabp4
04923	Regulation of lipolysis in adipocytes	8.665457219	8.07296E-07	3.44446E-06	18709	Pik3r2
04919	Thyroid hormone signaling pathway	5.66143205	8.246E-07	3.472E-06	56717	Mtor
04919	Thyroid hormone signaling pathway	5.66143205	8.246E-07	3.472E-06	18803	Plcg1
04919	Thyroid hormone signaling pathway	5.66143205	8.246E-07	3.472E-06	18642	Pfkm
04919	Thyroid hormone signaling pathway	5.66143205	8.246E-07	3.472E-06	18709	Pik3r2
04919	Thyroid hormone signaling pathway	5.66143205	8.246E-07	3.472E-06	11936	Fxyd2
04919	Thyroid hormone signaling pathway	5.66143205	8.246E-07	3.472E-06	18710	Pik3r3
04919	Thyroid hormone signaling pathway	5.66143205	8.246E-07	3.472E-06	12443	Ccnd1
04919	Thyroid hormone signaling pathway	5.66143205	8.246E-07	3.472E-06	329650	Med12l
04919	Thyroid hormone signaling pathway	5.66143205	8.246E-07	3.472E-06	210789	Tbc1d4
04919	Thyroid hormone signaling pathway	5.66143205	8.246E-07	3.472E-06	18640	Pfkfb2
04919	Thyroid hormone signaling pathway	5.66143205	8.246E-07	3.472E-06	18796	Plcb2
04919	Thyroid hormone signaling pathway	5.66143205	8.246E-07	3.472E-06	17979	Ncoa3
04919	Thyroid hormone signaling pathway	5.66143205	8.246E-07	3.472E-06	20181	Rxra
04919	Thyroid hormone signaling pathway	5.66143205	8.246E-07	3.472E-06	17977	Ncoa1
04550	Signaling pathways regulating pluripotency of stem cells	5.199274331	9.47281E-07	3.93675E-06	16195	Il6st
04550	Signaling pathways regulating pluripotency of stem cells	5.199274331	9.47281E-07	3.93675E-06	15903	Id3
04550	Signaling pathways regulating pluripotency of stem cells	5.199274331	9.47281E-07	3.93675E-06	16001	Igf1r
04550	Signaling pathways regulating pluripotency of stem cells	5.199274331	9.47281E-07	3.93675E-06	11481	Acvr2b
04550	Signaling pathways regulating pluripotency of stem cells	5.199274331	9.47281E-07	3.93675E-06	75590	Dusp9
04550	Signaling pathways regulating pluripotency of stem cells	5.199274331	9.47281E-07	3.93675E-06	15394	Hoxa1
04550	Signaling pathways regulating pluripotency of stem cells	5.199274331	9.47281E-07	3.93675E-06	14182	Fgfr1
04550	Signaling pathways regulating pluripotency of stem cells	5.199274331	9.47281E-07	3.93675E-06	18709	Pik3r2
04550	Signaling pathways regulating pluripotency of stem cells	5.199274331	9.47281E-07	3.93675E-06	23805	Apc2
04550	Signaling pathways regulating pluripotency of stem cells	5.199274331	9.47281E-07	3.93675E-06	55994	Smad9
04550	Signaling pathways regulating pluripotency of stem cells	5.199274331	9.47281E-07	3.93675E-06	16600	Klf4
04550	Signaling pathways regulating pluripotency of stem cells	5.199274331	9.47281E-07	3.93675E-06	18710	Pik3r3
04550	Signaling pathways regulating pluripotency of stem cells	5.199274331	9.47281E-07	3.93675E-06	16000	Igf1
04550	Signaling pathways regulating pluripotency of stem cells	5.199274331	9.47281E-07	3.93675E-06	16880	Lifr
04550	Signaling pathways regulating pluripotency of stem cells	5.199274331	9.47281E-07	3.93675E-06	17127	Smad3
00562	Inositol phosphate metabolism	7.413780065	1.05057E-06	4.31002E-06	269180	Inpp4a
00562	Inositol phosphate metabolism	7.413780065	1.05057E-06	4.31002E-06	20975	Synj2
00562	Inositol phosphate metabolism	7.413780065	1.05057E-06	4.31002E-06	18803	Plcg1
00562	Inositol phosphate metabolism	7.413780065	1.05057E-06	4.31002E-06	16329	Inpp1
00562	Inositol phosphate metabolism	7.413780065	1.05057E-06	4.31002E-06	170835	Inpp5j
00562	Inositol phosphate metabolism	7.413780065	1.05057E-06	4.31002E-06	18796	Plcb2

00562	Inositol phosphate metabolism	7.413780065	1.05057E-06	4.31002E-06	101490	Inpp5f
00562	Inositol phosphate metabolism	7.413780065	1.05057E-06	4.31002E-06	320404	Itpkb
00562	Inositol phosphate metabolism	7.413780065	1.05057E-06	4.31002E-06	114663	Impa2
00562	Inositol phosphate metabolism	7.413780065	1.05057E-06	4.31002E-06	16332	Inppl1
00562	Inositol phosphate metabolism	7.413780065	1.05057E-06	4.31002E-06	18718	Pip4k2a
05235	PD-L1 expression and PD-1 checkpoint pathway in cancer	6.61725824	1.09106E-06	4.41949E-06	14281	Fos
05235	PD-L1 expression and PD-1 checkpoint pathway in cancer	6.61725824	1.09106E-06	4.41949E-06	56717	Mtor
05235	PD-L1 expression and PD-1 checkpoint pathway in cancer	6.61725824	1.09106E-06	4.41949E-06	18709	Pik3r2
05235	PD-L1 expression and PD-1 checkpoint pathway in cancer	6.61725824	1.09106E-06	4.41949E-06	18803	Plcg1
05235	PD-L1 expression and PD-1 checkpoint pathway in cancer	6.61725824	1.09106E-06	4.41949E-06	18019	Nfatc2
05235	PD-L1 expression and PD-1 checkpoint pathway in cancer	6.61725824	1.09106E-06	4.41949E-06	81897	Tlr9
05235	PD-L1 expression and PD-1 checkpoint pathway in cancer	6.61725824	1.09106E-06	4.41949E-06	12504	Cd4
05235	PD-L1 expression and PD-1 checkpoint pathway in cancer	6.61725824	1.09106E-06	4.41949E-06	15979	Ifngr1
05235	PD-L1 expression and PD-1 checkpoint pathway in cancer	6.61725824	1.09106E-06	4.41949E-06	26399	Map2k6
05235	PD-L1 expression and PD-1 checkpoint pathway in cancer	6.61725824	1.09106E-06	4.41949E-06	18018	Nfatc1
05235	PD-L1 expression and PD-1 checkpoint pathway in cancer	6.61725824	1.09106E-06	4.41949E-06	12487	Cd28
05235	PD-L1 expression and PD-1 checkpoint pathway in cancer	6.61725824	1.09106E-06	4.41949E-06	18710	Pik3r3
03030	DNA replication	11.09178524	1.83242E-06	7.26607E-06	17219	Mcm6
03030	DNA replication	11.09178524	1.83242E-06	7.26607E-06	327762	Dna2
03030	DNA replication	11.09178524	1.83242E-06	7.26607E-06	19718	Rfc2
03030	DNA replication	11.09178524	1.83242E-06	7.26607E-06	18973	Pole
03030	DNA replication	11.09178524	1.83242E-06	7.26607E-06	19687	Rfc1
03030	DNA replication	11.09178524	1.83242E-06	7.26607E-06	18971	Pold1
03030	DNA replication	11.09178524	1.83242E-06	7.26607E-06	18974	Pole2
03030	DNA replication	11.09178524	1.83242E-06	7.26607E-06	18968	Pola1
04380	Osteoclast differentiation	5.307592546	1.83922E-06	7.26607E-06	320832	Sirpb1a
04380	Osteoclast differentiation	5.307592546	1.83922E-06	7.26607E-06	15979	Ifngr1
04380	Osteoclast differentiation	5.307592546	1.83922E-06	7.26607E-06	21682	Tec
04380	Osteoclast differentiation	5.307592546	1.83922E-06	7.26607E-06	18018	Nfatc1
04380	Osteoclast differentiation	5.307592546	1.83922E-06	7.26607E-06	26399	Map2k6
04380	Osteoclast differentiation	5.307592546	1.83922E-06	7.26607E-06	21812	Tgfbr1
04380	Osteoclast differentiation	5.307592546	1.83922E-06	7.26607E-06	19016	Pparg
04380	Osteoclast differentiation	5.307592546	1.83922E-06	7.26607E-06	751864	Gm9733
04380	Osteoclast differentiation	5.307592546	1.83922E-06	7.26607E-06	668101	Sirpb1b
04380	Osteoclast differentiation	5.307592546	1.83922E-06	7.26607E-06	18019	Nfatc2
04380	Osteoclast differentiation	5.307592546	1.83922E-06	7.26607E-06	14281	Fos
04380	Osteoclast differentiation	5.307592546	1.83922E-06	7.26607E-06	21813	Tgfbr2
04380	Osteoclast differentiation	5.307592546	1.83922E-06	7.26607E-06	18709	Pik3r2
04380	Osteoclast differentiation	5.307592546	1.83922E-06	7.26607E-06	18710	Pik3r3
04213	Longevity regulating pathway - multiple species	7.826864585	2.17972E-06	8.50622E-06	193740	Hspa1a
04213	Longevity regulating pathway - multiple species	7.826864585	2.17972E-06	8.50622E-06	18709	Pik3r2
04213	Longevity regulating pathway - multiple species	7.826864585	2.17972E-06	8.50622E-06	16000	Igf1

04213	Longevity regulating pathway - multiple species	7.826864585	2.17972E-06	8.50622E-06	16001	Igf1r
04213	Longevity regulating pathway - multiple species	7.826864585	2.17972E-06	8.50622E-06	104111	Adcy3
04213	Longevity regulating pathway - multiple species	7.826864585	2.17972E-06	8.50622E-06	108099	Prkag2
04213	Longevity regulating pathway - multiple species	7.826864585	2.17972E-06	8.50622E-06	11513	Adcy7
04213	Longevity regulating pathway - multiple species	7.826864585	2.17972E-06	8.50622E-06	15511	Hspa1b
04213	Longevity regulating pathway - multiple species	7.826864585	2.17972E-06	8.50622E-06	18710	Pik3r3
04213	Longevity regulating pathway - multiple species	7.826864585	2.17972E-06	8.50622E-06	56717	Mtor
04726	Serotonergic synapse	5.186044626	2.44499E-06	9.42647E-06	14702	Gng2
04726	Serotonergic synapse	5.186044626	2.44499E-06	9.42647E-06	16440	Itpr3
04726	Serotonergic synapse	5.186044626	2.44499E-06	9.42647E-06	15559	Htr2b
04726	Serotonergic synapse	5.186044626	2.44499E-06	9.42647E-06	16438	Itpr1
04726	Serotonergic synapse	5.186044626	2.44499E-06	9.42647E-06	19224	Ptgs1
04726	Serotonergic synapse	5.186044626	2.44499E-06	9.42647E-06	14697	Gnb5
04726	Serotonergic synapse	5.186044626	2.44499E-06	9.42647E-06	12287	Cacna1b
04726	Serotonergic synapse	5.186044626	2.44499E-06	9.42647E-06	12367	Casp3
04726	Serotonergic synapse	5.186044626	2.44499E-06	9.42647E-06	11689	Alox5
04726	Serotonergic synapse	5.186044626	2.44499E-06	9.42647E-06	12286	Cacna1a
04726	Serotonergic synapse	5.186044626	2.44499E-06	9.42647E-06	223864	Rapgef3
04726	Serotonergic synapse	5.186044626	2.44499E-06	9.42647E-06	18796	Plcb2
04726	Serotonergic synapse	5.186044626	2.44499E-06	9.42647E-06	66066	Gng11
04726	Serotonergic synapse	5.186044626	2.44499E-06	9.42647E-06	12289	Cacna1d
04929	GnRH secretion	7.702628639	2.54316E-06	9.68822E-06	12289	Cacna1d
04929	GnRH secretion	7.702628639	2.54316E-06	9.68822E-06	114229	Kiss1r
04929	GnRH secretion	7.702628639	2.54316E-06	9.68822E-06	84036	Kcnn1
04929	GnRH secretion	7.702628639	2.54316E-06	9.68822E-06	18710	Pik3r3
04929	GnRH secretion	7.702628639	2.54316E-06	9.68822E-06	16438	Itpr1
04929	GnRH secretion	7.702628639	2.54316E-06	9.68822E-06	22066	Trpc4
04929	GnRH secretion	7.702628639	2.54316E-06	9.68822E-06	18709	Pik3r2
04929	GnRH secretion	7.702628639	2.54316E-06	9.68822E-06	109689	Arrb1
04929	GnRH secretion	7.702628639	2.54316E-06	9.68822E-06	16440	Itpr3
04929	GnRH secretion	7.702628639	2.54316E-06	9.68822E-06	18796	Plcb2
04261	Adrenergic signaling in cardiomyocytes	4.788805305	2.75488E-06	1.03713E-05	12289	Cacna1d
04261	Adrenergic signaling in cardiomyocytes	4.788805305	2.75488E-06	1.03713E-05	319734	Cacna2d4
04261	Adrenergic signaling in cardiomyocytes	4.788805305	2.75488E-06	1.03713E-05	18796	Plcb2
04261	Adrenergic signaling in cardiomyocytes	4.788805305	2.75488E-06	1.03713E-05	208647	Creb3l2
04261	Adrenergic signaling in cardiomyocytes	4.788805305	2.75488E-06	1.03713E-05	11549	Adra1a
04261	Adrenergic signaling in cardiomyocytes	4.788805305	2.75488E-06	1.03713E-05	12322	Camk2a
04261	Adrenergic signaling in cardiomyocytes	4.788805305	2.75488E-06	1.03713E-05	11513	Adcy7
04261	Adrenergic signaling in cardiomyocytes	4.788805305	2.75488E-06	1.03713E-05	12298	Cacnb4
04261	Adrenergic signaling in cardiomyocytes	4.788805305	2.75488E-06	1.03713E-05	223864	Rapgef3
04261	Adrenergic signaling in cardiomyocytes	4.788805305	2.75488E-06	1.03713E-05	11936	Fxyd2
04261	Adrenergic signaling in cardiomyocytes	4.788805305	2.75488E-06	1.03713E-05	67972	Atp2b1

04261	Adrenergic signaling in cardiomyocytes	4.788805305	2.75488E-06	1.03713E-05	20541	Slc8a1
04261	Adrenergic signaling in cardiomyocytes	4.788805305	2.75488E-06	1.03713E-05	11555	Adrb2
04261	Adrenergic signaling in cardiomyocytes	4.788805305	2.75488E-06	1.03713E-05	104111	Adcy3
04261	Adrenergic signaling in cardiomyocytes	4.788805305	2.75488E-06	1.03713E-05	73086	Rps6ka5
04925	Aldosterone synthesis and secretion	5.709007109	5.51076E-06	2.05051E-05	67972	Atp2b1
04925	Aldosterone synthesis and secretion	5.709007109	5.51076E-06	2.05051E-05	16440	Itpr3
04925	Aldosterone synthesis and secretion	5.709007109	5.51076E-06	2.05051E-05	104111	Adcy3
04925	Aldosterone synthesis and secretion	5.709007109	5.51076E-06	2.05051E-05	18796	Plcb2
04925	Aldosterone synthesis and secretion	5.709007109	5.51076E-06	2.05051E-05	269060	Dagla
04925	Aldosterone synthesis and secretion	5.709007109	5.51076E-06	2.05051E-05	11513	Adcy7
04925	Aldosterone synthesis and secretion	5.709007109	5.51076E-06	2.05051E-05	12289	Cacna1d
04925	Aldosterone synthesis and secretion	5.709007109	5.51076E-06	2.05051E-05	231871	Daglb
04925	Aldosterone synthesis and secretion	5.709007109	5.51076E-06	2.05051E-05	12322	Camk2a
04925	Aldosterone synthesis and secretion	5.709007109	5.51076E-06	2.05051E-05	208647	Creb3l2
04925	Aldosterone synthesis and secretion	5.709007109	5.51076E-06	2.05051E-05	16438	Itpr1
04925	Aldosterone synthesis and secretion	5.709007109	5.51076E-06	2.05051E-05	52163	Camk1
05142	Chagas disease	5.653579855	6.12095E-06	2.25138E-05	18709	Pik3r2
05142	Chagas disease	5.653579855	6.12095E-06	2.25138E-05	18796	Plcb2
05142	Chagas disease	5.653579855	6.12095E-06	2.25138E-05	21812	Tgfbr1
05142	Chagas disease	5.653579855	6.12095E-06	2.25138E-05	18710	Pik3r3
05142	Chagas disease	5.653579855	6.12095E-06	2.25138E-05	21813	Tgfbr2
05142	Chagas disease	5.653579855	6.12095E-06	2.25138E-05	14281	Fos
05142	Chagas disease	5.653579855	6.12095E-06	2.25138E-05	12260	C1qb
05142	Chagas disease	5.653579855	6.12095E-06	2.25138E-05	81897	Tlr9
05142	Chagas disease	5.653579855	6.12095E-06	2.25138E-05	14676	Gna15
05142	Chagas disease	5.653579855	6.12095E-06	2.25138E-05	12262	C1qc
05142	Chagas disease	5.653579855	6.12095E-06	2.25138E-05	12259	C1qa
05142	Chagas disease	5.653579855	6.12095E-06	2.25138E-05	15979	Ifngr1
04934	Cushing syndrome	4.493200039	6.19364E-06	2.25223E-05	12289	Cacna1d
04934	Cushing syndrome	4.493200039	6.19364E-06	2.25223E-05	18796	Plcb2
04934	Cushing syndrome	4.493200039	6.19364E-06	2.25223E-05	18514	Pbx1
04934	Cushing syndrome	4.493200039	6.19364E-06	2.25223E-05	208647	Creb3l2
04934	Cushing syndrome	4.493200039	6.19364E-06	2.25223E-05	12322	Camk2a
04934	Cushing syndrome	4.493200039	6.19364E-06	2.25223E-05	23805	Apc2
04934	Cushing syndrome	4.493200039	6.19364E-06	2.25223E-05	104111	Adcy3
04934	Cushing syndrome	4.493200039	6.19364E-06	2.25223E-05	16842	Lef1
04934	Cushing syndrome	4.493200039	6.19364E-06	2.25223E-05	11513	Adcy7
04934	Cushing syndrome	4.493200039	6.19364E-06	2.25223E-05	320024	Nceh1
04934	Cushing syndrome	4.493200039	6.19364E-06	2.25223E-05	16438	Itpr1
04934	Cushing syndrome	4.493200039	6.19364E-06	2.25223E-05	12580	Cdkn2c
04934	Cushing syndrome	4.493200039	6.19364E-06	2.25223E-05	242705	E2f2
04934	Cushing syndrome	4.493200039	6.19364E-06	2.25223E-05	16440	Itpr3

04934	Cushing syndrome	4.493200039	6.19364E-06	2.25223E-05	12443	Ccnd1
05132	Salmonella infection	3.644287147	6.59684E-06	2.3719E-05	16574	Kif5c
05132	Salmonella infection	3.644287147	6.59684E-06	2.3719E-05	140579	Elmo2
05132	Salmonella infection	3.644287147	6.59684E-06	2.3719E-05	14281	Fos
05132	Salmonella infection	3.644287147	6.59684E-06	2.3719E-05	226421	Rab7b
05132	Salmonella infection	3.644287147	6.59684E-06	2.3719E-05	353047	Plekhm1
05132	Salmonella infection	3.644287147	6.59684E-06	2.3719E-05	16842	Lef1
05132	Salmonella infection	3.644287147	6.59684E-06	2.3719E-05	74764	Klc4
05132	Salmonella infection	3.644287147	6.59684E-06	2.3719E-05	81897	Tlr9
05132	Salmonella infection	3.644287147	6.59684E-06	2.3719E-05	235406	Snx33
05132	Salmonella infection	3.644287147	6.59684E-06	2.3719E-05	213575	Dync2li1
05132	Salmonella infection	3.644287147	6.59684E-06	2.3719E-05	26399	Map2k6
05132	Salmonella infection	3.644287147	6.59684E-06	2.3719E-05	16572	Kif5a
05132	Salmonella infection	3.644287147	6.59684E-06	2.3719E-05	66824	Pycard
05132	Salmonella infection	3.644287147	6.59684E-06	2.3719E-05	53791	Tlr5
05132	Salmonella infection	3.644287147	6.59684E-06	2.3719E-05	12367	Casp3
05132	Salmonella infection	3.644287147	6.59684E-06	2.3719E-05	100041194	Ahnak2
05132	Salmonella infection	3.644287147	6.59684E-06	2.3719E-05	19157	Cyth1
05132	Salmonella infection	3.644287147	6.59684E-06	2.3719E-05	80837	Rhoj
05132	Salmonella infection	3.644287147	6.59684E-06	2.3719E-05	66395	Ahnak
05161	Hepatitis B	4.465634395	6.69235E-06	2.3795E-05	21813	Tgfbr2
05161	Hepatitis B	4.465634395	6.69235E-06	2.3795E-05	107986	Ddb2
05161	Hepatitis B	4.465634395	6.69235E-06	2.3795E-05	242705	E2f2
05161	Hepatitis B	4.465634395	6.69235E-06	2.3795E-05	12428	Ccna2
05161	Hepatitis B	4.465634395	6.69235E-06	2.3795E-05	26399	Map2k6
05161	Hepatitis B	4.465634395	6.69235E-06	2.3795E-05	18709	Pik3r2
05161	Hepatitis B	4.465634395	6.69235E-06	2.3795E-05	17127	Smad3
05161	Hepatitis B	4.465634395	6.69235E-06	2.3795E-05	21812	Tgfbr1
05161	Hepatitis B	4.465634395	6.69235E-06	2.3795E-05	18018	Nfatc1
05161	Hepatitis B	4.465634395	6.69235E-06	2.3795E-05	14281	Fos
05161	Hepatitis B	4.465634395	6.69235E-06	2.3795E-05	12367	Casp3
05161	Hepatitis B	4.465634395	6.69235E-06	2.3795E-05	18019	Nfatc2
05161	Hepatitis B	4.465634395	6.69235E-06	2.3795E-05	18710	Pik3r3
05161	Hepatitis B	4.465634395	6.69235E-06	2.3795E-05	26401	Map3k1
05161	Hepatitis B	4.465634395	6.69235E-06	2.3795E-05	208647	Creb3l2
05418	Fluid shear stress and atherosclerosis	4.653231822	9.04918E-06	3.18213E-05	16598	Klf2
05418	Fluid shear stress and atherosclerosis	4.653231822	9.04918E-06	3.18213E-05	18709	Pik3r2
05418	Fluid shear stress and atherosclerosis	4.653231822	9.04918E-06	3.18213E-05	14281	Fos
05418	Fluid shear stress and atherosclerosis	4.653231822	9.04918E-06	3.18213E-05	18710	Pik3r3
05418	Fluid shear stress and atherosclerosis	4.653231822	9.04918E-06	3.18213E-05	21824	Thbd
05418	Fluid shear stress and atherosclerosis	4.653231822	9.04918E-06	3.18213E-05	18590	Pdgfa
05418	Fluid shear stress and atherosclerosis	4.653231822	9.04918E-06	3.18213E-05	17258	Mef2a

05418	Fluid shear stress and atherosclerosis	4.653231822	9.04918E-06	3.18213E-05	18591	Pdgfb
05418	Fluid shear stress and atherosclerosis	4.653231822	9.04918E-06	3.18213E-05	103140	Gstt3
05418	Fluid shear stress and atherosclerosis	4.653231822	9.04918E-06	3.18213E-05	16542	Kdr
05418	Fluid shear stress and atherosclerosis	4.653231822	9.04918E-06	3.18213E-05	17260	Mef2c
05418	Fluid shear stress and atherosclerosis	4.653231822	9.04918E-06	3.18213E-05	26399	Map2k6
05418	Fluid shear stress and atherosclerosis	4.653231822	9.04918E-06	3.18213E-05	66447	Mgst3
05418	Fluid shear stress and atherosclerosis	4.653231822	9.04918E-06	3.18213E-05	11481	Acvr2b
04750	Inflammatory mediator regulation of TRP channels	4.967285713	9.95956E-06	3.4642E-05	15465	Hrh1
04750	Inflammatory mediator regulation of TRP channels	4.967285713	9.95956E-06	3.4642E-05	26399	Map2k6
04750	Inflammatory mediator regulation of TRP channels	4.967285713	9.95956E-06	3.4642E-05	16438	Itpr1
04750	Inflammatory mediator regulation of TRP channels	4.967285713	9.95956E-06	3.4642E-05	11513	Adcy7
04750	Inflammatory mediator regulation of TRP channels	4.967285713	9.95956E-06	3.4642E-05	16000	Igf1
04750	Inflammatory mediator regulation of TRP channels	4.967285713	9.95956E-06	3.4642E-05	16440	Itpr3
04750	Inflammatory mediator regulation of TRP channels	4.967285713	9.95956E-06	3.4642E-05	12322	Camk2a
04750	Inflammatory mediator regulation of TRP channels	4.967285713	9.95956E-06	3.4642E-05	18796	Plcb2
04750	Inflammatory mediator regulation of TRP channels	4.967285713	9.95956E-06	3.4642E-05	104111	Adcy3
04750	Inflammatory mediator regulation of TRP channels	4.967285713	9.95956E-06	3.4642E-05	18710	Pik3r3
04750	Inflammatory mediator regulation of TRP channels	4.967285713	9.95956E-06	3.4642E-05	18803	Plcg1
04750	Inflammatory mediator regulation of TRP channels	4.967285713	9.95956E-06	3.4642E-05	15559	Htr2b
04750	Inflammatory mediator regulation of TRP channels	4.967285713	9.95956E-06	3.4642E-05	18709	Pik3r2
04612	Antigen processing and presentation	5.931024052	1.03184E-05	3.55043E-05	15511	Hspa1b
04612	Antigen processing and presentation	5.931024052	1.03184E-05	3.55043E-05	621823	Psme2b
04612	Antigen processing and presentation	5.931024052	1.03184E-05	3.55043E-05	193740	Hspa1a
04612	Antigen processing and presentation	5.931024052	1.03184E-05	3.55043E-05	16149	Cd74
04612	Antigen processing and presentation	5.931024052	1.03184E-05	3.55043E-05	14960	H2-Aa
04612	Antigen processing and presentation	5.931024052	1.03184E-05	3.55043E-05	12504	Cd4
04612	Antigen processing and presentation	5.931024052	1.03184E-05	3.55043E-05	19141	Lgmn
04612	Antigen processing and presentation	5.931024052	1.03184E-05	3.55043E-05	14999	H2-DMb1
04612	Antigen processing and presentation	5.931024052	1.03184E-05	3.55043E-05	14961	H2-Ab1
04612	Antigen processing and presentation	5.931024052	1.03184E-05	3.55043E-05	14998	H2-DMa
04612	Antigen processing and presentation	5.931024052	1.03184E-05	3.55043E-05	12265	Ciita
04630	JAK-STAT signaling pathway	4.307091162	1.05166E-05	3.58012E-05	15979	Ifngr1
04630	JAK-STAT signaling pathway	4.307091162	1.05166E-05	3.58012E-05	16162	Il12rb2
04630	JAK-STAT signaling pathway	4.307091162	1.05166E-05	3.58012E-05	229615	Pias3
04630	JAK-STAT signaling pathway	4.307091162	1.05166E-05	3.58012E-05	18591	Pdgfb
04630	JAK-STAT signaling pathway	4.307091162	1.05166E-05	3.58012E-05	18709	Pik3r2
04630	JAK-STAT signaling pathway	4.307091162	1.05166E-05	3.58012E-05	56717	Mtor
04630	JAK-STAT signaling pathway	4.307091162	1.05166E-05	3.58012E-05	18710	Pik3r3
04630	JAK-STAT signaling pathway	4.307091162	1.05166E-05	3.58012E-05	12443	Ccnd1
04630	JAK-STAT signaling pathway	4.307091162	1.05166E-05	3.58012E-05	18590	Pdgfa
04630	JAK-STAT signaling pathway	4.307091162	1.05166E-05	3.58012E-05	18596	Pdgfrb
04630	JAK-STAT signaling pathway	4.307091162	1.05166E-05	3.58012E-05	54607	Socs6

04630	JAK-STAT signaling pathway	4.307091162	1.05166E-05	3.58012E-05	16195	Il6st
04630	JAK-STAT signaling pathway	4.307091162	1.05166E-05	3.58012E-05	50931	Il27ra
04630	JAK-STAT signaling pathway	4.307091162	1.05166E-05	3.58012E-05	16880	Lifr
04630	JAK-STAT signaling pathway	4.307091162	1.05166E-05	3.58012E-05	16194	Il6ra
04723	Retrograde endocannabinoid signaling	4.59035031	1.06344E-05	3.58213E-05	104111	Adcy3
04723	Retrograde endocannabinoid signaling	4.59035031	1.06344E-05	3.58213E-05	66066	Gng11
04723	Retrograde endocannabinoid signaling	4.59035031	1.06344E-05	3.58213E-05	14702	Gng2
04723	Retrograde endocannabinoid signaling	4.59035031	1.06344E-05	3.58213E-05	12289	Cacna1d
04723	Retrograde endocannabinoid signaling	4.59035031	1.06344E-05	3.58213E-05	23945	Mgll
04723	Retrograde endocannabinoid signaling	4.59035031	1.06344E-05	3.58213E-05	231871	Daglb
04723	Retrograde endocannabinoid signaling	4.59035031	1.06344E-05	3.58213E-05	18796	Plcb2
04723	Retrograde endocannabinoid signaling	4.59035031	1.06344E-05	3.58213E-05	11513	Adcy7
04723	Retrograde endocannabinoid signaling	4.59035031	1.06344E-05	3.58213E-05	12287	Cacna1b
04723	Retrograde endocannabinoid signaling	4.59035031	1.06344E-05	3.58213E-05	269060	Dagla
04723	Retrograde endocannabinoid signaling	4.59035031	1.06344E-05	3.58213E-05	12286	Cacna1a
04723	Retrograde endocannabinoid signaling	4.59035031	1.06344E-05	3.58213E-05	16440	Itpr3
04723	Retrograde endocannabinoid signaling	4.59035031	1.06344E-05	3.58213E-05	16438	Itpr1
04723	Retrograde endocannabinoid signaling	4.59035031	1.06344E-05	3.58213E-05	14697	Gnb5
04931	Insulin resistance	5.293806592	1.23394E-05	4.11314E-05	208647	Creb3l2
04931	Insulin resistance	5.293806592	1.23394E-05	4.11314E-05	56717	Mtor
04931	Insulin resistance	5.293806592	1.23394E-05	4.11314E-05	110078	Pygb
04931	Insulin resistance	5.293806592	1.23394E-05	4.11314E-05	100705	Acacb
04931	Insulin resistance	5.293806592	1.23394E-05	4.11314E-05	12894	Cpt1a
04931	Insulin resistance	5.293806592	1.23394E-05	4.11314E-05	18709	Pik3r2
04931	Insulin resistance	5.293806592	1.23394E-05	4.11314E-05	108099	Prkag2
04931	Insulin resistance	5.293806592	1.23394E-05	4.11314E-05	74551	Pck2
04931	Insulin resistance	5.293806592	1.23394E-05	4.11314E-05	225579	Slc27a6
04931	Insulin resistance	5.293806592	1.23394E-05	4.11314E-05	170826	Ppargc1b
04931	Insulin resistance	5.293806592	1.23394E-05	4.11314E-05	18710	Pik3r3
04931	Insulin resistance	5.293806592	1.23394E-05	4.11314E-05	210789	Tbc1d4
05414	Dilated cardiomyopathy	5.67864005	1.58877E-05	5.24129E-05	16419	Itgb5
05414	Dilated cardiomyopathy	5.67864005	1.58877E-05	5.24129E-05	104111	Adcy3
05414	Dilated cardiomyopathy	5.67864005	1.58877E-05	5.24129E-05	20541	Slc8a1
05414	Dilated cardiomyopathy	5.67864005	1.58877E-05	5.24129E-05	12298	Cacnb4
05414	Dilated cardiomyopathy	5.67864005	1.58877E-05	5.24129E-05	12289	Cacna1d
05414	Dilated cardiomyopathy	5.67864005	1.58877E-05	5.24129E-05	241226	Itga8
05414	Dilated cardiomyopathy	5.67864005	1.58877E-05	5.24129E-05	319734	Cacna2d4
05414	Dilated cardiomyopathy	5.67864005	1.58877E-05	5.24129E-05	11513	Adcy7
05414	Dilated cardiomyopathy	5.67864005	1.58877E-05	5.24129E-05	16421	Itgb7
05414	Dilated cardiomyopathy	5.67864005	1.58877E-05	5.24129E-05	16000	Igf1
05414	Dilated cardiomyopathy	5.67864005	1.58877E-05	5.24129E-05	16403	Itga6
05412	Arrhythmogenic right ventricular cardiomyopathy	6.302150705	1.68224E-05	5.49302E-05	16421	Itgb7

05412	Arrhythmogenic right ventricular cardiomyopathy	6.302150705	1.68224E-05	5.49302E-05	241226	Itga8
05412	Arrhythmogenic right ventricular cardiomyopathy	6.302150705	1.68224E-05	5.49302E-05	16403	Itga6
05412	Arrhythmogenic right ventricular cardiomyopathy	6.302150705	1.68224E-05	5.49302E-05	12289	Cacna1d
05412	Arrhythmogenic right ventricular cardiomyopathy	6.302150705	1.68224E-05	5.49302E-05	20541	Slc8a1
05412	Arrhythmogenic right ventricular cardiomyopathy	6.302150705	1.68224E-05	5.49302E-05	16480	Jup
05412	Arrhythmogenic right ventricular cardiomyopathy	6.302150705	1.68224E-05	5.49302E-05	16842	Lef1
05412	Arrhythmogenic right ventricular cardiomyopathy	6.302150705	1.68224E-05	5.49302E-05	16419	Itgb5
05412	Arrhythmogenic right ventricular cardiomyopathy	6.302150705	1.68224E-05	5.49302E-05	12298	Cacnb4
05412	Arrhythmogenic right ventricular cardiomyopathy	6.302150705	1.68224E-05	5.49302E-05	319734	Cacna2d4
03430	Mismatch repair	13.23451648	1.70511E-05	5.51145E-05	19687	Rfc1
03430	Mismatch repair	13.23451648	1.70511E-05	5.51145E-05	17686	Msh3
03430	Mismatch repair	13.23451648	1.70511E-05	5.51145E-05	17688	Msh6
03430	Mismatch repair	13.23451648	1.70511E-05	5.51145E-05	19718	Rfc2
03430	Mismatch repair	13.23451648	1.70511E-05	5.51145E-05	17685	Msh2
03430	Mismatch repair	13.23451648	1.70511E-05	5.51145E-05	18971	Pold1
05135	Yersinia infection	4.707800638	1.81347E-05	5.80309E-05	22325	Vav2
05135	Yersinia infection	4.707800638	1.81347E-05	5.80309E-05	66824	Pycard
05135	Yersinia infection	4.707800638	1.81347E-05	5.80309E-05	18019	Nfatc2
05135	Yersinia infection	4.707800638	1.81347E-05	5.80309E-05	12504	Cd4
05135	Yersinia infection	4.707800638	1.81347E-05	5.80309E-05	140579	Elmo2
05135	Yersinia infection	4.707800638	1.81347E-05	5.80309E-05	14268	Fn1
05135	Yersinia infection	4.707800638	1.81347E-05	5.80309E-05	108100	Baiap2
05135	Yersinia infection	4.707800638	1.81347E-05	5.80309E-05	18709	Pik3r2
05135	Yersinia infection	4.707800638	1.81347E-05	5.80309E-05	18018	Nfatc1
05135	Yersinia infection	4.707800638	1.81347E-05	5.80309E-05	18803	Plcg1
05135	Yersinia infection	4.707800638	1.81347E-05	5.80309E-05	18710	Pik3r3
05135	Yersinia infection	4.707800638	1.81347E-05	5.80309E-05	26399	Map2k6
05135	Yersinia infection	4.707800638	1.81347E-05	5.80309E-05	14281	Fos
03410	Base excision repair	9.99076244	2.05791E-05	6.52012E-05	17193	Mbd4
03410	Base excision repair	9.99076244	2.05791E-05	6.52012E-05	18974	Pole2
03410	Base excision repair	9.99076244	2.05791E-05	6.52012E-05	382913	Neil2
03410	Base excision repair	9.99076244	2.05791E-05	6.52012E-05	18973	Pole
03410	Base excision repair	9.99076244	2.05791E-05	6.52012E-05	18971	Pold1
03410	Base excision repair	9.99076244	2.05791E-05	6.52012E-05	234258	Neil3
03410	Base excision repair	9.99076244	2.05791E-05	6.52012E-05	11545	Parp1
00230	Purine metabolism	4.638568276	2.13642E-05	6.63742E-05	66355	Gmpr
00230	Purine metabolism	4.638568276	2.13642E-05	6.63742E-05	68870	Ak8
00230	Purine metabolism	4.638568276	2.13642E-05	6.63742E-05	20135	Rrm2
00230	Purine metabolism	4.638568276	2.13642E-05	6.63742E-05	12495	Entpd1
00230	Purine metabolism	4.638568276	2.13642E-05	6.63742E-05	18575	Pde1c
00230	Purine metabolism	4.638568276	2.13642E-05	6.63742E-05	104111	Adcy3
00230	Purine metabolism	4.638568276	2.13642E-05	6.63742E-05	11513	Adcy7

00230	Purine metabolism	4.638568276	2.13642E-05	6.63742E-05	11486	Ada
00230	Purine metabolism	4.638568276	2.13642E-05	6.63742E-05	11565	Adssl1
00230	Purine metabolism	4.638568276	2.13642E-05	6.63742E-05	18576	Pde3b
00230	Purine metabolism	4.638568276	2.13642E-05	6.63742E-05	110639	Prps2
00230	Purine metabolism	4.638568276	2.13642E-05	6.63742E-05	22436	Xdh
00230	Purine metabolism	4.638568276	2.13642E-05	6.63742E-05	18605	Enpp1
04210	Apoptosis	4.638568276	2.13642E-05	6.63742E-05	16440	Itpr3
04210	Apoptosis	4.638568276	2.13642E-05	6.63742E-05	11545	Parp1
04210	Apoptosis	4.638568276	2.13642E-05	6.63742E-05	18710	Pik3r3
04210	Apoptosis	4.638568276	2.13642E-05	6.63742E-05	23882	Gadd45g
04210	Apoptosis	4.638568276	2.13642E-05	6.63742E-05	11920	Atm
04210	Apoptosis	4.638568276	2.13642E-05	6.63742E-05	12334	Capn2
04210	Apoptosis	4.638568276	2.13642E-05	6.63742E-05	170770	Bbc3
04210	Apoptosis	4.638568276	2.13642E-05	6.63742E-05	56464	Ctsf
04210	Apoptosis	4.638568276	2.13642E-05	6.63742E-05	14281	Fos
04210	Apoptosis	4.638568276	2.13642E-05	6.63742E-05	12368	Casp6
04210	Apoptosis	4.638568276	2.13642E-05	6.63742E-05	12367	Casp3
04210	Apoptosis	4.638568276	2.13642E-05	6.63742E-05	16438	Itpr1
04210	Apoptosis	4.638568276	2.13642E-05	6.63742E-05	18709	Pik3r2
00564	Glycerophospholipid metabolism	5.446858823	2.39238E-05	7.36116E-05	110197	Dgkg
00564	Glycerophospholipid metabolism	5.446858823	2.39238E-05	7.36116E-05	104759	Pld4
00564	Glycerophospholipid metabolism	5.446858823	2.39238E-05	7.36116E-05	13139	Dgka
00564	Glycerophospholipid metabolism	5.446858823	2.39238E-05	7.36116E-05	210992	Lpcat1
00564	Glycerophospholipid metabolism	5.446858823	2.39238E-05	7.36116E-05	218121	Mboat1
00564	Glycerophospholipid metabolism	5.446858823	2.39238E-05	7.36116E-05	241274	Pnpla7
00564	Glycerophospholipid metabolism	5.446858823	2.39238E-05	7.36116E-05	236899	Pcyt1b
00564	Glycerophospholipid metabolism	5.446858823	2.39238E-05	7.36116E-05	13026	Pcyt1a
00564	Glycerophospholipid metabolism	5.446858823	2.39238E-05	7.36116E-05	333433	Gpd1l
00564	Glycerophospholipid metabolism	5.446858823	2.39238E-05	7.36116E-05	14555	Gpd1
00564	Glycerophospholipid metabolism	5.446858823	2.39238E-05	7.36116E-05	192654	Pla2g15
04662	B cell receptor signaling pathway	5.990933386	2.67421E-05	8.14997E-05	12483	Cd22
04662	B cell receptor signaling pathway	5.990933386	2.67421E-05	8.14997E-05	18019	Nfatc2
04662	B cell receptor signaling pathway	5.990933386	2.67421E-05	8.14997E-05	240168	Rasgrp3
04662	B cell receptor signaling pathway	5.990933386	2.67421E-05	8.14997E-05	108723	Card11
04662	B cell receptor signaling pathway	5.990933386	2.67421E-05	8.14997E-05	18018	Nfatc1
04662	B cell receptor signaling pathway	5.990933386	2.67421E-05	8.14997E-05	16332	Inppl1
04662	B cell receptor signaling pathway	5.990933386	2.67421E-05	8.14997E-05	18710	Pik3r3
04662	B cell receptor signaling pathway	5.990933386	2.67421E-05	8.14997E-05	14281	Fos
04662	B cell receptor signaling pathway	5.990933386	2.67421E-05	8.14997E-05	22325	Vav2
04662	B cell receptor signaling pathway	5.990933386	2.67421E-05	8.14997E-05	18709	Pik3r2
04910	Insulin signaling pathway	4.538455292	2.71619E-05	8.19982E-05	17347	Mknk2
04910	Insulin signaling pathway	4.538455292	2.71619E-05	8.19982E-05	108099	Prkag2

04910	Insulin signaling pathway	4.538455292	2.71619E-05	8.19982E-05	271849	Shc4
04910	Insulin signaling pathway	4.538455292	2.71619E-05	8.19982E-05	16332	Inppl1
04910	Insulin signaling pathway	4.538455292	2.71619E-05	8.19982E-05	18710	Pik3r3
04910	Insulin signaling pathway	4.538455292	2.71619E-05	8.19982E-05	74551	Pck2
04910	Insulin signaling pathway	4.538455292	2.71619E-05	8.19982E-05	18709	Pik3r2
04910	Insulin signaling pathway	4.538455292	2.71619E-05	8.19982E-05	110094	Phka2
04910	Insulin signaling pathway	4.538455292	2.71619E-05	8.19982E-05	216148	Shc2
04910	Insulin signaling pathway	4.538455292	2.71619E-05	8.19982E-05	18576	Pde3b
04910	Insulin signaling pathway	4.538455292	2.71619E-05	8.19982E-05	110078	Pygb
04910	Insulin signaling pathway	4.538455292	2.71619E-05	8.19982E-05	100705	Acacb
04910	Insulin signaling pathway	4.538455292	2.71619E-05	8.19982E-05	56717	Mtor
04114	Oocyte meiosis	4.893434665	2.81001E-05	8.40376E-05	12322	Camk2a
04114	Oocyte meiosis	4.893434665	2.81001E-05	8.40376E-05	11513	Adcy7
04114	Oocyte meiosis	4.893434665	2.81001E-05	8.40376E-05	30939	Pttg1
04114	Oocyte meiosis	4.893434665	2.81001E-05	8.40376E-05	12442	Ccnb2
04114	Oocyte meiosis	4.893434665	2.81001E-05	8.40376E-05	16000	Igf1
04114	Oocyte meiosis	4.893434665	2.81001E-05	8.40376E-05	104111	Adcy3
04114	Oocyte meiosis	4.893434665	2.81001E-05	8.40376E-05	16001	Igf1r
04114	Oocyte meiosis	4.893434665	2.81001E-05	8.40376E-05	16440	Itpr3
04114	Oocyte meiosis	4.893434665	2.81001E-05	8.40376E-05	12877	Cpeb1
04114	Oocyte meiosis	4.893434665	2.81001E-05	8.40376E-05	208922	Cpeb3
04114	Oocyte meiosis	4.893434665	2.81001E-05	8.40376E-05	16438	Itpr1
04114	Oocyte meiosis	4.893434665	2.81001E-05	8.40376E-05	18817	Plk1
05169	Epstein-Barr virus infection	3.571218733	2.98573E-05	8.8466E-05	107986	Ddb2
05169	Epstein-Barr virus infection	3.571218733	2.98573E-05	8.8466E-05	12428	Ccna2
05169	Epstein-Barr virus infection	3.571218733	2.98573E-05	8.8466E-05	23882	Gadd45g
05169	Epstein-Barr virus infection	3.571218733	2.98573E-05	8.8466E-05	12367	Casp3
05169	Epstein-Barr virus infection	3.571218733	2.98573E-05	8.8466E-05	12399	Runx3
05169	Epstein-Barr virus infection	3.571218733	2.98573E-05	8.8466E-05	242705	E2f2
05169	Epstein-Barr virus infection	3.571218733	2.98573E-05	8.8466E-05	27015	Polk
05169	Epstein-Barr virus infection	3.571218733	2.98573E-05	8.8466E-05	27401	Skp2
05169	Epstein-Barr virus infection	3.571218733	2.98573E-05	8.8466E-05	12443	Ccnd1
05169	Epstein-Barr virus infection	3.571218733	2.98573E-05	8.8466E-05	14961	H2-Ab1
05169	Epstein-Barr virus infection	3.571218733	2.98573E-05	8.8466E-05	26399	Map2k6
05169	Epstein-Barr virus infection	3.571218733	2.98573E-05	8.8466E-05	14960	H2-Aa
05169	Epstein-Barr virus infection	3.571218733	2.98573E-05	8.8466E-05	12495	Entpd1
05169	Epstein-Barr virus infection	3.571218733	2.98573E-05	8.8466E-05	14999	H2-DMb1
05169	Epstein-Barr virus infection	3.571218733	2.98573E-05	8.8466E-05	18709	Pik3r2
05169	Epstein-Barr virus infection	3.571218733	2.98573E-05	8.8466E-05	14998	H2-DMa
05169	Epstein-Barr virus infection	3.571218733	2.98573E-05	8.8466E-05	18710	Pik3r3
05216	Thyroid cancer	9.180700621	3.7318E-05	0.000109557	27015	Polk
05216	Thyroid cancer	9.180700621	3.7318E-05	0.000109557	16842	Lef1

05216	Thyroid cancer	9.180700621	3.7318E-05	0.000109557	23882	Gadd45g
05216	Thyroid cancer	9.180700621	3.7318E-05	0.000109557	12443	Ccnd1
05216	Thyroid cancer	9.180700621	3.7318E-05	0.000109557	19016	Pparg
05216	Thyroid cancer	9.180700621	3.7318E-05	0.000109557	107986	Ddb2
05216	Thyroid cancer	9.180700621	3.7318E-05	0.000109557	20181	Rxra
04611	Platelet activation	4.696118751	4.2928E-05	0.000124882	18796	Plcb2
04611	Platelet activation	4.696118751	4.2928E-05	0.000124882	18710	Pik3r3
04611	Platelet activation	4.696118751	4.2928E-05	0.000124882	16440	Itpr3
04611	Platelet activation	4.696118751	4.2928E-05	0.000124882	19395	Rasgrp2
04611	Platelet activation	4.696118751	4.2928E-05	0.000124882	18709	Pik3r2
04611	Platelet activation	4.696118751	4.2928E-05	0.000124882	18441	P2ry1
04611	Platelet activation	4.696118751	4.2928E-05	0.000124882	11513	Adcy7
04611	Platelet activation	4.696118751	4.2928E-05	0.000124882	104111	Adcy3
04611	Platelet activation	4.696118751	4.2928E-05	0.000124882	70839	P2ry12
04611	Platelet activation	4.696118751	4.2928E-05	0.000124882	19224	Ptgs1
04611	Platelet activation	4.696118751	4.2928E-05	0.000124882	16438	Itpr1
04611	Platelet activation	4.696118751	4.2928E-05	0.000124882	14723	Gp1ba
05010	Alzheimer disease	2.893182464	4.48283E-05	0.000128961	27373	Csnk1e
05010	Alzheimer disease	2.893182464	4.48283E-05	0.000128961	16973	Lrp5
05010	Alzheimer disease	2.893182464	4.48283E-05	0.000128961	12289	Cacna1d
05010	Alzheimer disease	2.893182464	4.48283E-05	0.000128961	12367	Casp3
05010	Alzheimer disease	2.893182464	4.48283E-05	0.000128961	16438	Itpr1
05010	Alzheimer disease	2.893182464	4.48283E-05	0.000128961	18710	Pik3r3
05010	Alzheimer disease	2.893182464	4.48283E-05	0.000128961	16572	Kif5a
05010	Alzheimer disease	2.893182464	4.48283E-05	0.000128961	22241	Ulk1
05010	Alzheimer disease	2.893182464	4.48283E-05	0.000128961	23821	Bace1
05010	Alzheimer disease	2.893182464	4.48283E-05	0.000128961	68318	Aph1c
05010	Alzheimer disease	2.893182464	4.48283E-05	0.000128961	14296	Frat1
05010	Alzheimer disease	2.893182464	4.48283E-05	0.000128961	18796	Plcb2
05010	Alzheimer disease	2.893182464	4.48283E-05	0.000128961	56717	Mtor
05010	Alzheimer disease	2.893182464	4.48283E-05	0.000128961	16440	Itpr3
05010	Alzheimer disease	2.893182464	4.48283E-05	0.000128961	12334	Capn2
05010	Alzheimer disease	2.893182464	4.48283E-05	0.000128961	74764	Klc4
05010	Alzheimer disease	2.893182464	4.48283E-05	0.000128961	16971	Lrp1
05010	Alzheimer disease	2.893182464	4.48283E-05	0.000128961	18709	Pik3r2
05010	Alzheimer disease	2.893182464	4.48283E-05	0.000128961	16574	Kif5c
05010	Alzheimer disease	2.893182464	4.48283E-05	0.000128961	23805	Apc2
05010	Alzheimer disease	2.893182464	4.48283E-05	0.000128961	18125	Nos1
05010	Alzheimer disease	2.893182464	4.48283E-05	0.000128961	212398	Frat2
04540	Gap junction	5.642623305	4.59423E-05	0.000128961	15559	Htr2b
04540	Gap junction	5.642623305	4.59423E-05	0.000128961	11513	Adcy7
04540	Gap junction	5.642623305	4.59423E-05	0.000128961	54635	Pdgfc

04540	Gap junction	5.642623305	4.59423E-05	0.000128961	16438	Itpr1
04540	Gap junction	5.642623305	4.59423E-05	0.000128961	18796	Plcb2
04540	Gap junction	5.642623305	4.59423E-05	0.000128961	104111	Adcy3
04540	Gap junction	5.642623305	4.59423E-05	0.000128961	16440	Itpr3
04540	Gap junction	5.642623305	4.59423E-05	0.000128961	18591	Pdgfb
04540	Gap junction	5.642623305	4.59423E-05	0.000128961	18590	Pdgfa
04540	Gap junction	5.642623305	4.59423E-05	0.000128961	18596	Pdgfrb
04911	Insulin secretion	5.642623305	4.59423E-05	0.000128961	16440	Itpr3
04911	Insulin secretion	5.642623305	4.59423E-05	0.000128961	84036	Kcnn1
04911	Insulin secretion	5.642623305	4.59423E-05	0.000128961	11936	Fxyd2
04911	Insulin secretion	5.642623305	4.59423E-05	0.000128961	208647	Creb3l2
04911	Insulin secretion	5.642623305	4.59423E-05	0.000128961	11513	Adcy7
04911	Insulin secretion	5.642623305	4.59423E-05	0.000128961	19339	Rab3a
04911	Insulin secretion	5.642623305	4.59423E-05	0.000128961	12322	Camk2a
04911	Insulin secretion	5.642623305	4.59423E-05	0.000128961	12289	Cacna1d
04911	Insulin secretion	5.642623305	4.59423E-05	0.000128961	104111	Adcy3
04911	Insulin secretion	5.642623305	4.59423E-05	0.000128961	18796	Plcb2
04970	Salivary secretion	5.642623305	4.59423E-05	0.000128961	104111	Adcy3
04970	Salivary secretion	5.642623305	4.59423E-05	0.000128961	18125	Nos1
04970	Salivary secretion	5.642623305	4.59423E-05	0.000128961	16438	Itpr1
04970	Salivary secretion	5.642623305	4.59423E-05	0.000128961	11513	Adcy7
04970	Salivary secretion	5.642623305	4.59423E-05	0.000128961	11555	Adrb2
04970	Salivary secretion	5.642623305	4.59423E-05	0.000128961	16440	Itpr3
04970	Salivary secretion	5.642623305	4.59423E-05	0.000128961	11936	Fxyd2
04970	Salivary secretion	5.642623305	4.59423E-05	0.000128961	67972	Atp2b1
04970	Salivary secretion	5.642623305	4.59423E-05	0.000128961	18796	Plcb2
04970	Salivary secretion	5.642623305	4.59423E-05	0.000128961	11549	Adra1a
05230	Central carbon metabolism in cancer	6.32955136	4.88888E-05	0.000136038	15926	Idh1
05230	Central carbon metabolism in cancer	6.32955136	4.88888E-05	0.000136038	18709	Pik3r2
05230	Central carbon metabolism in cancer	6.32955136	4.88888E-05	0.000136038	16832	Ldhb
05230	Central carbon metabolism in cancer	6.32955136	4.88888E-05	0.000136038	64384	Sirt3
05230	Central carbon metabolism in cancer	6.32955136	4.88888E-05	0.000136038	18596	Pdgfrb
05230	Central carbon metabolism in cancer	6.32955136	4.88888E-05	0.000136038	56717	Mtor
05230	Central carbon metabolism in cancer	6.32955136	4.88888E-05	0.000136038	18642	Pfkm
05230	Central carbon metabolism in cancer	6.32955136	4.88888E-05	0.000136038	14182	Fgfr1
05230	Central carbon metabolism in cancer	6.32955136	4.88888E-05	0.000136038	18710	Pik3r3
05221	Acute myeloid leukemia	6.239129197	5.50795E-05	0.000151944	18710	Pik3r3
05221	Acute myeloid leukemia	6.239129197	5.50795E-05	0.000151944	12606	Cebpa
05221	Acute myeloid leukemia	6.239129197	5.50795E-05	0.000151944	18627	Per2
05221	Acute myeloid leukemia	6.239129197	5.50795E-05	0.000151944	18709	Pik3r2
05221	Acute myeloid leukemia	6.239129197	5.50795E-05	0.000151944	16842	Lef1
05221	Acute myeloid leukemia	6.239129197	5.50795E-05	0.000151944	56717	Mtor

05221	Acute myeloid leukemia	6.239129197	5.50795E-05	0.000151944	12428	Ccna2
05221	Acute myeloid leukemia	6.239129197	5.50795E-05	0.000151944	16480	Jup
05221	Acute myeloid leukemia	6.239129197	5.50795E-05	0.000151944	12443	Ccnd1
05146	Amoebiasis	4.988711819	5.59141E-05	0.000152927	20708	Serpinb6b
05146	Amoebiasis	4.988711819	5.59141E-05	0.000152927	14676	Gna15
05146	Amoebiasis	4.988711819	5.59141E-05	0.000152927	226421	Rab7b
05146	Amoebiasis	4.988711819	5.59141E-05	0.000152927	12830	Col4a5
05146	Amoebiasis	4.988711819	5.59141E-05	0.000152927	18796	Plcb2
05146	Amoebiasis	4.988711819	5.59141E-05	0.000152927	12367	Casp3
05146	Amoebiasis	4.988711819	5.59141E-05	0.000152927	20719	Serpinb6a
05146	Amoebiasis	4.988711819	5.59141E-05	0.000152927	18709	Pik3r2
05146	Amoebiasis	4.988711819	5.59141E-05	0.000152927	16774	Lama3
05146	Amoebiasis	4.988711819	5.59141E-05	0.000152927	18710	Pik3r3
05146	Amoebiasis	4.988711819	5.59141E-05	0.000152927	14268	Fn1
04520	Adherens junction	6.151254138	6.19258E-05	0.000167934	17127	Smad3
04520	Adherens junction	6.151254138	6.19258E-05	0.000167934	14182	Fgfr1
04520	Adherens junction	6.151254138	6.19258E-05	0.000167934	21813	Tgfbr2
04520	Adherens junction	6.151254138	6.19258E-05	0.000167934	21812	Tgfbr1
04520	Adherens junction	6.151254138	6.19258E-05	0.000167934	58235	Nectin1
04520	Adherens junction	6.151254138	6.19258E-05	0.000167934	71740	Nectin4
04520	Adherens junction	6.151254138	6.19258E-05	0.000167934	16842	Lef1
04520	Adherens junction	6.151254138	6.19258E-05	0.000167934	108100	Baiap2
04520	Adherens junction	6.151254138	6.19258E-05	0.000167934	16001	Igf1r
04740	Olfactory transduction	0.249493884	6.71828E-05	0.00018066	104111	Adcy3
04740	Olfactory transduction	0.249493884	6.71828E-05	0.00018066	19735	Rgs2
04740	Olfactory transduction	0.249493884	6.71828E-05	0.00018066	12322	Camk2a
04740	Olfactory transduction	0.249493884	6.71828E-05	0.00018066	18575	Pde1c
04740	Olfactory transduction	0.249493884	6.71828E-05	0.00018066	109689	Arrb1
04740	Olfactory transduction	0.249493884	6.71828E-05	0.00018066	20541	Slc8a1
04912	GnRH signaling pathway	5.391840047	6.89167E-05	0.000183757	12289	Cacna1d
04912	GnRH signaling pathway	5.391840047	6.89167E-05	0.000183757	26401	Map3k1
04912	GnRH signaling pathway	5.391840047	6.89167E-05	0.000183757	11513	Adcy7
04912	GnRH signaling pathway	5.391840047	6.89167E-05	0.000183757	26407	Map3k4
04912	GnRH signaling pathway	5.391840047	6.89167E-05	0.000183757	16438	Itpr1
04912	GnRH signaling pathway	5.391840047	6.89167E-05	0.000183757	104111	Adcy3
04912	GnRH signaling pathway	5.391840047	6.89167E-05	0.000183757	16440	Itpr3
04912	GnRH signaling pathway	5.391840047	6.89167E-05	0.000183757	12322	Camk2a
04912	GnRH signaling pathway	5.391840047	6.89167E-05	0.000183757	18796	Plcb2
04912	GnRH signaling pathway	5.391840047	6.89167E-05	0.000183757	26399	Map2k6
04927	Cortisol synthesis and secretion	6.065820053	6.94832E-05	0.000183757	16440	Itpr3
04927	Cortisol synthesis and secretion	6.065820053	6.94832E-05	0.000183757	16438	Itpr1
04927	Cortisol synthesis and secretion	6.065820053	6.94832E-05	0.000183757	18514	Pbx1

04927	Cortisol synthesis and secretion	6.065820053	6.94832E-05	0.000183757	18796	Plcb2
04927	Cortisol synthesis and secretion	6.065820053	6.94832E-05	0.000183757	320024	Nceh1
04927	Cortisol synthesis and secretion	6.065820053	6.94832E-05	0.000183757	11513	Adcy7
04927	Cortisol synthesis and secretion	6.065820053	6.94832E-05	0.000183757	12289	Cacna1d
04927	Cortisol synthesis and secretion	6.065820053	6.94832E-05	0.000183757	208647	Creb3l2
04927	Cortisol synthesis and secretion	6.065820053	6.94832E-05	0.000183757	104111	Adcy3
05032	Morphine addiction	5.332589058	7.60028E-05	0.000197731	12287	Cacna1b
05032	Morphine addiction	5.332589058	7.60028E-05	0.000197731	11513	Adcy7
05032	Morphine addiction	5.332589058	7.60028E-05	0.000197731	66066	Gng11
05032	Morphine addiction	5.332589058	7.60028E-05	0.000197731	104111	Adcy3
05032	Morphine addiction	5.332589058	7.60028E-05	0.000197731	18576	Pde3b
05032	Morphine addiction	5.332589058	7.60028E-05	0.000197731	14702	Gng2
05032	Morphine addiction	5.332589058	7.60028E-05	0.000197731	14697	Gnb5
05032	Morphine addiction	5.332589058	7.60028E-05	0.000197731	12286	Cacna1a
05032	Morphine addiction	5.332589058	7.60028E-05	0.000197731	18575	Pde1c
05032	Morphine addiction	5.332589058	7.60028E-05	0.000197731	109689	Arrb1
05410	Hypertrophic cardiomyopathy	5.332589058	7.60028E-05	0.000197731	241226	Itga8
05410	Hypertrophic cardiomyopathy	5.332589058	7.60028E-05	0.000197731	12289	Cacna1d
05410	Hypertrophic cardiomyopathy	5.332589058	7.60028E-05	0.000197731	16421	Itgb7
05410	Hypertrophic cardiomyopathy	5.332589058	7.60028E-05	0.000197731	16403	Itga6
05410	Hypertrophic cardiomyopathy	5.332589058	7.60028E-05	0.000197731	16419	Itgb5
05410	Hypertrophic cardiomyopathy	5.332589058	7.60028E-05	0.000197731	319734	Cacna2d4
05410	Hypertrophic cardiomyopathy	5.332589058	7.60028E-05	0.000197731	12298	Cacnb4
05410	Hypertrophic cardiomyopathy	5.332589058	7.60028E-05	0.000197731	16000	Igf1
05410	Hypertrophic cardiomyopathy	5.332589058	7.60028E-05	0.000197731	108099	Prkag2
05410	Hypertrophic cardiomyopathy	5.332589058	7.60028E-05	0.000197731	20541	Slc8a1
04150	mTOR signaling pathway	4.043880035	9.4213E-05	0.00024313	16973	Lrp5
04150	mTOR signaling pathway	4.043880035	9.4213E-05	0.00024313	16000	Igf1
04150	mTOR signaling pathway	4.043880035	9.4213E-05	0.00024313	27401	Skp2
04150	mTOR signaling pathway	4.043880035	9.4213E-05	0.00024313	16001	Igf1r
04150	mTOR signaling pathway	4.043880035	9.4213E-05	0.00024313	97998	Deptor
04150	mTOR signaling pathway	4.043880035	9.4213E-05	0.00024313	56717	Mtor
04150	mTOR signaling pathway	4.043880035	9.4213E-05	0.00024313	329679	Fnip2
04150	mTOR signaling pathway	4.043880035	9.4213E-05	0.00024313	22241	Ulk1
04150	mTOR signaling pathway	4.043880035	9.4213E-05	0.00024313	52187	Rragd
04150	mTOR signaling pathway	4.043880035	9.4213E-05	0.00024313	216805	Flcn
04150	mTOR signaling pathway	4.043880035	9.4213E-05	0.00024313	18709	Pik3r2
04150	mTOR signaling pathway	4.043880035	9.4213E-05	0.00024313	18710	Pik3r3
04150	mTOR signaling pathway	4.043880035	9.4213E-05	0.00024313	80909	Castor2
00062	Fatty acid elongation	10.03997802	9.59555E-05	0.000245646	26897	Acot1
00062	Fatty acid elongation	10.03997802	9.59555E-05	0.000245646	52538	Acaa2
00062	Fatty acid elongation	10.03997802	9.59555E-05	0.000245646	68801	Elovl5

00062	Fatty acid elongation	10.03997802	9.59555E-05	0.000245646	171210	Acot2
00062	Fatty acid elongation	10.03997802	9.59555E-05	0.000245646	66775	Hacd4
00062	Fatty acid elongation	10.03997802	9.59555E-05	0.000245646	15107	Hadh
05213	Endometrial cancer	6.693318679	9.81345E-05	0.00024923	12443	Ccnd1
05213	Endometrial cancer	6.693318679	9.81345E-05	0.00024923	23805	Apc2
05213	Endometrial cancer	6.693318679	9.81345E-05	0.00024923	18710	Pik3r3
05213	Endometrial cancer	6.693318679	9.81345E-05	0.00024923	23882	Gadd45g
05213	Endometrial cancer	6.693318679	9.81345E-05	0.00024923	107986	Ddb2
05213	Endometrial cancer	6.693318679	9.81345E-05	0.00024923	16842	Lef1
05213	Endometrial cancer	6.693318679	9.81345E-05	0.00024923	27015	Polk
05213	Endometrial cancer	6.693318679	9.81345E-05	0.00024923	18709	Pik3r2
03420	Nucleotide excision repair	7.899672627	0.000104884	0.000264275	22591	Xpc
03420	Nucleotide excision repair	7.899672627	0.000104884	0.000264275	19687	Rfc1
03420	Nucleotide excision repair	7.899672627	0.000104884	0.000264275	18974	Pole2
03420	Nucleotide excision repair	7.899672627	0.000104884	0.000264275	19718	Rfc2
03420	Nucleotide excision repair	7.899672627	0.000104884	0.000264275	18973	Pole
03420	Nucleotide excision repair	7.899672627	0.000104884	0.000264275	107986	Ddb2
03420	Nucleotide excision repair	7.899672627	0.000104884	0.000264275	18971	Pold1
04710	Circadian rhythm	9.705312085	0.000117852	0.000294631	18143	Npas2
04710	Circadian rhythm	9.705312085	0.000117852	0.000294631	18628	Per3
04710	Circadian rhythm	9.705312085	0.000117852	0.000294631	18627	Per2
04710	Circadian rhythm	9.705312085	0.000117852	0.000294631	217166	Nr1d1
04710	Circadian rhythm	9.705312085	0.000117852	0.000294631	108099	Prkag2
04710	Circadian rhythm	9.705312085	0.000117852	0.000294631	27373	Csnk1e
04730	Long-term depression	6.470208057	0.00012632	0.000313352	18125	Nos1
04730	Long-term depression	6.470208057	0.00012632	0.000313352	12286	Cacna1a
04730	Long-term depression	6.470208057	0.00012632	0.000313352	18796	Plcb2
04730	Long-term depression	6.470208057	0.00012632	0.000313352	16001	Igf1r
04730	Long-term depression	6.470208057	0.00012632	0.000313352	16438	Itpr1
04730	Long-term depression	6.470208057	0.00012632	0.000313352	16440	Itpr3
04730	Long-term depression	6.470208057	0.00012632	0.000313352	14673	Gna12
04730	Long-term depression	6.470208057	0.00012632	0.000313352	16000	Igf1
00920	Sulfur metabolism	17.64602197	0.000211184	0.000519839	22117	Tst
00920	Sulfur metabolism	17.64602197	0.000211184	0.000519839	211389	Suox
00920	Sulfur metabolism	17.64602197	0.000211184	0.000519839	20342	Selenbp2
00920	Sulfur metabolism	17.64602197	0.000211184	0.000519839	20341	Selenbp1
05022	Pathways of neurodegeneration - multiple diseases	2.467452225	0.000240614	0.000587759	16438	Itpr1
05022	Pathways of neurodegeneration - multiple diseases	2.467452225	0.000240614	0.000587759	12289	Cacna1d
05022	Pathways of neurodegeneration - multiple diseases	2.467452225	0.000240614	0.000587759	22241	Ulk1
05022	Pathways of neurodegeneration - multiple diseases	2.467452225	0.000240614	0.000587759	50873	Prkn
05022	Pathways of neurodegeneration - multiple diseases	2.467452225	0.000240614	0.000587759	16572	Kif5a
05022	Pathways of neurodegeneration - multiple diseases	2.467452225	0.000240614	0.000587759	18803	Plcg1

05022	Pathways of neurodegeneration - multiple diseases	2.467452225	0.000240614	0.000587759	12287	Cacna1b
05022	Pathways of neurodegeneration - multiple diseases	2.467452225	0.000240614	0.000587759	18796	Plcb2
05022	Pathways of neurodegeneration - multiple diseases	2.467452225	0.000240614	0.000587759	18125	Nos1
05022	Pathways of neurodegeneration - multiple diseases	2.467452225	0.000240614	0.000587759	215114	Hip1
05022	Pathways of neurodegeneration - multiple diseases	2.467452225	0.000240614	0.000587759	12322	Camk2a
05022	Pathways of neurodegeneration - multiple diseases	2.467452225	0.000240614	0.000587759	74764	Klc4
05022	Pathways of neurodegeneration - multiple diseases	2.467452225	0.000240614	0.000587759	56717	Mtor
05022	Pathways of neurodegeneration - multiple diseases	2.467452225	0.000240614	0.000587759	27373	Csnk1e
05022	Pathways of neurodegeneration - multiple diseases	2.467452225	0.000240614	0.000587759	12367	Casp3
05022	Pathways of neurodegeneration - multiple diseases	2.467452225	0.000240614	0.000587759	16440	Itpr3
05022	Pathways of neurodegeneration - multiple diseases	2.467452225	0.000240614	0.000587759	26399	Map2k6
05022	Pathways of neurodegeneration - multiple diseases	2.467452225	0.000240614	0.000587759	16973	Lrp5
05022	Pathways of neurodegeneration - multiple diseases	2.467452225	0.000240614	0.000587759	14296	Frat1
05022	Pathways of neurodegeneration - multiple diseases	2.467452225	0.000240614	0.000587759	23805	Apc2
05022	Pathways of neurodegeneration - multiple diseases	2.467452225	0.000240614	0.000587759	16574	Kif5c
05022	Pathways of neurodegeneration - multiple diseases	2.467452225	0.000240614	0.000587759	68943	Pink1
05022	Pathways of neurodegeneration - multiple diseases	2.467452225	0.000240614	0.000587759	212398	Frat2
05022	Pathways of neurodegeneration - multiple diseases	2.467452225	0.000240614	0.000587759	12334	Capn2
00600	Sphingolipid metabolism	6.793718459	0.000286818	0.000695316	67260	Cers4
00600	Sphingolipid metabolism	6.793718459	0.000286818	0.000695316	20598	Smpd2
00600	Sphingolipid metabolism	6.793718459	0.000286818	0.000695316	81535	Sgpp1
00600	Sphingolipid metabolism	6.793718459	0.000286818	0.000695316	230101	Gba2
00600	Sphingolipid metabolism	6.793718459	0.000286818	0.000695316	14420	Galc
00600	Sphingolipid metabolism	6.793718459	0.000286818	0.000695316	223753	Cerk
00600	Sphingolipid metabolism	6.793718459	0.000286818	0.000695316	58994	Smpd3
05323	Rheumatoid arthritis	5.019989009	0.000317972	0.000765045	24099	Tnfsf13b
05323	Rheumatoid arthritis	5.019989009	0.000317972	0.000765045	14999	H2-DMb1
05323	Rheumatoid arthritis	5.019989009	0.000317972	0.000765045	14281	Fos
05323	Rheumatoid arthritis	5.019989009	0.000317972	0.000765045	69583	Tnfsf13
05323	Rheumatoid arthritis	5.019989009	0.000317972	0.000765045	12487	Cd28
05323	Rheumatoid arthritis	5.019989009	0.000317972	0.000765045	14998	H2-DMa
05323	Rheumatoid arthritis	5.019989009	0.000317972	0.000765045	242341	Atp6v0d2
05323	Rheumatoid arthritis	5.019989009	0.000317972	0.000765045	14960	H2-Aa
05323	Rheumatoid arthritis	5.019989009	0.000317972	0.000765045	14961	H2-Ab1
04512	ECM-receptor interaction	4.96294368	0.000347767	0.000830489	12830	Col4a5
04512	ECM-receptor interaction	4.96294368	0.000347767	0.000830489	16419	Itgb5
04512	ECM-receptor interaction	4.96294368	0.000347767	0.000830489	16421	Itgb7
04512	ECM-receptor interaction	4.96294368	0.000347767	0.000830489	16774	Lama3
04512	ECM-receptor interaction	4.96294368	0.000347767	0.000830489	241226	Itga8
04512	ECM-receptor interaction	4.96294368	0.000347767	0.000830489	14268	Fn1
04512	ECM-receptor interaction	4.96294368	0.000347767	0.000830489	14723	Gp1ba
04512	ECM-receptor interaction	4.96294368	0.000347767	0.000830489	16403	Itga6

04512	ECM-receptor interaction	4.96294368	0.000347767	0.000830489	64051	Sv2a
05152	Tuberculosis	3.504696031	0.00041322	0.000979484	17533	Mrc1
05152	Tuberculosis	3.504696031	0.00041322	0.000979484	16985	Lsp1
05152	Tuberculosis	3.504696031	0.00041322	0.000979484	14961	H2-Ab1
05152	Tuberculosis	3.504696031	0.00041322	0.000979484	16149	Cd74
05152	Tuberculosis	3.504696031	0.00041322	0.000979484	14998	H2-DMa
05152	Tuberculosis	3.504696031	0.00041322	0.000979484	81897	Tlr9
05152	Tuberculosis	3.504696031	0.00041322	0.000979484	12367	Casp3
05152	Tuberculosis	3.504696031	0.00041322	0.000979484	15979	Ifngr1
05152	Tuberculosis	3.504696031	0.00041322	0.000979484	14960	H2-Aa
05152	Tuberculosis	3.504696031	0.00041322	0.000979484	12265	Ciita
05152	Tuberculosis	3.504696031	0.00041322	0.000979484	242341	Atp6v0d2
05152	Tuberculosis	3.504696031	0.00041322	0.000979484	14999	H2-DMb1
05152	Tuberculosis	3.504696031	0.00041322	0.000979484	12322	Camk2a
00270	Cysteine and methionine metabolism	6.409168358	0.000419515	0.000987093	104923	Adi1
00270	Cysteine and methionine metabolism	6.409168358	0.000419515	0.000987093	70266	Kyat1
00270	Cysteine and methionine metabolism	6.409168358	0.000419515	0.000987093	22117	Tst
00270	Cysteine and methionine metabolism	6.409168358	0.000419515	0.000987093	13433	Dnmt1
00270	Cysteine and methionine metabolism	6.409168358	0.000419515	0.000987093	13435	Dnmt3a
00270	Cysteine and methionine metabolism	6.409168358	0.000419515	0.000987093	14718	Got1
00270	Cysteine and methionine metabolism	6.409168358	0.000419515	0.000987093	16832	Ldhb
04390	Hippo signaling pathway	3.709036466	0.000441731	0.001031781	21812	Tgfbr1
04390	Hippo signaling pathway	3.709036466	0.000441731	0.001031781	23805	Apc2
04390	Hippo signaling pathway	3.709036466	0.000441731	0.001031781	16842	Lef1
04390	Hippo signaling pathway	3.709036466	0.000441731	0.001031781	53310	Dlg3
04390	Hippo signaling pathway	3.709036466	0.000441731	0.001031781	12443	Ccnd1
04390	Hippo signaling pathway	3.709036466	0.000441731	0.001031781	27373	Csnk1e
04390	Hippo signaling pathway	3.709036466	0.000441731	0.001031781	170770	Bbc3
04390	Hippo signaling pathway	3.709036466	0.000441731	0.001031781	17127	Smad3
04390	Hippo signaling pathway	3.709036466	0.000441731	0.001031781	18016	Nf2
04390	Hippo signaling pathway	3.709036466	0.000441731	0.001031781	27494	Amot
04390	Hippo signaling pathway	3.709036466	0.000441731	0.001031781	21813	Tgfbr2
04390	Hippo signaling pathway	3.709036466	0.000441731	0.001031781	12695	Patj
00330	Arginine and proline metabolism	6.290480055	0.000473463	0.001089988	20229	Sat1
00330	Arginine and proline metabolism	6.290480055	0.000473463	0.001089988	67217	L3hypdh
00330	Arginine and proline metabolism	6.290480055	0.000473463	0.001089988	19125	Prodh
00330	Arginine and proline metabolism	6.290480055	0.000473463	0.001089988	18125	Nos1
00330	Arginine and proline metabolism	6.290480055	0.000473463	0.001089988	14718	Got1
00330	Arginine and proline metabolism	6.290480055	0.000473463	0.001089988	12709	Ckb
00330	Arginine and proline metabolism	6.290480055	0.000473463	0.001089988	14431	Gamt
04330	Notch signaling pathway	6.290480055	0.000473463	0.001089988	14357	Dtx1
04330	Notch signaling pathway	6.290480055	0.000473463	0.001089988	17305	Mfng

04330	Notch signaling pathway	6.290480055	0.000473463	0.001089988	68318	Aph1c
04330	Notch signaling pathway	6.290480055	0.000473463	0.001089988	270118	Maml2
04330	Notch signaling pathway	6.290480055	0.000473463	0.001089988	15213	Hey1
04330	Notch signaling pathway	6.290480055	0.000473463	0.001089988	433586	Maml3
04330	Notch signaling pathway	6.290480055	0.000473463	0.001089988	16848	Lfng
04724	Glutamatergic synapse	4.294385878	0.000488766	0.00111718	12289	Cacna1d
04724	Glutamatergic synapse	4.294385878	0.000488766	0.00111718	104111	Adcy3
04724	Glutamatergic synapse	4.294385878	0.000488766	0.00111718	11513	Adcy7
04724	Glutamatergic synapse	4.294385878	0.000488766	0.00111718	18796	Plcb2
04724	Glutamatergic synapse	4.294385878	0.000488766	0.00111718	12286	Cacna1a
04724	Glutamatergic synapse	4.294385878	0.000488766	0.00111718	16440	Itpr3
04724	Glutamatergic synapse	4.294385878	0.000488766	0.00111718	14702	Gng2
04724	Glutamatergic synapse	4.294385878	0.000488766	0.00111718	14697	Gnb5
04724	Glutamatergic synapse	4.294385878	0.000488766	0.00111718	16438	Itpr1
04724	Glutamatergic synapse	4.294385878	0.000488766	0.00111718	66066	Gng11
00980	Metabolism of xenobiotics by cytochrome P450	5.317979225	0.000524288	0.001189872	54486	Hpgds
00980	Metabolism of xenobiotics by cytochrome P450	5.317979225	0.000524288	0.001189872	66447	Mgst3
00980	Metabolism of xenobiotics by cytochrome P450	5.317979225	0.000524288	0.001189872	103140	Gstt3
00980	Metabolism of xenobiotics by cytochrome P450	5.317979225	0.000524288	0.001189872	74134	Cyp2s1
00980	Metabolism of xenobiotics by cytochrome P450	5.317979225	0.000524288	0.001189872	76263	Gstk1
00980	Metabolism of xenobiotics by cytochrome P450	5.317979225	0.000524288	0.001189872	12409	Cbr2
00980	Metabolism of xenobiotics by cytochrome P450	5.317979225	0.000524288	0.001189872	22236	Ugt1a2
00980	Metabolism of xenobiotics by cytochrome P450	5.317979225	0.000524288	0.001189872	13849	Ephx1
04350	TGF-beta signaling pathway	4.597253093	0.000629199	0.001417912	21813	Tgfbr2
04350	TGF-beta signaling pathway	4.597253093	0.000629199	0.001417912	268977	Ltbp1
04350	TGF-beta signaling pathway	4.597253093	0.000629199	0.001417912	68010	Bambi
04350	TGF-beta signaling pathway	4.597253093	0.000629199	0.001417912	21812	Tgfbr1
04350	TGF-beta signaling pathway	4.597253093	0.000629199	0.001417912	15903	Id3
04350	TGF-beta signaling pathway	4.597253093	0.000629199	0.001417912	55994	Smad9
04350	TGF-beta signaling pathway	4.597253093	0.000629199	0.001417912	11481	Acvr2b
04350	TGF-beta signaling pathway	4.597253093	0.000629199	0.001417912	18007	Neo1
04350	TGF-beta signaling pathway	4.597253093	0.000629199	0.001417912	17127	Smad3
04971	Gastric acid secretion	5.176166445	0.00063464	0.001420174	104111	Adcy3
04971	Gastric acid secretion	5.176166445	0.00063464	0.001420174	16440	Itpr3
04971	Gastric acid secretion	5.176166445	0.00063464	0.001420174	16513	Kcnj10
04971	Gastric acid secretion	5.176166445	0.00063464	0.001420174	16518	Kcnj2
04971	Gastric acid secretion	5.176166445	0.00063464	0.001420174	18796	Plcb2
04971	Gastric acid secretion	5.176166445	0.00063464	0.001420174	16438	Itpr1
04971	Gastric acid secretion	5.176166445	0.00063464	0.001420174	12322	Camk2a
04971	Gastric acid secretion	5.176166445	0.00063464	0.001420174	11513	Adcy7
04216	Ferroptosis	7.278984064	0.000639164	0.001420365	53945	Slc40a1
04216	Ferroptosis	7.278984064	0.000639164	0.001420365	67547	Slc39a8

04216	Ferroptosis	7.278984064	0.000639164	0.001420365	22041	Trf
04216	Ferroptosis	7.278984064	0.000639164	0.001420365	68428	Steap3
04216	Ferroptosis	7.278984064	0.000639164	0.001420365	22042	Tfrc
04216	Ferroptosis	7.278984064	0.000639164	0.001420365	20229	Sat1
05020	Prion disease	2.897108085	0.000662254	0.001461527	15511	Hspa1b
05020	Prion disease	2.897108085	0.000662254	0.001461527	18709	Pik3r2
05020	Prion disease	2.897108085	0.000662254	0.001461527	12367	Casp3
05020	Prion disease	2.897108085	0.000662254	0.001461527	16440	Itpr3
05020	Prion disease	2.897108085	0.000662254	0.001461527	18710	Pik3r3
05020	Prion disease	2.897108085	0.000662254	0.001461527	208647	Creb3l2
05020	Prion disease	2.897108085	0.000662254	0.001461527	16438	Itpr1
05020	Prion disease	2.897108085	0.000662254	0.001461527	12289	Cacna1d
05020	Prion disease	2.897108085	0.000662254	0.001461527	193740	Hspa1a
05020	Prion disease	2.897108085	0.000662254	0.001461527	12262	C1qc
05020	Prion disease	2.897108085	0.000662254	0.001461527	12287	Cacna1b
05020	Prion disease	2.897108085	0.000662254	0.001461527	12259	C1qa
05020	Prion disease	2.897108085	0.000662254	0.001461527	16574	Kif5c
05020	Prion disease	2.897108085	0.000662254	0.001461527	16572	Kif5a
05020	Prion disease	2.897108085	0.000662254	0.001461527	74764	Klc4
05020	Prion disease	2.897108085	0.000662254	0.001461527	12260	C1qb
04924	Renin secretion	5.108058992	0.00069659	0.001526774	11555	Adrb2
04924	Renin secretion	5.108058992	0.00069659	0.001526774	12289	Cacna1d
04924	Renin secretion	5.108058992	0.00069659	0.001526774	16438	Itpr1
04924	Renin secretion	5.108058992	0.00069659	0.001526774	18575	Pde1c
04924	Renin secretion	5.108058992	0.00069659	0.001526774	16518	Kcnj2
04924	Renin secretion	5.108058992	0.00069659	0.001526774	16440	Itpr3
04924	Renin secretion	5.108058992	0.00069659	0.001526774	18576	Pde3b
04924	Renin secretion	5.108058992	0.00069659	0.001526774	18796	Plcb2
03440	Homologous recombination	7.101447867	0.000735733	0.001601596	11920	Atm
03440	Homologous recombination	7.101447867	0.000735733	0.001601596	12190	Brca2
03440	Homologous recombination	7.101447867	0.000735733	0.001601596	233826	Palb2
03440	Homologous recombination	7.101447867	0.000735733	0.001601596	12189	Brca1
03440	Homologous recombination	7.101447867	0.000735733	0.001601596	18971	Pold1
03440	Homologous recombination	7.101447867	0.000735733	0.001601596	114714	Rad51c
04530	Tight junction	3.486938474	0.000789331	0.001706663	12443	Ccnd1
04530	Tight junction	3.486938474	0.000789331	0.001706663	68178	Cgnl1
04530	Tight junction	3.486938474	0.000789331	0.001706663	53310	Dlg3
04530	Tight junction	3.486938474	0.000789331	0.001706663	21844	Tiam1
04530	Tight junction	3.486938474	0.000789331	0.001706663	70737	Cgn
04530	Tight junction	3.486938474	0.000789331	0.001706663	12289	Cacna1d
04530	Tight junction	3.486938474	0.000789331	0.001706663	27375	Tjp3
04530	Tight junction	3.486938474	0.000789331	0.001706663	26401	Map3k1

04530	Tight junction	3.486938474	0.000789331	0.001706663	108099	Prkag2
04530	Tight junction	3.486938474	0.000789331	0.001706663	27494	Amot
04530	Tight junction	3.486938474	0.000789331	0.001706663	18016	Nf2
04530	Tight junction	3.486938474	0.000789331	0.001706663	12695	Patj
04913	Ovarian steroidogenesis	5.391840047	0.001259828	0.002705672	11513	Adcy7
04913	Ovarian steroidogenesis	5.391840047	0.001259828	0.002705672	171210	Acot2
04913	Ovarian steroidogenesis	5.391840047	0.001259828	0.002705672	16000	Igf1
04913	Ovarian steroidogenesis	5.391840047	0.001259828	0.002705672	104111	Adcy3
04913	Ovarian steroidogenesis	5.391840047	0.001259828	0.002705672	26897	Acot1
04913	Ovarian steroidogenesis	5.391840047	0.001259828	0.002705672	16001	Igf1r
04913	Ovarian steroidogenesis	5.391840047	0.001259828	0.002705672	11689	Alox5
05203	Viral carcinogenesis	2.966689284	0.001301578	0.002776699	56233	Hdac7
05203	Viral carcinogenesis	2.966689284	0.001301578	0.002776699	18709	Pik3r2
05203	Viral carcinogenesis	2.966689284	0.001301578	0.002776699	15184	Hdac5
05203	Viral carcinogenesis	2.966689284	0.001301578	0.002776699	79221	Hdac9
05203	Viral carcinogenesis	2.966689284	0.001301578	0.002776699	27401	Skp2
05203	Viral carcinogenesis	2.966689284	0.001301578	0.002776699	242341	Atp6v0d2
05203	Viral carcinogenesis	2.966689284	0.001301578	0.002776699	12428	Ccna2
05203	Viral carcinogenesis	2.966689284	0.001301578	0.002776699	12774	Ccr5
05203	Viral carcinogenesis	2.966689284	0.001301578	0.002776699	12367	Casp3
05203	Viral carcinogenesis	2.966689284	0.001301578	0.002776699	19651	Rbl2
05203	Viral carcinogenesis	2.966689284	0.001301578	0.002776699	18710	Pik3r3
05203	Viral carcinogenesis	2.966689284	0.001301578	0.002776699	12443	Ccnd1
05203	Viral carcinogenesis	2.966689284	0.001301578	0.002776699	16195	Il6st
05203	Viral carcinogenesis	2.966689284	0.001301578	0.002776699	208647	Creb3l2
04012	ErbB signaling pathway	4.621577183	0.00139383	0.002953813	56717	Mtor
04012	ErbB signaling pathway	4.621577183	0.00139383	0.002953813	18709	Pik3r2
04012	ErbB signaling pathway	4.621577183	0.00139383	0.002953813	271849	Shc4
04012	ErbB signaling pathway	4.621577183	0.00139383	0.002953813	216148	Shc2
04012	ErbB signaling pathway	4.621577183	0.00139383	0.002953813	17974	Nck2
04012	ErbB signaling pathway	4.621577183	0.00139383	0.002953813	18710	Pik3r3
04012	ErbB signaling pathway	4.621577183	0.00139383	0.002953813	12322	Camk2a
04012	ErbB signaling pathway	4.621577183	0.00139383	0.002953813	18803	Plcg1
00511	Other glycan degradation	10.78368009	0.001745102	0.003673898	217364	Engase
00511	Other glycan degradation	10.78368009	0.001745102	0.003673898	110173	Manba
00511	Other glycan degradation	10.78368009	0.001745102	0.003673898	230101	Gba2
00511	Other glycan degradation	10.78368009	0.001745102	0.003673898	17159	Man2b1
04930	Type II diabetes mellitus	6.065820053	0.001776591	0.003715746	12286	Cacna1a
04930	Type II diabetes mellitus	6.065820053	0.001776591	0.003715746	12289	Cacna1d
04930	Type II diabetes mellitus	6.065820053	0.001776591	0.003715746	12287	Cacna1b
04930	Type II diabetes mellitus	6.065820053	0.001776591	0.003715746	56717	Mtor
04930	Type II diabetes mellitus	6.065820053	0.001776591	0.003715746	18710	Pik3r3

04930	Type II diabetes mellitus	6.065820053	0.001776591	0.003715746	18709	Pik3r2
04727	GABAergic synapse	4.361938016	0.002063386	0.004287555	11513	Adcy7
04727	GABAergic synapse	4.361938016	0.002063386	0.004287555	14697	Gnb5
04727	GABAergic synapse	4.361938016	0.002063386	0.004287555	12289	Cacna1d
04727	GABAergic synapse	4.361938016	0.002063386	0.004287555	104111	Adcy3
04727	GABAergic synapse	4.361938016	0.002063386	0.004287555	14702	Gng2
04727	GABAergic synapse	4.361938016	0.002063386	0.004287555	12287	Cacna1b
04727	GABAergic synapse	4.361938016	0.002063386	0.004287555	66066	Gng11
04727	GABAergic synapse	4.361938016	0.002063386	0.004287555	12286	Cacna1a
04625	C-type lectin receptor signaling pathway	3.899455748	0.002157556	0.00445431	16440	Itpr3
04625	C-type lectin receptor signaling pathway	3.899455748	0.002157556	0.00445431	69810	Clec4b1
04625	C-type lectin receptor signaling pathway	3.899455748	0.002157556	0.00445431	66824	Pycard
04625	C-type lectin receptor signaling pathway	3.899455748	0.002157556	0.00445431	18018	Nfatc1
04625	C-type lectin receptor signaling pathway	3.899455748	0.002157556	0.00445431	16438	Itpr1
04625	C-type lectin receptor signaling pathway	3.899455748	0.002157556	0.00445431	16985	Lsp1
04625	C-type lectin receptor signaling pathway	3.899455748	0.002157556	0.00445431	18709	Pik3r2
04625	C-type lectin receptor signaling pathway	3.899455748	0.002157556	0.00445431	18710	Pik3r3
04625	C-type lectin receptor signaling pathway	3.899455748	0.002157556	0.00445431	18019	Nfatc2
02010	ABC transporters	5.823187251	0.002221625	0.00455718	76408	Abcc3
02010	ABC transporters	5.823187251	0.002221625	0.00455718	27404	Abca8b
02010	ABC transporters	5.823187251	0.002221625	0.00455718	11307	Abcg1
02010	ABC transporters	5.823187251	0.002221625	0.00455718	26874	Abcd2
02010	ABC transporters	5.823187251	0.002221625	0.00455718	76184	Abca6
02010	ABC transporters	5.823187251	0.002221625	0.00455718	217262	Abca9
05140	Leishmaniasis	4.852656042	0.002410204	0.004912517	15979	Ifngr1
05140	Leishmaniasis	4.852656042	0.002410204	0.004912517	14999	H2-DMb1
05140	Leishmaniasis	4.852656042	0.002410204	0.004912517	13628	Eef1a2
05140	Leishmaniasis	4.852656042	0.002410204	0.004912517	14998	H2-DMa
05140	Leishmaniasis	4.852656042	0.002410204	0.004912517	14281	Fos
05140	Leishmaniasis	4.852656042	0.002410204	0.004912517	14961	H2-Ab1
05140	Leishmaniasis	4.852656042	0.002410204	0.004912517	14960	H2-Aa
01040	Biosynthesis of unsaturated fatty acids	7.136258886	0.002499628	0.005062538	66775	Hacd4
01040	Biosynthesis of unsaturated fatty acids	7.136258886	0.002499628	0.005062538	80911	Acox3
01040	Biosynthesis of unsaturated fatty acids	7.136258886	0.002499628	0.005062538	68801	Elovl5
01040	Biosynthesis of unsaturated fatty acids	7.136258886	0.002499628	0.005062538	26897	Acot1
01040	Biosynthesis of unsaturated fatty acids	7.136258886	0.002499628	0.005062538	171210	Acot2
04610	Complement and coagulation cascades	4.174327778	0.00276863	0.005572085	21824	Thbd
04610	Complement and coagulation cascades	4.174327778	0.00276863	0.005572085	12259	C1qa
04610	Complement and coagulation cascades	4.174327778	0.00276863	0.005572085	12267	C3ar1
04610	Complement and coagulation cascades	4.174327778	0.00276863	0.005572085	12262	C1qc
04610	Complement and coagulation cascades	4.174327778	0.00276863	0.005572085	12628	Cfh
04610	Complement and coagulation cascades	4.174327778	0.00276863	0.005572085	19128	Pros1

04610	Complement and coagulation cascades	4.174327778	0.00276863	0.005572085	12260	C1qb
04610	Complement and coagulation cascades	4.174327778	0.00276863	0.005572085	14069	F8
04061	Viral protein interaction with cytokine and cytokine receptor	4.086447194	0.003187663	0.006375326	12772	Ccr2
04061	Viral protein interaction with cytokine and cytokine receptor	4.086447194	0.003187663	0.006375326	12774	Ccr5
04061	Viral protein interaction with cytokine and cytokine receptor	4.086447194	0.003187663	0.006375326	16194	Il6ra
04061	Viral protein interaction with cytokine and cytokine receptor	4.086447194	0.003187663	0.006375326	16195	Il6st
04061	Viral protein interaction with cytokine and cytokine receptor	4.086447194	0.003187663	0.006375326	12766	Cxcr3
04061	Viral protein interaction with cytokine and cytokine receptor	4.086447194	0.003187663	0.006375326	12767	Cxcr4
04061	Viral protein interaction with cytokine and cytokine receptor	4.086447194	0.003187663	0.006375326	16174	Il18rap
04061	Viral protein interaction with cytokine and cytokine receptor	4.086447194	0.003187663	0.006375326	13051	Cx3cr1
00531	Glycosaminoglycan degradation	9.243154367	0.003249315	0.006458266	15586	Hyal1
00531	Glycosaminoglycan degradation	9.243154367	0.003249315	0.006458266	27029	Sgsh
00531	Glycosaminoglycan degradation	9.243154367	0.003249315	0.006458266	15442	Hpse
00531	Glycosaminoglycan degradation	9.243154367	0.003249315	0.006458266	11881	Arsb
04917	Prolactin signaling pathway	4.59035031	0.003370206	0.006616356	18710	Pik3r3
04917	Prolactin signaling pathway	4.59035031	0.003370206	0.006616356	54607	Socs6
04917	Prolactin signaling pathway	4.59035031	0.003370206	0.006616356	12443	Ccnd1
04917	Prolactin signaling pathway	4.59035031	0.003370206	0.006616356	216148	Shc2
04917	Prolactin signaling pathway	4.59035031	0.003370206	0.006616356	18709	Pik3r2
04917	Prolactin signaling pathway	4.59035031	0.003370206	0.006616356	271849	Shc4
04917	Prolactin signaling pathway	4.59035031	0.003370206	0.006616356	14281	Fos
04918	Thyroid hormone synthesis	4.59035031	0.003370206	0.006616356	104111	Adcy3
04918	Thyroid hormone synthesis	4.59035031	0.003370206	0.006616356	16438	Itpr1
04918	Thyroid hormone synthesis	4.59035031	0.003370206	0.006616356	16440	Itpr3
04918	Thyroid hormone synthesis	4.59035031	0.003370206	0.006616356	208647	Creb3l2
04918	Thyroid hormone synthesis	4.59035031	0.003370206	0.006616356	11513	Adcy7
04918	Thyroid hormone synthesis	4.59035031	0.003370206	0.006616356	18796	Plcb2
04918	Thyroid hormone synthesis	4.59035031	0.003370206	0.006616356	11936	Fxyd2
04722	Neurotrophin signaling pathway	3.609413585	0.003766097	0.007348482	12322	Camk2a
04722	Neurotrophin signaling pathway	3.609413585	0.003766097	0.007348482	17179	Matk
04722	Neurotrophin signaling pathway	3.609413585	0.003766097	0.007348482	26401	Map3k1
04722	Neurotrophin signaling pathway	3.609413585	0.003766097	0.007348482	18710	Pik3r3
04722	Neurotrophin signaling pathway	3.609413585	0.003766097	0.007348482	73086	Rps6ka5
04722	Neurotrophin signaling pathway	3.609413585	0.003766097	0.007348482	216148	Shc2
04722	Neurotrophin signaling pathway	3.609413585	0.003766097	0.007348482	18803	Plcg1
04722	Neurotrophin signaling pathway	3.609413585	0.003766097	0.007348482	18709	Pik3r2
04722	Neurotrophin signaling pathway	3.609413585	0.003766097	0.007348482	271849	Shc4
05162	Measles	3.323737015	0.003796419	0.007362752	12367	Casp3
05162	Measles	3.323737015	0.003796419	0.007362752	18709	Pik3r2
05162	Measles	3.323737015	0.003796419	0.007362752	81897	Tlr9
05162	Measles	3.323737015	0.003796419	0.007362752	12443	Ccnd1
05162	Measles	3.323737015	0.003796419	0.007362752	170770	Bbc3

05162	Measles	3.323737015	0.003796419	0.007362752	14281	Fos
05162	Measles	3.323737015	0.003796419	0.007362752	12487	Cd28
05162	Measles	3.323737015	0.003796419	0.007362752	193740	Hspa1a
05162	Measles	3.323737015	0.003796419	0.007362752	18710	Pik3r3
05162	Measles	3.323737015	0.003796419	0.007362752	15511	Hspa1b
04960	Aldosterone-regulated sodium reabsorption	6.38507374	0.004212741	0.008120947	16000	Igf1
04960	Aldosterone-regulated sodium reabsorption	6.38507374	0.004212741	0.008120947	11936	Fxyd2
04960	Aldosterone-regulated sodium reabsorption	6.38507374	0.004212741	0.008120947	18709	Pik3r2
04960	Aldosterone-regulated sodium reabsorption	6.38507374	0.004212741	0.008120947	65962	Slc9a3r2
04960	Aldosterone-regulated sodium reabsorption	6.38507374	0.004212741	0.008120947	18710	Pik3r3
04976	Bile secretion	3.882124834	0.004460748	0.008453418	11513	Adcy7
04976	Bile secretion	3.882124834	0.004460748	0.008453418	320024	Nceh1
04976	Bile secretion	3.882124834	0.004460748	0.008453418	22236	Ugt1a2
04976	Bile secretion	3.882124834	0.004460748	0.008453418	13849	Ephx1
04976	Bile secretion	3.882124834	0.004460748	0.008453418	104111	Adcy3
04976	Bile secretion	3.882124834	0.004460748	0.008453418	20181	Rxra
04976	Bile secretion	3.882124834	0.004460748	0.008453418	11936	Fxyd2
04976	Bile secretion	3.882124834	0.004460748	0.008453418	76408	Abcc3
04071	Sphingolipid signaling pathway	3.522089063	0.004479557	0.008453418	13611	S1pr4
04071	Sphingolipid signaling pathway	3.522089063	0.004479557	0.008453418	67260	Cers4
04071	Sphingolipid signaling pathway	3.522089063	0.004479557	0.008453418	18710	Pik3r3
04071	Sphingolipid signaling pathway	3.522089063	0.004479557	0.008453418	81535	Sgpp1
04071	Sphingolipid signaling pathway	3.522089063	0.004479557	0.008453418	20598	Smpd2
04071	Sphingolipid signaling pathway	3.522089063	0.004479557	0.008453418	18709	Pik3r2
04071	Sphingolipid signaling pathway	3.522089063	0.004479557	0.008453418	13609	S1pr1
04071	Sphingolipid signaling pathway	3.522089063	0.004479557	0.008453418	18796	Plcb2
04071	Sphingolipid signaling pathway	3.522089063	0.004479557	0.008453418	14673	Gna12
05150	Staphylococcus aureus infection	3.522089063	0.004479557	0.008453418	12259	C1qa
05150	Staphylococcus aureus infection	3.522089063	0.004479557	0.008453418	12267	C3ar1
05150	Staphylococcus aureus infection	3.522089063	0.004479557	0.008453418	14999	H2-DMb1
05150	Staphylococcus aureus infection	3.522089063	0.004479557	0.008453418	12260	C1qb
05150	Staphylococcus aureus infection	3.522089063	0.004479557	0.008453418	12628	Cfh
05150	Staphylococcus aureus infection	3.522089063	0.004479557	0.008453418	14998	H2-DMa
05150	Staphylococcus aureus infection	3.522089063	0.004479557	0.008453418	14960	H2-Aa
05150	Staphylococcus aureus infection	3.522089063	0.004479557	0.008453418	12262	C1qc
05150	Staphylococcus aureus infection	3.522089063	0.004479557	0.008453418	14961	H2-Ab1
05144	Malaria	5.108058992	0.004490878	0.008453418	15234	Hgf
05144	Malaria	5.108058992	0.004490878	0.008453418	17057	Klrb1a
05144	Malaria	5.108058992	0.004490878	0.008453418	16971	Lrp1
05144	Malaria	5.108058992	0.004490878	0.008453418	16635	Klra4
05144	Malaria	5.108058992	0.004490878	0.008453418	81897	Tlr9
05144	Malaria	5.108058992	0.004490878	0.008453418	17059	Klrb1c

00515	Mannose type O-glycan biosynthesis	8.439401813	0.004652591	0.008706603	74653	Pomk
00515	Mannose type O-glycan biosynthesis	8.439401813	0.004652591	0.008706603	108902	B4gat1
00515	Mannose type O-glycan biosynthesis	8.439401813	0.004652591	0.008706603	216395	Rxylt1
00515	Mannose type O-glycan biosynthesis	8.439401813	0.004652591	0.008706603	14345	Fut4
05204	Chemical carcinogenesis	3.843687954	0.00475826	0.008852578	20887	Sult1a1
05204	Chemical carcinogenesis	3.843687954	0.00475826	0.008852578	22236	Ugt1a2
05204	Chemical carcinogenesis	3.843687954	0.00475826	0.008852578	103140	Gstt3
05204	Chemical carcinogenesis	3.843687954	0.00475826	0.008852578	66447	Mgst3
05204	Chemical carcinogenesis	3.843687954	0.00475826	0.008852578	54486	Hpgds
05204	Chemical carcinogenesis	3.843687954	0.00475826	0.008852578	70266	Kyat1
05204	Chemical carcinogenesis	3.843687954	0.00475826	0.008852578	13849	Ephx1
05204	Chemical carcinogenesis	3.843687954	0.00475826	0.008852578	76263	Gstk1
04370	VEGF signaling pathway	5.019989009	0.004922697	0.009105567	16542	Kdr
04370	VEGF signaling pathway	5.019989009	0.004922697	0.009105567	18803	Plcg1
04370	VEGF signaling pathway	5.019989009	0.004922697	0.009105567	216148	Shc2
04370	VEGF signaling pathway	5.019989009	0.004922697	0.009105567	18709	Pik3r2
04370	VEGF signaling pathway	5.019989009	0.004922697	0.009105567	18710	Pik3r3
04370	VEGF signaling pathway	5.019989009	0.004922697	0.009105567	18019	Nfatc2
04145	Phagosome	2.932923982	0.005993866	0.011023201	14998	H2-DMa
04145	Phagosome	2.932923982	0.005993866	0.011023201	16419	Itgb5
04145	Phagosome	2.932923982	0.005993866	0.011023201	17533	Mrc1
04145	Phagosome	2.932923982	0.005993866	0.011023201	14999	H2-DMb1
04145	Phagosome	2.932923982	0.005993866	0.011023201	18125	Nos1
04145	Phagosome	2.932923982	0.005993866	0.011023201	226421	Rab7b
04145	Phagosome	2.932923982	0.005993866	0.011023201	242341	Atp6v0d2
04145	Phagosome	2.932923982	0.005993866	0.011023201	140792	Colec12
04145	Phagosome	2.932923982	0.005993866	0.011023201	22042	Tfrc
04145	Phagosome	2.932923982	0.005993866	0.011023201	14961	H2-Ab1
04145	Phagosome	2.932923982	0.005993866	0.011023201	14960	H2-Aa
04064	NF-kappa B signaling pathway	3.697261747	0.00611007	0.0111727	11920	Atm
04064	NF-kappa B signaling pathway	3.697261747	0.00611007	0.0111727	24099	Tnfsf13b
04064	NF-kappa B signaling pathway	3.697261747	0.00611007	0.0111727	108723	Card11
04064	NF-kappa B signaling pathway	3.697261747	0.00611007	0.0111727	171211	Edaradd
04064	NF-kappa B signaling pathway	3.697261747	0.00611007	0.0111727	245527	Eda2r
04064	NF-kappa B signaling pathway	3.697261747	0.00611007	0.0111727	23882	Gadd45g
04064	NF-kappa B signaling pathway	3.697261747	0.00611007	0.0111727	11545	Parp1
04064	NF-kappa B signaling pathway	3.697261747	0.00611007	0.0111727	18803	Plcg1
05134	Legionellosis	4.773104304	0.006410111	0.011629829	53791	Tlr5
05134	Legionellosis	4.773104304	0.006410111	0.011629829	15511	Hspa1b
05134	Legionellosis	4.773104304	0.006410111	0.011629829	66824	Pycard
05134	Legionellosis	4.773104304	0.006410111	0.011629829	193740	Hspa1a
05134	Legionellosis	4.773104304	0.006410111	0.011629829	12367	Casp3

05134	Legionellosis	4.773104304	0.006410111	0.011629829	13628	Eef1a2
05310	Asthma	7.764249668	0.006432749	0.011629829	14999	H2-DMb1
05310	Asthma	7.764249668	0.006432749	0.011629829	14998	H2-DMa
05310	Asthma	7.764249668	0.006432749	0.011629829	14960	H2-Aa
05310	Asthma	7.764249668	0.006432749	0.011629829	14961	H2-Ab1
01212	Fatty acid metabolism	4.696118751	0.00697477	0.012538912	68801	Elovl5
01212	Fatty acid metabolism	4.696118751	0.00697477	0.012538912	52538	Acaa2
01212	Fatty acid metabolism	4.696118751	0.00697477	0.012538912	12894	Cpt1a
01212	Fatty acid metabolism	4.696118751	0.00697477	0.012538912	15107	Hadh
01212	Fatty acid metabolism	4.696118751	0.00697477	0.012538912	66775	Hacd4
01212	Fatty acid metabolism	4.696118751	0.00697477	0.012538912	80911	Acox3
04974	Protein digestion and absorption	3.594560031	0.007310513	0.013069073	12819	Col15a1
04974	Protein digestion and absorption	3.594560031	0.007310513	0.013069073	12815	Col11a2
04974	Protein digestion and absorption	3.594560031	0.007310513	0.013069073	57442	Kcne3
04974	Protein digestion and absorption	3.594560031	0.007310513	0.013069073	20541	Slc8a1
04974	Protein digestion and absorption	3.594560031	0.007310513	0.013069073	20540	Slc7a7
04974	Protein digestion and absorption	3.594560031	0.007310513	0.013069073	12830	Col4a5
04974	Protein digestion and absorption	3.594560031	0.007310513	0.013069073	11936	Fxyd2
04974	Protein digestion and absorption	3.594560031	0.007310513	0.013069073	246049	Slc36a2
05217	Basal cell carcinoma	4.621577183	0.007576342	0.013469053	16576	Kif7
05217	Basal cell carcinoma	4.621577183	0.007576342	0.013469053	27015	Polk
05217	Basal cell carcinoma	4.621577183	0.007576342	0.013469053	23882	Gadd45g
05217	Basal cell carcinoma	4.621577183	0.007576342	0.013469053	107986	Ddb2
05217	Basal cell carcinoma	4.621577183	0.007576342	0.013469053	16842	Lef1
05217	Basal cell carcinoma	4.621577183	0.007576342	0.013469053	23805	Apc2
04080	Neuroactive ligand-receptor interaction	2.304333875	0.007933037	0.014025258	15465	Hrh1
04080	Neuroactive ligand-receptor interaction	2.304333875	0.007933037	0.014025258	19218	Ptger3
04080	Neuroactive ligand-receptor interaction	2.304333875	0.007933037	0.014025258	12802	Cnr2
04080	Neuroactive ligand-receptor interaction	2.304333875	0.007933037	0.014025258	140795	P2ry14
04080	Neuroactive ligand-receptor interaction	2.304333875	0.007933037	0.014025258	18439	P2rx7
04080	Neuroactive ligand-receptor interaction	2.304333875	0.007933037	0.014025258	11555	Adrb2
04080	Neuroactive ligand-receptor interaction	2.304333875	0.007933037	0.014025258	13611	S1pr4
04080	Neuroactive ligand-receptor interaction	2.304333875	0.007933037	0.014025258	11549	Adra1a
04080	Neuroactive ligand-receptor interaction	2.304333875	0.007933037	0.014025258	233571	P2ry6
04080	Neuroactive ligand-receptor interaction	2.304333875	0.007933037	0.014025258	67168	Lpar6
04080	Neuroactive ligand-receptor interaction	2.304333875	0.007933037	0.014025258	18441	P2ry1
04080	Neuroactive ligand-receptor interaction	2.304333875	0.007933037	0.014025258	13609	S1pr1
04080	Neuroactive ligand-receptor interaction	2.304333875	0.007933037	0.014025258	15559	Htr2b
04080	Neuroactive ligand-receptor interaction	2.304333875	0.007933037	0.014025258	12267	C3ar1
04080	Neuroactive ligand-receptor interaction	2.304333875	0.007933037	0.014025258	22354	Vipr1
04080	Neuroactive ligand-receptor interaction	2.304333875	0.007933037	0.014025258	114229	Kiss1r
04080	Neuroactive ligand-receptor interaction	2.304333875	0.007933037	0.014025258	18440	P2rx6

04146	Peroxisome	3.949836314	0.008137529	0.014307743	80911	Acox3
04146	Peroxisome	3.949836314	0.008137529	0.014307743	212503	Paox
04146	Peroxisome	3.949836314	0.008137529	0.014307743	74114	Crot
04146	Peroxisome	3.949836314	0.008137529	0.014307743	22436	Xdh
04146	Peroxisome	3.949836314	0.008137529	0.014307743	76263	Gstk1
04146	Peroxisome	3.949836314	0.008137529	0.014307743	26874	Abcd2
04146	Peroxisome	3.949836314	0.008137529	0.014307743	15926	Idh1
04392	Hippo signaling pathway - multiple species	7.189120063	0.008637826	0.015104395	18016	Nf2
04392	Hippo signaling pathway - multiple species	7.189120063	0.008637826	0.015104395	233651	Dchs1
04392	Hippo signaling pathway - multiple species	7.189120063	0.008637826	0.015104395	27373	Csnk1e
04392	Hippo signaling pathway - multiple species	7.189120063	0.008637826	0.015104395	215653	Rassf2
04260	Cardiac muscle contraction	3.904435896	0.008694701	0.015121218	12298	Cacnb4
04260	Cardiac muscle contraction	3.904435896	0.008694701	0.015121218	319734	Cacna2d4
04260	Cardiac muscle contraction	3.904435896	0.008694701	0.015121218	236727	Slc9a7
04260	Cardiac muscle contraction	3.904435896	0.008694701	0.015121218	11936	Fxyd2
04260	Cardiac muscle contraction	3.904435896	0.008694701	0.015121218	65973	Asph
04260	Cardiac muscle contraction	3.904435896	0.008694701	0.015121218	12289	Cacna1d
04260	Cardiac muscle contraction	3.904435896	0.008694701	0.015121218	20541	Slc8a1
05416	Viral myocarditis	3.860067307	0.009280855	0.01605337	14999	H2-DMb1
05416	Viral myocarditis	3.860067307	0.009280855	0.01605337	14998	H2-DMa
05416	Viral myocarditis	3.860067307	0.009280855	0.01605337	12367	Casp3
05416	Viral myocarditis	3.860067307	0.009280855	0.01605337	14961	H2-Ab1
05416	Viral myocarditis	3.860067307	0.009280855	0.01605337	12487	Cd28
05416	Viral myocarditis	3.860067307	0.009280855	0.01605337	12443	Ccnd1
05416	Viral myocarditis	3.860067307	0.009280855	0.01605337	14960	H2-Aa
04140	Autophagy - animal	3.16477568	0.009387513	0.016150559	97998	Deptor
04140	Autophagy - animal	3.16477568	0.009387513	0.016150559	56717	Mtor
04140	Autophagy - animal	3.16477568	0.009387513	0.016150559	226421	Rab7b
04140	Autophagy - animal	3.16477568	0.009387513	0.016150559	16438	Itpr1
04140	Autophagy - animal	3.16477568	0.009387513	0.016150559	52187	Rragd
04140	Autophagy - animal	3.16477568	0.009387513	0.016150559	18710	Pik3r3
04140	Autophagy - animal	3.16477568	0.009387513	0.016150559	18709	Pik3r2
04140	Autophagy - animal	3.16477568	0.009387513	0.016150559	22241	Ulk1
04140	Autophagy - animal	3.16477568	0.009387513	0.016150559	16001	Igf1r
04664	Fc epsilon RI signaling pathway	4.411505493	0.009617854	0.01645836	18710	Pik3r3
04664	Fc epsilon RI signaling pathway	4.411505493	0.009617854	0.01645836	22325	Vav2
04664	Fc epsilon RI signaling pathway	4.411505493	0.009617854	0.01645836	18803	Plcg1
04664	Fc epsilon RI signaling pathway	4.411505493	0.009617854	0.01645836	11689	Alox5
04664	Fc epsilon RI signaling pathway	4.411505493	0.009617854	0.01645836	18709	Pik3r2
04664	Fc epsilon RI signaling pathway	4.411505493	0.009617854	0.01645836	26399	Map2k6
04972	Pancreatic secretion	3.405372661	0.010265231	0.017472733	67972	Atp2b1
04972	Pancreatic secretion	3.405372661	0.010265231	0.017472733	11936	Fxyd2

04972	Pancreatic secretion	3.405372661	0.010265231	0.017472733	16438	Itpr1
04972	Pancreatic secretion	3.405372661	0.010265231	0.017472733	19340	Rab3d
04972	Pancreatic secretion	3.405372661	0.010265231	0.017472733	18796	Plcb2
04972	Pancreatic secretion	3.405372661	0.010265231	0.017472733	16440	Itpr3
04972	Pancreatic secretion	3.405372661	0.010265231	0.017472733	11513	Adcy7
04972	Pancreatic secretion	3.405372661	0.010265231	0.017472733	104111	Adcy3
05211	Renal cell carcinoma	4.281755332	0.011191782	0.01894905	18591	Pdgfb
05211	Renal cell carcinoma	4.281755332	0.011191782	0.01894905	216805	Flcn
05211	Renal cell carcinoma	4.281755332	0.011191782	0.01894905	15234	Hgf
05211	Renal cell carcinoma	4.281755332	0.011191782	0.01894905	18710	Pik3r3
05211	Renal cell carcinoma	4.281755332	0.011191782	0.01894905	18709	Pik3r2
05211	Renal cell carcinoma	4.281755332	0.011191782	0.01894905	13819	Epas1
04270	Vascular smooth muscle contraction	3.054119188	0.011927353	0.019996214	11549	Adra1a
04270	Vascular smooth muscle contraction	3.054119188	0.011927353	0.019996214	16440	Itpr3
04270	Vascular smooth muscle contraction	3.054119188	0.011927353	0.019996214	12289	Cacna1d
04270	Vascular smooth muscle contraction	3.054119188	0.011927353	0.019996214	18796	Plcb2
04270	Vascular smooth muscle contraction	3.054119188	0.011927353	0.019996214	14673	Gna12
04270	Vascular smooth muscle contraction	3.054119188	0.011927353	0.019996214	51801	Ramp1
04270	Vascular smooth muscle contraction	3.054119188	0.011927353	0.019996214	16438	Itpr1
04270	Vascular smooth muscle contraction	3.054119188	0.011927353	0.019996214	11513	Adcy7
04270	Vascular smooth muscle contraction	3.054119188	0.011927353	0.019996214	104111	Adcy3
00983	Drug metabolism - other enzymes	3.692238293	0.01193524	0.019996214	103140	Gstt3
00983	Drug metabolism - other enzymes	3.692238293	0.01193524	0.019996214	22236	Ugt1a2
00983	Drug metabolism - other enzymes	3.692238293	0.01193524	0.019996214	22017	Tpmt
00983	Drug metabolism - other enzymes	3.692238293	0.01193524	0.019996214	66447	Mgst3
00983	Drug metabolism - other enzymes	3.692238293	0.01193524	0.019996214	20135	Rrm2
00983	Drug metabolism - other enzymes	3.692238293	0.01193524	0.019996214	22436	Xdh
00983	Drug metabolism - other enzymes	3.692238293	0.01193524	0.019996214	110074	Dut
04973	Carbohydrate digestion and absorption	5.054850044	0.012133422	0.020222369	18710	Pik3r3
04973	Carbohydrate digestion and absorption	5.054850044	0.012133422	0.020222369	12289	Cacna1d
04973	Carbohydrate digestion and absorption	5.054850044	0.012133422	0.020222369	18796	Plcb2
04973	Carbohydrate digestion and absorption	5.054850044	0.012133422	0.020222369	18709	Pik3r2
04973	Carbohydrate digestion and absorption	5.054850044	0.012133422	0.020222369	11936	Fxyd2
04940	Type I diabetes mellitus	4.159419465	0.01295086	0.021472928	12487	Cd28
04940	Type I diabetes mellitus	4.159419465	0.01295086	0.021472928	14998	H2-DMa
04940	Type I diabetes mellitus	4.159419465	0.01295086	0.021472928	14999	H2-DMb1
04940	Type I diabetes mellitus	4.159419465	0.01295086	0.021472928	14960	H2-Aa
04940	Type I diabetes mellitus	4.159419465	0.01295086	0.021472928	15893	Ica1
04940	Type I diabetes mellitus	4.159419465	0.01295086	0.021472928	14961	H2-Ab1
05164	Influenza A	2.805003493	0.013299049	0.021936575	14998	H2-DMa
05164	Influenza A	2.805003493	0.013299049	0.021936575	14961	H2-Ab1
05164	Influenza A	2.805003493	0.013299049	0.021936575	12265	Ciita

05164	Influenza A	2.805003493	0.013299049	0.021936575	14999	H2-DMb1
05164	Influenza A	2.805003493	0.013299049	0.021936575	18710	Pik3r3
05164	Influenza A	2.805003493	0.013299049	0.021936575	15979	Ifngr1
05164	Influenza A	2.805003493	0.013299049	0.021936575	66824	Pycard
05164	Influenza A	2.805003493	0.013299049	0.021936575	18709	Pik3r2
05164	Influenza A	2.805003493	0.013299049	0.021936575	14960	H2-Aa
05164	Influenza A	2.805003493	0.013299049	0.021936575	12367	Casp3
00480	Glutathione metabolism	4.100836092	0.013903854	0.02258494	76263	Gstk1
00480	Glutathione metabolism	4.100836092	0.013903854	0.02258494	15926	Idh1
00480	Glutathione metabolism	4.100836092	0.013903854	0.02258494	20135	Rrm2
00480	Glutathione metabolism	4.100836092	0.013903854	0.02258494	54486	Hpgds
00480	Glutathione metabolism	4.100836092	0.013903854	0.02258494	103140	Gstt3
00480	Glutathione metabolism	4.100836092	0.013903854	0.02258494	66447	Mgst3
00982	Drug metabolism - cytochrome P450	4.100836092	0.013903854	0.02258494	14263	Fmo5
00982	Drug metabolism - cytochrome P450	4.100836092	0.013903854	0.02258494	76263	Gstk1
00982	Drug metabolism - cytochrome P450	4.100836092	0.013903854	0.02258494	54486	Hpgds
00982	Drug metabolism - cytochrome P450	4.100836092	0.013903854	0.02258494	22236	Ugt1a2
00982	Drug metabolism - cytochrome P450	4.100836092	0.013903854	0.02258494	66447	Mgst3
00982	Drug metabolism - cytochrome P450	4.100836092	0.013903854	0.02258494	103140	Gstt3
04920	Adipocytokine signaling pathway	4.100836092	0.013903854	0.02258494	20181	Rxra
04920	Adipocytokine signaling pathway	4.100836092	0.013903854	0.02258494	12894	Cpt1a
04920	Adipocytokine signaling pathway	4.100836092	0.013903854	0.02258494	108099	Prkag2
04920	Adipocytokine signaling pathway	4.100836092	0.013903854	0.02258494	100705	Acacb
04920	Adipocytokine signaling pathway	4.100836092	0.013903854	0.02258494	56717	Mtor
04920	Adipocytokine signaling pathway	4.100836092	0.013903854	0.02258494	74551	Pck2
01200	Carbon metabolism	3.130745834	0.017177458	0.027761549	18642	Pfkm
01200	Carbon metabolism	3.130745834	0.017177458	0.027761549	434437	Amt
01200	Carbon metabolism	3.130745834	0.017177458	0.027761549	14718	Got1
01200	Carbon metabolism	3.130745834	0.017177458	0.027761549	68738	Acss1
01200	Carbon metabolism	3.130745834	0.017177458	0.027761549	80911	Acox3
01200	Carbon metabolism	3.130745834	0.017177458	0.027761549	226265	Eno4
01200	Carbon metabolism	3.130745834	0.017177458	0.027761549	110639	Prps2
01200	Carbon metabolism	3.130745834	0.017177458	0.027761549	15926	Idh1
04978	Mineral absorption	4.577977399	0.018691373	0.030056479	22041	Trf
04978	Mineral absorption	4.577977399	0.018691373	0.030056479	11936	Fxyd2
04978	Mineral absorption	4.577977399	0.018691373	0.030056479	20541	Slc8a1
04978	Mineral absorption	4.577977399	0.018691373	0.030056479	67972	Atp2b1
04978	Mineral absorption	4.577977399	0.018691373	0.030056479	53945	Slc40a1
04620	Toll-like receptor signaling pathway	3.39685923	0.0189456	0.030162149	14281	Fos
04620	Toll-like receptor signaling pathway	3.39685923	0.0189456	0.030162149	81897	Tlr9
04620	Toll-like receptor signaling pathway	3.39685923	0.0189456	0.030162149	26399	Map2k6
04620	Toll-like receptor signaling pathway	3.39685923	0.0189456	0.030162149	18710	Pik3r3

04620	Toll-like receptor signaling pathway	3.39685923	0.0189456	0.030162149	170744	Tlr8
04620	Toll-like receptor signaling pathway	3.39685923	0.0189456	0.030162149	53791	Tlr5
04620	Toll-like receptor signaling pathway	3.39685923	0.0189456	0.030162149	18709	Pik3r2
04916	Melanogenesis	3.39685923	0.0189456	0.030162149	104111	Adcy3
04916	Melanogenesis	3.39685923	0.0189456	0.030162149	17311	Kitl
04916	Melanogenesis	3.39685923	0.0189456	0.030162149	11513	Adcy7
04916	Melanogenesis	3.39685923	0.0189456	0.030162149	208647	Creb3l2
04916	Melanogenesis	3.39685923	0.0189456	0.030162149	18796	Plcb2
04916	Melanogenesis	3.39685923	0.0189456	0.030162149	16842	Lef1
04916	Melanogenesis	3.39685923	0.0189456	0.030162149	12322	Camk2a
05100	Bacterial invasion of epithelial cells	3.831044244	0.019468523	0.030841225	140579	Elmo2
05100	Bacterial invasion of epithelial cells	3.831044244	0.019468523	0.030841225	18709	Pik3r2
05100	Bacterial invasion of epithelial cells	3.831044244	0.019468523	0.030841225	18710	Pik3r3
05100	Bacterial invasion of epithelial cells	3.831044244	0.019468523	0.030841225	216148	Shc2
05100	Bacterial invasion of epithelial cells	3.831044244	0.019468523	0.030841225	14268	Fn1
05100	Bacterial invasion of epithelial cells	3.831044244	0.019468523	0.030841225	271849	Shc4
00500	Starch and sucrose metabolism	5.709007109	0.0203688	0.031951059	18605	Enpp1
00500	Starch and sucrose metabolism	5.709007109	0.0203688	0.031951059	27357	Gyg
00500	Starch and sucrose metabolism	5.709007109	0.0203688	0.031951059	76051	Ganc
00500	Starch and sucrose metabolism	5.709007109	0.0203688	0.031951059	110078	Pygb
00640	Propanoate metabolism	5.709007109	0.0203688	0.031951059	16832	Ldhb
00640	Propanoate metabolism	5.709007109	0.0203688	0.031951059	80911	Acox3
00640	Propanoate metabolism	5.709007109	0.0203688	0.031951059	100705	Acacb
00640	Propanoate metabolism	5.709007109	0.0203688	0.031951059	68738	Acss1
04721	Synaptic vesicle cycle	3.781290423	0.020752869	0.032237467	382018	Unc13a
04721	Synaptic vesicle cycle	3.781290423	0.020752869	0.032237467	19339	Rab3a
04721	Synaptic vesicle cycle	3.781290423	0.020752869	0.032237467	12286	Cacna1a
04721	Synaptic vesicle cycle	3.781290423	0.020752869	0.032237467	12287	Cacna1b
04721	Synaptic vesicle cycle	3.781290423	0.020752869	0.032237467	242341	Atp6v0d2
04721	Synaptic vesicle cycle	3.781290423	0.020752869	0.032237467	22249	Unc13b
05133	Pertussis	3.781290423	0.020752869	0.032237467	12259	C1qa
05133	Pertussis	3.781290423	0.020752869	0.032237467	12262	C1qc
05133	Pertussis	3.781290423	0.020752869	0.032237467	12367	Casp3
05133	Pertussis	3.781290423	0.020752869	0.032237467	66824	Pycard
05133	Pertussis	3.781290423	0.020752869	0.032237467	14281	Fos
05133	Pertussis	3.781290423	0.020752869	0.032237467	12260	C1qb
00440	Phosphonate and phosphinate metabolism	16.17552014	0.024097547	0.037252247	13026	Pcyt1a
00440	Phosphonate and phosphinate metabolism	16.17552014	0.024097547	0.037252247	236899	Pcyt1b
05016	Huntington disease	2.249575649	0.024962407	0.038165798	170770	Bbc3
05016	Huntington disease	2.249575649	0.024962407	0.038165798	16572	Kif5a
05016	Huntington disease	2.249575649	0.024962407	0.038165798	56717	Mtor
05016	Huntington disease	2.249575649	0.024962407	0.038165798	74764	Klc4

05016	Huntington disease	2.249575649	0.024962407	0.038165798	22241	Ulk1
05016	Huntington disease	2.249575649	0.024962407	0.038165798	16574	Kif5c
05016	Huntington disease	2.249575649	0.024962407	0.038165798	12367	Casp3
05016	Huntington disease	2.249575649	0.024962407	0.038165798	16513	Kcnj10
05016	Huntington disease	2.249575649	0.024962407	0.038165798	19016	Pparg
05016	Huntington disease	2.249575649	0.024962407	0.038165798	215114	Hip1
05016	Huntington disease	2.249575649	0.024962407	0.038165798	208647	Creb3l2
05016	Huntington disease	2.249575649	0.024962407	0.038165798	18796	Plcb2
05016	Huntington disease	2.249575649	0.024962407	0.038165798	12287	Cacna1b
05016	Huntington disease	2.249575649	0.024962407	0.038165798	16438	Itpr1
00051	Fructose and mannose metabolism	5.391840047	0.025046305	0.038165798	20322	Sord
00051	Fructose and mannose metabolism	5.391840047	0.025046305	0.038165798	18642	Pfkm
00051	Fructose and mannose metabolism	5.391840047	0.025046305	0.038165798	18640	Pfkfb2
00051	Fructose and mannose metabolism	5.391840047	0.025046305	0.038165798	16548	Khk
05340	Primary immunodeficiency	5.391840047	0.025046305	0.038165798	12265	Ciita
05340	Primary immunodeficiency	5.391840047	0.025046305	0.038165798	54167	Icos
05340	Primary immunodeficiency	5.391840047	0.025046305	0.038165798	12504	Cd4
05340	Primary immunodeficiency	5.391840047	0.025046305	0.038165798	11486	Ada
00670	One carbon pool by folate	7.662088488	0.026450507	0.040114513	216188	Aldh1l2
00670	One carbon pool by folate	7.662088488	0.026450507	0.040114513	434437	Amt
00670	One carbon pool by folate	7.662088488	0.026450507	0.040114513	107885	Mthfs
00240	Pyrimidine metabolism	4.183324175	0.027440123	0.041419053	18605	Enpp1
00240	Pyrimidine metabolism	4.183324175	0.027440123	0.041419053	331487	Uprt
00240	Pyrimidine metabolism	4.183324175	0.027440123	0.041419053	110074	Dut
00240	Pyrimidine metabolism	4.183324175	0.027440123	0.041419053	20135	Rrm2
00240	Pyrimidine metabolism	4.183324175	0.027440123	0.041419053	12495	Entpd1
00250	Alanine, aspartate and glutamate metabolism	4.977083121	0.033316044	0.049818384	66514	Asrgl1
00250	Alanine, aspartate and glutamate metabolism	4.977083121	0.033316044	0.049818384	14718	Got1
00250	Alanine, aspartate and glutamate metabolism	4.977083121	0.033316044	0.049818384	269642	Nat8l
00250	Alanine, aspartate and glutamate metabolism	4.977083121	0.033316044	0.049818384	11565	Adssl1
00620	Pyruvate metabolism	4.977083121	0.033316044	0.049818384	68738	Acss1
00620	Pyruvate metabolism	4.977083121	0.033316044	0.049818384	74551	Pck2
00620	Pyruvate metabolism	4.977083121	0.033316044	0.049818384	16832	Ldhb
00620	Pyruvate metabolism	4.977083121	0.033316044	0.049818384	100705	Acacb
04668	TNF signaling pathway	3.006070115	0.036399453	0.054090427	14281	Fos
04668	TNF signaling pathway	3.006070115	0.036399453	0.054090427	18710	Pik3r3
04668	TNF signaling pathway	3.006070115	0.036399453	0.054090427	18709	Pik3r2
04668	TNF signaling pathway	3.006070115	0.036399453	0.054090427	26399	Map2k6
04668	TNF signaling pathway	3.006070115	0.036399453	0.054090427	73086	Rps6ka5
04668	TNF signaling pathway	3.006070115	0.036399453	0.054090427	12367	Casp3
04668	TNF signaling pathway	3.006070115	0.036399453	0.054090427	208647	Creb3l2
05017	Spinocerebellar ataxia	2.753280024	0.036511038	0.054090427	16438	Itpr1

05017	Spinocerebellar ataxia	2.753280024	0.036511038	0.054090427	16440	Itpr3
05017	Spinocerebellar ataxia	2.753280024	0.036511038	0.054090427	18710	Pik3r3
05017	Spinocerebellar ataxia	2.753280024	0.036511038	0.054090427	12286	Cacna1a
05017	Spinocerebellar ataxia	2.753280024	0.036511038	0.054090427	18796	Plcb2
05017	Spinocerebellar ataxia	2.753280024	0.036511038	0.054090427	56717	Mtor
05017	Spinocerebellar ataxia	2.753280024	0.036511038	0.054090427	18709	Pik3r2
05017	Spinocerebellar ataxia	2.753280024	0.036511038	0.054090427	22241	Ulk1
05330	Allograft rejection	3.851314319	0.038710376	0.056822571	14960	H2-Aa
05330	Allograft rejection	3.851314319	0.038710376	0.056822571	14998	H2-DMa
05330	Allograft rejection	3.851314319	0.038710376	0.056822571	14961	H2-Ab1
05330	Allograft rejection	3.851314319	0.038710376	0.056822571	14999	H2-DMb1
05330	Allograft rejection	3.851314319	0.038710376	0.056822571	12487	Cd28
05332	Graft-versus-host disease	3.851314319	0.038710376	0.056822571	14998	H2-DMa
05332	Graft-versus-host disease	3.851314319	0.038710376	0.056822571	12487	Cd28
05332	Graft-versus-host disease	3.851314319	0.038710376	0.056822571	14960	H2-Aa
05332	Graft-versus-host disease	3.851314319	0.038710376	0.056822571	14961	H2-Ab1
05332	Graft-versus-host disease	3.851314319	0.038710376	0.056822571	14999	H2-DMb1
00760	Nicotinate and nicotinamide metabolism	4.734298578	0.039716252	0.05803288	68346	Sirt5
00760	Nicotinate and nicotinamide metabolism	4.734298578	0.039716252	0.05803288	18605	Enpp1
00760	Nicotinate and nicotinamide metabolism	4.734298578	0.039716252	0.05803288	64384	Sirt3
00760	Nicotinate and nicotinamide metabolism	4.734298578	0.039716252	0.05803288	223646	Naprt
00310	Lysine degradation	3.791137533	0.041294616	0.060064896	232811	Kmt5c
00310	Lysine degradation	3.791137533	0.041294616	0.060064896	69726	Smyd3
00310	Lysine degradation	3.791137533	0.041294616	0.060064896	15107	Hadh
00310	Lysine degradation	3.791137533	0.041294616	0.060064896	73251	Setd7
00310	Lysine degradation	3.791137533	0.041294616	0.060064896	192289	Tmlhe
04613	Neutrophil extracellular trap formation	2.367149289	0.042369991	0.06107386	18709	Pik3r2
04613	Neutrophil extracellular trap formation	2.367149289	0.042369991	0.06107386	15184	Hdac5
04613	Neutrophil extracellular trap formation	2.367149289	0.042369991	0.06107386	18796	Plcb2
04613	Neutrophil extracellular trap formation	2.367149289	0.042369991	0.06107386	18710	Pik3r3
04613	Neutrophil extracellular trap formation	2.367149289	0.042369991	0.06107386	170744	Tlr8
04613	Neutrophil extracellular trap formation	2.367149289	0.042369991	0.06107386	56233	Hdac7
04613	Neutrophil extracellular trap formation	2.367149289	0.042369991	0.06107386	79221	Hdac9
04613	Neutrophil extracellular trap formation	2.367149289	0.042369991	0.06107386	14723	Gp1ba
04613	Neutrophil extracellular trap formation	2.367149289	0.042369991	0.06107386	56717	Mtor
04613	Neutrophil extracellular trap formation	2.367149289	0.042369991	0.06107386	18803	Plcg1
05034	Alcoholism	2.367149289	0.042369991	0.06107386	14702	Gng2
05034	Alcoholism	2.367149289	0.042369991	0.06107386	271849	Shc4
05034	Alcoholism	2.367149289	0.042369991	0.06107386	208647	Creb3l2
05034	Alcoholism	2.367149289	0.042369991	0.06107386	71279	Slc29a3
05034	Alcoholism	2.367149289	0.042369991	0.06107386	216148	Shc2
05034	Alcoholism	2.367149289	0.042369991	0.06107386	15184	Hdac5

05034	Alcoholism	2.367149289	0.042369991	0.06107386	79221	Hdac9
05034	Alcoholism	2.367149289	0.042369991	0.06107386	56233	Hdac7
05034	Alcoholism	2.367149289	0.042369991	0.06107386	66066	Gng11
05034	Alcoholism	2.367149289	0.042369991	0.06107386	14697	Gnb5
04670	Leukocyte transendothelial migration	2.878694262	0.045537878	0.065345834	54354	Rassf5
04670	Leukocyte transendothelial migration	2.878694262	0.045537878	0.065345834	22325	Vav2
04670	Leukocyte transendothelial migration	2.878694262	0.045537878	0.065345834	12767	Cxcr4
04670	Leukocyte transendothelial migration	2.878694262	0.045537878	0.065345834	223864	Rapgef3
04670	Leukocyte transendothelial migration	2.878694262	0.045537878	0.065345834	18709	Pik3r2
04670	Leukocyte transendothelial migration	2.878694262	0.045537878	0.065345834	18803	Plcg1
04670	Leukocyte transendothelial migration	2.878694262	0.045537878	0.065345834	18710	Pik3r3
04137	Mitophagy - animal	3.676254578	0.046812228	0.066650449	22241	Ulk1
04137	Mitophagy - animal	3.676254578	0.046812228	0.066650449	226421	Rab7b
04137	Mitophagy - animal	3.676254578	0.046812228	0.066650449	21425	Tfeb
04137	Mitophagy - animal	3.676254578	0.046812228	0.066650449	50873	Prkn
04137	Mitophagy - animal	3.676254578	0.046812228	0.066650449	68943	Pink1
00514	Other types of O-glycan biosynthesis	4.514098644	0.046863597	0.066650449	17305	Mfng
00514	Other types of O-glycan biosynthesis	4.514098644	0.046863597	0.066650449	230145	Galnt12
00514	Other types of O-glycan biosynthesis	4.514098644	0.046863597	0.066650449	16848	Lfng
00514	Other types of O-glycan biosynthesis	4.514098644	0.046863597	0.066650449	20440	St6gal1
05322	Systemic lupus erythematosus	2.62305732	0.047994213	0.067956407	14998	H2-DMa
05322	Systemic lupus erythematosus	2.62305732	0.047994213	0.067956407	14960	H2-Aa
05322	Systemic lupus erythematosus	2.62305732	0.047994213	0.067956407	12487	Cd28
05322	Systemic lupus erythematosus	2.62305732	0.047994213	0.067956407	14999	H2-DMb1
05322	Systemic lupus erythematosus	2.62305732	0.047994213	0.067956407	14961	H2-Ab1
05322	Systemic lupus erythematosus	2.62305732	0.047994213	0.067956407	12262	C1qc
05322	Systemic lupus erythematosus	2.62305732	0.047994213	0.067956407	12259	C1qa
05322	Systemic lupus erythematosus	2.62305732	0.047994213	0.067956407	12260	C1qb
04666	Fc gamma R-mediated phagocytosis	3.16477568	0.048240918	0.068004818	230837	Asap3
04666	Fc gamma R-mediated phagocytosis	3.16477568	0.048240918	0.068004818	18803	Plcg1
04666	Fc gamma R-mediated phagocytosis	3.16477568	0.048240918	0.068004818	16332	Inppl1
04666	Fc gamma R-mediated phagocytosis	3.16477568	0.048240918	0.068004818	22325	Vav2
04666	Fc gamma R-mediated phagocytosis	3.16477568	0.048240918	0.068004818	18710	Pik3r3
04666	Fc gamma R-mediated phagocytosis	3.16477568	0.048240918	0.068004818	18709	Pik3r2
00010	Glycolysis / Gluconeogenesis	3.621385106	0.049750111	0.069519806	74551	Pck2
00010	Glycolysis / Gluconeogenesis	3.621385106	0.049750111	0.069519806	68738	Acss1
00010	Glycolysis / Gluconeogenesis	3.621385106	0.049750111	0.069519806	18642	Pfkm
00010	Glycolysis / Gluconeogenesis	3.621385106	0.049750111	0.069519806	226265	Eno4
00010	Glycolysis / Gluconeogenesis	3.621385106	0.049750111	0.069519806	16832	Ldhb
04720	Long-term potentiation	3.621385106	0.049750111	0.069519806	223864	Rapgef3
04720	Long-term potentiation	3.621385106	0.049750111	0.069519806	16440	Itpr3
04720	Long-term potentiation	3.621385106	0.049750111	0.069519806	18796	Plcb2

04720	Long-term potentiation	3.621385106	0.049750111	0.069519806	12322	Camk2a
04720	Long-term potentiation	3.621385106	0.049750111	0.069519806	16438	Itpr1
04932	Non-alcoholic fatty liver disease	2.570943599	0.053651626	0.07464574	12606	Cebpa
04932	Non-alcoholic fatty liver disease	2.570943599	0.053651626	0.07464574	18709	Pik3r2
04932	Non-alcoholic fatty liver disease	2.570943599	0.053651626	0.07464574	16194	Il6ra
04932	Non-alcoholic fatty liver disease	2.570943599	0.053651626	0.07464574	20181	Rxra
04932	Non-alcoholic fatty liver disease	2.570943599	0.053651626	0.07464574	108099	Prkag2
04932	Non-alcoholic fatty liver disease	2.570943599	0.053651626	0.07464574	18710	Pik3r3
04932	Non-alcoholic fatty liver disease	2.570943599	0.053651626	0.07464574	14281	Fos
04932	Non-alcoholic fatty liver disease	2.570943599	0.053651626	0.07464574	12367	Casp3
01240	Biosynthesis of cofactors	2.520860282	0.059781017	0.082813531	68870	Ak8
01240	Biosynthesis of cofactors	2.520860282	0.059781017	0.082813531	216134	Pdxk
01240	Biosynthesis of cofactors	2.520860282	0.059781017	0.082813531	11565	Adssl1
01240	Biosynthesis of cofactors	2.520860282	0.059781017	0.082813531	107766	Haao
01240	Biosynthesis of cofactors	2.520860282	0.059781017	0.082813531	20148	Dhrs3
01240	Biosynthesis of cofactors	2.520860282	0.059781017	0.082813531	75735	Pank1
01240	Biosynthesis of cofactors	2.520860282	0.059781017	0.082813531	223646	Naprt
01240	Biosynthesis of cofactors	2.520860282	0.059781017	0.082813531	22236	Ugt1a2
00650	Butanoate metabolism	5.199274331	0.07798322	0.107563062	217666	L2hgdh
00650	Butanoate metabolism	5.199274331	0.07798322	0.107563062	67041	Oxct1
00650	Butanoate metabolism	5.199274331	0.07798322	0.107563062	15107	Hadh
05171	Coronavirus disease - COVID-19	2.161101881	0.080149258	0.110076234	12260	C1qb
05171	Coronavirus disease - COVID-19	2.161101881	0.080149258	0.110076234	18803	Plcg1
05171	Coronavirus disease - COVID-19	2.161101881	0.080149258	0.110076234	12262	C1qc
05171	Coronavirus disease - COVID-19	2.161101881	0.080149258	0.110076234	16195	Il6st
05171	Coronavirus disease - COVID-19	2.161101881	0.080149258	0.110076234	18710	Pik3r3
05171	Coronavirus disease - COVID-19	2.161101881	0.080149258	0.110076234	12259	C1qa
05171	Coronavirus disease - COVID-19	2.161101881	0.080149258	0.110076234	12267	C3ar1
05171	Coronavirus disease - COVID-19	2.161101881	0.080149258	0.110076234	16194	Il6ra
05171	Coronavirus disease - COVID-19	2.161101881	0.080149258	0.110076234	14281	Fos
05171	Coronavirus disease - COVID-19	2.161101881	0.080149258	0.110076234	18709	Pik3r2
05171	Coronavirus disease - COVID-19	2.161101881	0.080149258	0.110076234	170744	Tlr8
00071	Fatty acid degradation	3.73281234	0.088979072	0.121162991	80911	Acox3
00071	Fatty acid degradation	3.73281234	0.088979072	0.121162991	52538	Acaa2
00071	Fatty acid degradation	3.73281234	0.088979072	0.121162991	15107	Hadh
00071	Fatty acid degradation	3.73281234	0.088979072	0.121162991	12894	Cpt1a
04340	Hedgehog signaling pathway	3.73281234	0.088979072	0.121162991	16576	Kif7
04340	Hedgehog signaling pathway	3.73281234	0.088979072	0.121162991	12443	Ccnd1
04340	Hedgehog signaling pathway	3.73281234	0.088979072	0.121162991	27373	Csnk1e
04340	Hedgehog signaling pathway	3.73281234	0.088979072	0.121162991	109689	Arrb1
01230	Biosynthesis of amino acids	3.071301293	0.095105944	0.128413089	226265	Eno4
01230	Biosynthesis of amino acids	3.071301293	0.095105944	0.128413089	110639	Prps2

01230	Biosynthesis of amino acids	3.071301293	0.095105944	0.128413089	14718	Got1
01230	Biosynthesis of amino acids	3.071301293	0.095105944	0.128413089	18642	Pfkm
01230	Biosynthesis of amino acids	3.071301293	0.095105944	0.128413089	15926	Idh1
05320	Autoimmune thyroid disease	3.071301293	0.095105944	0.128413089	14961	H2-Ab1
05320	Autoimmune thyroid disease	3.071301293	0.095105944	0.128413089	12487	Cd28
05320	Autoimmune thyroid disease	3.071301293	0.095105944	0.128413089	14999	H2-DMb1
05320	Autoimmune thyroid disease	3.071301293	0.095105944	0.128413089	14998	H2-DMa
05320	Autoimmune thyroid disease	3.071301293	0.095105944	0.128413089	14960	H2-Aa
04714	Thermogenesis	2.109850453	0.120529652	0.162056674	108099	Prkag2
04714	Thermogenesis	2.109850453	0.120529652	0.162056674	23945	Mgll
04714	Thermogenesis	2.109850453	0.120529652	0.162056674	56717	Mtor
04714	Thermogenesis	2.109850453	0.120529652	0.162056674	104111	Adcy3
04714	Thermogenesis	2.109850453	0.120529652	0.162056674	12894	Cpt1a
04714	Thermogenesis	2.109850453	0.120529652	0.162056674	14182	Fgfr1
04714	Thermogenesis	2.109850453	0.120529652	0.162056674	11513	Adcy7
04714	Thermogenesis	2.109850453	0.120529652	0.162056674	208647	Creb3l2
04714	Thermogenesis	2.109850453	0.120529652	0.162056674	19016	Pparg
04714	Thermogenesis	2.109850453	0.120529652	0.162056674	67155	Smarca2
04120	Ubiquitin mediated proteolysis	2.342661538	0.124853521	0.167167894	107568	Wwp1
04120	Ubiquitin mediated proteolysis	2.342661538	0.124853521	0.167167894	26401	Map3k1
04120	Ubiquitin mediated proteolysis	2.342661538	0.124853521	0.167167894	229615	Pias3
04120	Ubiquitin mediated proteolysis	2.342661538	0.124853521	0.167167894	27401	Skp2
04120	Ubiquitin mediated proteolysis	2.342661538	0.124853521	0.167167894	107986	Ddb2
04120	Ubiquitin mediated proteolysis	2.342661538	0.124853521	0.167167894	50873	Prkn
04120	Ubiquitin mediated proteolysis	2.342661538	0.124853521	0.167167894	12189	Brca1
00040	Pentose and glucuronate interconversions	4.159419465	0.140201263	0.186935018	102448	Xylb
00040	Pentose and glucuronate interconversions	4.159419465	0.140201263	0.186935018	22236	Ugt1a2
00040	Pentose and glucuronate interconversions	4.159419465	0.140201263	0.186935018	20322	Sord
04961	Endocrine and other factor-regulated calcium reabsorption	3.182069536	0.148613747	0.197317822	20541	Slc8a1
04961	Endocrine and other factor-regulated calcium reabsorption	3.182069536	0.148613747	0.197317822	67972	Atp2b1
04961	Endocrine and other factor-regulated calcium reabsorption	3.182069536	0.148613747	0.197317822	18796	Plcb2
04961	Endocrine and other factor-regulated calcium reabsorption	3.182069536	0.148613747	0.197317822	11936	Fxyd2
00604	Glycosphingolipid biosynthesis - ganglio series	6.470208057	0.149221603	0.197317822	14421	B4galnt1
00604	Glycosphingolipid biosynthesis - ganglio series	6.470208057	0.149221603	0.197317822	20454	St3gal5
00561	Glycerolipid metabolism	3.130745834	0.156363701	0.205510775	23945	Mgll
00561	Glycerolipid metabolism	3.130745834	0.156363701	0.205510775	218121	Mboat1
00561	Glycerolipid metabolism	3.130745834	0.156363701	0.205510775	110197	Dgkg
00561	Glycerolipid metabolism	3.130745834	0.156363701	0.205510775	13139	Dgka
05012	Parkinson disease	1.964638074	0.156701966	0.205510775	18803	Plcg1
05012	Parkinson disease	1.964638074	0.156701966	0.205510775	16572	Kif5a
05012	Parkinson disease	1.964638074	0.156701966	0.205510775	12322	Camk2a
05012	Parkinson disease	1.964638074	0.156701966	0.205510775	74764	Klc4

05012	Parkinson disease	1.964638074	0.156701966	0.205510775	16574	Kif5c
05012	Parkinson disease	1.964638074	0.156701966	0.205510775	16440	Itpr3
05012	Parkinson disease	1.964638074	0.156701966	0.205510775	12367	Casp3
05012	Parkinson disease	1.964638074	0.156701966	0.205510775	16438	Itpr1
05012	Parkinson disease	1.964638074	0.156701966	0.205510775	68943	Pink1
05012	Parkinson disease	1.964638074	0.156701966	0.205510775	50873	Prkn
04742	Taste transduction	2.637313067	0.167492752	0.218766043	16440	Itpr3
04742	Taste transduction	2.637313067	0.167492752	0.218766043	18796	Plcb2
04742	Taste transduction	2.637313067	0.167492752	0.218766043	12286	Cacna1a
04742	Taste transduction	2.637313067	0.167492752	0.218766043	18575	Pde1c
04742	Taste transduction	2.637313067	0.167492752	0.218766043	18441	P2ry1
00450	Selenocompound metabolism	5.709007109	0.188134422	0.244727704	70266	Kyat1
00450	Selenocompound metabolism	5.709007109	0.188134422	0.244727704	214580	Pstk
00350	Tyrosine metabolism	3.639492032	0.196384723	0.254425552	14718	Got1
00350	Tyrosine metabolism	3.639492032	0.196384723	0.254425552	68636	Fahd1
00350	Tyrosine metabolism	3.639492032	0.196384723	0.254425552	12846	Comt
00830	Retinol metabolism	2.501369094	0.202233851	0.260946904	74134	Cyp2s1
00830	Retinol metabolism	2.501369094	0.202233851	0.260946904	20148	Dhrs3
00830	Retinol metabolism	2.501369094	0.202233851	0.260946904	13086	Cyp2a4
00830	Retinol metabolism	2.501369094	0.202233851	0.260946904	22236	Ugt1a2
00830	Retinol metabolism	2.501369094	0.202233851	0.260946904	241452	Dhrs9
05219	Bladder cancer	3.550723934	0.208753286	0.267294777	12443	Ccnd1
05219	Bladder cancer	3.550723934	0.208753286	0.267294777	242705	E2f2
05219	Bladder cancer	3.550723934	0.208753286	0.267294777	73086	Rps6ka5
00120	Primary bile acid biosynthesis	5.391840047	0.208824044	0.267294777	104086	Cyp27a1
00120	Primary bile acid biosynthesis	5.391840047	0.208824044	0.267294777	13116	Cyp46a1
05031	Amphetamine addiction	2.813133938	0.216946011	0.276584555	12322	Camk2a
05031	Amphetamine addiction	2.813133938	0.216946011	0.276584555	208647	Creb3l2
05031	Amphetamine addiction	2.813133938	0.216946011	0.276584555	14281	Fos
05031	Amphetamine addiction	2.813133938	0.216946011	0.276584555	12289	Cacna1d
04962	Vasopressin-regulated water reabsorption	3.30862912	0.248038524	0.31313022	213575	Dync2li1
04962	Vasopressin-regulated water reabsorption	3.30862912	0.248038524	0.31313022	208647	Creb3l2
04962	Vasopressin-regulated water reabsorption	3.30862912	0.248038524	0.31313022	104111	Adcy3
05014	Amyotrophic lateral sclerosis	1.70960782	0.248293124	0.31313022	56717	Mtor
05014	Amyotrophic lateral sclerosis	1.70960782	0.248293124	0.31313022	56009	Alyref2
05014	Amyotrophic lateral sclerosis	1.70960782	0.248293124	0.31313022	26399	Map2k6
05014	Amyotrophic lateral sclerosis	1.70960782	0.248293124	0.31313022	16572	Kif5a
05014	Amyotrophic lateral sclerosis	1.70960782	0.248293124	0.31313022	16440	Itpr3
05014	Amyotrophic lateral sclerosis	1.70960782	0.248293124	0.31313022	11727	Ang
05014	Amyotrophic lateral sclerosis	1.70960782	0.248293124	0.31313022	22241	Ulk1
05014	Amyotrophic lateral sclerosis	1.70960782	0.248293124	0.31313022	50873	Prkn
05014	Amyotrophic lateral sclerosis	1.70960782	0.248293124	0.31313022	16574	Kif5c

05014	Amyotrophic lateral sclerosis	1.70960782	0.248293124	0.31313022	68943	Pink1
05014	Amyotrophic lateral sclerosis	1.70960782	0.248293124	0.31313022	74764	Klc4
05014	Amyotrophic lateral sclerosis	1.70960782	0.248293124	0.31313022	12367	Casp3
05014	Amyotrophic lateral sclerosis	1.70960782	0.248293124	0.31313022	18125	Nos1
00100	Steroid biosynthesis	4.852656042	0.25246124	0.31313022	13360	Dhcr7
00100	Steroid biosynthesis	4.852656042	0.25246124	0.31313022	16889	Lipa
00220	Arginine biosynthesis	4.852656042	0.25246124	0.31313022	18125	Nos1
00220	Arginine biosynthesis	4.852656042	0.25246124	0.31313022	14718	Got1
00532	Glycosaminoglycan biosynthesis - chondroitin sulfate / dermatan sulfate	4.852656042	0.25246124	0.31313022	53374	Chst3
00532	Glycosaminoglycan biosynthesis - chondroitin sulfate / dermatan sulfate	4.852656042	0.25246124	0.31313022	233781	Xylt1
00770	Pantothenate and CoA biosynthesis	4.852656042	0.25246124	0.31313022	18605	Enpp1
00770	Pantothenate and CoA biosynthesis	4.852656042	0.25246124	0.31313022	75735	Pank1
01210	2-Oxocarboxylic acid metabolism	4.852656042	0.25246124	0.31313022	15926	Idh1
01210	2-Oxocarboxylic acid metabolism	4.852656042	0.25246124	0.31313022	14718	Got1
04964	Proximal tubule bicarbonate reclamation	4.411505493	0.298846917	0.369231713	74551	Pck2
04964	Proximal tubule bicarbonate reclamation	4.411505493	0.298846917	0.369231713	11936	Fxyd2
00510	N-Glycan biosynthesis	2.911593625	0.335908396	0.413425718	140481	Man2a2
00510	N-Glycan biosynthesis	2.911593625	0.335908396	0.413425718	230815	Man1c1
00510	N-Glycan biosynthesis	2.911593625	0.335908396	0.413425718	20440	St6gal1
04621	NOD-like receptor signaling pathway	1.83986959	0.338066346	0.414487474	66824	Pycard
04621	NOD-like receptor signaling pathway	1.83986959	0.338066346	0.414487474	195046	Nlrp1a
04621	NOD-like receptor signaling pathway	1.83986959	0.338066346	0.414487474	637515	Nlrp1b
04621	NOD-like receptor signaling pathway	1.83986959	0.338066346	0.414487474	56338	Txnip
04621	NOD-like receptor signaling pathway	1.83986959	0.338066346	0.414487474	16438	Itpr1
04621	NOD-like receptor signaling pathway	1.83986959	0.338066346	0.414487474	18796	Plcb2
04621	NOD-like receptor signaling pathway	1.83986959	0.338066346	0.414487474	18439	P2rx7
04621	NOD-like receptor signaling pathway	1.83986959	0.338066346	0.414487474	16440	Itpr3
00534	Glycosaminoglycan biosynthesis - heparan sulfate / heparin	4.043880035	0.347684581	0.424652923	15531	Ndst1
00534	Glycosaminoglycan biosynthesis - heparan sulfate / heparin	4.043880035	0.347684581	0.424652923	233781	Xylt1
00380	Tryptophan metabolism	2.799609255	0.367770625	0.447477566	15107	Hadh
00380	Tryptophan metabolism	2.799609255	0.367770625	0.447477566	70266	Kyat1
00380	Tryptophan metabolism	2.799609255	0.367770625	0.447477566	107766	Haao
00563	Glycosylphosphatidylinositol (GPI)-anchor biosynthesis	3.882124834	0.372935639	0.452043199	241062	Pgap1
00563	Glycosylphosphatidylinositol (GPI)-anchor biosynthesis	3.882124834	0.372935639	0.452043199	239827	Pigz
04141	Protein processing in endoplasmic reticulum	1.974918157	0.376897077	0.455120999	50873	Prkn
04141	Protein processing in endoplasmic reticulum	1.974918157	0.376897077	0.455120999	75744	Svip
04141	Protein processing in endoplasmic reticulum	1.974918157	0.376897077	0.455120999	12334	Capn2
04141	Protein processing in endoplasmic reticulum	1.974918157	0.376897077	0.455120999	67397	Erp29
04141	Protein processing in endoplasmic reticulum	1.974918157	0.376897077	0.455120999	193740	Hspa1a
04141	Protein processing in endoplasmic reticulum	1.974918157	0.376897077	0.455120999	230815	Man1c1
04141	Protein processing in endoplasmic reticulum	1.974918157	0.376897077	0.455120999	15511	Hspa1b
03018	RNA degradation	2.310788592	0.382938359	0.460677725	18642	Pfkm

03018	RNA degradation	2.310788592	0.382938359	0.460677725	226265	Eno4
03018	RNA degradation	2.310788592	0.382938359	0.460677725	22057	Tob1
03018	RNA degradation	2.310788592	0.382938359	0.460677725	231464	Cnot6l
04217	Necroptosis	1.952217948	0.385548397	0.462080476	66824	Pycard
04217	Necroptosis	1.952217948	0.385548397	0.462080476	12322	Camk2a
04217	Necroptosis	1.952217948	0.385548397	0.462080476	78779	Spata2l
04217	Necroptosis	1.952217948	0.385548397	0.462080476	15979	Ifngr1
04217	Necroptosis	1.952217948	0.385548397	0.462080476	110078	Pygb
04217	Necroptosis	1.952217948	0.385548397	0.462080476	11545	Parp1
04217	Necroptosis	1.952217948	0.385548397	0.462080476	12334	Capn2
00590	Arachidonic acid metabolism	2.257049322	0.408572822	0.487848146	12409	Cbr2
00590	Arachidonic acid metabolism	2.257049322	0.408572822	0.487848146	54486	Hpgds
00590	Arachidonic acid metabolism	2.257049322	0.408572822	0.487848146	11689	Alox5
00590	Arachidonic acid metabolism	2.257049322	0.408572822	0.487848146	19224	Ptgs1
00601	Glycosphingolipid biosynthesis - lacto and neolacto series	3.594560031	0.424937897	0.505502331	14347	Fut7
00601	Glycosphingolipid biosynthesis - lacto and neolacto series	3.594560031	0.424937897	0.505502331	14345	Fut4
00512	Mucin type O-glycan biosynthesis	3.466182887	0.451626718	0.534359799	14537	Gcnt1
00512	Mucin type O-glycan biosynthesis	3.466182887	0.451626718	0.534359799	230145	Galnt12
00280	Valine, leucine and isoleucine degradation	2.554029496	0.452535955	0.534359799	52538	Acaa2
00280	Valine, leucine and isoleucine degradation	2.554029496	0.452535955	0.534359799	15107	Hadh
00280	Valine, leucine and isoleucine degradation	2.554029496	0.452535955	0.534359799	67041	Oxct1
00232	Caffeine metabolism	8.087760071	0.469799537	0.552705337	22436	Xdh
00020	Citrate cycle (TCA cycle)	3.032910027	0.56228798	0.651928093	74551	Pck2
00020	Citrate cycle (TCA cycle)	3.032910027	0.56228798	0.651928093	15926	Idh1
00052	Galactose metabolism	3.032910027	0.56228798	0.651928093	18642	Pfkm
00052	Galactose metabolism	3.032910027	0.56228798	0.651928093	76051	Ganc
00630	Glyoxylate and dicarboxylate metabolism	3.032910027	0.56228798	0.651928093	68738	Acss1
00630	Glyoxylate and dicarboxylate metabolism	3.032910027	0.56228798	0.651928093	434437	Amt
04215	Apoptosis - multiple species	3.032910027	0.56228798	0.651928093	170770	Bbc3
04215	Apoptosis - multiple species	3.032910027	0.56228798	0.651928093	12367	Casp3
00030	Pentose phosphate pathway	2.941003662	0.590794351	0.682506109	110639	Prps2
00030	Pentose phosphate pathway	2.941003662	0.590794351	0.682506109	18642	Pfkm
00740	Riboflavin metabolism	6.065820053	0.613808676	0.704009951	18605	Enpp1
04122	Sulfur relay system	6.065820053	0.613808676	0.704009951	22117	Tst
05160	Hepatitis C	1.764602197	0.636850781	0.727829464	242705	E2f2
05160	Hepatitis C	1.764602197	0.636850781	0.727829464	18710	Pik3r3
05160	Hepatitis C	1.764602197	0.636850781	0.727829464	12367	Casp3
05160	Hepatitis C	1.764602197	0.636850781	0.727829464	12443	Ccnd1
05160	Hepatitis C	1.764602197	0.636850781	0.727829464	20181	Rxra
05160	Hepatitis C	1.764602197	0.636850781	0.727829464	18709	Pik3r2
00400	Phenylalanine, tyrosine and tryptophan biosynthesis	5.391840047	0.683596479	0.775712317	14718	Got1
00750	Vitamin B6 metabolism	5.391840047	0.683596479	0.775712317	216134	Pdxk

05143	African trypanosomiasis	2.48854156	0.767024108	0.867306411	81897	Tlr9
05143	African trypanosomiasis	2.48854156	0.767024108	0.867306411	18796	Plcb2
00260	Glycine, serine and threonine metabolism	2.426328021	0.797057759	0.891812878	14431	Gamt
00260	Glycine, serine and threonine metabolism	2.426328021	0.797057759	0.891812878	434437	Amt
00513	Various types of N-glycan biosynthesis	2.426328021	0.797057759	0.891812878	230815	Man1c1
00513	Various types of N-glycan biosynthesis	2.426328021	0.797057759	0.891812878	140481	Man2a2
05033	Nicotine addiction	2.426328021	0.797057759	0.891812878	12287	Cacna1b
05033	Nicotine addiction	2.426328021	0.797057759	0.891812878	12286	Cacna1a
00072	Synthesis and degradation of ketone bodies	4.043880035	0.884453469	0.98615021	67041	Oxct1
00430	Taurine and hypotaurine metabolism	3.73281234	0.948666926	1	246277	Csad
03450	Non-homologous end-joining	3.73281234	0.948666926	1	19090	Prkdc
00565	Ether lipid metabolism	2.021940018	1	1	210992	Lpcat1
00565	Ether lipid metabolism	2.021940018	1	1	104759	Pld4
00730	Thiamine metabolism	3.235104028	1	1	68870	Ak8
03010	Ribosome	0.271098103	1	1	121022	Mrps6
00910	Nitrogen metabolism	2.854503554	1	1	56078	Car5b
00061	Fatty acid biosynthesis	2.554029496	1	1	100705	Acacb
04623	Cytosolic DNA-sensing pathway	1.540525728	1	1	66824	Pycard
04623	Cytosolic DNA-sensing pathway	1.540525728	1	1	67486	Polr3g
00360	Phenylalanine metabolism	2.109850453	1	1	14718	Got1
00900	Terpenoid backbone biosynthesis	2.109850453	1	1	66881	Pcyox1
00970	Aminoacyl-tRNA biosynthesis	1.470501831	1	1	214580	Pstk
00970	Aminoacyl-tRNA biosynthesis	1.470501831	1	1	272396	Tarsl2
00592	alpha-Linolenic acid metabolism	1.941062417	1	1	80911	Acox3
00340	Histidine metabolism	1.86640617	1	1	71761	Amdhd1
04950	Maturity onset diabetes of the young	1.797280016	1	1	15242	Hhex
04966	Collecting duct acid secretion	1.797280016	1	1	242341	Atp6v0d2
04657	IL-17 signaling pathway	1.599776717	1	1	12367	Casp3
04657	IL-17 signaling pathway	1.599776717	1	1	22032	Traf4
04657	IL-17 signaling pathway	1.599776717	1	1	14281	Fos
03020	RNA polymerase	1.67332967	1	1	67486	Polr3g
01523	Antifolate resistance	1.617552014	1	1	76408	Abcc3
00053	Ascorbate and aldarate metabolism	1.565372917	1	1	22236	Ugt1a2
00410	beta-Alanine metabolism	1.565372917	1	1	80911	Acox3
04136	Autophagy - other	1.516455013	1	1	56717	Mtor
04130	SNARE interactions in vesicular transport	1.470501831	1	1	22317	Vamp1
00190	Oxidative phosphorylation	0.364861357	1	1	242341	Atp6v0d2
00860	Porphyrin and chlorophyll metabolism	1.155394296	1	1	22236	Ugt1a2
03013	RNA transport	0.533258906	1	1	13628	Eef1a2
03013	RNA transport	0.533258906	1	1	56009	Alyref2
03022	Basal transcription factors	1.102876373	1	1	14886	Gtf2i
03050	Proteasome	1.032480009	1	1	621823	Psme2b

00140	Steroid hormone biosynthesis	1.054925227	1	1	12846	Comt
00140	Steroid hormone biosynthesis	1.054925227	1	1	22236	Ugt1a2
03015	mRNA surveillance pathway	0.475750592	1	1	56009	Alyref2
03040	Spliceosome	1.086415532	1	1	56009	Alyref2
03040	Spliceosome	1.086415532	1	1	193740	Hspa1a
03040	Spliceosome	1.086415532	1	1	15511	Hspa1b
00520	Amino sugar and nucleotide sugar metabolism	0.970531208	1	1	26384	Gnpda1
04622	RIG-I-like receptor signaling pathway	0.693236577	1	1	26401	Map3k1
05030	Cocaine addiction	1.010970009	1	1	208647	Creb3l2
