# Supplementary material for: Guanylate-Binding protein 2b regulates the AMPK/mTOR/ULK1 signalling pathway to induce autophagy during Mycobacterium bovis infection
Source: Virulence. 2022 May 21;13(1):875–89. doi: 10.1080/21505594.2022.2073024 (PMC9132469; doi:10.1080/21505594.2022.2073024)
Supplement: Supplemental Material [file KVIR_A_2073024_SM9990.zip › Supplementary table 4.pdf]

st_gene_id	gene_id	gene_symbol	log2FoldChange	pvalue	padj	count_M0_6h_Uninfection1_normCounts	count_M0_6h_Uninfection2_normCounts	count_M0_6h_Uninfection3_normCounts	count_M0_6h_Infection1_normCounts	count_M0_6h_Infection2_normCounts	count_M0_6h_Infection3_normCounts
G10090_10059	171171	Ntng2	4.796772857	6.02859E-22	2.80512E-20	9.36878817	8.859664247	3.068204334	256.3878681	217.1748148	125.3637821
G10090_10511	16878	Lif	6.792319161	1.39401E-21	6.24203E-20	1.873757634	2.657899274	1.022734778	173.503859	305.4655106	147.1211327
G10090_10574	20310	Cxcl2	7.728635238	7.08503E-60	2.98828E-57	479.6819543	298.5706851	111.4780908	87493.46511	37329.7121	63963.50261
G10090_11386	14468	Gbp2b	5.419889977	2.34853E-29	1.91919E-27	9.36878817	3.543865699	3.068204334	197.8165017	195.8632676	293.2062011
G10090_12526	18124	Nr4a3	5.575814177	6.76296E-24	3.57999E-22	4.684394085	4.429832124	3.068204334	233.1803456	234.4270197	117.0752676
G10090_13592	16176	Il1b	7.937315434	6.50351E-18	2.05395E-16	183.6282481	30.12285844	42.95486068	31944.60222	19240.26782	11741.71688
G10090_13780	13655	Egr3	5.962548746	3.11832E-23	1.5989E-21	1.873757634	5.315798548	5.113673891	320.4848351	319.6732087	126.3998464
G10090_15116	20303	Ccl4	6.407171583	1.56024E-85	1.27501E-82	68.39215364	81.50891108	161.5920949	11057.83193	8148.114898	7169.565059
G10090_16409	320910	Itgb8	4.554669594	3.75433E-19	1.33029E-17	8.431909353	9.745630672	16.36375645	296.1721925	128.8841191	382.3077322
G10090_16411	19225	Ptgs2	7.480746634	1.21135E-83	9.31672E-81	278.2530086	108.0879038	123.7509082	37612.76332	19369.15194	34138.31918
G10090_18702	12703	Socs1	5.522116054	1.66266E-61	7.24644E-59	23.42197042	29.23689202	10.22734778	1209.001412	893.0553133	816.4186802
G10090_18998	20296	Ccl2	6.500224483	7.50396E-18	2.32498E-16	8.431909353	43.41235481	19.43196078	1737.24883	3442.322299	1284.719751
G10090_19061	14609	Gja1	5.935982589	1.06732E-39	1.64179E-37	24.35884924	14.1754628	8.181878225	1254.311337	1101.096608	516.9960932
G10090_20164	22271	Upp1	5.557976131	4.92949E-17	1.45165E-15	13.11630344	24.80705989	5.113673891	849.8373731	1007.731734	182.3473194
G10090_20212	19419	Rasgrp1	5.378062029	6.3174E-101	1.65199E-97	31.85387978	31.89479129	26.59110423	1274.2035	994.5388717	1497.112935
G10090_20420	20302	Ccl3	6.394034529	4.6278E-219	3.0255E-215	628.6456862	726.4924683	904.0975439	69801.59709	49952.23708	70195.42947
G10090_2060	330122	Cxcl3	7.617987278	1.67522E-21	7.37489E-20	793.536358	243.6407668	150.3420124	112807.3466	58576.30987	61914.1674
G10090_21609	12702	Socs3	5.236146723	0	0	237.0303407	225.0354719	187.1604644	8670.77247	8244.524279	7588.135043
G10090_21977	12494	Cd38	5.679330296	2.54338E-52	7.91778E-50	30.91700096	61.13168331	25.56836945	2135.092074	2610.15712	1308.54923
G10090_22095	14825	Cxcl1	8.540039366	4.27091E-89	4.65351E-86	156.4587624	71.7632804	45.00033024	44782.78267	21834.18758	35172.31136
G10090_2280	108078	Olr1	5.796263961	6.91415E-45	1.36974E-42	6.558151719	9.745630672	3.068204334	402.2637241	289.2281412	405.1011471
G10090_23580	229898	Gbp5	5.826194276	3.0122E-51	8.2051E-49	97.43539696	41.64042196	61.36408669	3751.882811	2219.445421	5394.786888
G10090_25739	12700	Cish	6.974157939	5.63343E-34	6.13809E-32	2.810636451	6.201764973	1.022734778	498.4091746	534.8183524	262.1242717
G10090_26551	18126	Nos2	5.832000555	1.47269E-46	3.37814E-44	82.44533589	124.9212659	33.75024768	3234.686594	5054.896041	5480.780226
G10090_29396	21664	Phlda1	4.880201783	2.90617E-32	2.81468E-30	69.32903246	52.27201906	27.61383901	2277.65257	983.3756802	1142.778939
G10090_29841	18788	Serpinb2	7.260448986	9.55658E-13	1.70235E-11	197.6814304	70.87731398	16.36375645	25859.81083	13221.27798	4616.702587
G10090_30722	20471	Six1	4.832229972	8.97224E-12	1.3916E-10	2.810636451	0.885966425	4.090939112	98.35569078	45.66760125	73.56056636
G10090_30788	20311	Cxcl5	8.402037732	1.17889E-18	3.9523E-17	14.05318225	2.657899274	4.090939112	4579.617782	1483.689623	974.9365204
G10090_32309	26464	Vnn3	6.115429515	4.52881E-22	2.1611E-20	1.873757634	5.315798548	2.045469556	245.3366669	287.1984701	118.1113319
G10090_32612	12044	Bcl2a1a	6.235496665	7.91346E-67	4.31119E-64	20.61133397	18.60529492	4.090939112	1297.411022	984.3905158	1025.703672
G10090_3471	13838	Epha4	4.677783851	1.32124E-12	2.32193E-11	1.873757634	8.859664247	2.045469556	77.35840848	163.3885289	92.20972403
G10090_4537	16175	Il1a	9.253197494	1.26425E-27	8.83958E-26	29.04324333	23.03512704	7.159143447	18515.18251	14341.65646	3362.028702
G10090_545	20306	Ccl7	6.96682846	4.69523E-35	5.43276E-33	3.747515268	14.1754628	8.181878225	787.9506463	1921.083759	572.9435662
G10090_6461	70355	Gprc5c	-5.256303388	1.23068E-19	4.59747E-18	476.8713178	310.0882487	599.32258	1.105120121	14.20769817	20.7212863
G10090_69	11876	Artn	4.851560942	1.51148E-21	6.69917E-20	7.495030536	7.087731398	2.045469556	209.972823	161.3588577	116.0392033

st_gene_id	count_M0_6h_Uninfection1_Counts	count_M0_6h_Uninfection2_Counts	count_M0_6h_Uninfection3_Counts	count_M0_6h_Infection1_Counts	count_M0_6h_Infection2_Counts	count_M0_6h_Infection3_Counts	style	foldchange	gene_id (#1)	gene_symbol (#1)	log2FoldChange (#1)	pvalue (#1)	padj (#1)
G10090_10059	10	10	3	232	214	121	up	27.79537337	171171	Ntng2	5.156351	9.87214E-33	7.84105E-31
G10090_10511	2	3	1	157	301	142	up	110.8387981	16878	Lif	5.747966	3.21996E-06	1.9664E-05
G10090_10574	512	337	109	79171	36784	61737	up	212.1050615	20310	Cxcl2	6.371197	1.22365E-36	1.26347E-34
G10090_11386	10	4	3	179	193	283	up	42.81041682	14468	Gbp2b	4.699349	1.13378E-18	2.92107E-17
G10090_12526	5	5	3	211	231	113	up	47.69658854	18124	Nr4a3	4.619926	1.98473E-12	2.8132E-11
G10090_13592	196	34	42	28906	18959	11333	up	245.1150799	16176	Il1b	9.071354	8.46484E-39	9.96698E-37
G10090_13780	2	6	5	290	315	122	up	62.35998801	13655	Egr3	5.096888	1.79885E-16	3.74356E-15
G10090_15116	73	92	158	10006	8029	6920	up	84.86934178	20303	Ccl4	6.27465	3.49113E-85	3.34725E-82
G10090_16409	9	11	16	268	127	369	up	23.50131525	320910	Itgb8	5.795454	3.52468E-31	2.45139E-29
G10090_16411	297	122	121	34035	19086	32950	up	178.6196059	19225	Ptgs2	7.46468	7.93213E-20	2.27333E-18
G10090_18702	25	33	10	1094	880	788	up	45.95392084	12703	Socs1	6.364196	2.1893E-102	3.26527E-99
G10090_18998	9	49	19	1572	3392	1240	up	90.52375239	20296	Ccl2	7.161767	2.00207E-25	8.95791E-24
G10090_19061	26	16	8	1135	1085	499	up	61.22218327	14609	Gja1	5.330286	5.3272E-10	5.67748E-09
G10090_20164	14	28	5	769	993	176	up	47.11048016	22271	Upp1	6.557964	4.55214E-29	2.74006E-27
G10090_20212	34	36	26	1153	980	1445	up	41.587038	19419	Rasgrp1	5.381283	5.2629E-210	3.5322E-206
G10090_20420	671	820	884	63162	49222	67752	up	84.10003693	20302	Ccl3	5.610276	8.3516E-199	2.8026E-195
G10090_2060	847	275	147	1E+05	57720	59759	up	196.445768	330122	Cxcl3	8.425283	1.69976E-32	1.3265E-30
G10090_21609	253	254	183	7846	8124	7324	up	37.69096225	12702	Socs3	4.839292	4.9649E-234	6.6643E-230
G10090_21977	33	69	25	1932	2572	1263	up	51.24467868	12494	Cd38	6.83172	2.04117E-27	1.06609E-25
G10090_22095	167	81	44	40523	21515	33948	up	372.227121	14825	Cxcl1	7.09471	1.73892E-35	1.65543E-33
G10090_2280	7	11	3	364	285	391	up	55.57114114	108078	Olr1	4.840947	1.73063E-37	1.91985E-35
G10090_23580	104	47	60	3395	2187	5207	up	56.73606843	229898	Gbp5	4.602766	2.18752E-68	1.22346E-65
G10090_25739	3	7	1	451	527	253	up	125.7276315	12700	Cish	7.178797	5.56069E-31	3.82775E-29
G10090_26551	88	141	33	2927	4981	5290	up	56.96486884	18126	Nos2	7.760107	3.92548E-34	3.46656E-32
G10090_29396	74	59	27	2061	969	1103	up	29.45012358	21664	Phlda1	4.834092	9.89066E-37	1.04537E-34
G10090_29841	211	80	16	23400	13028	4456	up	153.3249836	18788	Serpinb2	8.484087	4.35698E-18	1.05949E-16
G10090_30722	3	1	4	89	45	71	up	28.48696404	20471	Six1	5.191338	1.021E-22	3.65464E-21
G10090_30788	15	3	4	4144	1462	941	up	338.2714789	20311	Cxcl5	8.695299	7.1552E-08	5.73741E-07
G10090_32309	2	6	2	222	283	114	up	69.33104089	26464	Vnn3	4.772175	1.00034E-26	4.95483E-25
G10090_32612	22	21	4	1174	970	990	up	75.34796617	12044	Bcl2a1a	6.360086	4.30804E-51	9.17886E-49
G10090_3471	2	10	2	70	161	89	up	25.59488911	13838	Epha4	4.901958	1.8407E-15	3.41738E-14
G10090_4537	31	26	7	16754	14132	3245	up	610.2250078	16175	Il1a	10.13748	2.8397E-154	7.6235E-151
G10090_545	4	16	8	713	1893	553	up	125.0905036	20306	Ccl7	8.054046	1.30411E-15	2.47597E-14
G10090_6461	509	350	586	1	14	20	down	0.02616345	70355	Gprc5c	-5.33949	7.26817E-54	1.87617E-51
G10090_69	8	8	2	190	159	112	up	28.8712355	11876	Artn	6.271072	1.6494E-25	7.42951E-24

st_gene_id	count_M0_24h_Uninfection1_normCounts	count_M0_24h_Uninfection2_normCounts	count_M0_24h_Uninfection3_normCounts	count_M0_24h_Infection1_normCounts	count_M0_24h_Infection2_normCounts	count_M0_24h_Infection3_normCounts	count_M0_24h_Uninfection1_Counts	count_M0_24h_Uninfection2_Counts	count_M0_24h_Uninfection3_Counts	count_M0_24h_Infection1_Counts	count_M0_24h_Infection2_Counts	count_M0_24h_Infection3_Counts	foldchange (#1)
G10090_10059	6.72908245	24.32928664	8.628204079	595.3546175	337.7230541	486.612081	7	26	9	559	322	474	35.66286976
G10090_10511	3.845189972	10.29315973	0	154.4300886	480.3638471	126.2727552	4	11	0	145	458	123	53.74154793
G10090_10574	310.4990902	175.9194572	94.91024487	19595.58069	16305.10124	12219.71224	323	188	99	18399	15546	11903	82.77926274
G10090_11386	2.883892479	9.357417937	5.752136053	109.6986147	231.7912887	127.299363	3	10	6	103	221	124	25.98034474
G10090_12526	4.806487465	6.550192556	0.958689342	68.16224601	161.5197215	73.91575914	5	7	1	64	154	72	24.58874381
G10090_13592	200.911176	94.50992116	29.71936961	69240.06152	64700.18559	40964.72968	209	101	31	65012	61688	39903	537.9597014
G10090_13780	1.922594986	6.550192556	5.752136053	132.0643516	267.4514869	88.28826787	2	7	6	124	255	86	34.22286112
G10090_15116	27.87762729	36.49392995	39.30626303	2354.792593	3907.938197	1765.765357	29	39	41	2211	3726	1720	77.42082575
G10090_16409	2.883892479	2.807225381	4.793446711	223.6573697	181.4474794	176.5765357	3	3	5	210	173	172	55.53996467
G10090_16411	113.4331042	49.59431506	12.46296145	11053.99924	12643.63794	7299.181216	118	53	13	10379	12055	7110	176.6414374
G10090_18702	18.26465237	13.10038511	19.17378684	1765.828186	1302.646066	1090.257447	19	14	20	1658	1242	1062	82.37850521
G10090_18998	7.690379943	30.87947919	10.54558276	1777.543572	3644.682028	1618.960447	8	33	11	1669	3475	1577	143.1880274
G10090_19061	18.26465237	25.26502843	2.876068026	820.0770223	766.6942626	281.2905279	19	27	3	770	731	274	40.23241003
G10090_20164	3.845189972	14.0361269	4.793446711	693.3378461	1161.054102	288.4767822	4	15	5	651	1107	281	94.22014437
G10090_20212	37.49060222	33.68670457	41.22364171	1466.553324	1596.318287	1618.960447	39	36	43	1377	1522	1577	41.67998397
G10090_20420	210.5241509	212.4133872	244.4657822	11471.493	12836.62254	8292.937533	219	227	255	10771	12239	8078	48.84962344
G10090_2060	323.9572551	153.4616542	51.76922448	80007.56632	63795.04586	38109.73349	337	164	54	75122	60825	37122	343.765959
G10090_21609	289.3505454	246.1000917	238.7136462	8245.501697	7596.671059	6317.744191	301	263	249	7742	7243	6154	28.62674082
G10090_21977	72.09731197	80.47379425	16.29771882	6869.476355	7216.99483	5152.544377	75	86	17	6450	6881	5019	113.9075859
G10090_22095	129.7751615	80.47379425	30.67805895	14752.86612	9995.343806	8182.063894	135	86	32	13852	9530	7970	136.6848844
G10090_2280	37.49060222	13.10038511	12.46296145	681.6224601	629.2976163	493.7983354	39	14	13	640	600	481	28.65960956
G10090_23580	24.99373482	44.9156061	35.47150566	665.6469337	985.8995989	911.6276961	26	48	37	625	940	888	24.29801253
G10090_25739	0.961297493	4.678708968	3.834757369	422.8189323	711.1063065	243.3060405	1	5	4	397	678	237	144.8882297
G10090_26551	24.03243732	47.72283148	12.46296145	5778.880419	8918.196053	3571.568418	25	51	13	5426	8503	3479	216.78281
G10090_29396	49.02617214	35.55818816	25.88461224	1498.504377	1087.636047	564.6342712	51	38	27	1407	1037	550	28.52374979
G10090_29841	108.6266167	92.63843757	11.50427211	34666.89231	32894.43524	8627.611665	113	99	12	32550	31363	8404	358.0673024
G10090_30722	1.922594986	5.614450762	2.876068026	118.2188954	151.0314279	112.9268542	2	6	3	111	144	110	36.53831805
G10090_30788	17.30335487	1.871483587	0	5668.11677	1404.382514	871.5899932	18	2	0	5322	1339	849	414.5204102
G10090_32309	8.651677436	20.58631946	3.834757369	315.2503878	350.3090064	241.252825	9	22	4	296	334	235	27.32548713
G10090_32612	5.767784957	18.71483587	10.54558276	864.8084962	1387.601244	632.3903838	6	20	11	812	1323	616	82.14412917
G10090_3471	3.845189972	25.26502843	11.50427211	429.2091428	621.9558108	165.2838503	4	27	12	403	593	161	29.89761163
G10090_4537	35.56800724	31.81522098	11.50427211	36793.76739	35125.29529	16973.9328	37	34	12	34547	33490	16534	1126.378514
G10090_545	0.961297493	11.22890152	1.917378684	906.3448649	2129.123602	724.7850827	1	12	2	851	2030	706	265.7721861
G10090_6461	596.0044456	458.5134789	853.2335145	14.91049131	18.87892849	13.34590096	620	490	890	14	18	13	0.02469747
G10090_69	2.883892479	5.614450762	0.958689342	330.1608791	256.9631933	146.8049105	3	6	1	310	245	143	77.22904912
