# Supplementary material for: Guanylate-Binding protein 2b regulates the AMPK/mTOR/ULK1 signalling pathway to induce autophagy during Mycobacterium bovis infection
Source: Virulence. 2022 May 21;13(1):875–89. doi: 10.1080/21505594.2022.2073024 (PMC9132469; doi:10.1080/21505594.2022.2073024)
Supplement: Supplemental Material [file KVIR_A_2073024_SM9990.zip › Supplementary table 5 .pdf]

Gbp2b	Gene Symbol	style	Pearson R	P-Value	path_id	path_name	enrichment	pvalue	FDR
Gbp2b	Acacb	down	-0.8371	0.0007	04152	AMPK signaling pathway	6.932365775	5.57423E-10	5.75404E-09
Gbp2b	Acvr2b	down	-0.9029	0.0001	04060	Cytokine-cytokine receptor interaction	4.140491504	1.27647E-08	8.50978E-08
Gbp2b	Acvr2b	down	-0.9029	0.0001	04350	TGF-beta signaling pathway	4.597253093	0.000629199	0.001417912
Gbp2b	Adora2a	up	0.9268	0	04020	Calcium signaling pathway	6.106930037	4.54499E-14	2.31648E-13
Gbp2b	Adora2a	up	0.9268	0	04024	cAMP signaling pathway	4.701405642	5.70498E-08	1.72286E-07
Gbp2b	Aph1c	down	-0.93	0	04330	Notch signaling pathway	6.290480055	0.000473463	0.001089988
Gbp2b	Atp6v1e1	up	0.8743	0.0002	04150	mTOR signaling pathway	4.211675888	6.18202E-05	0.000121337
Gbp2b	Bcl2a1a	up	0.9398	0	04064	NF-kappa B signaling pathway	18.29070671	7.1923E-37	3.72573E-35
Gbp2b	Bcl2a1a	up	0.9398	0	04210	Apoptosis	10.4053169	7.2193E-20	6.518E-19
Gbp2b	Bcl2a1b	up	0.9577	0	04064	NF-kappa B signaling pathway	18.29070671	7.1923E-37	3.72573E-35
Gbp2b	Bcl2a1b	up	0.9577	0	04210	Apoptosis	10.4053169	7.2193E-20	6.518E-19
Gbp2b	Bcl2a1c	up	0.9674	0	04064	NF-kappa B signaling pathway	18.29070671	7.1923E-37	3.72573E-35
Gbp2b	Bcl2a1c	up	0.9674	0	04210	Apoptosis	10.4053169	7.2193E-20	6.518E-19
Gbp2b	Bcl2a1d	up	0.9097	0	04064	NF-kappa B signaling pathway	18.29070671	7.1923E-37	3.72573E-35
Gbp2b	Bcl2a1d	up	0.9097	0	04210	Apoptosis	10.4053169	7.2193E-20	6.518E-19
Gbp2b	Birc3	up	0.8939	0.0001	04064	NF-kappa B signaling pathway	18.29070671	7.1923E-37	3.72573E-35
Gbp2b	Birc3	up	0.8939	0.0001	04210	Apoptosis	10.4053169	7.2193E-20	6.518E-19
Gbp2b	Birc3	up	0.8939	0.0001	04390	Hippo signaling pathway	4.828672992	2.51973E-06	6.17236E-06
Gbp2b	Birc3	up	0.8939	0.0001	04621	NOD-like receptor signaling pathway	11.73680295	8.2532E-37	3.72573E-35
Gbp2b	Birc3	up	0.8939	0.0001	04668	TNF signaling pathway	23.25739605	7.13226E-57	1.1269E-54
Gbp2b	Capn2	down	-0.9151	0	04210	Apoptosis	4.638568276	2.13642E-05	6.63742E-05
Gbp2b	Casp3	down	-0.859	0.0003	04010	MAPK signaling pathway	7.427534759	5.8609E-25	6.25163E-23
Gbp2b	Casp3	down	-0.859	0.0003	04115	p53 signaling pathway	9.435720083	9.21235E-10	8.93318E-09
Gbp2b	Casp3	down	-0.859	0.0003	04210	Apoptosis	4.638568276	2.13642E-05	6.63742E-05
Gbp2b	Casp3	down	-0.859	0.0003	05152	Tuberculosis	3.504696031	0.00041322	0.000979484
Gbp2b	Casp4	up	0.8939	0.0001	04621	NOD-like receptor signaling pathway	11.73680295	8.2532E-37	3.72573E-35
Gbp2b	Casp7	up	0.8541	0.0004	04210	Apoptosis	10.4053169	7.2193E-20	6.518E-19
Gbp2b	Casp7	up	0.8541	0.0004	04668	TNF signaling pathway	23.25739605	7.13226E-57	1.1269E-54
Gbp2b	Castor2	down	-0.8804	0.0002	04150	mTOR signaling pathway	4.043880035	9.4213E-05	0.00024313
Gbp2b	Ccl2	up	0.8053	0.0016	04060	Cytokine-cytokine receptor interaction	12.41941286	1.15099E-55	1.21237E-53
Gbp2b	Ccl2	up	0.8053	0.0016	04621	NOD-like receptor signaling pathway	11.73680295	8.2532E-37	3.72573E-35
Gbp2b	Ccl2	up	0.8053	0.0016	04657	IL-17 signaling pathway	18.32773243	3.70837E-32	1.06531E-30
Gbp2b	Ccl2	up	0.8053	0.0016	04668	TNF signaling pathway	23.25739605	7.13226E-57	1.1269E-54
Gbp2b	Ccl22	up	0.9522	0	04060	Cytokine-cytokine receptor interaction	12.41941286	1.15099E-55	1.21237E-53
Gbp2b	Cebpb	up	0.9162	0	04657	IL-17 signaling pathway	18.32773243	3.70837E-32	1.06531E-30
Gbp2b	Cebpb	up	0.9162	0	04668	TNF signaling pathway	23.25739605	7.13226E-57	1.1269E-54
Gbp2b	Cebpb	up	0.9162	0	05152	Tuberculosis	11.2311357	2.15881E-29	4.54789E-28
Gbp2b	Cflar	up	0.9013	0.0001	04064	NF-kappa B signaling pathway	18.29070671	7.1923E-37	3.72573E-35
Gbp2b	Cflar	up	0.9013	0.0001	04140	Autophagy - animal	4.761024916	1.6243E-05	3.41408E-05
Gbp2b	Cflar	up	0.9013	0.0001	04210	Apoptosis	10.4053169	7.2193E-20	6.518E-19
Gbp2b	Cflar	up	0.9013	0.0001	04668	TNF signaling pathway	23.25739605	7.13226E-57	1.1269E-54
Gbp2b	Cpt1a	down	-0.9332	0	04152	AMPK signaling pathway	6.932365775	5.57423E-10	5.75404E-09

Gbp2b	Ctsc	up	0.9761	0	04210	Apoptosis	10.4053169	7.2193E-20	6.518E-19
Gbp2b	Cxcl1	up	0.8342	0.0007	04060	Cytokine-cytokine receptor interaction	12.41941286	1.15099E-55	1.21237E-53
Gbp2b	Cxcl1	up	0.8342	0.0007	04064	NF-kappa B signaling pathway	18.29070671	7.1923E-37	3.72573E-35
Gbp2b	Cxcl1	up	0.8342	0.0007	04621	NOD-like receptor signaling pathway	11.73680295	8.2532E-37	3.72573E-35
Gbp2b	Cxcl1	up	0.8342	0.0007	04657	IL-17 signaling pathway	18.32773243	3.70837E-32	1.06531E-30
Gbp2b	Cxcl1	up	0.8342	0.0007	04668	TNF signaling pathway	23.25739605	7.13226E-57	1.1269E-54
Gbp2b	Cxcl2	up	0.8021	0.0017	04060	Cytokine-cytokine receptor interaction	12.41941286	1.15099E-55	1.21237E-53
Gbp2b	Cxcl2	up	0.8021	0.0017	04064	NF-kappa B signaling pathway	18.29070671	7.1923E-37	3.72573E-35
Gbp2b	Cxcl2	up	0.8021	0.0017	04621	NOD-like receptor signaling pathway	11.73680295	8.2532E-37	3.72573E-35
Gbp2b	Cxcl2	up	0.8021	0.0017	04657	IL-17 signaling pathway	18.32773243	3.70837E-32	1.06531E-30
Gbp2b	Cxcl2	up	0.8021	0.0017	04668	TNF signaling pathway	23.25739605	7.13226E-57	1.1269E-54
Gbp2b	Cxcl3	up	0.834	0.0007	04060	Cytokine-cytokine receptor interaction	12.41941286	1.15099E-55	1.21237E-53
Gbp2b	Cxcl3	up	0.834	0.0007	04064	NF-kappa B signaling pathway	18.29070671	7.1923E-37	3.72573E-35
Gbp2b	Cxcl3	up	0.834	0.0007	04621	NOD-like receptor signaling pathway	11.73680295	8.2532E-37	3.72573E-35
Gbp2b	Cxcl3	up	0.834	0.0007	04657	IL-17 signaling pathway	18.32773243	3.70837E-32	1.06531E-30
Gbp2b	Cxcl3	up	0.834	0.0007	04668	TNF signaling pathway	23.25739605	7.13226E-57	1.1269E-54
Gbp2b	Cycs	up	0.9106	0	04115	p53 signaling pathway	8.423351775	7.08757E-08	2.1129E-07
Gbp2b	Cycs	up	0.9106	0	04210	Apoptosis	10.4053169	7.2193E-20	6.518E-19
Gbp2b	Cycs	up	0.9106	0	05152	Tuberculosis	11.2311357	2.15881E-29	4.54789E-28
Gbp2b	Ebi3	up	0.9118	0	04060	Cytokine-cytokine receptor interaction	12.41941286	1.15099E-55	1.21237E-53
Gbp2b	Eea1	up	0.9381	0	05152	Tuberculosis	11.2311357	2.15881E-29	4.54789E-28
Gbp2b	Eef2k	down	-0.8826	0.0001	04152	AMPK signaling pathway	6.932365775	5.57423E-10	5.75404E-09
Gbp2b	Eif4e	up	0.9063	0	04066	HIF-1 signaling pathway	13.30002912	1.36396E-24	1.95914E-23
Gbp2b	Eif4e	up	0.9063	0	04150	mTOR signaling pathway	4.211675888	6.18202E-05	0.000121337
Gbp2b	Eif4e	up	0.9063	0	04151	PI3K-Akt signaling pathway	7.88369414	3.29921E-32	1.04255E-30
Gbp2b	Ets2	up	0.9178	0	04014	Ras signaling pathway	6.75320444	3.18253E-16	1.97192E-15
Gbp2b	Fas	up	0.9019	0.0001	04010	MAPK signaling pathway	9.110972328	8.74058E-34	3.45253E-32
Gbp2b	Fas	up	0.9019	0.0001	04060	Cytokine-cytokine receptor interaction	12.41941286	1.15099E-55	1.21237E-53
Gbp2b	Fas	up	0.9019	0.0001	04115	p53 signaling pathway	8.423351775	7.08757E-08	2.1129E-07
Gbp2b	Fas	up	0.9019	0.0001	04210	Apoptosis	10.4053169	7.2193E-20	6.518E-19
Gbp2b	Fas	up	0.9019	0.0001	04668	TNF signaling pathway	23.25739605	7.13226E-57	1.1269E-54
Gbp2b	Fzd1	up	0.8155	0.0012	04150	mTOR signaling pathway	4.211675888	6.18202E-05	0.000121337
Gbp2b	Fzd1	up	0.8155	0.0012	04390	Hippo signaling pathway	4.828672992	2.51973E-06	6.17236E-06
Gbp2b	Gbp2	up	0.9721	0	04621	NOD-like receptor signaling pathway	11.73680295	8.2532E-37	3.72573E-35
Gbp2b	Gbp2b	up	1	0	04621	NOD-like receptor signaling pathway	11.73680295	8.2532E-37	3.72573E-35
Gbp2b	Gbp3	up	0.9148	0	04621	NOD-like receptor signaling pathway	11.73680295	8.2532E-37	3.72573E-35
Gbp2b	Gbp5	up	0.8541	0.0004	04621	NOD-like receptor signaling pathway	11.73680295	8.2532E-37	3.72573E-35
Gbp2b	Gbp7	up	0.9428	0	04621	NOD-like receptor signaling pathway	11.73680295	8.2532E-37	3.72573E-35
Gbp2b	Icam1	up	0.9374	0	04064	NF-kappa B signaling pathway	18.29070671	7.1923E-37	3.72573E-35
Gbp2b	Icam1	up	0.9374	0	04668	TNF signaling pathway	23.25739605	7.13226E-57	1.1269E-54
Gbp2b	Il16	down	-0.918	0	04060	Cytokine-cytokine receptor interaction	4.140491504	1.27647E-08	8.50978E-08
Gbp2b	Il6	up	0.8179	0.0012	04060	Cytokine-cytokine receptor interaction	12.41941286	1.15099E-55	1.21237E-53
Gbp2b	Il6	up	0.8179	0.0012	04066	HIF-1 signaling pathway	13.30002912	1.36396E-24	1.95914E-23

Gbp2b	Il6	up	0.8179	0.0012	04151	PI3K-Akt signaling pathway	7.88369414	3.29921E-32	1.04255E-30
Gbp2b	Il6	up	0.8179	0.0012	04620	Toll-like receptor signaling pathway	14.15123098	7.86568E-24	1.03565E-22
Gbp2b	Il6	up	0.8179	0.0012	04621	NOD-like receptor signaling pathway	11.73680295	8.2532E-37	3.72573E-35
Gbp2b	Il6	up	0.8179	0.0012	04630	JAK-STAT signaling pathway	10.76594073	7.5243E-26	1.13223E-24
Gbp2b	Il6	up	0.8179	0.0012	04657	IL-17 signaling pathway	18.32773243	3.70837E-32	1.06531E-30
Gbp2b	Il6	up	0.8179	0.0012	04659	Th17 cell differentiation	11.17713986	3.29888E-17	2.21797E-16
Gbp2b	Il6	up	0.8179	0.0012	04668	TNF signaling pathway	23.25739605	7.13226E-57	1.1269E-54
Gbp2b	Il6	up	0.8179	0.0012	05152	Tuberculosis	11.2311357	2.15881E-29	4.54789E-28
Gbp2b	Irak2	up	0.9233	0	05152	Tuberculosis	11.2311357	2.15881E-29	4.54789E-28
Gbp2b	Irf1	up	0.841	0.0006	04668	TNF signaling pathway	23.25739605	7.13226E-57	1.1269E-54
Gbp2b	Itgb8	up	0.9083	0	04151	PI3K-Akt signaling pathway	7.88369414	3.29921E-32	1.04255E-30
Gbp2b	Junb	up	0.9226	0	04668	TNF signaling pathway	23.25739605	7.13226E-57	1.1269E-54
Gbp2b	Lrp5	down	-0.8187	0.0011	04150	mTOR signaling pathway	4.043880035	9.4213E-05	0.00024313
Gbp2b	Map3k4	down	-0.9012	0.0001	04010	MAPK signaling pathway	7.427534759	5.8609E-25	6.25163E-23
Gbp2b	Mknk2	down	-0.9571	0	04010	MAPK signaling pathway	7.427534759	5.8609E-25	6.25163E-23
Gbp2b	Mknk2	down	-0.9571	0	04066	HIF-1 signaling pathway	6.810745323	7.5931E-09	5.39954E-08
Gbp2b	Mlkl	up	0.9175	0	04668	TNF signaling pathway	23.25739605	7.13226E-57	1.1269E-54
Gbp2b	Mtor	down, up	-0.8421	0.0006	04066	HIF-1 signaling pathway	6.810745323	7.5931E-09	5.39954E-08
Gbp2b	Mtor	down, up	-0.8421	0.0006	04066	HIF-1 signaling pathway	13.30002912	1.36396E-24	1.95914E-23
Gbp2b	Mtor	down, up	-0.8421	0.0006	04140	Autophagy - animal	3.16477568	0.009387513	0.016150559
Gbp2b	Mtor	down, up	-0.8421	0.0006	04140	Autophagy - animal	4.761024916	1.6243E-05	3.41408E-05
Gbp2b	Mtor	down, up	-0.8421	0.0006	04150	mTOR signaling pathway	4.043880035	9.4213E-05	0.00024313
Gbp2b	Mtor	down, up	-0.8421	0.0006	04150	mTOR signaling pathway	4.211675888	6.18202E-05	0.000121337
Gbp2b	Mtor	down, up	-0.8421	0.0006	04151	PI3K-Akt signaling pathway	4.731001713	2.05622E-13	5.48325E-12
Gbp2b	Mtor	down, up	-0.8421	0.0006	04151	PI3K-Akt signaling pathway	7.88369414	3.29921E-32	1.04255E-30
Gbp2b	Mtor	down, up	-0.8421	0.0006	04152	AMPK signaling pathway	5.214455861	5.84474E-06	1.33836E-05
Gbp2b	Mtor	down, up	-0.8421	0.0006	04152	AMPK signaling pathway	6.932365775	5.57423E-10	5.75404E-09
Gbp2b	Mtor	down, up	-0.8421	0.0006	04630	JAK-STAT signaling pathway	4.307091162	1.05166E-05	3.58012E-05
Gbp2b	Mtor	down, up	-0.8421	0.0006	04630	JAK-STAT signaling pathway	10.76594073	7.5243E-26	1.13223E-24
Gbp2b	Mtor	down, up	-0.8421	0.0006	04659	Th17 cell differentiation	8.398827766	2.02734E-11	4.05469E-10
Gbp2b	Mtor	down, up	-0.8421	0.0006	04659	Th17 cell differentiation	11.17713986	3.29888E-17	2.21797E-16
Gbp2b	Nfatc1	down	-0.9181	0	04010	MAPK signaling pathway	7.427534759	5.8609E-25	6.25163E-23
Gbp2b	Nfatc1	down	-0.9181	0	04024	cAMP signaling pathway	4.288393712	5.50128E-07	2.37893E-06
Gbp2b	Nfatc1	down	-0.9181	0	04658	Th1 and Th2 cell differentiation	8.2715728	1.49009E-09	1.32453E-08
Gbp2b	Nfatc1	down	-0.9181	0	04659	Th17 cell differentiation	8.398827766	2.02734E-11	4.05469E-10
Gbp2b	Nfkb1	up	0.8531	0.0004	04010	MAPK signaling pathway	9.110972328	8.74058E-34	3.45253E-32
Gbp2b	Nfkb1	up	0.8531	0.0004	04014	Ras signaling pathway	6.75320444	3.18253E-16	1.97192E-15
Gbp2b	Nfkb1	up	0.8531	0.0004	04024	cAMP signaling pathway	4.701405642	5.70498E-08	1.72286E-07
Gbp2b	Nfkb1	up	0.8531	0.0004	04064	NF-kappa B signaling pathway	18.29070671	7.1923E-37	3.72573E-35
Gbp2b	Nfkb1	up	0.8531	0.0004	04066	HIF-1 signaling pathway	13.30002912	1.36396E-24	1.95914E-23
Gbp2b	Nfkb1	up	0.8531	0.0004	04151	PI3K-Akt signaling pathway	7.88369414	3.29921E-32	1.04255E-30
Gbp2b	Nfkb1	up	0.8531	0.0004	04210	Apoptosis	10.4053169	7.2193E-20	6.518E-19
Gbp2b	Nfkb1	up	0.8531	0.0004	04620	Toll-like receptor signaling pathway	14.15123098	7.86568E-24	1.03565E-22

Gbp2b	Nfkb1	up	0.8531	0.0004	04621	NOD-like receptor signaling pathway	11.73680295	8.2532E-37	3.72573E-35
Gbp2b	Nfkb1	up	0.8531	0.0004	04622	RIG-I-like receptor signaling pathway	12.27402687	1.16908E-13	5.68354E-13
Gbp2b	Nfkb1	up	0.8531	0.0004	04657	IL-17 signaling pathway	18.32773243	3.70837E-32	1.06531E-30
Gbp2b	Nfkb1	up	0.8531	0.0004	04658	Th1 and Th2 cell differentiation	10.91206934	3.7305E-14	1.96473E-13
Gbp2b	Nfkb1	up	0.8531	0.0004	04659	Th17 cell differentiation	11.17713986	3.29888E-17	2.21797E-16
Gbp2b	Nfkb1	up	0.8531	0.0004	04668	TNF signaling pathway	23.25739605	7.13226E-57	1.1269E-54
Gbp2b	Nfkb1	up	0.8531	0.0004	05152	Tuberculosis	11.2311357	2.15881E-29	4.54789E-28
Gbp2b	Nfkb2	up	0.942	0	04010	MAPK signaling pathway	9.110972328	8.74058E-34	3.45253E-32
Gbp2b	Nfkb2	up	0.942	0	04064	NF-kappa B signaling pathway	18.29070671	7.1923E-37	3.72573E-35
Gbp2b	Nfkbia	up	0.9544	0	04024	cAMP signaling pathway	4.701405642	5.70498E-08	1.72286E-07
Gbp2b	Nfkbia	up	0.9544	0	04064	NF-kappa B signaling pathway	18.29070671	7.1923E-37	3.72573E-35
Gbp2b	Nfkbia	up	0.9544	0	04210	Apoptosis	10.4053169	7.2193E-20	6.518E-19
Gbp2b	Nfkbia	up	0.9544	0	04620	Toll-like receptor signaling pathway	14.15123098	7.86568E-24	1.03565E-22
Gbp2b	Nfkbia	up	0.9544	0	04621	NOD-like receptor signaling pathway	11.73680295	8.2532E-37	3.72573E-35
Gbp2b	Nfkbia	up	0.9544	0	04622	RIG-I-like receptor signaling pathway	12.27402687	1.16908E-13	5.68354E-13
Gbp2b	Nfkbia	up	0.9544	0	04657	IL-17 signaling pathway	18.32773243	3.70837E-32	1.06531E-30
Gbp2b	Nfkbia	up	0.9544	0	04658	Th1 and Th2 cell differentiation	10.91206934	3.7305E-14	1.96473E-13
Gbp2b	Nfkbia	up	0.9544	0	04659	Th17 cell differentiation	11.17713986	3.29888E-17	2.21797E-16
Gbp2b	Nfkbia	up	0.9544	0	04668	TNF signaling pathway	23.25739605	7.13226E-57	1.1269E-54
Gbp2b	Nfkbib	up	0.9005	0.0001	04621	NOD-like receptor signaling pathway	11.73680295	8.2532E-37	3.72573E-35
Gbp2b	Nfkbib	up	0.9005	0.0001	04622	RIG-I-like receptor signaling pathway	12.27402687	1.16908E-13	5.68354E-13
Gbp2b	Nfkbib	up	0.9005	0.0001	04658	Th1 and Th2 cell differentiation	10.91206934	3.7305E-14	1.96473E-13
Gbp2b	Nfkbib	up	0.9005	0.0001	04659	Th17 cell differentiation	11.17713986	3.29888E-17	2.21797E-16
Gbp2b	Nlrp3	up	0.9586	0	04621	NOD-like receptor signaling pathway	11.73680295	8.2532E-37	3.72573E-35
Gbp2b	Nod2	up	0.9745	0	04621	NOD-like receptor signaling pathway	11.73680295	8.2532E-37	3.72573E-35
Gbp2b	Nod2	up	0.9745	0	04668	TNF signaling pathway	23.25739605	7.13226E-57	1.1269E-54
Gbp2b	Nod2	up	0.9745	0	05152	Tuberculosis	11.2311357	2.15881E-29	4.54789E-28
Gbp2b	Nos2	up	0.871	0.0002	04020	Calcium signaling pathway	6.106930037	4.54499E-14	2.31648E-13
Gbp2b	Nos2	up	0.871	0.0002	04066	HIF-1 signaling pathway	13.30002912	1.36396E-24	1.95914E-23
Gbp2b	Nos2	up	0.871	0.0002	05152	Tuberculosis	11.2311357	2.15881E-29	4.54789E-28
Gbp2b	Pfkfb2	down	-0.922	0	04152	AMPK signaling pathway	6.932365775	5.57423E-10	5.75404E-09
Gbp2b	Pik3cg	up	0.9403	0	04151	PI3K-Akt signaling pathway	7.88369414	3.29921E-32	1.04255E-30
Gbp2b	Pik3r2	down	-0.9172	0	04014	Ras signaling pathway	6.902484888	1.55422E-17	9.94698E-16
Gbp2b	Pik3r2	down	-0.9172	0	04024	cAMP signaling pathway	4.288393712	5.50128E-07	2.37893E-06
Gbp2b	Pik3r2	down	-0.9172	0	04066	HIF-1 signaling pathway	6.810745323	7.5931E-09	5.39954E-08
Gbp2b	Pik3r2	down	-0.9172	0	04140	Autophagy - animal	3.16477568	0.009387513	0.016150559
Gbp2b	Pik3r2	down	-0.9172	0	04150	mTOR signaling pathway	4.043880035	9.4213E-05	0.00024313
Gbp2b	Pik3r2	down	-0.9172	0	04151	PI3K-Akt signaling pathway	4.731001713	2.05622E-13	5.48325E-12
Gbp2b	Pik3r2	down	-0.9172	0	04152	AMPK signaling pathway	6.932365775	5.57423E-10	5.75404E-09
Gbp2b	Pik3r2	down	-0.9172	0	04210	Apoptosis	4.638568276	2.13642E-05	6.63742E-05
Gbp2b	Pik3r2	down	-0.9172	0	04620	Toll-like receptor signaling pathway	3.39685923	0.0189456	0.030162149
Gbp2b	Pik3r2	down	-0.9172	0	04630	JAK-STAT signaling pathway	4.307091162	1.05166E-05	3.58012E-05
Gbp2b	Ppp2cb	up	0.9001	0.0001	04140	Autophagy - animal	4.761024916	1.6243E-05	3.41408E-05

Gbp2b	Ppp2cb	up	0.9001	0.0001	04151	PI3K-Akt signaling pathway	7.88369414	3.29921E-32	1.04255E-30
Gbp2b	Ppp2cb	up	0.9001	0.0001	04152	AMPK signaling pathway	5.214455861	5.84474E-06	1.33836E-05
Gbp2b	Ppp2cb	up	0.9001	0.0001	04350	TGF-beta signaling pathway	7.980017471	2.6132E-09	9.07441E-09
Gbp2b	Ppp2cb	up	0.9001	0.0001	04390	Hippo signaling pathway	4.828672992	2.51973E-06	6.17236E-06
Gbp2b	Ppp3cc	up	0.9069	0	04010	MAPK signaling pathway	9.110972328	8.74058E-34	3.45253E-32
Gbp2b	Ppp3cc	up	0.9069	0	04020	Calcium signaling pathway	6.106930037	4.54499E-14	2.31648E-13
Gbp2b	Ppp3cc	up	0.9069	0	04658	Th1 and Th2 cell differentiation	10.91206934	3.7305E-14	1.96473E-13
Gbp2b	Ppp3cc	up	0.9069	0	04659	Th17 cell differentiation	11.17713986	3.29888E-17	2.21797E-16
Gbp2b	Ppp3cc	up	0.9069	0	05152	Tuberculosis	11.2311357	2.15881E-29	4.54789E-28
Gbp2b	Prkag2	down	-0.9268	0	04152	AMPK signaling pathway	6.932365775	5.57423E-10	5.75404E-09
Gbp2b	Rab10	up	0.8154	0.0012	04152	AMPK signaling pathway	5.214455861	5.84474E-06	1.33836E-05
Gbp2b	Rapgef2	up	0.91	0	04010	MAPK signaling pathway	9.110972328	8.74058E-34	3.45253E-32
Gbp2b	Rasa3	down	-0.9351	0	04014	Ras signaling pathway	6.902484888	1.55422E-17	9.94698E-16
Gbp2b	Ripk2	up	0.8072	0.0015	04621	NOD-like receptor signaling pathway	11.73680295	8.2532E-37	3.72573E-35
Gbp2b	Ripk2	up	0.8072	0.0015	05152	Tuberculosis	11.2311357	2.15881E-29	4.54789E-28
Gbp2b	Rps6ka2	up	0.8397	0.0006	04010	MAPK signaling pathway	9.110972328	8.74058E-34	3.45253E-32
Gbp2b	Rps6ka2	up	0.8397	0.0006	04150	mTOR signaling pathway	4.211675888	6.18202E-05	0.000121337
Gbp2b	Sav1	up	0.9063	0	04390	Hippo signaling pathway	4.828672992	2.51973E-06	6.17236E-06
Gbp2b	Sav1	up	0.9063	0	04392	Hippo signaling pathway - multiple species	7.4874238	0.007451944	0.011003806
Gbp2b	Socs3	up	0.9053	0.0001	04630	JAK-STAT signaling pathway	10.76594073	7.5243E-26	1.13223E-24
Gbp2b	Socs3	up	0.9053	0.0001	04668	TNF signaling pathway	23.25739605	7.13226E-57	1.1269E-54
Gbp2b	Syk	up	0.9246	0	04064	NF-kappa B signaling pathway	18.29070671	7.1923E-37	3.72573E-35
Gbp2b	Syk	up	0.9246	0	04151	PI3K-Akt signaling pathway	7.88369414	3.29921E-32	1.04255E-30
Gbp2b	Syk	up	0.9246	0	05152	Tuberculosis	11.2311357	2.15881E-29	4.54789E-28
Gbp2b	Tank	up	0.9276	0	04140	Autophagy - animal	4.761024916	1.6243E-05	3.41408E-05
Gbp2b	Tank	up	0.9276	0	04621	NOD-like receptor signaling pathway	11.73680295	8.2532E-37	3.72573E-35
Gbp2b	Tank	up	0.9276	0	04622	RIG-I-like receptor signaling pathway	12.27402687	1.16908E-13	5.68354E-13
Gbp2b	Tbk1	up	0.8863	0.0001	04014	Ras signaling pathway	6.75320444	3.18253E-16	1.97192E-15
Gbp2b	Tbk1	up	0.8863	0.0001	04140	Autophagy - animal	4.761024916	1.6243E-05	3.41408E-05
Gbp2b	Tbk1	up	0.8863	0.0001	04620	Toll-like receptor signaling pathway	14.15123098	7.86568E-24	1.03565E-22
Gbp2b	Tbk1	up	0.8863	0.0001	04621	NOD-like receptor signaling pathway	11.73680295	8.2532E-37	3.72573E-35
Gbp2b	Tbk1	up	0.8863	0.0001	04622	RIG-I-like receptor signaling pathway	12.27402687	1.16908E-13	5.68354E-13
Gbp2b	Tbk1	up	0.8863	0.0001	04657	IL-17 signaling pathway	18.32773243	3.70837E-32	1.06531E-30
Gbp2b	Tiam1	down	-0.931	0	04014	Ras signaling pathway	6.902484888	1.55422E-17	9.94698E-16
Gbp2b	Tiam1	down	-0.931	0	04024	cAMP signaling pathway	4.288393712	5.50128E-07	2.37893E-06
Gbp2b	Tnf	up	0.8337	0.0008	04010	MAPK signaling pathway	9.110972328	8.74058E-34	3.45253E-32
Gbp2b	Tnf	up	0.8337	0.0008	04060	Cytokine-cytokine receptor interaction	12.41941286	1.15099E-55	1.21237E-53
Gbp2b	Tnf	up	0.8337	0.0008	04064	NF-kappa B signaling pathway	18.29070671	7.1923E-37	3.72573E-35
Gbp2b	Tnf	up	0.8337	0.0008	04150	mTOR signaling pathway	4.211675888	6.18202E-05	0.000121337
Gbp2b	Tnf	up	0.8337	0.0008	04210	Apoptosis	10.4053169	7.2193E-20	6.518E-19
Gbp2b	Tnf	up	0.8337	0.0008	04350	TGF-beta signaling pathway	7.980017471	2.6132E-09	9.07441E-09
Gbp2b	Tnf	up	0.8337	0.0008	04620	Toll-like receptor signaling pathway	14.15123098	7.86568E-24	1.03565E-22
Gbp2b	Tnf	up	0.8337	0.0008	04621	NOD-like receptor signaling pathway	11.73680295	8.2532E-37	3.72573E-35

Gbp2b	Tnf	up	0.8337	0.0008	04622	RIG-I-like receptor signaling pathway	12.27402687	1.16908E-13	5.68354E-13
Gbp2b	Tnf	up	0.8337	0.0008	04657	IL-17 signaling pathway	18.32773243	3.70837E-32	1.06531E-30
Gbp2b	Tnf	up	0.8337	0.0008	04668	TNF signaling pathway	23.25739605	7.13226E-57	1.1269E-54
Gbp2b	Tnf	up	0.8337	0.0008	05152	Tuberculosis	11.2311357	2.15881E-29	4.54789E-28
Gbp2b	Tnfaip3	up	0.9282	0	04064	NF-kappa B signaling pathway	18.29070671	7.1923E-37	3.72573E-35
Gbp2b	Tnfaip3	up	0.9282	0	04621	NOD-like receptor signaling pathway	11.73680295	8.2532E-37	3.72573E-35
Gbp2b	Tnfaip3	up	0.9282	0	04657	IL-17 signaling pathway	18.32773243	3.70837E-32	1.06531E-30
Gbp2b	Tnfaip3	up	0.9282	0	04668	TNF signaling pathway	23.25739605	7.13226E-57	1.1269E-54
Gbp2b	Tnfsf15	up	0.945	0	04060	Cytokine-cytokine receptor interaction	12.41941286	1.15099E-55	1.21237E-53
Gbp2b	Traf1	up	0.9545	0	04064	NF-kappa B signaling pathway	18.29070671	7.1923E-37	3.72573E-35
Gbp2b	Traf1	up	0.9545	0	04210	Apoptosis	10.4053169	7.2193E-20	6.518E-19
Gbp2b	Traf1	up	0.9545	0	04668	TNF signaling pathway	23.25739605	7.13226E-57	1.1269E-54
Gbp2b	Traf2	up	0.8636	0.0003	04010	MAPK signaling pathway	9.110972328	8.74058E-34	3.45253E-32
Gbp2b	Traf2	up	0.8636	0.0003	04064	NF-kappa B signaling pathway	18.29070671	7.1923E-37	3.72573E-35
Gbp2b	Traf2	up	0.8636	0.0003	04210	Apoptosis	10.4053169	7.2193E-20	6.518E-19
Gbp2b	Traf2	up	0.8636	0.0003	04621	NOD-like receptor signaling pathway	11.73680295	8.2532E-37	3.72573E-35
Gbp2b	Traf2	up	0.8636	0.0003	04622	RIG-I-like receptor signaling pathway	12.27402687	1.16908E-13	5.68354E-13
Gbp2b	Traf2	up	0.8636	0.0003	04657	IL-17 signaling pathway	18.32773243	3.70837E-32	1.06531E-30
Gbp2b	Traf2	up	0.8636	0.0003	04668	TNF signaling pathway	23.25739605	7.13226E-57	1.1269E-54
Gbp2b	Traf5	up	0.8223	0.001	04064	NF-kappa B signaling pathway	18.29070671	7.1923E-37	3.72573E-35
Gbp2b	Traf5	up	0.8223	0.001	04621	NOD-like receptor signaling pathway	11.73680295	8.2532E-37	3.72573E-35
Gbp2b	Traf5	up	0.8223	0.001	04657	IL-17 signaling pathway	18.32773243	3.70837E-32	1.06531E-30
Gbp2b	Traf5	up	0.8223	0.001	04668	TNF signaling pathway	23.25739605	7.13226E-57	1.1269E-54
Gbp2b	Ulk1	down	-0.8435	0.0006	04140	Autophagy - animal	3.16477568	0.009387513	0.016150559
Gbp2b	Ulk1	down	-0.8435	0.0006	04150	mTOR signaling pathway	4.043880035	9.4213E-05	0.00024313
Gbp2b	Ulk1	down	-0.8435	0.0006	04152	AMPK signaling pathway	6.932365775	5.57423E-10	5.75404E-09
